# Supplementary material for: Catalytic asymmetric nucleophilic fluorination using BF3·Et2O as fluorine source and activating reagent
Source: Nat Commun. 2021 Jun 25;12:3957. doi: 10.1038/s41467-021-24278-3 (PMC8233348; doi:10.1038/s41467-021-24278-3)
Supplement: Supplementary file 1 — Supplementary Information [file 41467_2021_24278_MOESM1_ESM.pdf]

Supplementary Information

Catalytic Asymmetric Nucleophilic Fluorination Using $BF_3 \cdot Et_2O$ as Fluorine Source and Activating Reagent

Weiwei Zhu, Xiang Zhen, Jingyuan Wu, Yaping Cheng, Junkai An, Xingyu Ma, Jikun Liu, Yuji Qin, Hao Zhu, Jijun Xue and Xianxing Jiang*
School of Pharmaceutical Sciences, Sun Yat-Sen University, Guangzhou, 510006, China
*Email: jiangxx5@mail.sysu.edu.cn

Table of Contents

1. Supplementary Materials and Methods 3
2. Catalysts, Solvent and Temperature Optimization 4
Supplementary Table 1. Optimization of the Reaction Conditions 4
3. Synthesis and Characterization of Catalysts 5
3.1 Synthesis of **CIC1** and **CIC2** 5
3.2 Synthesis of **CIC3-CIC8** 7
3.3 Synthesis of **CIC9** and **CIC10** 8
4. Synthesis and Characterization of Substrates 9
4.1 General Procedure for Synthesis of *N*-cinnamylbenzamides (**1a-28a**, **35a-38a**) 9
4.2 General Procedure for Synthesis of **29a-34a** 16
4.3 General Procedure for Synthesis of **39a** and **40a** 18
4.4 General Procedure for Synthesis of **41a** and **42a** 19
4.5 General Procedure for Synthesis of **43a-54a** 21
5. Procedures for Asymmetric Aminofluorination and Characterization of Products 24
5.1 General Procedure A (Synthesis of **1b-42b**) 24
5.2 General Procedure B (Synthesis of **43b-54b**) 38
6. Chiral HPLC Traces of Aminofluorination Products and Derivatives 43
6.1 HPLC Traces of **1b-42b** 43
Supplementary Figures 1-36. HPLC Traces of **1b-42b** 43
6.2 HPLC Traces of 43b-54b 66
Supplementary Figures 41-52. HPLC Traces of **43b-54b** 66

7. X-Ray Crystallography Information of 4b 72
Supplementary Figure 53. The Crystal Structure of 4b 72
Supplementary Table 2. Crystal Data and Structure Refinement for 4b 72
Supplementary Table 3. Fractional Atomic Coordinates (×104) and Equivalent Isotropic Displacement Parameters (Å2×103) for 4ba 73
Supplementary Table 4. Anisotropic Displacement Parameters ($Å^2×10^3$) for 4b 74
Supplementary Table 5. Bond Lengths for 4b 75
Supplementary Table 6. Bond Angles for 4b 76
Supplementary Table 7. Hydrogen Atom Coordinates ($Å×10^4$) and Isotropic Displacement Parameters ($Å^2×10^3$) for 4b 76
8. DFT Calculations 77
8.1 Method 77
8.2 Cartesian Coordinates of Species Reported in Figure 6. 78
Supplementary Table 8. Cartesian Coordinates of Species Reported in Figure 6 79
9. ^{1}H, ^{13}C, and ^{19}F NMR Spectra of Aminofluorination Products and Derivatives 94
Supplementary Figures 54-215: Spectral Copies of ^{1}H ^{13}C and ^{19}F NMR spectra of **1b-54b**........94
10. Supplementary References 175

1. Supplementary Materials and Methods

Reagents were purchased in analytical grade from commercial suppliers and unless otherwise noted, all commercial reagents were used without further purification. Merck F-254 silica gel plates were used for thin layer analytical chromatography (TLC). Column chromatography purification was carried out using EMD (Merck) Silica Gel 60 (40-63 am).

^{1}H, ^{13}C and ^{19}F NMR spectra were recorded with BRUKER Ascend 400M & BRUKER Ascend 500M at 25°C. The spectra were recorded in $CDCl_3$ as solvent. Multiplicity was described as follows: s (singlet); d (doublet); t (triplet); m (multiplet); dd (doublet of doublets), etc. and coupling constants (*J*) were given in Hz. Chemical shifts are reported in ppm relative to TMS as an internal standard. The peak around delta value of ^{1}H NMR (7.25) is corresponding to deuterated solvent chloroform, and the peaks around delta value of ^{1}H NMR (1.56) is corresponding to water contained in the solvent. The peak around delta value of ^{13}C NMR (76.7~77.4) referenced to the appropriate NMR solvent residual peaks. High-resolution mass spectrometric data were obtained on an Agilent 6210 time-of-flight HPLC/MS spectrometer (ESI-TOF). Chiral HPLC analysis was performed using Thermo Scientific Dionex UltiMate 3000 series quaternary HPLC system with commercially available Phenomenex (4.6 × 250 mm) or CHIRALPAK analytical columns (4.6 × 250 mm).

2. Catalysts, Solvent and Temperature Optimization

Supplementary Table 1. Optimization of the Reaction Conditions[a]

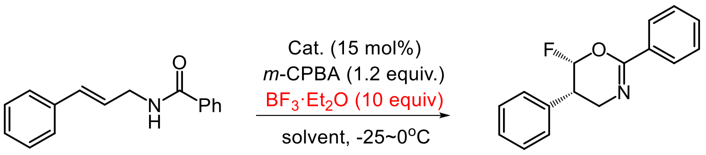

Entry	Cat.	Solvent[b]	T	ee% (dr)[c,d]	Yield[e] (%)
1	IB	DCE	0°C	0 (1:1)	80
2	**CIC3**	DCE	0°C	73 (>20:1)	76
3	**CIC3**	EtOAc	0°C	60 (9:1)	75
4	**CIC3**	C_6H_5F	0°C	60 (8:1)	70
5	**CIC3**	$C_6H_5CF_3$	0°C	70 (10:1)	72
6	**CIC3**	C_6H_5Cl	0°C	69 (10:1)	80
7	**CIC3**	DCM	0°C	70 (8:1)	82
8[f]	**CIC3**	DCE	0°C	60 (10:1)	76
9[g]	**CIC3**	DCE	0°C	65 (10:1)	78
10[h]	**CIC3**	DCE	0°C	43 (8:1)	76
11[h]	**CIC3**	DCM	0°C	60 (10:1)	72
12[i]	**CIC3**	DCE	-25°C	73 (>20:1)	60
13[i]	**CIC4**	DCE	-25°C	74 (>20:1)	56
14[i]	**CIC5**	DCE	-25°C	76 (>20:1)	55
15[i]	**CIC6**	DCE	-25°C	74 (>20:1)	50
16[i]	**CIC7**	DCE	-25°C	72 (15:1)	62
17[i]	**CIC8**	DCE	-25°C	35 (10:1)	62
18[i]	**CIC9**	DCE	-25°C	60 (9:1)	60
19[i]	**CIC10**	DCE	-25°C	62 (10:1)	56
20	**CIC1**	DCE	0°C	80 (>20:1)	75
21[i]	**CIC1**	DCE	-25°C	86 (>20:1)	70

[a]The reaction was carried out on 0.01 mmol scale for 4 h. [b]4.0 ml solvent was used (0.025 M). [c]ee% values were determined by HPLC. [d]dr values were determined by ^{19}F NMR. [e]Yields refer to isolated yieds. [f]0.1 equiv of PhCOOH was added. [g]2.0 equiv of Na_3PO_4 was added. [h]4Å molecular sieve was added. [i]The reaction was carried out on 0.01 mmol scale for 48 h.

From the results above we got the optimal reaction conditions for this catalytic, nucleophilic asymmetric aminofluorination: the reaction was carried out at −25°C in DCE (0.025 M) for 48 h with 15 mol% of **CIC1** as the catalysts, 1.2 equiv of *m*-CPBA as oxidant, 10.0 equiv of $BF_3 \cdot Et_2O$ as the nucleophilic fluorine reagent. Additional additives (4 Å MS, base or acid) were disadvantageous for the reaction.

3. Synthesis and Characterization of Catalysts

3.1 Synthesis of CIC1 and CIC2

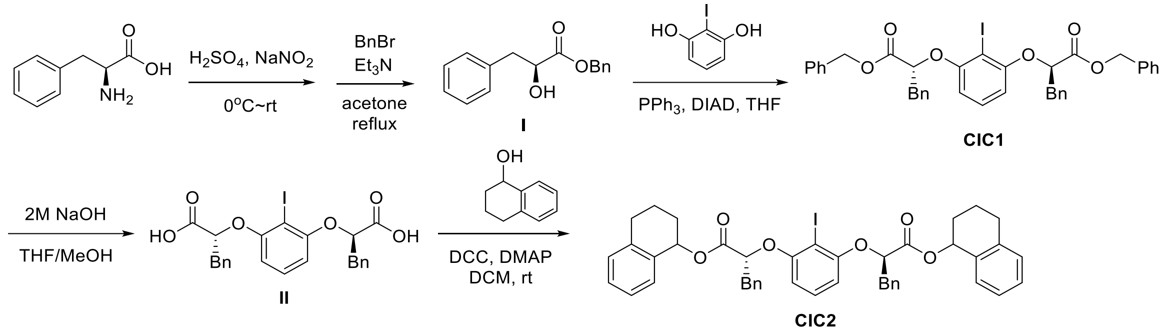

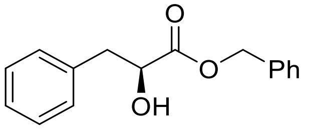

Benzyl (S)-2-hydroxy-3-phenylpropanoate: The α-hydroxy acid derivative **I** was synthesized following the reported procedure[1]. In brief, the L-phenylalanine (10 mmol) was dissolved in 1 M H_2SO_4 (10 mmol) and cooled to 0°C. Then 2 M $NaNO_2$ (20 mmol) was added dropwise and the reaction was allowed to slowly warm to room temperature and stirred overnight followed by addition of another aliquot of 2 M $NaNO_2$ (10 mmol) and the reaction was stirred for a further 24 h at rt. The reaction mixture was extracted with EtOAc (7 × 10 mL). The combined organic layers were washed with brine (15 mL), dried over Na_2SO_4, filtered and concentrated. The crude products (*S*)-2-hydroxy-3-phenylpropanoic acid was obtained as white solid and used without further purification in the next step. (*S*)-2-hydroxy-3-phenylpropanoic acid (10 mmol) was dissolved in 50 mL acetone, to this solution, benzyl bromide (10 mmol) and Et_3N (10 mmol) was added dropwise at rt. Then the mixture was refluxed for 16 h. The mixture was allowed to cool to room temperature and concentrated under reduced pressure. Then EtOAc (50 mL) was added to this residue and washed with water (50 mL) and brine (50 mL), the organic layer was dried over anhydrous Na_2SO_4. The solvents were evaporated under reduced pressure to obtain the crude products that was purified by silica gel flash column chromatography (hexanes/EtOAc (V/V) = 15:1~10:1) to give **I** as a colorless oil (72% over two steps).

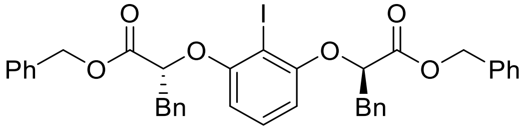

CIC1 was synthesized following the reported procedure[2]. Diisopropyl azodicarboxylate (DIAD, 4.65 g, 23.0 mmol, 2.30 equiv) was added dropwise via syringe over 30 minutes to a stirred suspension of resorcinol (2.36 g, 10.0 mmol, 1.0 equiv), triphenylphosphine (6.03 g, 23.0 mmol, 2.30 equiv), and **I** (3.66 g, 22.0 mmol, 2.2 equiv) in tetrahydrofuran (50 mL) at 0°C. The reaction mixture was warmed to room temperature. After 12 hours, the reaction mixture was concentrated under reduced pressure. The residue was purified by silica gel flash column chromatography (hexanes/EtOAc (V/V) = 15:1~10:1) to give (+)-**1a** (4.13 g, 58%) as a white solid. ^{1}H NMR (500 MHz, $CDCl_3$) δ 7.44-7.42 (d, *J* = 7.5 Hz, 5H), 7.33-7.27 (m, 11H), 7.19-7.18 (d, *J* = 7.5 Hz, 4H), 6.95-6.92 (t, *J* = 8.0 Hz, 1H), 6.22-6.20 (d, *J* = 8.0 Hz, 2H), 5.12 (s, 4H), 4.90-4.88 (q, *J* = 7.0 Hz, 2H), 3.43-3.39 (dd, *J* = 7.9, 14.0 Hz, 2H), 3.35-3.31 (dd, *J* = 7.9, 14.0 Hz, 2H); ^{13}C NMR (126 MHz, $CDCl_3$) δ 170.3, 158.0, 135.9, 135.1, 130.0, 129.4, 128.5, 128.4, 128.3 (2C), 127.1, 106.1, 79.6, 78.8, 67.0, 39.1.

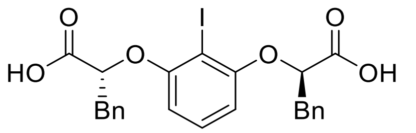

(2R,2'R)-2,2'-((2-iodo-1,3-phenylene)bis(oxy))bis(3-phenylpropanoic acid): According to the previous work[3], **CIC1** (3.56 g, 5.0 mmol, 1.0 equiv) was dissolved in 30 mL THF/MeOH (1:1). After addition of aqueous NaOH (2 M, 15.0 mL) the reaction mixture was stirred overnight at rt and then extracted with Et_2O (3 × 10 mL), the aqueous layers were acidified with aqueous HCl (3 M). After extraction with EtOAc (2 × 10 mL) the combined organic phases were washed with brine, dried over anhydrous Na_2SO_4 and concentrated under reduced pressure affording pure (2R,2'R)-2,2'-((2-iodo-1,3-phenylene)bis(oxy))bis- (3-phenylpropanoic acid) **II** (5.16 g, 4.85 mmol, 97 %). ^{1}H NMR (500 MHz, $CDCl_3$) δ 13.13 (b, 2H), 7.39-7.37 (d, *J* = 8.0 Hz, 4H), 7.23-7.21 (t, *J* = 7.5 Hz, 4H), 7.17-7.14 (t, *J* = 7.5 Hz, 2H), 7.09-7.05 (t, *J* = 8.5 Hz, 1H), 6.29-6.27 (d, *J* = 8.5 Hz, 2H), 4.88-4.86 (dd, *J* = 7.0 Hz, 2H), 3.19-3.16 (dd, *J* = 7.9, 14.0 Hz, 2H), 3.13-3.09 (dd, *J* = 7.9, 14.0 Hz, 2H); ^{13}C NMR (126 MHz, $CDCl_3$) δ 172.4, 158.2, 136.5, 129.7, 129.1, 127.8, 126.4, 105.5, 78.3, 38.6.

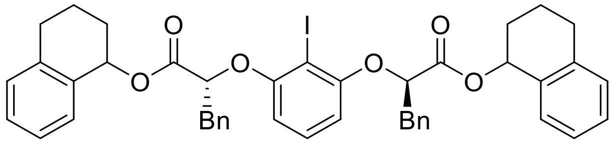

CIC2: Following the previous work[4], to a stirred solution of **II** (532 mg, 1.0 mmol, 1.0 equiv) in CH_2Cl_2 (5 mL) was added DMAP (61.1 mg, 0.5 mmol, 0.5 equiv) and DCC (619 mg, 3.0 mmol, 3.0 equiv) at 0°C. After 10 min, the 1,2,3,4-tetrahydronaphthalen-1-ol (445 mg, 3.0 mmol, 3.0 equiv) was added to the reaction mixture and stirred at rt for 12 h. The resulting suspension was filtered through Celite. The filtrate was then washed with 0.5 N HCl (2 × 5 mL) and sat. $NaHCO_3$ solution (2 × 5 mL). The organic layer was extracted with CH_2Cl_2 (3 × 10 mL), the combined organic layers were washed with brine (10 mL) and then dried over Na_2SO_4. The solvents were evaporated under reduced pressure to obtain the crude that was purified by silica gel column chromatography using hexanes/EtOAc (50:1~10:1) to yield the catalyst (+)-**1b** as a white solid (634 mg, 0.8 mmol, 80%). ^{1}H NMR (500 MHz, $CDCl_3$) δ 7.40-7.38 (d, *J* = 8.0 Hz, 4H), 7.29-7.20 (m, 7H), 7.11-7.06 (m, 4H), 6.99-6.89 (m, 3H), 6.73-6.70 (d, *J* = 7.5 Hz, 1H), 6.27-6.18 (m, 2H), 5.98-5.97 (d, *J* = 2.5 Hz, 1H), 5.86 (d, *J* = 2.5 Hz, 1H), 4.87-4.78 (m, 2H), 3.40-3.26 (m, 4H), 2.77-2.61 (m, 4H), 1.90-1.60 (m, 8H); ^{13}C NMR (126 MHz, $CDCl_3$) δ 170.2, 158.1, 137.8, 135.9, 133.7, 130.1, 130.0, 129.8, 129.4, 129.1, 128.9, 128.4, 128.1, 127.0, 125.9, 106.1, 79.8, 78.9, 71.0, 39.0, 28.8, 18.7, 18.4.

3.2 Synthesis of CIC3-CIC8

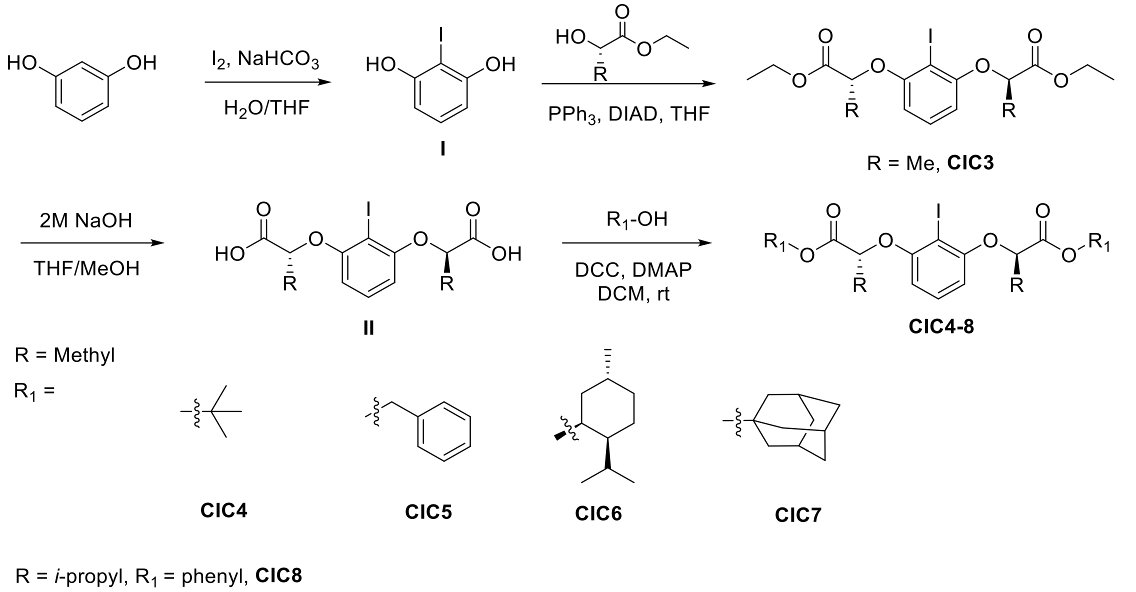

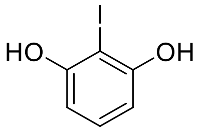

2-iodobenzene-1,3-diol: The chiral iodine catalysts were synthesis according to the previous work[5,6], which was described as follow: resorcinol (10.0 g, 90.818 mmol, 1 equiv) was dissolved in 70 mL H_2O. After cooling to 0°C, iodine (24.203 g, 95.359 mmol, 1.05 equiv) was added followed by slow addition of $NaHCO_3$ (8.393 g, 99.900 mmol, 1.1 equiv). The resulting reaction mixture was stirred for 10 min at 0°C and then allowed to warm to room temperature over a period of 20 min. The reaction was then quenched by addition of saturated aqueous $Na_2S_2O_3$ solution. After extraction with EtOAc, the combined organic phases were washed with brine, dried over anhydrous Na_2SO_4 and concentrated under reduced pressure. Purification by recrystallization from cold $CHCl_3$ afforded pure **I** (13.500 g, 57.201 mmol, 63 %).

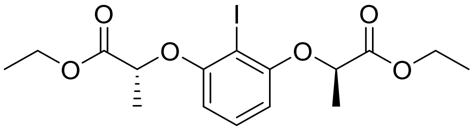

CIC3: Following a process by Fujita[7], 2-iodobenzene-1,3-diol **I** (5.5 g, 23.30 mmol, 1 equiv) was dissolved in 120 mL dry THF under an argon atmosphere. After addition of methyl (*S*)-lactate (5.34 g, 51.27 mmol, 2.2 equiv), triphenylphosphine (14.06 g, 55.60 mmol, 2.3 equiv) and diisopropyl azodicarboxylate (11.310 g, 55.930 mmol, 2.4 equiv) the reaction mixture was stirred for 16 h at room temperature and then concentrated under reduced pressure. Purification by flash column chromatography (hexanes/EtOAc (V/V) = 5:1) afforded pure **CIC3** (8.05 g, 19.73 mmol, 88 %) as colorless oil. ^{1}H NMR (500 MHz, $CDCl_3$) δ 7.07-7.04 (t, *J* = 8.5 Hz, 1H), 6.31-6.29 (d, *J* = 8.0 Hz, 2H), 4.70-4.66 (q, *J* = 7.0 Hz, 2H), 4.16-4.12 (d, *J* = 7.0 Hz, 4H), 1.63-1.62 (d, *J* = 7.0 Hz, 6H), 1.18-1.16 (d, *J* = 7.0 Hz, 6H); ^{13}C NMR (126 MHz, $CDCl_3$) δ 171.7, 158.3, 129.5, 107.0, 80.7, 74.3, 61.3, 18.6, 14.1.

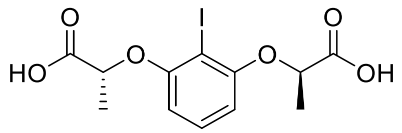

(2R,2'R)-2,2'-((2-iodo-1,3-phenylene)bis(oxy))dipropi-onic acid: According to the previous work[3], **CIC3** (2.2 g, 5.39 mmol, 1 equiv) was dissolved in 30 mL THF/MeOH (1:1). After addition of aqueous NaOH (2 M, 15.10 mL, 30.18 mmol, 5.6 equiv) the reaction mixture was stirred for 6 h at room temperature and then acidified with aqueous HCl (3 M). After extraction with EtOAc the combined organic phases were washed with brine, dried over anhydrous Na_2SO_4 and concentrated under reduced pressure affording pure **II** (1.87 g, 5.10 mmol, 95 %) as white solid.

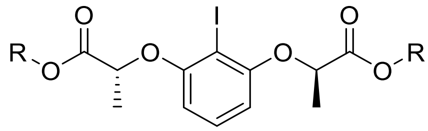

General procedure for synthesis of CIC4-7: Following the previous work[4], to a stirred solution of **II** (380 mg, 1.0 mmol, 1.0 equiv) in CH_2Cl_2 (5 mL) was added DMAP (61.1 mg, 0.5 mmol, 0.5 equiv), DCC (619 mg, 3.0 mmol, 3.0 equiv) at 0°C. After 10 min, the alcohol (3.0 mmol, 3.0 equiv) was added to the reaction mixture and stirred at rt for 12 h. The resulting suspension was filtered through Celite. The filtrate was then washed with 0.5 N HCl (2 × 5 mL) and sat. $NaHCO_3$ solution (2 × 5 mL). The organic layer was extracted with CH_2Cl_2 (3 × 10 mL), the combined organic layers were washed with brine (10 mL) and then dried over Na_2SO_4. The solvents were evaporated under reduced pressure to obtain the crude that was purified by silica gel column chromatography using hexanes/EtOAc (V/V) = (50:1~10:1) to yield the catalyst.

CIC4: colorless oil, 90%, ^{1}H NMR (400 MHz, $CDCl_3$) δ 7.24-7.19 (t, *J* = 8.4 Hz, 1H), 6.57-6.55 (d, *J* = 8.0 Hz, 2H), 4.20-4.18 (d, *J* = 8.0 Hz, 4H), 4.13-3.90 (m, 2H), 3.48-3.41 (m, 4H), 2.22-2.05 (m, 6H), 1.89-1.85 (m, 2H), 1.49 (s, 18H); ^{13}C NMR (101 MHz, $CDCl_3$) δ 158.8, 154.6, 129.8, 105.4, 100.0, 79.5, 69.5, 55.9, 47.0, 28.6.

CIC5: white solid, 92%, ^{1}H NMR (400 MHz, $CDCl_3$) δ 7.07-7.03 (t, *J* = 8.0 Hz, 1H), 6.29-6.27 (d, *J* = 8.0 Hz, 2H), 4.60-4.55 (q, *J* = 6.8 Hz, 2H), 1.59-1.58 (d, *J* = 6.8 Hz, 6H), 1.35 (s, 18H); ^{13}C NMR (101 MHz, $CDCl_3$) δ 170.8, 158.3, 129.2, 106.5, 81.9, 80.4, 74.5, 27.9, 18.5.

CIC6: colorless oil, 85%, ^{1}H NMR (400 MHz, $CDCl_3$) δ 7.06-7.02 (t, *J* = 8.0 Hz, 1H), 6.30-6.28 (d, *J* = 8.0 Hz, 2H), 4.71-4.66 (q, *J* = 6.8 Hz, 2H), 4.58-4.52 (dt, *J* = 4.4, 10.8 Hz, 2H), 1.94-1.91 (m, 2H), 1.63-1.61 (d, *J* = 6.8 Hz, 6H), 1.57-1.19 (m, 12H), 0.98-0.89 (m, 4H), 0.83-0.82 (d, *J* = 6.4 Hz, 6H), 0.66-0.64 (d, *J* = 6.8 Hz, 6H), 0.47-0.45 (d, *J* = 6.8 Hz, 6H); ^{13}C NMR (101 MHz, $CDCl_3$) δ 171.5, 158.3, 129.4, 106.3, 80.1, 75.4, 74.3, 46.7, 40.5, 34.1, 31.4, 25.5, 22.9, 22.0, 20.8, 18.6, 15.6.

CIC7: white solid, 88%, ^{1}H NMR (500 MHz, $CDCl_3$) δ 7.16-7.12 (t, *J* = 8.0 Hz, 1H), 6.39-6.37 (d, *J* = 8.0 Hz, 2H), 4.67-4.63 (q, *J* = 7.0 Hz, 2H), 3.43-3.39 (dd, *J* = 7.9, 14.0 Hz, 2H), 2.17-2.02 (m, 20H), 1.68-1.67 (d, *J* = 7.0 Hz, 6H), 1.66-1.64 (m, 10H); ^{13}C NMR (126 MHz, $CDCl_3$) δ 170.6, 158.3, 1292, 106.5, 82.0, 80.4, 74.4, 41.2, 36.1, 30.8, 18.6.

CIC8: white solid, 90%, ^{1}H NMR (500 MHz, $CDCl_3$) δ 7.35-7.34 (m, 5H), 7.29-7.27 (m, 4H), 7.09-7.06 (d, *J* = 8.2 Hz, 2H), 6.68-6.66 (d, *J* = 8.0 Hz, 2H), 5.20 (s, 4H), 4.53 (d, *J* = 5.0 Hz, 2H), 2.41-2.35 (m, 2H), 1.16-1.15 (d, *J* = 6.5 Hz, 6H), 1.14-1.13 (d, *J* = 6.5 Hz, 6H); ^{13}C NMR (126 MHz, $CDCl_3$) δ 170.4, 157.1, 156.4, 135.3, 130.0, 128.4, 108.3, 104.0, 82.1, 78.9, 66.8, 31.8, 19.0, 17.6.

3.3 Synthesis of CIC9 and CIC10

CIC9 and **CIC10** were synthesized following the previous work[8,9].

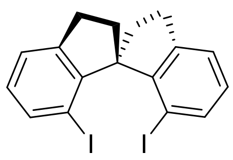

CIC9: ^{1}H NMR (500 MHz, $CDCl_3$) δ 7.66-7.65 (d, J = 7.5 Hz, 2H), 7.30-7.28 (d, J = 7.0 Hz, 2H), 6.95-6.92 (t, J = 7.5 Hz, 2H), 3.15-3.04 (m, 4H), 2.41-2.35 (m, 2H), 2.28-2.23 (m, 2H); ^{13}C NMR (126 MHz, $CDCl_3$) δ 148.3, 146.8, 138.0, 128.6, 124.7, 93.7, 66.3, 37.0, 30.7.

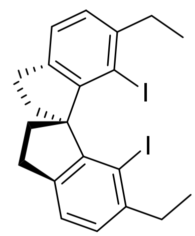

CIC10: ^{1}H NMR (400 MHz, $CDCl_3$) δ 7.17-7.15 (d, J = 8.0 Hz, 2H), 7.08-7.26 (d, J = 8.0 Hz, 2H), 3.06-3.02 (m, 4H), 2.77-2.70 (m, 4H), 2.49-2.41 (m, 2H), 2.24-2.18 (m, 2H), 1.19-1.15 (t, J = 7.5 Hz, 6H); ^{13}C NMR (101 MHz, $CDCl_3$) δ 149.5, 145.2, 143.8, 126.8, 124.5, 100.4, 68.3, 37.0, 34.4, 30.1, 15.1.

4. Synthesis and Characterization of Substrates

4.1 General Procedure for Synthesis of *N*-cinnamylbenzamides (1a-28a, 35a-38a)

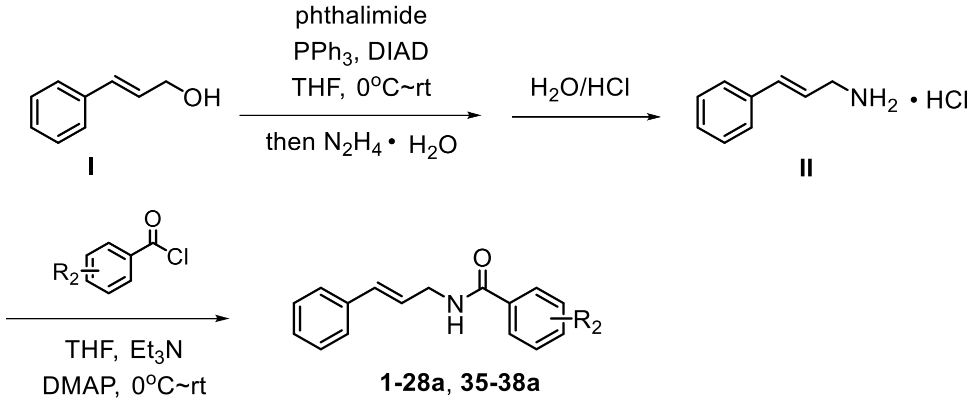

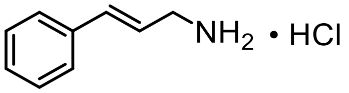

(*E*)-3-phenylprop-2-en-1-amine hydrochloride (II): Cinnamyl alcohol **I** (5 mmol, 1.0 equiv), phthalimide (1.1 equiv) and PPh_3 (1.1 equiv) was added to the reaction flask and dissolved in THF (5 mL/mmol). Then diisopropyl azodicarboxylate (DIAD) (1.1 equiv) was added drop wise at 0°C to the mixture. After TLC analysis revealed the complete consumption of starting material (30 min~60 min), 3 equiv of hydrazine hydrate was added to the reaction vessel and the resulting suspension was stirred overnight at room temperature. The reaction was diluted with water, concentrated HCl (3 mL) was added, and the resulting suspension was stirred for further 30 min at ambient temperature. The precipitated solids were filtered and the filter cake was washed with 10% aq. HCl (2 × 2 mL). The combined filtrates were washed with ether (3 × 5 mL) and the aqueous phase was concentrated under reduced pressure giving the amine salts **II**, which were used in the next reaction without any purification[10].

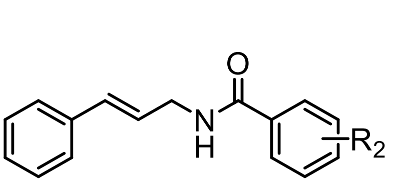

1a-28a, **35a-38a**

A solution of crude ammonium chloride salt **II** (5 mmol, 1 equiv) from the previous step, triethyl amine (5 equiv) and catalytic amount of DMAP in THF (20 mL) were cooled to 0°C. To this suspension was added benzoyl chloride (1.5 equiv). After the addition was completed, the reaction was

warmed to room temperature. After 3 h, the reaction was quenched with methanol (1.0 mL) and then diluted with an equal amount of water, concentrated under reduced pressure, and extracted with DCM (3 × 25 mL). The combined organic layer was washed with brine (1 × 20 mL), dried over anhydrous Na_2SO_4 and concentrated under reduced pressure in the presence of silica gel. Column chromatography (EtOAc-Hexanes gradient elution: hexanes/EtOAc (V/V) = 5:1~2:1) gave the desired products **1a-28a**, **35a-38a**[10].

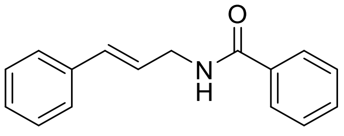

1a: R_f: 0.16 (PE/EtOAc (V/V) = 5:1); white solid, 85% yield. ^{1}H NMR (500 MHz, $CDCl_3$) δ 7.84-7.83 (d, *J* = 7.0 Hz, 2H), 7.54-7.51 (t, *J* = 7 Hz, 1H), 7.46-7.43 (t, *J* = 7.0 Hz, 3H), 7.39-7.37 (d, *J* = 7.0 Hz, 2H), 7.35-7.32 (t, *J* = 7.0 Hz, 2H), 7.28-7.25 (t, *J* = 7.0, 1H), 6.61-6.58 (d, *J* = 16 Hz, 1H), 6.49 (b, 1H), 6.33-6.29 (m, 1H), 4.27-4.25 (dd, *J* = 6.5 Hz, 2H); ^{13}C NMR (126 MHz, $CDCl_3$) δ 167.4, 136.5, 134.4, 132.4, 131.5, 128.6, 128.6, 127.8, 127.0, 126.4, 125.5, 42.1. LC-MS(EI): $[M+H]^+$, 238.2.

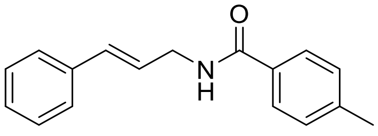

2a: R_f: 0.20 (PE/EtOAc (V/V) = 5:1); white solid, 86% yield. ^{1}H NMR (400 MHz, $CDCl_3$) δ 7.80-7.78 (d, *J* = 8.4 Hz, 2H), 7.33-7.24 (m, 5H), 7.20-7.18 (d, *J* = 8.4 Hz, 2H), 7.10 (b, 1H), 6.54-6.50 (d, *J* = 16 Hz, 1H), 6.29-6.22 (dt, *J* = 16.0, 6.0 Hz, 1H), 4.22-4.19 (dd, *J* = 6.4 Hz, 2H), 2.38 (s, 3H); ^{13}C NMR (101 MHz, $CDCl_3$) δ 167.5, 141.8, 136.7, 131.9, 129.2, 128.6, 127.6, 127.2, 126.4, 125.9, 42.1, 21.5. LC-MS(EI): $[M+H]^+$, 252.3.

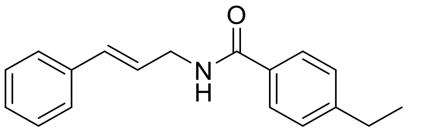

3a: R_f: 0.24 (PE/EtOAc (V/V) = 5:1); white solid, 85% yield. ^{1}H NMR (500 MHz, $CDCl_3$) δ 7.76-7.75 (d, *J* = 8.2 Hz, 2H), 7.29-7.24 (m, 4H), 7.20-7.16 (m, 3H), 6.94 (b, 1H), 6.50-6.47 (d, *J* = 16 Hz, 1H), 6.24-6.18 (dt, *J* = 16.0, 6.0 Hz, 1H), 4.18-4.15 (dd, *J* = 6.4 Hz, 2H), 2.66-2.61 (q, *J* = 7.5 Hz, 2H), 1.22-1.19 (t, *J* = 7.5 Hz, 3H); ^{13}C NMR (126 MHz, $CDCl_3$) δ 167.6, 148.1, 136.7, 132.0, 131.8, 128.6, 128.0, 127.7, 127.3, 126.4, 125.8, 42.1, 28.8, 15.4. LC-MS(EI): $[M+H]^+$, 266.2.

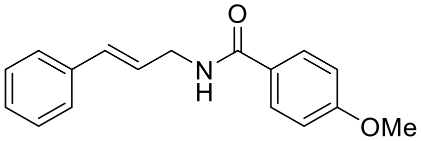

4a: R_f: 0.08 (PE/EtOAc (V/V) = 5:1); White solid, 90%. ^{1}H NMR (400 MHz, $CDCl_3$) δ 7.71-7.69 (d, *J* = 8.8 Hz, 2H), 7.24-7.18 (m, 4H), 7.15-7.13 (d, *J* = 8.0 Hz, 1H), 6.80-6.78 (d, *J* = 8.8Hz, 2H), 6.54 (b, 1H), 6.46-6.42 (d, *J* = 15.6 Hz, 1H), 6.19-6.12 (dt, *J* = 16.0, 6.4 Hz, 1H), 4.11-4.09 (dd, *J* = 5.6 Hz, 2H), 3.71 (s, 3H); ^{13}C NMR (101 MHz, $CDCl_3$) δ 167.0, 162.2, 136.6, 132.1, 128.9, 127.7, 126.7, 126.4, 125.8, 113.7, 55.4, 42.1. LC-MS(EI): $[M+H]^+$, 268.2.

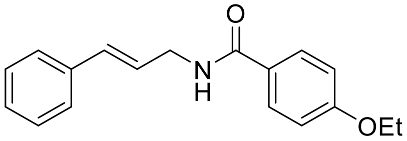

5a: R_f: 0.14 (PE/EtOAc (V/V) = 5:1); White solid, 90% yield. ^{1}H NMR (500 MHz, $CDCl_3$) δ 7.80-7.78 (d, *J* = 9 Hz, 2H), 7.38-7.37 (d, *J* = 7.5 Hz, 2H), 7.34-7.31 (t, *J* = 7.5 Hz, 2H), 7.28-7.23 (m, 1H), 6.93-6.91 (d, *J* = 8.5 Hz, 2H), 6.60-6.57 (d, *J* = 16.0 Hz, 1H), 6.38 (b, 1H), 6.32-6.27 (dt, *J* = 16.0, *J* = 6.0 Hz, 1H), 4.25-4.22 (dd, *J* = 5.5 Hz, 2H), 4.10-4.06 (q, *J* = 7.0 Hz, 2H), 1.46-1.43 (t, *J* = 7.0 Hz, 3H); ^{13}C NMR (126 MHz, $CDCl_3$) δ 166.9, 161.6, 136.6, 132.3, 128.8, 128.6, 128.4, 127.7, 126.4, 125.7, 114.2, 63.7, 42.1, 14.7. LC-MS(EI): $[M+H]^+$, 282.3.

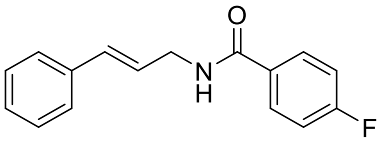

6a: R_f: 0.22 (PE/EtOAc (V/V) = 5:1); White solid, 92% yield. ^{1}H NMR (400 MHz, $CDCl_3$) δ 7.76-7.72 (m, 2H), 7.30-7.29 (d, *J* = 8.8Hz, 2H), 7.26-7.22 (t, *J* = 8.0 Hz, 2H), 7.19-7.17 (d, *J* = 8.0 Hz, 1H), 7.06-7.02 (t, *J* = 8.4 Hz, 2H), 6.54-6.50 (d, *J* = 16 Hz, 1H), 6.24-6.17 (m, 2H), 4.18-4.15 (dd, *J* = 6.4 Hz, 2H); ^{13}C NMR (101 MHz, $CDCl_3$) δ 166.3, 166.0, 136.4, 132.7, 129.3, 129.2, 128.6, 127.9, 126.4, 125.2, 115.8, 115.6, 42.2. LC-MS(EI): $[M+H]^+$, 256.2.

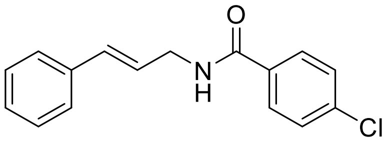

7a: R_f: 0.22 (PE/EtOAc (V/V) = 5:1); White solid, 93% yield. ^{1}H NMR (400 MHz, $CDCl_3$) δ 7.67-7.65 (d, *J* = 8.4 Hz, 2H), 7.30-7.16 (m, 7H), 6.49-6.45 (*J* = 16.0 Hz, d+b, 2H), 6.20-6.13 (dt, *J* = 16.0, 6.0 Hz, 1H), 4.14-4.11 (dd, *J* = 6.4 Hz, 2H); ^{13}C NMR (101 MHz, $CDCl_3$) δ 166.4, 137.8, 136.4, 132.8, 128.8, 128.6, 128.5, 127.9, 126.4, 125.1, 42.2. LC-MS(EI): $[M+H]^+$, 272.2.

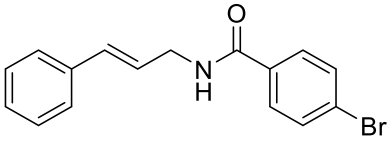

8a: R_f: 0.24 (PE/EtOAc (V/V) = 5:1); White solid, 89% yield. ^{1}H NMR (500 MHz, $CDCl_3$) δ 7.59-7.58 (d, *J* = 8.5 Hz, 2H), 7.46-7.45 (d, *J* = 9.0 Hz, 2H), 7.26-7.21 (m, 4H), 7.18-7.14 (m, 1H), 6.49-6.45 (d, *J* = 16.0 Hz, 1H), 6.47 (b, 1H), 6.19-6.13 (dt, *J* = 16.0, 6.5 Hz, 1H), 4.13-4.11 (dd, *J* = 5.5 Hz, 2H); ^{13}C NMR (126 MHz, $CDCl_3$) δ 166.5, 136.4, 133.2, 132.7, 131.8, 128.7, 127.9, 126.4, 126.2, 125.1, 42.3. LC-MS(EI): $[M+H]^+$, 316.2, 318.1.

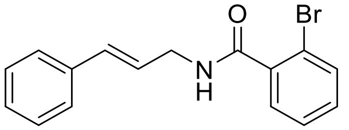

9a: R_f: 0.13 (PE/EtOAc (V/V) = 5:1); White solid, 82% yield. ^{1}H NMR (500 MHz, $CDCl_3$) δ 7.60-7.59 (d, *J* = 8.0 Hz, 1H), 7.55-7.54 (d, *J* = 8.0 Hz, 1H), 7.39-7.37 (t, *J* = 7.5 Hz, 2H), 7.35-7.32 (m, 3H), 7.29-7.26 (m, 2H), 6.66-6.62 (d, *J* = 16.0, 6.0 Hz, 1H), 6.32-6.27 (m, 2H), 4.25-4.23 (dd, *J* = 6.0 Hz, 2H); ^{13}C NMR (126 MHz, $CDCl_3$) δ 167.5, 137.7, 136.5, 133.4, 132.6, 131.3, 129.6, 128.6, 127.8, 127.6, 126.5, 124.9, 119.3, 42.1. LC-MS(EI): $[M+H]^+$, 316.1, 318.1.

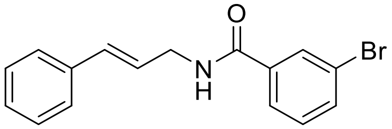

10a: R_f: 0.26 (PE/EtOAc (V/V) = 5:1); White solid, 85% yield. ^{1}H NMR (400 MHz, $CDCl_3$) δ 7.86 (s, 1H), 7.64-7.62 (d, *J* = 8.0 Hz, 1H), 7.50-7.47 (d, *J* = 8.0 Hz, 1H), 7.22-7.12 (m, 6H), 6.80 (b, 1H), 6.44-6.41 (d, *J* = 16.0 Hz, 1H), 6.15-6.08 (dt, *J* = 16.0, 6.0 Hz, 1H), 4.10-4.07 (dd, *J* = 6.4 Hz, 2H); ^{13}C NMR (101 MHz, $CDCl_3$) δ 166.2, 136.4, 136.3, 134.5, 132.6, 130.3, 130.1, 128.6, 127.8, 126.4, 125.8, 125.0, 122.7, 42.33. LC-MS(EI): $[M+H]^+$, 316.2, 318.1.

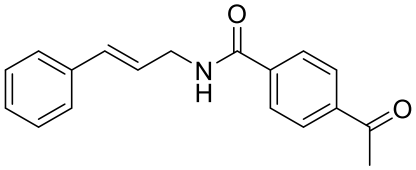

11a: R_f: 0.14 (PE/EtOAc (V/V) = 3:1); White solid, 75% yield. ^{1}H NMR (500 MHz, $CDCl_3$) δ 7.98-7.96 (d, *J* = 8.5 Hz, 2H), 7.89-7.88 (d, *J* = 8.5 Hz, 2H), 7.35-7.34 (d, *J* = 7.5 Hz, 2H), 7.32-7.29 (t, *J* = 7.5 Hz, 2H), 7.25-7.22 (t, *J* = 7.2 Hz, 1H), 6.70 (b, 1H), 6.59-6.56 (d, *J* = 16.0 Hz, 1H), 6.30-6.24 (dt, *J* = 16.0, 6.5 Hz, 1H,), 4.25-4.23 (dd, *J* = 5.5 Hz, 2H), 2.60 (s, 3H); ^{13}C NMR (126 MHz, $CDCl_3$) δ 197.6,

166.4, 139.2, 138.3, 136.4, 132.8, 128.6, 128.5, 127.9, 127.4, 126.4, 125.0, 42.3, 26.8. LC-MS(EI): $[M+H]^+$, 280.2.

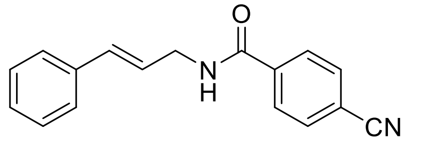

12a: R_f: 0.11 (PE/EtOAc (V/V) = 5:1); White solid, 89% yield. ^{1}H NMR (400 MHz, $CDCl_3$) δ 7.91-7.89 (d, *J* = 8.4 Hz, 2H), 7.68-7.66 (d, *J* = 8.4 Hz, 2H), 7.34-7.23 (m, 5H), 6.84 (b, 1H), 6.58-6.54 (d, *J* = 16.0 Hz, 1H), 6.27-6.20 (dt, *J* = 16.0, 6.4 Hz, 1H), 4.23-4.20 (dd, *J* = 6.0 Hz, 2H); ^{13}C NMR (101 MHz, $CDCl_3$) δ 165.7, 138.3, 136.2, 132.9, 132.4, 128.7, 128.0, 127.8, 126.4, 124.6, 118.1, 115.0, 42.4. LC-MS(EI): $[M+H]^+$, 263.1.

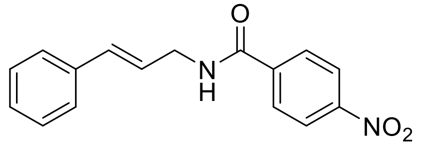

13a: R_f: 0.11 (PE/EtOAc (V/V) = 5:1); White solid, 80% yield. ^{1}H NMR (500 MHz, $CDCl_3$) δ 8.30-8.29 (d, *J* = 8.5 Hz, 2H), 7.99-7.97 (d, *J* = 8.5 Hz, 2H), 7.39-7.38 (d, *J* = 8.5 Hz, 2H), 7.36-7.33 (t, *J* = 8.5 Hz, 2H), 7.29-7.27 (t, *J* = 8.5 Hz, 1H), 6.65-6.62 (d, *J* = 16.0 Hz, 1H), 6.49 (b, 1H), 6.33-6.27 (dt, *J* = 16.0, 6.5 Hz, 1H), 4.30-4.27 (dd, *J* = 6.0 Hz, 2H); ^{13}C NMR (126 MHz, $CDCl_3$) δ 165.3, 149.6, 140.0, 136.2, 133.3, 128.7, 128.2, 128.1, 126.4, 124.5, 123.9, 42.5. LC-MS(EI): $[M+H]^+$, 283.2.

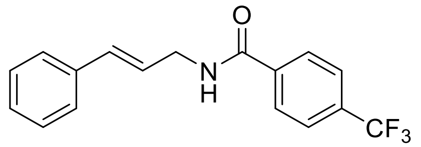

14a: R_f: 0.35 (PE/EtOAc (V/V) = 5:1); White solid, 92% yield. ^{1}H NMR (400 MHz, $CDCl_3$) δ 7.80-7.78 (d, *J* = 8.4 Hz, 2H), 7.51-7.49 (d, *J* = 8.4 Hz, 2H), 7.18-7.13 (m, 5H), 6.98 (b, 1H), 6.44-6.40 (d, *J* = 16.0 Hz, 1H), 6.15-6.08 (dt, *J* = 16.0, 6.0 Hz, 1H), 4.10-4.07 (dd, *J* = 6.4 Hz, 2H); ^{13}C NMR (101 MHz, $CDCl_3$) δ 166.4, 137.6, 136.3, 133.3, 133.0, 128.6, 127.9, 127.6, 126.4, 125.59-125.48 (q, *J* = 3.6 Hz), 124.9, 42.3. LC-MS(EI): $[M+H]^+$, 306.2.

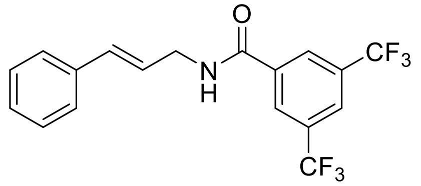

15a: R_f: 0.60 (PE/EtOAc (V/V) = 5:1); White solid, 90% yield. ^{1}H NMR (500 MHz, $CDCl_3$) δ 8.34 (s, 2H), 7.97 (s, 1H), 7.87 (b, 1H), 7.30-7.24 (m, 5H), 6.54-6.51 (d, *J* = 16.0 Hz, 1H), 6.15-6.08 (dt, *J* = 16.0, 6.0 Hz, 1H), 4.25-4.23 (dd, *J* = 5.5 Hz, 2H); ^{13}C NMR (101 MHz, $CDCl_3$) δ 165.0, 136.3, 136.1, 132.9, 132.4-131.6 (q, *J* = 33.9 Hz), 128.6, 127.9, 127.6, 126.3, 124.2, 124.0, 121.8, 42.6. ^{19}F (471 Hz, $CDCl_3$) 63.04. LC-MS(EI): $[M+H]^+$, 374.2.

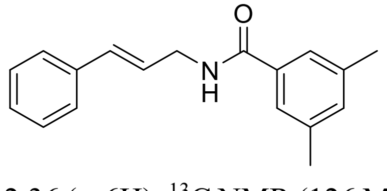

16a: R_f: 0.29 (PE/EtOAc (V/V) = 5:1); White solid, 89% yield. ^{1}H NMR (500 MHz, $CDCl_3$) δ 7.45 (s, 2H), 7.37-7.36 (d, *J* = 7.0 Hz, 2H), 7.34-7.31 (t, *J* = 7.0 Hz, 2H), 7.27-7.24 (t, *J* = 7.0 Hz, 1H), 7.14 (s, 1H), 6.59-6.56 (d, *J* =15.5 Hz, 1H), 6.54 (b, 1H), 6.31-6.26 (dt, *J* = 15.5, 6.5 Hz, 1H), 4.25-4.22 (dd, *J* = 6.0 Hz, 2H), 2.36 (s, 6H); ^{13}C NMR (126 MHz, $CDCl_3$) δ 167.8, 138.2, 136.6, 134.5, 133.1, 132.2, 128.6, 127.7, 126.4, 125.6, 124.8, 42.1, 21.3. LC-MS(EI): $[M+H]^+$, 266.2.

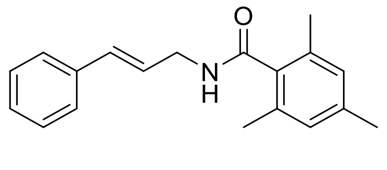

17a: R_f: 0.21 (PE/EtOAc (V/V) = 5:1); White solid, 88% yield. ^{1}H NMR (500 MHz, $CDCl_3$) δ 7.39-7.38 (d, *J* = 7.0 Hz, 2H), 7.36-7.33 (t, *J* = 8.0 Hz, 2H), 7.29-7.26 (t, *J* = 7.0 Hz, 2H), 6.86 (s, 2H), 6.63-6.60 (d, *J* =15.5 Hz, 1H), 6.32-6.26 (dt, *J* = 15.5, 6.5 Hz 1H),

5.87 (b, 1H), 4.26-4.23 (dd, J = 6.0 Hz, 2H), 2.33 (s, 6H), 2.29 (s, 1H); ^{13}C NMR (126 MHz, $CDCl_3$) δ 170.4, 138.5, 136.5, 134.8, 134.1, 132.5, 128.6, 128.2, 127.8, 126.4, 125.3, 41.7, 21.1, 19.2. LC-MS(EI): $[M+H]^+$, 280.3.

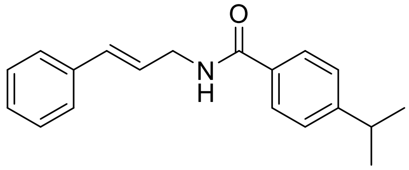

18a: R_f: 0.29 (PE/EtOAc (V/V) = 5:1); White solid, 92% yield. ^{1}H NMR (500 MHz, $CDCl_3$) δ 7.79-7.77 (d, J = 8.0 Hz, 2H), 7.38-7.37 (d, J = 8.0 Hz, 2H), 7.34-7.25 (m, 5H), 7.27-7.24 (t, J = 7.0 Hz, 2H), 6.61-6.58 (d, J =15.5 Hz, 1H), 6.49 (b, 1H), 6.33-6.27 (dt, J = 15.5, 6.5 Hz, 1H), 4.27-4.24 (dd, J = 6.0 Hz, 2H), 3.01-2.93 (m, 1H), 1.29-1.28 (d, J = 7.0 Hz, 6H); ^{13}C NMR (126 MHz, $CDCl_3$) δ 167.3, 152.8, 136.6, 132.2, 132.0, 128.6, 127.7, 127.1, 126.7, 126.4, 125.7, 42.1, 34.1, 23.8. LC-MS(EI): $[M+H]^+$, 280.2.

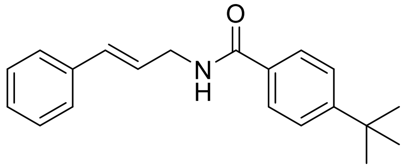

19a: R_f: 0.32 (PE/EtOAc (V/V) = 5:1); White solid, 93% yield. ^{1}H NMR (500 MHz, $CDCl_3$) δ 7.78-7.76 (d, J = 8.0 Hz, 2H), 7.48-7.46 (d, J = 8.0 Hz, 2H), 7.40-7.38 (d, J = 8.0 Hz, 2H), 7.35-7.32 (t, J = 7.0 Hz, 2H), 7.28-7.25 (t, J = 7.0 Hz, 2H), 6.63-6.59 (d, J =16.0 Hz, 1H), 6.34-6.28 (m, 2H), 4.28-4.26 (dd, J = 6.0 Hz, 2H), 1.36 (s, 9H); ^{13}C NMR (126 MHz, $CDCl_3$) δ 167.3, 155.0, 136.6, 132.3, 131.6, 128.6, 127.7, 126.8, 126.4, 125.6, 125.5, 42.0, 34.9, 31.2. LC-MS(EI): $[M+H]^+$, 294.3.

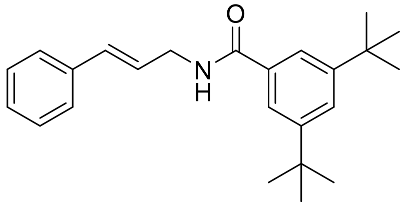

20a: R_f: 0.41 (PE/EtOAc (V/V) = 5:1); White solid, 90% yield. ^{1}H NMR (500 MHz, $CDCl_3$) δ 7.54 (s, 2H), 7.50 (s, 1H), 7.32-7.30 (d, J = 8.5 Hz, 2H), 7.26-7.23 (t, J = 7.0 Hz, 2H), 7.19-7.17 (d, J = 8.5 Hz, 1H), 6.56-6.52 (d, J =16.0 Hz, 1H), 6.27-6.22 (dt, J = 15.5, 6.5 Hz, 1H), 6.18 (b, 1H), 4.20-4.18 (dd, J = 6.0 Hz, 2H), 1.28 (s, 18H); ^{13}C NMR (126 MHz, $CDCl_3$) δ 168.4, 151.3, 136.6, 134.1, 132.4, 128.6, 127.8, 126.4, 125.7, 121.1, 42.2, 35.0, 31.4. LC-MS(EI): $[M+H]^+$, 350.4.

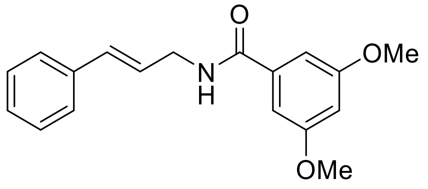

21a: R_f: 0.28 (PE/EtOAc (V/V) = 3:1); White solid, 80% yield. ^{1}H NMR (400 MHz, $CDCl_3$) δ 7.18-7.08 (m, 5H), 6.94 (b, 1H), 6.87 (s, 2H), 6.42 (s, 1H), 6.38-6.34 (d, J = 16.0 Hz, 1H), 6.12-6.05 (dt, J = 16.0, 6.0 Hz, 1H), 4.04-4.01 (dd, J = 6.4 Hz, 2H), 3.63 (s, 6H); ^{13}C NMR (101 MHz, $CDCl_3$) δ 167.4, 160.8, 136.6, 136.5, 132.1, 128.6, 127.7, 126.4, 125.5, 105.0, 103.7, 55.5, 42.2. LC-MS(EI): $[M+H]^+$, 298.2.

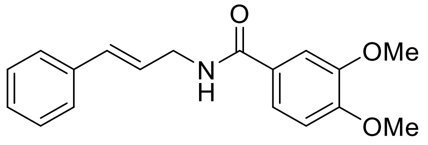

22a: R_f: 0.09 (PE/EtOAc (V/V) = 3:1); White solid, 75% yield. ^{1}H NMR (500 MHz, $CDCl_3$) δ 7.48 (s, 1H), 7.38-7.33 (m, 3H), 7.32-7.28 (t, J = 7.5 Hz, 2H), 7.25-7.22 (t, J = 7.0 Hz, 1H), 6.85-6.83 (d, J = 8.5 Hz, 1H), 6.62 (b, 1H), 6.57-6.53 (d, J =16.0 Hz, 1H), 6.30-6.24 (dt, J = 15.5 Hz, J = 6.5 Hz, 1H), 4.22-4.20 (dd, J = 6.0 Hz, 2H), 3.90 (s, 6H); ^{13}C NMR (126 MHz, $CDCl_3$) δ 167.0, 151.7, 148.9, 136.5, 132.2, 128.6, 127.7, 127.0, 126.4, 125.7, 119.6, 110.6, 110.3, 56.0, 42.2. LC-MS(EI): $[M+H]^+$, 298.2.

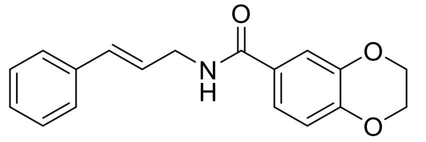

23a: R_f: 0.2 (PE/EtOAc (V/V) = 3:1); White solid, 85% yield. ^{1}H NMR (500 MHz, $CDCl_3$) δ 7.39 (s, 1H), 7.32-7.30 (m, 3H), 7.29-7.26 (t, *J* = 8.0 Hz, 2H), 7.22-7.19 (t, *J* = 8.0 Hz, 1H), 6.86-6.84 (d, *J* = 8.0 Hz, 1H), 6.57 (b, 1H), 6.53-6.50 (d, *J* =16.0 Hz, 1H), 6.26-6.20 (dt, *J* = 15.5, 6.5 Hz, 1H), 4.24-4.21 (m, 4H), 4.18-4.15 (dd, *J* = 6.0 Hz, 2H); ^{13}C NMR (126 MHz, $CDCl_3$) δ 166.7, 146.5, 143.4, 136.6, 132.1, 128.6, 127.7, 127.6, 126.4, 125.7, 120.5, 117.2, 116.7, 64.5, 64.2, 42.1. LC-MS(EI): $[M+H]^+$, 296.2.

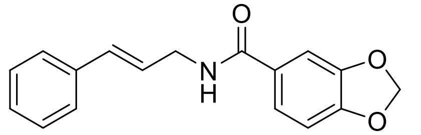

24a: R_f: 0.12 (PE/EtOAc (V/V) = 5:1); White solid, 85% yield. ^{1}H NMR (500 MHz, $CDCl_3$) δ 7.35-7.33 (m, 3H), 7.31-7.28 (t, *J* = 8.0 Hz, 2H), 7.24-7.21 (t, *J* = 7.2 Hz, 1H), 6.81-6.79 (d, *J* = 8.0 Hz, 1H), 6.56-6.53 (d, *J* =16.0 Hz, 1H), 6.40 (b, 1H), 6.28-6.22 (dt, *J* = 15.5, 6.5 Hz, 1H), 5.99 (s, 2H), 4.20-4.18 (dd, *J* = 6.0 Hz, 2H); ^{13}C NMR (126 MHz, $CDCl_3$) δ 166.7, 150.4, 148.0, 136.5, 132.4, 128.6, 127.8, 126.4, 125.5, 121.6, 108.0, 107.7, 101.7, 42.2. LC-MS(EI): $[M+H]^+$, 282.1.

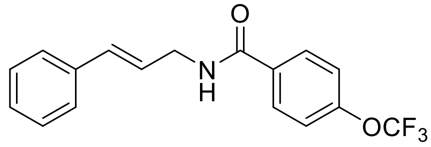

25a: R_f: 0.28 (PE/EtOAc (V/V) = 5:1); white solid, 88% yield. ^{1}H NMR (500 MHz, $CDCl_3$) δ 7.88-7.87 (d, *J* = 8.5 Hz, 2H), 7.36-7.31 (m, 4H), 7.28-7.23 (m, 3H), 6.76 (b, 1H), 6.59-6.55 (d, *J* = 16.0 Hz, 1H), 6.29-6.24 (dt, *J* = 16 Hz, *J* = 6.0 Hz, 1H), 4.24-4.22 (dd, *J* = 6.0 Hz, 2H); ^{13}C NMR (126 MHz, $CDCl_3$) δ 166.2, 151.5-151.4 (q, 1C, *J* = 1.6 Hz), 136.4, 132.8, 132.6, 129.0, 128.6, 127.9, 126.4, 125.1, 120.6, 42.6. LC-MS(EI): $[M+H]^+$, 322.2.

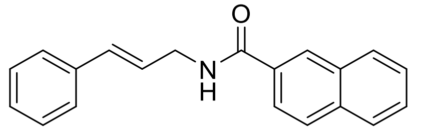

26a: R_f: 0.19 (PE/EtOAc (V/V) = 5:1); White solid, 90% yield. ^{1}H NMR (400 MHz, $CDCl_3$) δ 8.25 (s, 1H), 7.87-7.78 (m, 4H), 7.52-7.45 (m, 2H), 7.33-7.32 (d, *J* = 8.8 Hz, 2H), 7.27-7.23 (t, *J* = 8.0 Hz, 2H), 7.20-7.18 (m, 1H), 6.59-6.55 (d, *J* = 16.0 Hz, 1H), 6.35 (b, 1H), 6.31-6.24 (dt, *J* = 16.0, 6.0 Hz, 1H), 4.27-4.24 (dd, *J* = 5.6 Hz, 2H); ^{13}CNMR (101 MHz, $CDCl_3$) δ 167.4, 136.5, 134.8, 132.7, 132.6, 131.7, 128.9, 128.6, 128.5, 127.8 (2C), 127.7, 127.5, 126.8, 126.4, 125.5, 123.6. LC-MS(EI): $[M+H]^+$, 288.3.

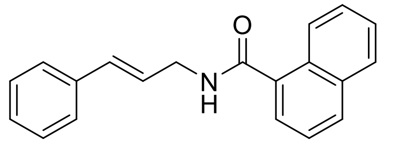

27a: R_f: 0.19 (PE/EtOAc (V/V) = 5:1); white solid, 88% yield. ^{1}H NMR (500 MHz, $CDCl_3$) δ 8.29-8.27 (d, *J* = 8.5 Hz, 1H), 7.86-7.84 (d, *J* = 8.0 Hz, 1H), 7.81-7.79 (d, *J* = 8.0 Hz, 1H), 7.58-7.56 (d, *J* = 7.5 Hz, 1H), 7.49-7.46 (t, *J* = 8.5 Hz, 2H), 740-7.37 (t, *J* = 7.5 Hz, 1H), 7.32-7.31 (d, *J* = 6.5 Hz, 2H), 7.27-7.24 (t, *J* = 8.5 Hz, 2H), 7.20-7.16 (t, *J* = 8.5 Hz, 1H), 6.58-6.55 (d, *J* =15.5 Hz, 1H), 6.30-6.25 (dt, *J* = 15.5, 6.5 Hz, 1H), 6.08 (b, 1H), 4.28-4.25 (dd, *J* = 6.0 Hz, 2H); ^{13}C NMR (126 MHz, $CDCl_3$) δ 169.4, 136.5, 134.4, 133.7, 132.6, 130.7, 130.2, 128.6, 128.3, 127.8, 127.2, 126.5, 126.4, 125.4, 125.3, 124.9, 124.7, 42.1. LC-MS(EI): $[M+H]^+$, 288.2.

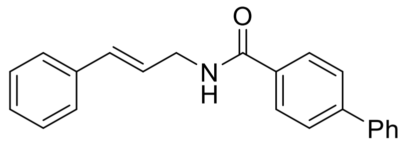

28a: R_f: 0.14 (PE/EtOAc (V/V) = 5:1); white solid, 85% yield. ^{1}H NMR (500 MHz, $CDCl_3$) δ 7.81-7.80 (d, *J* = 7.8 Hz, 2H), 7.58-7.56 (d, *J* = 7.5 Hz, 2H), 7.53-7.51 (d, *J* = 7.5 Hz, 2H), 7.39-7.36 (t, *J* = 7.5 Hz, 2H), 7.30-7.28 (d, *J* = 8.5 Hz, 2H), 7.24-7.22

(t, J = 7.5 Hz, 2H),7.18-7.15 (t, J = 7.5 Hz, 1H), 6.54-6.51 (d, J = 16.0 Hz, 1H), 6.38 (b, 1H), 6.25-6.20 (dt, J = 16.0, 6.0 Hz, 1H), 4.20-4.18 (dd, J = 6.0 Hz, 2H); ^{13}C NMR (126 MHz, $CDCl_3$) δ 167.1, 144.4, 140.0, 136.5, 133.0, 132.5, 128.9, 128.6, 128.0, 127.8, 127.5, 127.3, 127.2, 126.4, 125.4, 42.2. LC-MS(EI): $[M+H]^+$, 314.2.

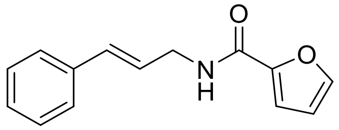

35a: R_f: 0.15 (PE/EtOAc (V/V) = 5:1); white solid, 90% yield. ^{1}H NMR (400 MHz, $CDCl_3$) δ 7.46 (s, 1H), 7.40-7.38 (d, J = 8.0 Hz, 2H), 7.35-7.31 (t, J = 8.0 Hz, 2H), 7.28-7.26 (d, J = 8.4 Hz, 2H), 7.17-7.16 (d, J = 3.6 Hz, 1H), 6.63-6.59 (d, J = 16.0 Hz, 1H), 6.55-6.52 (m, 2H), 6.32-6.25 (dt, J = 16.0, 6.0 Hz, 1H), 4.25-4.22 (dd, J = 6.0 Hz, 2H); ^{13}C NMR (101 MHz, $CDCl_3$) δ 158.22, 148.0, 143.9, 136.5, 132.5, 128.6, 127.8, 126.4, 125.2, 114.4, 112.2, 41.2. LC-MS(EI): $[M+H]^+$, 228.2.

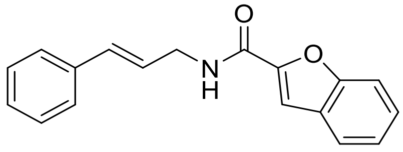

36a: R_f: 0.24 (PE/EtOAc (V/V) = 5:1); white solid, 80% yield. ^{1}H NMR (400 MHz, $CDCl_3$) δ 7.70-7.68 (d, J = 8.0 Hz, 1H), 7.53-7.51 (d, J = 8.0 Hz, 2H), 7.45-7.39 (m, 3H), 7.36-7.32 (t, J = 8.4 Hz, 3H), 7.29-7.27 (d, J = 8.0 Hz, 1H), 6.90 (b, 1H), 6.67-6.63 (d, J = 16.0 Hz, 1H), 6.35-6.28 (dt, J = 16.0, 6.0 Hz, 1H), 4.32-4.28 (dd, J = 6.0 Hz, 2H); ^{13}C NMR (101 MHz, $CDCl_3$) δ 158.7, 154.8, 148.7, 136.4, 132.8, 128.6, 127.9, 127.6, 126.9, 126.5, 125.0, 123.7, 122.8, 111.7, 110.6, 41.4. LC-MS(EI): $[M+H]^+$, 278.2

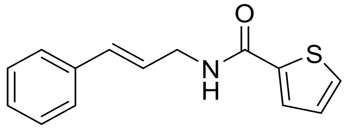

37a: R_f: 0.17 (PE/EtOAc (V/V) = 5:1); white solid, 90% yield. ^{1}H NMR (500 MHz, $CDCl_3$) δ 7.53-7.52 (dd, J = 3.7, 1.1 Hz, 1H), 7.33-7.32 (dd, J = 5.0, 1.1 Hz, 1H), 7.20-7.15 (m, 4H), 7.12-7.10 (m, 1H), 6.93-6.91 (dd, J = 5.0, 3.8 Hz, 1H,), 6.80 (b, 1H), 6.42-6.38 (d, J =16.0 Hz, 1H), 6.13-6.08 (dt, J = 16.0, 6.0 Hz, 1H), 4.22-4.20 (dd, J = 6.0 Hz, 2H); ^{13}C NMR (126 MHz, $CDCl_3$) δ 162.1, 139.1, 136.6, 132.3, 130.1, 128.6, 128.3, 127.7, 126.4, 125.4, 42.1. LC-MS(EI): $[M+H]^+$, 244.2

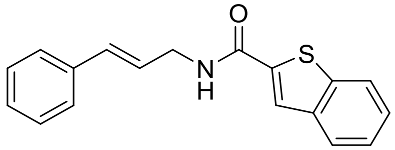

38a: R_f: 0.20 (PE/EtOAc (V/V) = 5:1); white solid, 82% yield. ^{1}H NMR (400 MHz, $CDCl_3$) δ 7.87-7.81 (m, 3H), 7.43-7.37 (m, 4H), 7.33-7.30 (t, J = 8.0 Hz, 2H), 7.26-7.21 (d, J = 8.0 Hz, 1H), 6.64-6.60 (d, J = 16.0 Hz, 1H), 6.31-6.26 (m, 2H), 4.28-4.25 (dd, J = 6.0 Hz, 2H).

^{13}C NMR (101 MHz, $CDCl_3$) δ 162.2, 140.8, 139.1, 138.2, 136.4, 132.8, 128.6, 127.9, 126.4, 125.5, 125.2, 125.1, 125.0, 124.9, 122.7, 42.3. LC-MS(EI): $[M+H]^+$, 294.2.

4.2 General Procedure for Synthesis of 29a-34a

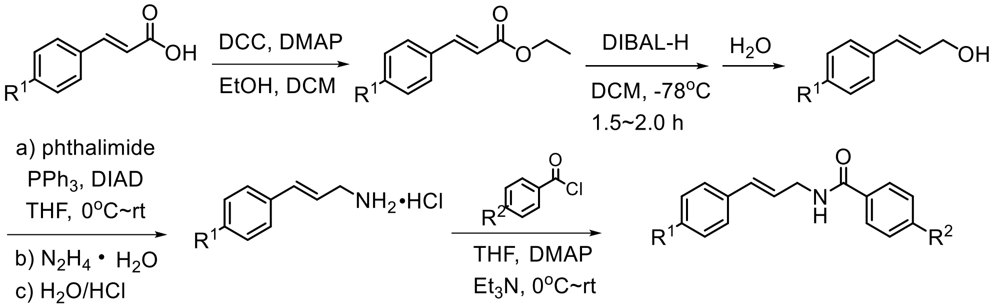

The cinnamic acid (10.0 mmol 1.0 equiv) was dissolved in DCM (0.2 M) at 0°C, then DCC (12 mmol, 1.2 equiv) and DMAP (1.0 mmol, 0.1 equiv) were added in one portion. Following by addition of EtOH (12 mmol, 1.2 equiv) dropwise. The reaction was allowed warm to rt and stirred for 12 h. The reaction mixture was filtrated and the filtrate was washed with water (30 ml × 3). The organic layer was dried over anhydrous Na_2SO_4 and concentrated under reduced pressure in the presence of silica gel. Column chromatography (EtOAc-Hexanes elution: hexanes/EtOAc (V/V) = 25:1) gave the desired ethyl cinnamate (90~95%)[4].

The ethyl cinnamate was dissolved in dry DCM (0.2 M) under N_2 atmosphere and the solution was cooled to -78°C. To this solution, DIBAL-H (2.2 equiv) was added dropwise during 30 min. the mixture was stirred at -78°C for 1.5 h, and the reaction was quenched with water (1.0 mL per 1.0 mL of DIBAL-H solution). The resulting mixture was allowed to warm to rt and stirred for additional 1 h. The aqueous layers were extracted with DCM (30 ml × 3), and the combined organic layers were washed with brine, dried over Na_2SO_4 concentrated under reduced pressure in the presence of silica gel, Column chromatography (EtOAc-Hexanes gradient elution: hexanes/EtOAc (V/V) = 5:1) gave the corresponding cinnamyl alcohol (90-92%)[11].

The cinnamon ammonium chloride salt was synthesized from the corresponding cinnamyl alcohol according the procedure described in previous work[10]. A solution of crude ammonium chloride salt from the previous step (1.0 equiv), triethyl amine (5.0 equiv) and catalytic amount of DMAP in THF (20 mL) were cooled to 0°C. To this suspension was added benzoyl chloride (1.5 equiv). After the addition was completed, the reaction was warmed to room temperature. After 3 h, the reaction was quenched with methanol (1.0 mL) and then diluted with an equal amount of water, concentrated under reduced pressure, and extracted with DCM (3 × 25 mL). The combined organic layer was washed with brine (1 × 20 mL), dried over anhydrous Na_2SO_4 and concentrated under reduced pressure in the presence of silica gel. Column chromatography (EtOAc-Hexanes gradient elution: hexanes/EtOAc (V/V) = 10:1 ~ 5:1) gave the desired products **29a-33a** (80-85%).

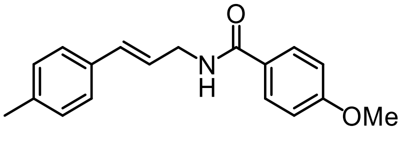

29a: R_f: 0.24 (PE/EtOAc (V/V) = 5:1); white solid, 82% yield. ^{1}H NMR (500 MHz, $CDCl_3$) δ 7.81-7.79 (d, J = 8.8 Hz, 2H), 7.27-7.26 (d, J = 8.0 Hz, 2H), 7.14-7.12 (d, J = 8.0 Hz, 2H), 6.93-6.92 (d, J = 8.0 Hz, 2H), 6.56-6.53 (d, J =15.5 Hz, 1H), 6.43 (b, 1H), 6.27-6.21 (dt, J = 16.5, 6.0 Hz, 1H), 4.23-4.21 (dd, J = 6.0 Hz, 2H), 3.85 (s, 3H), 2.35 (s, 3H); ^{13}C NMR (126 MHz, $CDCl_3$) δ 166.9, 162.2, 137.6, 133.8, 132.2, 129.3, 128.8, 126.7, 126.3, 124.6, 113.7, 55.4, 42.2, 21.2. LC-MS(EI): $[M+H]^+$, 282.2.

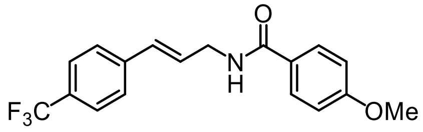

30a: R_f: 0.17 (PE/EtOAc (V/V) = 5:1); white solid, 80% yield. ^{1}H NMR (400 MHz, $CDCl_3$) δ 7.80-7.78 (d, *J* = 8.0 Hz, 2H), 7.57-7.55 (d, *J* = 8.0 Hz, 2H), 7.46-7.44 (d, *J* = 8.0 Hz, 2H), 6.95-6.92 (d, *J* = 8.0 Hz, 2H), 6.62-6.58 (d, *J* = 16.0 Hz, 1H), 6.43 -6.36 (m, 2H), 4.28-4.25 (dd, *J* = 6.0 Hz, 2H), 3.85 (s, 3H); ^{13}C NMR (126 MHz, $CDCl_3$) δ 166.9, 162.3, 132.1, 132.0, 130.6, 128.8, 128.7, 128.6, 128.5, 126.5, 125.5 (q, *J* = 3.8 Hz), 113.8, 55.4, 41.9; ^{19}F NMR (471 MHz, $CDCl_3$) δ -62.51. LC-MS(EI): $[M+H]^+$, 336.2.

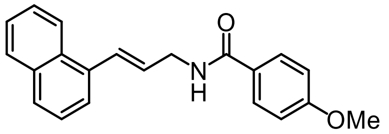

31a: R_f: 0.20 (PE/EtOAc (V/V) = 5:1); white solid, 80% yield. ^{1}H NMR (400 MHz, $CDCl_3$) δ 8.05-8.03 (d, *J* = 9.2 Hz, 1H), 7.82-7.79 (m, 3H), 7.75-7.73 (d, *J* = 8.4 Hz, 1H), 7.53-7.51 (d, *J* = 8.0 Hz, 1H), 7.47-7.45 (m, 2H), 7.41-7.37 (t, *J* = 8.0 Hz, 1H), 7.30-7.26 (d, *J* = 16.0 Hz, 1H), 6.87-6.85 (d, *J* = 8.0 Hz, 2H), 6.70 (b, 1H), 6.30-6.23 (dt, *J* = 16.0, 6.0 Hz, 1H), 4.31-4.28 (dd, *J* = 6.0 Hz, 2H), 3.77 (s, 3H); ^{13}C NMR (126 MHz, $CDCl_3$) δ 167.0, 162.2, 134.3, 133.6, 131.1, 129.4, 129.0, 128.9, 128.6, 128.1, 126.7, 126.1, 125.8, 125.6, 124.0, 123.7, 113.8, 55.4, 42.4. LC-MS(EI): $[M+H]^+$, 318.3.

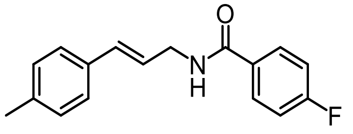

32a: R_f: 0.35 (PE/EtOAc (V/V) = 5:1); white solid, 81% yield. ^{1}H NMR (400 MHz, $CDCl_3$) δ 7.83-7.79 (dd, *J* = 8.7, 5.3 Hz, 2H), 7.26-7.24 (d, *J* = 8.0 Hz, 2H), 7.13-7.08 (m, 4H), 6.57-6.53 (d, *J* = 16.0 Hz, 1H), 6.32 (b, 1H), 6.25-6.18 (dt, *J* = 16.0, 6.0 Hz, 1H), 4.22-4.19 (dd, *J* = 6.0 Hz, 2H), 2.33 (s, 3H); ^{13}C NMR (101 MHz, $CDCl_3$) δ 166.3, 164.7 (d, *J* = 251.8 Hz), 137.7, 133.6, 132.6, 129.3, 129.2, 126.3, 124.1, 115.7, 115.7, 115.5, 42.3, 21.2. ^{19}F NMR (101 MHz, $CDCl_3$) δ -108.2. LC-MS(EI): $[M+H]^+$, 270.2.

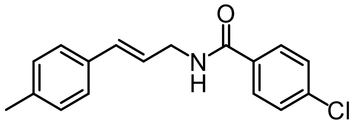

33a: R_f: 0.33 (PE/EtOAc (V/V) = 5:1); white solid, 85% yield. ^{1}H NMR (500 MHz, $CDCl_3$) δ 7.75-7.73 (d, *J* = 8.5 Hz, 2H), 7.41-7.39 (d, *J* = 8.5 Hz, 2H), 7.27-7.25 (d, *J* = 8.5 Hz, 2H), 7.13-7.11 (d, *J* = 8.5 Hz, 2H), 6.57-6.54 (d, *J* = 15.5 Hz, 1H), 6.29 (b, 1H), 6.29-6.19 (dt, *J* = 15.5, 6.0 Hz, 1H), 4.23-4.20 (dd, *J* = 6.0 Hz, 2H), 2.33 (s, 3H); ^{13}C NMR (101 MHz, $CDCl_3$) δ 166.2, 137.8, 133.6, 132.8, 129.3, 128.9, 128.7, 128.4, 128.2, 126.3, 124.0, 42.3, 21.0. LC-MS(EI): $[M+H]^+$, 286.3.

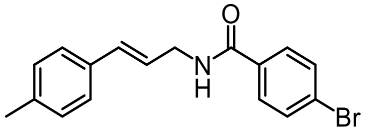

34a: R_f: 0.36 (PE/EtOAc (V/V) = 5:1); white solid, 82% yield. ^{1}H NMR (500 MHz, $CDCl_3$) δ 7.67-7.66 (d, *J* = 8.5 Hz, 2H), 7.58-7.56 (d, *J* = 8.5 Hz, 2H), 7.27-7.25 (d, *J* = 8.5 Hz, 2H), 7.13-7.12 (d, *J* = 8.5 Hz, 2H), 6.58-6.54 (d, *J* = 15.5 Hz, 1H), 6.25-6.19 (m, 2H), 4.23-4.20 (dd, *J* = 6.0 Hz, 2H), 2.33 (s, 3H); ^{13}C NMR (126 MHz, $CDCl_3$) δ 166.3, 137.8, 133.6, 133.3, 132.8, 131.8, 129.3, 128.6, 126.3, 126.2, 123.9, 42.3, 21.2. LC-MS(EI): $[M+H]^+$, 330.1, 332.1.

4.3 General Procedure for Synthesis of 39a and 40a

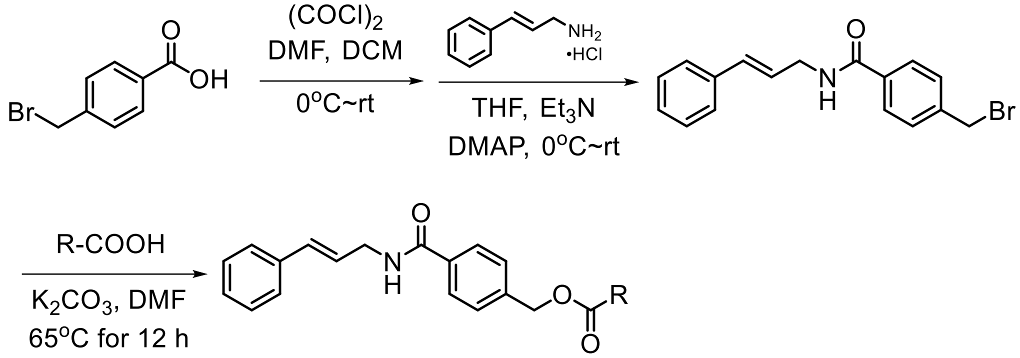

According to previous work[12], 4-bromomethylbenzoic acid (1.08 g, 5.0 mmol) was suspended in DCM, (30 ml), excess oxalyl chloride (2.54 g, 20.0 mmol) was added following by two drops of *N*,*N*-dimethylformamide (DMF). This mixture was stirred at room temperature until insoluble substance completely dissolved. Then the solvent was evaporated under vacuum, and the residue was dissolved in tetrohydrofuran (THF, 10 ml). This acyl chloride solution was then added drop wise to a THF (20 ml) solution of cinnamon ammonium chloride salt (5.0 mmol) and triethylamine (TEA, 5.0 equiv) cooled in an ice-water bath. The reaction mixture was stirred for 1 hours, then diluted with EtOAc (50 ml), and washed with water (30 ml) and brine (30 ml). The organic layer was dried over Na_2SO_4, then concentrated under reduced pressure in the presence of silica gel. Column chromatography (EtOAc-Hexanes gradient elution: hexanes/EtOAc (V/V) = 5:1) gave the desired products: **4-(bromomethyl)- *N*-cinnamylbenzamide** (80%).

Following the previous work[13] with a modification: Acid (1.0 equiv) in DMF (10 mL) was added 4-(bromomethyl)-*N*-cinnamylbenzamide (1.5 equiv) and K_2CO_3 (1.73 g, 12.5 mmol, 2.5 equiv). The mixture was stirred at 65°C for 12 h. Water was added and extracted with EtOAc for 3 times. The combined organic layers were washed with brine and dried over sodium sulfate and concentrated under reduced pressure in the presence of silica gel. Column chromatography (EtOAc-Hexanes gradient elution: hexanes/EtOAc (V/V) = 5:1 ~ 2:1) gave the desired products **39a** and **40a**.

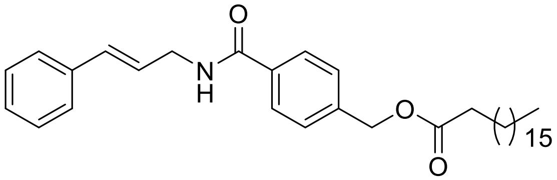

39a: R_f: 0.48 (hexanes/EtOAc (V/V) = 3:1); White solid, 65% yield of the total steps; White solid, 50% yield of the total steps. ^{1}H NMR (400 MHz, $CDCl_3$) δ 7.73-7.71 (d, J = 8.0 Hz, 2H), 7.35-7.33 (d, J = 8.0 Hz, 2H), 7.31-7.29 (d, J = 8.0 Hz, 2H), 7.26-7.22 (t, J = 8.0 Hz, 2H), 7.19-7.17 (d, J = 8.0 Hz, 2H), 6.55-6.51 (d, J = 16.0 Hz, 1H), 6.20 (b, 1H), 6.25-6.18 (dt, J = 16, 6.0 Hz, 1H), 5.08 (s, 2H), 4.20-4.17 (dd, J = 6.0 Hz, 2H), 2.31-2.27 (t, J = 8.0 Hz, 2H), 1.61-1.52 (m, 2H), 1.21-1.18 (m, 28H), 0.82-0.79 (t, J = 6.8 Hz, 3H); ^{13}C NMR (101 MHz, $CDCl_3$) δ 173.5, 166.8, 139.8, 136.4, 134.1, 132.7, 128.6, 128.0, 127.8, 127.2, 126.4, 125.3, 65.3, 42.2, 34.3, 31.9, 29.7 (2C), 29.6, 29.5, 29.4, 29.2, 29.1 (2C), 24.9, 22.7, 14.1. LC-MS(EI): $[M+H]^+$, 534.3.

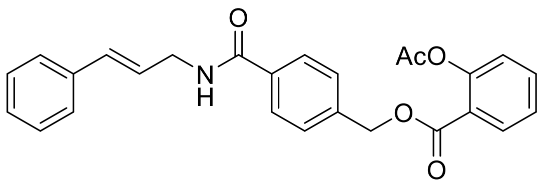

40a: R_f: 0.22 (hexanes/EtOAc (V/V) = 3:1); White solid, 50% yield of the total steps. ^1H NMR (400 MHz, $CDCl_3$) δ 8.80-8.06 (d, J = 8.0 Hz, 1H), 7.86-7.84 (d, J = 8.4 Hz, 2H), 7.61-7.57 (t, J = 8.0 Hz, 1H), 7.50-7.48 (d, J = 8.0 Hz, 2H), 7.40-7.38 (d, J = 8.0 Hz, 2H), 7.33-7.28 (t, J = 8.0 Hz, 3H),7.28-7.26 (d, J = 8.0 Hz, 1H), 7.14-7.12 (d, J = 8.0 Hz, 1H), 6.64-6.60 (d, J = 16.0 Hz, 1H), 6.38 (b, 1H), 6.34-6.27 (dt, J = 16, 6.0 Hz, 1H), 5.36 (s, 2H), 4.28-4.25 (dd, J = 6.0 Hz, 2H), 2.20 (s, 3H); ^{13}C NMR (101 MHz, $CDCl_3$) δ 169.6, 166.8, 164.2, 150.8, 139.2, 136.5, 134.4, 134.1, 132.6, 131.9, 128.6, 128.3, 127.8, 127.3, 126.4, 126.1, 125.3, 123.9, 123.0, 66.2, 42.2, 20.9. LC-MS(EI): $[M+H]^+$, 430.3.

4.4 General Procedure for Synthesis of 41a and 42a

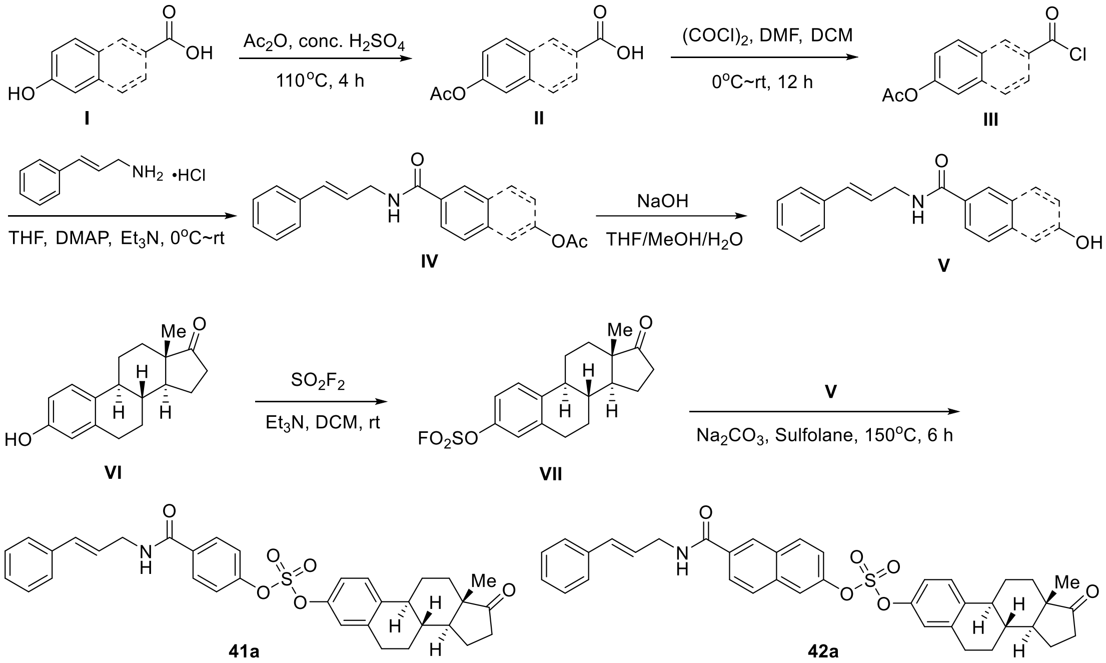

The acid **I** (10 mmol) was dissolved in acetic anhydride (100 mmol). To this solution, catalytic conc. H_2SO_4 was added. The solution was then heated at 110°C for 4 h, after which the deionized water (4 ml) was add to the solution to remove the unreacted acetic anhydride. The solvent was removed using a rotary evaporator under reduced pressure to yield white solid. The product was recrystallized in CH_2Cl_2 and dried under vacuum. Yielding 95% compound **II** as a white solid[14].

Compound **II** (5.0 mmol) was suspended in dichloromethane (DCM, 20 ml), excess oxalyl chloride (2.54 g, 20.0 mmol) was added following by two drops of *N*,*N*-dimethylformamide (DMF)[12]. This mixture was stirred at room temperature until insoluble substance completely dissolved. Then the solvent was evaporated under vacuum, and the residue was dissolved in tetrohydrofuran (THF, 10 ml). This acyl chloride solution was then added drop wise to a THF (20 ml) solution of cinnamon ammonium chloride salt (5.0 mmol) and triethylamine (TEA, 5.0 equiv) cooled in an ice-water bath. The reaction mixture was stirred for 3 hours (determined by TLC), then diluted with EtOAc (50 ml), and washed with water (30 ml) and brine (30 ml). The organic layer was dried over Na_2SO_4, then concentrated under reduced pressure in the presence of silica

gel. Column chromatography (EtOAc-Hexanes gradient elution: hexanes/EtOAc (V/V) = 3:1) gave the desired products **IV** (white solid, 80% yield).

IV (5.0 mmol, 1.0 equiv) was dissolved in 30 mL THF/MeOH (1:1). After addition of aqueous NaOH (1 M, 15.0 mL) the reaction mixture was stirred overnight at rt and then extracted with Et_2O (3 × 10 mL), the aqueous layers were acidified with aqueous HCl (3 M). After extraction with EtOAc (2 × 10 mL), the combined organic phases were washed with brine, dried over anhydrous Na_2SO_4 and concentrated under reduced pressure affording pure **V** as white solid (95%)[3].

In a 100 mL round-bottom flask equipped with a stir bar, estrone **VI** (10.0 mmol) was dissolved in 30 mL dichloromethane (DCM). Et_3N (25 mmol) was added and the resulting solution was stirred at room temperature for 10 mins. The flask was charged with gentle vacuum, then quickly filled with SO_2F_2 gas via a syringe attached balloon. The reaction was allowed stirring at room temperature until the full conversion of starting compound to target bisfluorosulfate, monitored by TLC and LC-MS. DCM was then evaporated away on rotary evaporator, the resulting crude product was dissolved in 30 mL ethyl acetate (EtOAc). It was subsequently washed with 30 mL aqueous HCl (1.0 M, 3 times), 30 mL saturated aqueous solution of $NaHCO_3$, then 30 mL saturated brine. The organic phase was dried over anhydrous Na_2SO_4. After filtration, the removal of EtOAc gave crude **VII**, which was further purified through recrystallization in EtOH to give the pure product **VII** as white solid (90% yield)[15].

Based on our previous work[16], compound **V** (5.0 mmol, 1.0 equiv) and **VI** (5.2 mmol, 1.04 equiv) were combined in a 25 mL glass vial equipped with a Teflon-coated magnetic stir bar. Sulfolane (10.0 mL) was added, and the vial was placed into a pre-heated 150°C oil bath. After 2 min, commercially available anhydrous K_2CO_3 (1.1 equiv) was added in one portion. The reaction was run for 6 hours. At the end of the reaction, it was allowed to cool to rt and the mixture was slowly poured into 20 mL of water and then extracted with EtOAc (20 mL × 2). The organic layer was collected and dried over Na_2SO_4, then concentrated under reduced pressure in the presence of silica gel. Column chromatography (EtOAc-Hexanes gradient elution: hexanes/EtOAc (V/V) = 5:1~3:1) gave the desired products **41a or 42a** (white solid, 80% yield for **41a** and 70% yield for **42a**).

NMR data of **41a**: ^{1}H NMR (500 MHz, $CDCl_3$) δ 7.82-7.80 (d, J = 8.0 Hz, 2H), 7.30-7.20 (m, 8H), 6.98-6.96 (d, J = 8.0 Hz, 1H), 6.94 (s, 1H), 6.68 (b, 1H), 6.50-6.47 (d, J = 15.5 Hz, 1H), 6.21-6.15 (m, 1H), 4.15-4.12 (t, J = 5.5 Hz), 2.83-2.82 (m, 2H), 2.44-2.39 (dd, J = 19.0, 8.5 Hz, 1H), 2.29-2.28 (m, 1H), 2.20-2.16 (t, J = 6.0 Hz, 1H), 2.11-1.99 (m, 3H), 2.04-1.96 (m, 3H), 1.88-1.86 (m, 1H), 1.54-1.49 (m, 2H), 1.43-1.36 (m, 4H), 0.81 (s, 3H); ^{13}C NMR (126 MHz, $CDCl_3$) δ 220.7, 166.0, 152.4, 148.3, 139.7, 139.1, 136.4, 133.7, 132.6, 129.2, 128.6, 127.9, 127.1, 126.4, 125.1, 121.1, 120.9, 118.0, 50.3, 47.9, 44.7, 42.3, 37.8, 35.8, 31.5, 29.4, 26.1, 25.7, 21.6, 13.8.

NMR data of **42a**: ^{1}H NMR (500 MHz, $CDCl_3$) δ 8.36 (s, 1H), 7.98-7.96 (d, J = 8.5 Hz, 1H), 7.94-7.88 (m, 2H), 7.84 (s, 1H), 7.49-7.48 (d, J = 8.5 Hz, 1H), 7.37-7.26 (m, 6H), 7.11-7.09 (d, J = 8.5 Hz, 1H), 7.06 (s, 1H), 6.65-6.62 (d, J = 16.0 Hz, 1H), 6.56 (b, 1H), 6.36-6.30 (m, 1H), 4.32-4.30 (t, J = 6.0 Hz, 2H), 2.92-2.90 (m, 2H), 2.54-2.48 (dd, J = 19.0, 8.5 Hz, 1H), 2.40-2.38 (m, 1H), 2.31-2.27 (t, J = 6.0 Hz, 1H), 2.17-2.11 (m, 1H), 2.04-1.96 (m, 3H), 1.67-1.61 (m, 3H), 1.51-1.45 (m, 3H), 0.91 (s, 3H); ^{13}C NMR (126 MHz, $CDCl_3$) δ 220.6, 166.8, 149.2, 148.4, 139.6, 139.1, 136.4, 134.9, 132.9, 132.6, 131.4, 131.3, 128.7, 128.6, 127.9, 127.4, 127.1, 126.4, 125.2, 125.0,

121.0, 120.8, 118.5, 118.1, 50.4, 47.9, 44.1, 42.4, 37.8, 35.8, 31.5, 29.4, 26.2, 25.7, 21.6, 13.8. LC-MS(EI): $[M+H]^+$, 636.3.

4.5 General Procedure for Synthesis of 43a-54a

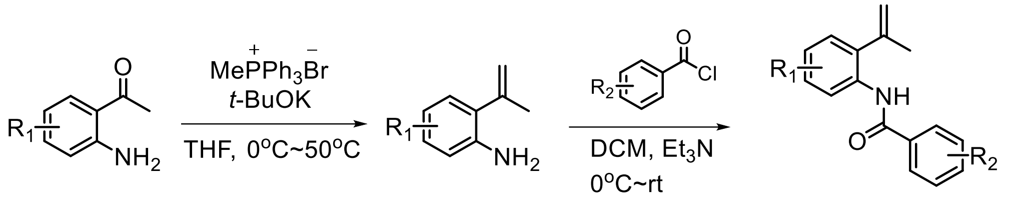

Under argon, an oven-dried round-bottom flask equipped with a magnetic stir bar was charged with Methyltriphenylphosphonium bromide (5.36g, 15 mmol), Potassium tert-butoxide (2.24g, 20 mmol) and anhydrous THF (10 ml) were added at 0°C. After the round-bottom flask was stirred at room temperature for 30 min, 2-Aminoacetophenone (1.35g, 10 mmol) was added to the reaction mixture. Then, the reaction mixture was stirred at 50°C overnight. After cooling to room temperature, the solvent was evaporated under vacuum. The residue was dissolved in water (50 mL) and extracted with EA (50 mL × 3). The combined organic layers were washed with saturated NaCl solution (50 mL × 3), dried (anhydrous Na_2SO_4), filtered over Celite, and evaporated in vacuo. The crude mixture was directly purified by silica gel column chromatography (eluent: PE/EA = 25:1) to give the corresponding product **2-(prop-1-en-2-yl)anilines** (90-92%)[17].

In an oven-dried round-bottom flask equipped with a magnetic stir bar, **2-(prop-1-en-2-yl)aniline** (266 mg, 2.0 mmol, 1.0 equiv) was added. Next, the flask was evacuated and backfilled with nitrogen three times. Triethylamine (2.0 equiv) and anhydrous THF (5 mL) were added into the flask. After the mixture was stirred at 0°C for 10 minutes, Benzoyl chloride (1.1 equiv) was slowly added to the reaction mixture. The reaction was stirred at rt and monitored by TLC. After 12 h, the mixture was poured into water (30 mL) and extracted with EA (3 × 30 mL). The combined organic layers were dried (anhydrous Na_2SO_4), filtered over Celite, and evaporated in vacuo. The residue was purified by column chromatography on silica gel to afford the substrate as a white solid[17].

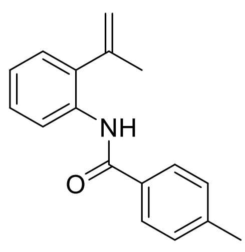

43a: R_f: 0.11 (PE/EtOAc (V/V) = 50:1); white solid, 95% yield.
^{1}H NMR (400 MHz, $CDCl_3$) δ 8.53-8.51 (d, *J* = 8.0 Hz, 1H), 8.45 (b, 1H), 7.77-7.75 (d, *J* = 8.0 Hz, 2H), 7.37-7.30 (m, 3H), 7.22-7.20 (d, *J* = 8.0 Hz, 1H), 7.15-7.12 (t, *J* = 8.0 Hz, 1H), 5.50 (s, 1H), 5.14 (s, 1H), 2.45 (s, 3H), 2.14 (s, 3H). ^{13}C NMR (101 MHz, $CDCl_3$) δ 165.0, 143.4, 142.3, 134.2, 133.5, 132.3, 129.5, 128.0, 127.7, 126.9, 123.8, 120.8, 116.8, 24.6, 21.5. LC-MS(EI): $[M+H]^+$, 252.2.

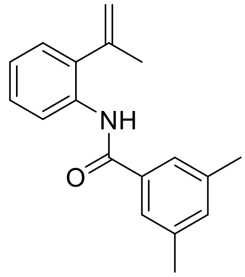

44a: R_f: 0.11 (PE/EtOAc (V/V) = 50:1); white solid, 90% yield.
^{1}H NMR (400 MHz, $CDCl_3$) δ 8.34-8.32 (d, *J* = 8.0 Hz, 1H), 8.32 (b, 1H), 7.31 (s, 2H), 7.19-7.16 (t, *J* = 8.0 Hz, 1H), 7.06-7.05 (d, *J* = 8.0 Hz, 1H), 7.03 (s, 1H), 6.99-6.96 (t, *J* = 8.0 Hz, 1H), 5.33 (s, 1H), 4.99 (s, 1H), 2.25 (s, 6H), 1.99 (s, 3H). ^{13}C NMR (101 MHz, $CDCl_3$) δ 165.6, 143.4, 138.6, 135.2, 134.2, 133.7, 133.4, 128.0, 127.7, 124.8, 123.9, 121.0, 116.7, 24.6, 21.4. LC-MS(EI): $[M+H]^+$, 266.3.

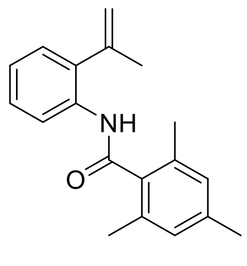

45a: R_f: 0.08 (PE/EtOAc (V/V) = 50:1); white solid, 88% yield.
^{1}H NMR (500 MHz, $CDCl_3$) δ 8.50-8.48 (d, *J* = 8.5 Hz, 1H), 7.63 (b, 1H), 7.35-7.32 (t, *J* = 8.5 Hz, 1H), 7.17-7.16 (d, *J* = 7.5 Hz, 1H), 7.14-7.11 (d, *J* = 9.0 Hz, 1H), 6.89 (s, 2H), 5.28 (s, 1H), 4.97 (s, 1H), 2.35 (s, 6H), 2.30 (s, 3H), 2.03 (s, 3H).^{13}C NMR (126 MHz, $CDCl_3$) δ 168.8, 142.6, 138.8, 135.2, 134.3, 133.9, 133.7, 128.4, 128.0, 127.9, 124.1, 121.1, 117.2, 24.7, 21.1, 19.2. LC-MS(EI): $[M+H]^+$, 280.2.

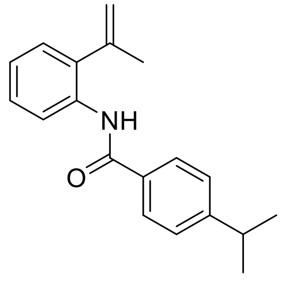

46a: R_f: 0.11 (PE/EtOAc (V/V) = 50:1); white solid, 92% yield.
^{1}H NMR (400 MHz, $CDCl_3$) δ 8.54-8.52 (d, *J* = 8.4 Hz, 1H), 8.49 (b, 1H), 7.82-7.80 (d, *J* = 8.0 Hz, 2H), 7.39-7.37 (d, *J* = 8.4 Hz, 2H), 7.35-7.33 (d, *J* = 8.0 Hz, 1H), 7.23-7.21 (d, *J* = 8.0 Hz, 1H), 7.16-7.12 (t, *J* = 8.0 Hz, 1H), 5.51 (s, 1H), 5.15 (s, 1H), 3.06-2.96 (m, 1H), 2.15 (s, 3H), 1.33-1.31 (d, *J* = 6.8 Hz, 6H). ^{13}C NMR (101 MHz, $CDCl_3$) δ 165.0, 153.1, 143.4, 134.2, 133.6, 132.7, 128.0, 127.7, 127.1, 126.9, 123.8, 120.8, 116.8, 34.1, 24.6, 23.8. LC-MS(EI): $[M+H]^+$, 280.3.

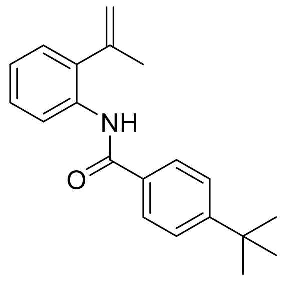

47a: R_f: 0.13 (PE/EtOAc (V/V) = 50:1); white solid, 95% yield.
^{1}H NMR (400 MHz, $CDCl_3$) δ 8.53-8.53 (d, *J* = 8.0 Hz, 1H), 8.50 (b, 1H), 7.83-7.81 (d, *J* = 8.4 Hz, 2H), 7.56-7.53 (d, *J* = 8.4 Hz, 2H), 7.37-7.33 (t, *J* = 8.4 Hz, 1H), 7.23-7.21 (d, *J* = 8.0 Hz, 1H), 7.16-7.12 (t, *J* = 8.4 Hz, 1H), 5.51 (s, 1H), 5.16 (s, 1H), 2.15 (s, 3H), 1.40 (s, 9H). ^{13}C NMR (101 MHz, $CDCl_3$) δ 165.0, 155.4, 143.4, 134.2, 133.5, 132.3, 128.0, 127.7, 126.8, 125.8, 123.8, 120.8, 116.8, 35.0, 31.2, 24.6. LC-MS(EI): $[M+H]^+$, 294.3.

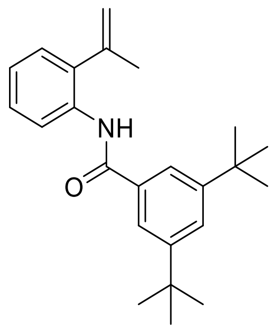

48a: R_f: 0.19 (PE/EtOAc (V/V) = 50:1); white solid, 92% yield.
^{1}H NMR (500 MHz, $CDCl_3$) δ 8.60-8.59 (d, *J* = 8.0 Hz, 1H), 8.60 (b, 1H), 7.71 (s, 2H), 7.64 (s, 1H), 7.38-7.34 (t, *J* = 8.5 Hz, 1H), 7.22-7.21 (d, *J* = 9.0 Hz, 1H), 7.15-7.12 (t, *J* = 8.5 Hz, 1H), 5.52 (s, 1H), 5.18 (s, 1H), 2.16 (s, 3H), 1.40 (s, 18H). ^{13}C NMR (126 MHz, $CDCl_3$) δ 166.0, 151.6, 143.7, 134.8, 134.4, 133.2, 128.1, 127.6, 126.0, 123.6, 121.2, 120.1, 116.6, 35.1, 31.4, 24.8. LC-MS(EI): $[M+H]^+$, 350.4.

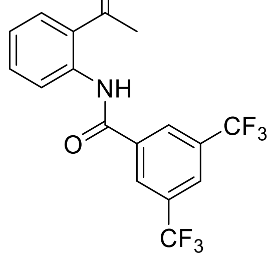

49a: R_f: 0.17 (PE/EtOAc (V/V) = 50:1); white solid, 90% yield.
^{1}H NMR (400 MHz, $CDCl_3$) δ 8.55 (b, 1H), 8.42-8.40 (d, *J* = 8.0 Hz, 1H), 8.29 (s, 2H), 8.08 (s, 1H), 7.38-7.35 (t, *J* = 8.0 Hz, 1H), 7.27-7.19 (m, 2H), 5.54 (s, 1H), 5.16 (s, 1H), 2.16 (s, 3H). ^{13}C NMR (101 MHz, $CDCl_3$) δ 162.1, 143.4, 137.3, 134.0, 133.1, 133.1-132.0 (q, *J* = 33.9 Hz), 128.2, 127.0, 127.2, 125.3-125.2 (q, *J* = 3.5 Hz), 124.2, 121.5, 121.0, 116.8, 24.6. ^{19}F NMR (376 MHz, $CDCl_3$) δ -63.11. LC-MS(EI): $[M+H]^+$, 374.2.

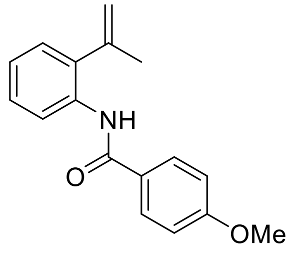

50a: R_f: 0.11 (PE/EtOAc (V/V) = 20:1); white solid, 92% yield.
^{1}H NMR (400 MHz, $CDCl_3$) δ 8.48-8.46 (d, *J* = 8.0 Hz, 1H), 8.38 (b, 1H), 7.80-7.78 (d, *J* = 8.0 Hz, 2H), 7.34-7.29 (t, *J* = 8.0 Hz, 1H), 7.19-7.17 (d, *J* = 8.0 Hz, 1H), 7.12-7.08 (t, *J* = 8.0 Hz, 1H), 6.99-6.97 (d, *J* = 8.0 Hz, 2H), 5.47 (s, 1H), 5.11 (s, 1H), 3.87 (s, 3H), 2.12 (s, 3H). ^{13}C NMR (101 MHz, $CDCl_3$) δ 164.6, 162.5, 143.4, 134.2, 133.4, 128.8, 128.1, 127.6, 127.3, 123.7, 120.7, 116.8, 114.1, 22.5, 24.7. LC-MS(EI): $[M+H]^+$, 268.2.

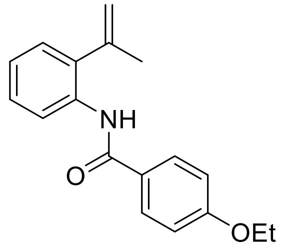

51a: R_f: 0.12 (PE/EtOAc (V/V) = 50:1); white solid, 93% yield.
^{1}H NMR (400 MHz, $CDCl_3$) δ 8.50-8.48 (d, *J* = 8.0 Hz, 1H), 8.38 (b, 1H), 7.81-7.79 (d, *J* = 8.0 Hz, 2H), 7.35-7.32 (t, *J* = 8.0 Hz, 1H), 7.21-7.19 (d, *J* = 8.0 Hz, 1H), 7.14-7.10 (t, *J* = 8.0 Hz, 1H), 7.00-6.97 (d, *J* = 8.8 Hz, 2H), 5.50 (s, 1H), 5.14 (s, 1H), 4.15-4.10 (q, 2H, *J* = 6.8 Hz), 2.14 (s, 3H), 1.49-1.45 (t, *J* = 7.2 Hz, 3H). ^{13}C NMR (101 MHz, $CDCl_3$) δ 164.6, 161.9, 143.5, 134.3, 133.4, 128.8, 128.0, 127.6, 127.2, 123.6, 120.7, 116.7, 114.5, 63.7, 24.6, 14.7. LC-MS(EI): $[M+H]^+$, 282.2.

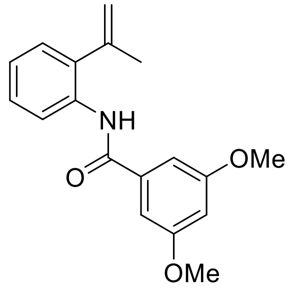

52a: R_f: 0.11 (PE/EtOAc (V/V) = 20:1); white solid, 85% yield.
^{1}H NMR (400 MHz, $CDCl_3$) δ 8.47-8.45 (d, *J* = 8.0 Hz, 1H), 8.41 (b, 1H), 7.34-7.31 (t, *J* = 8.0 Hz, 1H), 7.20-7.18 (d, *J* = 8.0 Hz, 1H), 7.14-7.10 (t, *J* = 8.0 Hz, 1H), 6.95 (s, 2H), 6.62 (s, 1H),5.48 (s, 1H), 5.12 (s, 1H), 3.87 (s, 6H), 2.11 (s, 3H). ^{13}C NMR (101 MHz, $CDCl_3$) δ 164.9, 161.1, 143.3, 137.3, 134.0, 133.6, 128.1, 127.7, 124.0, 120.7, 116.9, 104.9, 103.8, 55.6, 24.7. LC-MS(EI): $[M+H]^+$, 298.2.

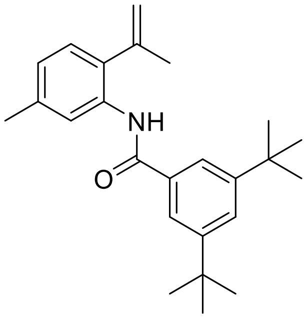

53a: R_f: 0.23 (PE/EtOAc (V/V) = 50:1); white solid, 85% yield.
^{1}H NMR (400 MHz, $CDCl_3$) δ 8.49 (b, 1H), 8.36 (s, 1H), 7.60 (s, 2H), 7.53 (s, 1H), 7.10-6.98 (d, *J* = 8.0 Hz, 1H), 6.85-6.83 (d, *J* = 8.0 Hz, 1H), 5.39 (s, 1H), 5.04 (s, 1H), 2.31 (s, 3H), 2.03 (s, 3H), 1.29 (s, 18H). ^{13}C NMR (101 MHz, $CDCl_3$) δ 165.9, 151.5, 143.7, 138.1, 134.8, 134.2, 130.3, 127.4, 126.0, 124.4, 121.2, 120.7, 116.4, 35.1, 31.4, 24.9, 21.6. LC-MS(EI): $[M+H]^+$, 364.3.

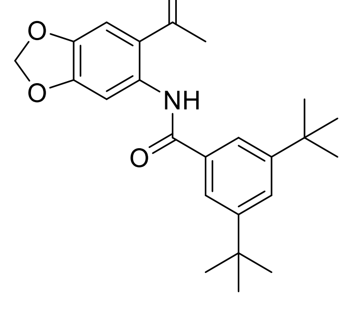

54a: R_f: 0.07 (PE/EtOAc (V/V) = 50:1); white solid, 85% yield.
^{1}H NMR (400 MHz, $CDCl_3$) δ 8.38 (b, 1H), 8.04 (s, 1H), 7.57 (s, 2H), 7.52 (s, 1H), 6.56 (s, 1H), 5.86 (s, 2H), 5.37 (s, 1H), 5.02 (s, 1H), 1.99 (s, 3H), 1.27 (s, 18H). ^{13}C NMR (101 MHz, $CDCl_3$) δ 165.7, 151.5, 146.9, 143.6, 134.7, 128.6, 126.6, 125.9, 121.2, 116.8, 107.4, 102.6, 101.3, 35.1, 31.4, 24.8. LC-MS(EI): $[M+H]^+$, 394.2.

5. Procedures for Asymmetric Aminofluorination and Characterization of Products

5.1 General Procedure A (Synthesis of 1b-42b)

The substrate (0.2 mmol) and catalyst (15 mol%) were mixed into the reaction tube, and then DCE (8.0 ml) was added. The mixture was cooled to −25°C, after stirring for 10 min at this temperature, *m*-CPBA (1.2 equiv.) was added in one portion, followed by addition of $BF_3\cdot Et_2O$ (10.0 equiv.) dropwise. The reaction was run at −25°C for 36 h. The reaction mixture was poured into *s*-$NaHCO_3$ (aq), the organic layer was collected and washed with brine, dried over Na_2SO_4 concentrated under reduced pressure in the presence of basic Al_2O_3, Column chromatography (EtOAc-hexane (0.5% Et_3N) elution: hexane/EtOAc (V/V) = 50:1~5:1) gave the corresponding fluorinated products.

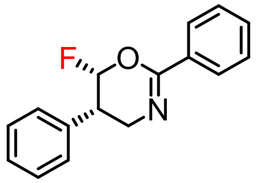

1b: Prepared according to general procedure A using **1a** (47.4 mg, 0.2 mmol). After work-up, the crude residue was purified by flash column chromatography on basic Al_2O_3 (EtOAc-Hexanes (0.5% Et_3N) elution: hexanes/EtOAc (V/V) = 50:1) to give **1b** (35.7 mg, 70%) as a white solid. R_f = 0.21 (PE/EA (V/V) = 25:1). **1b** was determined to be of 86% e.e. by HPLC (phenomenex Cellulose-1, *i*-PrOH/Hexanes (V/V) = 95:5, 1.0 mL/min; t_r (major) = 13.843 min, t_r (minor) = 12.347 min). **1b** was determined to be of > 20:1 dr by ^{19}F NMR. ^{1}H NMR (400 MHz, $CDCl_3$) δ 7.99−7.97 (d, *J* = 8.0 Hz, 2H), 7.45-7.33 (m, 8H), 6.17-6.02 (dd, *J* = 3.2, 56.8 Hz, 1H), 4.05-3.97 (m, 1H), 3.87-3.80 (m, 1H), 3.30-3.18 (dddd, *J* = 1.2, 6.0, 7.2, 29.6 Hz, 1H); ^{13}C NMR (126 MHz, $CDCl_3$) δ 151.5, 136.1, 132.3, 131.0, 128.9, 128.2, 127.9, 105.0 (d, *J* = 228.7 Hz), 43.5 (d, *J* = 5.0 Hz), 41.7 (d, *J* = 21.3 Hz); ^{19}F NMR (376 MHz, $CDCl_3$) δ -117.2 (s, 1F), -132.0 (s, 25F). HRMS (ESI-TOF) Calc'd for $C_{16}H_{14}FNO$ $[M+H]^+$: 256.1132; found 256.1130.

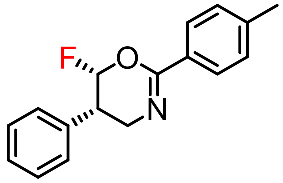

2b: Prepared according to general procedure A using **1a** (50.2 mg, 0.2 mmol). After work-up, the crude residue was purified by flash column chromatography on basic Al_2O_3 (EtOAc-Hexanes (0.5% Et_3N) elution: hexanes/EtOAc (V/V) = 25:1) to give **2b** (38.2 mg, 71%) as a white solid. R_f = 0.14 (PE/EA (V/V) = 25:1). **2b** was determined to be of 99% e.e. by HPLC (phenomenex Lux Amylose-1, *i*-PrOH/Hexanes (V/V) = 98:2, 1.0 mL/min; t_r (major) = 16.807 min, t_r (minor) = 19.600 min). **2b** was determined to be of > 20:1 dr by ^{19}F NMR. ^{1}H NMR (400 MHz, $CDCl_3$) δ 7.88−7.86 (d, *J* = 8.0 Hz, 2H), 7.41-7.33 (m, 5H), 7.22-7.25 (d, *J* = 8.0 Hz, 2H), 6.15-5.99 (dd, *J* = 3.2, 56.8 Hz, 1H), 4.04-3.96 (m, 1H), 3.89-3.78 (m, 1H), 3.29-3.17 (m, 1H), 3.06 (s, 3H); ^{13}C NMR (101 MHz, $CDCl_3$) δ 151.5, 141.3, 136.2, 129.6, 129.0, 128.9, 128.5, 127.9, 127.2, 105.0 (d, *J* = 228.9 Hz), 43.5 (d, *J* = 5.1 Hz), 41.7 (d, *J* = 21.4 Hz); ^{19}F NMR (376 MHz, $CDCl_3$) δ -117.2 (s, 1F), -132.0 (s, 25F). HRMS (ESI-TOF) Calc'd for $C_{17}H_{16}FNO$ $[M+H]^+$: 270.1289; found 270.1278.

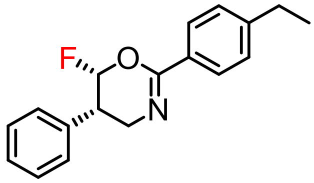

3b: Prepared according to general procedure A using **3a** (53.0 mg, 0.2 mmol). After work-up, the crude residue was purified by flash column chromatography on basic Al_2O_3 (EtOAc-Hexanes (0.5% Et_3N) elution: hexanes/EtOAc (V/V) = 25:1) to give **3b** (40.7 mg, 72%) as a white solid. R_f = 0.33 (PE/EA (V/V) = 10:1). **3b** was determined to be of 91% e.e. by HPLC (phenomenex Lux Amylose-1, *i*-PrOH/Hexanes (V/V) = 98:2, 1.0 mL/min; t_r (major) = 13.673 min, t_r (minor) = 13.017 min). **3b** was determined to be of >

20:1 dr by ^{19}F NMR. ^{1}H NMR (500 MHz, CDCl$_3$) δ 7.90−7.88 (d, *J* = 8.0 Hz, 2H), 7.41-7.32 (m, 5H), 7.25-7.23 (d, *J* = 8.0 Hz, 2H), 6.14-6.02 (dd, *J* = 3.0, 57.0 Hz, 1H), 4.08-3.96 (m, 1H), 3.84-3.80 (m, 1H), 3.27-3.19 (dddd, *J* = 1.2, 6.0, 7.5, 29.6 Hz, 1H), 2.71-2.67 (q, *J* = 7.5 Hz, 2H), 1.27-1.24 (t, *J* = 7.5 Hz, 3H); ^{13}C NMR (126 MHz, CDCl$_3$) δ 151.6, 147.6, 136.2, 129.8, 128.9, 128.5, 127.9, 127.8, 127.3, 1050 (d, *J* = 227.9 Hz), 43.5 (d, *J* = 5.0 Hz), 41.7 (d, *J* = 21.3 Hz), 28.8, 15.4; ^{19}F NMR (471 MHz, CDCl$_3$) δ -117.1 (s, 1F), -132.0 (s, 29F). HRMS (ESI-TOF) Calc'd for $C_{18}H_{18}FNO$ [M+H]$^+$: 284.1445; found 284.1445.

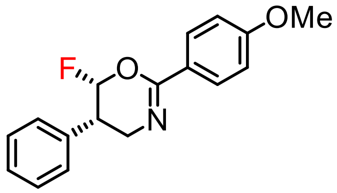

4b: Prepared according to general procedure A using **4a** (53.5 mg, 0.2 mmol). After work-up, the crude residue was purified by flash column chromatography on basic Al_2O_3 (EtOAc-Hexanes (0.5% Et_3N) elution: hexanes/EtOAc (V/V) = 10:1) to give **4b** (42.8 mg, 75%) as a white solid. R_f = 0.13 (PE/EA (V/V) = 10:1). **4b** was determined to be of 99% e.e. by HPLC (phenomenex Lux Amylose-1, *i*-PrOH/Hexanes (V/V) = 90:10, 1.0 mL/min; t_r (major) = 12.703 min, t_r (minor) = 9.537 min). **4b** was determined to be of > 20:1 dr by ^{19}F NMR. ^{1}H NMR (500 MHz, CDCl$_3$) δ 7.93−7.92 (d, *J* = 9.0 Hz, 2H), 7.39-7.33 (m, 4H), 7.27-7.26 (d, *J* = 9.0 Hz, 2H), 6.92-6.90 (d, *J* = 8.5 Hz, 2H) 6.14-6.01 (dd, *J* = 3.0, 57.0 Hz, 1H), 4.02-3.96 (m, 1H), 3.85-3.78 (m, 4H), 3.27-3.18 (m, 1H); ^{13}C NMR (126 MHz, CDCl$_3$) δ 162.9, 151.2, 136.3, 128.9, 128.8, 128.5, 127.9, 124.8, 113.5, 105.0 (d, *J* = 228.7 Hz), 55.4, 43.4 (d, *J* = 4.6 Hz), 41.7 (d, *J* = 21.5 Hz); ^{19}F NMR (471 MHz, CDCl$_3$) δ -117.2 (s, 1F), -132.0 (s, 26F). HRMS (ESI-TOF) Calc'd for $C_{17}H_{16}FNO_2$ [M+H]$^+$: 286.1238; found 286.1237.

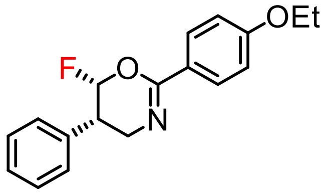

5b: Prepared according to general procedure A using **5a** (56.3 mg, 0.2 mmol). After work-up, the crude residue was purified by flash column chromatography on basic Al_2O_3 (EtOAc-Hexanes (0.5% Et_3N) elution: hexanes/EtOAc (V/V) = 10:1) to give **5b** (40.1 mg, 67%) as a white solid. R_f = 0.18 (PE/EA (V/V) = 10:1). **5b** was determined to be of 94% e.e. by HPLC (phenomenex Cellulose-1, *i*-PrOH/Hexanes (V/V) = 98:2, 1.0 mL/min; t_r (major) = 28.147 min, t_r (minor) = 23.373 min). **5b** was determined to be of > 20:1 dr by ^{19}F NMR. ^{1}H NMR (400 MHz, CDCl$_3$) δ 7.86−7.83 (d, *J* = 9.2 Hz, 2H), 7.32-7.22 (m, 5H), 6.84-6.82 (d, *J* = 9.2 Hz, 2H), 6.08-5.93 (dd, *J* = 3.6, 56.8 Hz, 1H), 4.03-3.98 (q, *J* = 7.2 Hz, 3H), 3.95-3.87 (m, 1H), 3.76-3.70 (m, 1H), 3.22-3.09 (dddd, *J* = 1.2, 5.6, 12.8, 30.4 Hz, 1H); ^{13}C NMR (101 MHz, CDCl$_3$) δ 161.4, 136.2, 129.0, 128.9, 128.49, 128.47, 127.9, 127.7, 114.1, 105.0 (d, *J* = 229.0 Hz), 63.6, 43.4 (d, *J* = 4.6 Hz), 41.7 (d, *J* = 21.5 Hz), 14.8; ^{19}F NMR (376 MHz, CDCl$_3$) δ -117.3 (s, 1F), -132.1 (s, 21F). HRMS (ESI-TOF) Calc'd for $C_{18}H_{18}FNO_2$ [M+H]$^+$: 300.1394; found 300.1393.

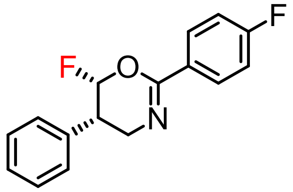

6b: Prepared according to general procedure A using **6a** (51.1 mg, 0.2 mmol). After work-up, the crude residue was purified by flash column chromatography on basic Al_2O_3 (EtOAc-Hexanes (0.5% Et_3N) elution: hexanes/EtOAc (V/V) = 25:1) to give **6b** (38.8 mg, 71%) as a white solid. R_f = 0.18 (PE/EA (V/V) = 25:1). **6b** was determined to be of 92% e.e. by HPLC (phenomenex Cellulose-1, *i*-PrOH/Hexanes (V/V) = 95:5, 1.0 mL/min; t_r (major) = 14.740 min, t_r (minor) = 12.807 min). **6b** was determined to be of 11:1 dr by ^{19}F NMR. ^{1}H NMR (400 MHz, CDCl$_3$) δ 7.92−7.90 (d, *J* = 8.8 Hz, 1H), 7.91-7.89 (d, *J* = 8.8 Hz, 1H), 7.31-7.26 (m, 5H), 7.03-6.98 (t, *J* = 8.8 Hz, 2H), 6.08-5.93 (dd, *J* = 3.6, 56.4 Hz, 1H), 3.95-

3.87 (m, 1H), 3.77-3.71 (m, 1H), 3.21-3.09 (dddd, J = 1.2, 5.6, 13.2, 30.0 Hz, 1H); ^{13}C NMR (101 MHz, $CDCl_3$) δ 164.6 (d, J = 251.0 Hz), 150.6, 136.0, 129.5 (d, J = 8.8 Hz), 128.9, 128.5, 128.0, 115.3 (d, J = 21.9 Hz), 105.0 (d, J = 229.5 Hz), 43.5 (d, J = 5.0 Hz), 41.7 (d, J = 21.3 Hz); ^{19}F NMR (376 MHz, $CDCl_3$) δ -109.2 (s, 12F) -117.3 (s, 1F), -132.1 (s, 11F). HRMS (ESI-TOF) Calc'd for $C_{16}H_{13}F_2NO$ $[M+H]^+$: 274.1038; found 274.1035.

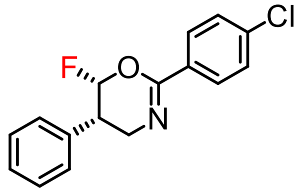

7b: Prepared according to general procedure A using **7a** (54.2 mg, 0.2 mmol). After work-up, the crude residue was purified by flash column chromatography on basic Al_2O_3 (EtOAc-Hexanes (0.5% Et_3N) elution: hexanes/EtOAc (V/V) = 50:1) to give **7b** (42.9 mg, 74%) as a white solid. R_f = 0.24 (PE/EA (V/V) = 25:1). **7b** was determined to be of 91% e.e. by HPLC (phenomenex Lux Amylose-1, *i*-PrOH/Hexanes (V/V) = 99:1, 1.0 mL/min; t_r (major) = 18.547 min, t_r (minor) = 21.227 min). **7b** was determined to be of > 20:1 dr by ^{19}F NMR. ^{1}H NMR (400 MHz, $CDCl_3$) δ 7.92−7.90 (d, J = 8.4 Hz, 2H), 7.39-7.34 (m, 7H), 6.15-6.00 (dd, J = 4.0, 56.4 Hz, 1H), 4.03-3.95 (m, 1H), 3.85-3.79 (m, 1H), 3.29-3.16 (dddd, J = 1.2, 5.6, 13.2, 30.0 Hz, 1H); ^{13}C NMR (101 MHz, $CDCl_3$) δ 150.7, 137.3, 135.9, 130.8, 128.9, 128.7, 128.5, 128.1, 105.0 (d, J = 229.6 Hz), 43.5 (d, J = 5.0 Hz), 41.6 (d, J = 21.3 Hz); ^{19}F NMR (376 MHz, $CDCl_3$) δ -117.7 (s, 1F), -132.1 (s, 29F). HRMS (ESI-TOF) Calc'd for $C_{16}H_{13}FNOCl$ $[M+H]^+$: 290.0742; found 290.0739.

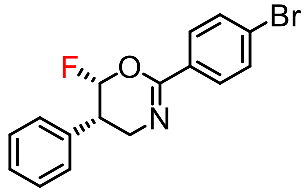

8b: Prepared according to general procedure A using **8a** (63.2 mg, 0.2 mmol). After work-up, the crude residue was purified by flash column chromatography on basic Al_2O_3 (EtOAc-Hexanes (0.5% Et_3N) elution: hexanes/EtOAc (V/V) = 50:1) to give **8b** (46.8 mg, 70%) as a white solid. R_f = 0.26 (PE/EA (V/V) = 25:1). **8b** was determined to be of 91% e.e. by HPLC (IC, *i*-PrOH/Hexanes (V/V) = 99.5:0.5, 0.2 mL/min; t_r (major) = 44.723 min, t_r (minor) = 41.867 min). **8b** was determined to be of > 20:1 dr by ^{19}F NMR. ^{1}H NMR (400 MHz, $CDCl_3$) δ 7.89−7.87 (d, J = 8.4 Hz, 2H), 7.58-7.56 (d, J = 8.4 Hz, 2H), 7.44-7.37 (m, 5H), 6.18-6.03 (dd, J = 3.6, 56.4 Hz, 1H), 4.05-3.97 (m, 1H), 3.88-3.81 (m, 1H), 3.32-3.19 (dddd, J = 1.2, 5.6, 13.2, 29.6 Hz, 1H); ^{13}C NMR (101 MHz, $CDCl_3$) δ 150.8, 135.9, 131.5, 128.9, 128.5, 128.4, 128.0, 125.7, 105.0 (d, J = 229.6 Hz), 43.5 (d, J = 5.0 Hz), 41.6 (d, J = 21.3 Hz); ^{19}F NMR (376 MHz, $CDCl_3$) δ -117.3 (s, 1F), -132.1 (s, 24F). HRMS (ESI-TOF) Calc'd for $C_{16}H_{13}FNOBr$ $[M+H]^+$: 334.0237, 336.0218; found 334.0223, 336.0189.

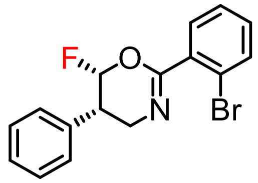

9b: Prepared according to general procedure A using **9a** (63.2 mg, 0.2 mmol). After work-up, the crude residue was purified by flash column chromatography on basic Al_2O_3 (EtOAc-Hexanes (0.5% Et_3N) elution: hexanes/EtOAc (V/V) = 50:1) to give **9b** (36.7 mg, 55%) as a white solid. R_f = 0.21 (PE/EA (V/V) = 25:1). **9b** was determined to be of 99% e.e. by HPLC (AD, i-PrOH/Hexanes (V/V) = 99:1, 0.8 mL/min; t_r (major) = 22.903 min). **9b** was determined to be of 11:1 dr by ^{19}F NMR. ^{1}H NMR (400 MHz, $CDCl_3$) δ 7.86-7.83 (d, J = 8.8 Hz, 2H), 7.32-7.25 (m, 7H), 6.08-5.93 (dd, J = 3.3, 56.8 Hz, 1H), 4.01-3.88 (m, 1H), 3.77-3.71 (m, 1H), 3.22-3.09 (dddd, J = 1.2, 5.6, 13.2, 29.6 Hz, 1H); ^{13}C NMR (101 MHz, $CDCl_3$) δ 150.7, 137.3, 135.9, 130.8, 128.9, 128.7, 128.51, 128.48, 128.46, 128.0, 127.2, 105.0 (d, J = 229.6 Hz), 43.5 (d, J = 5.0 Hz), 41.6 (d, J = 21.3 Hz); ^{19}F NMR (376 MHz, $CDCl_3$) δ -117.3 (s,

1F), -132.1 (s, 11F). HRMS (ESI-TOF) Calc'd for $C_{16}H_{13}FNOBr$ $[M+H]^+$: 334.0237, 336.0218; found 334.0232, 336.0215.

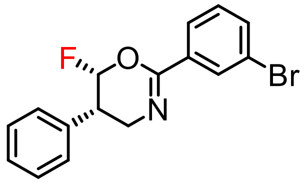

10b: Prepared according to general procedure A using **10a** (63.2 mg, 0.2 mmol). After work-up, the crude residue was purified by flash column chromatography on basic Al_2O_3 (EtOAc-Hexanes (0.5% Et_3N) elution: hexanes/EtOAc (V/V) = 50:1) to give **10b** (44.6 mg, 67%) as a white solid. R_f = 0.19 (PE/EA (V/V) = 25:1). **10b** was determined to be of 88% e.e. by HPLC (phenomenex Cellulose-1, *i*-PrOH/Hexanes (V/V) = 99:1, 1.0 mL/min; t_r (major) = 21.930 min, t_r (minor) = 17.773 min). **10b** was determined to be of 10:1 dr by ^{19}F NMR. ^1H NMR (400 MHz, $CDCl_3$) δ 8.07 (s, 1H), 7.85-7.83 (d, *J* = 8.0 Hz, 1H), 7.53-7.51 (d, *J* = 8.0 Hz, 1H), 7.32-7.27 (m, 5H), 7.21-7.18 (m, 1H), 6.09-5.94 (dd, *J* = 4.0, 56.8 Hz, 1H), 4.01-73 (m, 2H), 3.22-3.09 (dddd, *J* = 1.2, 5.6, 13.2, 30.0 Hz, 1H); ^{13}C NMR (101 MHz, $CDCl_3$) δ 150.3, 135.8, 134.0, 130.4, 129.8, 128.9, 128.5, 128.0, 127.2, 125.9, 122.4, 105.0 (d, *J* = 229.8 Hz), 43.5 (d, *J* = 5.0 Hz), 41.6 (d, *J* = 21.3 Hz); ^{19}F NMR (376 MHz, $CDCl_3$) δ -117.1 (s, 1F), -132.1 (s, 10F). HRMS (ESI-TOF) Calc'd for $C_{16}H_{13}FNOBr$ $[M+H]^+$: 334.0237, 336.0218; found 334.0228, 336.0200.

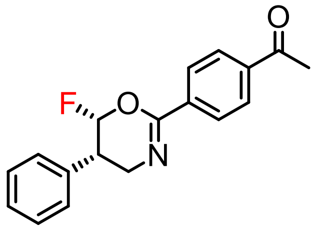

11b: Prepared according to general procedure A using **11a** (55.8 mg, 0.2 mmol). After work-up, the crude residue was purified by flash column chromatography on basic Al_2O_3 (EtOAc-Hexanes (0.5% Et_3N) elution: hexanes/EtOAc (V/V) = 10:1) to give **11b** (38.5 mg, 65%) as a white solid. R_f = 0.09 (PE/EA (V/V) = 10:1). **11b** was determined to be of 91% e.e. by HPLC (AD, *i*-PrOH/Hexanes (V/V) = 97:3, 1.0 mL/min; t_r (major) = 29.570 min, t_r (minor) = 36.287 min). **14b** was determined to be of 11:1 dr by ^{19}F NMR. ^1H NMR (400 MHz, $CDCl_3$) δ 8.09-8.07(d, 2H, *J* = 8.5 Hz, 2H), 8.00-7.98 (d, *J* = 8.5 Hz, 2H), 7.40-7.28 (m, 5H), 6.17-5.05 (dd, *J* = 3.2, 56.5 Hz, 1H), 4.14-4.00 (m, 1H), 3.91-3.85 (m, 1H), 3.31-3.21 (m, 1H), 2.64 (s, 3H); ^{13}C NMR (101 MHz, $CDCl_3$) δ 197.7, 150.8, 138.8, 136.4, 135.8, 129.1, 128.9, 128.7, 128.5, 128.2, 128.1, 128.0, 127.9, 127.6, 127.5, 127.4, 127.2, 106.4 (d, *J* = 229.3 Hz), 105.0 (d, *J* = 229.3 Hz), 44.4 (d, *J* = 4.9 Hz), 43.6 (d, *J* = 4.9 Hz), 41.6 (d, *J* = 21.2 Hz), 40.4 (d, *J* = 21.2 Hz), 26.8, 23.7; ^{19}F NMR (376 MHz, $CDCl_3$) δ -117.2 (s, 1F), -132.0 (s, 11F). HRMS (ESI-TOF) Calc'd for $C_{18}H_{16}FNO_2$ $[M+H]^+$: 298.1238; found 298.1237.

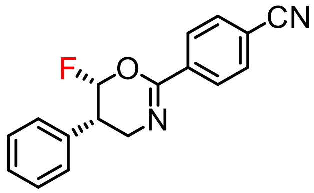

12b: Prepared according to general procedure A using **12a** (52.4 mg, 0.2 mmol). After work-up, the crude residue was purified by flash column chromatography on basic Al_2O_3 (EtOAc-Hexanes (0.5% Et_3N) elution: hexanes/EtOAc (V/V) = 20:1) to give **12b** (40.3 mg, 72%) as a white solid. R_f = 0.16 (PE/EA (V/V) = 10:1). **12b** was determined to be of 86% e.e. by HPLC (phenomenex Lux Amylose-1, *i*-PrOH/Hexanes (V/V) = 85:15, 0.4 mL/min; t_r (major) = 42.837 min, t_r (minor) = 47.440 min). **14b** was determined to be of 6:1 dr by ^{19}F NMR. ^1H NMR (400 MHz, $CDCl_3$) δ 8.03-8.01(d, *J* = 8.0 Hz, 2H), 7.65-7.63 (d, *J* = 8.0 Hz, 2H), 7.33-7.28 (m, 4H), 7.20-7.18 (t, *J* = 8.0 Hz, 1H), 6.10-5.96 (dd, *J* = 2.9, 56.0 Hz, 1H), 4.06-3.77 (m, 2H), 3.24-3.11 (dddd, *J* = 1.2, 5.6, 13.2, 30.0 Hz, 1H); ^{13}C NMR (101 MHz, $CDCl_3$) δ 150.1, 136.4, 135.5, 132.1, 132.0, 129.2, 129.0, 128.8, 128.5 (2C), 128.1, 127.9, 127.8, 127.6, 127.2, 118.4, 114.5, 104.9 (d, *J* = 230.4 Hz), 44.3 (d, *J* = 5.1 Hz), 43.7 (d, *J* = 5.1

Hz), 41.5 (d, J = 21.0 Hz), 40.3 (d, J = 21.0 Hz); ^{19}F NMR (376 MHz, $CDCl_3$) δ -117.2 (s, 1F), -132.1 (s, 6F). HRMS (ESI-TOF) Calc'd for $C_{17}H_{13}FN_2O$ $[M+H]^+$: 281.1085; found 281.1085.

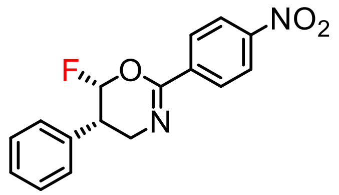

13b: Prepared according to general procedure A using **13a** (56.4 mg, 0.2 mmol). After work-up, the crude residue was purified by flash column chromatography on basic Al_2O_3 (EtOAc-Hexanes (0.5% Et_3N) elution: hexanes/EtOAc (V/V) = 10:1) to give **13b** (36.0 mg, 60%) as a white solid. R_f = 0.15 (PE/EA (V/V) = 10:1). **13b** was determined to be of 91% e.e. by HPLC (phenomenex Lux Amylose-1, *i*-PrOH/Hexanes (V/V) = 99:1, 1.0 mL/min; t_r (major) = 47.190 min, t_r (minor) = 66.607 min). **13b** was determined to be of 4:1 dr by ^{19}F NMR. ^{1}H NMR (500 MHz, $CDCl_3$) δ 8.19-8.17(d, J = 8.5 Hz, 2H), 8.09-8.07 (d, J = 9.0 Hz, 2H), 7.33-7.18 (m, 5H), 6.10-5.98 (dd, J = 3.0, 56.5 Hz, 1H), 4.06-3.80 (m, 2H), 3.25-3.14 (m, 1H); ^{13}C NMR (126 MHz, $CDCl_3$) δ 149.4, 138.0, 135.5, 129.0, 128.5, 128.3, 128.2, 127.6, 123.4, 105.0 (d, J = 230.0 Hz), 43.7 (d, J = 4.9 Hz), 41.5 (d, J = 21.2 Hz); ^{19}F NMR (471 MHz, $CDCl_3$) δ -117.2 (s, 1F), -132.0 (s, 4F). HRMS (ESI-TOF) Calc'd for $C_{16}H_{13}FN_2O_3$ $[M+H]^+$: 301.0983; found 301.0981.

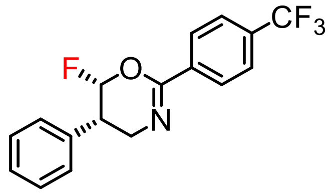

14b: Prepared according to general procedure A using **14a** (61.0 mg, 0.2 mmol). After work-up, the crude residue was purified by flash column chromatography on basic Al_2O_3 (EtOAc-Hexanes (0.5% Et_3N) elution: hexanes/EtOAc (V/V) = 50:1) to give **14b** (42.0 mg, 65%) as a white solid. R_f = 0.23 (PE/EA (V/V) = 25:1). **14b** was determined to be of 91% e.e. by HPLC (phenomenex Cellulose-1, *i*-PrOH/Hexanes (V/V) = 98:2, 1.0 mL/min; t_r (major) = 12.360 min, t_r (minor) = 10.500 min). **14b** was determined to be of > 20:1 dr by ^{19}F NMR. ^{1}H NMR (400 MHz, $CDCl_3$) δ 8.03-8.01(d, J = 8.0 Hz, 2H), 7.60-7.58 (d, J = 8.0 Hz, 2H), 7.32-7.27 (m, 5H), 6.10-5.95 (dd, J = 3.3, 56.8 Hz, 1H), 4.03-3.91 (m, 1H), 3.81-3.75 (m, 1H), 3.24-3.11 (dddd, J = 1.2, 5.6, 13.2, 30.0 Hz, 1H); ^{13}C NMR (101 MHz, $CDCl_3$) δ 150.4, 135.7, 129.1, 129.0, 128.48, 128.46, 128.1, 127.7, 127.6, 125.27-125.16 (q, J = 3.8 Hz), 105.0 (d, J = 229.9 Hz), 43.7 (d, J = 4.9 Hz), 41.5 (d, J = 21.2 Hz); ^{19}F NMR (376 MHz, $CDCl_3$) δ -62.9 (s, 63F) -117.2 (s, 1F), -132.0 (s, 20F). HRMS (ESI-TOF) Calc'd for $C_{17}H_{13}F_4NO$ $[M+H]^+$: 324.1006; found 324.1001.

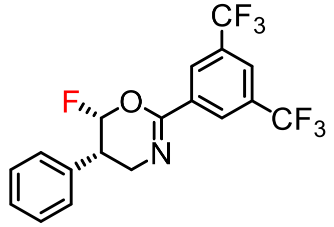

15b: Prepared according to general procedure A using **15a** (61.0 mg, 0.2 mmol). After work-up, the crude residue was purified by flash column chromatography on basic Al_2O_3 (EtOAc-Hexanes (0.5% Et_3N) elution: hexanes/EtOAc (V/V) = 50:1) to give **15b** (54.7 mg, 70%) as a white solid. R_f = 0.23 (PE/EA (V/V) = 25:1). **15b** was determined to be of 91% e.e. by HPLC (phenomenex Cellulose-1, *i*-PrOH/Hexanes (V/V) = 98:2, 1.0 mL/min; t_r (major) = 19.823 min, t_r (minor) = 23.810 min). **15b** was determined to be of 2:1 dr by ^{19}F NMR. ^{1}H NMR (400 MHz, $CDCl_3$) δ 8.39(s, 2H), 7.90 (s, 1H), 7.32-7.27 (m, 4H), 7.20-7.18 (d, J = 8.0 Hz, 1H), 6.13-5.99 (dd, J = 3.6, 56.8 Hz, 1H), 4.08-3.79 (m, 2H), 3.25-3.12 (dddd, J = 1.2, 5.6, 13.2, 30.0 Hz, 1H); ^{13}C NMR (101 MHz, $CDCl_3$) δ 150.0, 149.1, 136.4 (2C), 135.6, 134.4, 134.3, 131.8 (q, J = 34.0 Hz), 129.2, 129.0, 128.5 (2C), 128.2, 128.0, 127.5 (2C), 127.4, 124.5, 124.4, 121.8, 106.3 (d, J= 229.3 Hz), 105.0 (d, J = 229.3 Hz), 44.0 (d, J = 5.1 Hz),, 43.6 (d, J = 5.1 Hz), 41.6 (d, J = 21.1 Hz), 40.2 (d, J = 21.1 Hz); ^{19}F

NMR (376 MHz, $CDCl_3$) δ -62.9 (s, 18F), -117.0 (s, 1F), -132.2 (s, 2F). HRMS (ESI-TOF) Calc'd for $C_{17}H_{13}F_4NO$ $[M+H]^+$: 324.1006; found 324.1001.

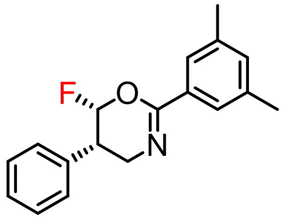

16b: Prepared according to general procedure A using **16a** (53.1 mg, 0.2 mmol). After work-up, the crude residue was purified by flash column chromatography on basic Al_2O_3 (EtOAc-Hexanes (0.5% Et_3N) elution: hexanes/EtOAc (V/V) = 50:1) to give **16b** (42.5 mg, 75%) as a white solid. R_f = 0.19 (PE/EA (V/V) = 25:1). **16b** was determined to be of 98% e.e. by HPLC (phenomenex Lux Amylose-1, *i*-PrOH/Hexanes (V/V) = 98:2, 1.0 mL/min; t_r (major) = 15.077 min, t_r (minor) = 18.847 min). **16b** was determined to be of > 20:1 dr by ^{19}F NMR. ^{1}H NMR (400 MHz, $CDCl_3$) δ 7.53 (s, 2H), 7.32-7.28 (m, 5H), 7.03 (s, 1H), 6.08-5.93 (dd, *J* = 3.2, 56.8 Hz, 1H), 4.01-3.89 (m, 1H), 3.78-3.71 (m, 1H), 3.21-3.09 (dddd, *J* = 1.2, 5.6, 13.2, 29.6 Hz, 1H); ^{13}C NMR (101 MHz, $CDCl_3$) δ 151.9, 137.9, 136.1, 132.7, 132.1, 128.9, 127.9, 105.0 (d, *J* = 229.0 Hz), 43.5 (d, *J* = 5.1 Hz), 41.5 (d, *J* = 21.4 Hz), 21.3; ^{19}F NMR (376 MHz, $CDCl_3$) δ -117.0 (s, 1F), -132.0 (s, 22F). HRMS (ESI-TOF) Calc'd for $C_{18}H_{18}FNO$ $[M+H]^+$: 284.1445; found 284.1448.

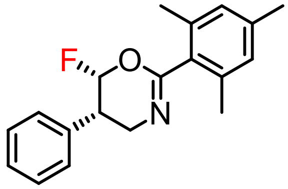

17b: Prepared according to general procedure A using **17a** (55.9 mg, 0.2 mmol). After work-up, the crude residue was purified by flash column chromatography on basic Al_2O_3 (EtOAc-Hexanes (0.5% Et_3N) elution: hexanes/EtOAc (V/V) = 50:1) to give **17b** (35.6 mg, 60%) as a white solid. R_f = 0.14 (PE/EA (V/V) = 25:1). **17b** was determined to be of 85% e.e. by HPLC (phenomenex Lux Amylose-1, *i*-PrOH/Hexanes (V/V) = 98:2, 1.0 mL/min; t_r (major) = 12.613 min, t_r (minor) = 14.160 min). **17b** was determined to be of 5:1 dr by ^{19}F NMR. ^{1}H NMR (500 MHz, $CDCl_3$) δ 7.32-7.26 (m, 5H), 6.79 (s, 2H), 6.74, 6.04-5.85 (dd, *J* = 3.0, 56.8 Hz, 1H), 4.09-3.95 (m, 1H), 3.92-3.73 (m, 1H), 3.23-3.14 (m, 1H), 2.27 (s, 6H), 2.20 (s, 3H); ^{13}C NMR (126 MHz, $CDCl_3$) δ 158.0, 153.1, 138.8, 137.8, 136.2, 136.1, 135.8, 135.4, 131.4, 130.8, 129.3, 128.9 (2C), 128.5, 128.4, 128.3, 128.2, 127.8, 127.5, 106.5 (d, *J* = 225.9 Hz), 105.0 (d, *J* = 225.9 Hz), 43.6 (d, *J* = 4.5 Hz), 41.8 (d, *J* = 21.4 Hz), 41.8 (d, *J* = 21.4 Hz), 39.7 (d, *J* = 21.4 Hz), 21.2, 21.1, 19.6, 19.2; ^{19}F NMR (471 MHz, $CDCl_3$) δ -116.8 (s, 1F), -132.3 (s, 5F). HRMS (ESI-TOF) Calc'd for $C_{19}H_{20}FNO$ $[M+H]^+$: 298.1602; found 298.1605.

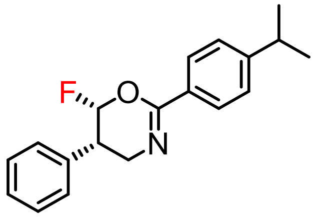

18b: Prepared according to general procedure A using **18a** (55.9 mg, 0.2 mmol). After work-up, the crude residue was purified by flash column chromatography on basic Al_2O_3 (EtOAc-Hexanes (0.5% Et_3N) elution: hexanes/EtOAc (V/V) = 10:1) to give **18b** (41.6 mg, 70%) as a white solid. R_f = 0.18 (PE/EA (V/V) = 10:1). **18b** was determined to be of 99% e.e. by HPLC (phenomenex Lux Amylose-1, *i*-PrOH/Hexanes (V/V) = 98:2, 1.0 mL/min; t_r (major) = 25.867 min, t_r (minor) = 35.870 min). **18b** was determined to be of > 20:1 dr by ^{19}F NMR. ^{1}H NMR (400 MHz, $CDCl_3$) δ 7.84-7.82 (d, *J* = 8.4 Hz, 2H), 7.33-7.27 (m, 5H), 7.20-7.18 (d, *J* = 8.4 Hz, 2H), 6.08-5.93 (dd, *J* = 3.3, 56.8 Hz, 1H), 4.01-3.88 (m, 1H), 3.76-3.71 (m, 1H), 3.21-3.08 (dddd, *J* = 1.2, 5.6, 13.2, 30.0 Hz, 1H), 2.92-2.81 (m, 1H), 1.20-1.18 (d, *J* = 6.8 Hz, 6H); ^{13}C NMR (101 MHz, $CDCl_3$) δ 152.2, 136.2, 129.9, 128.9, 128.5, 127.9, 127.4, 127.2, 126.4, 105.0 (d, *J* = 228.8 Hz), 43.5 (d, *J* = 4.9 Hz), 41.7 (d, *J* = 21.2 Hz), 34.1, 23.8; ^{19}F NMR (376 MHz, $CDCl_3$) δ -116.9 (s, 1F), -132.0 (s, 20F). HRMS (ESI-TOF) Calc'd for $C_{19}H_{20}FNO$ $[M+H]^+$: 298.1602; found 298.1602.

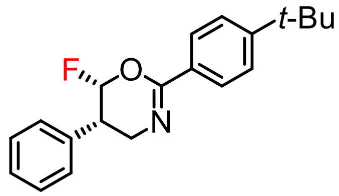

19b: Prepared according to general procedure A using **19a** (58.7 mg, 0.2 mmol). After work-up, the crude residue was purified by flash column chromatography on basic Al_2O_3 (EtOAc-Hexanes (0.5% Et_3N) elution: hexanes/EtOAc (V/V) = 10:1) to give **19b** (41.7 mg, 67%) as a white solid. R_f = 0.15 (PE/EA (V/V) = 10:1). **19b** was determined to be of 88% e.e. by HPLC (phenomenex Cellulose-1, *i*-PrOH/Hexanes (V/V) = 98:2, 1.0 mL/min; t_r (major) = 13.933 min, t_r (minor) = 10.827 min). **19b** was determined to be of 10:1 dr by ^{19}F NMR. ^{1}H NMR (500 MHz, $CDCl_3$) δ 7.84-7.83 (d, *J* = 8.5 Hz, 2H), 7.36-7.26 (m, 7H), 6.07-5.93 (dd, *J* = 3.0, 56.8 Hz, 1H), 3.99-3.89 (m, 1H), 3.82-3.73 (m, 1H), 3.20-3.10 (m, 1H), 1.26 (s, 9H); ^{13}C NMR (126 MHz, $CDCl_3$) δ 154.4, 129.5, 128.9, 128.6, 128.5, 127.9, 127.2, 127.1, 125.2, 105.0 (d, *J* = 228.3 Hz), 43.5 (d, *J* = 5.3 Hz), 41.7 (d, *J* = 21.3 Hz), 34.9, 31.2; ^{19}F NMR (471 MHz, $CDCl_3$) δ -116.9 (s, 1F), -132.1 (s, 10F). HRMS (ESI-TOF) Calc'd for $C_{20}H_{22}FNO$ $[M+H]^+$: 312.1758; found 392.1759.

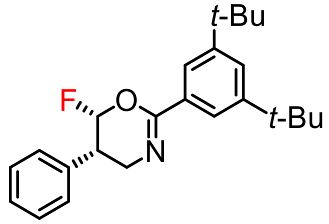

20b: Prepared according to general procedure A using **20a** (69.9 mg, 0.2 mmol). After work-up, the crude residue was purified by flash column chromatography on basic Al_2O_3 (EtOAc-Hexanes (0.5% Et_3N) elution: hexanes/EtOAc (V/V) = 25:1) to give **20b** (47.7 mg, 65%) as a white solid. R_f = 0.1 (PE/EA (V/V) = 25:1). **20b** was determined to be of >99% e.e. by HPLC (phenomenex Cellulose-1, *i*-PrOH/Hexanes (V/V) = 99:1, 1.0 mL/min; t_r (major) = 10.547min). **20b** was determined to be of 14:1 dr by ^{19}F NMR. ^{1}H NMR (400 MHz, $CDCl_3$) δ 7.87 (s, 2H), 7.59 (s, 1H), 7.45-7.34 (m, 5H), 6.22-6.07 (dd, *J* = 3.2, 56.8 Hz, 1H), 4.13-4.01 (m, 1H), 3.91-3.85 (m, 1H), 3.33-3.20 (dddd, *J* = 1.2, 5.6, 13.2, 29.6 Hz, 1H), 1.40 (s, 18H); ^{13}C NMR (101 MHz, $CDCl_3$) δ 150.8, 136.3, 131.6, 128.9, 128.5, 127.9, 127.1, 125.3, 121.5, 105.0 (d, *J* = 228.7 Hz), 43.5 (d, *J* = 5.2 Hz), 41.8 (d, *J* = 21.4 Hz), 35.0, 31.5; ^{19}F NMR (376 MHz, $CDCl_3$) δ -116.9 (s, 1F), -132.0 (s, 14F). HRMS (ESI-TOF) Calc'd for $C_{24}H_{30}FNO$ $[M+H]^+$: 368.2384; found 368.2383.

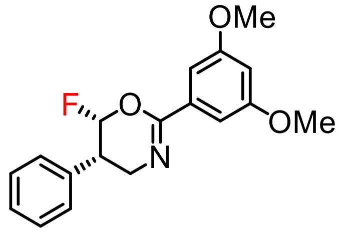

21b: Prepared according to general procedure A using **21a** (53.3 mg, 0.2 mmol). After work-up, the crude residue was purified by flash column chromatography on basic Al_2O_3 (EtOAc-Hexanes (0.5% Et_3N) elution: hexanes/EtOAc (V/V) = 10:1) to give **21b** (42.8 mg, 68%) as a white solid. R_f = 0.1 (PE/EA (V/V) = 25:1). **21b** was determined to be of 98% e.e. by HPLC (IC, *i*-PrOH/Hexanes (V/V) = 92:8, 1.0 mL/min; t_r (major) = 18.027 min, t_r (minor) = 13.534 min). **21b** was determined to be of > 20:1 dr by ^{19}F NMR. ^{1}H NMR (500 MHz, $CDCl_3$) δ 7.41-7.34 (m, 5H), 7.15 (s, 1H), 7.08 (s, 1H), 6.58 (s, 1H), 6.15-6.03 (dd, *J* = 3.2, 56.8 Hz, 1H), 4.08-3.97 (m, 1H), 3.83-3.73 (m, 7H), 3.23-3.11 (dddd, *J* = 1.2, 5.6, 13.2, 29.6 Hz, 1H); ^{13}C NMR (126 MHz, $CDCl_3$) δ 160.6, 151.3, 136.0, 134.3, 128.9, 128.5, 128.0, 105.0 (d, *J* = 228.8 Hz), 105.0, 104.1, 55.6, 43.5 (d, *J* = 5.0 Hz), 41.6 (d, *J* = 21.3 Hz); ^{19}F NMR (471 MHz, $CDCl_3$) δ -117.1 (s, 1F), -132.1 (s, 26F). HRMS (ESI-TOF) Calc'd for $C_{18}H_{18}FNO_3$ $[M+H]^+$: 316.1343; found 316.1340.

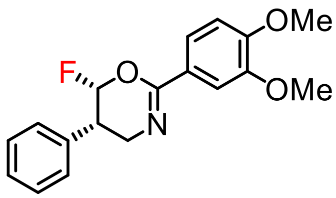

22b: Prepared according to general procedure A using **22a** (53.3 mg, 0.2 mmol). After work-up, the crude residue was purified by flash column chromatography on basic Al_2O_3 (EtOAc-Hexanes (0.5% Et_3N) elution: hexanes/EtOAc (V/V) = 10:1) to give **22b** (40.3 mg, 64%) as a white solid. R_f = 0.1 (PE/EA (V/V) = 25:1). **22b** was determined to be of 97% e.e. by HPLC (phenomenex Cellulose-1, *i*-PrOH/Hexanes (V/V) = 85:15, 0.8 mL/min; t_r (major) = 36.347 min, t_r (minor) = 23.480 min). **22b** was determined to be of > 20:1 dr by ^{19}F NMR. ^{1}H NMR (400 MHz, $CDCl_3$) δ 7.47-7.45 (d, *J* = 8.0 Hz, 1H), 7.35-7.33 (m, 5H), 7.19 (s, 1H), 6.82-6.80 (d, *J* = 8.0 Hz, 1H), 6.82-6.80 (d, *J* = 8.0 Hz, 1H), 6.10-5.95 (dd, *J* = 3.3, 56.8 Hz, 1H), 4.02-3.93 (m, 1H), 3.8(s, 3H), 3.86 (s, 3H), 3.78-3.72 (m, 1H), 3.23-3.11 (dddd, *J* = 1.2, 5.6, 13.2, 29.6 Hz, 1H); ^{13}C NMR (126 MHz, $CDCl_3$) δ 151.5, 148.7, 136.2, 129.0, 128.9, 128.6, 128.5, 127.9, 127.2, 125.0, 120.6, 120.3, 110.0, 105.0 (d, *J* = 228.4 Hz), 55.8, 43.5 (d, *J* = 5.0 Hz), 41.8 (d, *J* = 21.4 Hz); ^{19}F NMR (376 MHz, $CDCl_3$) δ -117.1 (s, 1F), -132.1 (s, 20F). HRMS (ESI-TOF) Calc'd for $C_{18}H_{18}FNO_3$ $[M+H]^+$: 316.1343; found 316.1340.

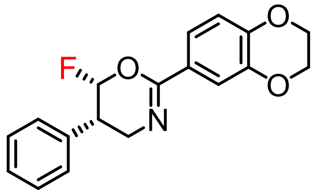

23b: Prepared according to general procedure A using **23a** (59.0 mg, 0.2 mmol). After work-up, the crude residue was purified by flash column chromatography on basic Al_2O_3 (EtOAc-Hexanes (0.5% Et_3N) elution: hexanes/EtOAc (V/V) = 10:1) to give **23b** (42.5 mg, 68%) as a white solid. R_f = 0.11 (PE/EA (V/V) = 10:1). **23b** was determined to be of 93% e.e. by HPLC (AD, *i*-PrOH/Hexanes (V/V) = 97:3, 1.0 mL/min; t_r (major) = 47.450 min, t_r (minor) = 62.867 min). **23b** was determined to be of > 20:1 dr by ^{19}F NMR. ^{1}H NMR (500 MHz, $CDCl_3$) δ 7.51-7.48 (m, 2H), 7.40-7.34 (m, 5H), 6.88-6.87 (d, *J* = 8.0 Hz, 1H), 6.12-6.00 (dd, *J* = 3.5, 56.8 Hz, 1H), 4.28-4.26 (t, *J* = 6.5 Hz, 4H), 4.05-3.94 (m, 1H), 3.82-3.76 (m, 1H), 3.78-3.72 (m, 1H), 3.26-3.17 (dddd, *J* = 1.2, 5.6, 13.2, 29.6 Hz, 1H); ^{13}C NMR (126 MHz, $CDCl_3$) δ 151.0, 146.1, 143.2, 136.2, 128.9, 128.5, 127.9, 127.2, 125.7, 120.9, 117.0, 116.7, 104.9 (d, *J* = 228.4 Hz), 64.6, 64.2, 43.4 (d, *J* = 5.0 Hz), 41.8 (d, *J* = 20.7 Hz); ^{19}F NMR (471 MHz, $CDCl_3$) δ -117.1 (s, 1F), -132.1 (s, 29F). HRMS (ESI-TOF) Calc'd for $C_{18}H_{16}FNO_3$ $[M+H]^+$: 314.1187; found 314.1189.

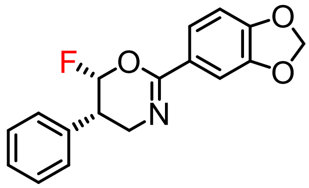

24b: Prepared according to general procedure A using **24a** (56.2 mg, 0.2 mmol). After work-up, the crude residue was purified by flash column chromatography on basic Al_2O_3 (EtOAc-Hexanes (0.5% Et_3N) elution: hexanes/EtOAc (V/V) = 15:1) to give **24b** (36.5 mg, 61%) as a white solid. R_f = 0.22 (PE/EA (V/V) = 10:1). **24b** was determined to be of 94% e.e. by HPLC (phenomenex Lux Amylose-1, *i*-PrOH/Hexanes (V/V) = 98:2, 1.0 mL/min; t_r (major) = 48.877 min, t_r (minor) = 59.397 min). **24b** was determined to be of 15:1 dr by ^{19}F NMR. ^{1}H NMR (500 MHz, $CDCl_3$) δ 7.55-7.53 (d, *J* = 8.0 Hz, 2H), 7.55 (s, 1H), 7.41-7.36 (m, 5H), 6.83-6.81(d, *J* = 8.0 Hz, 1H), 6.12-5.98 (m, 3H), 4.06-3.94 (m, 1H), 3.86-3.78 (m, 1H), 3.26-3.17 (m, 1H); ^{13}C NMR (126 MHz, $CDCl_3$) δ 150.9, 150.0, 147.7, 136.1, 129.0, 128.9, 128.6, 128.5, 127.9, 127.2, 126.5, 122.1, 107.8, 107.7, 105.0 (d, *J* = 228.6 Hz), 101.5, 55.8, 43.4 (d, *J* = 5.0 Hz), 41.8 (d, *J* = 21.0 Hz); ^{19}F NMR (471 MHz, $CDCl_3$) δ -117.2 (s, 1F), -132.1 (s, 15F). HRMS (ESI-TOF) Calc'd for $C_{17}H_{14}FNO_3$ $[M+H]^+$: 300.1030; found 300.1029.

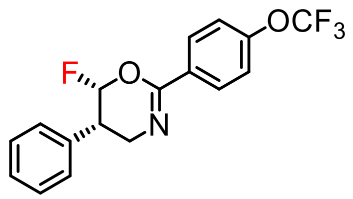

25b: Prepared according to general procedure A using **25a** (64.3 mg, 0.2 mmol). After work-up, the crude residue was purified by flash column chromatography on basic Al_2O_3 (EtOAc-Hexanes (0.5% Et_3N) elution: hexanes/EtOAc (V/V) = 50:1) to give **25b** (44.1 mg, 65%) as a white solid. R_f = 0.26 (PE/EA (V/V) = 25:1). **25b** was determined to be of 91% e.e. by HPLC (phenomenex Lux Amylose-1, *i*-PrOH/Hexanes (V/V) = 98:2, 1.0 mL/min; t_r (major) = 10.273 min, t_r (minor) = 10.910 min). **25b** was determined to be of 13:1 dr by ^{19}F NMR. ^{1}H NMR (400 MHz, $CDCl_3$) δ 7.96−7.94 (d, *J* = 8.8 Hz, 2H), 7.32-7.27 (m, 5H), 7.18-7.16 (d, *J* = 8.0 Hz, 2H), 6.09-5.94 (dd, *J* = 4.0, 56.4 Hz, 1H), 4.02-3.89 (m, 1H), 3.79-3.72 (m, 1H), 3.22-3.10 (dddd, *J* = 1.2, 6.0, 13.2, 30.0 Hz, 1H); ^{13}C NMR (101 MHz, $CDCl_3$) δ 151.3, 150.47, 150.42, 150.39, 150.34 (q, *J* = 2.6 Hz), 135.9, 130.8, 129.1, 128.9, 128.5, 128.0, 127.2, 120.4, 106.1, 103.8 (d, *J* = 229.7 Hz), 43.5 (d, *J* = 4.6 Hz), 41.7, 41.5 (d, *J* = 21.3 Hz); ^{19}F NMR (376 MHz, $CDCl_3$) δ -57.7 (s, 45F) -117.1 (s, 1F), -132.1 (s, 14F). HRMS (ESI-TOF) Calc'd for $C_{17}H_{13}F_4NO_2$ $[M+H]^+$: 340.0955; found 340.0952.

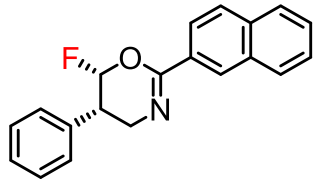

26b: Prepared according to general procedure A using **26a** (57.5 mg, 0.2 mmol). After work-up, the crude residue was purified by flash column chromatography on basic Al_2O_3 (EtOAc-Hexanes (0.5% Et_3N) elution: hexanes/EtOAc (V/V) = 25:1) to give **26b** (47.0 mg, 77%) as a white solid. R_f = 0.11 (PE/EA (V/V) = 25:1). **26b** was determined to be of 87% e.e. by HPLC (IC, *i*-PrOH/Hexanes (V/V) = 98:2, 1.0 mL/min; t_r (major) = 15.657 min, t_r (minor) = 14.203 min). **26b** was determined to be of > 20:1 dr by ^{19}F NMR. ^{1}H NMR (400 MHz, $CDCl_3$) δ 8.48 (s, 1H), 8.10-8.07 (d, *J* = 8.8 Hz, 1H), 7.93-7.91 (d, *J* = 8.8 Hz, 1H), 7.88-7.86 (d, *J* = 8.8 Hz, 2H), 7.54-7.49 (m, 2H), 7.41-7.35 (m, 5H), 6.24-6.09 (dd, *J* = 3.6, 56.8 Hz, 1H), 4.11-4.03 (m, 1H), 3.93-3.86 (m, 1H), 3.36-3.24 (dddd, *J* = 1.2, 5.6, 13.2, 29.6 Hz, 1H); ^{13}C NMR (126 MHz, $CDCl_3$) δ 151.6, 136.1, 134.7, 132.8, 129.6, 129.0, 128.9, 128.5 (2C), 128.0, 127.7, 127.6, 127.4, 126.4, 124.1, 105.1 (d, *J* = 228.7 Hz), 43.6 (d, *J* = 5.2 Hz), 41.9-41.7 (d, *J* = 21.4 Hz); ^{19}F NMR (471 MHz, $CDCl_3$) δ -117.1 (s, 1F), -131.9 (s, 30F). HRMS (ESI-TOF) Calc'd for $C_{20}H_{16}FNO$ $[M+H]^+$: 306.1289; found 306.1281.

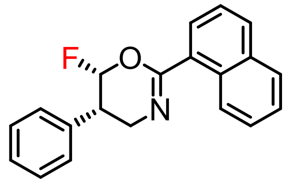

27b: Prepared according to general procedure A using **27a** (57.5 mg, 0.2 mmol). After work-up, the crude residue was purified by flash column chromatography on basic Al_2O_3 (EtOAc-Hexanes (0.5% Et_3N) elution: hexanes/EtOAc (V/V) = 25:1) to give **27b** (45.1 mg, 74%) as a white solid. R_f = 0.11 (PE/EA (V/V) = 25:1). **27b** was determined to be of 86% e.e. by HPLC (phenomenex Cellulose-1, *i*-PrOH/Hexanes (V/V) = 95:5, 1.0 mL/min; 1.0 mL/min; t_r (major) = 30.907 min, t_r (minor) = 28.800 min). **27b** was determined to be of > 20:1 dr by ^{19}F NMR. ^{1}H NMR (400 MHz, $CDCl_3$) δ 8.72-8.70 (d, *J* = 8.2 Hz, 1H), 7.98-7.90 (m, 3H), 7.64-7.60 (t, *J* = 8.4 Hz, 1H), 7.57-7.52 (m, 2H), 7.46-7.39 (m, 5H), 6.24-6.09 (dd, *J* = 3.6, 56.8 Hz, 1H), 4.22-4.14 (m, 1H), 4.03-3.98 (m, 1H), 3.48-3.36 (dddd, *J* = 1.2, 5.6, 13.2, 29.6 Hz, 1H); ^{13}C NMR (126 MHz, $CDCl_3$) δ 152.9, 136.1, 133.8, 131.1, 130.8, 130.2, 128.9, 128.5, 128.4, 128.0, 127.7, 127.0, 126.0, 125.7, 124.8, 105.2 (d, *J* = 228.8 Hz), 43.9 (d, *J* = 5.0 Hz), 41.6 (d, *J* = 21.3 Hz); ^{19}F NMR (376 MHz, $CDCl_3$) δ -116.3 (s, 1F), -132.1 (s, 27F). HRMS (ESI-TOF) Calc'd for $C_{20}H_{16}FNO$ $[M+H]^+$: 306.1289; found 306.1282.

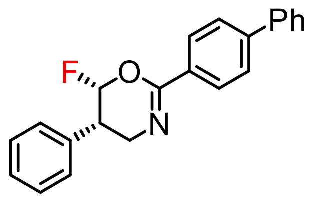

28b: Prepared according to general procedure A using **28a** (62.6 mg, 0.2 mmol). After work-up, the crude residue was purified by flash column chromatography on basic Al_2O_3 (EtOAc-Hexanes (0.5% Et_3N) elution: hexanes/EtOAc (V/V) = 20:1) to give **28b** (45.7 mg, 69%) as a white solid. R_f = 0.29 (PE/EA (V/V) = 10:1). **28b** was determined to be of 81% e.e. by HPLC (phenomenex Lux Amylose-1, *i*-PrOH/Hexanes (V/V) = 90:10, 1.0 mL/min; t_r (major) = 22.063 min, t_r (minor) = 28.510 min). **28b** was determined to be of 11:1 dr by ^{19}F NMR. ^{1}H NMR (400 MHz, $CDCl_3$) δ 8.10-8.08 (d, *J* = 8.4 Hz, 2H), 7.69-7.66 (m, 4H), 7.51-7.45 (m, 3H), 7.42-7.36 (m, 5H), 6.22-6.07 (dd, *J* = 3.6, 56.4 Hz, 1H), 4.16-4.03 (m, 1H), 3.92-3.86 (m, 1H), 3.36-3.23 (dddd, *J* = 1.2, 6.0, 13.2, 30.0 Hz, 1H); ^{13}C NMR (101 MHz, $CDCl_3$) δ 151.4, 143.8, 140.3, 136.1, 131.1, 128.9 (2C), 128.5, 128.0, 127.8 (2C), 127.2, 127.0, 105.0 (d, *J* = 229.3 Hz), 43.5 (d, *J* = 5.1 Hz), 41.7 (d, *J* = 21.1 Hz); ^{19}F NMR (376 MHz, $CDCl_3$) δ -117.2 (s, 1F), -132.1 (s, 11F). HRMS (ESI-TOF) Calc'd for $C_{22}H_{18}FNO$ $[M+H]^+$: 332.1445; found 332.1446.

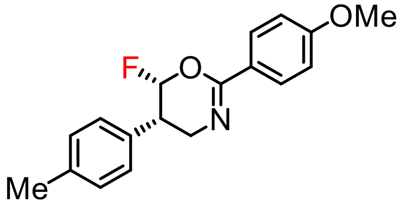

29b: Prepared according to general procedure A using **29a** (56.2 mg, 0.2 mmol). After work-up, the crude residue was purified by flash column chromatography on basic Al_2O_3 (EtOAc-Hexanes (0.5% Et_3N) elution: hexanes/EtOAc (V/V) = 15:1) to give **29b** (42.5 mg, 71%) as a white solid. R_f = 0.18 (PE/EA (V/V) = 10:1). **29b** was determined to be of 94% e.e. by HPLC (IC, *i*-PrOH/Hexanes (V/V) = 95:5, 1.0 mL/min; t_r (major) = 16.187 min, t_r (minor) = 18.817 min). **29b** was determined to be of 2:1 dr by ^{19}F NMR. ^{1}H NMR (500 MHz, $CDCl_3$) δ 7.85−7.84 (d, *J* = 8.5 Hz, 2H), 7.10-7.06 (m, 4H), 6.83-6.82 (d, *J* = 8.5 Hz, 2H), 6.02-5.90 (dd, *J* = 4.0, 56.4 Hz, 1H), 3.99-3.93 (m, 1H), 3.78-3.74 (m, 4H), 3.16-3.12 (m, 1H), 2.24 (s, 3H); ^{13}C NMR (101 MHz, $CDCl_3$) δ 162.0, 137.4, 134.0, 133.9, 129.7, 129.0, 124.7, 113.5, 106.7 (d, *J* = 225.8 Hz), 55.37, 43.3 (d, *J* = 4.6 Hz), 40.3 (d, *J* = 21.3 Hz), 21.0; ^{19}F NMR (376 MHz, $CDCl_3$) δ -117.4 (s, 2F), -132.2 (s, 1F). HRMS (ESI-TOF) Calc'd for $C_{18}H_{18}FNO_2$ $[M+H]^+$: 300.1394; found 300.1398.

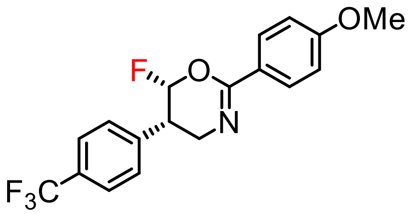

30b: Prepared according to general procedure A using **30a** (67.1 mg, 0.2 mmol). After work-up, the crude residue was purified by flash column chromatography on basic Al_2O_3 (EtOAc-Hexanes (0.5% Et_3N) elution: hexanes/EtOAc (V/V) = 15:1) to give **30b** (52.9 mg, 75%) as a white solid. R_f = 0.14 (PE/EA (V/V) = 10:1). **30b** was determined to be of > 20:1 dr by ^{19}F NMR. ^{1}H NMR (400 MHz, $CDCl_3$) δ 7.90−7.88 (d, *J* = 8.8 Hz, 2H), 7.59-7.57 (d, *J* = 8.0 Hz, 2H), 7.43-7.41 (d, *J* = 8.0 Hz, 2H), 6.87-6.85 (d, *J* = 8.0 Hz, 2H), 6.11-5.95 (dd, *J* = 4.0 , 56.4 Hz, 1H), 3.96-3.89 (m, 1H), 3.84-3.75 (m, 4H), 3.31-3.19 (m, 1H); ^{13}C NMR (101 MHz, $CDCl_3$) δ 162.1, 130.4, 129.1, 128.9, 128.1, 125.8 (q, *J* = 3.5 Hz), 125.1, 122.9, 113.6 104.4 (d, *J* = 228.4 Hz), 55.4, 43.2 (d, *J* = 4.6 Hz), 41.6 (d, *J* = 21.3 Hz); ^{19}F NMR (376 MHz, $CDCl_3$) δ -62.7 (s, 69F), -131.8 (s, 22F), -134.9 (s, 1F). HRMS (ESI-TOF) Calc'd for $C_{18}H_{15}F_4NO_2$ $[M+H]^+$: 354.1112; found 354.1109.

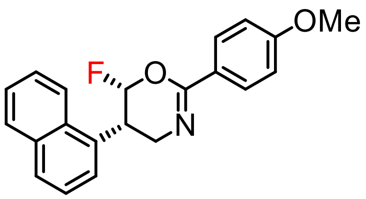

31b: Prepared according to general procedure A using **31a** (63.4 mg, 0.2 mmol). After work-up, the crude residue was purified by flash column chromatography on basic Al_2O_3 (EtOAc-Hexanes (0.5% Et_3N) elution: hexanes/EtOAc (V/V) = 50:1) to give **31b** (37.5 mg, 56%) as a white solid. R_f = 0.16 (PE/EA (V/V) = 10:1). **31b** was determined to be of 4:1 dr by ^{19}F NMR. ^{1}H NMR (400 MHz, $CDCl_3$) δ 7.97−7.95 (d, *J* = 8.4 Hz, 1H), 7.89-7.86 (d, *J* = 8.8 Hz, 2H), 7.84-7.82 (d, *J* = 8.0 Hz, 1H), 7.74-7.71 (d, J = 8.0 Hz, 1H), 7.55-7.52 (t, *J* = 8.0 Hz, 1H), 7.48-7.44(t, *J* = 8.0 Hz, 1H), 7.43-7.42 (q, *J* = 8.0 Hz, 1H), 7.35-7.31 (t, *J* = 8.0 Hz, 1H), 6.85-6.83 (d, *J* = 8.0 Hz, 2H), 6.28-5.13 (dd, *J* = 4.0, 56.4 Hz, 1H), 4.23-4.16 (m, 1H), 4.07-3.98 (m, 1H), 3.77 (s, 3H); ^{3}C NMR (101 MHz, $CDCl_3$) δ 153.5, 128.8, 126.1, 124.2, 123.9, 123.3, 121.7, 120.7, 120.5, 118.9, 117.1, 108.4, 100.5 (d, *J* = 226.5 Hz), 100.0, 38.4 (d, *J* = 4.6 Hz), 30.2 (d, *J* = 21.3 Hz); ^{19}F NMR (376 MHz, $CDCl_3$) δ -115.1 (s, 4F), -130.5 (s, 1F). HRMS (ESI-TOF) Calc'd for $C_{21}H_{18}FNO_2$ $[M+H]^+$: 336.1394; found 336.1398.

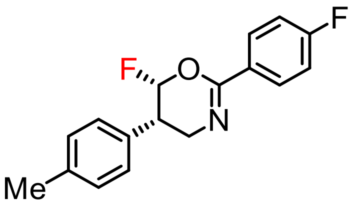

32b: Prepared according to general procedure A using **32a** (65.8 mg, 0.2 mmol). After work-up, the crude residue was purified by flash column chromatography on basic Al_2O_3 (EtOAc-Hexanes (0.5% Et_3N) elution: hexanes/EtOAc (V/V) = 50:1) to give **32b** (39.1 mg, 68%) as a white solid. R_f = 0.37 (PE/EA (V/V) = 10:1). **32b** was determined to be of 80% e.e. by HPLC (phenomenex Lux Amylose-1, *i*-PrOH/Hexanes (V/V) = 98:2, 1.0 mL/min; t_r (major) = 9.447 min, t_r (minor) = 10.850 min). **32b** was determined to be of > 20:1 dr by ^{19}F NMR. ^{1}H NMR (500 MHz, $CDCl_3$) δ 7.97-7.95 (dd, *J* = 8.8, 5.5 Hz, 2H), 7.17-7.14 (m, 4H), 7.08-7.05 (t, *J* = 8.5 Hz, 2H), 6.10-5.98 (dd, *J* = 56.5, 3.5 Hz, 1H), 4.07-4.01 (dt, *J* = 17.0, 5.5 Hz, 1H), 3.87-3.83 (m, 1H), 3.24-3.20 (m, 1H), 2.32 (s, 3H); ^{13}C NMR (126 MHz, $CDCl_3$) δ 164.6 (d, *J* = 250.9 Hz), 151.8, 137.5, 129.7, 129.5, 129.4, 127.5, 115.3, 115.1, 106.6 (d, *J* = 226.7 Hz), 44.3 (d, *J* = 2.4 Hz), 41.5 (d, *J* = 21.5 Hz), 21.0; ^{19}F NMR (471 MHz, $CDCl_3$) δ -109.3 (s, 43F), -117.4 (s, 42F), -132.3 (s, 1F). HRMS (ESI-TOF) Calc'd for $C_{17}H_{15}F_2NO$ $[M+H]^+$: 288.1194; found 288.1196.

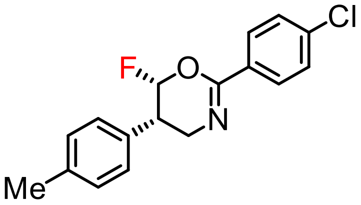

33b: Prepared according to general procedure A using **33a** (65.8 mg, 0.2 mmol). After work-up, the crude residue was purified by flash column chromatography on basic Al_2O_3 (EtOAc-Hexanes (0.5% Et_3N) elution: hexanes/EtOAc (V/V) = 50:1) to give **33b** (40.0 mg, 66%) as a white solid. R_f = 0.38 (PE/EA (V/V) = 10:1). **33b** was determined to be of 90% e.e. by HPLC (phenomenex Lux Amylose-1, *i*-PrOH/Hexanes (V/V) = 98:2, 1.0 mL/min; t_r (major) = 9.413 min, t_r (minor) = 12.217 min). **30b** was determined to be of > 20:1 dr by ^{19}F NMR. ^{1}H NMR (500 MHz, $CDCl_3$) δ 7.90-7.89 (d, *J* = 8.6 Hz, 2H), 7.37-7.35 (d, *J* = 8.5 Hz, 2H), 7.17-7.13 (m, 4H), , 6.10-5.98 (dd, *J* = 3.5, 56.5 Hz, 1H), 4.07-4.02 (dt, *J* = 17.0, 5.5 Hz, 1H), 3.88-3.84 (m, 1H), 3.25-3.21 (m, 1H), 2.32 (s, 3H); ^{13}C NMR (101 MHz, $CDCl_3$) δ 151.8, 137.6, 137.2, 133.8, 130.8, 129.8, 128.6, 128.5, 127.5, 106.6 (d, *J* = 229.7 Hz), 44.3 (d, *J* = 4.6 Hz), 40.1 (d, *J* = 21.3 Hz), 21.0; ^{19}F NMR (471 MHz, $CDCl_3$) δ -117.1 (s, 60F), -132.2 (s, 14F). HRMS (ESI-TOF) Calc'd for $C_{17}H_{15}FClNO$ $[M+H]^+$: 304.0899; found 304.0898.

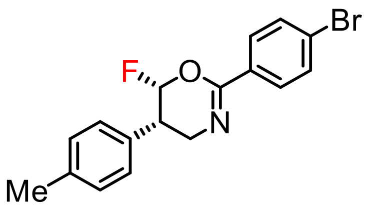

34b: Prepared according to general procedure A using **34a** (65.8 mg, 0.2 mmol). After work-up, the crude residue was purified by flash column chromatography on basic Al_2O_3 (EtOAc-Hexanes (0.5% Et_3N) elution: hexanes/EtOAc (V/V) = 50:1) to give **34b** (41.8 mg, 60%) as a white solid. R_f = 0.38 (PE/EA (V/V) = 10:1). **34b** was determined to be of 81% e.e. by HPLC (phenomenex Lux Amylose-1, *i*-PrOH/Hexanes (V/V) = 98:2, 1.0 mL/min; t_r (major) = 10.880 min, t_r (minor) = 12.200 min). **34b** was determined to be of 10:1 dr by ^{19}F NMR. ^{1}H NMR (500 MHz, $CDCl_3$) δ 7.76-7.74 (d, *J* = 8.5 Hz, 2H), 7.45-7.43 (d, J = 8.5 Hz, 2H), 7.09-7.04 (m, 4H), 6.09-5.94 (dd, *J* = 4.0, 56.0 Hz, 1H), 3.99-3.93 (dt, *J* = 17.0, 5.5 Hz, 1H), 3.79-3.75 (m, 1H), 3.17-3.13 (m, 1H), 2.24 (s, 3H); ^{13}C NMR (101 MHz, $CDCl_3$) δ 151.9, 137.6, 133.8, 131.4, 131.2, 129.8, 128.9, 127.5, 125.7, 106.6 (d, *J* = 226.6 Hz), 44.4 (d, *J* = 4.6 Hz), 40.1 (d, *J* = 21.3 Hz), 21.1; ^{19}F NMR (471 MHz, $CDCl_3$) δ -117.4 (s, 60F), -132.2 (s, 1F). HRMS (ESI-TOF) Calc'd for $C_{17}H_{15}FBrNO$ $[M+H]^+$: 348.0394; found 348.0390.

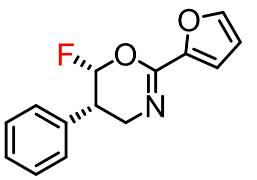

35b: Prepared according to general procedure A using **35a** (45.4 mg, 0.2 mmol). After work-up, the crude residue was purified by flash column chromatography on basic Al_2O_3 (EtOAc-Hexanes (0.5% Et_3N) elution: hexanes/EtOAc (V/V) = 20:1) to give **35b** (35.3 mg, 72%) as a white solid. R_f = 0.15 (PE/EA (V/V) = 10:1). **35b** was determined to be of 88% e.e. by HPLC (phenomenex Lux Amylose-1, *i*-PrOH/Hexanes (V/V) = 98:2, 1.0 mL/min; t_r (major) = 25.273 min, t_r (minor) = 23.630 min). **35b** was determined to be of 10:1 dr by ^{19}F NMR. ^{1}H NMR (400 MHz, $CDCl_3$) δ 46-7.44 (m, 1H), 7.31-7.26 (m, 5H), 6.86-6.85 (d, *J* = 3.4 Hz, 1H), 6.42-6.40 (m, 1H), 6.03-5.89 (dd, *J* = 3.2, 56.4 Hz, 1H), 3.99-3.88 (m, 1H), 3.80-3.73 (m, 1H), 3.24-3.11 (dddd, *J* = 1.2, 6.0, 13.2, 30.0 Hz, 1H); ^{13}C NMR (101 MHz, $CDCl_3$) δ 146.0, 145.0, 135.8, 128.9, 128.5, 128.0, 127.7, 113.1, 111.4, 104.8 (d, *J* = 230.6 Hz), 43.1 (d, *J* = 4.9 Hz), 41.7, 41.5 (d, *J* = 21.3 Hz); ^{19}F NMR (376 MHz, $CDCl_3$) δ -117.6 (s, 1F), -132.7 (s, 10F). HRMS (ESI-TOF) Calc'd for $C_{14}H_{12}FNO_2$ $[M+H]^+$: 246.0925; found 246.0927.

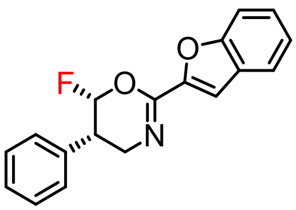

36b: Prepared according to general procedure A using **36a** (55.4 mg, 0.2 mmol). After work-up, the crude residue was purified by flash column chromatography on basic Al_2O_3 (EtOAc-Hexanes (0.5% Et_3N) elution: hexanes/EtOAc (V/V) = 20:1) to give **36b** (41.3 mg, 70%) as a white solid. R_f = 0.24 (PE/EA (V/V) = 10:1). **36b** was determined to be of 92% e.e. by HPLC (phenomenex Lux Amylose-1, *i*-PrOH/Hexanes (V/V) = 95:5, 1.0 mL/min; t_r (major) = 30.190 min, t_r (minor) = 32.887 min). **36b** was determined to be of > 20:1 dr by ^{19}F NMR.^{1}H NMR (500 MHz, $CDCl_3$) δ 7.65−7.64 (d, *J* = 7.8 Hz,1H), 7.59−7.58 (d, *J* = 8.4 Hz,1H), 7.39-7.35 (m, 6H), 7.31-7.27 (m, 2H), 6.14-6.02 (dd, *J* = 3.5, 56.4 Hz, 1H), 4.10-4.02 (m, 1H), 3.96-3.91 (m, 1H), 3.35-3.25 (m, 1H); ^{13}C NMR (126 MHz, $CDCl_3$) δ 155.5, 147.2, 145.4, 135.6, 129.0, 128.1, 127.4, 126.5, 123.5, 122.2, 112.0, 109.4, 104.9 (d, *J* = 230.8 Hz), 43.5 (d, *J* = 4.9 Hz), 41.8 (d, *J* = 21.3 Hz); ^{19}F NMR (376 MHz, $CDCl_3$) δ -117.4 (s, 1F), -132.5 (s, 26F). HRMS (ESI-TOF) Calc'd for $C_{18}H_{14}FNO_2$ $[M+H]^+$: 296.1081; found 296.1078.

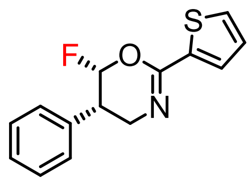

37b: Prepared according to general procedure A using **37a** (48.6 mg, 0.2 mmol). After work-up, the crude residue was purified by flash column chromatography on basic Al_2O_3 (EtOAc-Hexanes (0.5% Et_3N) elution: hexanes/EtOAc (V/V) = 25:1) to give **37b** (35.5 mg, 68%) as a white solid. R_f = 0.14 (PE/EA (V/V) = 25:1). **37b** was determined to be of 92% e.e. by HPLC (phenomenex Lux Amylose-1, *i*-PrOH/Hexanes (V/V) = 99:1, 0.5 mL/min; t_r (major) = 59.977 min, t_r (minor) = 69.660 min). **37b** was determined to be of > 20:1 dr by ^{19}F NMR. ^{1}H NMR (400 MHz, $CDCl_3$) δ 7.51-7.50 (d, *J* = 3.4 Hz, 1H), 7.34-7.26 (m, 6H), 6.99-6.97 (m, 1H), 6.04-5.90 (dd, *J* = 4.0, 56.4 Hz, 1H), 3.99-3.86 (m, 1H), 3.74-3.67 (m, 1H), 3.23-3.11 (dddd, *J* = 1.2, 6.0, 13.2, 30.0 Hz, 1H); ^{13}C NMR (101 MHz, $CDCl_3$) δ 148.4, 136.5, 135.9, 129.4, 128.9, 128.8, 128.5, 128.0, 127.5, 105.0 (d, *J* = 230.5 Hz), 43.4 (d, *J* = 5.1 Hz), 41.7 (d, *J* = 21.3 Hz); ^{19}F NMR (376 MHz, $CDCl_3$) δ -117.3 (s, 1F), -132.3 (s, 25F). HRMS (ESI-TOF) Calc'd for $C_{14}H_{12}FNOS$ $[M+H]^+$: 262.0696; found 262.0668.

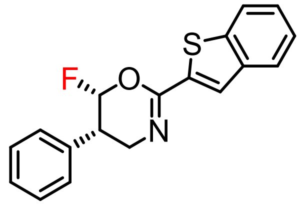

38b: Prepared according to general procedure A using **38a** (58.6 mg, 0.2 mmol). After work-up, the crude residue was purified by flash column chromatography on basic Al_2O_3 (EtOAc-Hexanes (0.5% Et_3N) elution: hexanes/EtOAc (V/V) = 25:1) to give **38b** (37.9 mg, 61%) as a white solid. R_f = 0.16 (PE/EA (V/V) = 25:1). **38b** was determined to be of 98% e.e. by HPLC (phenomenex Cellulose-1, *i*-PrOH/Hexanes (V/V) = 90:10, 1.0 mL/min; t_r (major) = 27.777 min, t_r (minor) = 21.503 min). **38b** was determined to be of 10:1 dr by ^{19}F NMR. ^{1}H NMR (400 MHz, $CDCl_3$) δ 7.66-7.64 (d, *J* = 8.0 Hz, 1H), 7.60-7.58 (d, *J* = 8.0 Hz, 1H), 7.44-7.35 (m, 6H), 7.30-7.26 (m, 2H), 6.16-6.01 (dd, *J* = 3.6, 56.4 Hz, 1H), 4.19-4.03 (m, 1H), 3.97-3.90 (m, 1H), 3.37-3.24 (dddd, *J* = 1.2, 6.0, 13.2, 30.0 Hz, 1H); ^{13}C NMR (101 MHz, $CDCl_3$) δ 155.5, 147.2, 145.4, 135.6, 129.0, 128.5, 128.1, 127.7, 126.5, 123.5, 122.2, 112.0, 109.4, 104.9 (d, *J* = 231.4 Hz), 43.4 (d, *J* = 4.8 Hz), 41.7, 41.5 (d, *J* = 21.1 Hz); ^{19}F NMR (376 MHz, $CDCl_3$) δ -117.4 (s, 1F), -132.5 (s, 10F). HRMS (ESI-TOF) Calc'd for $C_{18}H_{14}FNOS$ $[M+H]^+$: 312.0853; found 312.0853.

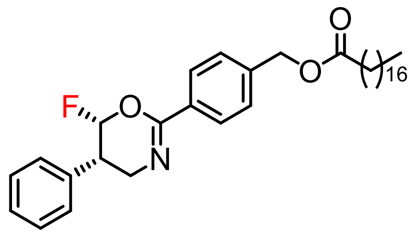

39b: Prepared according to general procedure A using **39a** (106.6 mg, 0.2 mmol). After work-up, the crude residue was purified by flash column chromatography on basic Al_2O_3 (EtOAc-Hexanes (0.5% Et_3N) elution: hexanes/EtOAc (V/V) = 10:1) to give **39b** (66.1 mg, 60%) as a white solid. R_f = 0.34 (PE/EA (V/V) = 10:1). **39b** was determined to be of 89% e.e. by HPLC (phenomenex Lux Amylose-1, *i*-PrOH/Hexanes (V/V) = 98.7:1.3, 1.0 mL/min; t_r (major) = 25.613 min, t_r (minor) = 28.493 min). **39b** was determined to be of 6:1 dr by ^{19}F NMR. ^{1}H NMR (400 MHz, $CDCl_3$) δ 7.91−7.88 (m, 2H), 7.34-7.28 (m, 6H), 7.21-7.19 (d, *J* = 8.0 Hz, 1H), 6.09-5.94 (dd, *J* = 4.0, 56.4 Hz, 1H), 5.08 (s, 2H), 4.15-3.89 (m, 1H), 3.84-3.69 (m, 1H), 3.23-3.11 (dddd, *J* = 1.2, 6.0, 13.2, 30.0 Hz, 1H), 2.32-2.28 (t, *J* = 6.8 Hz, 2H), 1.63-1.56 (m, 2H), 1.22-1.18 (m, 28H), 0.82-0.79 (t, *J* = 6.0 Hz, 3H); ^{13}C NMR (101 MHz, $CDCl_3$) δ 173.6, 151.2, 139.1, 136.0, 132.1, 129.1, 128.9, 128.8, 128.5, 127.7, 126.7, 104.9 (d, *J* = 229.2 Hz), 80.7, 65.5, 53.9, 49.8, 43.5 (d, *J* = 5.2 Hz), 41.7 (d, *J* = 21.2 Hz), 34.3, 32.0, 29.7, 25.0, 22.7, 14.2; ^{19}F NMR (376 MHz, $CDCl_3$) δ -117.1 (s, 1F), -132.0 (s, 6F).

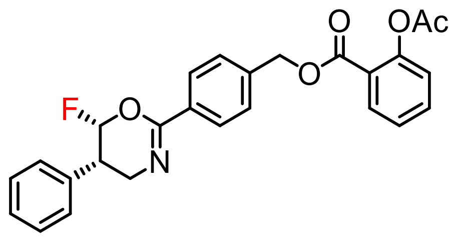

40b: Prepared according to general procedure A using **40a** (85.8 mg, 0.2 mmol). After work-up, the crude residue was purified by flash column chromatography on basic Al_2O_3 (EtOAc-Hexanes (0.5% Et_3N) elution: hexanes/EtOAc (V/V) = 5:1) to give **40b** (51.9 mg, 58%) as a white solid. R_f = 0.25 (PE/EA (V/V) = 5:1). **40b** was determined to be of 80% e.e. by HPLC (phenomenex Lux Amylose-1, *i*-PrOH/Hexanes (V/V) = 88:12, 1.0 mL/min; t_r (major) = 57.133 min, t_r (minor) = 50.663 min). **40b** was determined to be of > 20:1 dr by ^{19}F NMR. ^{1}H NMR (500 MHz, $CDCl_3$) δ 8.07-8.06 (d, *J* = 8.0 Hz, 1H), 8.01-7.99 (d, *J* = 8.0 Hz, 1H), 7.58-7.55 (t, *J* = 8.0 Hz, 1H), 7.46-7.45 (d, *J* = 8.5 Hz, 2H), 7.42-7.35 (m, 5H), 7.34-7.32 (d, *J* = 8.5 Hz, 1H), 7.10-7.09 (d, *J* = 8.0 Hz, 1H), 6.15-6.03 (dd, *J* = 4.0, 56.4 Hz, 1H), 5.34 (s, 2H), 4.04-3.91 (m, 1H), 3.80-3.75 (m, 1H), 3.23-3.13 (dddd, *J* = 1.2, 6.0, 13.2, 30.0 Hz, 1H), 2.16 (s, 3H); ^{13}C NMR (126 MHz, $CDCl_3$) δ 168.7, 163.3, 150.1, 149.7, 137.5, 134.9, 133.1, 130.9, 127.9, 127.6, 127.5, 127.0, 126.9, 126.6, 125.1, 122.9, 122.1, 103.9 (d, *J* = 228.9 Hz), 65.4, 42.4 (d, *J* = 5.2 Hz), 40.6 (d, *J* = 21.2 Hz), 19.8; ^{19}F NMR (376 MHz, $CDCl_3$) δ -117.2 (s, 1F), -132.0 (s, 8F). HRMS (ESI-TOF) Calc'd for $C_{26}H_{22}FNO_5$ $[M+H]^+$: 448.1555; found 448.1557.

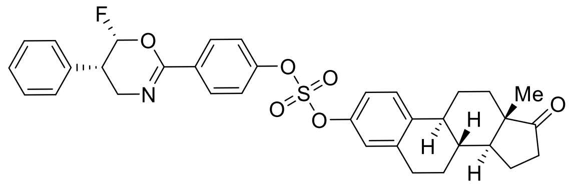

41b: Prepared according to general procedure A using **41a** (117.0 mg, 0.2 mmol). After work-up, the crude residue was purified by flash column chromatography on basic Al_2O_3 (EtOAc-Hexanes (0.5% Et_3N) elution: hexanes/EtOAc (V/V) = 5:1) to give **41b** (76.0 mg, 63%) as a white solid. R_f = 0.15 (PE/EA (V/V) = 5:1). **41b** was determined to be of 80% e.e. by HPLC (phenomenex Lux Amylose-1, *i*-PrOH/Hexanes (V/V) = 75:25, 0.8 mL/min; t_r (major) = 40.487 min, t_r (minor) = 35.973 min). **41b** was determined to be of 13:1 dr by ^{19}F NMR. ^{1}H NMR (500 MHz, $CDCl_3$) δ 8.07-8.05 (d, *J* = 8.8 Hz, 2H), 7.40-7.35 (m, 6H), 7.33-7.31 (d, *J* = 8.5 Hz, 1H), 7.26 (s, 1H), 7.08-7.05 (m, 2H), 6.15-6.03 (dd, *J* = 4.0, 56.5 Hz, 1H), 4.03-3.98 (m, 1H), 3.87-3.82 (m, 1H), 3.29-3.20 (m, 1H), 2.94-2.92 (m, 2H), 2.55-2.49 (dd, *J* = 19.0, 8.7 Hz, 1H), 2.41-2.39 (m, 1H), 2.32-2.30 (m, 1H), 2.17-1.97 (m, 4H), 1.66-1.62 (m, 3H), 1.54-1.46 (m, 3H), 0.92 (s, 3H). ^{13}C NMR (126 MHz, $CDCl_3$) δ 220.5, 152.3, 150.3, 148.3, 139.6, 139.0, 135.8, 131.7, 129.3, 128.9, 128.5, 128.1, 127.0, 121.0, 120.8, 118.1, 104.9 (d, *J* = 229.4 Hz), 50.4, 47.9, 44.1, 43.5 (d, *J* = 4.9 Hz), 41.6 (d, *J* = 21.3 Hz), 37.9, 35.8, 31.5, 29.4, 26.2, 25.7, 21.6, 13.8; ^{19}F NMR (376 MHz, $CDCl_3$) δ -117.1 (s, 1F), -132.1 (s, 13F). HRMS (ESI-TOF) Calc'd for $C_{34}H_{34}FNO_6S$ $[M+H]^+$: 604.2164; found 604.2167.

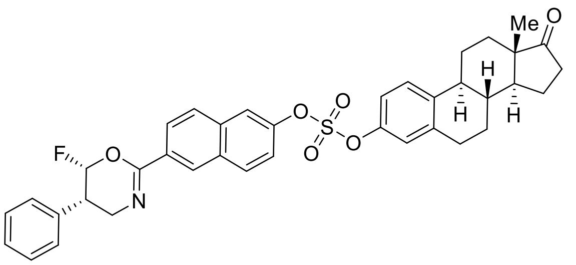

42b: Prepared according to general procedure A using **42a** (127.0 mg, 0.2 mmol). After work-up, the crude residue was purified by flash column chromatography on basic Al_2O_3 (EtOAc-Hexanes (0.5% Et_3N) elution: hexanes/EtOAc (V/V) = 5:1) to give **42b** (58.6 mg, 45%) as a white solid. R_f = 0.25 (PE/EA (V/V) = 3:1). **42b** was determined to be of 92% e.e. by HPLC (AD, *i*-PrOH/Hexanes (V/V) = 80:20, 1.0 mL/min; t_r (major) = 98.993 min, t_r (minor) = 124.287 min). **42b** was

determined to be of > 20:1 dr by ^{19}F NMR. ^{1}H NMR (500 MHz, $CDCl_3$) δ 8.51 (s, 1H), 8.17-8.15 (d, *J* = 8.5 Hz, 1H), 7.99-7.97 (d, *J* = 9.0 Hz, 1H), 7.88-7.86 (d, J = 8.5 Hz, 1H), 7.83 (s, 1H), 7.49-7.32 (m, 7H), 7.12-7.10 (d, *J* = 8.5 Hz, 1H), 7.07 (s, 1H), 6.22-6.10 (dd, *J* = 4.0, 56.5 Hz, 1H), 4.16-3.88 (m, 2H), 3.35-3.26 (m, 1H), 2.92-2.90 (m, 2H), 2.54-2.49 (dd, *J* = 19.0, 8.5 Hz, 1H), 2.41-2.38 (m, 1H), 2.31-2.27 (t, *J* = 11.0 Hz, 1H), 2.17-2.12 (m, 1H), 2.09-2.04 (m, 2H), 1.99-1.97 (m, 1H), 1.65-1.59 (m, 2H), 1.53-1.43 (m, 4H), 0.92 (s, 3H). ^{13}C NMR (126 MHz, $CDCl_3$) δ 220.5, 155.2, 151.1, 149.0, 148.4, 139.5, 139.0, 135.9, 134.8, 131.4, 130.6, 128.9, 128.5, 128.0, 127.7, 127.0, 125.6, 121.0, 120.4, 118.5, 118.1, 105.1 (d, *J* = 229.1 Hz), 50.4, 47.9, 44.1, 43.6 (d, *J* = 4.9 Hz), 41.7 (d, *J* = 21.3 Hz), 37.9, 35.8, 31.5, 29.4, 26.2, 25.7, 21.6, 14.2; ^{19}F NMR (376 MHz, $CDCl_3$) δ -117.1 (s, 1F), -131.9 (s, 42F). HRMS (ESI-TOF) Calc'd for $C_{38}H_{36}FNO_6S$ $[M+H]^+$: 654.2320; found 654.2318.

5.2 General Procedure B (Synthesis of 43b-54b)

The substrate (0.2 mmol) and catalyst (20 mol%) were mixed into the reaction tube, and then C_6H_5F (8.0 ml) was added. The mixture was cooled to −42°C, after stirring for 5 min at this temperature, *m*-CPBA (1.2 equiv) was added in one portion, followed by addition of $BF_3{\cdot}Et_2O$ (10.0 equiv) dropwise. The reaction was run at −42°C for 20 h. The reaction mixture was poured into *s*-$NaHCO_3$ (aq) sulotion, the organic layer was collected and washed with saturated $NaHCO_3$ (aq), brine, dried over Na_2SO_4 concentrated under reduced pressure in the presence of basic Al_2O_3, Column chromatography (basic Al_2O_3, EtOAc-Hexanes (0.5% Et_3N) elution: hexanes/EtOAc (V/V) = 100:1~25:1) gave the corresponding fluorinated products.

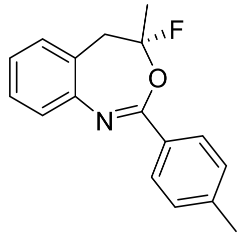

43b: Prepared according to general procedure B using **43a** (50.3 mg, 0.2 mmol). After work-up, the crude residue was purified by flash column chromatography on basic Al_2O_3 (EtOAc-Hexanes (0.5% Et_3N) elution: hexanes/EtOAc (V/V) = 100:1) to give **43b** (47.3 mg, 88%) as a white solid. R_f = 0.44 (PE/EA (V/V) = 50:1). **43b** was determined to be of 85% e.e. by HPLC (phenomenex Cellulose-1, *i*-PrOH/Hexanes (V/V) = 95:5, 1.0 mL/min; t_r (major) = 12.583 min, t_r (minor) = 9.023 min). ^{1}H NMR (500 MHz, $CDCl_3$) δ 8.01-8.00 (d, 2H, *J* = 8.2 Hz), 7.29-7.26 (t, 1H, *J* = 8.0 Hz), 7.21-7.13 (m, 4H), 7.10-7.07 (t, 1H, *J* = 8.0 Hz), 3.16-3.11 (dd, 1H, *J* = 9.5 Hz, 14.0 Hz), 3.00-2.97 (dd, 1H, *J* = 2 Hz, 14.5 Hz), 2.34 (s, 3H), 1.62-1.59 (d, 3H, *J* = 17.5 Hz); ^{13}C NMR (126 MHz, $CDCl_3$) δ 153.6, 144.5, 142.0, 129.1, 129.0, 128.9, 128.4, 127.5, 127.4, 125.5, 123.0 (d, *J* = 228.7 Hz), 42.3 (d, *J* = 31.5 Hz), 24.5 (d, *J* = 30.0 Hz), 21.6; ^{19}F NMR (376 MHz, $CDCl_3$) δ -72.23. HRMS (ESI-TOF) Calc'd for $C_{17}H_{16}FNO$ $[M+H]^+$: 270.1289; found 270.1288.

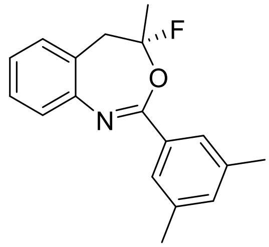

44b: Prepared according to general procedure B using **44a** (53.0 mg, 0.2 mmol). After work-up, the crude residue was purified by flash column chromatography on basic Al_2O_3 (EtOAc-Hexanes (0.5% Et_3N) elution: hexanes/EtOAc (V/V) = 100:1) to give **44b** (48.1 mg, 85%) as a white solid. R_f = 0.40 (PE/EA (V/V) = 50:1). **44b** was determined to be of 80% e.e. by HPLC (phenomenex Lux Amylose-1, *i*-PrOH/Hexanes (V/V) = 99:1, 1.0 mL/min; t_r (major) = 5.763 min, t_r (minor) = 5.457 min). ^{1}H NMR (500 MHz, $CDCl_3$) δ 7.73 (s, 2H), 7.30-7.27 (t, 1H, *J* = 7.5 Hz), 7.22-7.21 (d, 1H, *J* = 8.0 Hz), 7.14-7.13 (d, 1H, *J* = 8.0 Hz), 7.10-7.08 (d, 1H, *J* = 7.5 Hz), 7.07 (s, 1H), 3.15-3.10 (dd, 1H, *J* = 9.5 Hz,

14.0 Hz), 3.00-2.97 (d, 1H, *J* = 14.5 Hz), 2.31 (s, 6H), 1.62-1.59 (d, 3H, *J* = 17.5 Hz); ^{13}C NMR (126 MHz, $CDCl_3$) δ 151.5, 142.8, 138.4, 135.1, 133.6, 129.5, 128.4, 127.9, 126.2, 117.7 (d, *J* = 224.9 Hz), 42.3 (d, *J* = 31.5 Hz), 24.6 (d, *J* = 30.0 Hz), 21.3; ^{19}F NMR (376 MHz, $CDCl_3$) δ -78.35. HRMS (ESI-TOF) Calc'd for $C_{18}H_{18}FNO$ $[M+H]^+$: 298.1602; found 298.1602.

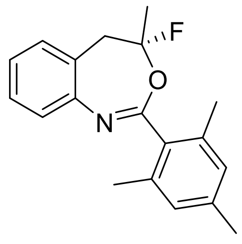

45b: Prepared according to general procedure B using **45a** (55.8 mg, 0.2 mmol). After work-up, the crude residue was purified by flash column chromatography on basic Al_2O_3 (EtOAc-Hexanes (0.5% Et_3N) elution: hexanes/EtOAc (V/V) = 100:1) to give **45b** (48.7 mg, 82%) as a white solid. R_f = 0.40 (PE/EA (V/V) = 50:1). **45b** was determined to be of 85% e.e. by HPLC (phenomenex Lux Amylose-1, *i*-PrOH/Hexanes (V/V) = 99:1, 1.0 mL/min; t_r (major) = 12.357 min, t_r (minor) = 10.830 min). ^{1}H NMR (400 MHz, $CDCl_3$) δ 7.39-7.36 (m, 2H), 7.24-7.23 (m, 2H), 6.94 (s, 2H), 3.49-3.45 (dd, 1H, *J* = 2.8 Hz, 14.8 Hz), 3.39-3.32 (dd, 1H, *J* = 11.6 Hz, 14.8 Hz), 2.44 (s, 6H), 2.33 (s, 3H), 1.71-1.66 (d, 3H, *J* = 17.5 Hz); ^{13}C NMR (101 MHz, $CDCl_3$) δ 153.9, 144.4, 137.9, 133.5, 133.3, 129.0, 128.4, 127.4, 126.7, 125.6, 125.4, 123.0 (d, *J* = 228.7 Hz), 43.8 (d, *J* = 31.5 Hz), 26.1 (d, *J* = 17.6 Hz), 21.1, 19.6; ^{19}F NMR (376 MHz, $CDCl_3$) δ -71.93. HRMS (ESI-TOF) Calc'd for $C_{19}H_{20}FNO$ $[M+H]^+$: 298.1602; found 298.1602.

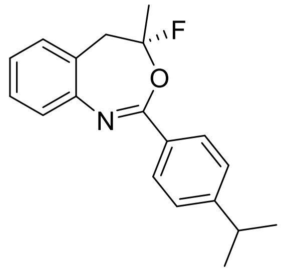

46b: Prepared according to general procedure B using **46a** (55.8 mg, 0.2 mmol). After work-up, the crude residue was purified by flash column chromatography on basic Al_2O_3 (EtOAc-Hexanes (0.5% Et_3N) elution: hexanes/EtOAc (V/V) = 100:1) to give **46b** (51.1 mg, 86%) as a white solid. R_f = 0.41 (PE/EA (V/V) = 50:1). **46b** was determined to be of 81% e.e. by HPLC (phenomenex Cellulose-1, *i*-PrOH/Hexanes (V/V) = 95:5, 1.0 mL/min; t_r (major) = 13.100 min, t_r (minor) = 9.283 min). ^{1}H NMR (500 MHz, $CDCl_3$) δ 8.05-8.03 (d, 2H, *J* = 8.0 Hz), 7.29-7.26 (t, 1H, *J* = 8.0 Hz), 7.24-7.20 (m, 3H), 7.14-7.13 (d, 1H, *J* = 8.0 Hz), 7.09-7.06 (t, 1H, *J* = 8.0 Hz), 3.15-3.11 (dd, 1H, *J* = 9.5 Hz, 14.0 Hz), 3.00-2.97 (d, 1H, *J* = 14.5 Hz), 2.92-2.87 (m, 1H), 1.62-1.58 (d, 3H, *J* = 17.5 Hz), 1.21-1.20 (d, 6H, *J* = 7.0 Hz); ^{13}C NMR (126 MHz, $CDCl_3$) δ 153.7, 152.8, 144.5, 131.3, 129.1, 129.0, 128.4, 127.5, 127.4, 126.4, 125.5, 125.4, 123.1 (d, *J* = 228.6 Hz), 42.3 (d, *J* = 31.5 Hz), 34.2, 24.6 (d, *J* = 30.0 Hz), 23.8; ^{19}F NMR (376 MHz, $CDCl_3$) δ -72.12. HRMS (ESI-TOF) Calc'd for $C_{19}H_{20}FNO$ $[M+H]^+$: 298.1602; found 298.1603.

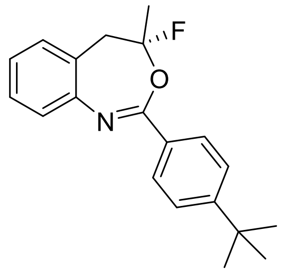

47b: Prepared according to general procedure B using **47a** (62.2 mg, 0.2 mmol). After work-up, the crude residue was purified by flash column chromatography on basic Al_2O_3 (EtOAc-Hexanes (0.5% Et_3N) elution: hexanes/EtOAc (V/V) = 100:1) to give **47b** (51.6 mg, 83%) as a white solid. R_f = 0.45 (PE/EA (V/V) = 50:1). **47b** was determined to be of 83% e.e. by HPLC (phenomenex Lux Amylose-1, *i*-PrOH/Hexanes (V/V) = 99:1, 0.5 mL/min; t_r (major) = 6.400 min, t_r (minor) = 5.497 min). ^{1}H NMR (400 MHz, $CDCl_3$) δ 8.05-8.03 (d, 2H, *J* = 8.8 Hz), 7.41-7.38 (d, 2H, *J* = 8.8 Hz), 7.30-7.26 (t, 1H, *J* = 8.0 Hz), 7.22-7.20 (d, 1H, *J* = 8.0 Hz), 7.15-7.13 (d, 1H, *J* = 8.0 Hz), 7.10-7.06 (t, 1H, *J* = 8.0 Hz), 3.16-3.10 (dd, 1H, *J* = 9.5 Hz, 14.0 Hz), 3.00-2.97 (dd, 1H, *J* = 2.0 Hz, 14.5 Hz), 1.63-1.58 (d, 3H, *J* = 17.6 Hz), 1.28 (s, 9H); ^{13}C NMR (101 MHz, $CDCl_3$) δ 155.0, 153.6, 144.5, 130.9, 129.0, 128.8, 128.4, 127.5, 125.5, 125.4, 125.3, 123.2 (d, *J* = 229.0 Hz), 42.3

(d, J = 31.1 Hz), 35.0, 31.2, 24.5 (d, J = 30.0 Hz); ^{19}F NMR (376 MHz, $CDCl_3$) δ -72.11. HRMS (ESI-TOF) Calc'd for $C_{20}H_{22}FNO_3$ $[M+H]^+$: 312.1758; found 312.1761.

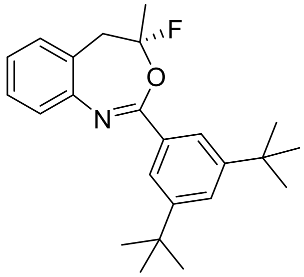

48b: Prepared according to general procedure B using **48a** (69.8 mg, 0.2 mmol). After work-up, the crude residue was purified by flash column chromatography on basic Al_2O_3 (EtOAc-Hexanes (0.5% Et_3N) elution: hexanes/EtOAc (V/V) = 100:1) to give **48b** (59.4 mg, 81%) as a white solid. R_f = 0.48 (PE/EA (V/V) = 50:1). **48b** was determined to be of 85% e.e. by HPLC (phenomenex Cellulose-1, *i*-PrOH/Hexanes (V/V) = 99.9:0.1, 0.5 mL/min; t_r (major) = 10.577 min, t_r (minor) = 13.407 min). ^{1}H NMR (400 MHz, $CDCl_3$) δ 7.98 (s, 2H), 7.51 (s, 1H), 7.27-7.22 (m, 2H), 7.13-7.12 (d, 1H, J = 8.0 Hz), 7.08-7.05 (t, 1H, J = 8.0 Hz), 3.16-3.11 (dd, 1H, J = 9.5 Hz, 14.0 Hz), 3.00-2.97 (d, 1H, J = 14.5 Hz), 1.62-1.58 (d, 3H, J = 17.5 Hz), 1.30 (s, 18H); ^{13}C NMR (101 MHz, $CDCl_3$) δ 154.4, 150.8, 144.7, 132.9, 129.0, 128.3, 127.5, 125.9, 125.5, 123.3, 123.8 (d, J = 229.2 Hz), 42.3 (d, J = 31.1 Hz), 35.0, 31.5, 24.6 (d, J = 30.0 Hz); ^{19}F NMR (376 MHz, $CDCl_3$) δ -72.23. HRMS (ESI-TOF) Calc'd for $C_{24}H_{30}FNO$ $[M+H]^+$: 368.2384; found 368.2384.

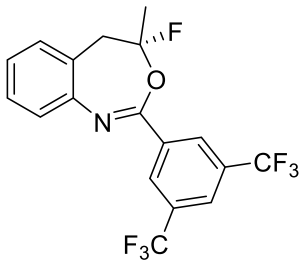

49b: Prepared according to general procedure B using **49a** (74.6 mg, 0.2 mmol). After work-up, the crude residue was purified by flash column chromatography on basic Al_2O_3 (EtOAc-Hexanes (0.5% Et_3N) elution: hexanes/EtOAc (V/V) = 100:1) to give **49b** (66.5 mg, 85%) as a white solid. R_f = 0.35 (PE/EA (V/V) = 50:1). **49b** was determined to be of 80% e.e. by HPLC (phenomenex Cellulose-1, *i*-PrOH/Hexanes (V/V) = 99:1, 1.0 mL/min; t_r (major) = 4.703 min, t_r (minor) = 9.920 min). ^{1}H NMR (400 MHz, $CDCl_3$) δ 8.55 (s, 2H), 7.92 (s, 1H), 7.36-7.28 (m, 2H), 7.19-7.17 (m, 2H), 3.18-3.07 (m, 2H), 1.69-1.65 (d, 3H, J = 17.6 Hz); ^{13}C NMR (101 MHz, $CDCl_3$) δ 143.1, 136.2, 132.0, 131.7, 129.3, 128.8, 128.6, 127.5, 127.4, 126.7, 126.2, 124.6 (q, J = 3.2 Hz), 122.5 (d, J = 229.2 Hz), 42.6 (d, J = 31.1 Hz), 24.7 (d, J = 30.0 Hz); ^{19}F NMR (376 MHz, $CDCl_3$) δ -73.22 (s, 1F), -62.85 (s, 6F). HRMS (ESI-TOF) Calc'd for $C_{18}H_{12}F_7NO_3$ $[M+H]^+$: 392.0880; found 392.0883.

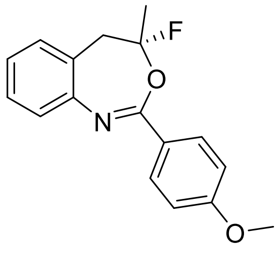

50b: Prepared according to general procedure B using **50a** (53.4 mg, 0.2 mmol). After work-up, the crude residue was purified by flash column chromatography on basic Al_2O_3 (EtOAc-Hexanes (0.5% Et_3N) elution: hexanes/EtOAc (V/V) = 100:1) to give **50b** (49.5 mg, 87%) as a white solid. R_f = 0.25 (PE/EA (V/V) = 20:1). **50b** was determined to be of 85% e.e. by HPLC (phenomenex Lux Amylose-1, *i*-PrOH/Hexanes (V/V) = 98:2, 1.0 mL/min; t_r (major) = 18.730 min, t_r (minor) = 22.327 min). ^{1}H NMR (400 MHz, $CDCl_3$) δ 8.07-8.05 (d, 2H, J = 9.2 Hz), 7.29-7.25 (d, 1H, J = 8.0 Hz), 7.19-7.17 (d, 1H, J = 8.0 Hz), 7.14-7.12 (d, 1H, J = 8.0 Hz),7.08-7.05 (t, 1H, J = 8.0 Hz), 6.88-6.86 (d, 2H, J = 8.8 Hz), 3.78 (s, 3H), 3.16-3.10 (dd, 1H, J = 9.5 Hz, 14.0 Hz), 2.98-2.94 (dd, 1H, J = 2.0 Hz, 14.5 Hz), 1.62-1.57 (d, 3H, J = 17.6 Hz); ^{13}C NMR (101 MHz, $CDCl_3$) δ 162.5, 153.5, 144.6, 130.8, 129.0, 128.4, 127.5, 127.4, 126.1, 125.4, 125.2, 123.2 (d, J = 229.0 Hz), 113.7, 55.4, 42.3 (d, J = 31.1 Hz), 24.5 (d, J = 30.0 Hz); ^{19}F NMR (376 MHz, $CDCl_3$) δ -77.04. HRMS (ESI-TOF) Calc'd for $C_{17}H_{16}FNO_2$ $[M+H]^+$: 286.1238; found 286.1237.

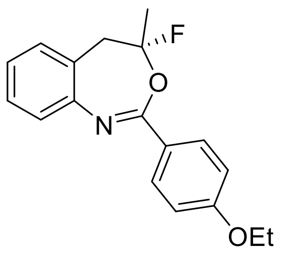

51b: Prepared according to general procedure B using **51a** (56.2 mg, 0.2 mmol). After work-up, the crude residue was purified by flash column chromatography on basic Al_2O_3 (EtOAc-Hexanes (0.5% Et_3N) elution: hexanes/EtOAc (V/V) = 100:1) to give **51b** (50.5 mg, 85%) as a white solid. R_f = 0.32 (PE/EA (V/V) = 20:1). **51b** was determined to be of 83% e.e. by HPLC (phenomenex Cellulose-1, *i*-PrOH/Hexanes (V/V) = 98:2, 1.0 mL/min; t_r (major) = 24.167 min, t_r (minor) = 18.230 min). ^{1}H NMR (400 MHz, $CDCl_3$) δ 8.06-8.03 (d, 2H, *J* = 8.8 Hz), 7.29-7.25 (t, 1H, *J* = 8.0 Hz), 7.19-7.17 (d, 1H, *J* = 8.0 Hz), 7.13-7.12 (d, 1H, *J* = 8.0 Hz),7.08-7.04 (t, 1H, *J* = 8.0 Hz), 6.86-6.84 (d, 2H, *J* = 9.2 Hz), 4.04-3.99 (q, 2H, *J* = 7.2 Hz), 3.16-3.10 (dd, 1H, *J* = 9.5 Hz, 14.0 Hz), 2.98-2.94 (dd, 1H, *J* = 2.0 Hz, 14.5 Hz), 1.61-1.57 (d, 3H, *J* = 17.6 Hz), 1.38-1.34 (t, 3H, *J* = 6.8 Hz); ^{13}C NMR (101 MHz, $CDCl_3$) δ 161.9, 153.6, 144.7, 130.8, 129.0, 128.4, 127.5, 127.4, 125.9, 125.3, 123.6 (d, *J* = 229.0 Hz), 114.1, 63.64, 42.3 (d, *J* = 31.1 Hz), 24.5 (d, *J* = 30.0 Hz), 14.8; ^{19}F NMR (376 MHz, $CDCl_3$) δ -72.03. HRMS (ESI-TOF) Calc'd for $C_{18}H_{18}FNO_2$ $[M+H]^+$: 300.1394; found 300.1394.

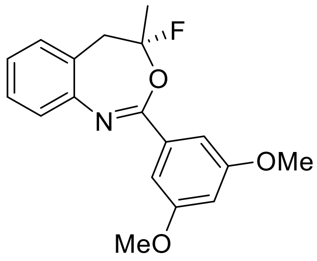

52b: Prepared according to general procedure B using **52a** (59.4 mg, 0.2 mmol). After work-up, the crude residue was purified by flash column chromatography on basic Al_2O_3 (EtOAc-Hexanes (0.5% Et_3N) elution: hexanes/EtOAc (V/V) = 100:1) to give **52b** (52.3 mg, 83%) as a white solid. R_f = 0.24 (PE/EA (V/V) = 20:1). **52b** was determined to be of 85% e.e. by HPLC (phenomenex Lux Amylose-1, *i*-PrOH/Hexanes (V/V) = 99:1, 1.0 mL/min; t_r (major) = 15.253 min, t_r (minor) = 20.420 min). ^{1}H NMR (400 MHz, $CDCl_3$) δ 7.31 (s, 2H), 7.24-7.22 (d, 1H, *J* = 8.0 Hz), 7.16-7.14 (d, 1H, *J* = 8.0 Hz), 7.12-7.09 (t, 1H, *J* = 8.0 Hz), 6.55 (s, 1H), 3.80 (s, 6H), 3.16-3.11 (dd, 1H, *J* = 9.5 Hz, 14.0 Hz), 3.02-2.99 (d, 1H, *J* = 14.5 Hz), 1.62-1.59 (d, 3H, *J* = 17.6 Hz); ^{13}C NMR (101 MHz, $CDCl_3$) δ 160.6, 153.3, 144.1, 135.7, 129.1, 128.4, 127.4, 125.8, 125.5, 123.1 (d, *J* = 229.0 Hz), 106.8, 104.3, 55.6, 42.3 (d, *J* = 31.1 Hz), 24.6 (d, *J* = 30.0 Hz), 14.8; ^{19}F NMR (376 MHz, $CDCl_3$) δ -72.33. HRMS (ESI-TOF) Calc'd for $C_{18}H_{18}FNO_3$ $[M+H]^+$: 316.1343; found 316.1343.

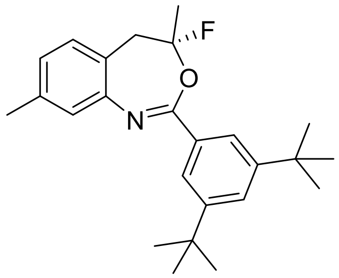

53b: Prepared according to general procedure B using **53a** (72.7 mg, 0.2 mmol). After work-up, the crude residue was purified by flash column chromatography on basic Al_2O_3 (EtOAc-Hexanes (0.5% Et_3N) elution: hexanes/EtOAc (V/V) = 100:1) to give **53b** (62.5 mg, 82%) as a white solid. R_f = 0.30 (PE/EA (V/V) = 50:1). **53b** was determined to be of 81% e.e. by HPLC (phenomenex Lux Amylose-1, *i*-PrOH/Hexanes (V/V) = 99.8:0.2, 0.4 mL/min; t_r (major) = 13.103 min, t_r (minor) = 12.503 min). ^{1}H NMR (400 MHz, $CDCl_3$) δ 7.98 (s, 2H), 7.51 (s, 1H), 7.07 (s, 1H), 7.03-7.02 (d, 1H, *J* = 8.0 Hz), 6.91-6.89 (d, 1H, *J* = 8.0 Hz), 3.13-3.07 (dd, 1H, *J* = 9.5 Hz, 14.0 Hz), 2.96-2.93 (dd, 1H, *J* = 2.0 Hz, 14.5 Hz), 2.31 (s, 3H), 1.63-1.58 (d, 3H, *J* = 17.5 Hz), 1.31 (s, 18H); ^{13}C NMR (101 MHz, $CDCl_3$) δ 150.7, 144.4, 138.2, 132.9, 128.8, 126.2, 125.9, 125.8, 124.6, 123.3 (d, *J* = 229.2 Hz), 123.2, 41.9 (d, *J* = 31.1 Hz), 35.0, 31.5, 24.6 (d, *J* = 30.0 Hz), 21.1; ^{19}F NMR (376 MHz, $CDCl_3$) δ -72.44. HRMS (ESI-TOF) Calc'd for $C_{25}H_{32}FNO$ $[M+H]^+$: 382.2541; found 382.2539.

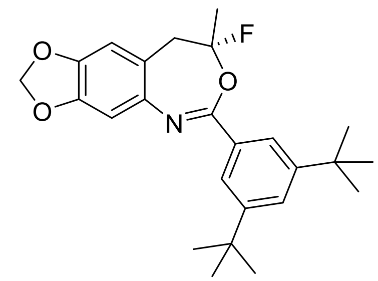

54b: Prepared according to general procedure B using **54a** (78.6 mg, 0.2 mmol). After work-up, the crude residue was purified by flash column chromatography on basic Al_2O_3 (EtOAc-Hexanes (0.5% Et_3N) elution: hexanes/EtOAc (V/V) = 100:1) to give **54b** (65.7 mg, 80%) as a white solid. R_f = 0.16 (PE/EA (V/V) = 20:1). **54b** was determined to be of 80% e.e. by HPLC (phenomenex Lux Amylose-1, *i*-PrOH/Hexanes (V/V) = 99.5:0.5, 1.0 mL/min; t_r (major) = 7.383 min, t_r (minor) = 6.893 min). ^{1}H NMR (500 MHz, $CDCl_3$) δ 8.04 (s, 2H), 7.59 (s, 1H), 7.28 (s, 1H), 6.85 (s, 1H), 6.71 (s, 1H), 6.00 (s, 2H), 3.15-3.00 (dd, 1H, *J* = 9.5 Hz, 14.0 Hz), 2.97-2.93 (dd, 1H, *J* = 2.0 Hz, 14.5 Hz), 1.74-1.69 (d, 3H, *J* = 17.5 Hz), 1.40 (s, 18H); ^{13}C NMR (126 MHz, $CDCl_3$) δ 154.1, 150.8, 147.7, 145.3, 139.1, 132.8, 125.7, 122.5 (d, *J* = 229.2 Hz), 123.1, 108.5, 106.3, 101.3, 42.1 (d, *J* = 31.4 Hz), 35.0, 31.4, 24.5 (d, *J* = 30.0 Hz), 21.1; ^{19}F NMR (376 MHz, $CDCl_3$) δ -72.05. HRMS (ESI-TOF) Calc'd for $C_{25}H_3FNO_3$ $[M+H]^+$: 412.2282; found 412.2271.

6. Chiral HPLC Traces of Aminofluorination Products and Derivatives

6.1 HPLC Traces of 1b-42b

Supplementary Figure 1. HPLC Traces of 1b (phenomenex Cellulose-1, *i*-PrOH/Hexanes (V/V) = 95:5, 1.0 mL/min).

Racemic

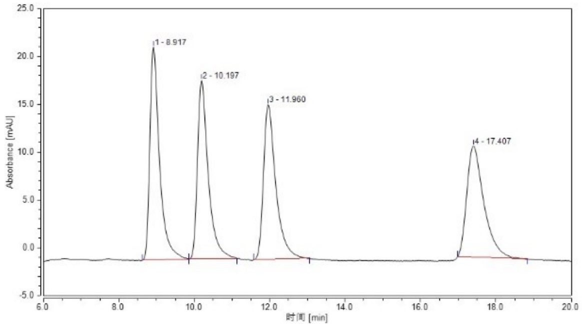

Chiral

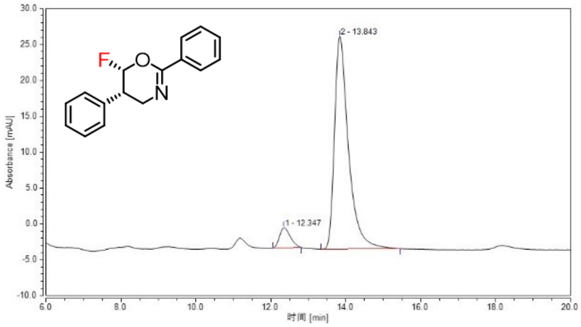

Racemic

Peak	RetTime (min)	Area (mAU*min)	Height (mAU)	Area%	Height%
1	8.917	6.619	22.267	26.54	32.42
2	10.197	6.144	18.654	24.64	27.16
3	11.960	6.097	16.158	24.45	23.53
4	17.407	6.081	11.602	24.38	16.89

Chiral

Peak	RetTime (min)	Area (mAU*min)	Height (mAU)	Area%	Height%
1	12.347	0.943	2.806	6.87	8.65
2	13.843	12.783	29.653	93.13	91.35

Supplementary Figure 2. HPLC Traces of 2b (phenomenex Lux Amylose-1, *i*-PrOH/Hexanes (V/V) = 98:2, 1.0 mL/min).

Racemic

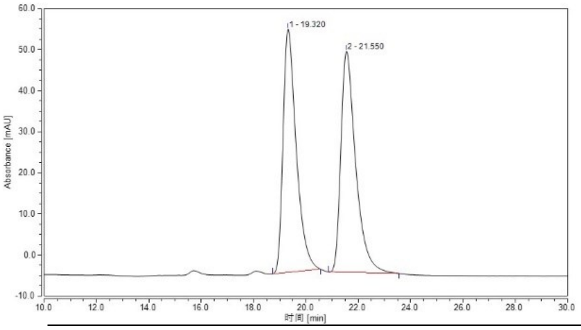

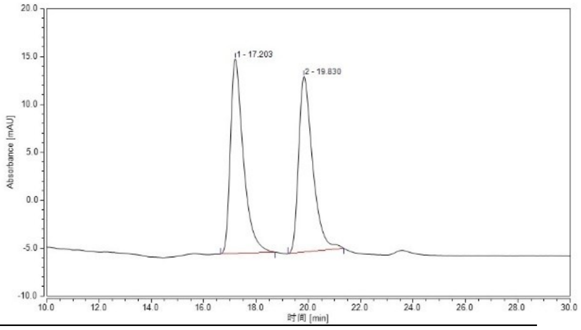

Peak	RetTime (min)	Area (mAU*min)	Height (mAU)	Area%	Height%
1	19.320	34.501	59.268	49.46	52.39
2	21.550	35.257	53.853	50.54	47.61

Peak	RetTime (min)	Area (mAU*min)	Height (mAU)	Area%	Height%
1	17.203	11.698	20.306	50.69	52.55
2	19.830	11.379	18.338	49.31	47.45

Chiral

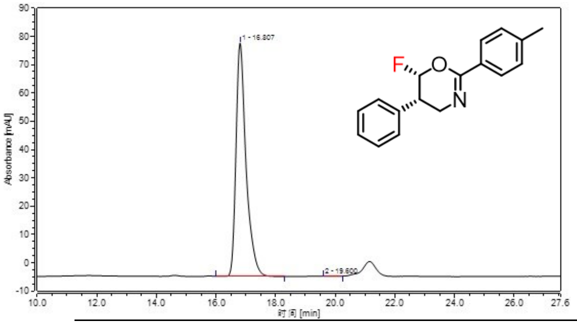

Peak	RetTime (min)	Area (mAU*min)	Height (mAU)	Area%	Height%
1	16.807	32.101	82.418	99.97	100.00
2	19.600	0.009	0.000	0.03	0.00

Supplementary Figure 3. HPLC Traces of 3b (phenomenex Lux Amylose-1, *i*-PrOH/Hexanes (V/V) = 98:2, 1.0 mL/min).

Racemic

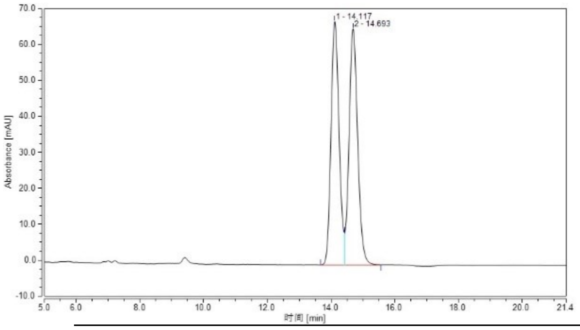

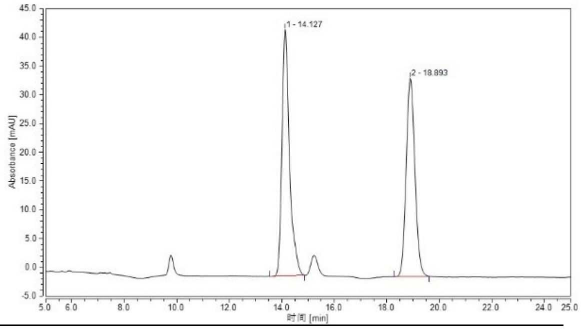

Peak	RetTime (min)	Area (mAU*min)	Height (mAU)	Area%	Height%
1	14.117	19.879	67.740	49.10	50.78
2	14.693	20.605	65.671	50.90	49.22

Peak	RetTime (min)	Area (mAU*min)	Height (mAU)	Area%	Height%
1	14.127	14.261	42.850	51.89	55.39
2	18.893	13.221	34.512	48.11	44.61

Chiral

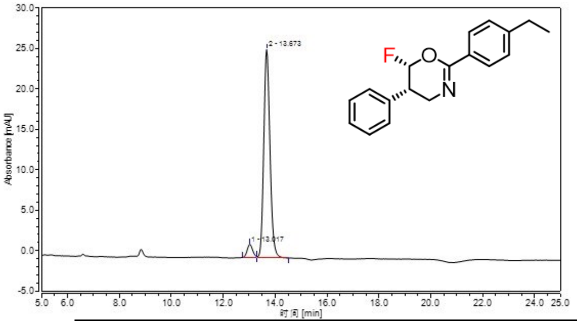

Peak	RetTime (min)	Area (mAU*min)	Height (mAU)	Area%	Height%
1	13.017	0.338	1.495	4.59	5.51
2	13.673	7.019	25.645	95.41	94.49

Supplementary Figure 4. HPLC Traces of 4b (phenomenex Lux Amylose-1, *i*-PrOH/Hexanes (V/V) = 90:10, 1.0 mL/min).

Racemic

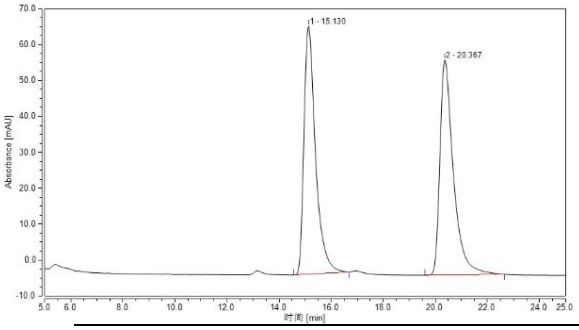

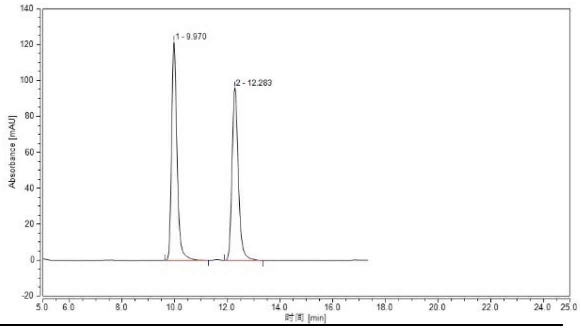

Peak	RetTime (min)	Area (mAU*min)	Height (mAU)	Area%	Height%
1	15.130	35.508	69.127	49.69	53.54
2	20.367	35.950	59.994	50.31	46.46

Peak	RetTime (min)	Area (mAU*min)	Height (mAU)	Area%	Height%
1	9.970	29.090	121.844	52.15	55.82
2	12.283	26.691	96.441	47.85	44.18

Chiral

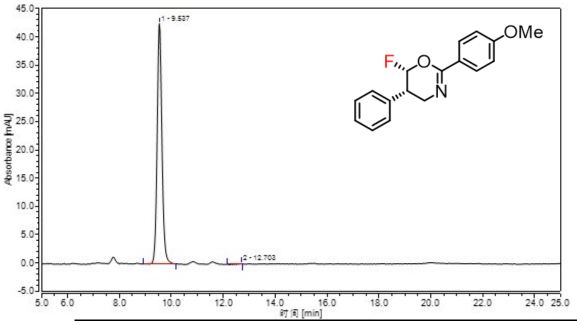

Peak	RetTime (min)	Area (mAU*min)	Height (mAU)	Area%	Height%
1	9.537	9.096	42.541	99.82	99.72
2	12.703	0.017	0.121	0.18	0.28

Supplementary Figure 5. HPLC Traces of 5b (phenomenex Cellulose-1, *i*-PrOH/Hexanes (V/V) = 98:2, 1.0 mL/min).

Racemic

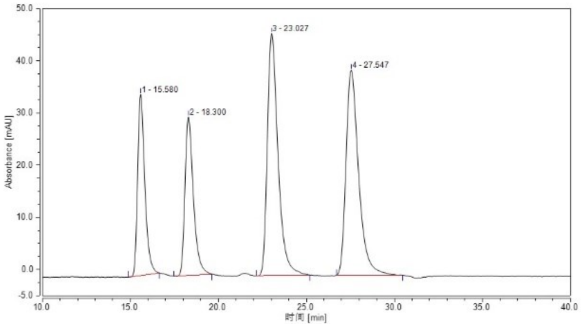

Chiral

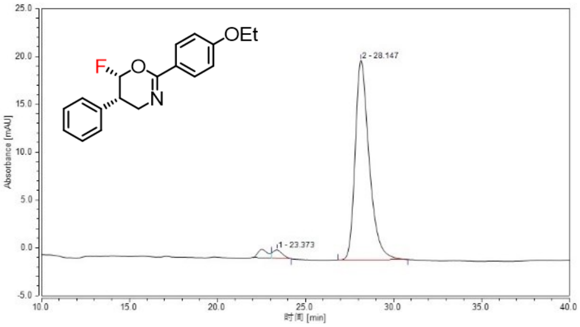

Racemic

Peak	RetTime (min)	Area (mAU*min)	Height (mAU)	Area%	Height%
1	15.580	16.364	34.712	16.64	23.01
2	18.300	16.904	30.403	17.18	20.15
3	23.027	32.533	46.432	33.07	30.78
4	27.547	32.565	39.301	33.11	26.05

Chiral

Peak	RetTime (min)	Area (mAU*min)	Height (mAU)	Area%	Height%
1	23.373	0.547	0.883	2.77	4.06
2	28.147	19.194	20.855	97.23	95.94

Supplementary Figure 6. HPLC Traces of 6b (phenomenex Cellulose-1, *i*-PrOH/Hexanes (V/V) = 95:5, 1.0 mL/min).

Racemic

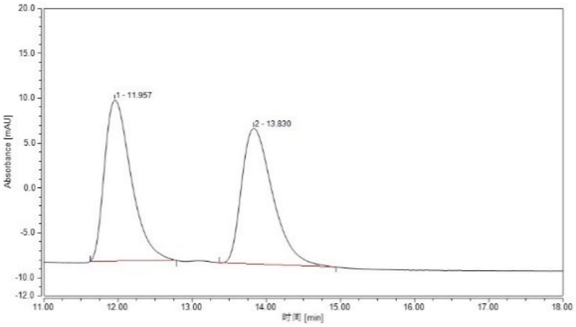

Chiral

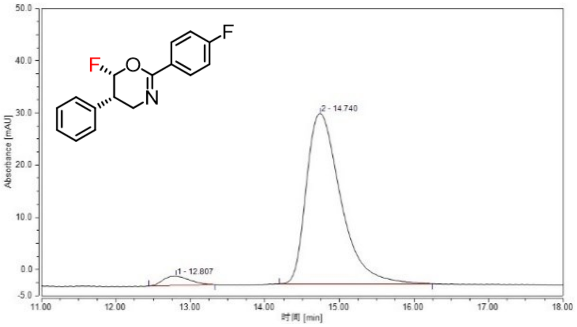

Racemic

Peak	RetTime (min)	Area (mAU*min)	Height (mAU)	Area%	Height%
1	11.957	7.501	17.935	50.49	54.29
2	13.830	7.356	15.100	49.51	45.71

Chiral

Peak	RetTime (min)	Area (mAU*min)	Height (mAU)	Area%	Height%
1	12.807	0.743	1.784	4.10	5.19
2	14.740	17.402	32.594	95.90	94.81

Supplementary Figure 7. HPLC Traces of 7b (phenomenex Lux Amylose-1, *i*-PrOH/Hexanes (V/V) = 99:1, 1.0 mL/min).

Racemic

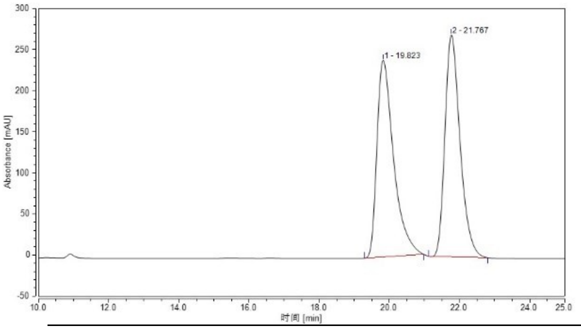

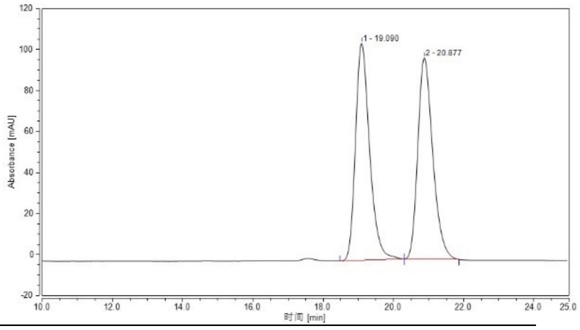

Peak	RetTime (min)	Area (mAU*min)	Height (mAU)	Area%	Height%
1	19.823	132.091	239.517	49.54	46.99
2	21.767	134.555	270.202	50.46	53.01

Peak	RetTime (min)	Area (mAU*min)	Height (mAU)	Area%	Height%
1	19.090	49.231	105.922	50.40	51.87
2	20.877	48.451	98.268	49.60	48.13

Chiral

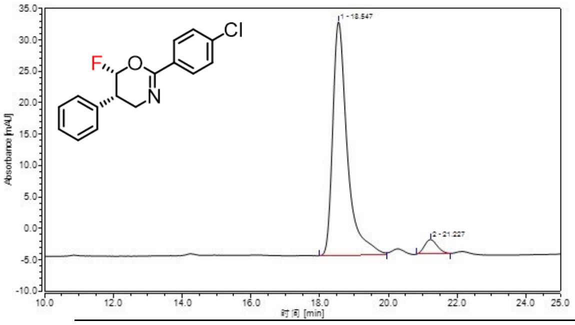

Peak	RetTime (min)	Area (mAU*min)	Height (mAU)	Area%	Height%
1	18.547	18.236	37.199	95.56	94.60
2	21.227	0.847	2.122	4.44	5.40

Supplementary Figure 8. HPLC Traces of 8b (IC, *i*-PrOH/Hexanes (V/V) = 99.5:0.5, 0.2 mL/min).

Racemic

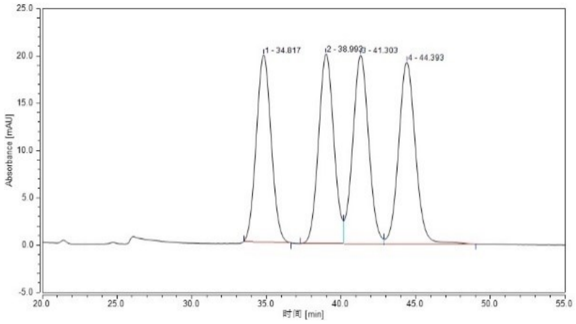

Chiral

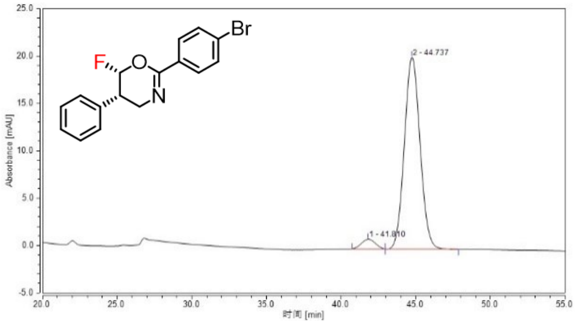

Racemic

Peak	RetTime (min)	Area (mAU*min)	Height (mAU)	Area%	Height%
1	34.817	22.776	19.767	23.73	25.04
2	38.993	23.707	20.051	24.70	25.40
3	41.303	24.476	19.912	25.50	25.23
4	44.393	25.033	19.201	26.08	24.33

Chiral

Peak	RetTime (min)	Area (mAU*min)	Height (mAU)	Area%	Height%
1	41.867	2.782	2.103	4.32	4.90
2	44.723	61.549	40.769	95.68	95.10

Supplementary Figure 9. HPLC Traces of 9b (AD, *i*-PrOH/Hexanes (V/V) = 99:1, 0.8 mL/min).

Racemic

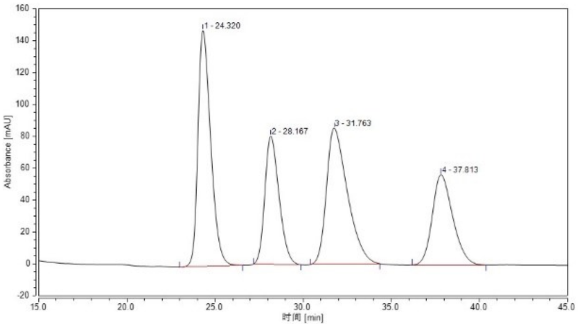

Chiral

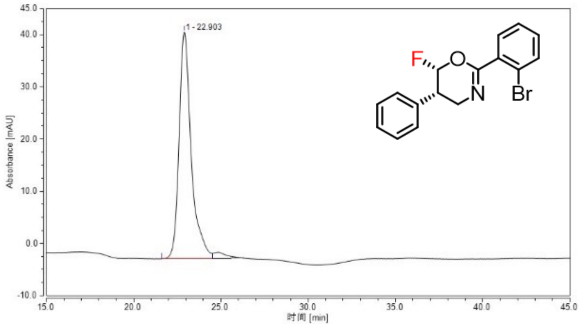

Racemic

Peak	RetTime (min)	Area (mAU*min)	Height (mAU)	Area%	Height%
1	24.320	124.135	147.931	31.59	39.98
2	28.167	74.996	80.302	19.09	21.70
3	31.763	117.592	85.263	29.93	23.04
4	37.813	76.203	56.550	19.39	15.28

Chiral

Peak	RetTime (min)	Area (mAU*min)	Height (mAU)	Area%	Height%
1	22.903	34.924	43.480	100.00	100.00

Supplementary Figure 10. HPLC Traces of 10b (phenomenex Cellulose-1, *i*-PrOH/Hexanes (V/V) = 99:1, 1.0 mL/min).

Racemic

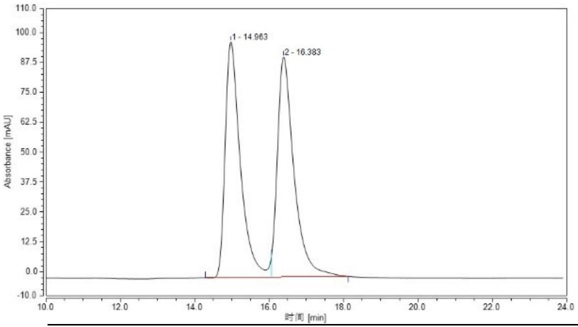

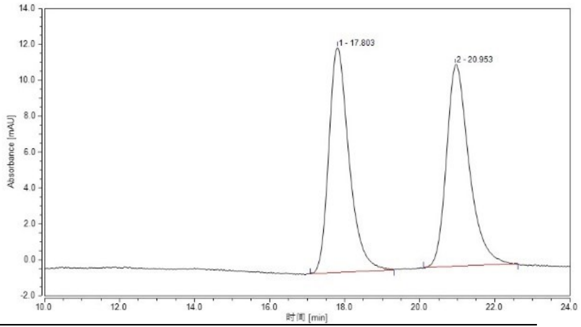

Peak	RetTime (min)	Area (mAU*min)	Height (mAU)	Area%	Height%
1	14.963	46.910	98.525	49.77	51.72
2	16.383	47.341	91.977	50.23	48.28

Peak	RetTime (min)	Area (mAU*min)	Height (mAU)	Area%	Height%
1	17.803	7.851	12.529	49.99	52.67
2	20.953	7.855	11.257	50.01	47.33

Chiral

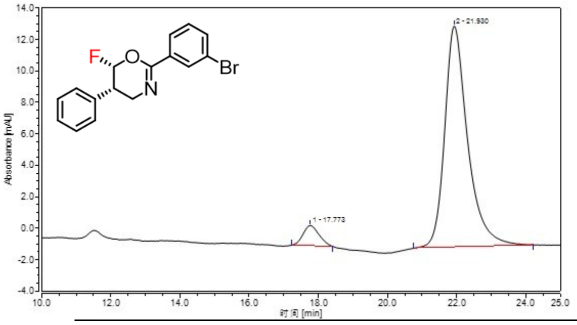

Peak	RetTime (min)	Area (mAU*min)	Height (mAU)	Area%	Height%
1	17.773	0.691	1.292	6.02	8.43
2	21.930	10.785	14.045	93.98	91.57

Supplementary Figure 11. HPLC Traces of 11b (AD, *i*-PrOH/Hexanes (V/V) = 97:3, 1.0 mL/min).

Racemic

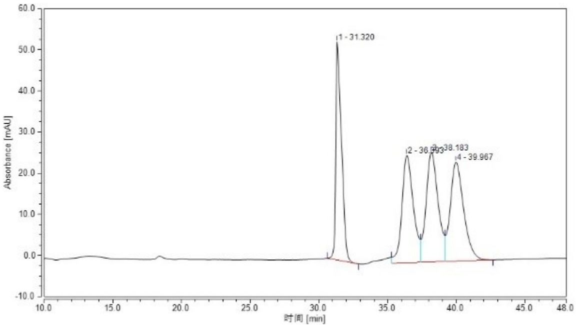

Chiral

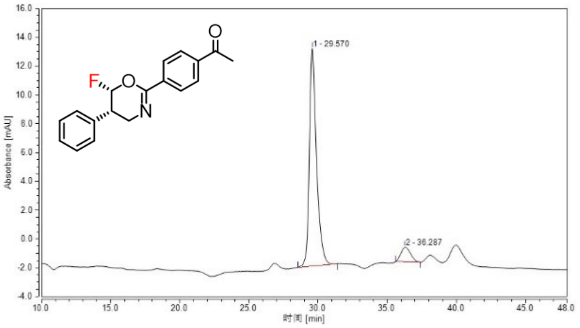

Racemic

Peak	RetTime (min)	Area (mAU*min)	Height (mAU)	Area%	Height%
1	31.320	25.237	53.032	23.99	40.82
2	36.393	25.249	26.132	24.01	20.11
3	38.183	27.217	26.665	25.88	20.52
4	31.320	25.237	53.032	23.99	40.82

Chiral

Peak	RetTime (min)	Area (mAU*min)	Height (mAU)	Area%	Height%
1	29.570	55.023	31.801	95.67	88.49
2	36.287	2.491	4.136	4.33	11.51

Supplementary Figure 12. HPLC Traces of 12b (phenomenex Lux Amylose-1, *i*-PrOH/Hexanes (V/V) = 85:15, 0.4 mL/min).

Racemic

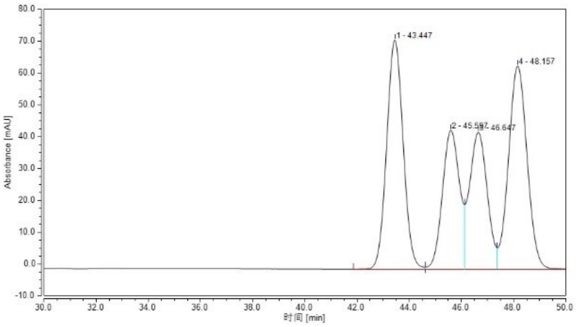

Chiral

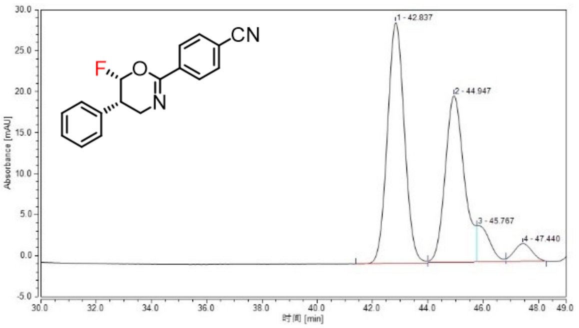

Racemic

Peak	RetTime (min)	Area (mAU*min)	Height (mAU)	Area%	Height%
1	43.447	52.718	71.924	30.56	32.34
2	45.597	33.151	43.631	19.22	19.62
3	46.647	33.928	42.987	19.67	19.33
4	48.157	52.693	63.838	30.55	28.71

Chiral

Peak	RetTime (min)	Area (mAU*min)	Height (mAU)	Area%	Height%
1	42.837	61.567	80.015	55.06	56.15
2	44.947	38.728	46.782	34.64	32.83
3	45.767	6.927	9.985	6.19	7.01
4	47.440	4.592	5.720	4.11	4.01

Supplementary Figure 13. HPLC Traces of 13b (phenomenex Lux Amylose-1, *i*-PrOH/Hexanes (V/V) = 99:1, 1.0 mL/min).

Racemic

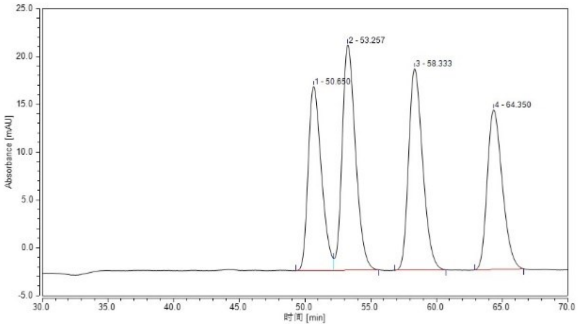

Chiral

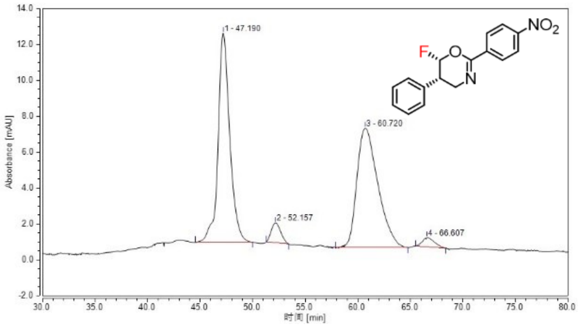

Racemic

Peak	RetTime (min)	Area (mAU*min)	Height (mAU)	Area%	Height%
1	50.650	22.508	19.261	22.85	23.90
2	53.257	27.731	23.563	28.15	29.24
3	58.333	26.104	21.056	26.50	26.13
4	64.350	22.177	16.700	22.51	20.72

Chiral

Peak	RetTime (min)	Area (mAU*min)	Height (mAU)	Area%	Height%
1	47.190	14.982	11.667	46.51	58.46
2	52.157	1.141	1.106	3.54	5.54
3	60.720	15.395	6.660	47.80	33.37
4	66.607	0.691	0.523	2.15	2.62

Supplementary Figure 14. HPLC Traces of 14b (phenomenex Cellulose-1, *i*-PrOH/Hexanes (V/V) = 98:2, 1.0 mL/min).

Racemic

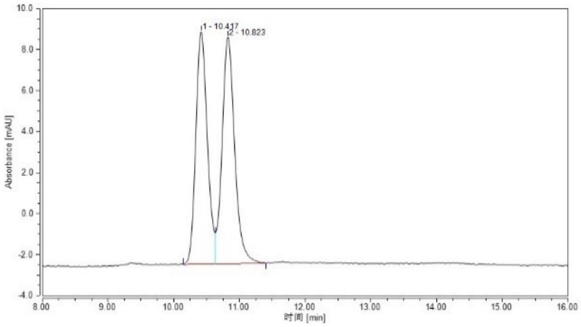

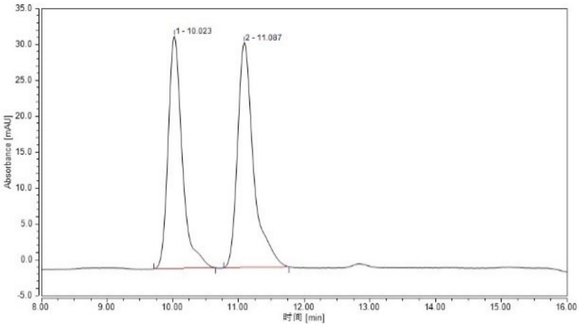

Peak	RetTime (min)	Area (mAU*min)	Height (mAU)	Area%	Height%
1	10.417	2.286	11.346	48.16	50.62
2	10.823	2.461	11.067	51.84	49.38

Peak	RetTime (min)	Area (mAU*min)	Height (mAU)	Area%	Height%
1	10.023	8.056	32.400	47.59	50.79
2	11.087	8.870	31.391	52.41	49.21

Chiral

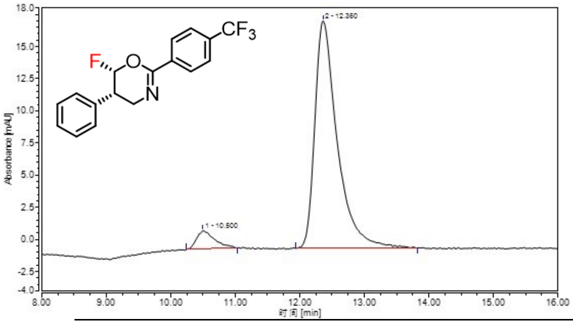

Peak	RetTime (min)	Area (mAU*min)	Height (mAU)	Area%	Height%
1	10.500	0.336	1.249	4.51	6.60
2	12.360	7.122	17.690	95.49	93.40

Supplementary Figure 15. HPLC Traces of 15b (phenomenex Cellulose-1, *i*-PrOH/Hexanes (V/V) = 98:2, 1.0 mL/min).

Racemic

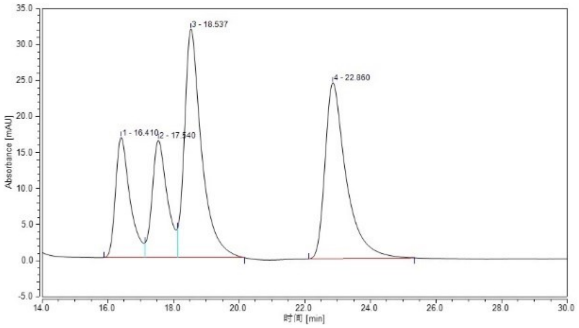

Chiral

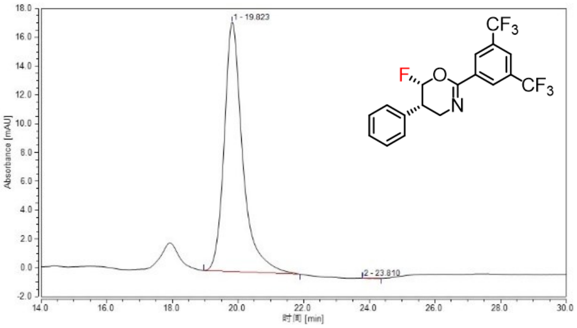

Racemic

Peak	RetTime (min)	Area (mAU*min)	Height (mAU)	Area%	Height%
1	16.410	8.563	16.709	15.68	18.73
2	17.540	8.659	16.313	15.85	18.28
3	18.537	19.199	31.757	35.14	35.59
4	22.860	18.209	24.444	33.33	27.40

Chiral

Peak	RetTime (min)	Area (mAU*min)	Height (mAU)	Area%	Height%
1	19.823	11.306	17.328	99.98	99.92
2	23.810	0.002	0.013	0.02	0.08

Supplementary Figure 16. HPLC Traces of 16b (phenomenex Lux Amylose-1, *i*-PrOH/Hexanes (V/V) = 98:2, 1.0 mL/min).

Racemic

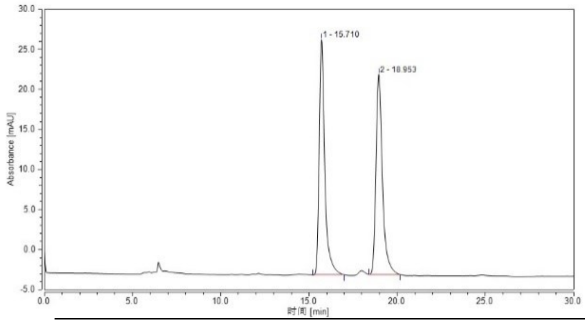

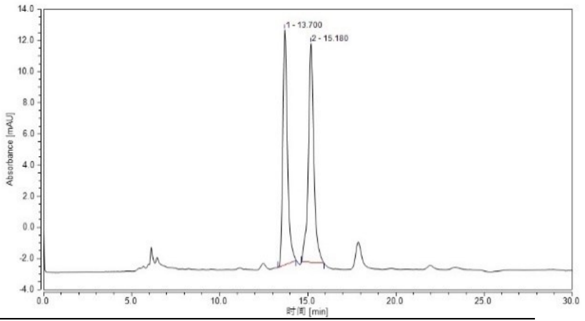

Peak	RetTime (min)	Area (mAU*min)	Height (mAU)	Area%	Height%
1	15.710	10.766	29.398	50.35	54.05
2	18.953	10.618	24.995	49.65	45.95

Peak	RetTime (min)	Area (mAU*min)	Height (mAU)	Area%	Height%
1	13.700	4.964	15.292	47.54	51.60
2	15.180	5.477	14.346	52.46	48.40

Chiral

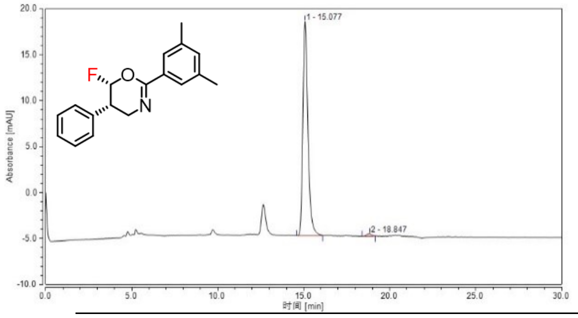

Peak	RetTime (min)	Area (mAU*min)	Height (mAU)	Area%	Height%
1	15.077	7.948	23.319	98.88	98.91
2	18.847	0.090	0.256	1.12	1.09

Supplementary Figure 17. HPLC Traces of 17b (phenomenex Lux Amylose-1, *i*-PrOH/Hexanes (V/V) = 98:2, 1.0 mL/min).

Racemic

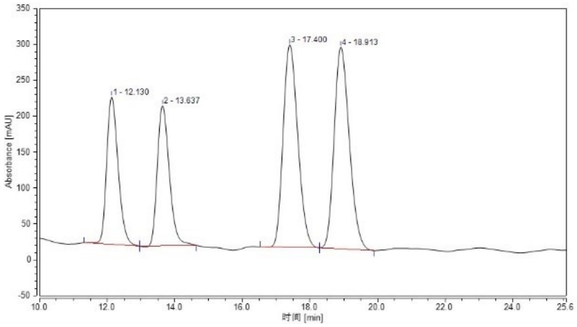

Chiral

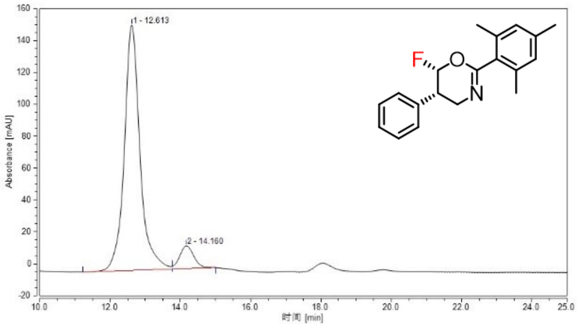

Racemic

Peak	RetTime (min)	Area (mAU*min)	Height (mAU)	Area%	Height%
1	12.130	80.827	205.376	18.03	21.30
2	13.637	80.021	195.373	17.85	20.26
3	17.400	141.577	282.298	31.58	29.28
4	18.913	145.854	281.208	32.54	29.16

Chiral

Peak	RetTime (min)	Area (mAU*min)	Height (mAU)	Area%	Height%
1	12.613	79.518	153.849	92.28	91.51
2	14.160	6.652	14.269	7.72	8.49

Supplementary Figure 18. HPLC Traces of 18b (phenomenex Lux Amylose-1, *i*-PrOH/Hexanes (V/V) = 98:2, 1.0 mL/min).

Racemic

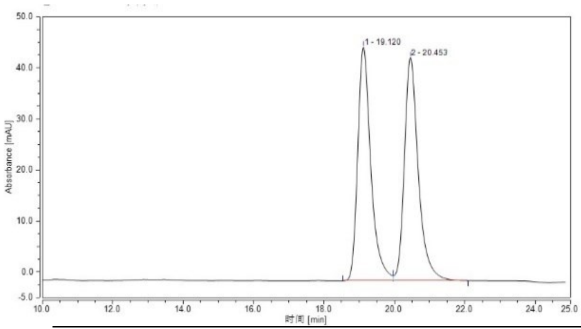

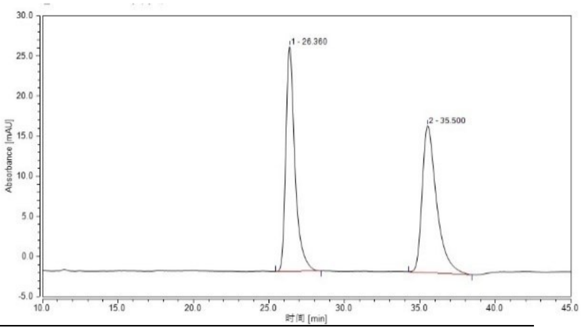

Peak	RetTime (min)	Area (mAU*min)	Height (mAU)	Area%	Height%
1	19.120	19.780	45.806	49.59	51.14
2	20.453	20.106	43.768	50.41	48.86

Peak	RetTime (min)	Area (mAU*min)	Height (mAU)	Area%	Height%
1	26.360	19.667	28.032	49.13	60.47
2	35.500	20.361	18.329	50.87	39.53

Chiral

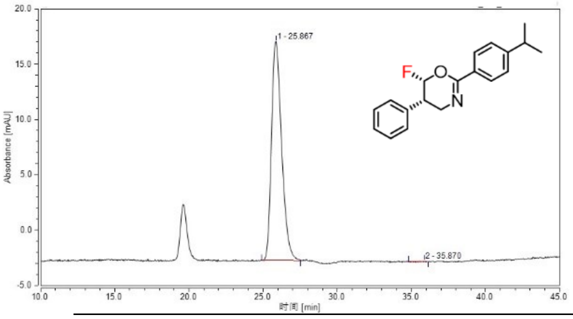

Peak	RetTime (min)	Area (mAU*min)	Height (mAU)	Area%	Height%
1	25.867	15.648	19.800	99.96	99.54
2	35.870	0.006	0.091	0.04	0.46

Supplementary Figure 19. HPLC Traces of 19b (phenomenex Cellulose-1, *i*-PrOH/Hexanes (V/V) = 98:2, 1.0 mL/min).

Racemic

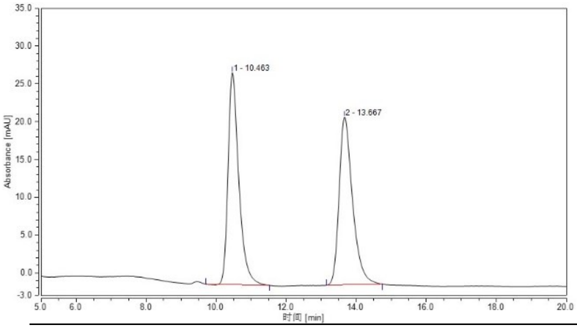

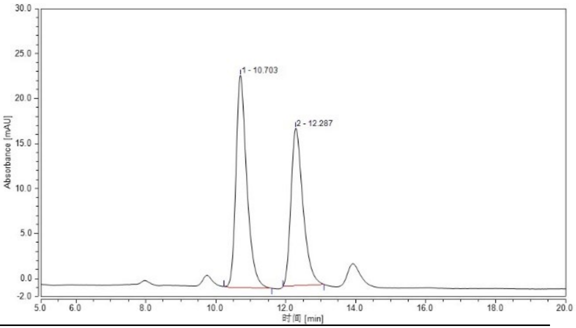

Peak	RetTime (min)	Area (mAU*min)	Height (mAU)	Area%	Height%
1	10.463	9.972	28.065	50.32	55.85
2	13.667	9.844	22.189	49.68	44.15

Peak	RetTime (min)	Area (mAU*min)	Height (mAU)	Area%	Height%
1	10.703	8.469	23.578	54.85	57.36
2	12.287	6.971	17.531	45.15	42.64

Chiral

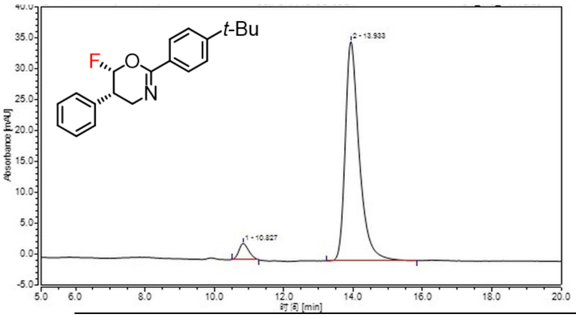

Peak	RetTime (min)	Area (mAU*min)	Height (mAU)	Area%	Height%
1	10.827	0.850	2.581	4.89	6.79
2	13.933	16.549	35.448	95.11	93.21

Supplementary Figure 20. HPLC Traces of 20b (phenomenex Cellulose-1, *i*-PrOH/Hexanes (V/V) = 99:1, 1.0 mL/min).

Racemic

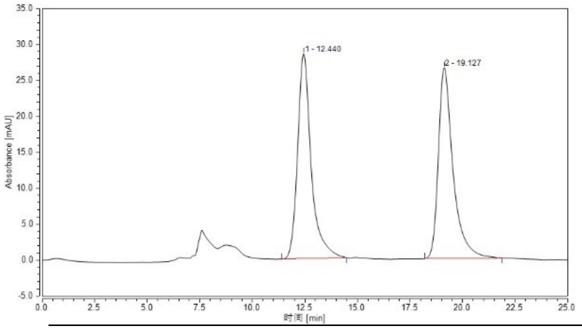

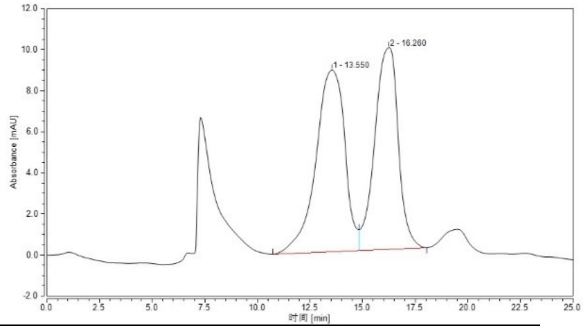

Peak	RetTime (min)	Area (mAU*min)	Height (mAU)	Area%	Height%
1	12.440	21.313	28.512	49.78	51.79
2	19.127	21.499	26.539	50.22	48.21

Peak	RetTime (min)	Area (mAU*min)	Height (mAU)	Area%	Height%
1	13.550	14.204	8.863	52.73	47.42
2	16.260	12.735	9.826	47.27	52.58

Chiral

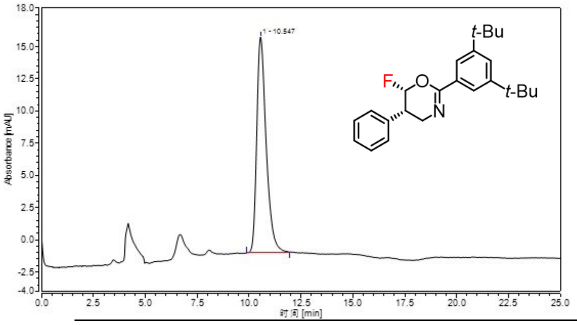

Peak	RetTime (min)	Area (mAU*min)	Height (mAU)	Area%	Height%
1	10.547	8.801	16.755	100.00	100.00

Supplementary Figure 21. HPLC Traces of 21b (IC, *i*-PrOH/Hexanes (V/V) = 92:8, 1.0 mL/min).

Racemic

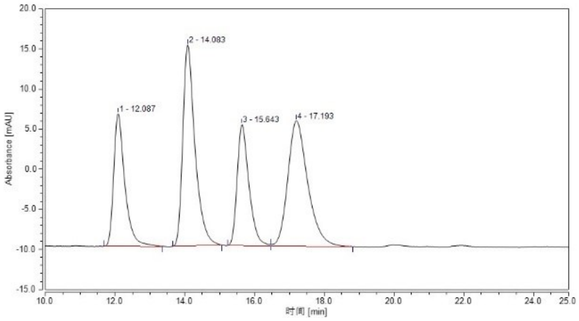

Chiral

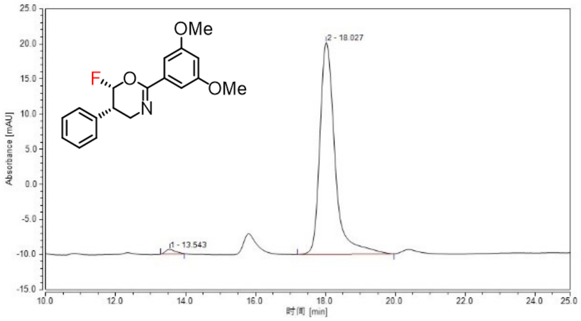

Racemic

Peak	RetTime (min)	Area (mAU*min)	Height (mAU)	Area%	Height%
1	12.087	6.111	16.520	18.79	22.84
2	14.083	10.127	25.063	31.13	34.65
3	15.643	5.914	15.098	18.18	20.87

4	17.193	10.377	15.648	31.90	21.63

Chiral

Peak	RetTime (min)	Area (mAU*min)	Height (mAU)	Area%	Height%
1	13.543	0.220	0.655	1.42	2.12
2	18.027	15.215	30.217	98.58	97.88

Supplementary Figure 22. HPLC Traces of 22b (phenomenex Cellulose-1, *i*-PrOH/Hexanes (V/V) = 85:15, 0.8 mL/min).

Racemic

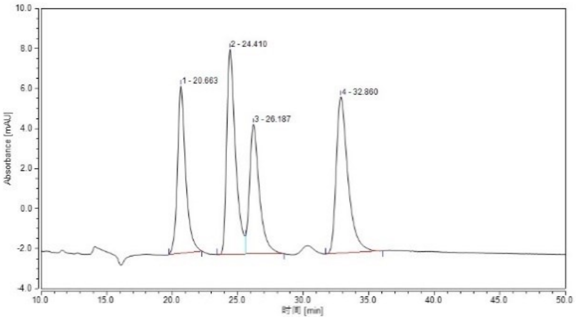

Chiral

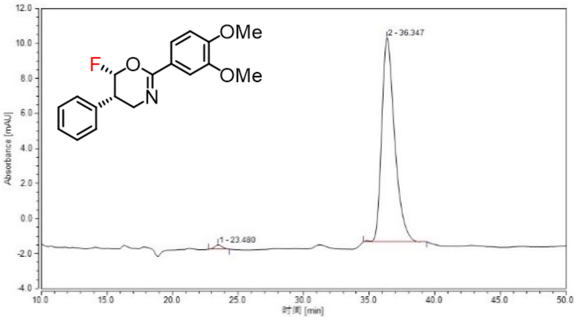

Racemic

Peak	RetTime (min)	Area (mAU*min)	Height (mAU)	Area%	Height%
1	20.663	6.034	8.375	21.80	25.44
2	24.410	7.912	10.242	28.59	31.11
3	26.187	5.628	6.484	20.34	19.69
4	32.860	8.099	7.824	29.27	23.76

Chiral

Peak	RetTime (min)	Area (mAU*min)	Height (mAU)	Area%	Height%
1	23.480	0.162	0.245	1.21	2.06
2	36.347	13.291	11.665	98.79	97.94

Supplementary Figure 23. HPLC Traces of 23b (AD, *i*-PrOH/Hexanes (V/V) = 97:3, 1.0 mL/min).

Racemic

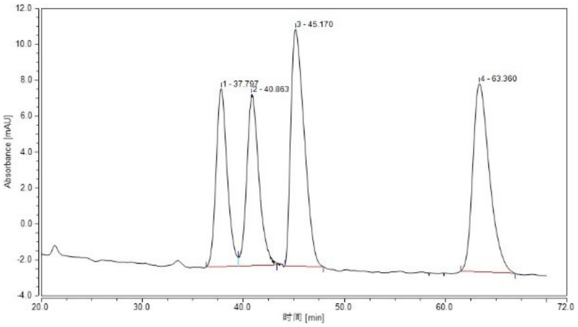

Chiral

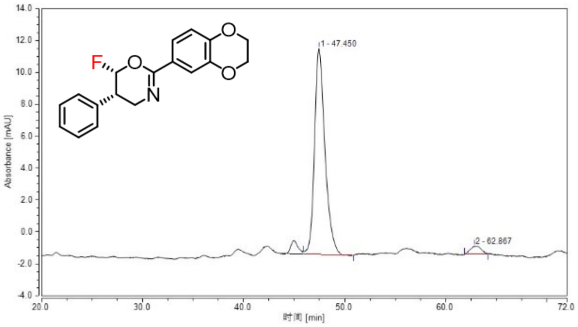

Racemic

Peak	RetTime (min)	Area (mAU*min)	Height (mAU)	Area%	Height%
1	37.797	12.626	9.918	19.06	22.97
2	40.863	13.272	9.555	20.03	22.13
3	45.170	20.286	13.203	30.62	30.58
4	63.360	20.066	10.507	30.29	24.33

Chiral

Peak	RetTime (min)	Area (mAU*min)	Height (mAU)	Area%	Height%
1	47.450	15.449	12.926	96.32	96.19
2	62.867	0.590	0.512	3.68	3.81

Supplementary Figure 24. HPLC Traces of 24b (phenomenex Lux Amylose-1, *i*-PrOH/Hexanes (V/V) = 98:2, 1.0 mL/min).

Racemic

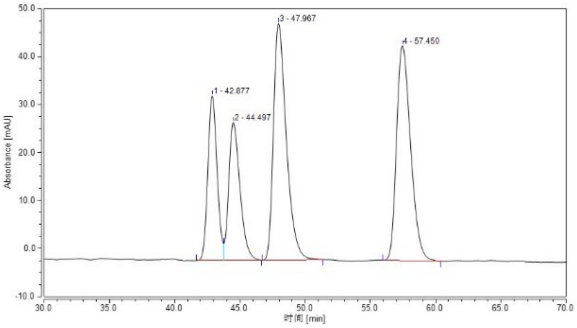

Chiral

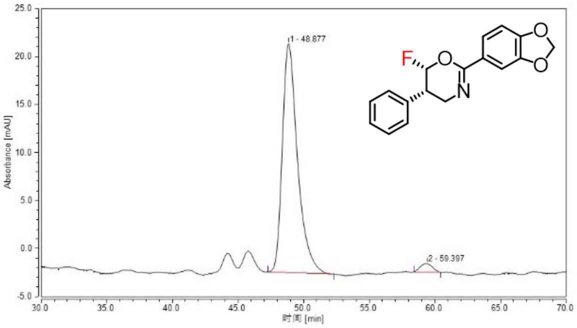

Racemic

Peak	RetTime (min)	Area (mAU*min)	Height (mAU)	Area%	Height%
1	42.877	28.206	34.311	16.64	21.83
2	44.497	28.737	28.782	16.95	18.31
3	47.967	56.532	49.335	33.34	31.39
4	57.450	56.079	44.747	33.07	28.47

Chiral

Peak	RetTime (min)	Area (mAU*min)	Height (mAU)	Area%	Height%
1	48.877	31.989	23.846	97.16	96.32
2	59.397	0.935	0.911	2.84	3.68

Supplementary Figure 25. HPLC Traces of 25b (phenomenex Lux Amylose-1, *i*-PrOH/Hexanes (V/V) = 98:2, 1.0 mL/min)

Racemic

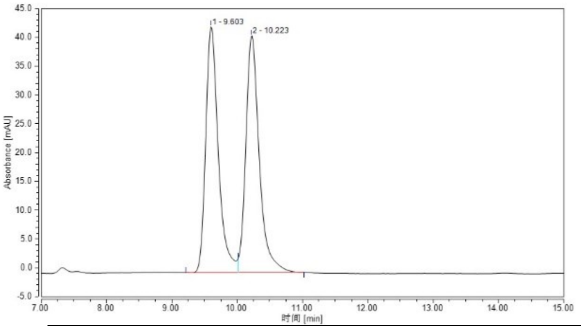

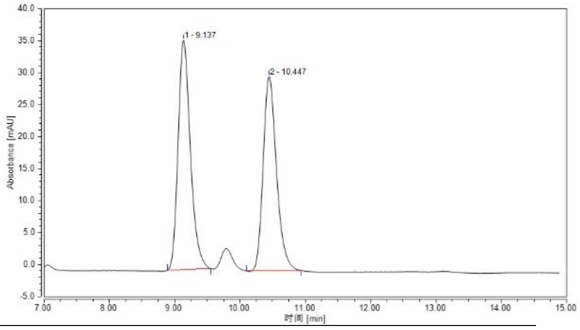

Peak	RetTime (min)	Area (mAU*min)	Height (mAU)	Area%	Height%
1	9.603	9.552	42.690	49.44	50.93
2	10.223	9.769	41.128	50.56	49.07

Peak	RetTime (min)	Area (mAU*min)	Height (mAU)	Area%	Height%
1	9.137	7.811	35.681	52.15	54.17
2	10.447	7.166	30.362	47.85	45.83

Chiral

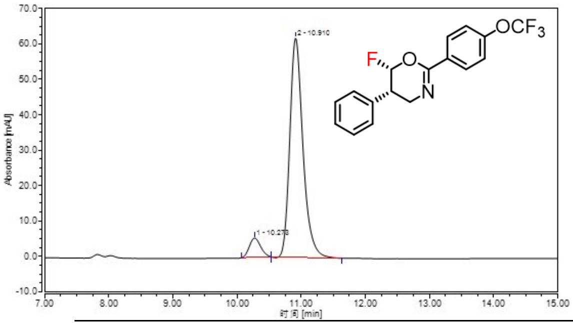

Peak	RetTime (min)	Area (mAU*min)	Height (mAU)	Area%	Height%
1	10.273	1.129	5.497	7.13	8.14
2	10.910	14.707	62.011	92.87	91.86

Supplementary Figure 26. HPLC Traces of 26b (IC, *i*-PrOH/Hexanes (V/V) = 98:2, 1.0 mL/min).

Racemic

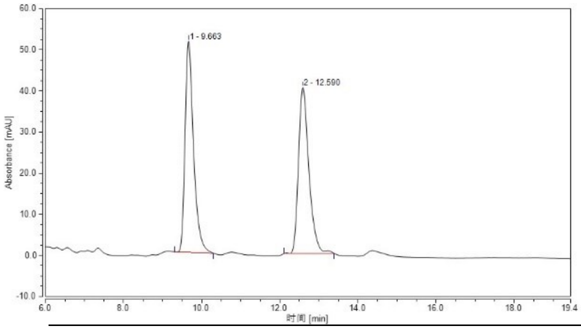

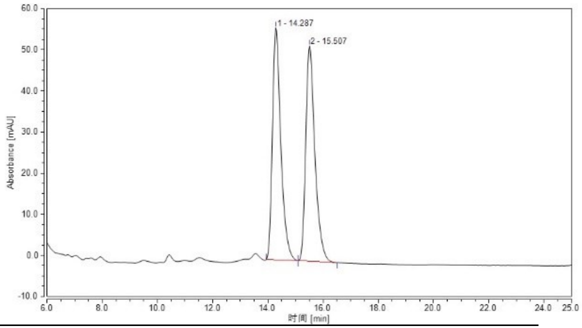

Peak	RetTime (min)	Area (mAU*min)	Height (mAU)	Area%	Height%
1	9.663	12.857	51.329	50.79	55.95
2	12.590	12.456	40.413	49.21	44.05

Peak	RetTime (min)	Area (mAU*min)	Height (mAU)	Area%	Height%
1	14.287	19.834	56.457	49.65	51.86
2	15.507	20.111	52.408	50.35	48.14

Chiral

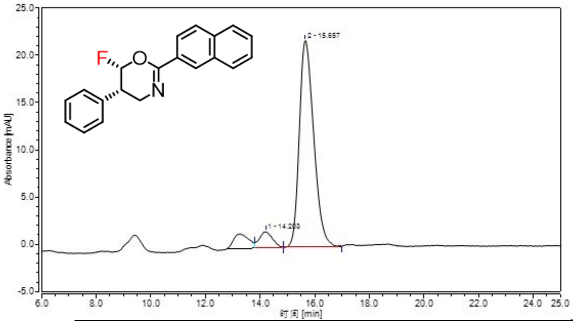

Peak	RetTime (min)	Area (mAU*min)	Height (mAU)	Area%	Height%
1	14.203	0.940	1.725	6.61	7.32
2	15.657	13.290	21.837	93.39	92.68

Supplementary Figure 27. HPLC Traces of 27b (phenomenex Cellulose-1, *i*-PrOH/Hexanes (V/V) = 95:5, 1.0 mL/min).

Racemic

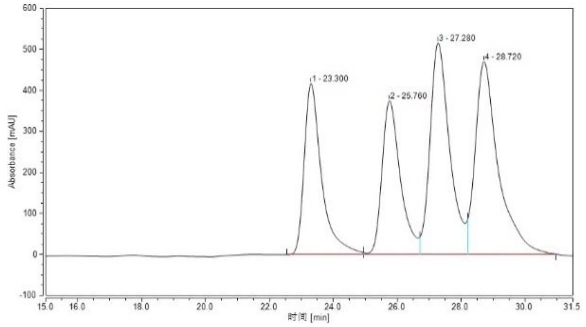

Chiral

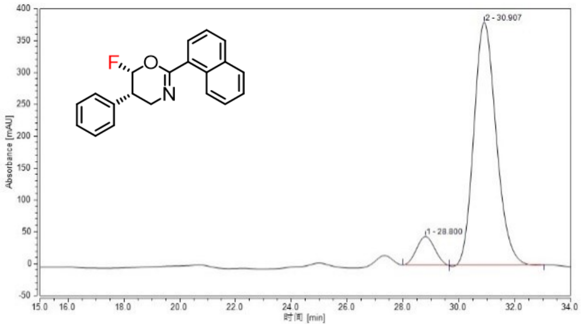

Racemic

Peak	RetTime (min)	Area (mAU*min)	Height (mAU)	Area%	Height%
1	23.300	264.374	417.070	20.73	23.46
2	25.760	250.850	374.641	19.67	21.08
3	27.280	368.707	515.804	28.91	29.02
4	28.720	391.479	470.038	30.69	26.44

Chiral

Peak	RetTime (min)	Area (mAU*min)	Height (mAU)	Area%	Height%
1	28.800	27.384	40.197	7.17	9.53
2	30.907	354.388	381.422	92.83	90.47

Supplementary Figure 28. HPLC Traces of 28b (phenomenex Lux Amylose-1, *i*-PrOH/Hexanes (V/V) = 90:10, 1.0 mL/min).

Racemic

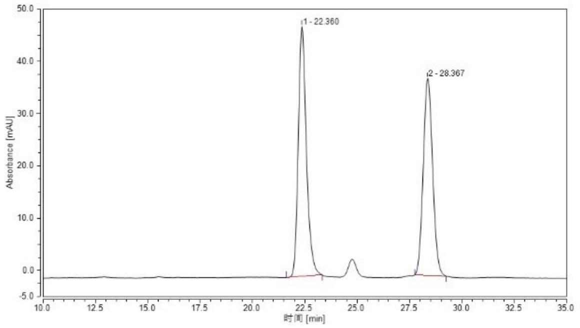

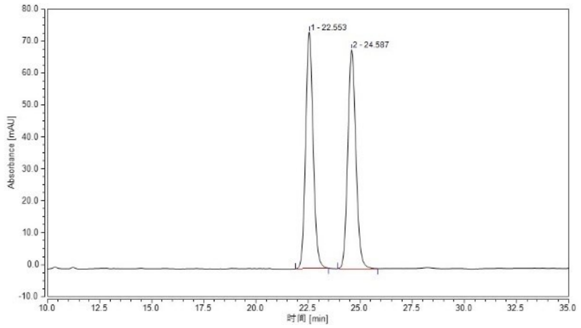

Peak	RetTime (min)	Area (mAU*min)	Height (mAU)	Area%	Height%
1	22.360	21.431	47.845	52.26	55.91
2	28.367	19.579	37.727	47.74	44.09

Peak	RetTime (min)	Area (mAU*min)	Height (mAU)	Area%	Height%
1	22.553	30.927	74.013	50.02	51.92
2	24.587	30.904	68.539	49.98	48.08

Chiral

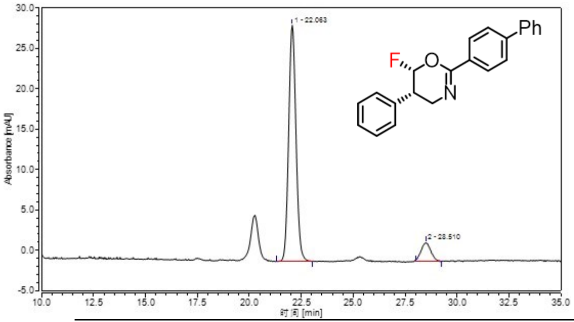

Peak	RetTime (min)	Area (mAU*min)	Height (mAU)	Area%	Height%
1	22.063	11.948	29.248	90.67	92.71
2	28.510	1.229	2.301	9.33	7.29

Supplementary Figure 29. HPLC Traces of 29b (IC, *i*-PrOH/Hexanes (V/V) = 95:5, 1.0 mL/min).

Racemic

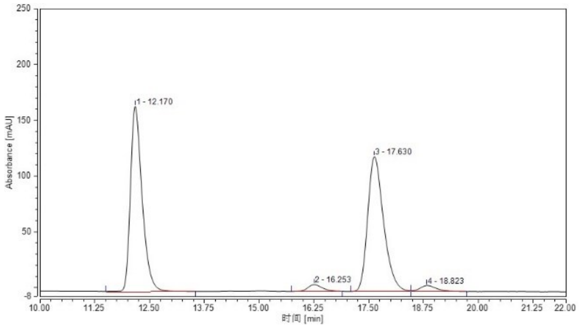

Chiral

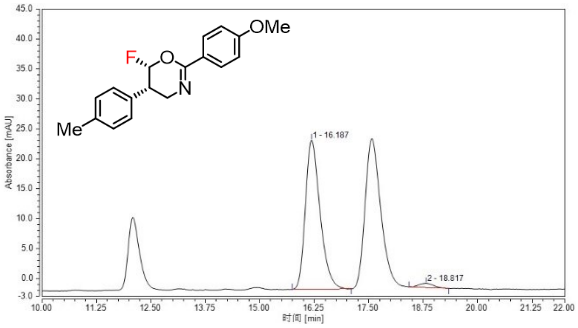

Racemic

Peak	RetTime (min)	Area (mAU*min)	Height (mAU)	Area%	Height%
1	12.170	51.738	166.188	48.17	55.65
2	16.253	2.364	6.118	2.20	2.05
3	17.630	50.872	120.915	47.37	40.49
4	18.823	2.429	5.386	2.26	1.80

Chiral

Peak	RetTime (min)	Area (mAU*min)	Height (mAU)	Area%	Height%
1	16.187	9.734	24.925	97.21	97.12
2	18.817	0.280	0.739	2.79	2.88

Supplementary Figure 30. HPLC Traces of 32b (phenomenex Lux Amylose-1, *i*-PrOH/Hexanes (V/V) = 98:2, 1.0 mL/min)

Racemic

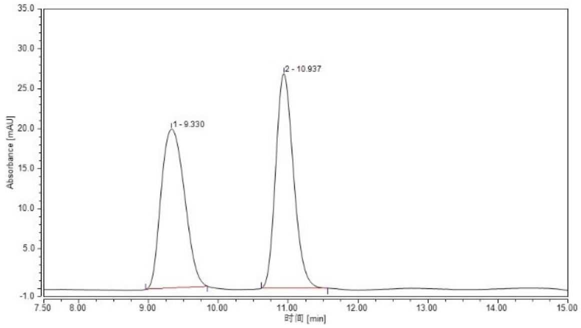

Chiral

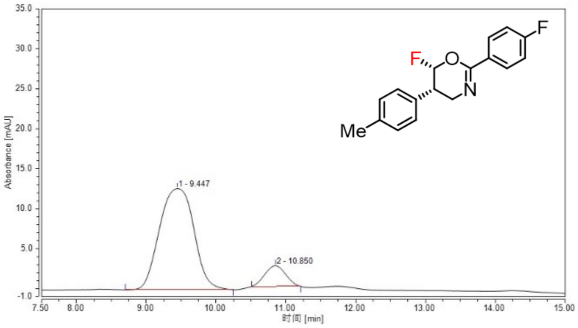

Racemic

Peak	RetTime (min)	Area (mAU*min)	Height (mAU)	Area%	Height%
1	9.330	7.706	19.863	49.38	42.55
2	10.937	7.898	26.813	50.62	57.45

Chiral

Peak	RetTime (min)	Area (mAU*min)	Height (mAU)	Area%	Height%
1	9.447	7.231	12.633	89.96	83.52
2	10.850	0.896	2.584	10.04	16.48

Supplementary Figure 31. HPLC Traces of 33b (phenomenex Lux Amylose-1, *i*-PrOH/Hexanes (V/V) = 98:2, 1.0 mL/min).

Racemic

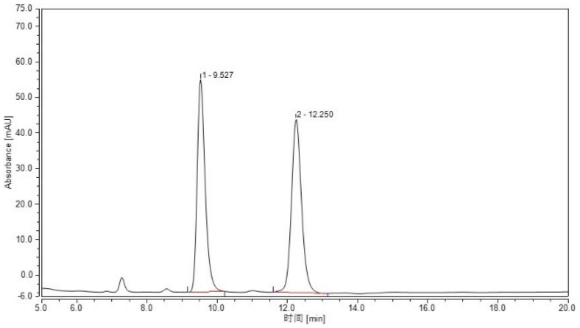

Chiral

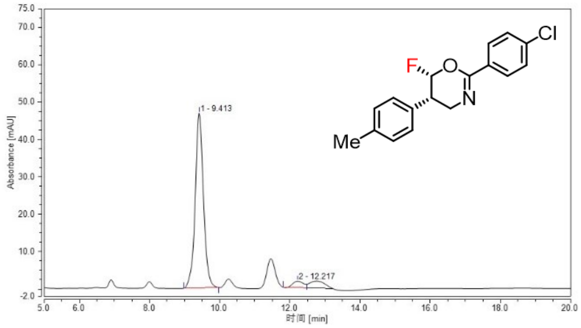

Racemic

Peak	RetTime (min)	Area (mAU*min)	Height (mAU)	Area%	Height%
1	9.527	15.753	59.716	49.33	55.04
2	12.250	16.182	48.780	50.67	44.96

Chiral

Peak	RetTime (min)	Area (mAU*min)	Height (mAU)	Area%	Height%
1	9.413	12.720	46.765	95.46	96.49
2	12.217	0.605	1.703	4.54	3.51

Supplementary Figure 32. HPLC Traces of 34b (phenomenex Lux Amylose-1, *i*-PrOH/Hexanes (V/V) = 98:2, 1.0 mL/min).

Racemic

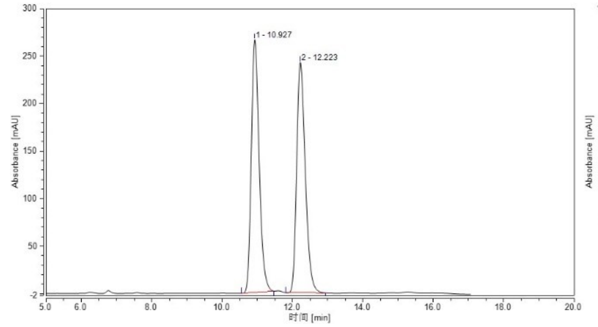

Chiral

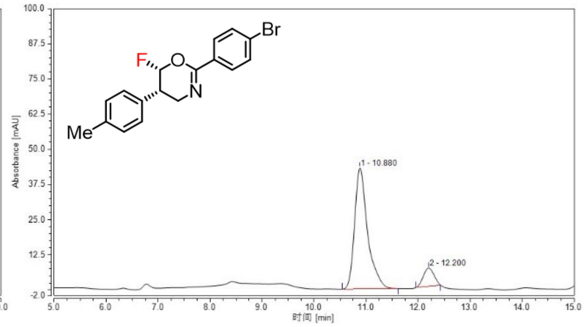

Racemic

Peak	RetTime (min)	Area (mAU*min)	Height (mAU)	Area%	Height%
1	10.927	68.743	265.844	49.95	52.30
2	12.223	68.874	242.427	50.05	47.70

Chiral

Peak	RetTime (min)	Area (mAU*min)	Height (mAU)	Area%	Height%
1	10.880	12.403	42.965	90.92	87.86
2	12.200	1.550	6.501	9.08	12.14

Supplementary Figure 33. HPLC Traces of 35b (phenomenex Lux Amylose-1, *i*-PrOH/Hexanes (V/V) = 98:2, 1.0 mL/min).

Racemic

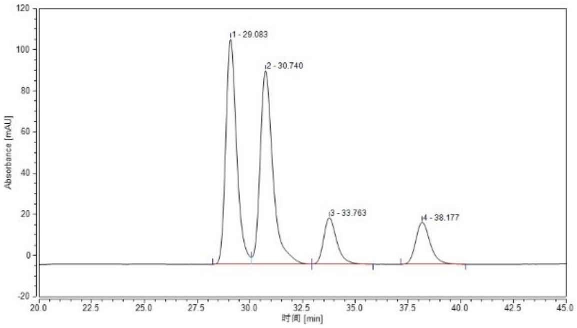

Chiral

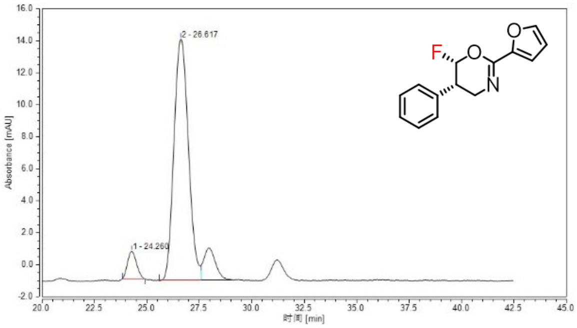

Racemic

Peak	RetTime (min)	Area (mAU*min)	Height (mAU)	Area%	Height%
1	29.083	65.410	109.465	39.74	44.37
2	30.740	66.520	94.128	40.41	38.16
3	33.763	16.336	22.615	9.92	9.17
4	38.177	16.334	20.490	9.92	8.31

Chiral

Peak	RetTime (min)	Area (mAU*min)	Height (mAU)	Area%	Height%
1	23.630	0.415	0.902	6.13	7.86
2	25.273	6.355	10.567	93.87	92.14

Supplementary Figure 34. HPLC Traces of 36b (phenomenex Lux Amylose-1, *i*-PrOH/Hexanes (V/V) = 95:5, 1.0 mL/min).

Racemic Chiral

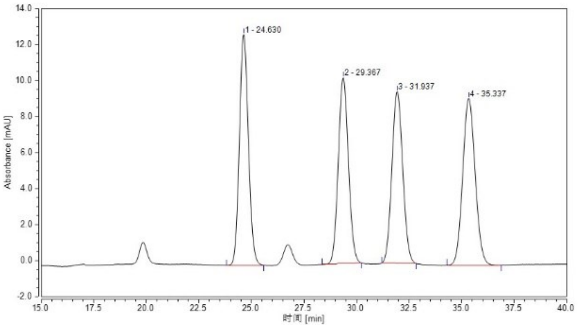

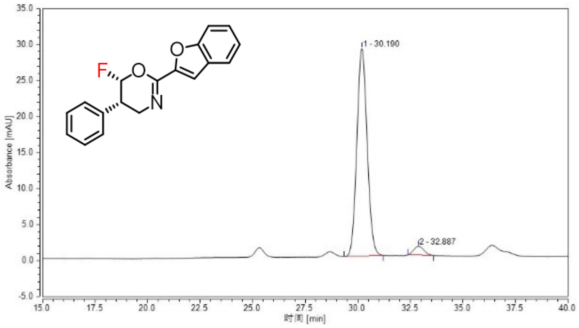

Racemic

Peak	RetTime (min)	Area (mAU*min)	Height (mAU)	Area%	Height%
1	24.630	6.349	12.823	26.28	30.56
2	29.367	5.767	10.336	23.87	24.63
3	31.937	5.689	9.537	23.55	22.73
4	35.337	6.353	9.268	26.30	22.09

Chiral

Peak	RetTime (min)	Area (mAU*min)	Height (mAU)	Area%	Height%
1	30.190	16.305	28.826	95.95	95.91

2	32.887	0.688	1.230	4.05	4.09

Supplementary Figure 35. HPLC Traces of 37b (phenomenex Lux Amylose-1, *i*-PrOH/Hexanes (V/V) = 99:1, 0.5 mL/min).

Racemic Chiral

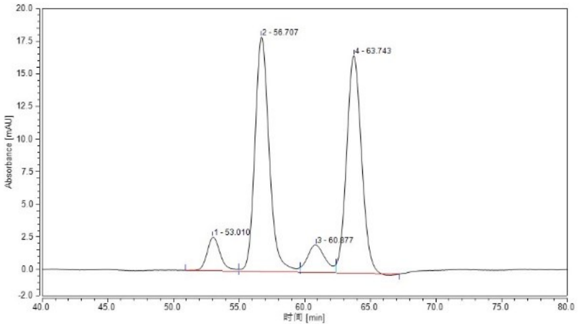

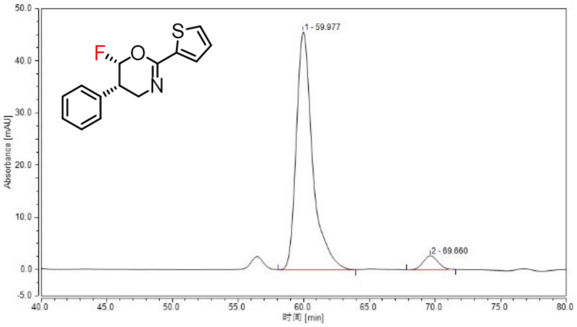

Racemic

Peak	RetTime (min)	Area (mAU*min)	Height (mAU)	Area%	Height%
1	53.010	3.029	2.581	5.95	6.56
2	56.707	22.711	17.980	44.61	45.69
3	60.877	3.299	2.127	6.48	5.41
4	63.743	21.872	16.665	42.96	42.35

Chiral

Peak	RetTime (min)	Area (mAU*min)	Height (mAU)	Area%	Height%
1	59.977	65.625	45.560	95.91	95.03
2	69.660	2.800	2.381	4.09	4.97

Supplementary Figure 36. HPLC Traces of 38b (phenomenex Cellulose-1, *i*-PrOH/Hexanes (V/V) = 90:10, 1.0 mL/min).

Racemic

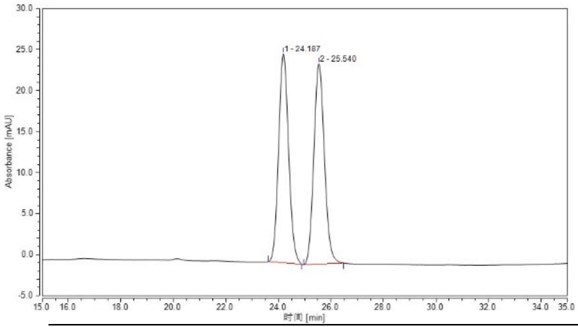

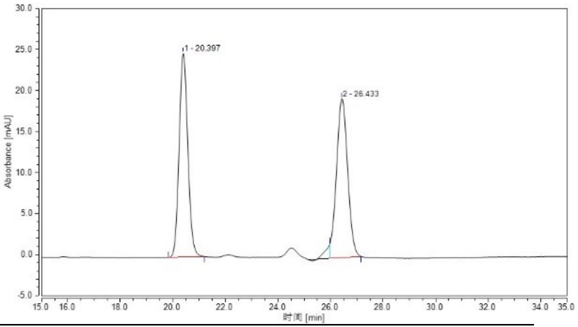

Peak	RetTime (min)	Area (mAU*min)	Height (mAU)	Area%	Height%
1	24.187	11.267	25.520	49.70	51.07
2	25.540	11.401	24.451	50.30	48.93

Peak	RetTime (min)	Area (mAU*min)	Height (mAU)	Area%	Height%
1	20.397	9.474	24.837	49.83	56.14
2	26.433	9.536	19.402	50.17	43.86

Chiral

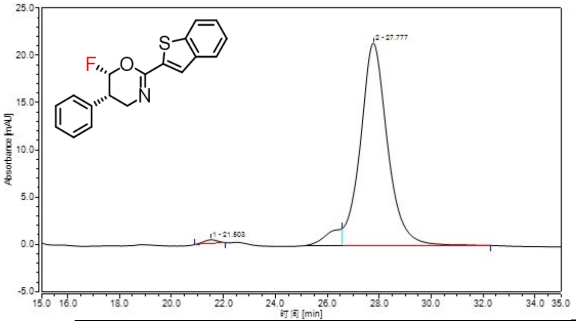

Peak	RetTime (min)	Area (mAU*min)	Height (mAU)	Area%	Height%
1	21.503	0.243	0.400	0.94	1.84
2	27.777	25.792	21.397	99.06	98.16

Supplementary Figure 37. HPLC Traces of 39b (phenomenex Lux Amylose-1, *i*-PrOH/Hexanes (V/V) = 98.7:1.3, 1.0 mL/min).

Racemic

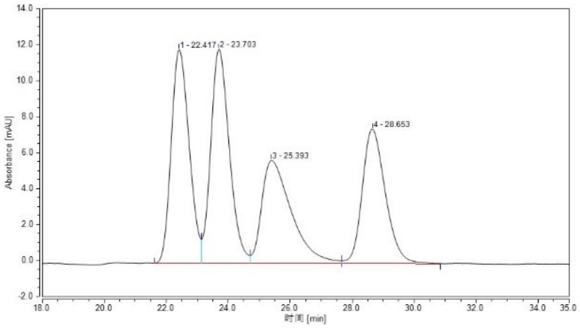

Chiral

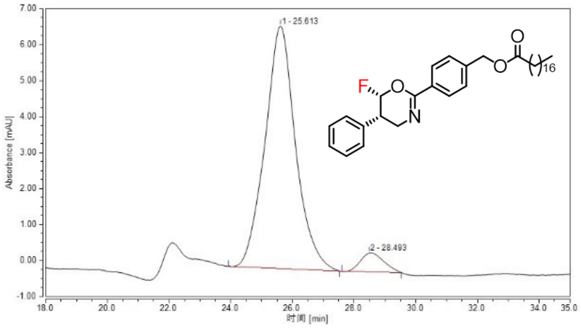

Racemic

Peak	RetTime (min)	Area (mAU*min)	Height (mAU)	Area%	Height%
1	22.417	7.934	11.868	27.32	32.09
2	23.703	8.261	11.902	28.45	32.18
3	25.393	6.360	5.732	21.90	15.50
4	28.653	6.483	7.481	22.33	20.23

Chiral

Peak	RetTime (min)	Area (mAU*min)	Height (mAU)	Area%	Height%
1	25.613	7.737	6.741	94.39	92.66
2	28.493	0.460	0.534	5.61	7.34

Supplementary Figure 38. HPLC Traces of 40b (phenomenex Lux Amylose-1, *i*-PrOH/Hexanes (V/V) = 88:12, 1.0 mL/min).

Racemic

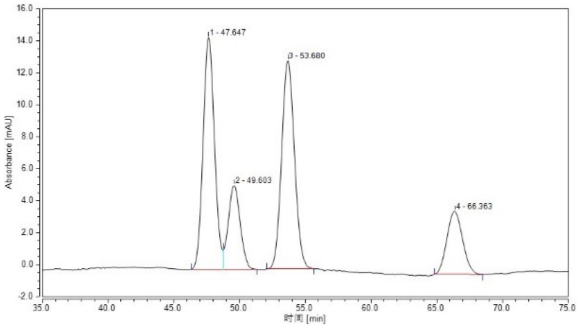

Chiral

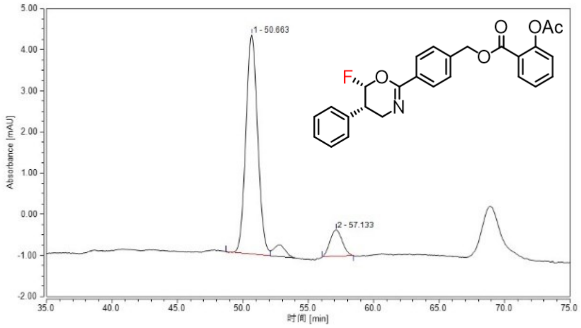

Racemic

Peak	RetTime (min)	Area (mAU*min)	Height (mAU)	Area%	Height%
1	47.647	14.653	14.506	36.34	39.47
2	49.603	5.482	5.263	13.59	14.32
3	53.680	14.739	13.041	36.55	35.49
4	66.363	5.449	3.940	13.51	10.72

Chiral

Peak	RetTime (min)	Area (mAU*min)	Height (mAU)	Area%	Height%
1	50.663	1.534	1.621	9.58	11.30
2	57.133	14.106	12.228	90.42	88.70

Supplementary Figure 39. HPLC Traces of 41b (phenomenex Lux Amylose-1, *i*-PrOH/Hexanes (V/V) = 75:25, 0.8 mL/min).

Racemic

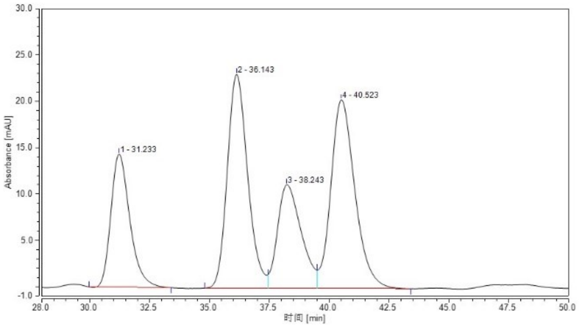

Chiral

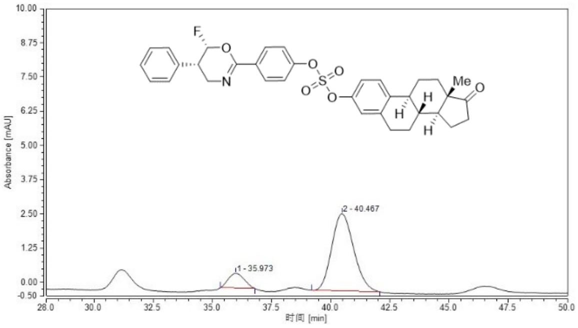

Racemic

Peak	RetTime (min)	Area (mAU*min)	Height (mAU)	Area%	Height%
1	31.233	13.139	14.353	18.17	20.83
2	36.143	22.714	23.045	31.41	33.44
3	38.243	12.814	11.180	17.72	16.22
4	40.523	23.639	20.344	32.69	29.52

Chiral

Peak	RetTime (min)	Area (mAU*min)	Height (mAU)	Area%	Height%
1	35.973	0.417	0.537	10.09	14.01
2	40.467	3.031	2.816	89.91	85.99

Supplementary Figure 40. HPLC Traces of 42b (AD, *i*-PrOH/Hexanes (V/V) = 80:20, 1.0 mL/min).

Racemic

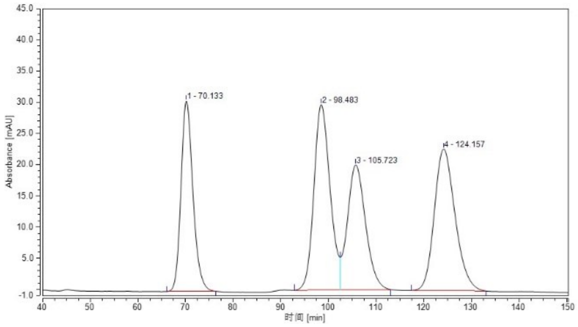

Chiral

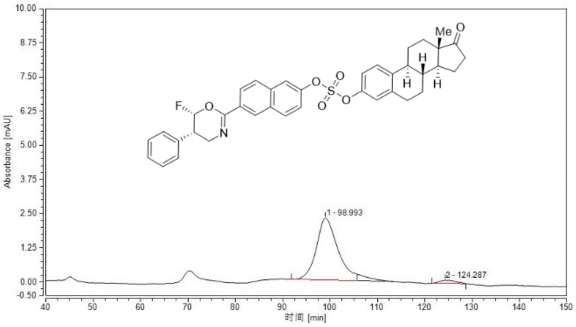

Racemic

Peak	RetTime (min)	Area (mAU*min)	Height (mAU)	Area%	Height%
1	70.133	88.900	30.544	21.71	29.64
2	98.483	117.136	29.753	28.61	28.87
3	105.723	88.582	20.043	21.63	19.45
4	124.157	114.842	22.708	28.05	22.04

Chiral

Peak	RetTime (min)	Area (mAU*min)	Height (mAU)	Area%	Height%
1	98.993	11.815	2.280	96.09	94.71
2	124.287	0.481	0.127	3.91	5.29

6.2 HPLC Traces of 43b-54b

Supplementary Figure 41. HPLC Traces of 43b (phenomenex Cellulose-1, *i*-PrOH/Hexanes (V/V) = 95:5, 1.0 mL/min).

Racemic

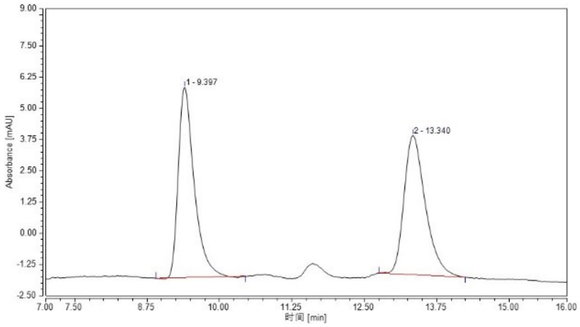

Chiral

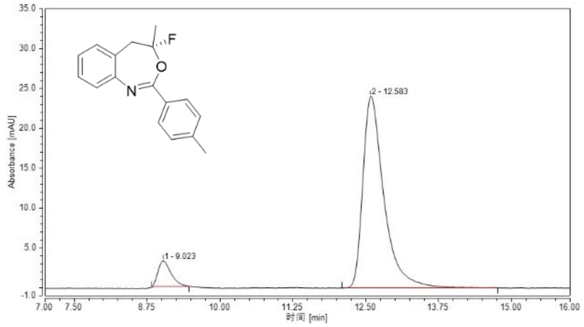

Racemic

Peak	RetTime (min)	Area (mAU*min)	Height (mAU)	Area%	Height%
1	9.397	2.416	7.630	50.40	57.76
2	13.340	2.379	5.578	49.60	42.24

Chiral

Peak	RetTime (min)	Area (mAU*min)	Height (mAU)	Area%	Height%
1	9.023	0.838	3.222	7.67	11.82
2	12.583	10.089	24.034	92.33	88.18

Supplementary Figure 42. HPLC Traces of 44b (phenomenex Lux Amylose-1, *i*-PrOH/Hexanes (V/V) = 99:1, 1.0 mL/min).

Racemic

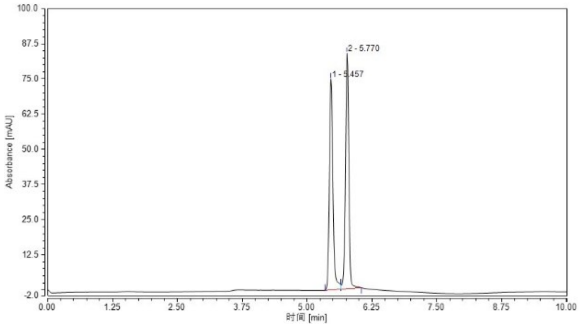

Chiral

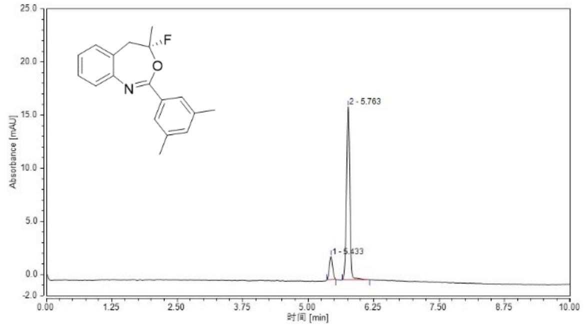

Racemic

Peak	RetTime (min)	Area (mAU*min)	Height (mAU)	Area%	Height%
1	5.457	6.018	74.897	49.20	47.22
2	5.770	6.213	83.703	50.80	52.78

Chiral

Peak	RetTime (min)	Area (mAU*min)	Height (mAU)	Area%	Height%
1	5.433	0.394	5.560	10.17	11.55
2	5.763	3.481	42.570	89.83	88.45

Supplementary Figure 43. HPLC Traces of 45b (phenomenex Lux Amylose-1, *i*-PrOH/Hexanes (V/V) = 99:1, 1.0 mL/min).

Racemic

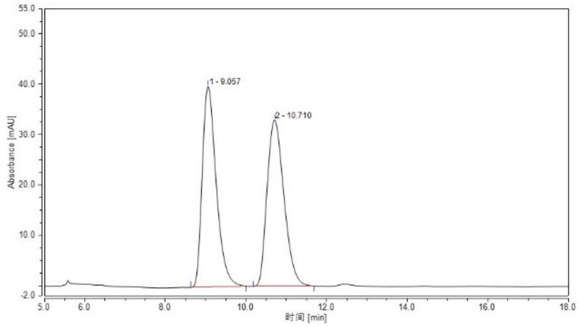

Chiral

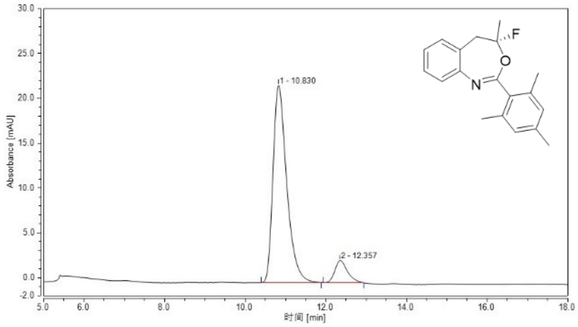

Racemic

Peak	RetTime (min)	Area (mAU*min)	Height (mAU)	Area%	Height%
1	9.057	15.999	39.879	49.99	54.71
2	10.710	16.003	33.012	50.01	45.29

Chiral

Peak	RetTime (min)	Area (mAU*min)	Height (mAU)	Area%	Height%
1	10.830	0.308	0.938	7.40	9.32
2	12.357	3.852	9.122	92.60	90.68

Supplementary Figure 44. HPLC Traces of 46b (phenomenex Cellulose-1, *i*-PrOH/Hexanes (V/V) = 95:5, 1.0 mL/min).

Racemic

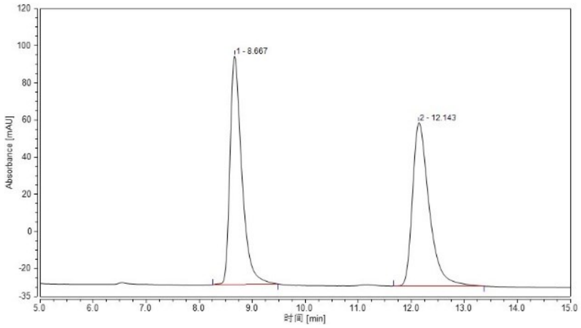

Chiral

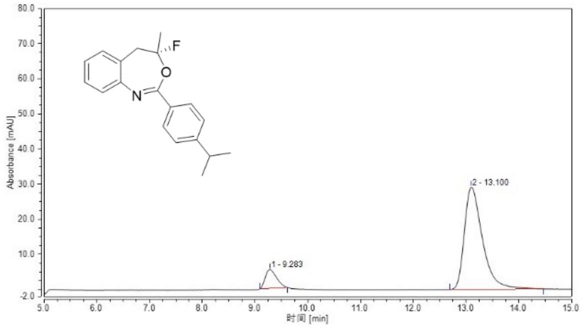

Racemic

Peak	RetTime (min)	Area (mAU*min)	Height (mAU)	Area%	Height%
1	8.667	31.847	122.987	49.85	58.29
2	12.143	32.039	88.006	50.15	41.71

Chiral

Peak	RetTime (min)	Area (mAU*min)	Height (mAU)	Area%	Height%
1	9.283	1.572	4.341	9.68	11.41
2	13.100	14.667	33.719	90.32	88.59

Supplementary Figure 45. HPLC Traces of 47b (phenomenex Lux Amylose-1, *i*-PrOH/Hexanes (V/V) = 99:1, 0.5 mL/min).

Racemic

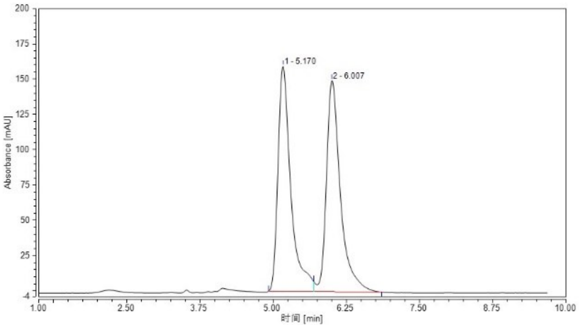

Chiral

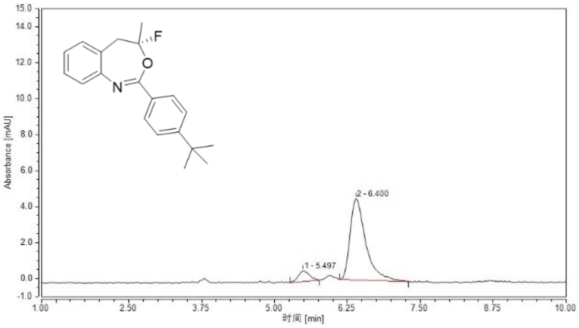

Racemic

Peak	RetTime (min)	Area (mAU*min)	Height (mAU)	Area%	Height%
1	5.170	40.407	159.491	49.31	51.57
2	6.007	41.540	149.802	50.69	48.43

Chiral

Peak	RetTime (min)	Area (mAU*min)	Height (mAU)	Area%	Height%
1	5.497	0.313	1.438	8.48	11.39
2	6.400	3.383	11.189	91.52	88.61

Supplementary Figure 46. HPLC Traces of 48b (phenomenex Cellulose-1, *i*-PrOH/Hexanes (V/V) = 99.9:0.1, 0.5 mL/min).

Racemic

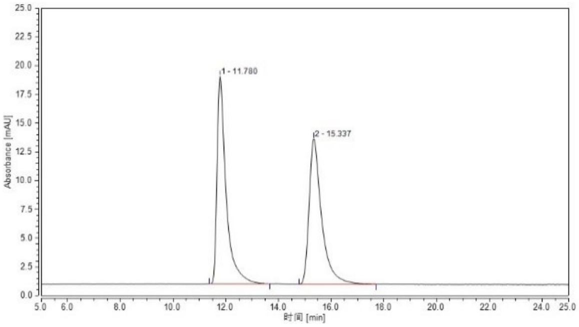

Chiral

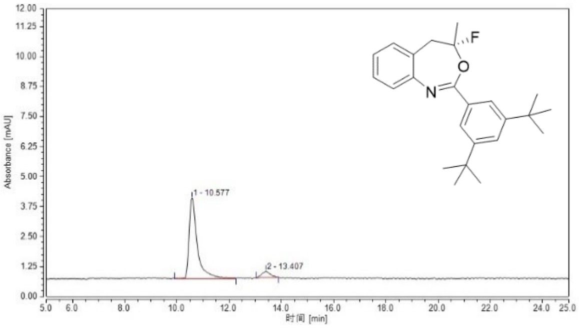

Racemic

Peak	RetTime (min)	Area (mAU*min)	Height (mAU)	Area%	Height%
1	11.780	7.223	18.062	50.82	58.76
2	15.337	6.990	12.677	49.18	41.24

Chiral

Peak	RetTime (min)	Area (mAU*min)	Height (mAU)	Area%	Height%
1	10.577	1.223	3.371	92.58	92.75
2	13.407	0.098	0.264	7.42	7.25

Supplementary Figure 47. HPLC Traces of 49b (phenomenex Cellulose-1, *i*-PrOH/Hexanes (V/V) = 99:1, 1.0 mL/min).

Racemic

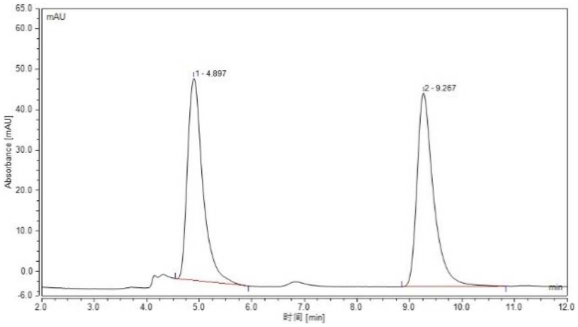

Chiral

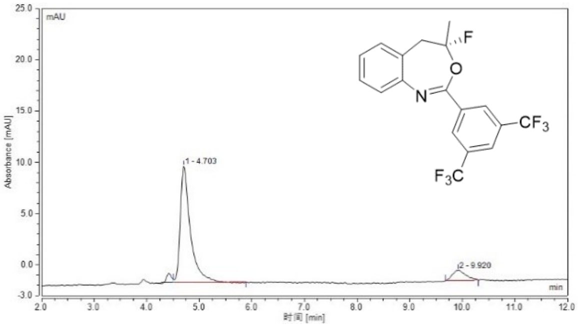

Racemic

Peak	RetTime (min)	Area (mAU*min)	Height (mAU)	Area%	Height%
1	4.897	16.814	49.947	49.13	51.03
2	9.267	17.408	47.924	50.87	48.97

Chiral

Peak	RetTime (min)	Area (mAU*min)	Height (mAU)	Area%	Height%
1	4.703	1.433	6.091	89.80	91.82
2	9.920	0.163	0.542	10.20	8.18

Supplementary Figure 48. HPLC Traces of 50b (phenomenex Lux Amylose-1, *i*-PrOH/Hexanes (V/V) = 98:2, 1.0 mL/min).

Racemic

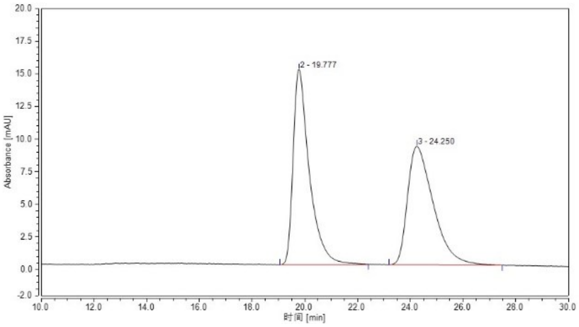

Chiral

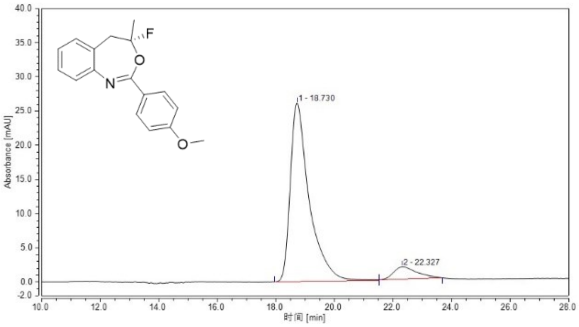

Racemic

Peak	RetTime (min)	Area (mAU*min)	Height (mAU)	Area%	Height%
1	19.777	10.634	14.962	50.82	60.97
2	24.250	10.293	9.089	49.18	37.03

Chiral

Peak	RetTime (min)	Area (mAU*min)	Height (mAU)	Area%	Height%
1	18.730	24.317	23.074	92.62	94.90
2	22.327	1.938	1.239	7.38	5.10

Supplementary Figure 49. HPLC Traces of 51b (phenomenex Cellulose-1, *i*-PrOH/Hexanes (V/V) = 98:2, 1.0 mL/min).

Racemic

Chiral

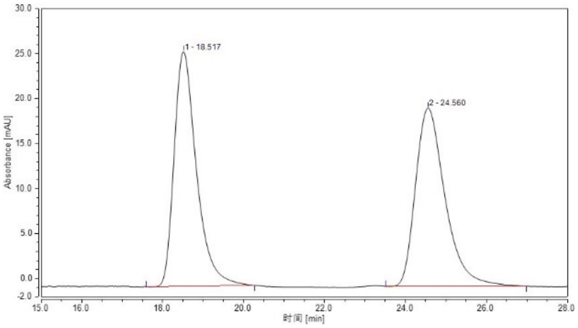

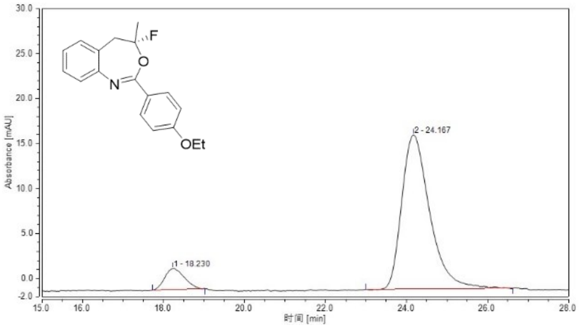

Racemic

Peak	RetTime (min)	Area (mAU*min)	Height (mAU)	Area%	Height%
1	18.517	16.670	26.078	49.87	56.83
2	24.560	16.754	19.808	50.13	43.17

Chiral

Peak	RetTime (min)	Area (mAU*min)	Height (mAU)	Area%	Height%
1	18.230	1.301	2.335	8.67	12.00
2	24.167	13.695	17.124	91.33	88.00

Supplementary Figure 50. HPLC Traces of 52b (phenomenex Lux Amylose-1, *i*-PrOH/Hexanes (V/V) = 99:1, 1.0 mL/min)

Racemic Chiral

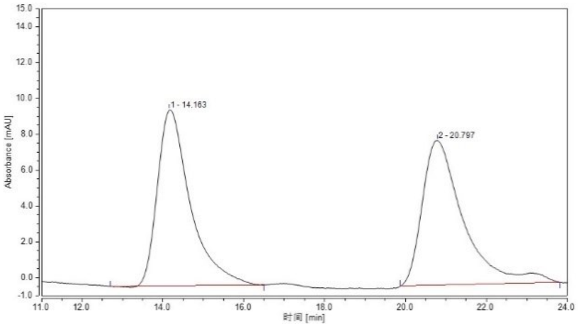

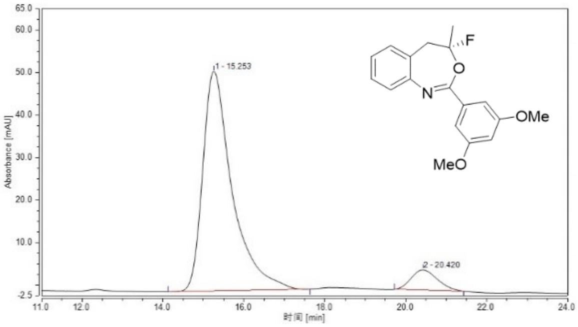

Racemic

Peak	RetTime (min)	Area (mAU*min)	Height (mAU)	Area%	Height%
1	14.163	9.218	9.814	49.26	54.89
2	20.797	9.494	8.064	50.74	45.11

Chiral

Peak	RetTime (min)	Area (mAU*min)	Height (mAU)	Area%	Height%
1	15.253	43.337	51.644	92.40	91.60
2	20.420	3.564	4.734	7.60	8.40

Supplementary Figure 51. HPLC Traces of 53b (phenomenex Lux Amylose-1, *i*-PrOH/Hexanes (V/V) = 99.8:0.2, 0.4 mL/min).

Racemic Chiral

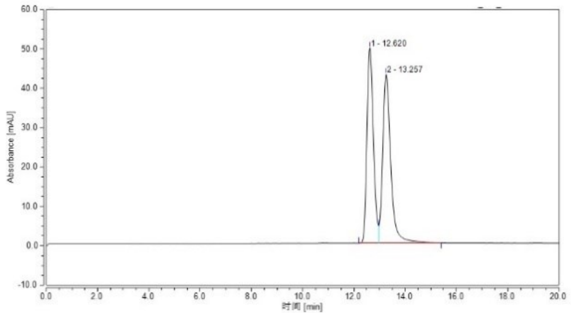

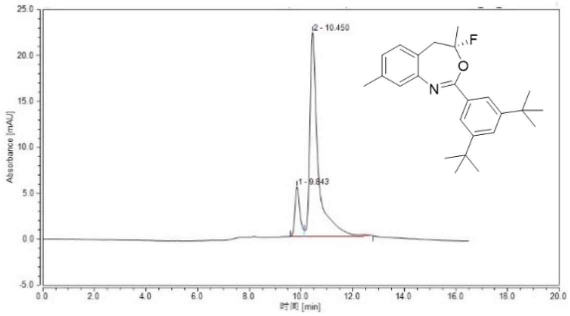

Racemic

Peak	RetTime (min)	Area (mAU*min)	Height (mAU)	Area%	Height%
1	12.620	14.510	49.623	47.98	53.63
2	13.257	15.729	42.913	52.02	46.37

Chiral

Peak	RetTime (min)	Area (mAU*min)	Height (mAU)	Area%	Height%
1	12.503	0.361	2.073	9.67	13.31
2	13.103	3.374	13.502	90.33	86.69

Supplementary Figure 52. HPLC Traces of 54b (phenomenex Lux Amylose-1, *i*-PrOH/Hexanes (V/V) = 99.5:0.5, 1.0 mL/min).

Racemic

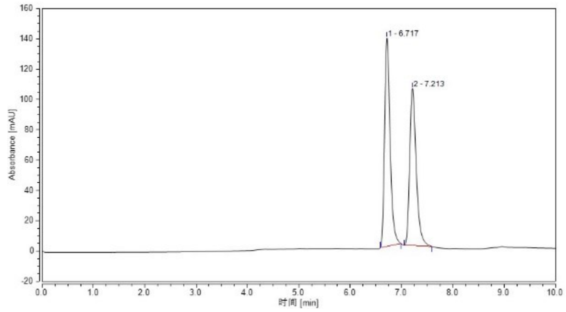

Chiral

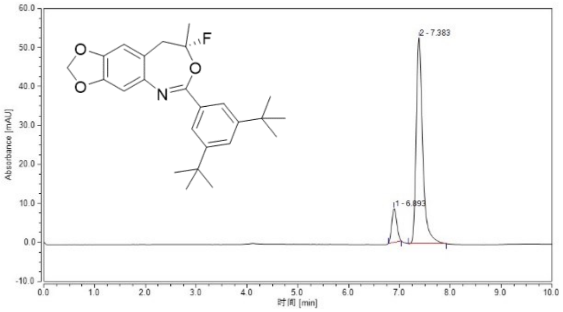

Racemic

Peak	RetTime (min)	Area (mAU*min)	Height (mAU)	Area%	Height%
1	6.717	17.036	137.383	52.82	56.98
2	7.213	15.218	103.707	47.18	43.12

Chiral

Peak	RetTime (min)	Area (mAU*min)	Height (mAU)	Area%	Height%
1	6.893	0.882	8.155	10.13	13.37
2	7.383	7.825	52.853	89.87	86.63

7. X-Ray Crystallography Information of 4b

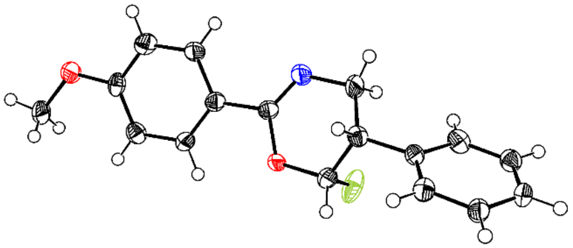

Supplementary Figure 53. The Crystal Structure of 4b. The single crystal of compound **4b** was obtained by recrystallization from dichloromethane/Hexane (V/V = 1:2) at room temperature. The ellipsoid contour was set at 50% probability levels. The crystal data of compound **4b** have been deposited in **CCDC** with number **1960281**. The crystal was kept at 100.00(10) K during data collection. Using Olex2, the structure was solved with the ShelXT structure solution program using Intrinsic Phasing and refined with the ShelXL refinement package using Least Squares minimisation. **Crystal Data** for $C_{17}H_{16}NO_2F$ (M =285.31 g/mol): monoclinic, space group $P2_1$ (no. 4), a = 5.97170(10) Å, b = 15.1439(3) Å, c = 15.5256(3) Å, β = 91.511(2)°, V = 1403.57(5) Å^3, Z = 4, T = 100.00(10) K, μ(CuKα) = 0.803 mm^{-1}, $Dcalc$ = 1.350 g/cm^3, 16525 reflections measured (5.694° ≤ 2Θ ≤ 144.136°), 5361 unique (R_{int} = 0.0436, R_{sigma} = 0.0435) which were used in all calculations. The final R_1 was 0.0902 (I > 2σ(I)) and wR_2 was 0.2909 (all data).

Supplementary Table 2. Crystal Data and Structure Refinement for 4b

Empirical formula	$C_{17}H_{16}NO_2F$
Formula weight	285.31
Temperature/K	100.00(10)
Crystal system	monoclinic
Space group	$P2_1$
a/Å	5.97170(10)
b/Å	15.1439(3)
c/Å	15.5256(3)
α/°	90
β/°	91.511(2)
γ/°	90
Volume/Å^3	1403.57(5)
Z	4
ρ_{calc}g/cm^3	1.350
μ/mm^{-1}	0.803
F(000)	600.0
Crystal size/mm^3	0.2 × 0.1 × 0.1
Radiation	Cu Kα (λ = 1.54184)
2Θ range for data collection/°	5.694 to 144.136
Index ranges	-5 ≤ h ≤ 7, -18 ≤ k ≤ 18, -19 ≤ l ≤ 19
Reflections collected	16525

Independent reflections	5361 [R_{int} = 0.0436, R_{sigma} = 0.0435]
Data/restraints/parameters	5361/1/381
Goodness-of-fit on F^2	1.320
Final R indexes [I>=2σ (I)]	R_1 = 0.0902, wR_2 = 0.2777
Final R indexes [all data]	R_1 = 0.0966, wR_2 = 0.2909
Largest diff. peak/hole / e Å^{-3}	1.46/-0.82
Flack parameter	0.15(11)

Supplementary Table 3. Fractional Atomic Coordinates (×104) and Equivalent Isotropic Displacement Parameters (Å2×103) for 4ba

Atom	*x*	*y*	*z*	U(eq)
O5	8535(6)	9112(3)	6030(3)	29.4(9)
F21	9041(8)	10162(3)	5024(2)	46.9(11)
O34	6008(8)	4612(3)	8482(3)	36.7(10)
O19	4261(7)	5372(3)	6608(3)	31.5(10)
C13	5800(9)	8018(4)	6234(3)	26.0(11)
N3	4781(8)	9563(4)	6157(4)	38.3(13)
C7	8721(9)	11577(4)	6273(3)	24.0(11)
N23	1784(10)	847(4)	9156(5)	50.7(17)
C14	3738(9)	7741(4)	6576(4)	27.5(12)
C4	6250(9)	8971(4)	6139(3)	24.5(11)
C17	6869(10)	6475(4)	6096(3)	28.1(12)
C29	2837(10)	2623(5)	9013(4)	32.0(13)
C1	7877(9)	10640(4)	6384(3)	23.8(11)
O27	4901(12)	456(4)	8408(6)	76(2)
C15	3313(9)	6848(4)	6699(4)	28.5(12)
C11	11437(9)	12696(4)	6697(4)	28.7(12)
C6	9253(9)	9980(4)	5903(4)	27.0(12)
C16	4864(10)	6216(4)	6461(3)	25.8(11)
C12	10557(9)	11856(4)	6773(4)	28.8(12)
C8	7801(10)	12177(4)	5671(3)	28.7(12)
C40	1437(12)	-3038(5)	9403(5)	40.6(15)
C39	-361(10)	-3298(4)	8865(4)	33.6(13)
C20	5847(12)	4697(4)	6376(5)	36.5(14)
C31	5349(10)	3757(4)	8569(4)	31.6(13)
C22	3695(11)	1019(5)	8766(4)	35.2(14)
C38	-1255(12)	-2722(5)	8290(4)	41.0(16)
C9	8695(9)	13016(4)	5594(3)	27.6(11)
C32	6809(10)	3107(4)	8280(4)	31.2(12)
C18	7318(9)	7387(4)	6004(4)	28.6(12)
C10	10503(9)	13290(4)	6105(4)	29.6(12)
C30	3316(10)	3494(4)	8935(4)	31.5(12)
C28	4284(10)	1966(4)	8716(4)	30.9(13)
C33	6292(10)	2223(4)	8351(4)	31.3(13)

C41	2247(12)	-2182(6)	9372(5)	48.2(19)
C37	-461(11)	-1871(5)	8235(5)	39.9(15)
C35	4416(12)	5279(5)	8694(5)	42.2(16)
C2	5454(10)	10493(4)	6102(5)	41.5(15)
C26	4329(14)	-474(6)	8487(8)	62(2)
C36	1322(11)	-1593(5)	8792(6)	48.3(19)
C24	950(15)	-8(5)	9162(10)	88(4)
C25	2150(20)	-645(6)	8768(14)	118(7)
F1	5450(30)	-628(14)	9226(10)	207(7)

[a]U_{eq} is defined as 1/3 of of the trace of the orthogonalised U_{IJ} tensor.

Supplementary Table 4. Anisotropic Displacement Parameters (Å^2×10^3) for 4b[a]

Atom	U_{11}	U_{22}	U_{33}	U_{23}	U_{13}	U_{12}
O5	17.2(17)	16.7(19)	55(2)	1.4(16)	4.3(15)	0.4(14)
F21	82(3)	26.7(18)	32.8(17)	-6.8(14)	24.5(17)	-15.6(18)
O34	39(2)	25(2)	46(2)	5.4(19)	5.0(18)	-1.7(18)
O19	29(2)	21(2)	45(2)	-0.2(17)	1.6(16)	-3.9(16)
C13	21(2)	26(3)	30(2)	0(2)	-3.7(19)	-3(2)
N3	19(2)	29(3)	68(3)	11(3)	-2(2)	-0.9(19)
C7	27(3)	18(3)	27(2)	-5(2)	5(2)	1(2)
N23	41(3)	23(3)	90(5)	-6(3)	35(3)	-5(2)
C14	21(2)	26(3)	36(3)	1(2)	-2(2)	-3(2)
C4	20(2)	23(3)	30(2)	-1(2)	-2.7(19)	-2.5(19)
C17	28(3)	28(3)	28(2)	-1(2)	-5(2)	2(2)
C29	27(3)	40(4)	29(3)	5(2)	-3(2)	-8(2)
C1	29(3)	22(3)	20(2)	-0.9(19)	2.1(18)	2(2)
O27	73(4)	30(3)	128(6)	-19(4)	56(4)	-15(3)
C15	20(2)	33(3)	32(3)	-3(2)	-5.8(19)	-7(2)
C11	27(3)	25(3)	34(3)	4(2)	-3(2)	-1(2)
C6	22(2)	21(3)	38(3)	-2(2)	2(2)	-4(2)
C16	35(3)	18(3)	23(2)	0.5(19)	-4(2)	-6(2)
C12	29(3)	25(3)	33(3)	1(2)	2(2)	0(2)
C8	31(3)	28(3)	27(2)	-6(2)	-3(2)	4(2)
C40	41(3)	36(4)	44(3)	-4(3)	0(3)	1(3)
C39	37(3)	31(3)	33(3)	-4(2)	3(2)	-2(2)
C20	41(3)	22(3)	46(3)	3(3)	7(3)	2(3)
C31	24(3)	29(3)	41(3)	-2(2)	-1(2)	1(2)
C22	41(3)	38(4)	27(2)	3(2)	-3(2)	-8(3)
C38	37(3)	52(4)	34(3)	-13(3)	-1(3)	-4(3)
C9	28(3)	28(3)	27(2)	2(2)	1.6(19)	3(2)
C32	31(3)	30(3)	33(3)	5(2)	10(2)	5(2)
C18	25(3)	24(3)	38(3)	1(2)	6(2)	-3(2)
C10	31(3)	18(3)	40(3)	1(2)	3(2)	-2(2)

C30	30(3)	30(3)	35(3)	0(2)	8(2)	2(2)
C28	34(3)	32(3)	27(2)	-2(2)	-3(2)	-1(2)
C33	31(3)	32(3)	31(3)	-1(2)	-1(2)	1(2)
C41	31(3)	55(5)	59(4)	-28(4)	5(3)	-8(3)
C37	37(3)	35(4)	48(3)	5(3)	6(3)	4(3)
C35	38(4)	32(4)	56(4)	3(3)	-1(3)	2(3)
C2	26(3)	21(3)	78(5)	9(3)	0(3)	-4(2)
C26	49(4)	39(4)	100(7)	-3(4)	37(5)	-1(3)
C36	30(3)	28(4)	88(6)	-5(4)	23(3)	2(3)
C24	52(5)	29(4)	185(13)	-25(6)	71(6)	-12(3)
C25	71(6)	32(5)	260(20)	-36(8)	85(10)	-14(4)
F1	199(13)	270(20)	147(12)	13(12)	-20(10)	-21(13)

[a]The Anisotropic displacement factor exponent takes the form: $-2\pi^2[h^2a^{*2}U_{11}+2hka^*b^*U_{12}+\ldots]$.

Supplementary Table 5. Bond Lengths for 4b

Atom	Atom	Length/Å	Atom	Atom	Length/Å
O5	C4	1.396(7)	C1	C2	1.516(8)
O5	C6	1.398(6)	O27	C22	1.255(9)
F21	C6	1.395(7)	O27	C26	1.455(10)
O34	C31	1.361(8)	C15	C16	1.388(8)
O34	C35	1.432(8)	C11	C12	1.383(8)
O19	C16	1.349(7)	C11	C10	1.393(8)
O19	C20	1.446(8)	C8	C9	1.384(8)
C13	C14	1.418(8)	C40	C39	1.400(9)
C13	C4	1.476(8)	C40	C41	1.385(11)
C13	C18	1.370(8)	C39	C38	1.349(10)
N3	C4	1.254(8)	C31	C32	1.396(8)
N3	C2	1.468(8)	C31	C30	1.411(9)
C7	C1	1.518(8)	C22	C28	1.480(9)
C7	C12	1.392(8)	C38	C37	1.377(11)
C7	C8	1.405(8)	C9	C10	1.387(8)
N23	C22	1.331(9)	C32	C33	1.380(9)
N23	C24	1.387(9)	C28	C33	1.395(9)
C14	C15	1.390(8)	C41	C36	1.373(13)
C17	C16	1.394(8)	C37	C36	1.418(11)
C17	C18	1.415(8)	C26	C25	1.410(12)
C29	C30	1.357(9)	C26	F1	1.335(19)
C29	C28	1.403(9)	C36	C25	1.518(12)
C1	C6	1.504(7)	C24	C25	1.357(12)

Supplementary Table 6. Bond Angles for 4b

Atom	Atom	Atom	Angle/°	Atom	Atom	Atom	Angle/°
C4	O5	C6	117.7(4)	C41	C40	C39	120.4(7)
C31	O34	C35	117.0(5)	C38	C39	C40	119.8(6)
C16	O19	C20	116.6(5)	O34	C31	C32	117.0(5)
C14	C13	C4	119.3(5)	O34	C31	C30	124.2(6)
C18	C13	C14	118.5(5)	C32	C31	C30	118.8(6)
C18	C13	C4	122.2(5)	N23	C22	C28	114.9(6)
C4	N3	C2	119.4(5)	O27	C22	N23	125.3(7)
C12	C7	C1	118.7(5)	O27	C22	C28	119.7(6)
C12	C7	C8	117.6(5)	C39	C38	C37	121.0(6)
C8	C7	C1	123.7(5)	C8	C9	C10	121.4(5)
C22	N23	C24	119.9(6)	C33	C32	C31	121.1(6)
C15	C14	C13	120.2(5)	C13	C18	C17	121.8(6)
O5	C4	C13	110.0(5)	C9	C10	C11	118.5(5)
N3	C4	O5	125.5(5)	C29	C30	C31	119.8(6)
N3	C4	C13	124.5(5)	C29	C28	C22	121.4(6)
C16	C17	C18	118.8(5)	C33	C28	C29	118.7(6)
C30	C29	C28	121.8(6)	C33	C28	C22	119.9(6)
C6	C1	C7	112.2(4)	C32	C33	C28	119.9(6)
C6	C1	C2	106.8(5)	C36	C41	C40	119.7(7)
C2	C1	C7	114.9(5)	C38	C37	C36	119.6(6)
C22	O27	C26	118.7(6)	N3	C2	C1	112.6(5)
C16	C15	C14	120.5(5)	C25	C26	O27	115.2(8)
C12	C11	C10	120.2(5)	F1	C26	O27	97.4(12)
O5	C6	C1	112.4(5)	F1	C26	C25	98.5(13)
F21	C6	O5	107.7(4)	C41	C36	C37	119.4(7)
F21	C6	C1	108.5(4)	C41	C36	C25	120.3(10)
O19	C16	C17	124.8(5)	C37	C36	C25	120.3(10)
O19	C16	C15	115.1(5)	C25	C24	N23	117.9(7)
C15	C16	C17	120.0(5)	C26	C25	C36	119.0(8)
C11	C12	C7	121.9(5)	C24	C25	C26	120.7(9)
C9	C8	C7	120.4(5)	C24	C25	C36	119.2(8)

Supplementary Table 7. Hydrogen Atom Coordinates (Å×10[4]) and Isotropic Displacement Parameters (Å[2]×10[3]) for 4b

Atom	*x*	*y*	*z*	U(eq)
H23	1068.69	1265.01	9402.99	61
H14	2665.59	8157.52	6719.04	33
H17	7892.94	6055.88	5915.17	34
H29	1512.44	2455.8	9269.74	38

H1	8012.06	10494.47	6997.79	29
H15	1979.02	6672.01	6942.93	34
H11	12657.18	12865.34	7043.79	34
H6	10828.46	10033.73	6088.22	32
H12	11209.43	11466.54	7168.43	35
H8	6583.32	12010	5322.87	34
H40	2092.23	-3442.25	9783.45	49
H39	-938.47	-3866.74	8904.35	40
H20A	6079.9	4719.23	5766.95	55
H20B	5269.68	4127.08	6526.13	55
H20C	7244.25	4795.45	6680.76	55
H38	-2426.3	-2902.77	7924.06	49
H9	8068.92	13403.6	5190.32	33
H32	8150.65	3273.6	8036.59	37
H18	8678.83	7563.55	5782.74	34
H10	11079.27	13857.66	6052.83	35
H30	2308.7	3916.97	9121.6	38
H33	7281.98	1797.83	8155.35	38
H41	3411.55	-2006.43	9743.38	58
H37	-1089.74	-1480.82	7834.08	48
H35A	3094.07	5212.77	8334.49	63
H35B	5057.73	5851.81	8601.28	63
H35C	4029.96	5221.29	9287.38	63
H2A	4495.08	10844.92	6461.92	50
H2B	5240.44	10694.01	5512.45	50
H26	4791.27	-841.56	8003.38	75
H24	-386.88	-139.35	9428.33	105
H25	1450.55	-529.14	8201.1	142

8. DFT Calculations

Gibbs free energy profiles (in kcal/mol) for the reaction at B3LYP-D3BJ/def2-TZVP/6-311+G(d,p)/SMD(DCE)//B3LYP-D3BJ/SDD/6-31G(d) level.

8.1 Method

All calculations were carried out with the Gaussian 09 software[1]. The B3LYP functional[2] was adopted for all calculations in combination with the D3BJ dispersion correction[3]. For geometry optimization and frequency calculations, the SDD ECP and basis set[4] was used for I and 6-31G(d) for others[5,6]. The singlet point energy calculations were performed with a larger basis set combination, in which the def2-TZVP basis set[7] was used for I, and 6-311+G(d,p)[8,9] for others. The SMD implicit solvation model[10] was used to account for the solvation effect of DCE when performing single point energy calculations.

References:

1. Frisch, M., Trucks, G. W., Schlegel, H. B., Scuseria, G. E., Robb, M. A., Cheeseman, J. R., Scalmani, G., Barone, V., Mennucci, B., Petersson, G. Others, Gaussian 09, revision D. 01. In Gaussian, Inc., Wallingford CT: 2009.
2. Stephens, P. J., Devlin, F. J., Chabalowski, C. F., Frisch, M. J. Ab initio calculation of vibrational absorption and circular dichroism spectra using density functional force fields. *J. Phys. Chem.* **98**, 11623-11627 (1994).
3. Grimme, S., Antony, J., Ehrlich S., Krieg, H. A consistent and accurate ab initio parametrization of density functional dispersion correction (DFT-D) for the 94 elements H-Pu. *J. Chem. Phys.* **132**, 154104 (2010).
4. Bergner, A. et al. *Ab initio* energy-adjusted pseudopotentials for elements of groups 13-17. *Mol. Phys.* **80**, 1431-1441 (1993).
5. Hariharan, P. C. & Pople, J. A. The influence of polarization functions on molecular orbital hydrogenation energies. *Theor. Chim. Acta.* **28**, 213-222 (1973).
6. Gnan, N. et al. Dynamical properties of different models of elastic polymer rings: Confirming the link between deformation and fragility. *J. Chem. Phys.* **56**, 2257-2261 (1972).
7. Weigend F., Ahlrichs, R., Balanced basis sets of split valence, triple zeta valence and quadruple zeta valence quality for H to Rn: Design and assessment of accuracy. *Phys. Chem. Chem. Phys.* **7**, 3297 (2005).
8. Clark, T., Chandrasekhar, J., Spitznagel, G. W. & Schleyer, P. V. Efficient diffuse function-augmented basis sets for anion calculations. III. The 3-21+G basis set for first-row elements, Li-F. *J. Comput. Chem.* **4**, 294-301 (1983).
9. Krishnan, R., Binkley, J. S., Seeger, R. & Pople, J. A. Self-consistent molecular orbital methods. XX. A basis set for correlated wave functions. *J. Chem. Phys.* **72**, 650-654 (1980).
10. Marenich, A. V., Cramer, C. J. &Truhlar, D. G. Universal solvation model based on solute electron density and on a continuum model of the solvent defined by the bulk dielectric constant and atomic surface tensions. *J. Phys. Chem. B* **113**, 6378-6396 (2009).

8.2 Cartesian Coordinates of Species Reported in Figure 6.

Supplementary Table 8. Cartesian Coordinates of Species Reported in Figure 6

1a

C	2.39413	-0.87510	-0.53068
H	2.39821	-1.38765	-1.49375
C	1.27610	-0.94190	0.20645
H	1.20539	-0.43362	1.16658
C	3.64450	-0.18065	-0.19836
C	4.67658	-0.14980	-1.15112
C	3.86228	0.46236	1.03391
C	5.87992	0.50365	-0.89123
H	4.52667	-0.64513	-2.10745
C	5.06233	1.11577	1.29445
H	3.08947	0.44483	1.79615
C	6.07775	1.14116	0.33353
H	6.66230	0.51363	-1.64493
H	5.20999	1.60485	2.25335
H	7.01435	1.65038	0.54148
C	0.04370	-1.68373	-0.21952
H	-0.23945	-2.45027	0.50889
H	0.21485	-2.19020	-1.17962
N	-1.10287	-0.78817	-0.30716
H	-1.01796	0.02418	-0.89990
C	-2.32453	-1.10623	0.22261
O	-2.53621	-2.16968	0.79994
C	-3.40098	-0.07335	0.05145
C	-4.72859	-0.51079	0.13367
C	-3.13848	1.28723	-0.15666
C	-5.77775	0.39194	-0.01841
H	-4.91045	-1.56359	0.32034
C	-4.18949	2.19264	-0.30037
H	-2.11607	1.65475	-0.16894
C	-5.51037	1.74547	-0.23883
H	-6.80471	0.04249	0.03799
H	-3.97679	3.24709	-0.45134
H	-6.32854	2.45090	-0.35350

ArI (**CIC1**)

C	0.02185	2.65740	-0.32914
C	1.23095	1.96906	-0.29200
C	1.22508	0.56646	-0.26987
C	0.00018	-0.11692	-0.28473
C	-1.21338	0.58436	-0.32320
C	-1.19734	1.98712	-0.34582
H	0.03048	3.74309	-0.34743
H	2.16257	2.51978	-0.28266
H	-2.12087	2.55084	-0.37868
O	-2.34436	-0.16916	-0.35196
O	2.34237	-0.20511	-0.22262
C	-3.61398	0.45599	-0.18542
H	-3.71236	1.32433	-0.84281
C	3.62744	0.41095	-0.17255
H	3.61950	1.26692	0.50770
C	-4.63161	-0.59832	-0.62062
O	-4.71933	-1.69784	-0.12221
O	-5.41931	-0.12505	-1.59942
C	4.54995	-0.66169	0.40824
O	4.76537	-1.72711	-0.12493
O	5.08931	-0.25123	1.56665
C	4.11451	0.81568	-1.57386
H	4.16217	-0.09701	-2.17525
H	3.36606	1.47546	-2.02372
C	-3.86281	0.83798	1.28457
H	-3.04818	1.49109	1.61246
H	-3.80911	-0.08492	1.86953
C	-6.46754	-1.02287	-2.06537
H	-6.67727	-0.66822	-3.07582
H	-6.05988	-2.03445	-2.10599
C	6.02821	-1.17112	2.19438
H	6.00758	-0.87971	3.24580
H	5.64233	-2.18599	2.08467
H	7.19717	-2.71508	0.28613
C	9.99720	-0.79674	0.56172
H	10.19736	0.90675	1.87022
H	9.49750	-2.51970	-0.63583
H	11.00255	-0.70761	10.15923
C	-7.69677	-0.95761	-1.19792
C	-8.65412	0.04013	-1.41149
C	-7.89798	-1.89001	-0.17301
C	-9.80258	0.10078	-0.62376
H	-8.49807	0.77080	-2.20114
C	-9.04707	-1.83179	0.61567
H	-7.14416	-2.64943	0.00623
C	-10.00378	-0.84000	40.38830
H	-10.54094	0.87743	-0.80147
H	-9.19798	-2.56365	1.40423
H	-10.90246	-0.80001	0.99780
C	5.45949	1.49865	-1.49756
C	6.64429	0.75396	-1.54881
C	5.54123	2.88209	-1.29522
C	7.88222	1.37774	-1.39887
H	6.59437	-0.32110	-1.69097
C	6.77796	3.50957	-1.14661
H	4.62746	3.47199	-1.26078
C	7.95242	2.75633	-1.19522
H	8.78746	0.78135	-1.42614
H	6.82432	4.58451	-0.99453
H	8.91707	3.24186	-1.07636
C	-5.19886	1.52056	1.45074
C	-6.34766	0.78216	1.75862
C	-5.32189	2.89792	1.22891
C	-7.59161	1.40656	1.84361
H	-6.26837	-0.28894	1.91844
C	-6.56449	3.52547	1.31178

C	7.41242	-1.04085	1.61571	H	-4.43454	3.48326	0.99634
C	8.26064	-0.01535	2.04828	C	-7.70377	2.77880	1.61804
C	7.86457	-1.93568	0.63886	H	-8.47192	0.8157	2.07118
C	9.54647	0.10871	1.52472	H	-6.64260	4.59566	1.14042
H	7.90976	0.68795	2.79927	H	-8.67351	3.26454	1.68163
C	9.15259	-1.81582	0.11635	I	-0.01680	-2.24738	-0.23473

Int1 (ArIO)

C	-0.24842	3.73201	-1.28486	C	8.76991	-2.44592	0.87837
C	1.01853	3.15935	-1.38608	H	8.69402	-2.18318	3.01785
C	1.14357	1.76997	-1.50883	H	8.52513	-2.67129	-1.25306
C	-0.01979	0.99390	-1.50874	H	9.85008	-2.56183	0.84858
C	-1.29181	1.56582	-1.44870	C	-7.45118	-1.11636	-0.59133
C	-1.40694	2.95437	-1.32866	C	-8.53126	-0.45805	0.00624
H	-0.33976	4.81034	-1.19589	C	-7.02000	-2.34081	-0.06563
H	1.91019	3.77575	-1.39657	C	-9.18156	-1.01722	1.10537
H	-2.38404	3.42240	-1.28530	H	-8.86461	0.49583	-0.39456
O	-2.34851	0.70805	-1.55487	C	-7.66792	-2.90056	1.03519
O	2.33884	1.15436	-1.69394	H	-6.17142	-2.84182	-0.51961
C	-3.50366	0.94071	-0.74211	C	-8.75306	-2.24273	1.61901
H	-3.93268	1.92638	-0.94317	H	-10.02224	-0.49958	1.55830
C	3.27407	1.10940	-0.59637	H	-7.33070	-3.85351	1.43296
H	2.77066	1.37360	0.33505	H	-9.26319	-2.68403	2.47082
C	-4.50333	-0.12981	-1.17264	C	5.55576	1.99924	0.10505
O	-4.23195	-1.30727	-1.25661	C	6.66282	1.17318	-0.1156
O	-5.70517	0.41193	-1.41423	C	5.47655	2.71492	1.30512
C	3.77877	-0.33134	-0.50909	C	7.66814	1.05841	0.84259
O	4.01908	-1.01360	-1.48203	H	6.72386	0.59637	-1.03377
O	4.06196	-0.64406	0.76407	C	6.4806	2.60431	2.26680
C	4.43890	2.06797	-0.90833	H	4.61976	3.36015	1.48789
H	4.80389	1.80605	-1.90684	C	7.57850	1.77290	2.03754
H	4.03367	3.08492	-0.96227	H	8.50615	0.39383	0.66299
C	-3.16863	0.78406	0.75169	H	6.40513	3.16565	3.19428
H	-2.38160	1.50535	0.99698	H	8.35891	1.68031	2.78784
H	-2.75535	-0.21962	0.89114	C	-4.38604	1.00368	1.61560
C	-6.76790	-0.51425	-1.78976	C	-5.15728	-0.07945	2.05121
H	-7.45504	0.10692	-2.36624	C	-4.79806	2.30175	1.94128
H	-6.33653	-1.28352	-2.43263	C	-6.31546	0.12923	2.79883
C	4.48056	-2.00946	1.00613	H	-4.85524	-1.09012	1.79268
H	4.10011	-2.22949	2.00511	C	-5.95712	2.51435	2.68661
H	3.97780	-2.64840	0.27894	H	-4.20155	3.15014	1.61222
C	5.97918	-2.16320	0.95464	C	-6.71972	1.42627	3.11578
C	6.73365	-2.09388	2.12983	H	-6.90695	-0.72158	3.11801
C	6.63752	-2.36968	-0.26499	H	-6.26247	3.52710	2.93499
C	8.12113	-2.23614	2.09606	H	-7.62382	1.58897	3.69578
H	6.22891	-1.92471	3.07770	I	0.20528	-1.12839	-1.32693
C	8.02460	-2.50832	-0.30233	O	1.46652	-1.19201	0.14324
H	6.05099	-2.40371	-1.17706				

Int2

C	-0.03299	-3.65132	-0.61020	H	-9.03685	2.36950	-0.05813
C	-1.20478	-2.91470	-0.77580	H	-9.79760	1.29045	2.04903
C	-1.13344	-1.56633	-1.15697	C	7.72712	0.38274	-0.68725
C	0.14254	-0.99957	-1.30606	C	8.73758	-0.25195	0.04280
C	1.31658	-1.74745	-1.17738	C	7.39938	1.71191	-0.39132
C	1.22864	-3.09586	-0.82431	C	9.42159	0.43168	1.04691
H	-0.10751	-4.69693	-0.32751	H	8.99050	-1.28546	-0.17964
H	-2.16746	-3.38491	-0.62264	C	8.08096	2.39647	0.61473
H	2.12509	-3.69770	-0.73010	H	6.60539	2.19859	-0.94837
O	2.47830	-1.09398	-1.45289	C	9.09665	1.75939	1.33139
O	-2.18803	-0.76818	-1.39438	H	10.20797	-0.06905	1.60429
C	3.60580	-1.29342	-0.58485	H	7.82452	3.42893	0.83402
H	3.92454	-2.33925	-0.60051	H	9.63366	2.29646	2.10834
C	-3.43219	-1.02244	-0.70484	C	-5.58615	-2.28178	-0.70709
H	-3.21104	-1.48358	0.26061	C	-6.66198	-1.40246	-0.52411
C	4.70444	-0.42164	-1.18747	C	-5.59917	-3.51013	-0.03431
O	4.53399	0.73292	-1.51262	C	-7.71944	-1.74245	0.31901
O	5.85428	-1.09967	-1.27540	H	-6.66351	-0.44303	-1.02955
C	-4.04933	0.36379	-0.47911	C	-6.65937	-3.85596	0.80368
O	-4.60918	0.97875	-1.35974	H	-4.77382	-4.20582	-0.17465
O	-3.91615	0.74370	0.78957	C	-7.72123	-2.96845	0.98564
C	-4.38583	-1.88550	-1.53997	H	-8.53211	-1.03837	0.46220
H	-4.67780	-1.29869	-2.41535	H	-6.65518	-4.81552	1.31362
H	-3.85603	-2.77570	-1.89288	H	-8.54541	-3.23165	1.64281
C	3.28946	-0.83404	0.84878	C	4.47295	-1.03084	1.76416
H	2.42473	-1.40516	1.20410	C	5.34139	0.02921	2.04522
H	2.99662	0.21919	0.80147	C	4.74912	-2.29427	2.30078
C	7.00757	-0.35897	-1.78060	C	6.46233	-0.16775	2.85069
H	7.63826	-1.13609	-2.21465	H	5.14379	1.01084	1.62455
H	6.65933	0.31664	-2.56362	C	5.87156	-2.49561	3.10283
C	-4.53063	2.02504	1.13712	H	4.07490	-3.12291	2.09401
H	-3.97980	2.33611	2.02393	C	6.73143	-1.43058	3.37878
H	-4.32903	2.72421	0.32903	H	7.12930	0.66305	3.05146
C	-6.00084	1.83535	1.39525	H	6.07168	-3.48028	3.51612
C	-6.43769	1.22015	2.57449	H	7.60615	-1.5844	4.00433
C	-6.94754	2.23946	0.44735	I	0.33236	1.09565	-1.47304
C	-7.7968	1.01966	2.80910	O	-0.82295	1.44538	0.11353
H	-5.70457	0.89599	3.30877	B	-1.32409	2.89167	-0.02371
C	-8.30982	2.04559	0.68153	F	-0.24635	3.61019	-0.60642
H	-6.60620	2.69352	-0.47742	F	-2.41438	2.93500	-0.87761
C	-8.73684	1.43772	1.86393	F	-1.62496	3.27934	1.25774
H	-8.12391	0.54284	3.72890				

m-CPBA

C	2.17906	1.19957	-0.00006	H	-0.93321	2.59264	-0.00010
C	1.79891	-0.14372	0.00002	H	1.48440	3.23342	-0.00014
C	0.45902	-0.51689	0.00008	Cl	3.03481	-1.38656	0.00003
C	-0.51857	0.48708	0.00004	C	-1.96428	0.16891	0.00007
C	-0.15414	1.83903	-0.00005	O	-2.87606	0.97942	0.00020
C	1.19347	2.18768	-0.00009	O	-2.20208	-1.16516	-0.00015

H	3.23122	1.46191	-0.00010	O	-3.61570	-1.43617	-0.00009
H	0.18107	-1.56326	0.00016	H	-3.96534	-0.50788	-0.00005

m-CBA

C	1.58298	1.38111	-0.00001	H	-1.73392	2.17511	0.00001
C	1.45376	-0.00822	0.00000	H	0.52595	3.25272	0.00000
C	0.20436	-0.62062	-0.00002	Cl	2.89832	-1.00555	0.00002
C	-0.94128	0.18442	-0.00003	C	-2.30703	-0.40302	-0.00001
C	-0.82808	1.57959	-0.00001	O	-3.33652	0.24098	0.00006
C	0.43186	2.17118	-0.00001	O	-2.30026	-1.75888	-0.00001
H	2.5695	1.83160	-0.00001	H	-3.23737	-2.02863	-0.00007
H	0.11923	-1.69979	0.00000				

$BF_3 \cdot Et_2O$

O	-0.18412	0.56922	-0.00616	H	-1.68168	0.16565	1.37680
B	0.28072	-1.10269	0.01284	C	-2.54798	0.70435	-0.54685
F	-0.9173	-1.73692	0.13125	H	-2.55134	-0.31737	-0.92949
F	0.90802	-1.24845	-1.1786	H	-3.54017	0.93003	-0.14031
F	1.08189	-1.1753	1.11323	H	-2.3437	1.39516	-1.37072
C	0.85640	1.50104	0.41676	C	2.08680	1.36280	-0.45731
H	0.42362	2.50033	0.31539	H	1.82244	1.45698	-1.51381
H	1.07978	1.30946	1.47051	H	2.78602	2.16475	-0.19613
C	-1.50895	0.85218	0.54603	H	2.58285	0.40456	-0.30421
H	-1.47962	1.87397	0.93235				

BF_4^-

B	0.00000	0.00000	0.00000	F	0.81389	-0.81389	-0.81389
F	0.81389	0.81389	0.81389	F	-0.81389	0.81389	-0.81389
F	-0.81389	-0.81389	0.81389				

BF_3

B	0.00000	0.00000	0.00000	F	-1.14115	-0.65884	0.00000
F	0.00000	1.31769	0.00000	F	1.14115	-0.65884	0.00000

BF_2OBF_2

B	-1.23491	-0.09079	-0.0487	B	1.23485	-0.09076	0.04872
F	-2.28406	-0.80509	0.31021	F	1.41668	1.14834	0.47648
F	-1.41656	1.14833	-0.47649	F	2.28401	-0.80519	-0.31011
O	-0.00003	-0.65872	-0.00011				

BF_2OH

B	0.00000	0.02494	0.00000	O	-1.24300	-0.50693	0.00000
F	0.14737	1.33961	0.00000	H	-1.22397	-1.47437	0.00000
F	1.09352	-0.73903	0.00000				

BF_2OHBF_3

B	1.69133	-0.04222	-0.00002	F	-1.49569	0.70764	1.14475
F	1.65403	1.27387	0.00023	F	-1.49626	0.70845	-1.1444
F	2.85885	-0.67258	0.00053	F	-2.09119	-1.18455	-0.00035

O	0.51845	-0.73333	-0.00072	H	0.61061	-1.69695	-0.00077
B	-1.61652	0.05583	-0.00003				

Int3

C	-1.14831	1.70434	4.46729	C	3.58438	-4.44181	2.63723
C	0.07145	1.37055	3.87866	H	1.99978	-3.19243	3.38756
C	0.16604	1.24296	2.48943	C	5.44842	-3.28707	1.62668
C	-0.98806	1.46078	1.72033	H	5.30652	-1.13263	1.58663
C	-2.21776	1.79662	2.30445	C	4.82550	-4.47846	1.99823
C	-2.28882	1.92185	3.69396	H	3.09334	-5.36594	2.93018
H	-1.20937	1.80384	5.54668	H	6.40397	-3.30905	1.11320
H	0.96385	1.21851	4.4753	H	5.30191	-5.43184	1.78672
H	-3.23612	2.19081	4.14914	C	-4.95932	-1.40231	1.57722
O	-3.28711	2.04550	1.50174	C	-5.05061	-2.37948	0.58171
O	1.35298	0.97251	1.88953	C	-5.89984	-1.39614	2.61455
C	-4.35479	1.07593	1.43539	C	-6.07604	-3.32513	0.61492
H	-5.07272	1.27561	2.23815	H	-4.32085	-2.38725	-0.21977
C	1.75081	-0.41769	1.75451	C	-6.92661	-2.33789	2.64690
H	0.89061	-1.06184	1.94779	H	-5.83072	-0.65086	3.40457
C	-5.02563	1.33164	0.08766	C	-7.01913	-3.30405	1.64223
O	-4.47268	1.83772	-0.86457	H	-6.14210	-4.06664	-0.17450
O	-6.28416	0.87757	0.10785	H	-7.65198	-2.32016	3.45603
C	2.16532	-0.61677	0.29626	H	-7.82085	-4.03723	1.66398
O	2.57749	0.27163	-0.42554	I	-0.88872	1.17754	-0.36122
O	2.01536	-1.89027	-0.04239	O	-0.79673	-0.80532	-0.15028
C	2.90356	-0.68854	2.73878	B	-1.25810	-1.42212	-1.46257
H	3.62403	0.12412	2.62051	F	-0.75706	-0.57905	-2.48915
H	2.48244	-0.62992	3.75065	F	-0.73830	-2.69110	-1.49782
C	-3.84744	-0.38000	1.52585	F	-2.65888	-1.41486	-1.51635
H	-3.23556	-0.45403	2.43271	C	1.23568	4.30690	0.14246
H	-3.19278	-0.58780	0.67758	H	1.27168	5.06715	0.92361
C	-6.98739	0.83882	-1.1718	C	2.21274	3.39594	0.05247
H	-8.03009	1.02633	-0.91153	H	2.14293	2.57766	-0.65856
H	-6.60536	1.65526	-1.78741	C	0.03103	4.27867	-0.70808
C	2.30659	-2.26035	-1.42339	C	-1.24669	4.44016	-0.13483
H	1.61252	-3.07485	-1.61743	C	0.11156	4.00977	-2.08609
H	2.04280	-1.41203	-2.05675	C	-2.40340	4.30632	-0.90652
C	3.74275	-2.67648	-1.58112	H	-1.32729	4.63732	0.93087
C	4.08245	-4.03270	-1.58656	C	-1.04479	3.88048	-2.85632
C	4.75833	-1.71908	-1.70343	H	1.08931	3.89461	-2.54376
C	5.41290	-4.43099	-1.71949	C	-2.30543	4.02063	-2.26866
H	3.29897	-4.77852	-1.48116	H	-3.37637	4.37355	-0.43456
C	6.08901	-2.11288	-1.82849	H	-0.96110	3.65976	-3.91646
H	4.49830	-0.66681	-1.68593	H	-3.20525	3.88433	-2.85884
C	6.41808	-3.47076	-1.84062	C	3.40607	3.32337	0.95769
H	5.66343	-5.48834	-1.72462	H	3.25886	2.53002	1.69477
H	6.86480	-1.36074	-1.91886	H	3.55439	4.26677	1.49985
H	7.45550	-3.77854	-1.94400	N	4.60359	2.96040	0.21343
C	-6.81913	-0.50029	-1.83718	H	4.80107	3.45988	-0.64117
C	-7.89253	-1.39335	-1.90152	C	5.37845	1.88858	0.5386

C	-5.57898	-0.87986	-2.36719
C	-7.73688	-2.64513	-2.49763
H	-8.85266	-1.11035	-1.47697
C	-5.41608	-2.13787	-2.94320
H	-4.72856	-0.21211	-2.29917
C	-6.49667	-3.01974	-3.01736
H	-8.57889	-3.33021	-2.54464
H	-4.43647	-2.42618	-3.30986
H	-6.37059	-3.99870	-3.47192
C	3.58583	-2.01852	2.51930
C	2.97024	-3.21731	2.89611
C	4.83186	-2.06309	1.88307
O	5.25492	1.27325	1.59901
C	6.43748	1.52169	-0.45746
C	7.60891	0.92620	0.02546
C	6.28799	1.72980	-1.83474
C	8.63543	0.58345	-0.85125
H	7.69472	0.74573	1.09156
C	7.30979	1.37202	-2.71425
H	5.35902	2.13331	-2.22793
C	8.49021	0.81108	-2.22259
H	9.54684	0.13406	-0.46777
H	7.18023	1.52156	-3.78202
H	9.28777	0.53802	-2.90761

Int3^{+}

C	0.34472	-3.75652	2.22879
C	-0.90729	-3.35403	1.76152
C	-0.98418	-2.41377	0.73089
C	0.20624	-1.88190	0.22394
C	1.46936	-2.29981	0.66900
C	1.52805	-3.25573	1.68653
H	0.39917	-4.49776	3.01966
H	-1.81699	-3.78119	2.16826
H	2.49168	-3.60415	2.04007
O	2.53579	-1.75992	0.04742
O	-2.14906	-2.03016	0.14586
C	3.69973	-1.32043	0.77719
H	4.37109	-2.16730	0.94540
C	-3.21375	-1.54268	1.00649
H	-2.82954	-1.42157	2.02113
C	4.35838	-0.34351	-0.19484
O	3.73978	0.53716	-0.77518
O	5.64940	-0.56817	-0.33141
C	-3.57179	-0.17304	0.42683
O	-4.03824	-0.03874	-0.69127
O	-3.28477	0.80476	1.27201
C	-4.42185	-2.47617	0.97815
H	-4.76607	-2.55700	-0.05752
H	-4.10257	-3.47161	1.30637
C	3.37013	-0.60225	2.09757
H	2.90306	-1.31863	2.77963
H	2.63405	0.17655	1.88439
C	6.40355	0.35613	-1.20182
H	7.26558	-0.24310	-1.49413
H	5.78147	0.56638	-2.0727
C	-3.63843	2.17219	0.85660
H	-2.98038	2.79034	1.46712
H	-3.38024	2.28429	-0.19469
C	-5.08814	2.45987	1.12594
C	-5.50141	2.82380	2.41257
C	-6.04005	2.36202	0.10370
C	-6.42345	-1.62447	4.10688
H	-4.72509	-2.86780	3.65526
C	-7.43056	-0.49188	2.22663
H	-6.50968	-0.83764	0.3129
C	-7.39817	-0.76661	3.59379
H	-6.39771	-1.84884	5.16916
H	-8.17952	0.17961	1.82252
H	-8.13129	-0.31696	4.25674
C	4.61628	-0.01667	2.72114
C	4.88269	1.35283	2.61786
C	5.53618	-0.84461	3.37584
C	6.04591	1.88622	3.17154
H	4.18230	1.99880	2.09888
C	6.70429	-0.31340	3.92053
H	5.33334	-1.90982	3.46822
C	6.96007	1.05551	3.81900
H	6.24308	2.94819	3.08055
H	7.40926	-0.96452	4.42918
H	7.86822	1.47242	4.24412
I	0.12121	-0.36779	-1.23278
O	1.08027	0.90652	0.10680
B	1.97806	1.78899	-0.45537
F	2.61520	2.61133	0.37620
F	1.92145	2.09310	-1.75874
C	-0.93222	-3.03632	-2.62163
H	-1.84660	-3.39462	-2.14994
C	-1.03346	-1.91816	-3.39593
H	-0.17770	-1.53654	-3.94372
C	0.26398	-3.80231	-2.30000
C	0.13633	-4.89073	-1.41717
C	1.54504	-3.46615	-2.78115
C	1.25357	-5.61673	-1.01511
H	-0.84743	-5.15206	-1.03797
C	2.65865	-4.18992	-2.37671
H	1.67024	-2.63052	-3.46251
C	2.51760	-5.26518	-1.49095

C	-6.84288	3.09897	2.67301
H	-4.76777	2.89549	3.21144
C	-7.38093	2.64722	0.35967
H	-5.72306	2.06174	-0.88807
C	-7.78351	3.01982	1.64399
H	-7.15246	3.38491	3.67369
H	-8.11088	2.58116	-0.44204
H	-8.82668	3.24831	1.84241
C	6.81411	1.60964	-0.48488
C	8.02450	1.64616	0.21643
C	6.00933	2.75569	-0.52781
C	8.43584	2.81470	0.85503
H	8.64964	0.75793	0.25610
C	6.41898	3.92347	0.11402
H	5.06668	2.73029	-1.06307
C	7.63509	3.95705	0.80006
H	9.38128	2.83559	1.38865
H	5.79300	4.80974	0.06988
H	7.95896	4.87169	1.28837
C	-5.50556	-1.92545	1.87955
C	-5.48193	-2.19745	3.25293
C	-6.48871	-1.06668	1.37400
H	1.13947	-6.45252	-0.33207
H	3.64122	-3.91917	-2.75018
H	3.39199	-5.82865	-1.17952
C	-2.37259	-1.30666	-3.76075
H	-3.17846	-1.96012	-3.41816
H	-2.41927	-1.26479	-4.85670
N	-2.66913	0.02126	-3.22911
H	-3.42635	0.09208	-2.55715
C	-1.80783	1.06458	-3.34612
O	-0.66700	0.91550	-3.81133
C	-2.27756	2.37328	-2.80529
C	-1.32040	3.24465	-2.26607
C	-3.62752	2.75013	-2.82684
C	-1.71773	4.46269	-1.71856
H	-0.27362	2.96171	-2.28671
C	-4.01755	3.98072	-2.29979
H	-4.36610	2.09318	-3.27548
C	-3.06555	4.83199	-1.73420
H	-0.97543	5.12942	-1.29031
H	-5.06201	4.27412	-2.32555
H	-3.37294	5.78578	-1.31616

Int4

C	0.36845	1.56654	-4.14930
C	-0.90324	1.69099	-3.59484
C	-1.06357	1.59209	-2.20919
C	0.06614	1.39396	-1.41813
C	1.34849	1.23719	-1.96722
C	1.49213	1.34815	-3.35496
H	0.49008	1.65022	-5.22471
H	-1.77038	1.87969	-4.21793
H	2.47384	1.26366	-3.80308
O	2.33830	1.03802	-1.08857
O	-2.28563	1.77022	-1.61576
C	3.61185	0.50206	-1.46383
H	4.14614	1.21867	-2.09599
C	-3.33994	0.85340	-1.97867
H	-3.22344	0.53234	-3.01555
C	4.33371	0.40424	-0.12230
O	3.78707	0.09103	0.91247
O	5.62912	0.70960	-0.23910
C	-3.25451	-0.36396	-1.05469
O	-3.20958	-0.24553	0.15906
O	-3.28096	-1.49126	-1.73441
C	-4.66755	1.59080	-1.77387
H	-4.67631	1.99085	-0.75453
H	-4.68832	2.44353	-2.46093
C	3.48435	-0.88133	-2.13979
H	3.20803	-0.73573	-3.18861
C	-7.42071	-0.96975	-1.15135
H	-5.92387	-0.00397	0.04946
C	-7.97296	-1.10664	-2.42526
H	-7.89667	-0.44884	-4.47828
H	-7.78951	-1.57005	-0.32730
H	-8.78937	-1.80337	-2.59245
C	4.74655	-1.70462	-2.04490
C	4.80576	-2.76947	-1.13871
C	5.87016	-1.40660	-2.82525
C	5.97307	-3.52344	-1.01960
H	3.93371	-3.00176	-0.53610
C	7.03850	-2.15584	-2.70064
H	5.82884	-0.58265	-3.53512
C	7.09146	-3.21660	-1.79374
H	6.01181	-4.34194	-0.30885
H	7.90545	-1.91453	-3.31011
H	8.00230	-3.79878	-1.68875
I	-0.10186	1.22703	0.69738
O	1.00350	-1.26394	0.62574
B	1.87268	-1.47911	1.61499
F	2.74783	-2.49239	1.64605
F	1.83640	-0.69426	2.70691
C	-0.00863	4.28913	0.11030
H	-0.44303	4.21621	-0.89298
C	-0.72790	3.32057	1.04374
H	-0.38989	3.44117	2.07307

H	2.66410	-1.41606	-1.65916
C	6.41549	0.62089	0.99160
H	7.26079	1.28174	0.79402
H	5.80717	1.03015	1.80070
C	-3.16505	-2.74456	-0.97492
H	-2.75938	-3.43544	-1.71366
H	-2.42853	-2.60525	-0.18842
C	-4.50085	-3.19895	-0.45718
C	-5.38791	-3.88653	-1.29359
C	-4.86362	-2.96118	0.87387
C	-6.61289	-4.34280	-0.80971
H	-5.11128	-4.06797	-2.32904
C	-6.08700	-3.42208	1.36162
H	-4.18639	-2.41526	1.52199
C	-6.9618	-4.11699	0.52368
H	-7.29028	-4.87971	-1.46787
H	-6.35009	-3.24825	2.40059
H	-7.90961	-4.48414	0.90841
C	6.88504	-0.77391	1.30745
C	8.16388	-1.18049	0.91226
C	6.07536	-1.66437	2.02649
C	8.63946	-2.44915	1.24226
H	8.79223	-0.49654	0.34696
C	6.55006	-2.93298	2.35372
H	5.07307	-1.36964	2.31225
C	7.83384	-3.32581	1.96960
H	9.63677	-2.75055	0.93432
H	5.91136	-3.61494	2.90739
H	8.20311	-4.31358	2.23186
C	-5.8534	0.67959	-1.99427
C	-6.41431	0.53697	-3.26808
C	-6.36804	-0.08237	-0.93794
C	-7.46969	-0.34940	-3.48421
H	-6.02336	1.12601	-4.09488
C	1.48854	4.15090	0.01512
C	2.09896	4.24850	-1.23851
C	2.28258	3.91727	1.14440
C	3.47859	4.09275	-1.37038
H	1.48641	4.42414	-2.11874
C	3.65932	3.75070	1.01434
H	1.82749	3.84602	2.12799
C	4.26065	3.83548	-0.24416
H	3.93936	4.16384	-2.35153
H	4.26193	3.54835	1.89443
H	5.33187	3.69193	-0.34635
C	-2.25571	3.45958	0.99551
H	-2.58432	3.57024	-0.0381
H	-2.48711	4.38171	1.53783
N	-3.00037	2.36404	1.57848
H	-3.23374	1.58423	0.96250
C	-2.81547	2.04058	2.90139
O	-2.03846	2.66417	3.62660
C	-3.61747	0.87867	3.38726
C	-3.1006	0.09897	4.42847
C	-4.87011	0.56830	2.84279
C	-3.81652	-0.99938	4.89929
H	-2.13836	0.36594	4.85270
C	-5.59568	-0.51588	3.33082
H	-5.28382	1.18955	2.05560
C	-5.06526	-1.30670	4.35253
H	-3.40462	-1.61320	5.69470
H	-6.56941	-0.74645	2.90980
H	-5.62608	-2.15828	4.72784
F	-0.35163	5.56157	0.60520
B	0.38579	-2.23580	-0.31813
F	1.37355	-2.91743	-1.03049
F	-0.40796	-1.46297	-1.19584
F	-0.41469	-3.11752	0.40771

Int4^{+}

C	0.80528	-2.37179	3.00342
C	-0.43211	-2.10924	2.41529
C	-0.6318	-0.89053	1.75902
C	0.42423	0.03443	1.73894
C	1.66342	-0.22136	2.33937
C	1.85406	-1.45496	2.97258
H	0.95497	-3.32371	3.50286
H	-1.21675	-2.85371	2.45131
H	2.80209	-1.68957	3.44067
O	2.59061	0.76586	2.28164
O	-1.75492	-0.51582	1.10693
C	3.98357	0.41804	2.26915
H	4.28609	-0.01524	3.22656
C	-2.92158	-1.35460	1.12177
C	-4.21394	-4.37892	0.94909
C	-5.22564	-2.93256	-0.69583
C	-5.39898	-5.10847	1.03977
H	-3.35450	-4.66815	1.55069
C	-6.41115	-3.66111	-0.60776
H	-5.16682	-2.07904	-1.36466
C	-6.50107	-4.74837	0.26191
H	-5.46029	-5.95891	1.71244
H	-7.26418	-3.37013	-1.21042
H	-7.42439	-5.31576	0.33112
C	6.21925	1.49788	1.81453
C	6.75878	1.40566	0.52659
C	7.06155	1.32492	2.91883
C	8.11714	1.15294	0.34390

H	-3.06839	-1.77858	2.11792
C	4.23804	-0.57950	1.13763
O	3.73572	-0.45581	0.03539
O	5.04632	-1.56189	1.51004
C	-4.06632	-0.38519	0.82244
O	-4.13512	0.25294	-0.21521
O	-4.94281	-0.32929	1.80933
C	-2.85694	-2.44838	0.04701
H	-2.73737	-1.95661	-0.92264
H	-1.96505	-3.05879	0.21711
C	4.73807	1.72729	2.00270
H	4.53799	2.40226	2.84173
H	4.29952	2.17227	1.10382
C	5.36226	-2.57585	0.49210
H	5.68107	-3.42785	1.09291
H	4.43494	-2.81598	-0.03095
C	-6.10154	0.55857	1.61754
H	-6.42606	0.75424	2.63973
H	-5.74575	1.48156	1.15647
C	-7.17622	-0.10374	0.80383
C	-8.03285	-1.03513	1.40190
C	-7.33240	0.19986	-0.55349
C	-9.03845	-1.64805	0.65715
H	-7.91195	-1.27714	2.45452
C	-8.34031	-0.41027	-1.30010
H	-6.66133	0.91436	-1.01979
C	-9.19671	-1.33221	-0.69434
H	-9.70206	-2.36508	1.13078
H	-8.46228	-0.16187	-2.35027
H	-9.98771	-1.80053	-1.27285
C	6.43792	-2.13309	-0.45808
C	7.78478	-2.28907	-0.11338
C	6.10610	-1.58359	-1.70302
C	8.78932	-1.90992	-1.00228
H	8.04692	-2.71054	0.85342
C	7.11040	-1.19974	-2.59036
H	5.06256	-1.46302	-1.97349
C	8.45329	-1.36581	-2.24350
H	9.83181	-2.04119	-0.72854
H	6.84495	-0.78420	-3.55845
H	9.23519	-1.07780	-2.94031
C	-4.11529	-3.28488	0.08105
H	6.11054	1.52543	-0.33721
C	8.42014	1.06651	2.73870
H	6.65406	1.40260	3.92439
C	8.94981	0.98016	1.44975
H	8.51900	1.07807	-0.66032
H	9.06532	0.93997	3.60316
H	10.00829	0.78250	1.30848
I	0.14088	1.83824	0.66755
C	1.02835	-0.35365	-1.42715
H	1.83196	-0.31107	-0.69162
C	0.25404	0.95068	-1.48183
H	0.83629	1.76772	-1.89650
C	1.61171	-0.61576	-2.79899
C	0.94476	-1.41917	-3.72768
C	2.83177	-0.01596	-3.13515
C	1.50039	-1.62294	-4.99107
H	0.01279	-1.90128	-3.45274
C	3.37952	-0.21873	-4.40068
H	3.35923	0.58259	-2.39812
C	2.71342	-1.02069	-5.33086
H	0.98740	-2.25568	-5.70888
H	4.32758	0.24394	-4.65825
H	3.14165	-1.18074	-6.31555
C	-1.15046	0.84138	-2.07748
H	-1.52897	-0.16043	-1.88637
H	-0.98479	0.94544	-3.16021
N	-2.16455	1.76015	-1.6264
H	-2.98982	1.32325	-1.22620
C	-1.94837	3.09103	-1.44499
O	-0.81393	3.57856	-1.58150
C	-3.11844	3.89646	-0.99501
C	-2.86289	5.15330	-0.43042
C	-4.44202	3.44016	-1.09796
C	-3.91273	5.93381	0.04744
H	-1.83651	5.50032	-0.37989
C	-5.49010	4.22344	-0.61981
H	-4.66251	2.48875	-1.56843
C	-5.22669	5.46790	-0.04129
H	-3.70833	6.90567	0.48598
H	-6.51279	3.86898	-0.71058
H	-6.04529	6.07770	0.32920
F	0.16131	-1.38513	-1.05394

Int5

C	-1.94848	0.99291	1.37307
H	-2.04893	0.73655	2.42398
C	-1.20186	0.22185	0.43886
H	-0.78859	-0.72783	0.76260
C	-2.87960	0.06857	0.40337
C	-3.58701	0.74542	-0.62912
H	-0.66882	0.13568	-1.63360
N	0.92025	0.84885	-0.49299
H	1.34268	1.72892	-0.23599
C	1.63695	-0.33254	-0.39891
O	1.03552	-1.40440	-0.46403
C	3.10446	-0.22146	-0.20812

C	-3.35516	-1.18350	0.88564	C	3.78000	-1.35708	0.26085
C	-4.71353	0.16307	-1.17884	C	3.82600	0.94869	-0.48987
H	-3.25462	1.72155	-0.96226	C	5.15501	-1.31527	0.46752
C	-4.48478	-1.75117	0.32824	H	3.20967	-2.25863	0.45536
H	-2.81366	-1.69540	1.67596	C	5.20279	0.98541	-0.28618
C	-5.15820	-1.08020	-0.70401	H	3.33189	1.82108	-0.90872
H	-5.25859	0.67020	-1.96746	C	5.86682	-0.14379	0.19812
H	-4.84684	-2.70988	0.68297	H	5.67370	-2.19489	0.83551
H	-6.04528	-1.52969	-1.14022	H	5.75893	1.88902	-0.51481
C	-0.49360	0.79671	-0.78145	H	6.94046	-0.11280	0.35667
H	-0.84693	1.80060	-1.01780	F	-2.10110	2.31034	1.14177

Int6

C	-0.59363	1.03322	0.39725	N	0.78271	-0.97250	-0.79535
C	-1.30209	-0.30956	0.32111	C	1.45370	-0.10255	-0.06863
H	-1.05102	-0.83396	1.25058	O	0.82512	0.82691	0.62605
C	-2.81054	-0.20493	0.21766	C	2.90369	-0.10669	0.02743
C	-3.42883	0.47388	-0.84142	C	3.56240	1.07526	0.41499
C	-3.60239	-0.81568	1.19669	C	3.64682	-1.26707	-0.26565
C	-4.81956	0.53621	-0.91426	C	4.95004	1.09588	0.48683
H	-2.83294	0.96730	-1.60165	H	2.98555	1.96511	0.63744
C	-4.99293	-0.75230	1.12124	C	5.03206	-1.23579	-0.18522
H	-3.13084	-1.34459	2.02133	H	3.15150	-2.20347	-0.50708
C	-5.60347	-0.07627	0.06455	C	5.68373	-0.05433	0.18559
H	-5.28999	1.06679	-1.73615	H	5.46043	2.00856	0.77520
H	-5.59649	-1.23009	1.88648	H	5.60549	-2.13174	-0.39743
H	-6.68609	-0.02597	0.00412	H	6.76727	-0.03463	0.24628
C	-0.68791	-1.07490	-0.85424	H	-0.91244	1.67430	1.21733
H	-1.02587	-0.66846	-1.81134	F	-0.69330	1.71287	-0.78354
H	-0.95897	-2.13175	-0.80873	H	1.32779	-1.58730	-1.38898

Int7

C	-0.55223	1.09385	0.24322	N	0.82177	-1.16025	-0.63183
C	-1.27673	-0.24293	0.36189	C	1.42203	-0.17855	-0.09278
H	-1.02462	-0.62040	1.36093	O	0.82077	0.93939	0.47001
C	-2.78168	-0.16271	0.24854	C	2.89611	-0.11617	0.01647
C	-3.41303	0.39847	-0.87145	C	3.54293	1.00770	0.54756
C	-3.57893	-0.69538	1.26957	C	3.65811	-1.20949	-0.42215
C	-4.80449	0.42582	-0.96004	C	4.93420	1.03451	0.63972
H	-2.81335	0.82564	-1.66638	H	2.95519	1.85411	0.88179
C	-4.97072	-0.67049	1.18130	C	5.04548	-1.17686	-0.32977
H	-3.10210	-1.13686	2.14155	H	3.13998	-2.06942	-0.83129
C	-5.58863	-0.10838	0.06388	C	5.68817	-0.05499	0.20262
H	-5.27693	0.86752	-1.83305	H	5.42880	1.90937	1.05197
H	-5.57010	-1.08954	1.98470	H	5.62874	-2.02711	-0.67169
H	-6.67226	-0.08652	-0.00883	H	6.77197	-0.03144	0.27486

TS1

C	-0.79236	0.82341	4.62291	C	6.28973	-3.25393	2.04059
C	0.53071	0.77409	4.18268	H	5.74590	-1.42685	1.04592

C	0.80322	0.79987	2.81260
C	-0.25631	0.86228	1.90498
C	-1.58614	0.92118	2.34397
C	-1.84877	0.90749	3.71855
H	-1.00187	0.81588	5.68800
H	1.35807	0.73504	4.88320
H	-2.87373	0.97636	4.06600
O	-2.53943	1.04670	1.39433
O	2.09911	0.83362	2.36223
C	-3.74060	0.25655	1.42735
H	-4.49469	0.75372	2.04587
C	2.83477	-0.41603	2.43377
H	2.29660	-1.11272	3.08029
C	-4.20704	0.25336	-0.02910
O	-3.46179	0.06207	-0.96871
O	-5.51826	0.46398	-0.12485
C	2.88391	-0.97013	1.00930
O	3.56139	-0.47569	0.12951
O	2.06919	-2.01167	0.86573
C	4.24504	-0.16154	2.95870
H	4.74694	0.52820	2.27348
H	4.16595	0.32691	3.93563
C	-3.50844	-1.19231	1.89755
H	-3.16935	-1.17080	2.93762
H	-2.69684	-1.60769	1.29565
C	-6.0796	0.41803	-1.47831
H	-6.98642	1.01747	-1.39166
H	-5.37248	0.90906	-2.14872
C	2.03424	-2.62968	-0.45925
H	1.11687	-3.21685	-0.43135
H	1.94034	-1.84168	-1.20706
C	3.25829	-3.47174	-0.69138
C	3.40797	-4.69358	-0.02509
C	4.26734	-3.03189	-1.55369
C	4.54879	-5.46940	-0.22229
H	2.62920	-5.03315	0.65307
C	5.40510	-3.81178	-1.75983
H	4.16182	-2.07829	-2.05556
C	5.54786	-5.03148	-1.09549
H	4.65721	-6.41570	0.29979
H	6.17671	-3.46486	-2.44128
H	6.43319	-5.64041	-1.25747
C	-6.39151	-0.98377	-1.92712
C	-7.66841	-1.51442	-1.71525
C	-5.4228	-1.76526	-2.57164
C	-7.98358	-2.80057	-2.15149
H	-8.4202	-0.91425	-1.20894
C	-5.73567	-3.05373	-3.00217
H	-4.42731	-1.36564	-2.72554
C	-7.01713	-3.57129	-2.79989
C	6.20313	-4.00461	3.21348
H	5.44925	-4.06675	5.23389
H	6.80883	-3.64923	1.17395
H	6.66872	-4.98445	3.26982
C	-4.76554	-2.02143	1.77883
C	-4.91163	-2.94232	0.73591
C	-5.81466	-1.85473	2.69133
C	-6.08394	-3.68770	0.61387
H	-4.10961	-3.06549	0.01623
C	-6.99079	-2.59227	2.56464
H	-5.70776	-1.14524	3.50968
C	-7.12694	-3.51203	1.52282
H	-6.18679	-4.39033	-0.20541
H	-7.79707	-2.45299	3.27960
H	-8.04228	-4.08814	1.42041
I	0.09742	0.88649	-0.18944
O	-0.87405	-1.12278	-0.14029
B	-1.4891	-1.45628	-1.28244
F	-2.28046	-2.54295	-1.33655
F	-1.24582	-0.85093	-2.45974
C	0.99539	3.52636	0.89691
H	1.65378	3.24201	1.71696
C	1.37150	3.11305	-0.37922
H	0.80789	3.46891	-1.23588
C	-0.21374	4.20702	1.26248
C	-0.62765	4.14794	2.61232
C	-1.01013	4.89230	0.31555
C	-1.83361	4.71372	3.00278
H	-0.00607	3.63020	3.33719
C	-2.20785	5.46439	0.72219
H	-0.65020	5.01506	-0.70135
C	-2.62706	5.36792	2.05504
H	-2.15476	4.65134	4.03800
H	-2.81376	6.00426	0.00132
H	-3.56768	5.81909	2.35874
C	2.81294	2.76402	-0.63207
H	3.28956	2.44701	0.30007
H	3.25209	3.71129	-0.97404
N	3.10003	1.72632	-1.62316
H	3.96450	1.23201	-1.45892
C	2.28407	1.19121	-2.55605
O	1.08124	1.48244	-2.67281
C	2.89010	0.12106	-3.40913
C	2.04334	-0.90524	-3.85103
C	4.24480	0.10621	-3.76512
C	2.55730	-1.95164	-4.61363
H	0.99286	-0.86941	-3.58127
C	4.75115	-0.93449	-4.54424
H	4.89501	0.92295	-3.46523
C	3.91038	-1.96840	-4.96142

H	-8.98067	-3.19907	-1.98732	H	1.90207	-2.75383	-4.93982
H	-4.97724	-3.65081	-3.50002	H	5.79875	-0.93340	-4.83079
H	-7.26148	-4.57190	-3.14611	H	4.30766	-2.78301	-5.55981
C	4.99059	-1.47608	3.06341	F	1.87787	5.79908	-0.39138
C	4.91110	-2.23687	4.23635	B	1.82741	5.90229	-1.81010
C	5.69016	-1.99716	1.96693	F	0.62074	5.25729	-2.24206
C	5.51600	-3.49118	4.31488	F	2.91951	5.18477	-2.33960
H	4.37277	-1.84137	5.09513	F	1.83590	7.21974	-2.20514

***ent*-TS1**

C	-2.93888	1.97099	3.96996	C	3.18645	-4.57309	3.38997
C	-1.63048	1.52211	4.10107	H	3.76834	-2.62870	4.10987
C	-0.89513	1.10799	2.98320	C	2.14363	-5.36439	2.90747
C	-1.51982	1.14596	1.72304	H	0.03930	-5.42234	2.41232
C	-2.83041	1.63125	1.58656	H	4.19255	-4.97707	3.45320
C	-3.54125	2.03545	2.71639	H	2.33409	-6.38914	2.60100
H	-3.48809	2.28953	4.85047	C	-5.22843	-1.49799	-0.06396
H	-1.13053	1.49758	5.06264	C	-4.75530	-2.80018	-0.25727
H	-4.54720	2.41972	2.58652	C	-6.57807	-1.22482	-0.31919
O	-3.35877	1.85360	0.34377	C	-5.61313	-3.80556	-0.70681
O	0.41194	0.81252	3.21303	H	-3.71402	-3.02895	-0.05359
C	-4.23726	0.89295	-0.28644	C	-7.43292	-2.22447	-0.77941
H	-5.24047	1.33700	-0.30927	H	-6.96751	-0.22100	-0.16485
C	0.97186	-0.43625	2.77769	C	-6.95167	-3.52139	-0.97709
H	0.22103	-1.01013	2.22998	H	-5.22802	-4.81153	-0.85049
C	-3.75240	0.80363	-1.73955	H	-8.47569	-1.99345	-0.98003
O	-2.83749	1.45637	-2.19790	H	-7.61804	-4.30268	-1.33224
O	-4.48486	-0.07206	-2.43031	I	-0.52634	0.47165	-0.03893
C	2.12106	-0.11904	1.81971	O	-1.34766	-1.63339	0.86589
O	2.50540	0.99826	1.53368	B	-0.79288	-2.74132	0.42304
O	2.60935	-1.24745	1.31209	F	-1.37120	-3.96027	0.55500
C	1.39580	-1.25690	4.01299	F	0.42032	-2.78212	-0.19485
H	2.26640	-0.79275	4.48853	C	-0.51879	3.63172	-0.15596
H	0.56191	-1.18272	4.72128	H	-1.53277	3.73731	-0.53398
C	-4.28122	-0.44837	0.47100	C	0.22255	2.50442	-0.75703
H	-4.57172	-0.20418	1.50072	H	1.24657	2.47249	-0.3988
H	-3.27612	-0.87355	0.53267	C	-0.35636	4.00688	1.25144
C	-3.94200	-0.50534	-3.71905	C	-1.40911	4.69849	1.87489
H	-4.81830	-0.86653	-4.25814	C	0.81351	3.72317	1.97451
H	-3.51606	0.36060	-4.22773	C	-1.30035	5.09567	3.20178
C	3.53130	-1.11964	0.19595	H	-2.31471	4.90728	1.31265
H	2.91990	-0.85824	-0.67132	C	0.91589	4.12219	3.30291
H	4.22566	-0.30038	0.39232	H	1.62879	3.17401	1.52590
C	4.24179	-2.42836	0.00507	C	-0.13381	4.80854	3.91613
C	3.54242	-3.63953	0.06717	H	-2.12099	5.62059	3.68081
C	5.61066	-2.43837	-0.28065	H	1.81465	3.88256	3.86136
C	4.21260	-4.84220	-0.15004	H	-0.04723	5.11532	4.95473
H	2.48324	-3.63485	0.29391	C	0.18520	2.46062	-2.30219
C	6.27695	-3.64278	-0.51250	H	-0.14029	3.42466	-2.68746
H	6.15395	-1.49819	-0.32669	H	-0.51054	1.70982	-2.67371

C	5.57817	-4.84898	-0.44433
H	3.66383	-5.77771	-0.08678
H	7.34097	-3.63892	-0.73349
H	6.09547	-5.78910	-0.61601
C	-2.93167	-1.59216	-3.47869
C	-3.36516	-2.90566	-3.26832
C	-1.57053	-1.29065	-3.34360
C	-2.45519	-3.89901	-2.90957
H	-4.42252	-3.13969	-3.34892
C	-0.65904	-2.28074	-2.97917
H	-1.22446	-0.27542	-3.50364
C	-1.10383	-3.58568	-2.75466
H	-2.80361	-4.91211	-2.73045
H	0.38303	-2.01899	-2.83828
H	-0.39919	-4.34863	-2.43999
C	1.66384	-2.70550	3.67015
C	0.62022	-3.51253	3.19884
C	2.94681	-3.25053	3.76352
C	0.85750	-4.82946	2.80842
H	-0.3837	-3.10369	3.11728

N	1.51728	2.21203	-2.81788
H	2.07390	3.03242	-3.02236
C	2.10841	1.02055	-2.57353
O	1.44168	0.04207	-2.18908
C	3.58700	0.93892	-2.73202
C	4.15824	-0.28812	-3.09195
C	4.40979	2.02949	-2.41617
C	5.54204	-0.41818	-3.16520
H	3.50737	-1.13208	-3.29397
C	5.79597	1.89118	-2.48238
H	3.97642	2.96048	-2.06150
C	6.36213	0.67300	-2.86342
H	5.98008	-1.37293	-3.43863
H	6.43275	2.73137	-2.22230
H	7.44240	0.56932	-2.91251
F	1.74500	5.04330	-2.65677
B	1.64697	5.33713	-1.31596
F	2.37542	4.47897	-0.53590
F	0.08492	4.97960	-0.99139
F	1.72441	6.64137	-0.99892

TS2

C	-0.79234	-2.25235	-3.06811
C	0.43158	-2.00758	-2.44793
C	0.64505	-0.77907	-1.81257
C	-0.37149	0.18853	-1.83617
C	-1.60772	-0.07201	-2.43979
C	-1.81630	-1.30859	-3.06310
H	-0.95301	-3.20625	-3.56027
H	1.19882	-2.77101	-2.44683
H	-2.76285	-1.52511	-3.54354
O	-2.54518	0.91056	-2.37897
O	1.76016	-0.44508	-1.11651
C	-3.92216	0.53724	-2.30406
H	-4.27848	0.14978	-3.26344
C	2.93358	-1.26364	-1.18715
H	3.07180	-1.64729	-2.20099
C	-4.11090	-0.53289	-1.22610
O	-3.55315	-0.49404	-0.14213
O	-4.94087	-1.48715	-1.62734
C	4.06210	-0.28837	-0.86049
O	4.09726	0.34213	0.18437
O	4.96127	-0.21474	-1.82519
C	2.91202	-2.39934	-0.15227
H	2.75500	-1.94467	0.82988
H	2.05301	-3.04587	-0.35280
C	-4.68455	1.81430	-1.92193
H	-4.54181	2.53666	-2.73254
H	-4.20059	2.22041	-1.02822
C	-5.21299	-2.57684	-0.68515

C	4.39467	-4.20274	-1.11878
C	5.26940	-2.82300	0.65621
C	5.61600	-4.87052	-1.20427
H	3.57581	-4.48838	-1.77595
C	6.49067	-3.49011	0.57405
H	5.14348	-2.01565	1.37180
C	6.66741	-4.51289	-0.35813
H	5.74534	-5.67037	-1.92748
H	7.30494	-3.19904	1.22797
H	7.61943	-5.03113	-0.42396
C	-6.14861	1.54892	-1.66374
C	-6.61980	1.41345	-0.35298
C	-7.04525	1.38658	-2.72618
C	-7.96208	1.13151	-0.10588
H	-5.92944	1.52784	0.47864
C	-8.38765	1.09739	-2.48261
H	-6.69262	1.49676	-3.74916
C	-8.84837	0.96999	-1.17088
H	-8.31042	1.02443	0.91550
H	-9.07452	0.97982	-3.31567
H	-9.89427	0.74854	-0.98003
I	-0.02579	2.06786	-0.90745
C	-0.82833	-0.71308	1.30828
H	-1.50806	-0.57248	0.46780
C	-0.17281	0.46941	1.84674
H	-0.73538	1.39112	1.91806
C	-1.58454	-0.88781	2.64022
C	-1.05924	-1.73083	3.63773

H	-5.51816	-3.39428	-1.33905	C	-2.78895	-0.18665	2.85064
H	-4.27408	-2.82987	-0.18948	C	-1.76698	-1.90627	4.82050
C	6.09702	0.69353	-1.60633	H	-0.13452	-2.26730	3.45889
H	6.43499	0.90555	-2.62090	C	-3.48102	-0.36567	4.04244
H	5.71268	1.60479	-1.14522	H	-3.18358	0.44838	2.06544
C	7.17371	0.04936	-0.78049	C	-2.97139	-1.22142	5.02542
C	8.06286	-0.85435	-1.37376	H	-1.38497	-2.57502	5.58480
C	7.30466	0.34679	0.58083	H	-4.41699	0.15845	4.20521
C	9.07557	-1.44495	-0.62072	H	-3.51370	-1.35591	5.95616
H	7.96192	-1.09186	-2.42947	C	1.15832	0.44577	2.55055
C	8.31974	-0.2409	1.33575	H	1.60758	-0.54527	2.50190
H	6.61142	1.04160	1.04449	H	0.97409	0.71770	3.59737
C	9.20862	-1.13457	0.73466	N	2.07592	1.40283	1.96801
H	9.76452	-2.13995	-1.09108	H	2.73409	1.04801	1.27319
H	8.42229	0.00427	2.38878	C	1.77109	2.73917	2.04363
H	10.00541	-1.58483	1.31962	O	0.73028	3.11121	2.59267
C	-6.28819	-2.22843	0.30448	C	2.72945	3.68197	1.40361
C	-7.63601	-2.35862	-0.0461	C	2.25081	4.95233	1.05474
C	-5.95335	-1.79053	1.59097	C	4.06573	3.34667	1.14364
C	-8.63677	-2.06436	0.87842	C	3.09025	5.86662	0.42343
H	-7.9016	-2.69123	-1.04587	H	1.22238	5.20418	1.29134
C	-6.95303	-1.49385	2.51589	C	4.90561	4.26834	0.52189
H	-4.90813	-1.69046	1.86201	H	4.45333	2.37831	1.43414
C	-8.29700	-1.63408	2.16245	C	4.41795	5.52449	0.15323
H	-9.68014	-2.17337	0.59870	H	2.71357	6.84677	0.14769
H	-6.68539	-1.16723	3.51715	H	5.94456	4.01107	0.33581
H	-9.07649	-1.41350	2.88593	H	5.07406	6.23975	-0.33381
C	4.20904	-3.17329	-0.18830	F	0.03576	-1.75807	1.07359

TS3

C	-1.54474	-0.51321	1.21597	N	0.86626	-0.68037	-0.85477
C	-1.45152	-1.01838	-0.11737	C	1.61922	0.37586	-0.34285
H	-1.55235	-2.10195	-0.15741	O	1.07601	1.44160	-0.07459
C	-2.90417	-0.38754	0.04386	C	3.07047	0.12209	-0.14943
C	-3.14144	0.97383	-0.28464	C	3.90637	1.23912	-0.01305
C	-4.00087	-1.26391	0.26309	C	3.61901	-1.16748	-0.08273
C	-4.44208	1.42105	-0.44182	C	5.27568	1.06840	0.16402
H	-2.31002	1.66364	-0.38006	H	3.46358	2.22821	-0.05183
C	-5.29470	-0.80115	0.10655	C	4.98923	-1.33465	0.10165
H	-3.81660	-2.30080	0.52978	H	2.98173	-2.04617	-0.13275
C	-5.51343	0.53782	-0.25099	C	5.81883	-0.21753	0.21882
H	-4.63008	2.45784	-0.69868	H	5.92042	1.93619	0.26137
H	-6.13526	-1.46951	0.25854	H	5.40863	-2.33404	0.16194
H	-6.53062	0.89906	-0.36836	H	6.88720	-0.34987	0.35996
C	-0.51546	-0.43528	-1.18536	H	-1.86474	-1.11112	2.06456
H	-0.64777	0.64336	-1.26838	F	-1.04046	0.64938	1.55585
H	-0.77483	-0.89832	-2.14059	H	1.35063	-1.42541	-1.33492

1b

C	-0.55223	1.09385	0.24322	N	0.82177	-1.16025	-0.63183

C	-1.27673	-0.24293	0.36189	C	1.42203	-0.17855	-0.09278
H	-1.02462	-0.62040	1.36093	O	0.82077	0.93939	0.47001
C	-2.78168	-0.16271	0.24854	C	2.89611	-0.11617	0.01647
C	-3.41303	0.39847	-0.87145	C	3.54293	1.00770	0.54756
C	-3.57893	-0.69538	1.26957	C	3.65811	-1.20949	-0.42215
C	-4.80449	0.42582	-0.96004	C	4.93420	1.03451	0.63972
H	-2.81335	0.82564	-1.66638	H	2.95519	1.85411	0.88179
C	-4.97072	-0.67049	1.18130	C	5.04548	-1.17686	-0.32977
H	-3.10210	-1.13686	2.14155	H	3.13998	-2.06942	-0.83129
C	-5.58863	-0.10838	0.06388	C	5.68817	-0.05499	0.20262
H	-5.27693	0.86752	-1.83305	H	5.42880	1.90937	1.05197
H	-5.57010	-1.08954	1.98470	H	5.62874	-2.02711	-0.67169
H	-6.67226	-0.08652	-0.00883	H	6.77197	-0.03144	0.27486
C	-0.62999	-1.18012	-0.67286	H	-0.89908	1.85105	0.94916
H	-0.95615	-0.90150	-1.68319	F	-0.72240	1.60927	-1.03731
H	-0.97056	-2.20734	-0.50635				

	Thermal Correction	E	G		Thermal Correction	E	G
ArI	0.551995	-2217.81	-2217.26	**TS2**	0.802339	-3066.5	-3065.69
ArIO	0.552251	-2292.96	-2292.41	**Int6**	0.229117	-848.658	-848.429
$ArIOBF_3$	0.558017	-2617.68	-2617.13	**Int5**	0.802699	-3066.5	-3065.7
OEt_2	0.107459	-233.758	-233.65	**TS3**	0.232313	-848.654	-848.422
$BF_3 \cdot OEt_2$	0.11696	-558.454	-558.337	**TS3**_BF_2OBF_3	0.247245	-1473.59	-1473.35
BF_3	-0.011909	-324.669	-324.681	**1b**	0.222764	-848.27	-848.047
BF_4^-	-0.010675	-424.768	-424.779	**Int7**	0.236663	-848.721	-848.484
**1a**	0.226638	-749.01	-748.783	**Int7**_BF_2OBF_3	0.252257	-1473.65	-1473.4
**Int3**	0.812134	-3366.71	-3365.9	BF_2O^-	-0.013434	-300.173	-300.186
**Int3**$^+$	0.815903	-3266.64	-3265.83	$BF_2OBF_3^-$	-0.007616	-624.916	-624.924
**TS1**	0.823904	-3691.41	-3690.58	BF_2OH	-0.001561	-300.65	-300.651
***ent*-TS1**	0.822655	-3691.38	-3690.56	*m*-CPBA	0.073287	-955.76	-955.687
**Int4**_Conf2	0.817353	-3366.71	-3365.9	*m*-CBA	0.072141	-880.614	-880.542
**Int4**_Conf3	0.822244	-3691.39	-3690.57	BF_2OHBF_3	0.002499	-625.326	-625.324
**Int4**	0.829024	-3691.45	-3690.63	HF	-0.007372	-100.491	-100.498
**Int4**$^+$	0.805829	-3066.52	-3065.71	BF_2OBF_2	-0.007582	-524.821	-524.829
ArI	0.551995	-2217.81	-2217.26				

9. ^{1}H, ^{13}C, and ^{19}F NMR Spectra of Aminofluorination Products and Derivatives

Supplementary Figure 54. ^{1}H NMR spectrum of 1b

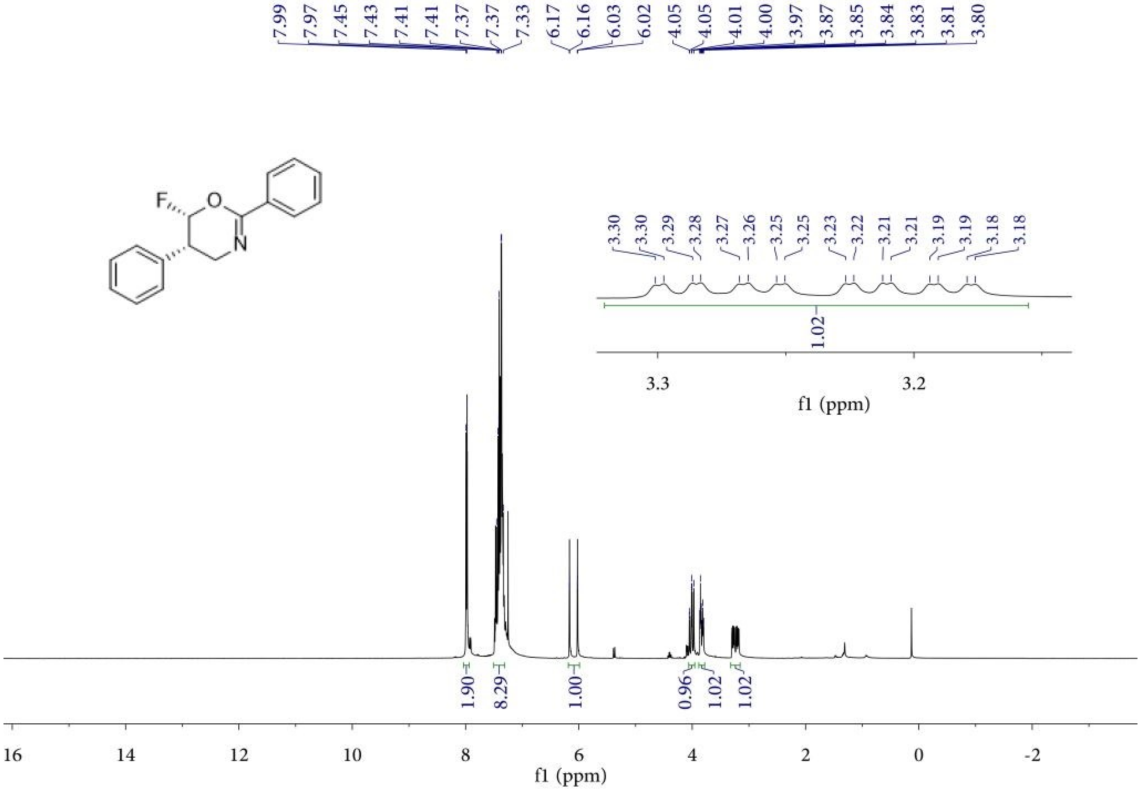

Supplementary Figure 55. ^{13}C NMR spectrum of 1b

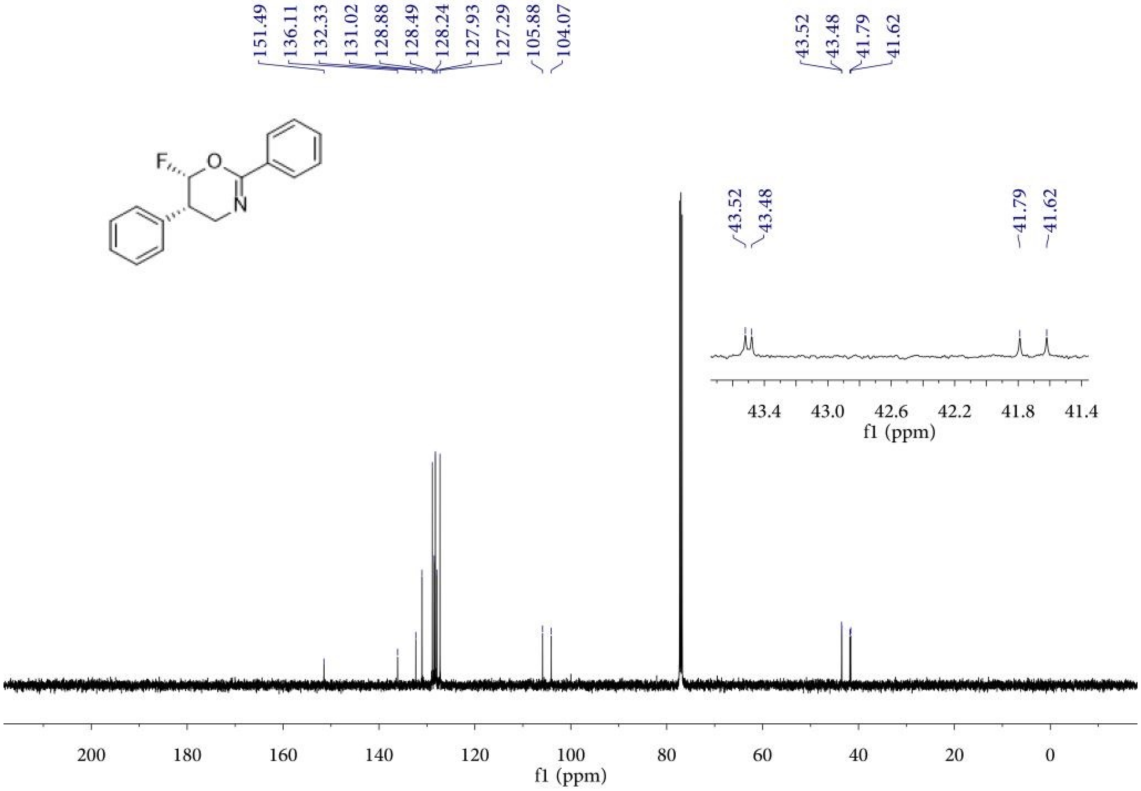

Supplementary Figure 56. ^{19}F NMR spectrum of 1b

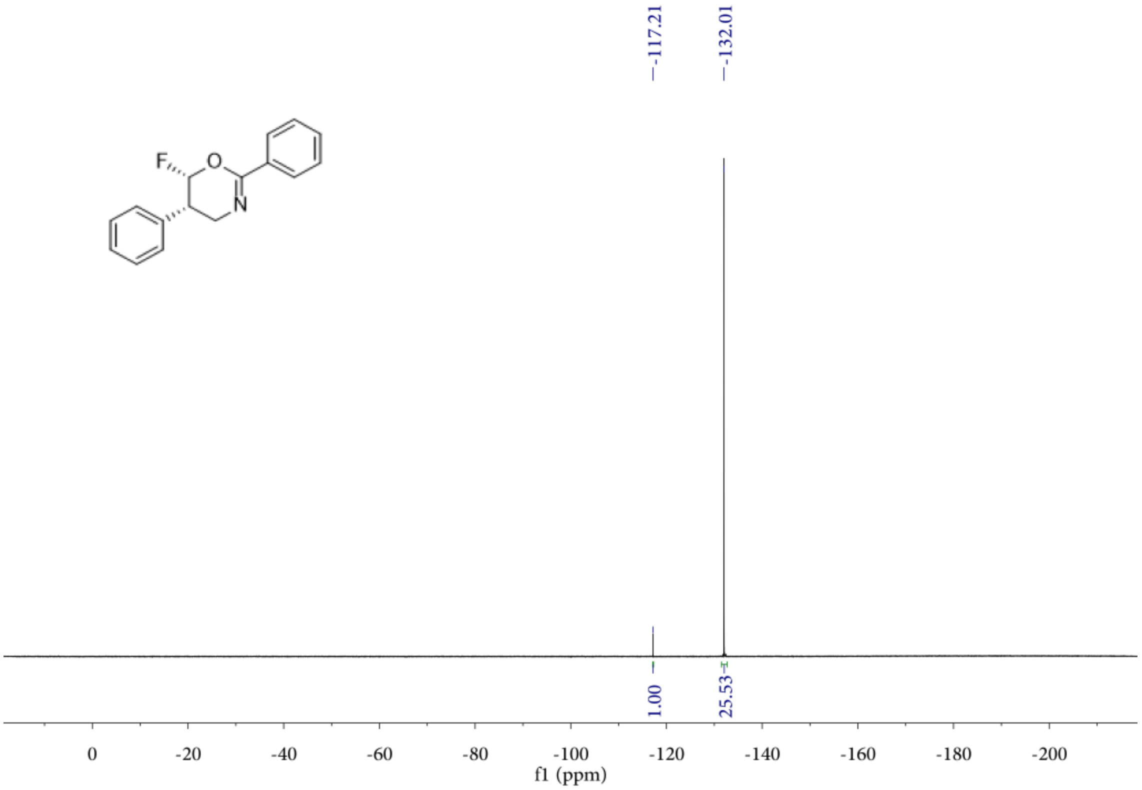

Supplementary Figure 57. ^{1}H NMR spectrum of 2b

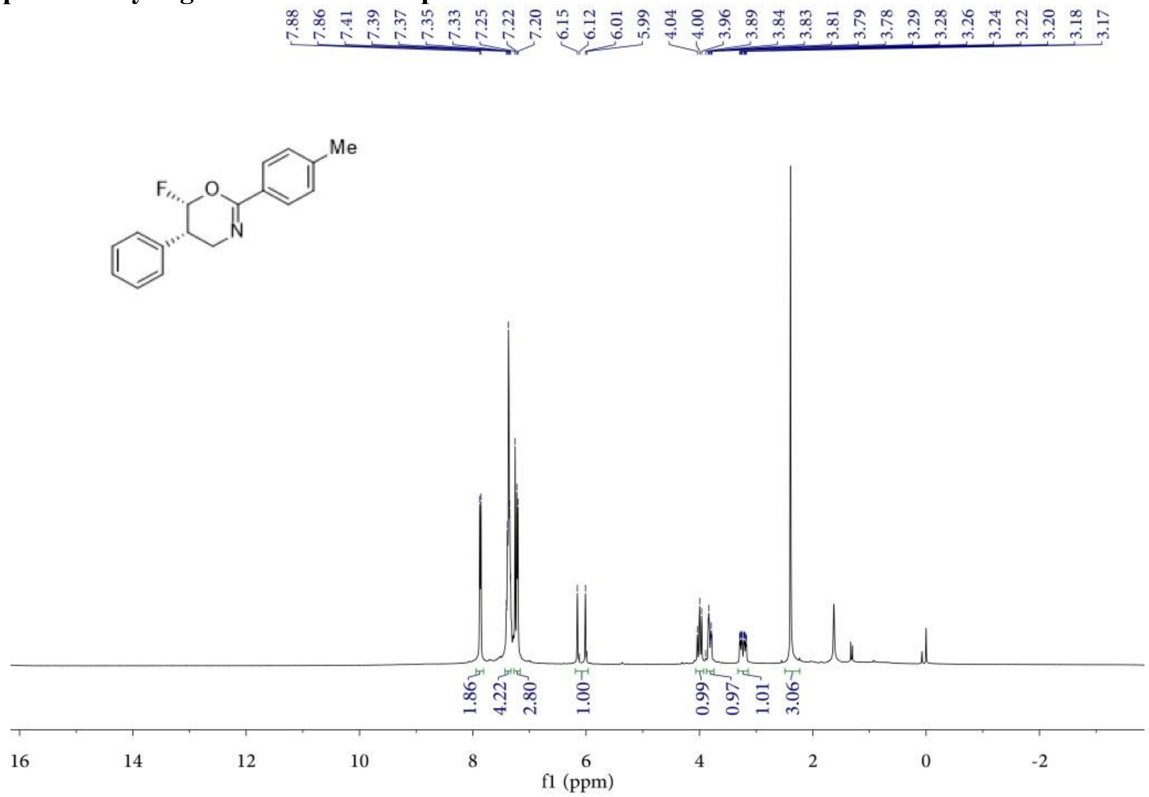

Supplementary Figure 58. ^{13}C NMR spectrum of 2b

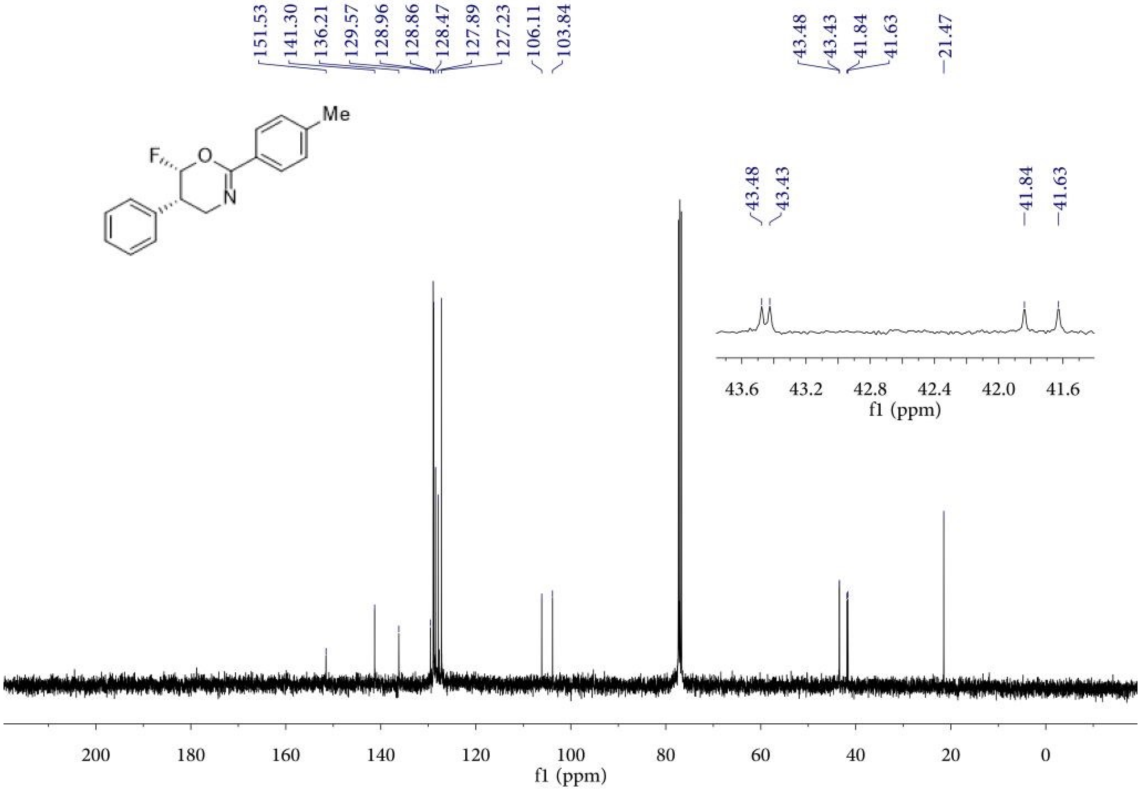

Supplementary Figure 59. ^{19}F NMR spectrum of 2b

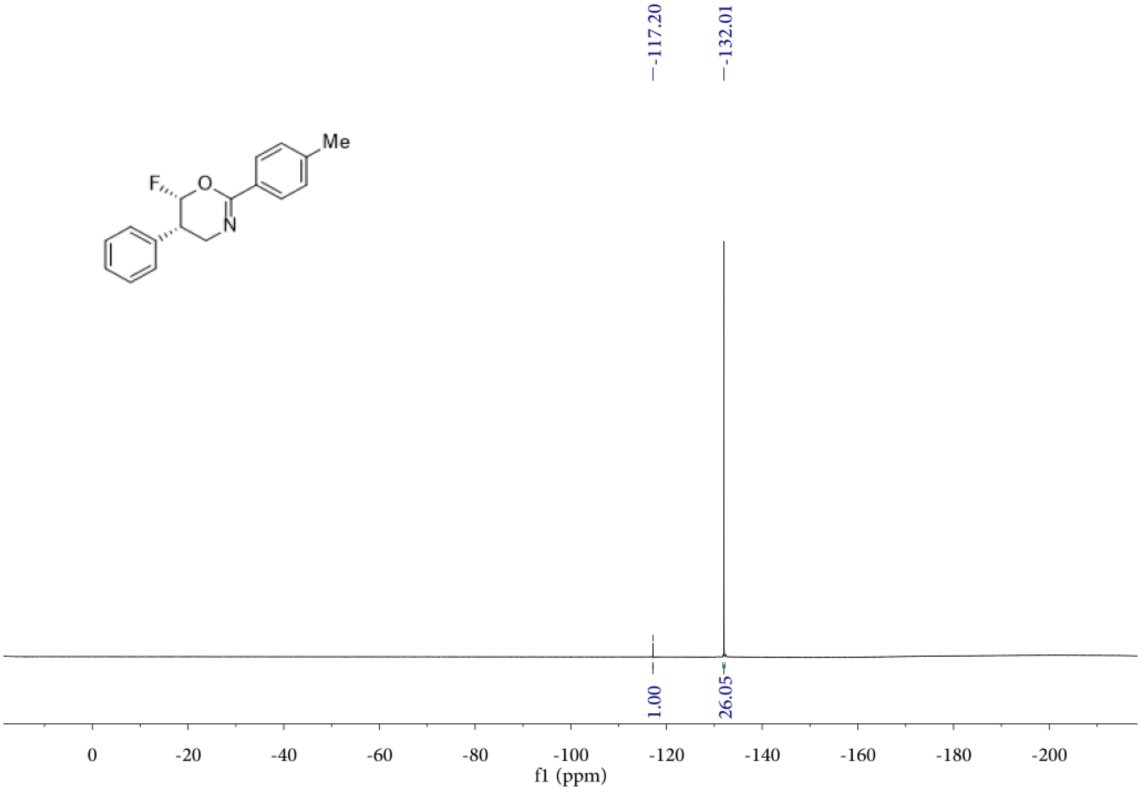

Supplementary Figure 60. ^{1}H NMR spectrum of 3b

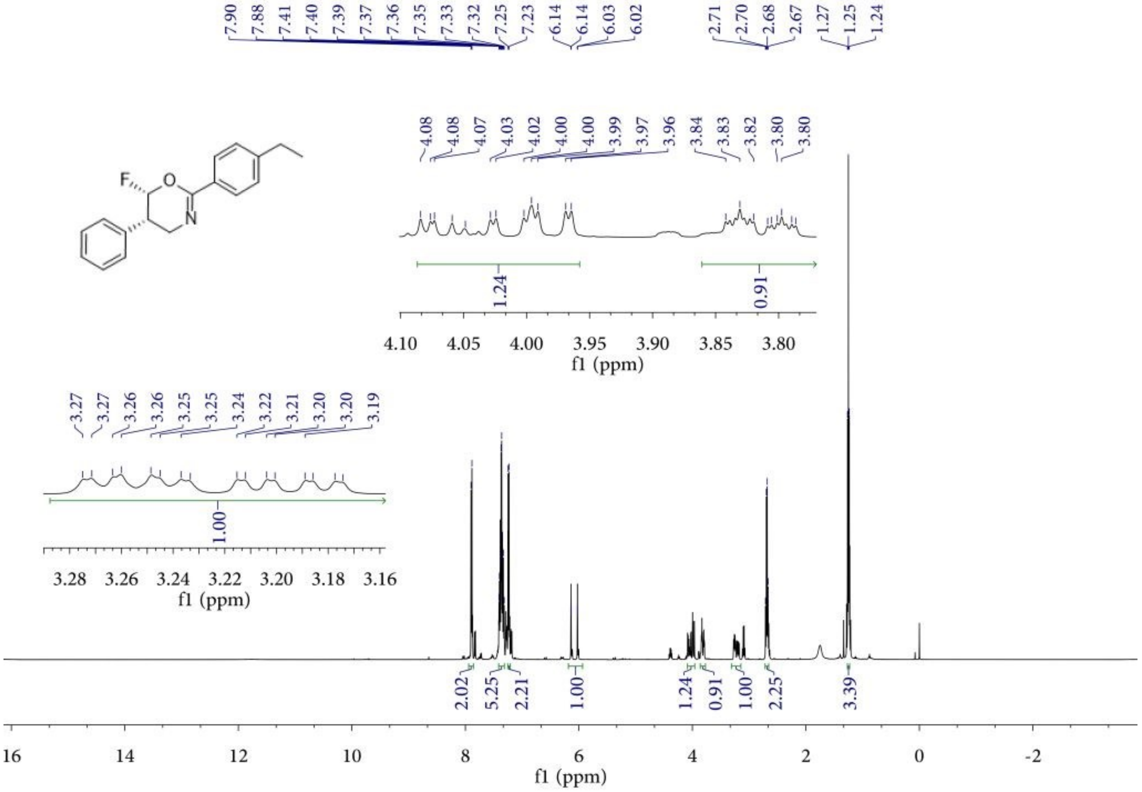

Supplementary Figure 61. ^{13}C NMR spectrum of 3b

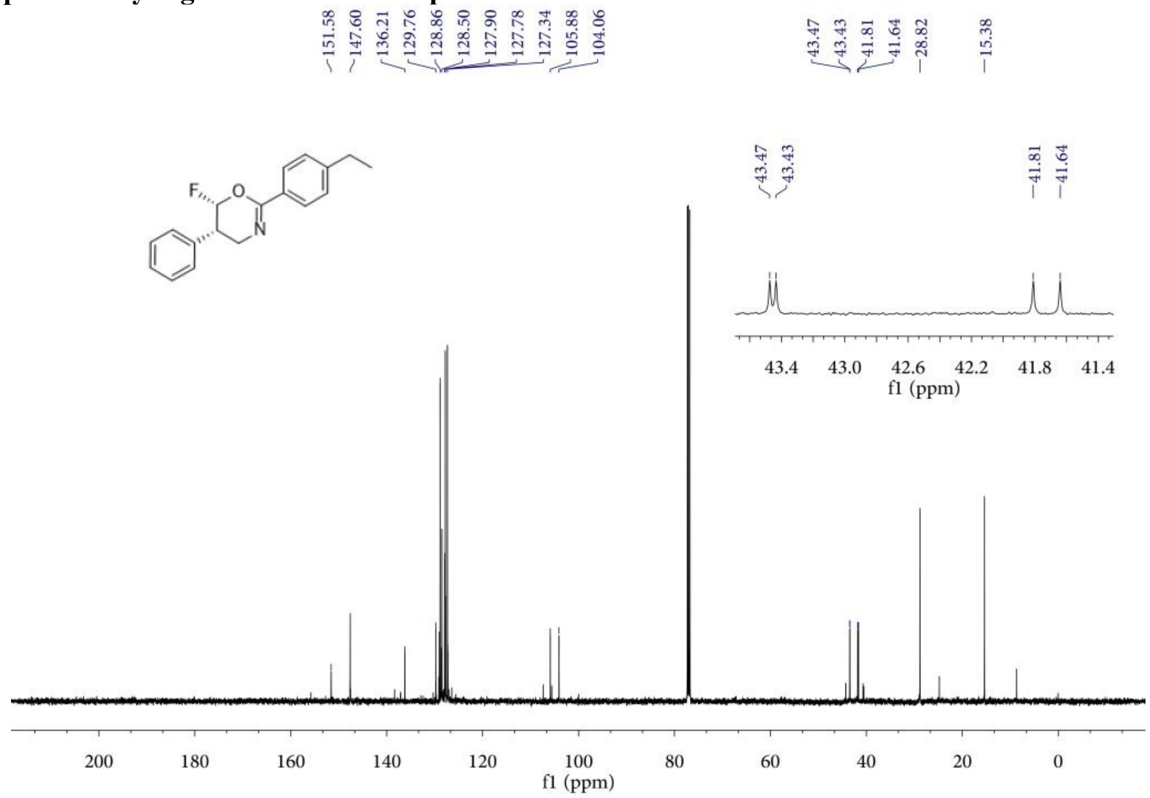

Supplementary Figure 62. ^{19}F NMR spectrum of 3b

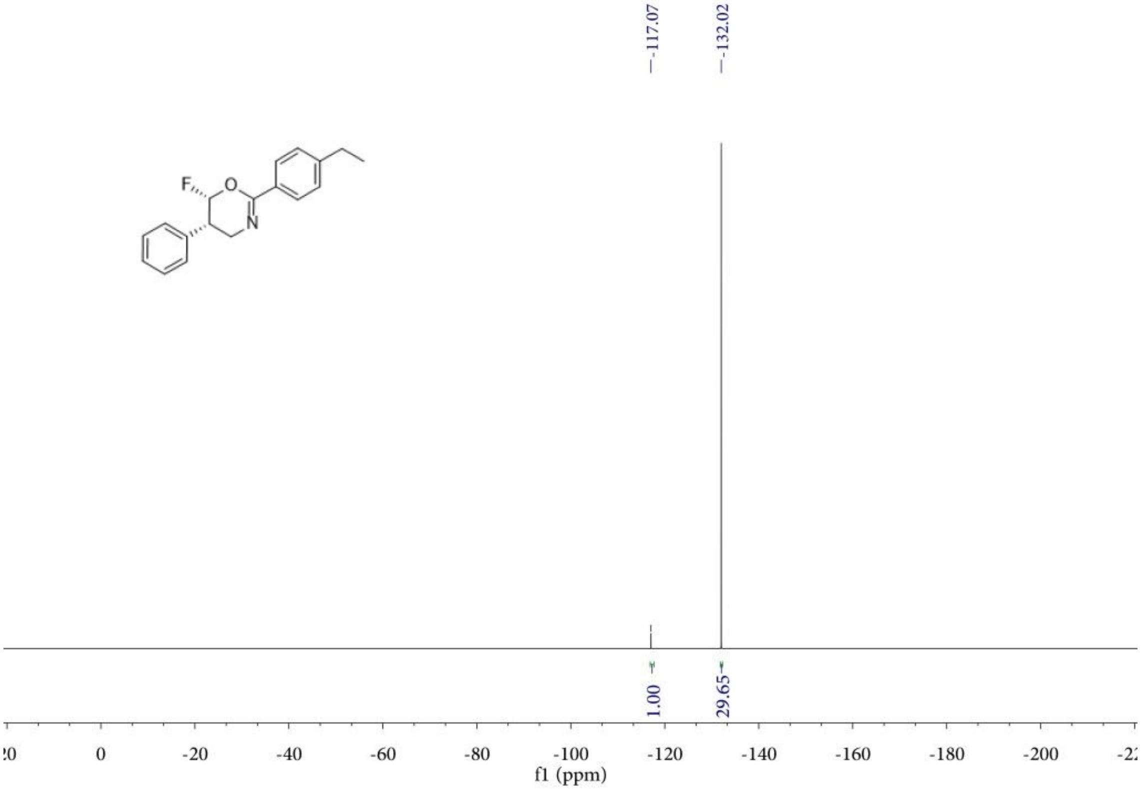

Supplementary Figure 63. ^{1}H NMR spectrum of 4b

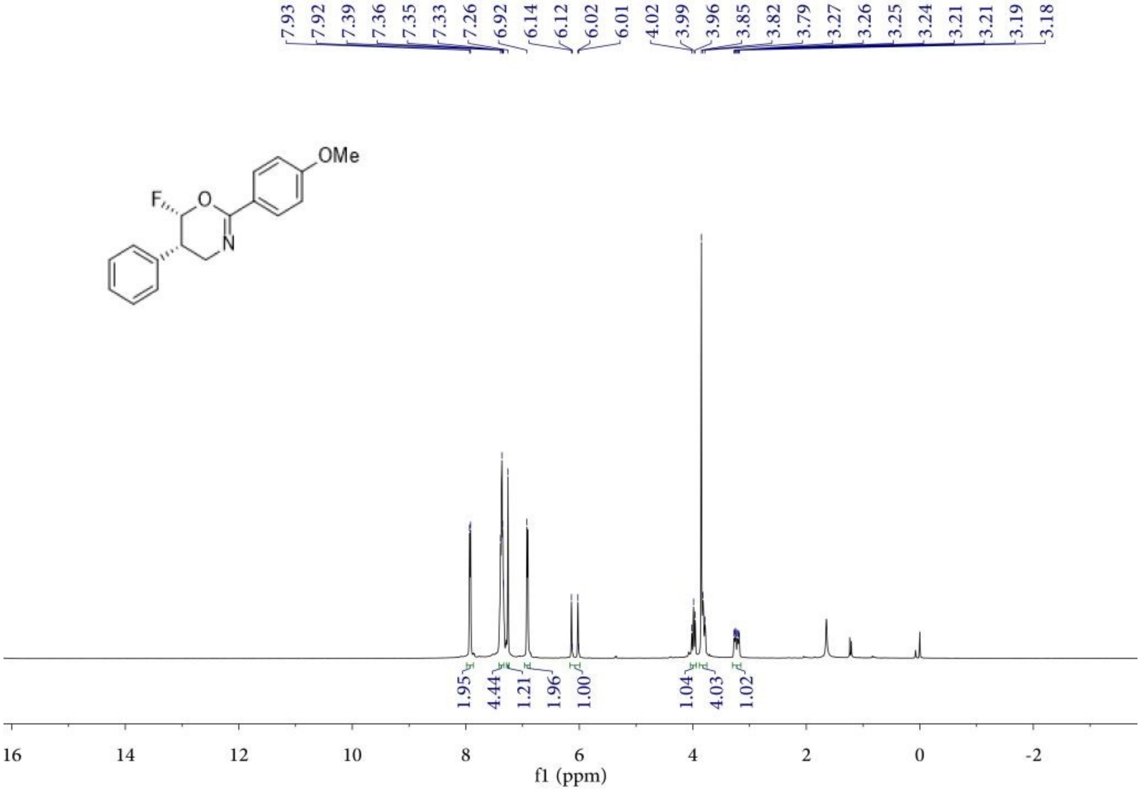

Supplementary Figure 64. ^{13}C NMR spectrum of 4b

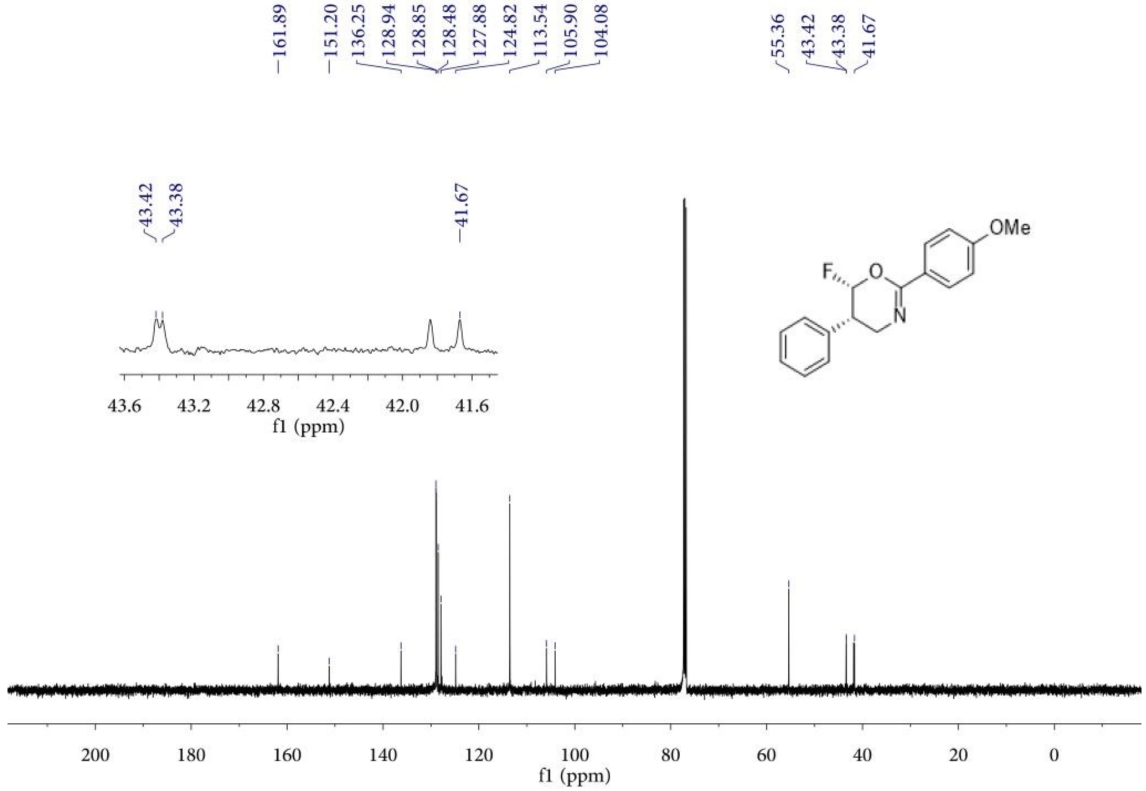

Supplementary Figure 65. ^{19}F NMR spectrum of 4b

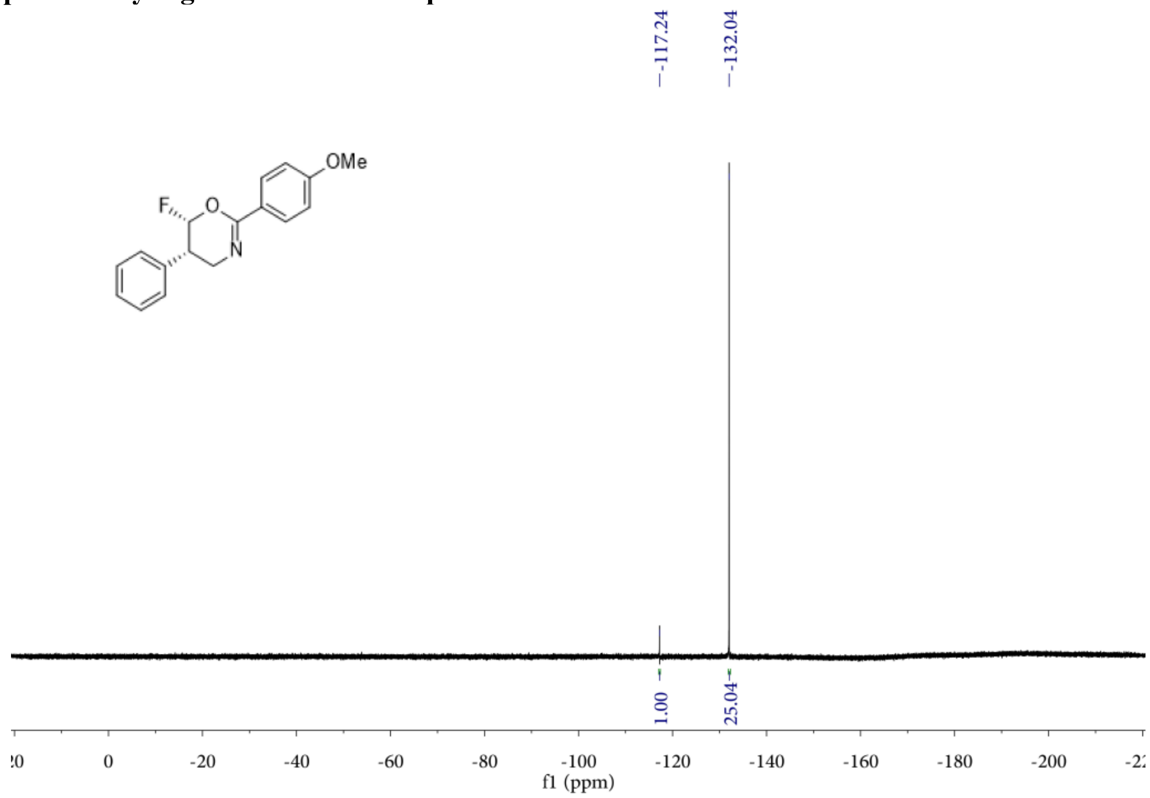

Supplementary Figure 66. ^{1}H NMR spectrum of 5b

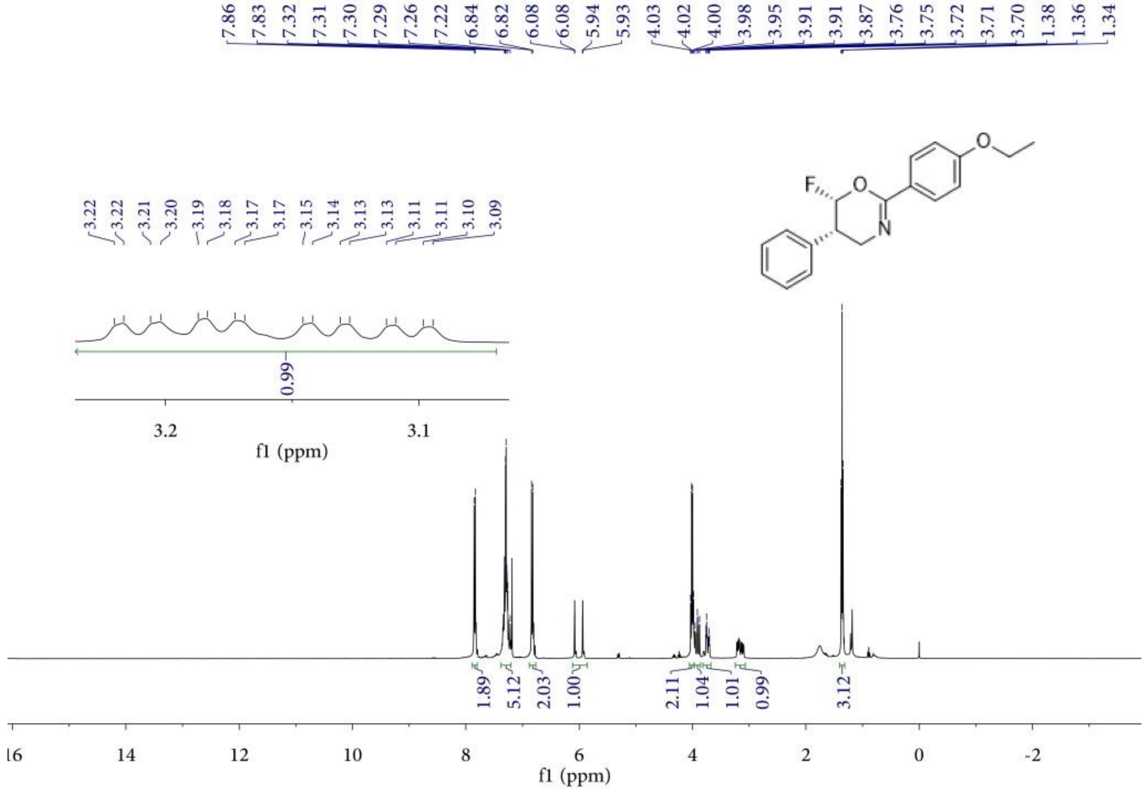

Supplementary Figure 67. ^{13}C NMR spectrum of 5b

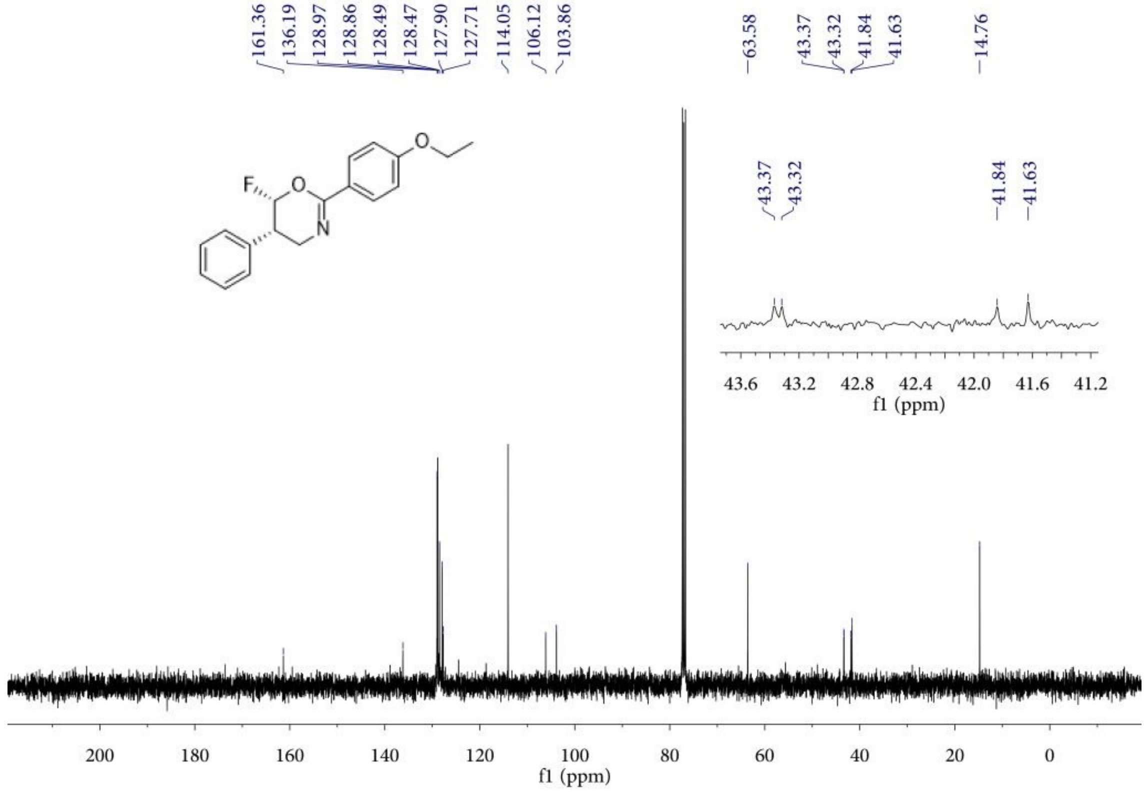

Supplementary Figure 68. ^{19}F NMR spectrum of 5b

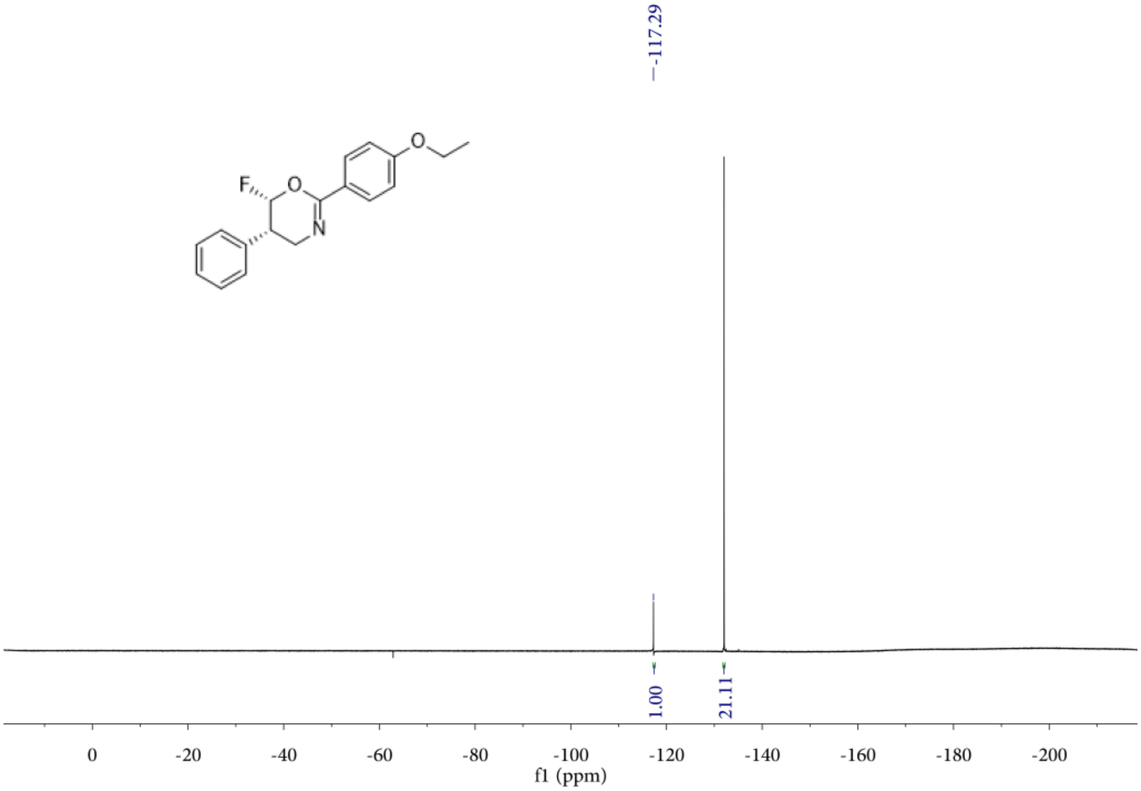

Supplementary Figure 69. ^{1}H NMR spectrum of 6b

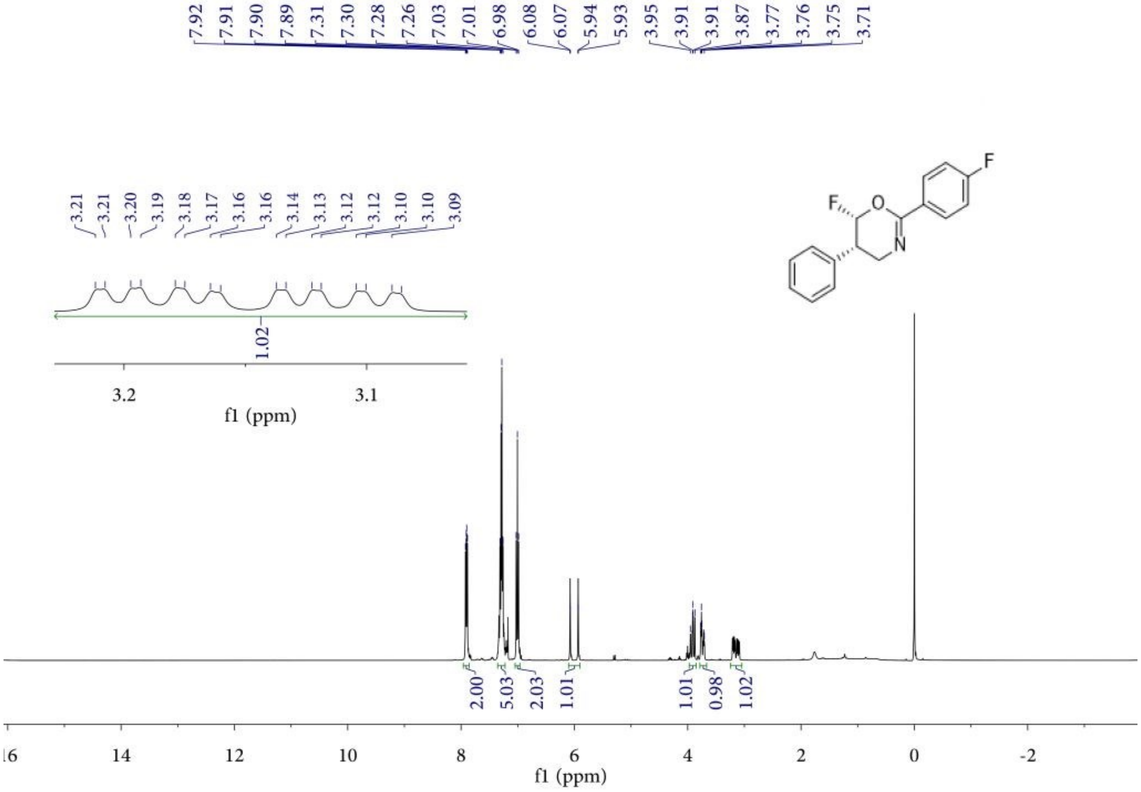

Supplementary Figure 70. ^{13}C NMR spectrum of 6b

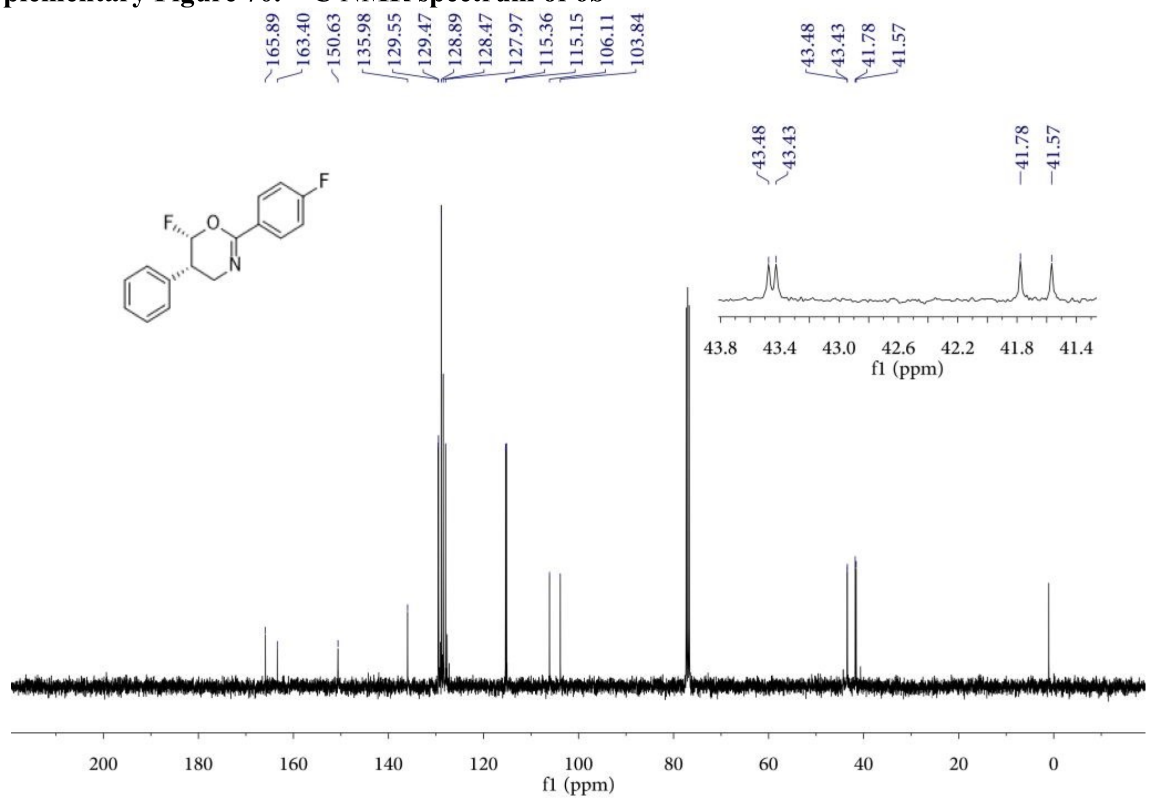

Supplementary Figure 71. ^{19}F NMR spectrum of 6b

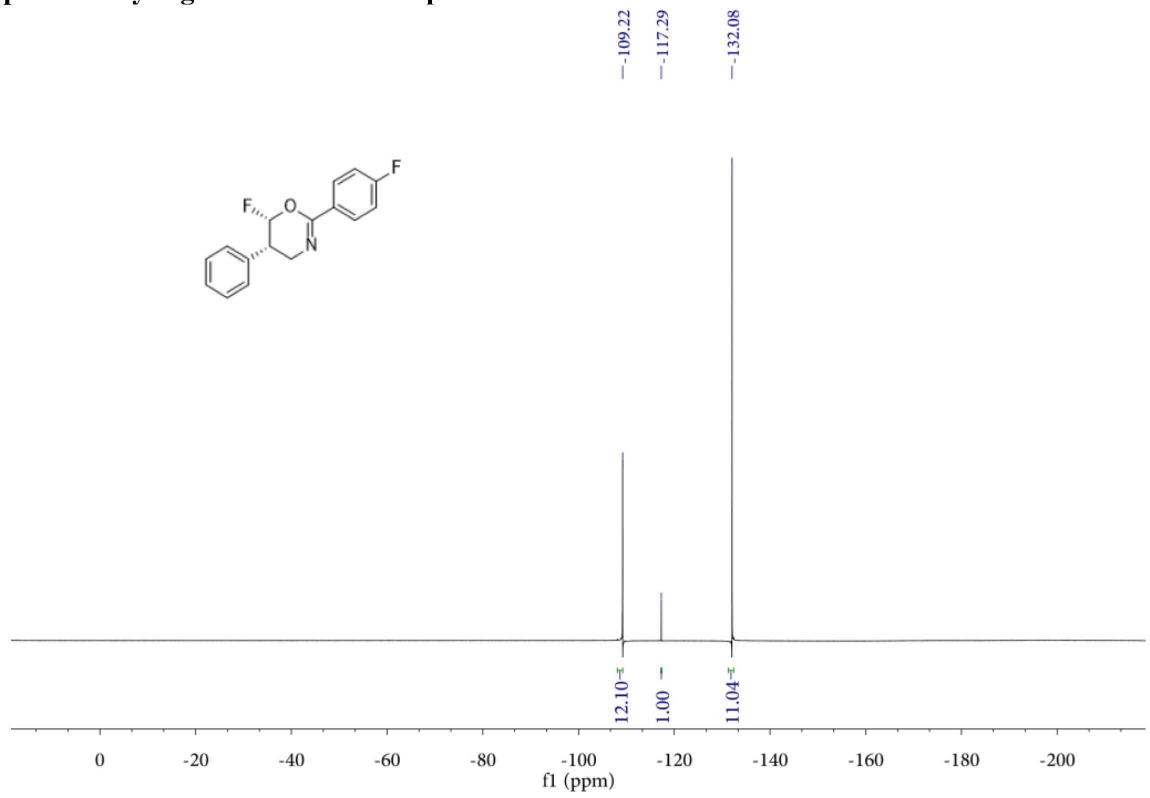

Supplementary Figure 72. ^{1}H NMR spectrum of 7b

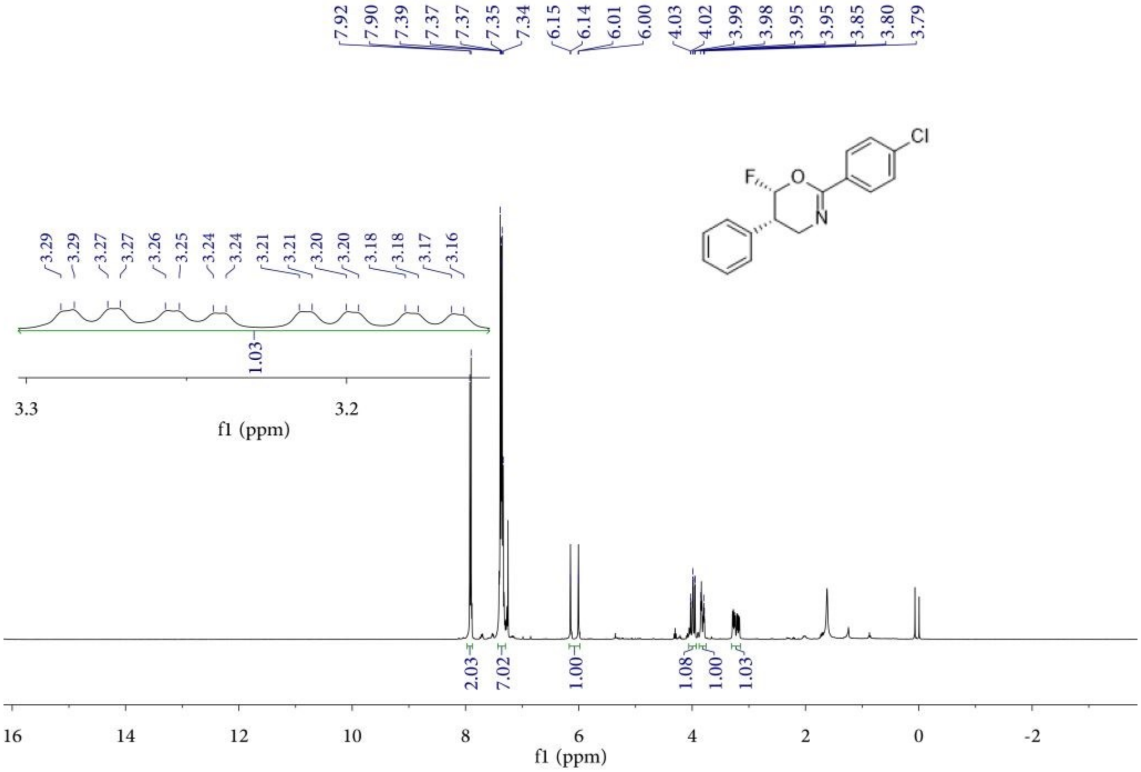

Supplementary Figure 73. ^{13}C NMR spectrum of 7b

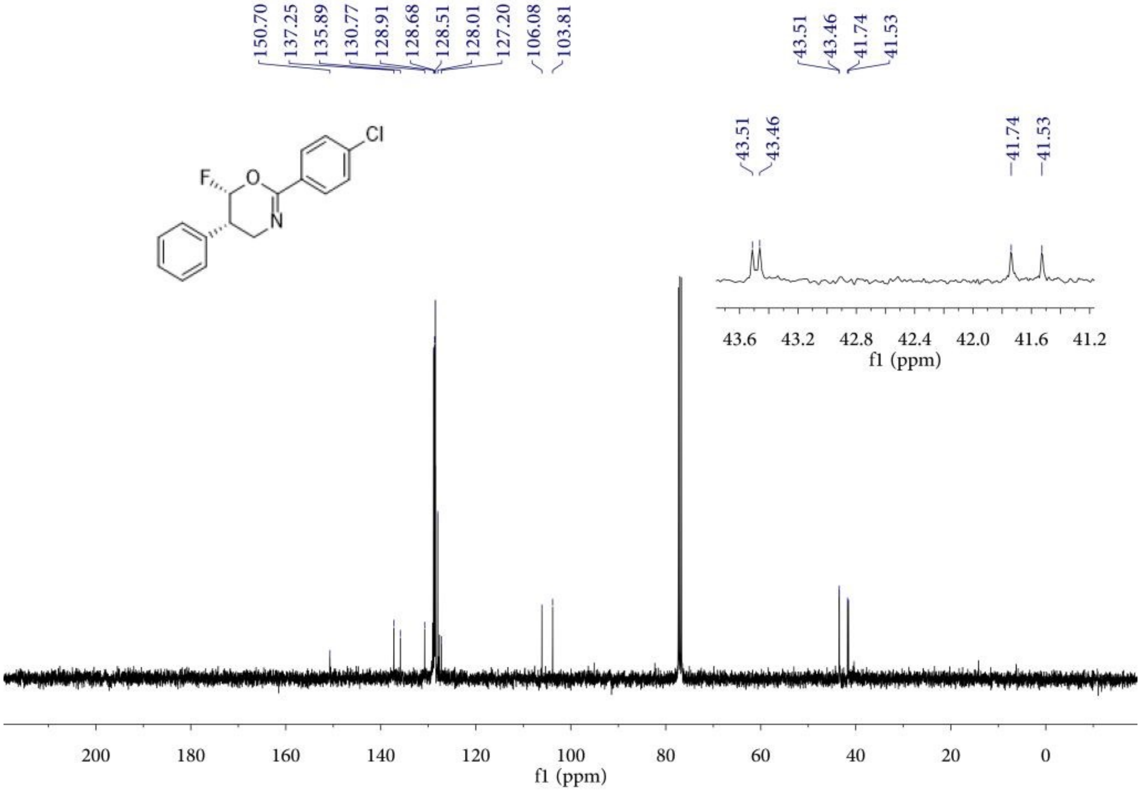

Supplementary Figure 74. ^{19}F NMR spectrum of 7b

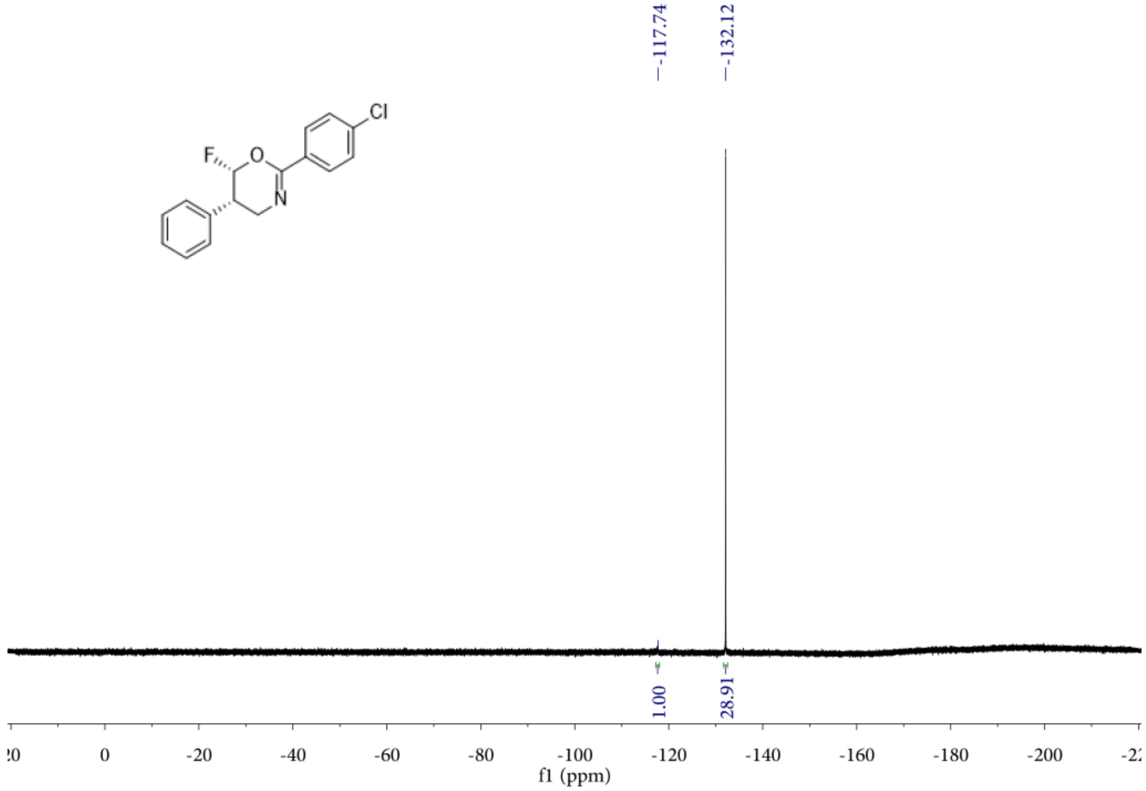

Supplementary Figure 75. ^{1}H NMR spectrum of 8b

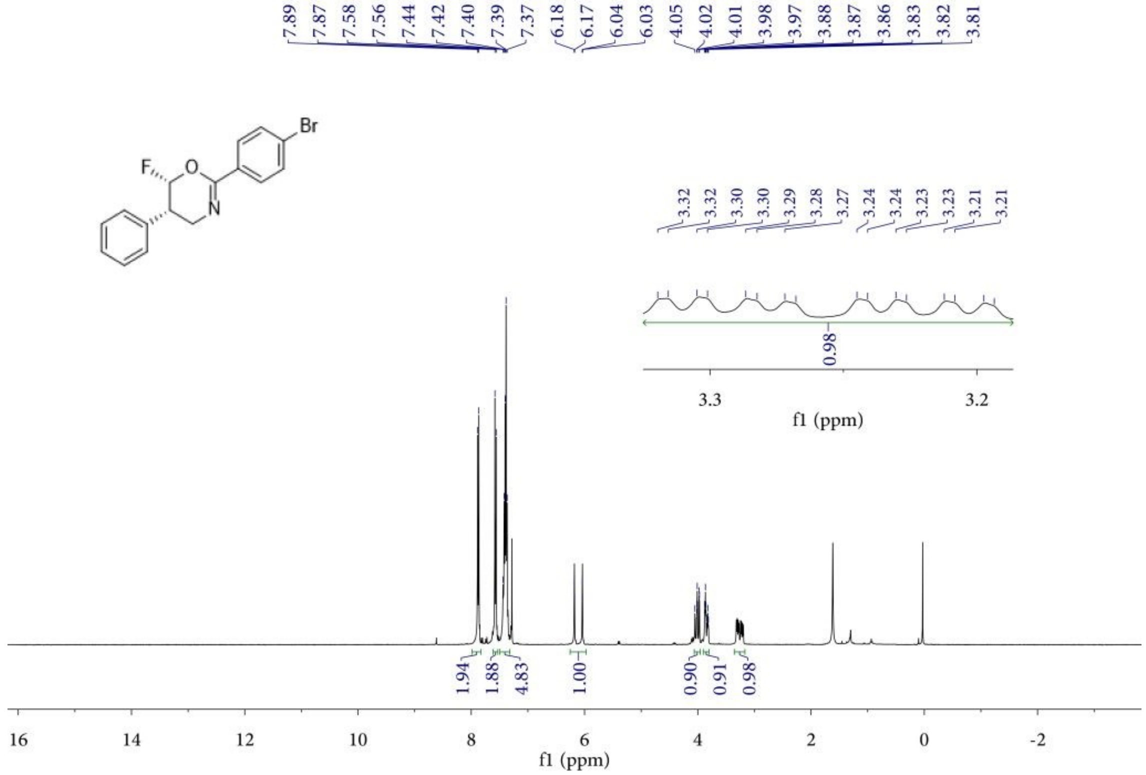

Supplementary Figure 76. ^{13}C NMR spectrum of 8b

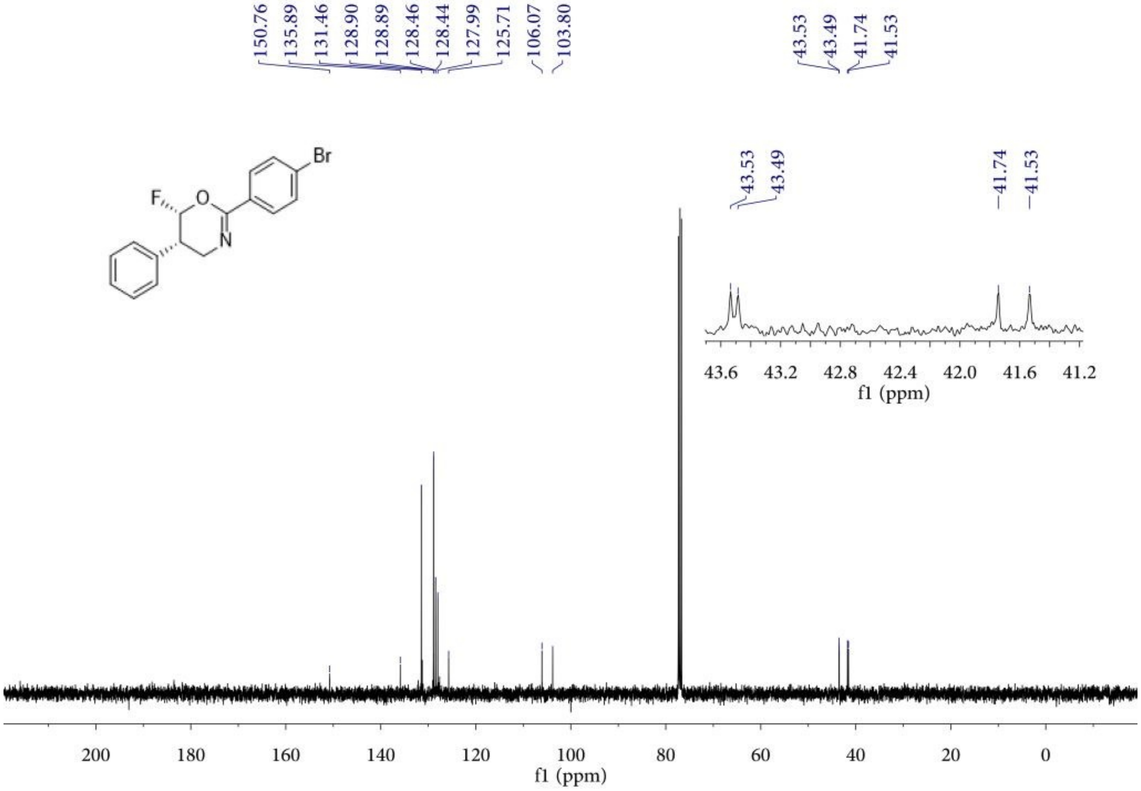

Supplementary Figure 77. ^{19}F NMR spectrum of 8b

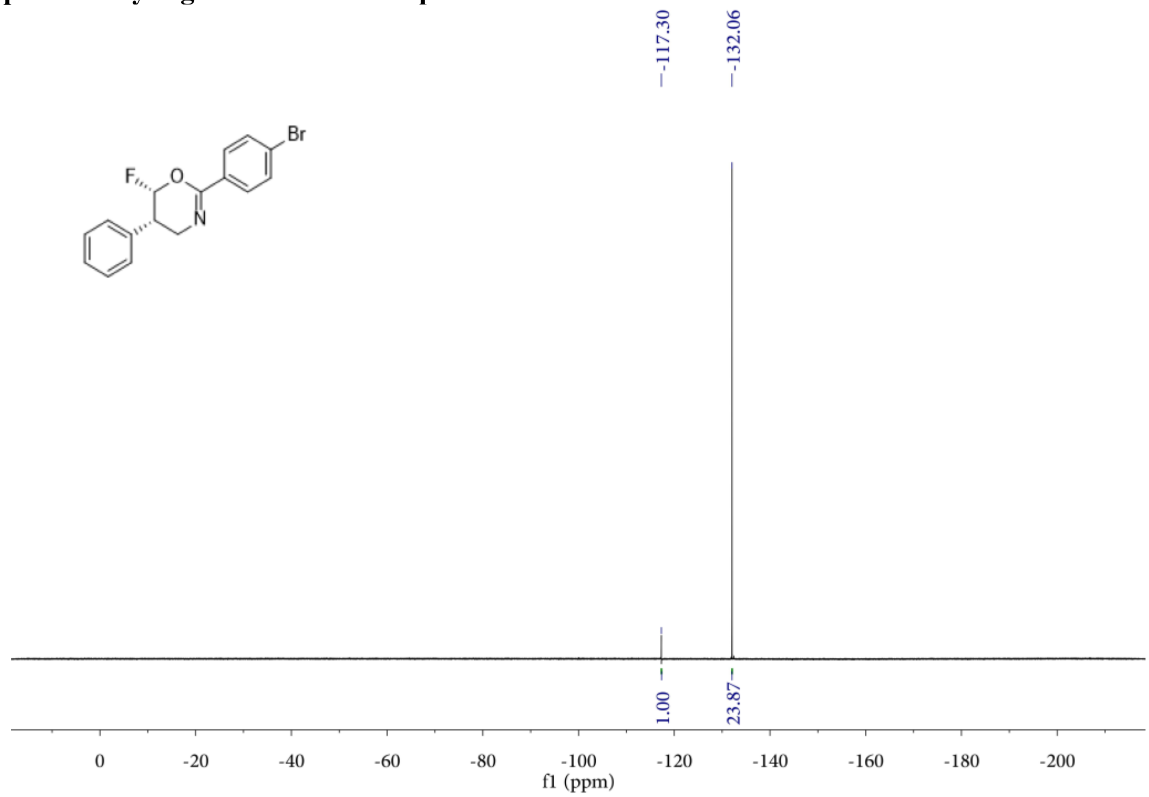

Supplementary Figure 78. ^{1}H NMR spectrum of 9b

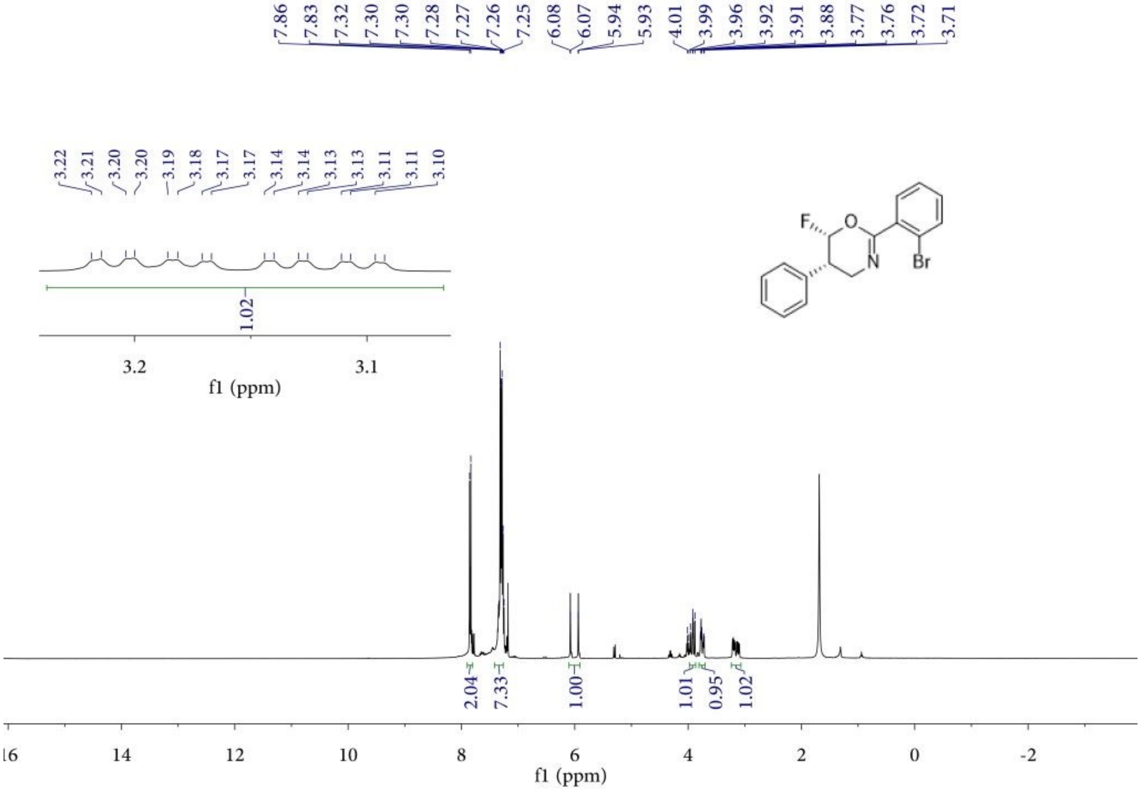

Supplementary Figure 79. ^{13}C NMR spectrum of 9b

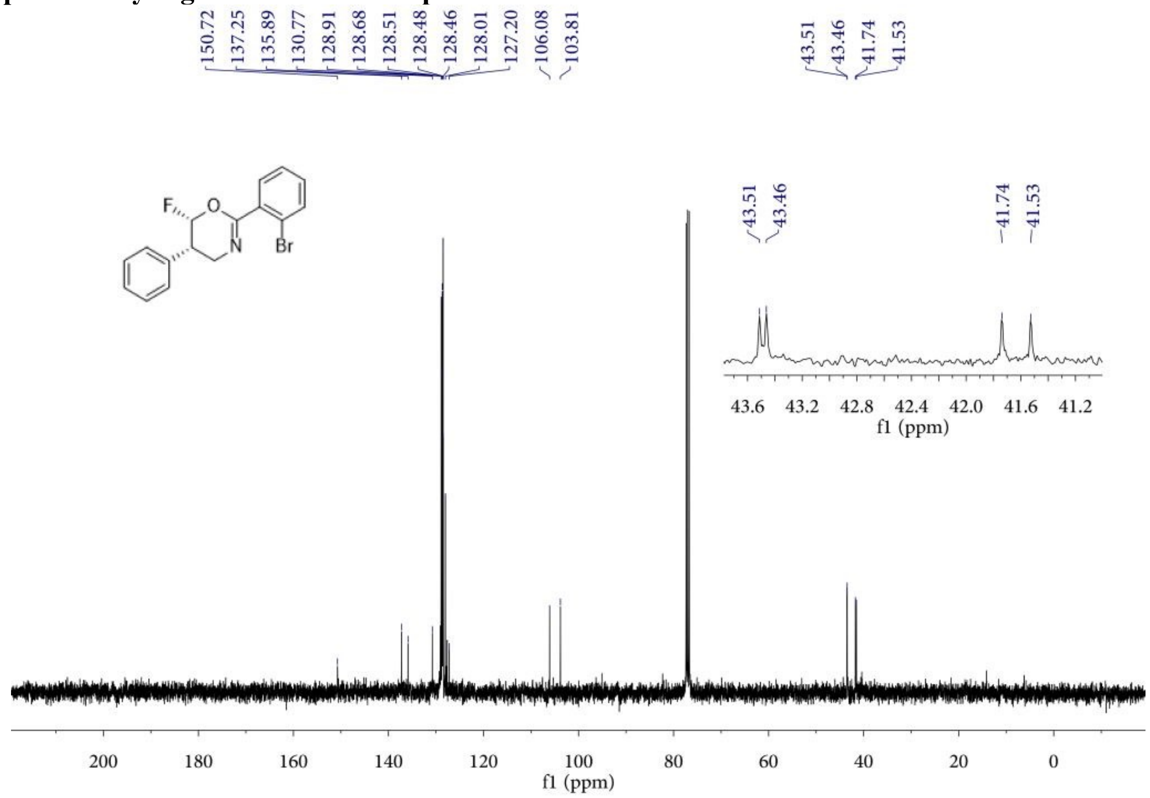

Supplementary Figure 80. ^{19}F NMR spectrum of 9b

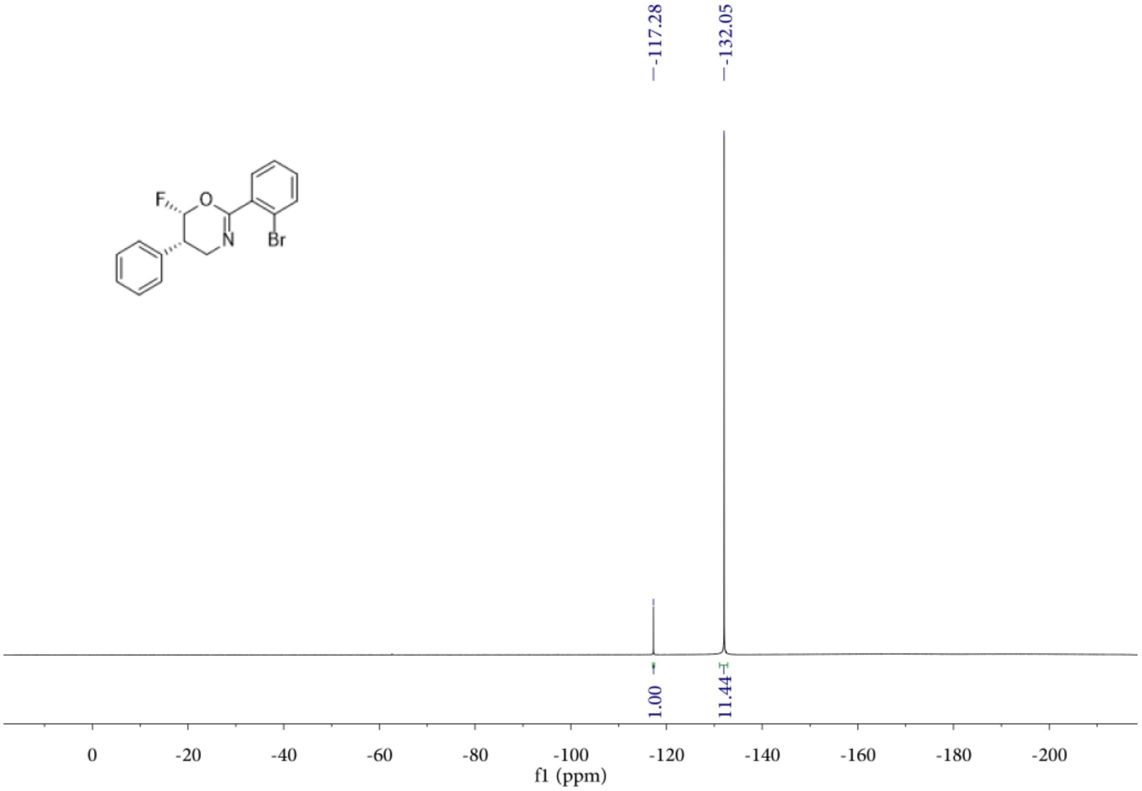

Supplementary Figure 81. ^{1}H NMR spectrum of 10b

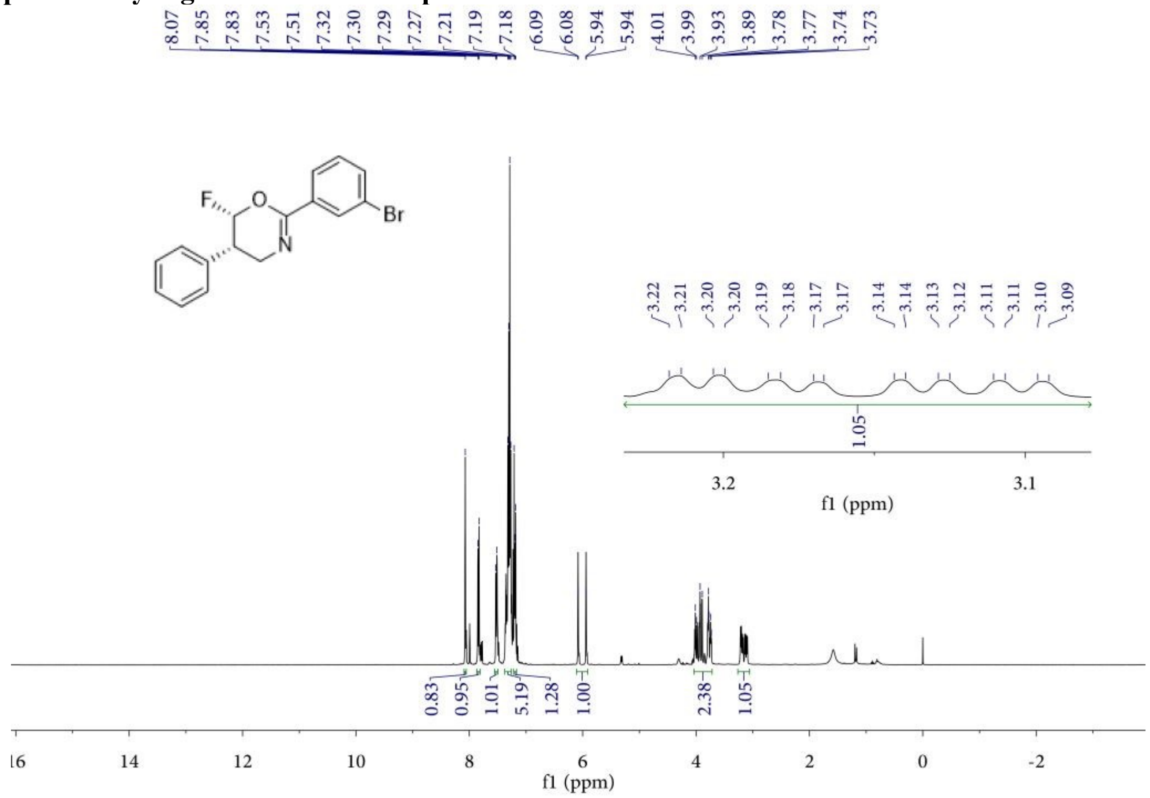

Supplementary Figure 82. ^{13}C NMR spectrum of 10b

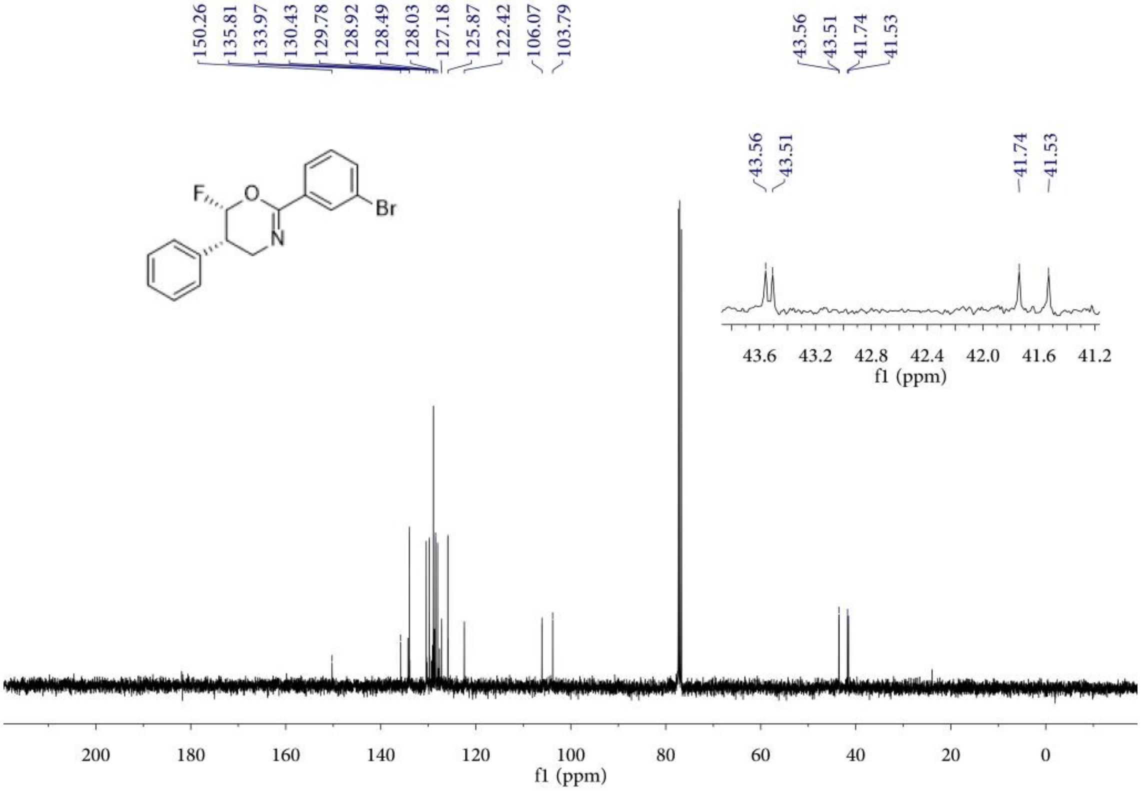

Supplementary Figure 83. ^{19}F NMR spectrum of 10b

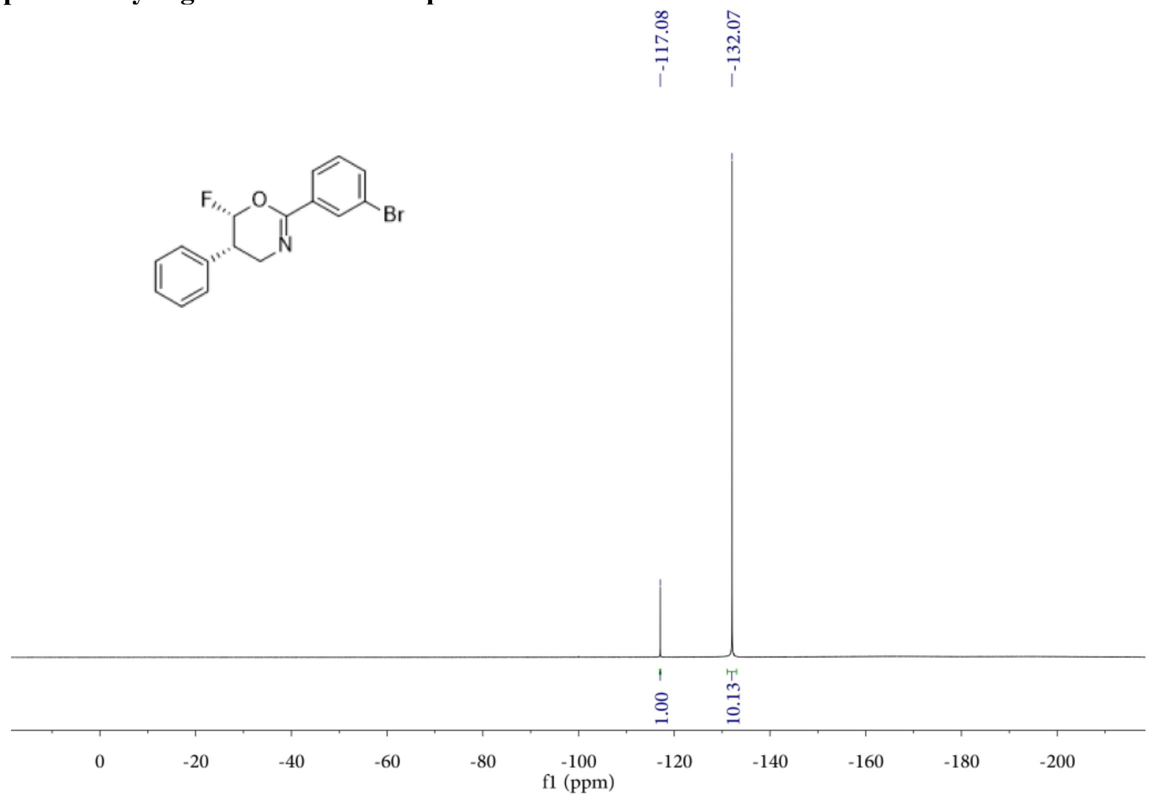

Supplementary Figure 84. ^{1}H NMR spectrum of 11b

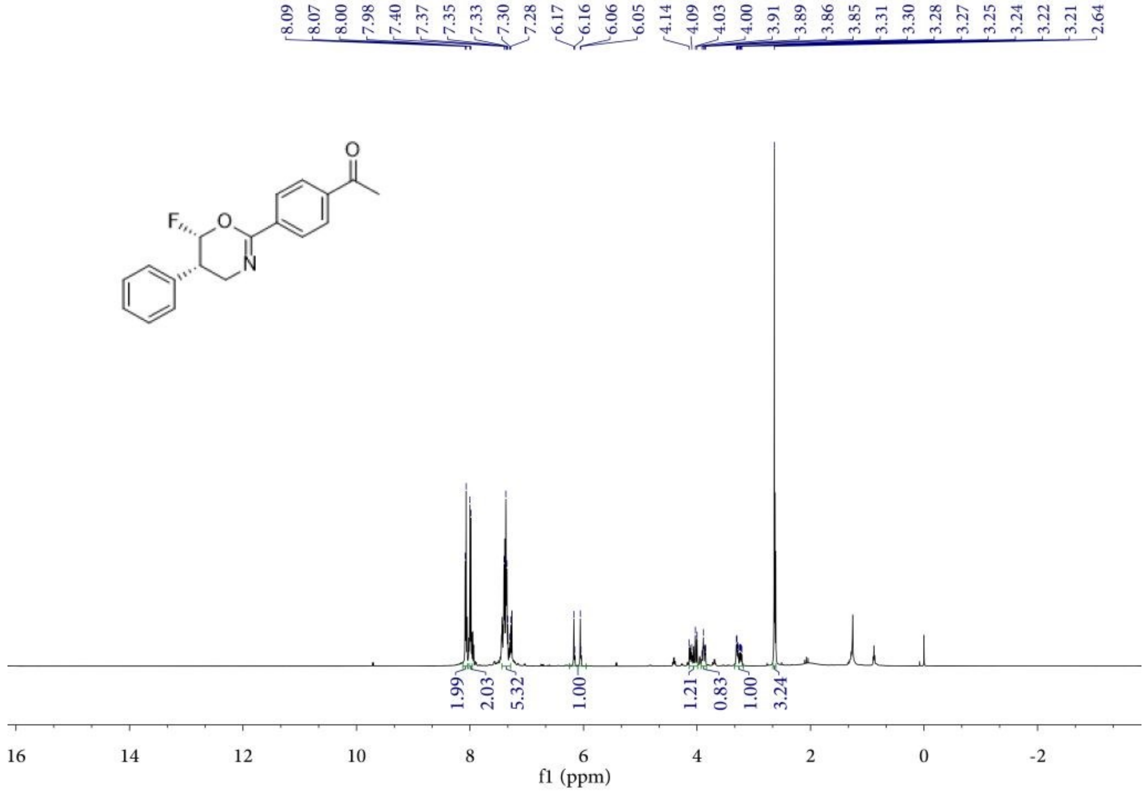

Supplementary Figure 85. ^{13}C NMR spectrum of 11b

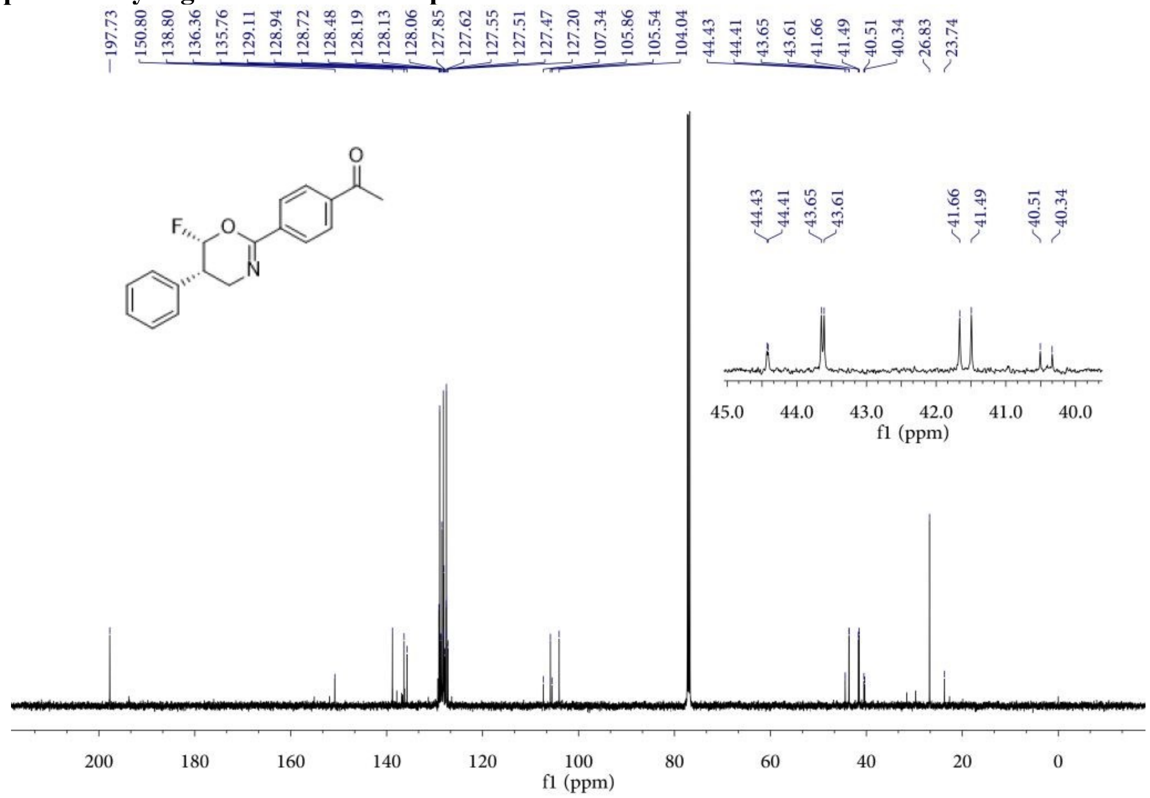

Supplementary Figure 86. ^{19}F NMR spectrum of 11b

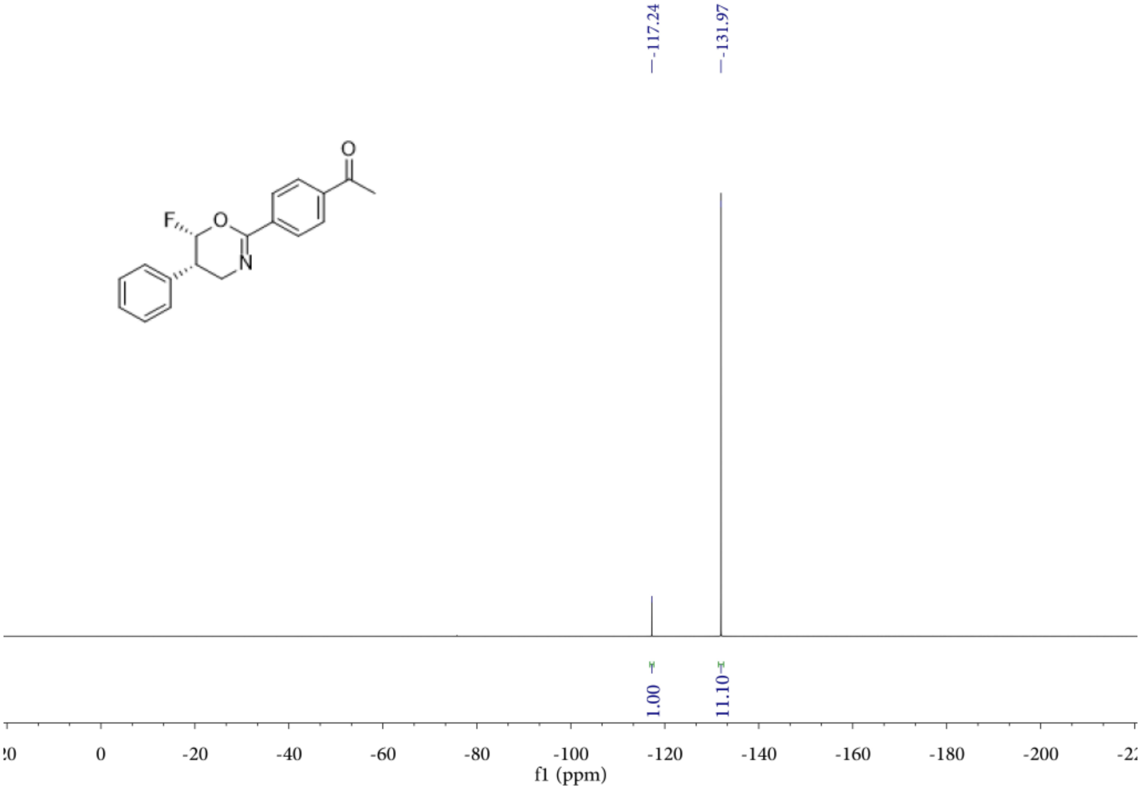

Supplementary Figure 87. ^{1}H NMR spectrum of 12b

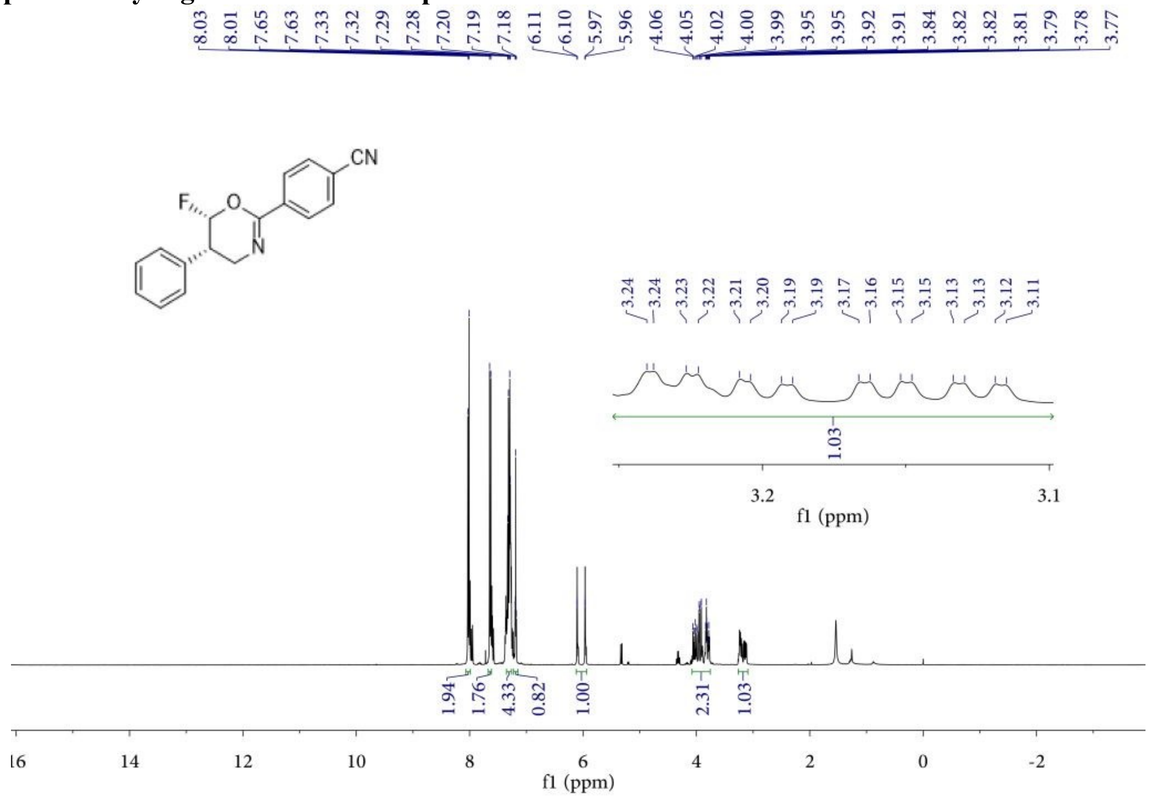

Supplementary Figure 88. ^{13}C NMR spectrum of 12b

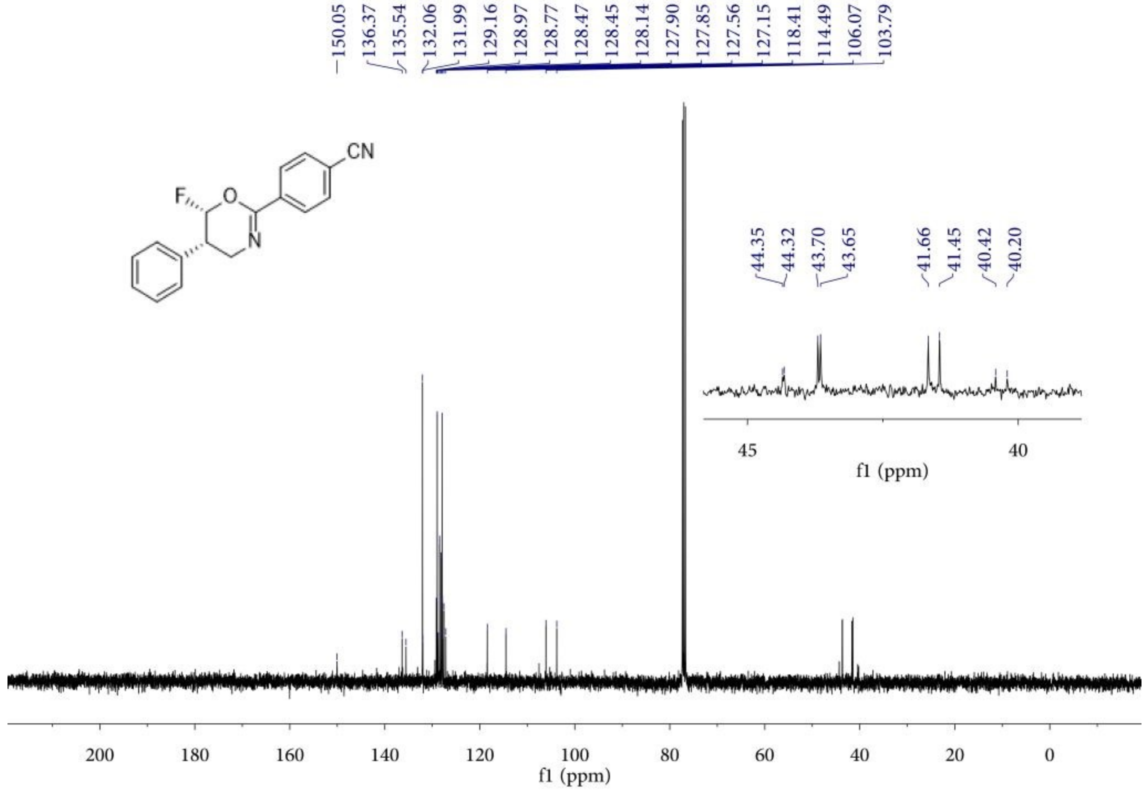

Supplementary Figure 89. ^{19}F NMR spectrum of 12b

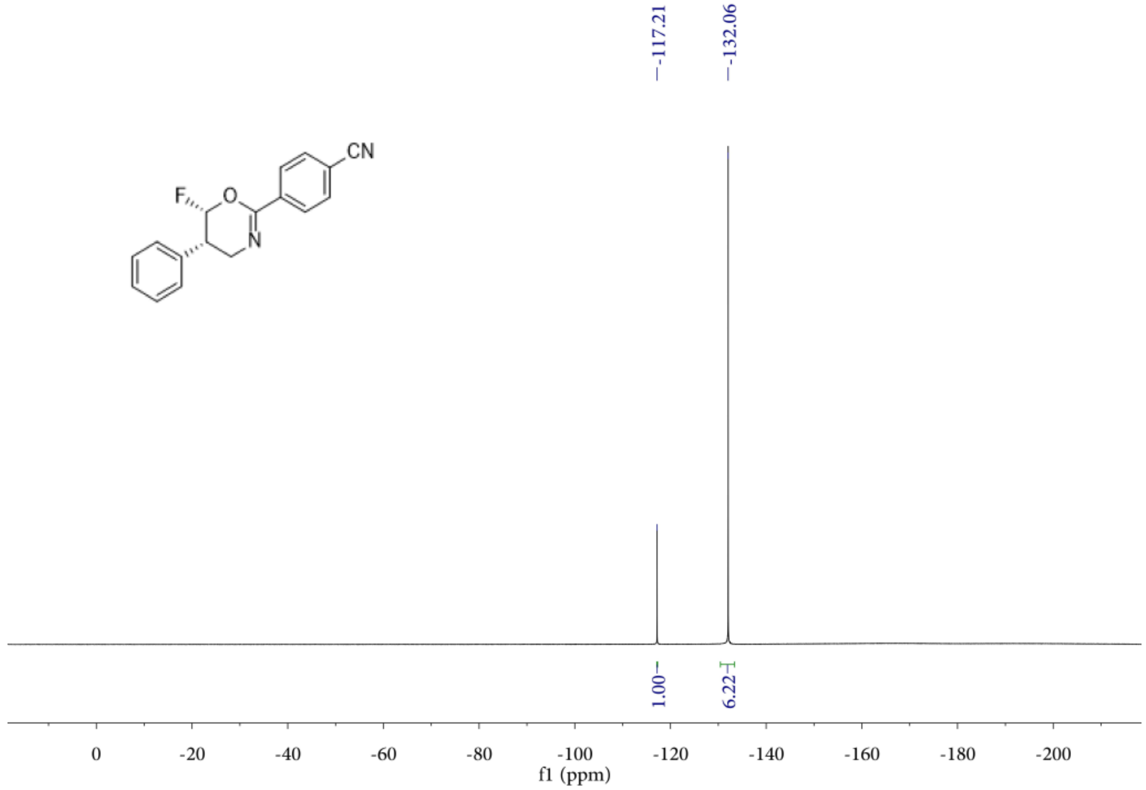

Supplementary Figure 90. ^{1}H NMR spectrum of 13b

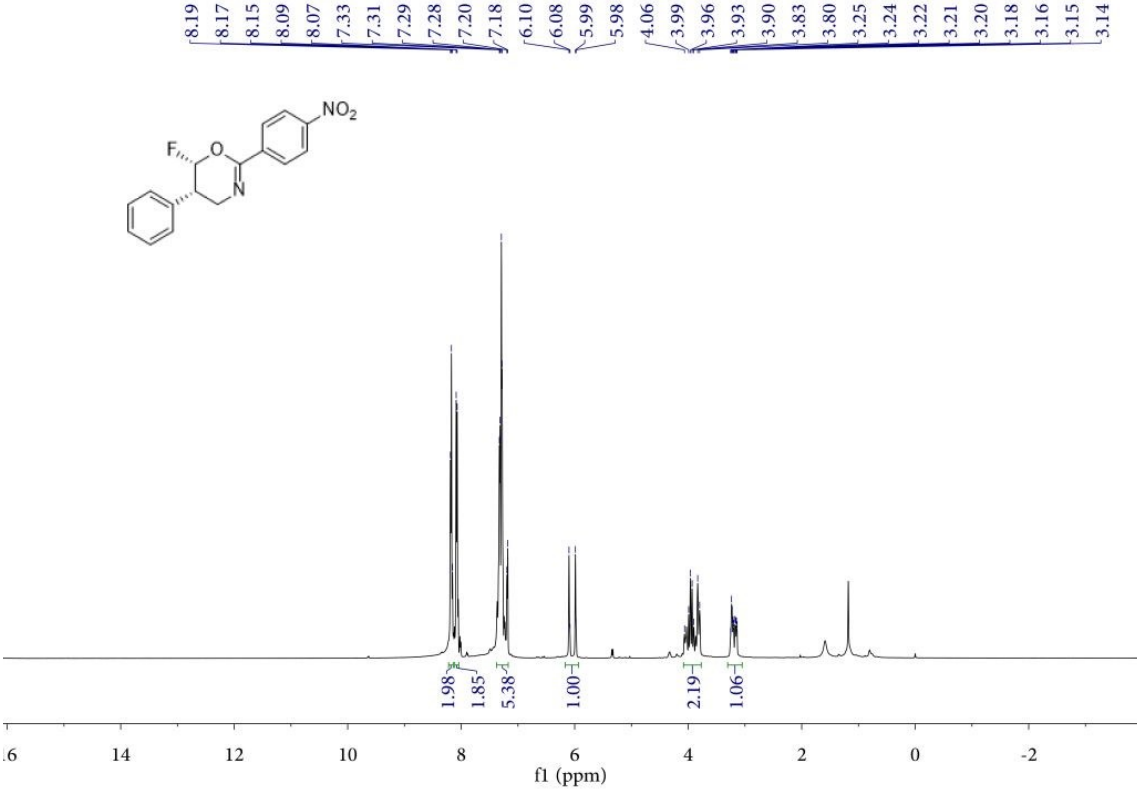

Supplementary Figure 91. ^{13}C NMR spectrum of 13b

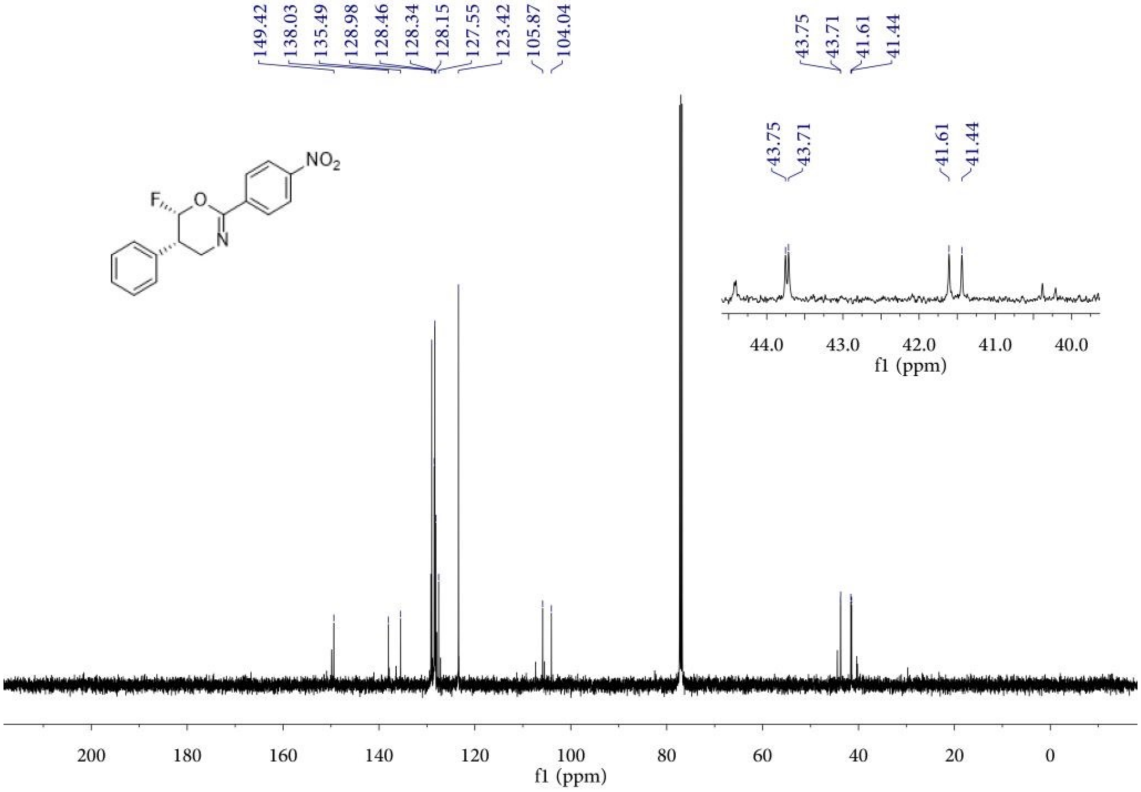

Supplementary Figure 92. ^{19}F NMR spectrum of 13b

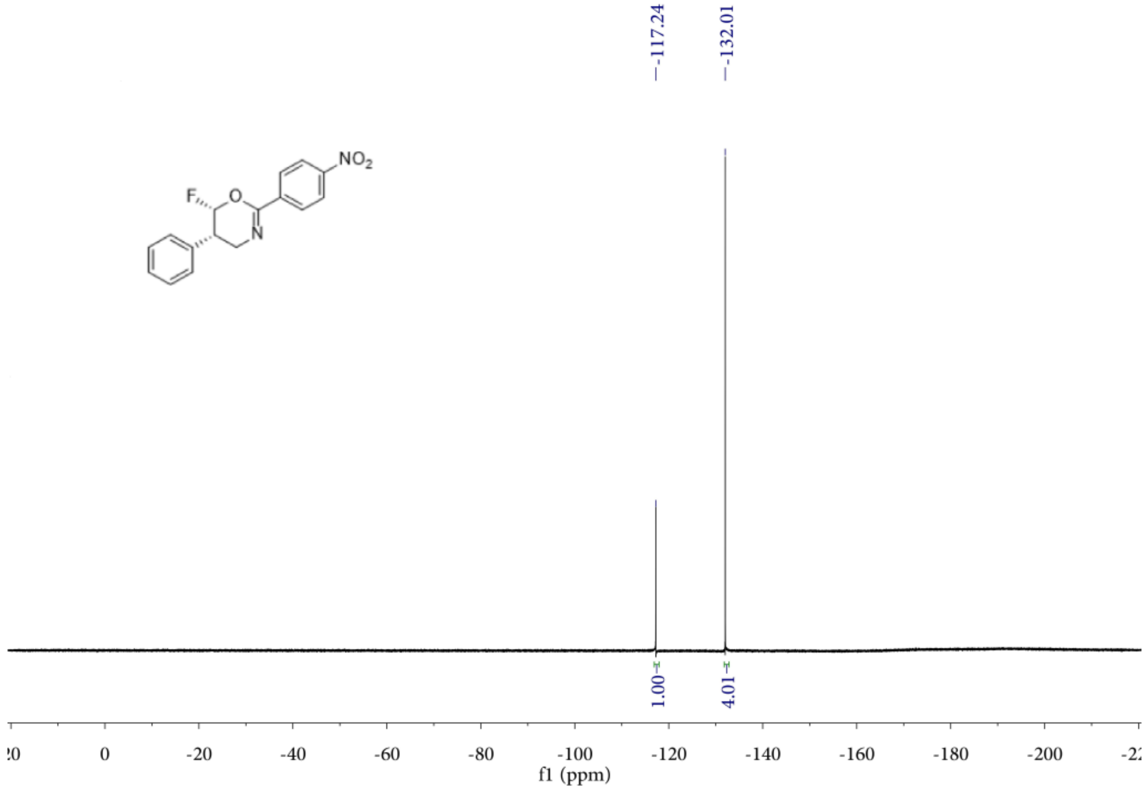

Supplementary Figure 93. ^{1}H NMR spectrum of 14b

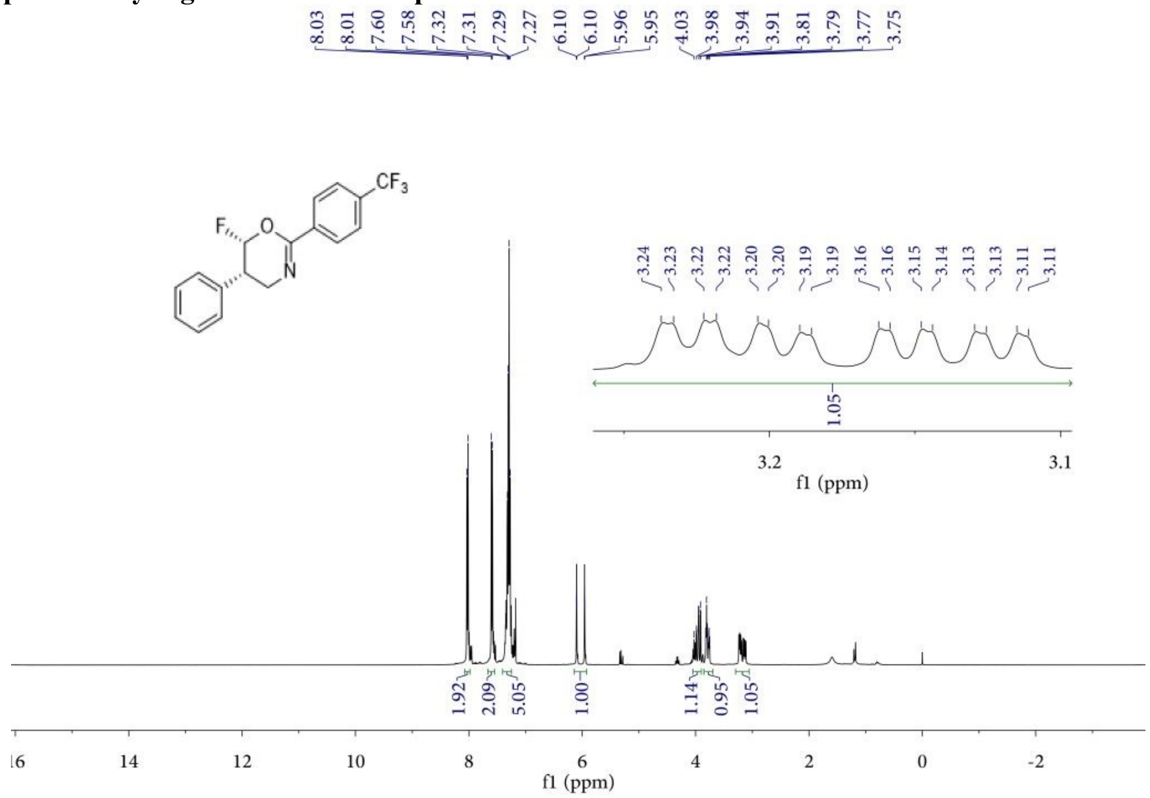

Supplementary Figure 94. ^{13}C NMR spectrum of 14b

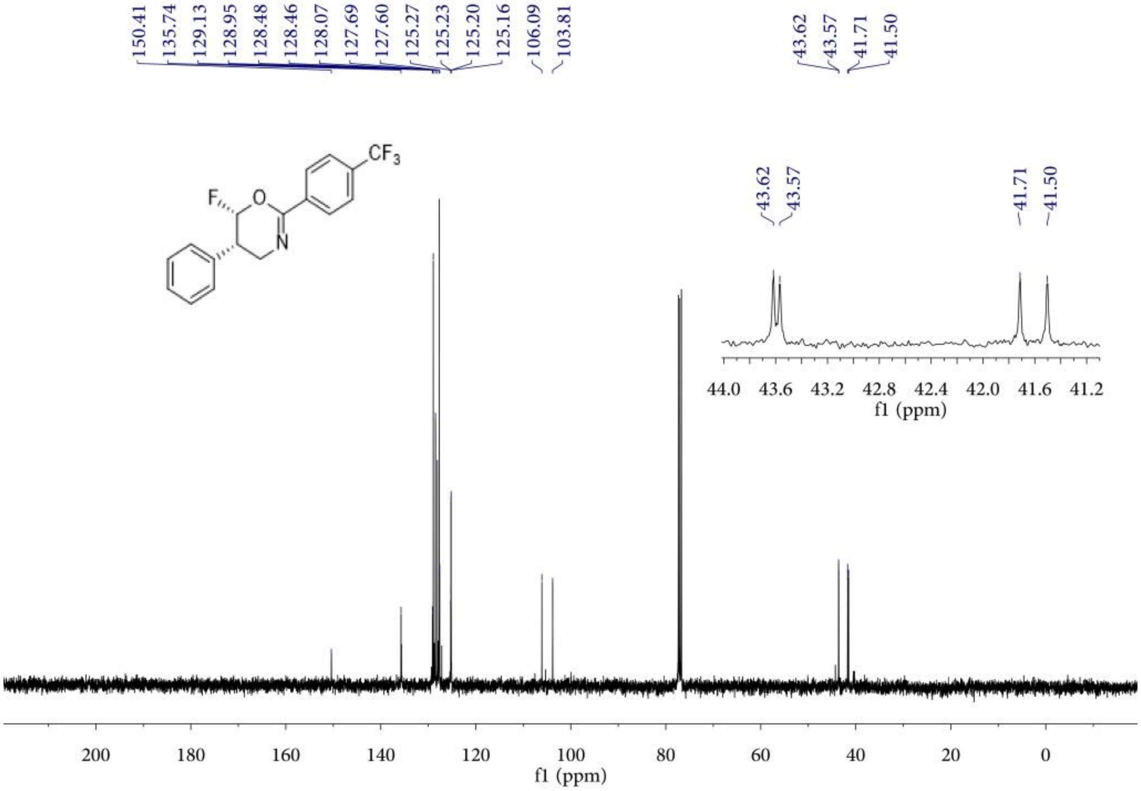

Supplementary Figure 95. ^{19}F NMR spectrum of 14b

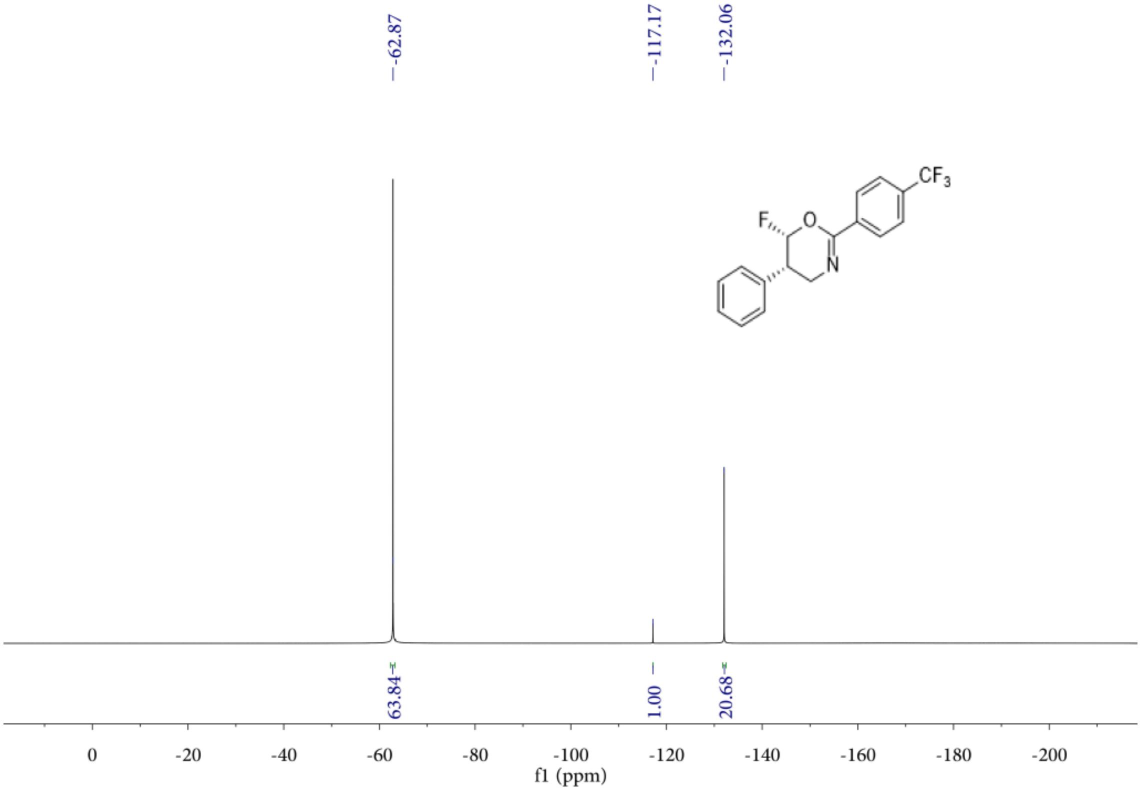

Supplementary Figure 96. ^{1}H NMR spectrum of 15b

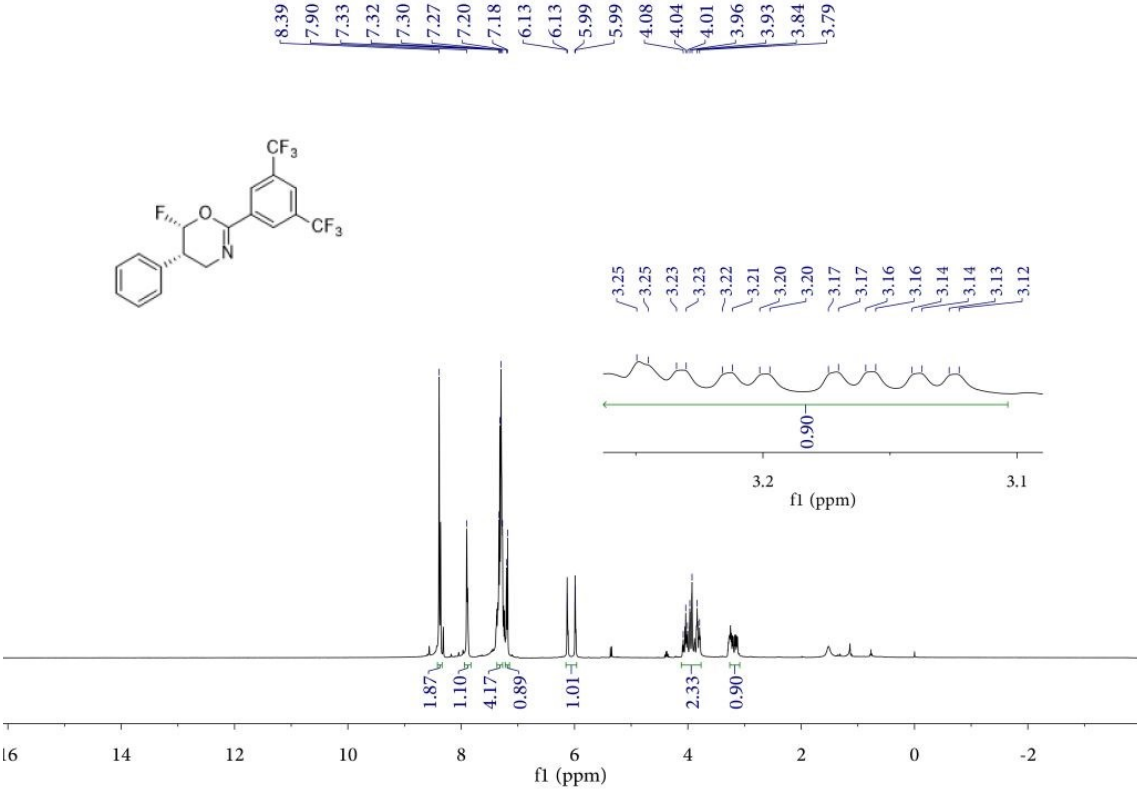

Supplementary Figure 97. ^{13}C NMR spectrum of 15b

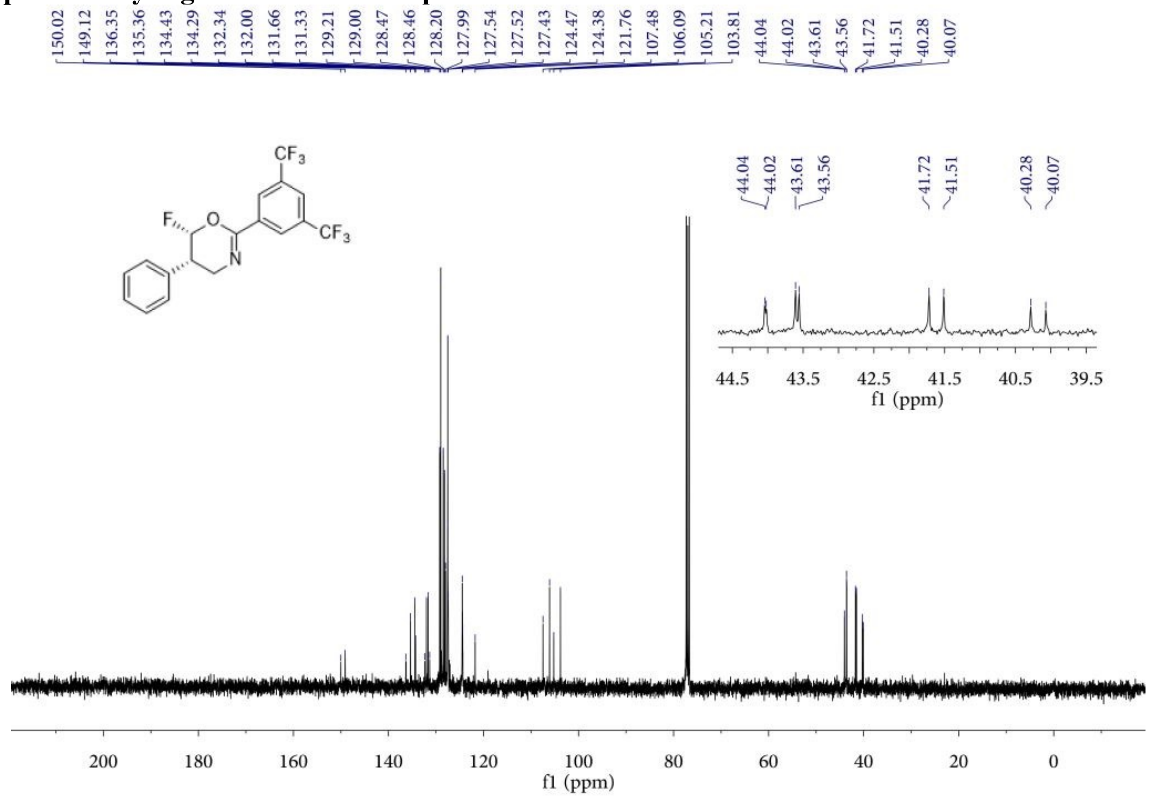

Supplementary Figure 98. ^{19}F NMR spectrum of 15b

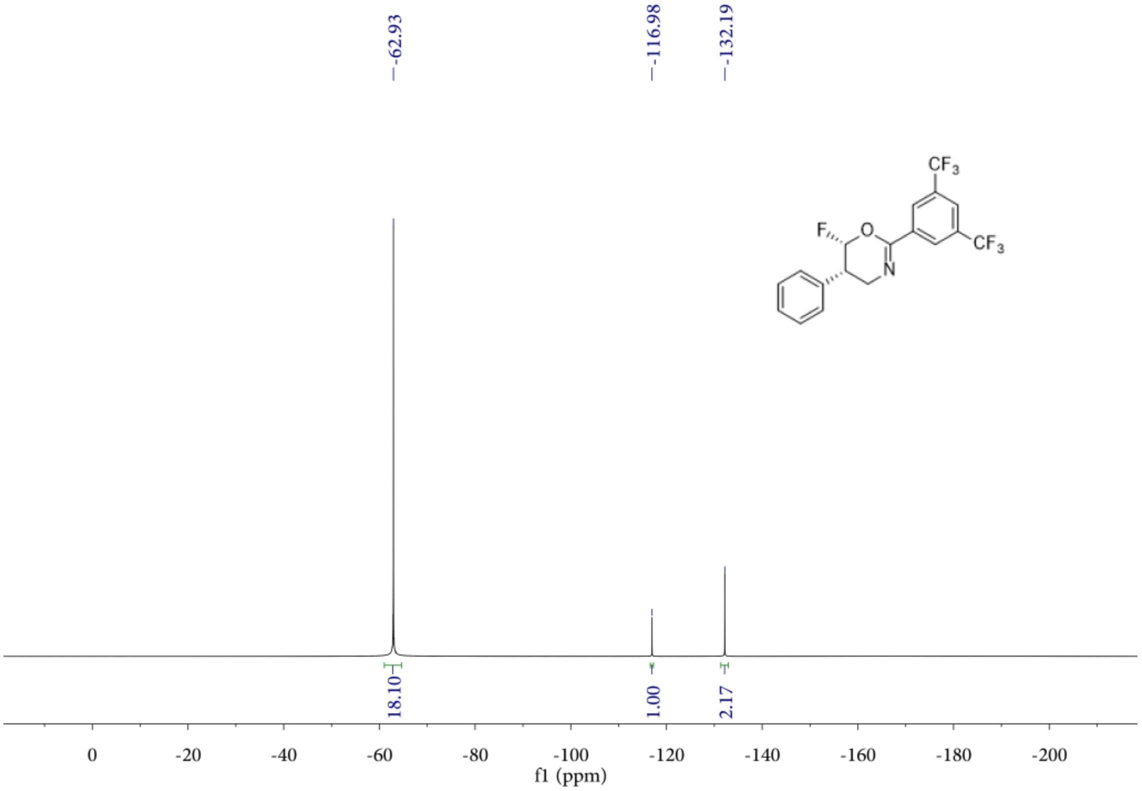

Supplementary Figure 99. ^{1}H NMR spectrum of 16b

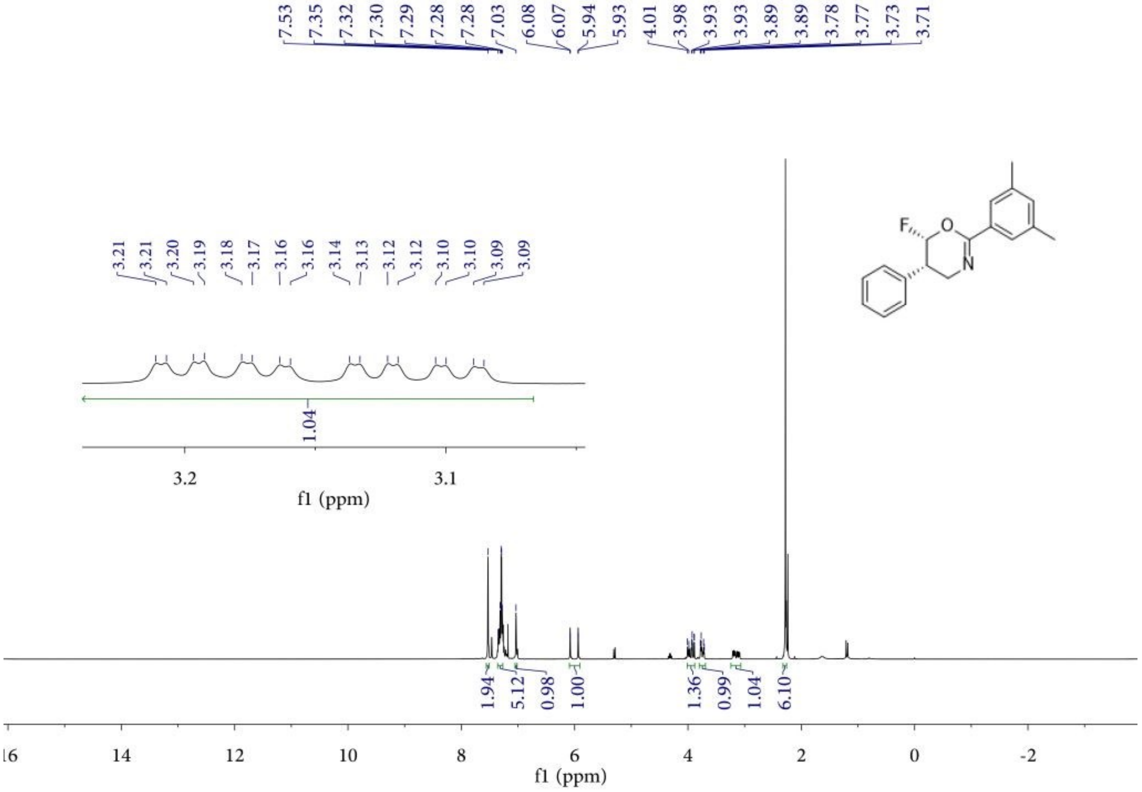

Supplementary Figure 100. ^{13}C NMR spectrum of 16b

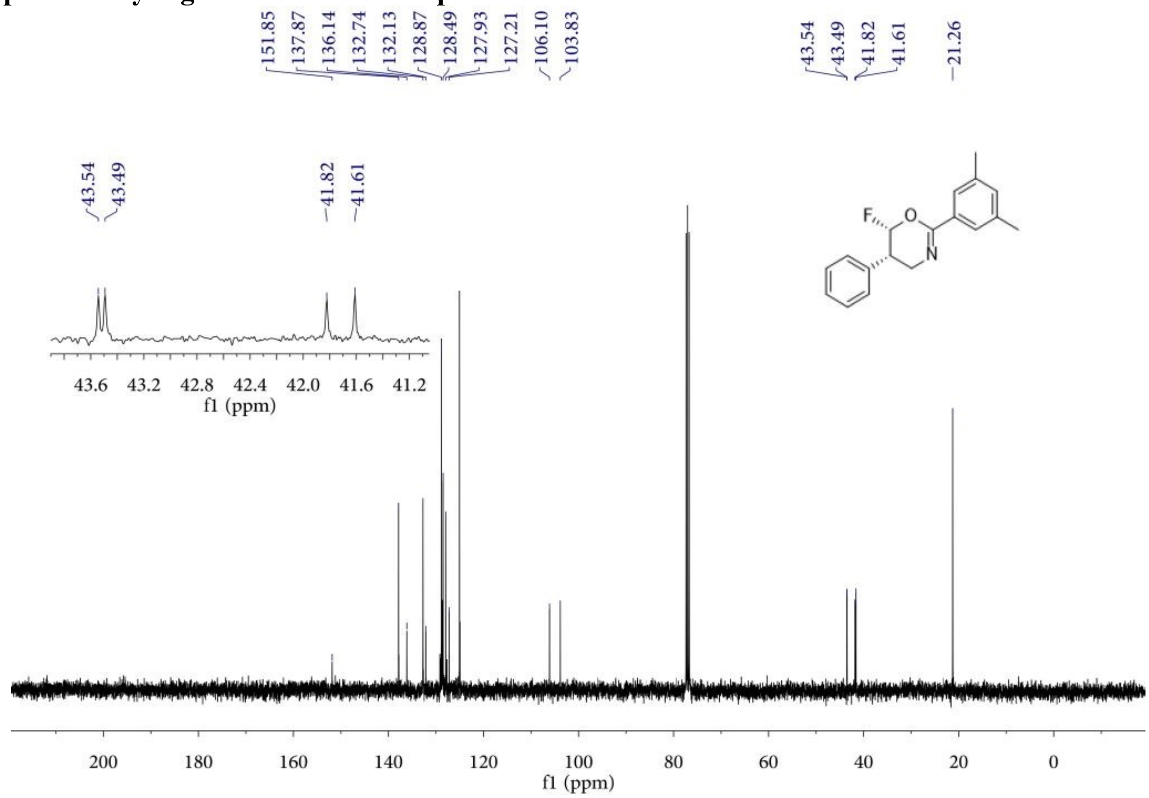

Supplementary Figure 101. ^{19}F NMR spectrum of 16b

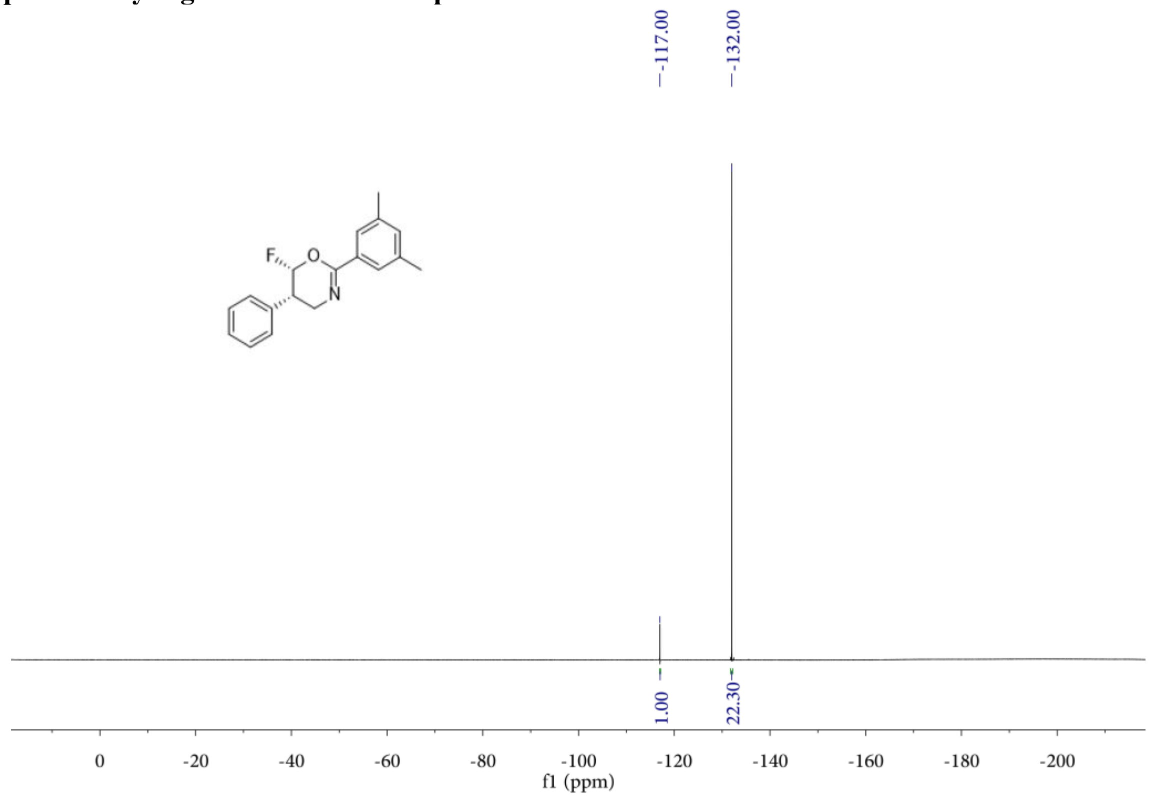

Supplementary Figure 102. ^{1}H NMR spectrum of 17b

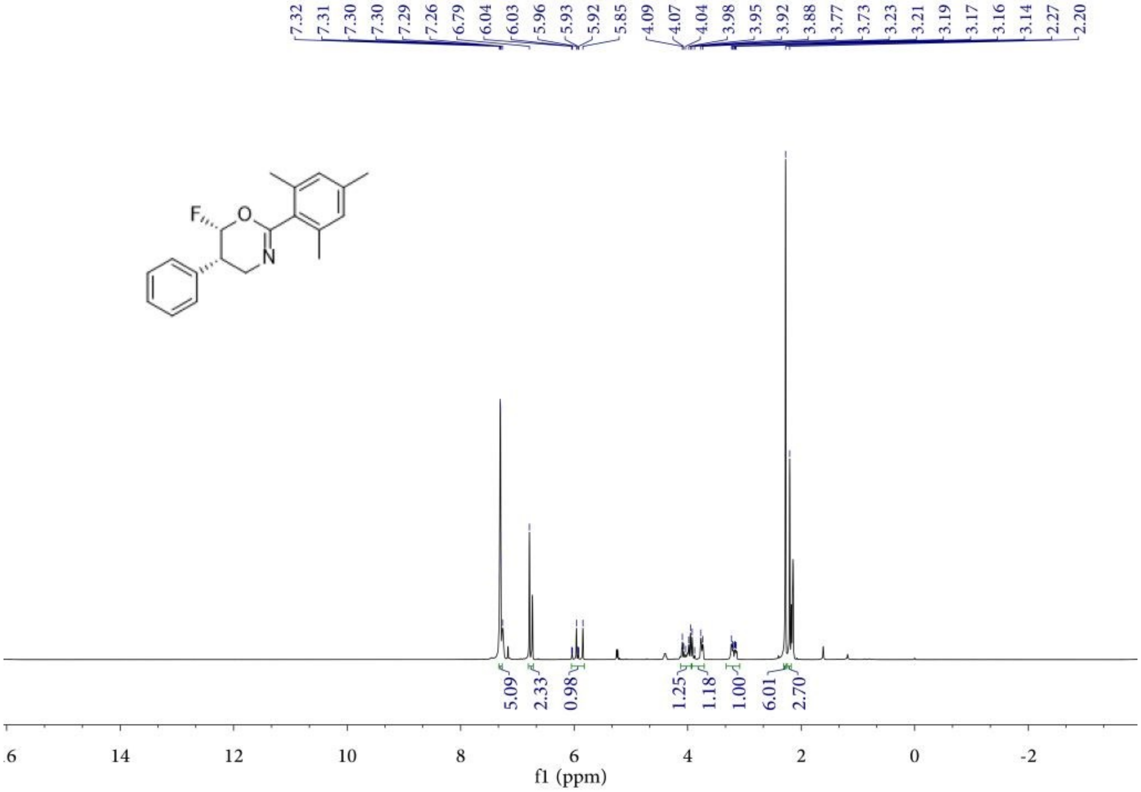

Supplementary Figure 103. ^{13}C NMR spectrum of 17b

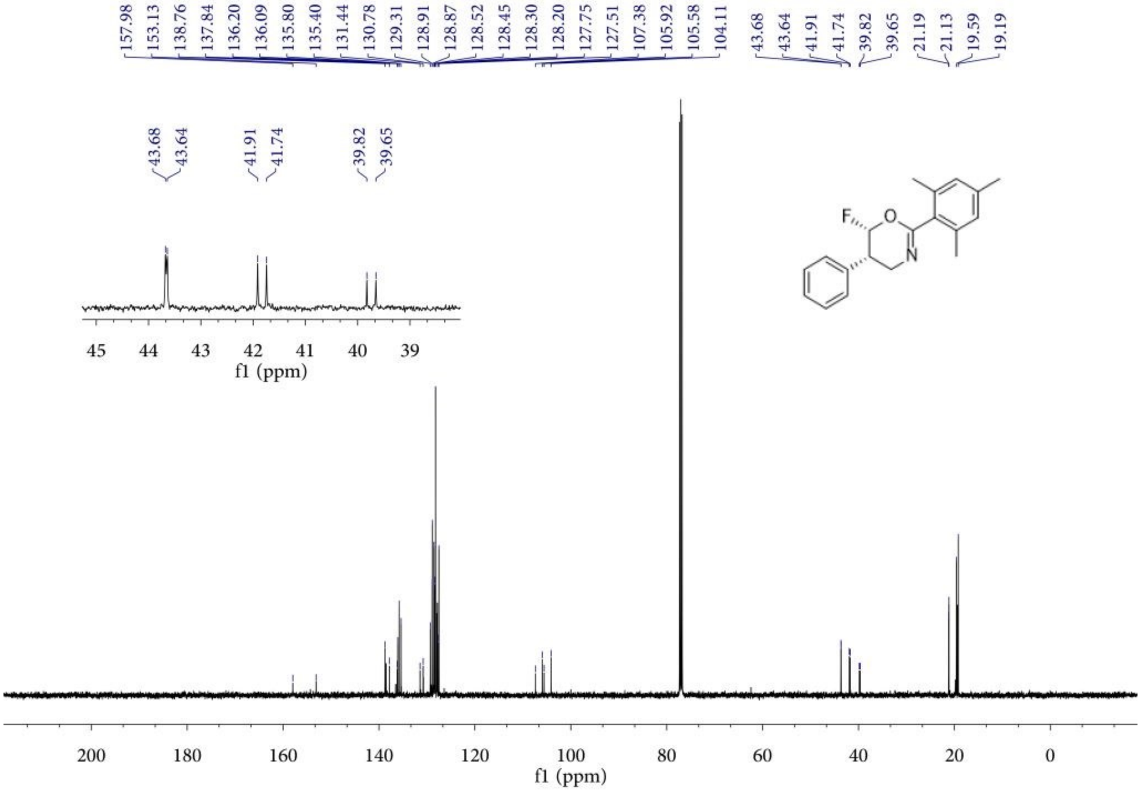

Supplementary Figure 104. ^{19}F NMR spectrum of 17b

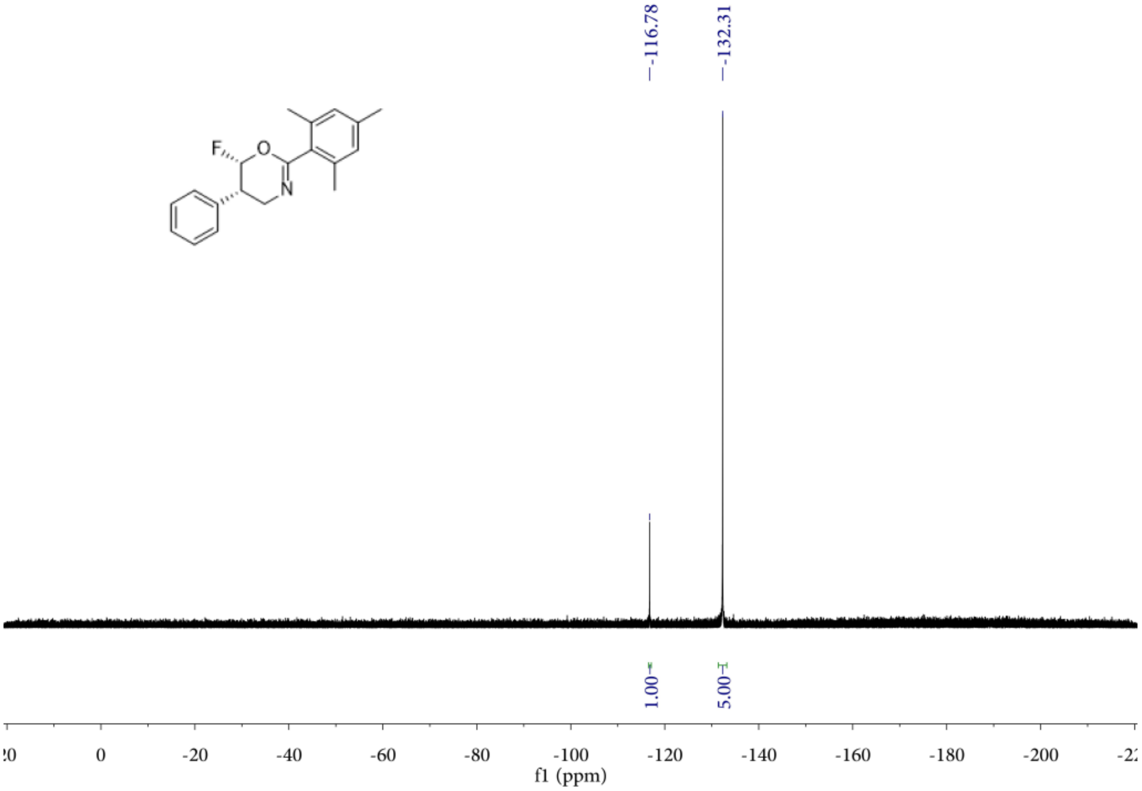

Supplementary Figure 105. ^{1}H NMR spectrum of 18b

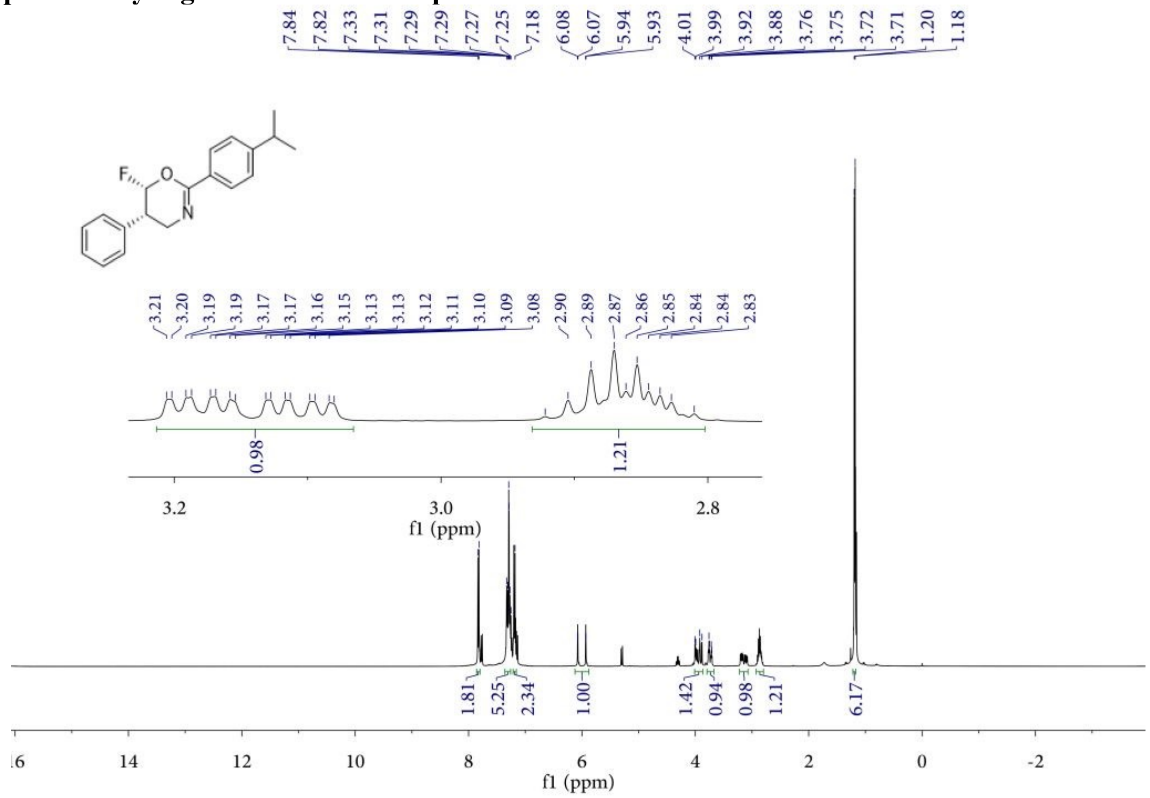

Supplementary Figure 106. ^{13}C NMR spectrum of 18b

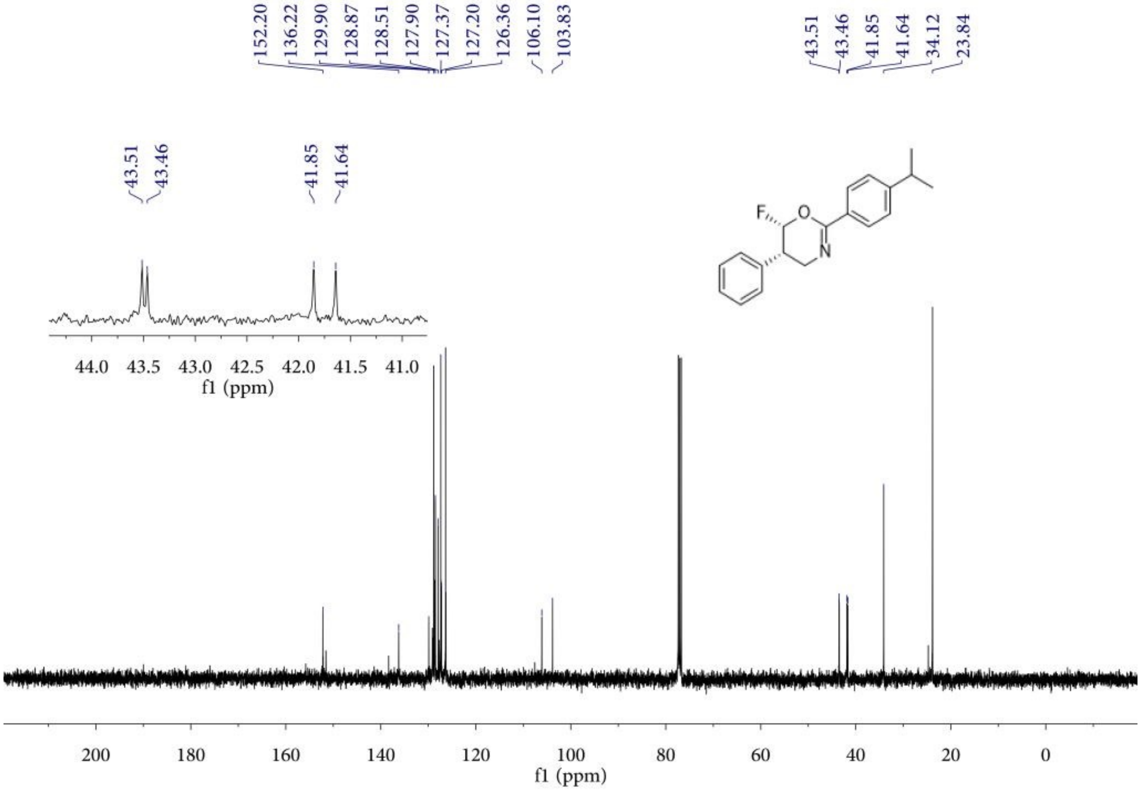

Supplementary Figure 107. ^{19}F NMR spectrum of 18b

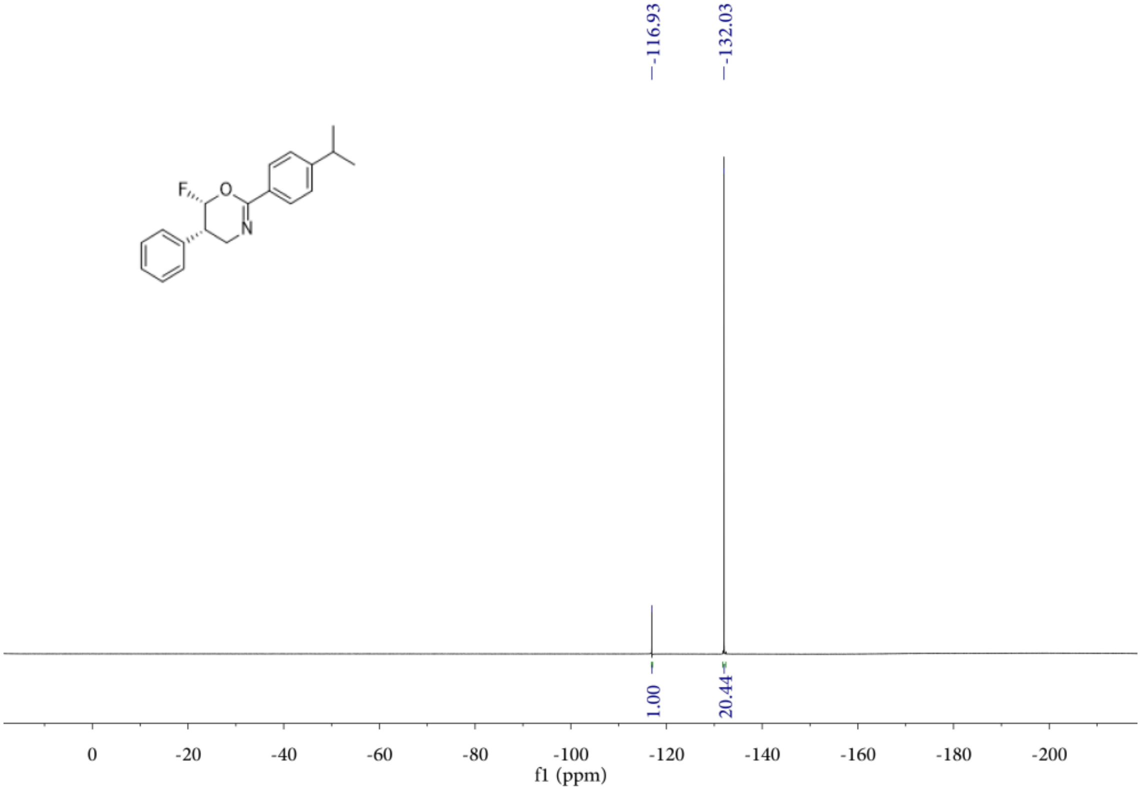

Supplementary Figure 108. ^{1}H NMR spectrum of 19b

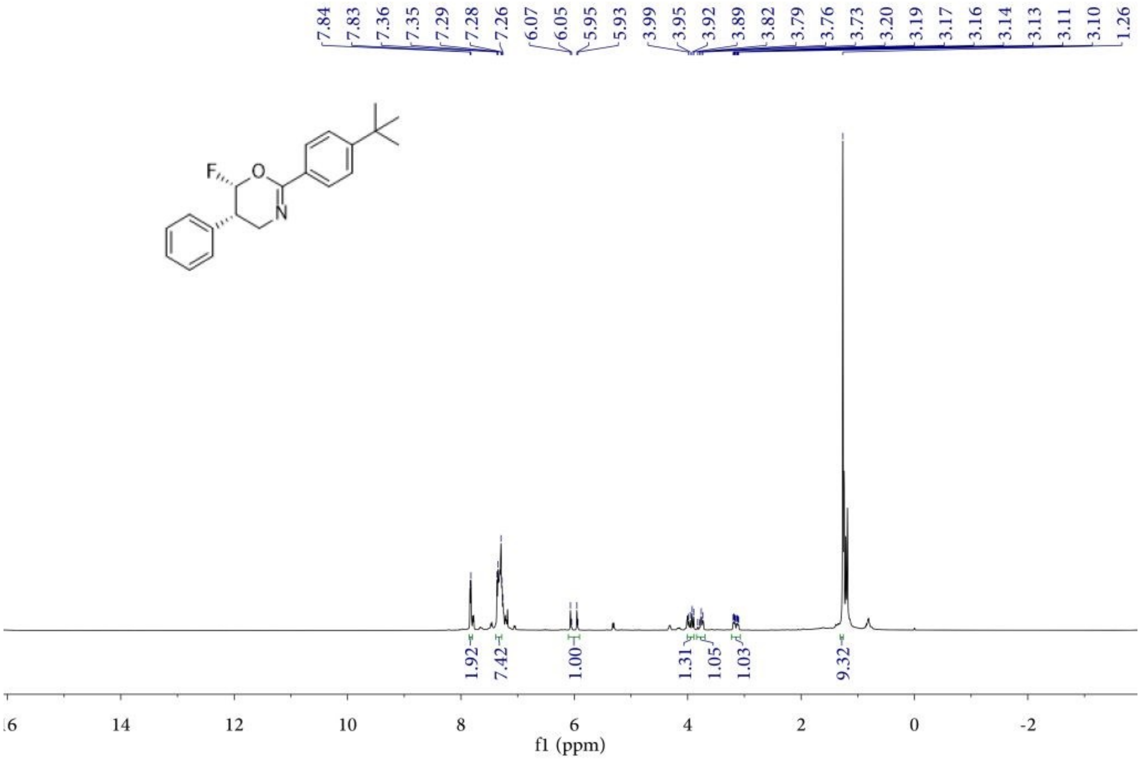

Supplementary Figure 109. ^{13}C NMR spectrum of 19b

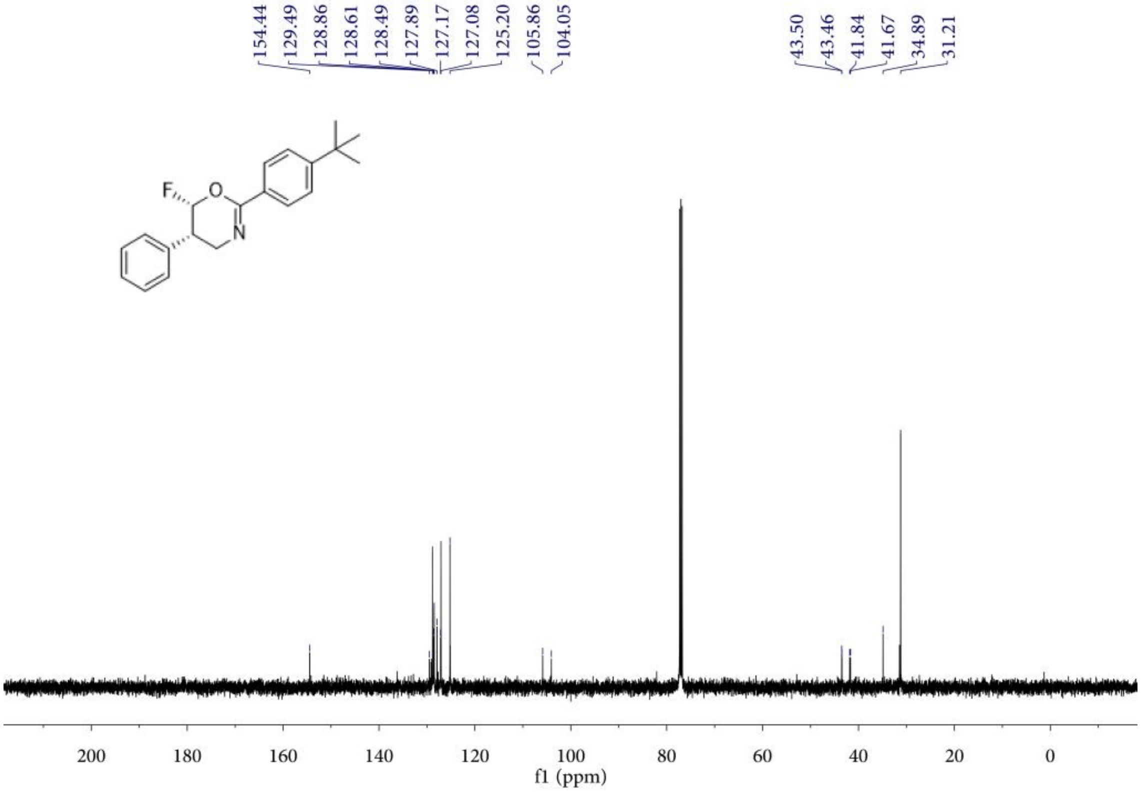

Supplementary Figure 110. ^{19}F NMR spectrum of 19b

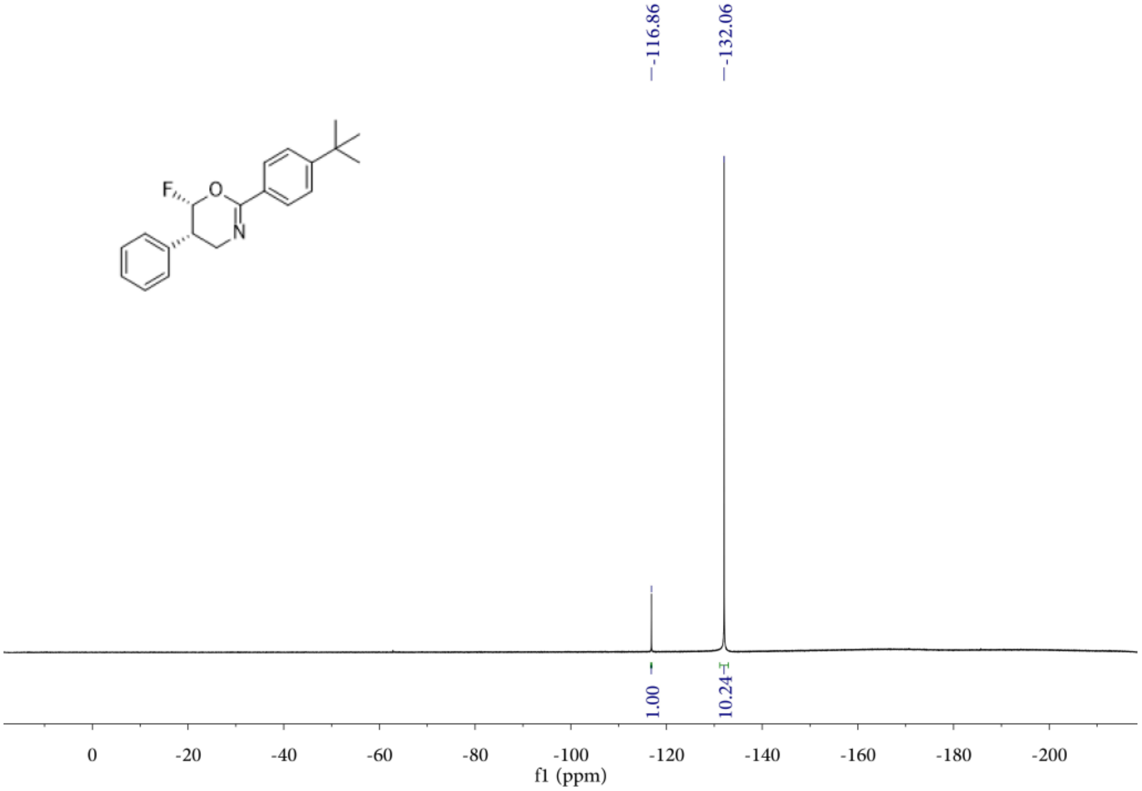

Supplementary Figure 111. ^{1}H NMR spectrum of 20b

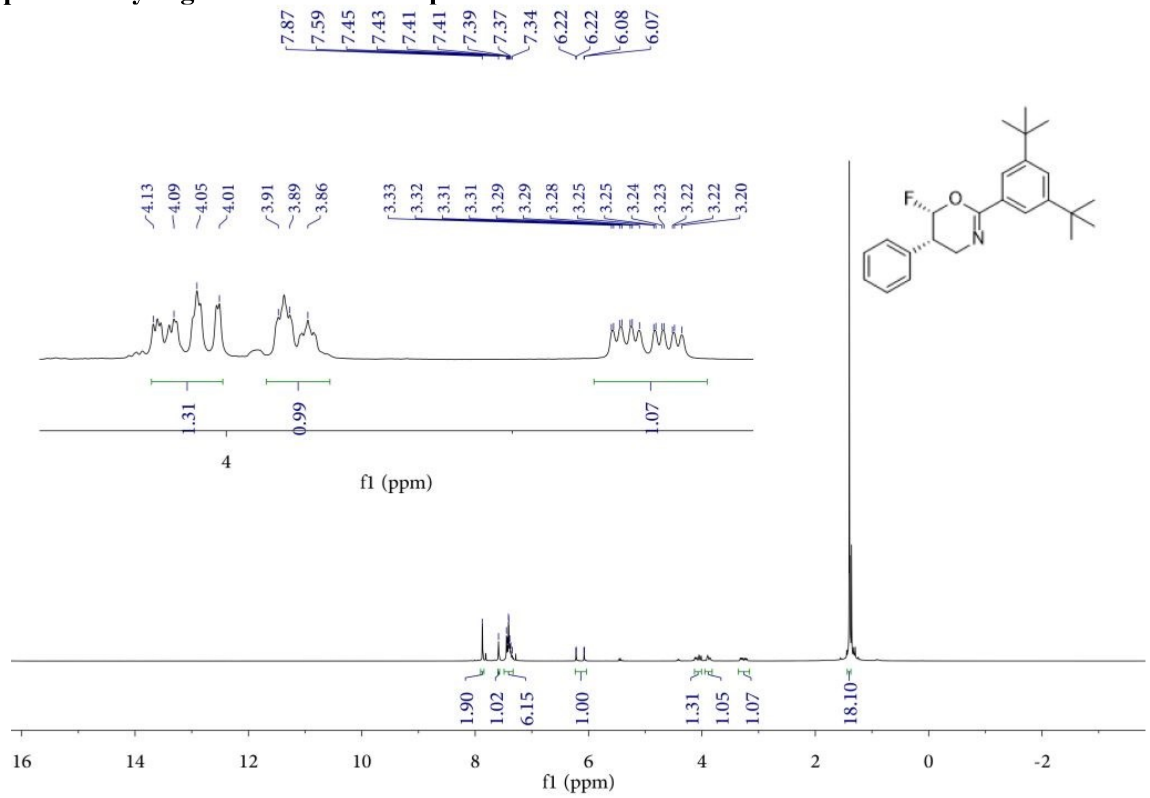

Supplementary Figure 112. ^{13}C NMR spectrum of 20b

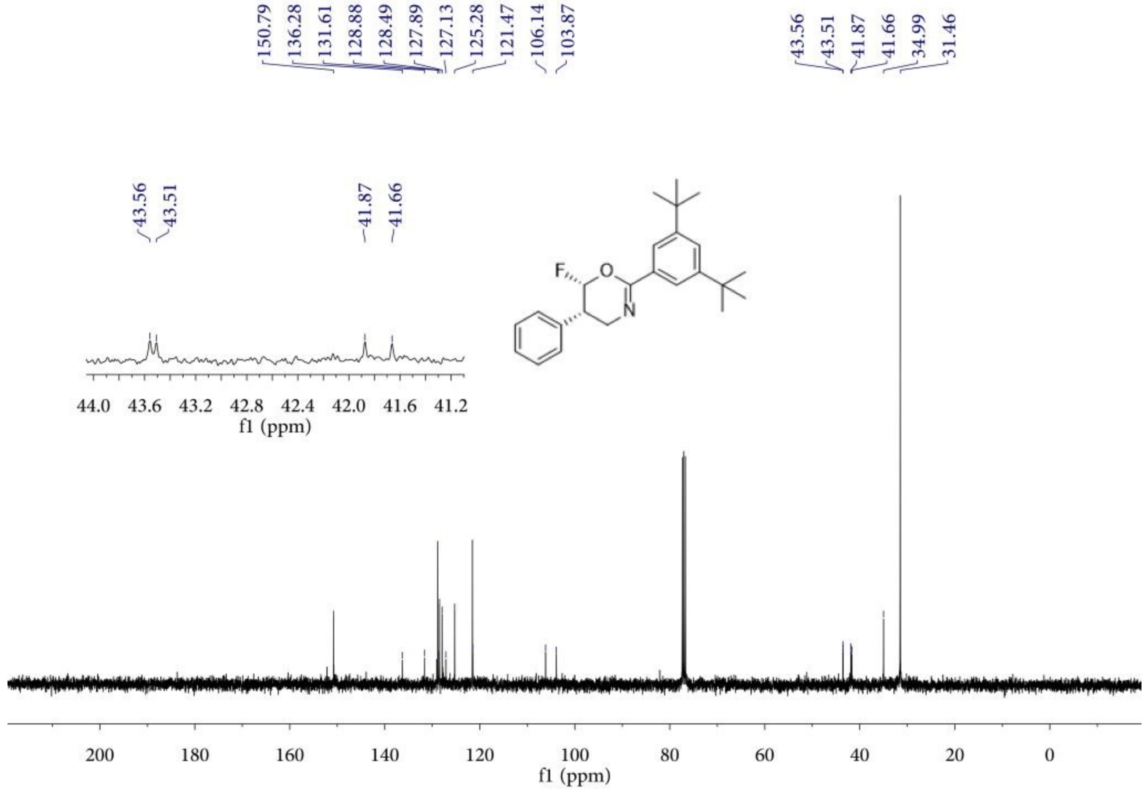

Supplementary Figure 113. ^{19}F NMR spectrum of 20b

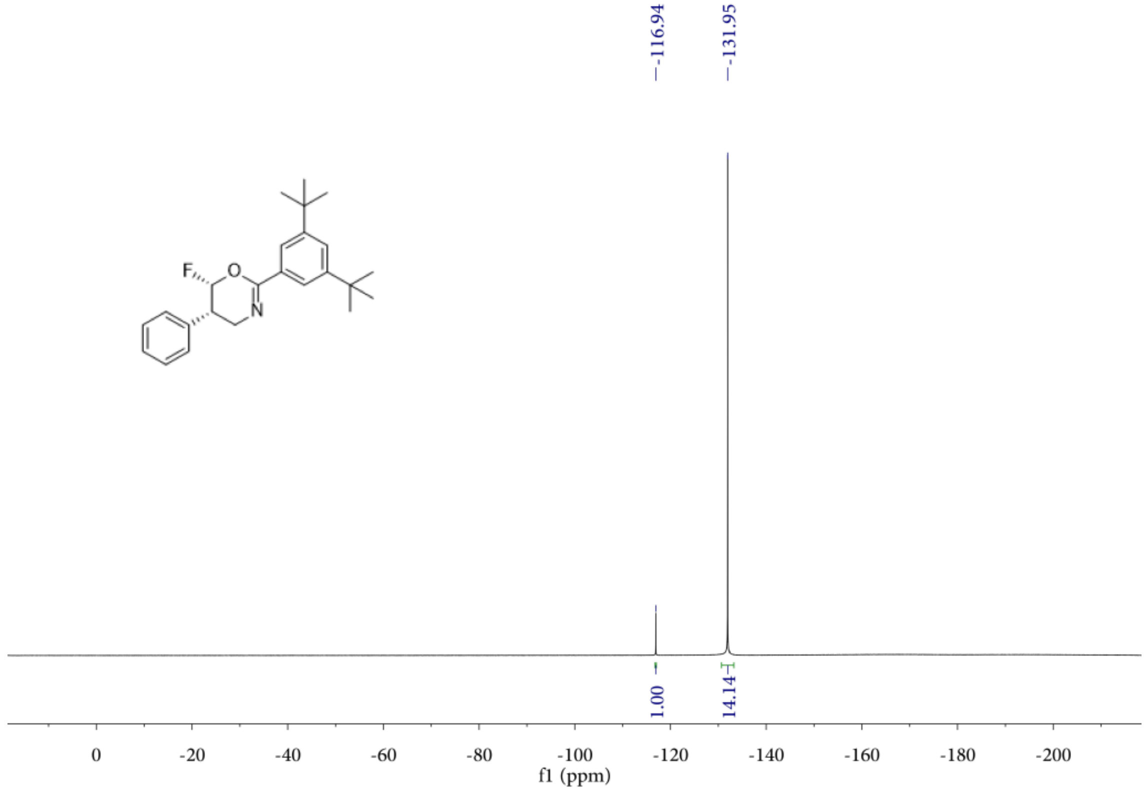

Supplementary Figure 114. ^{1}H NMR spectrum of 21b

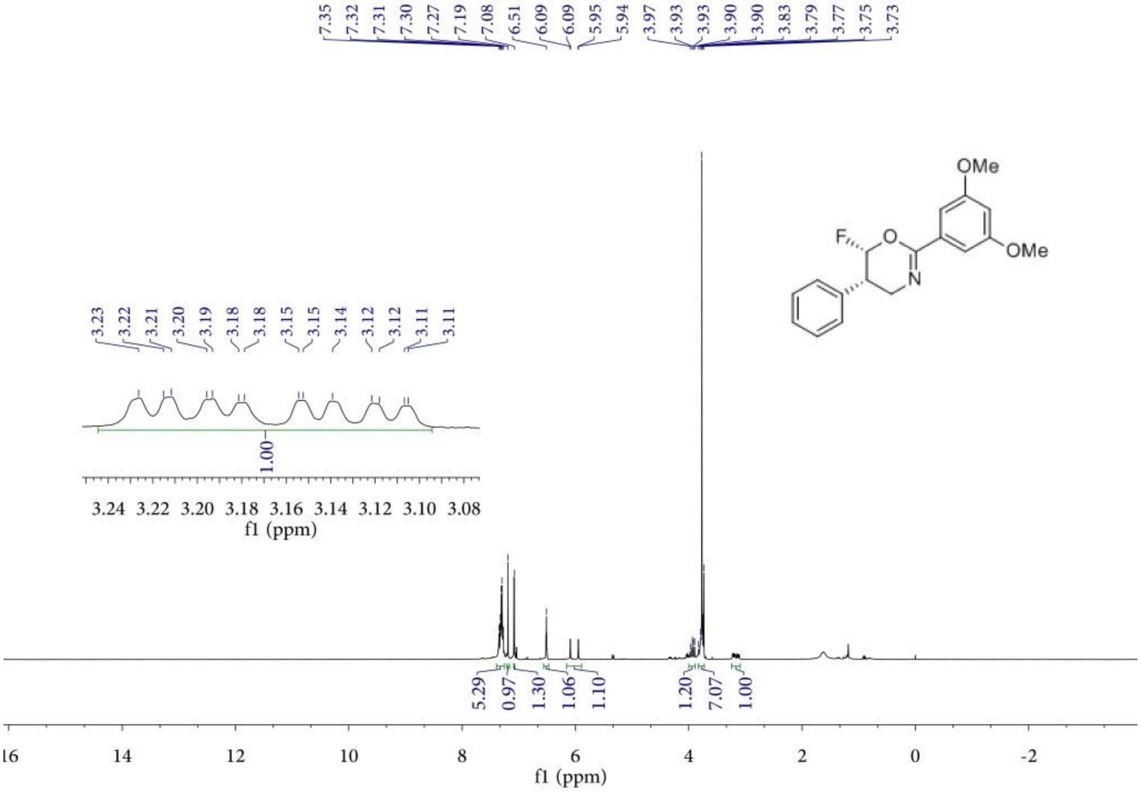

Supplementary Figure 115. ^{13}C NMR spectrum of 21b

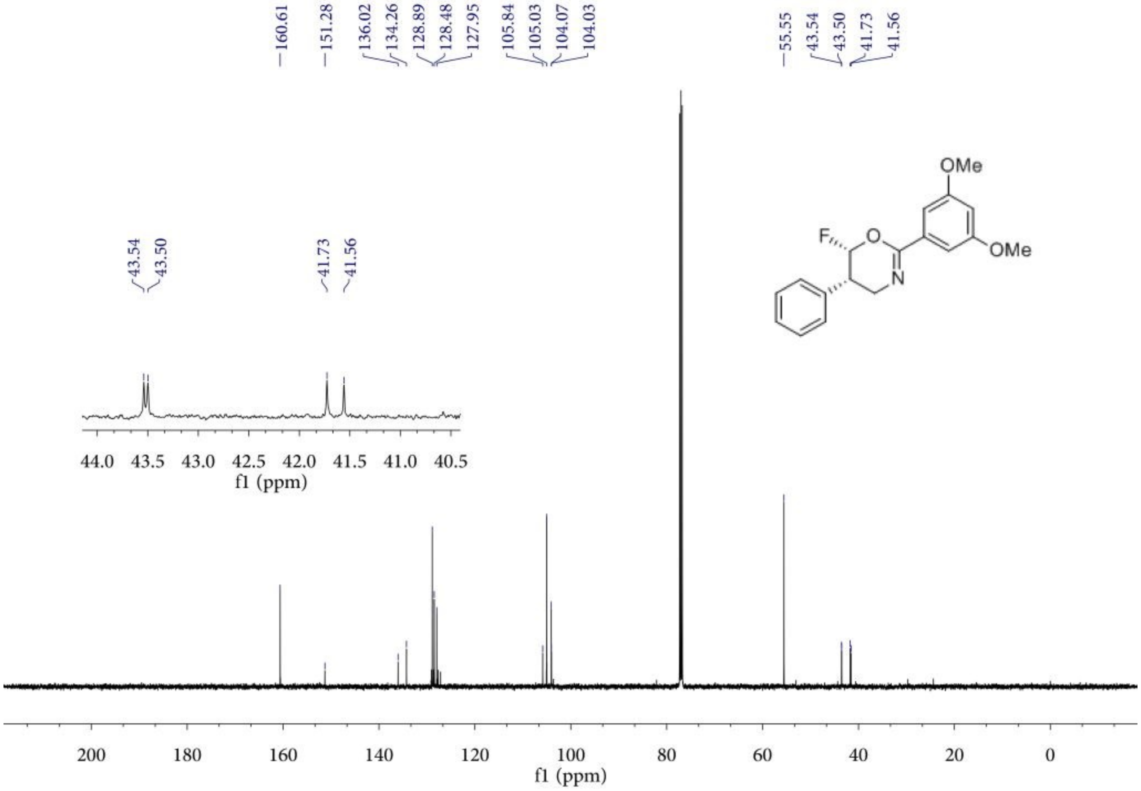

Supplementary Figure 116. ^{19}F NMR spectrum of 21b

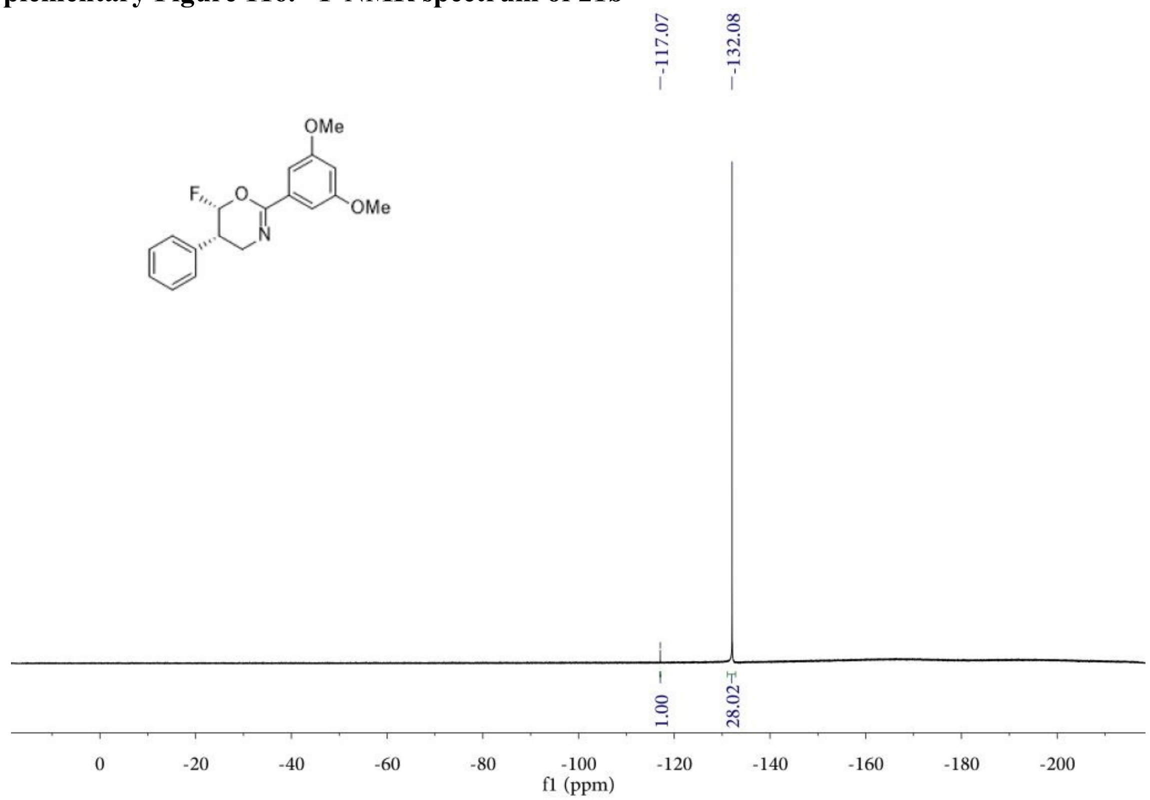

Supplementary Figure 117. ^{1}H NMR spectrum of 22b

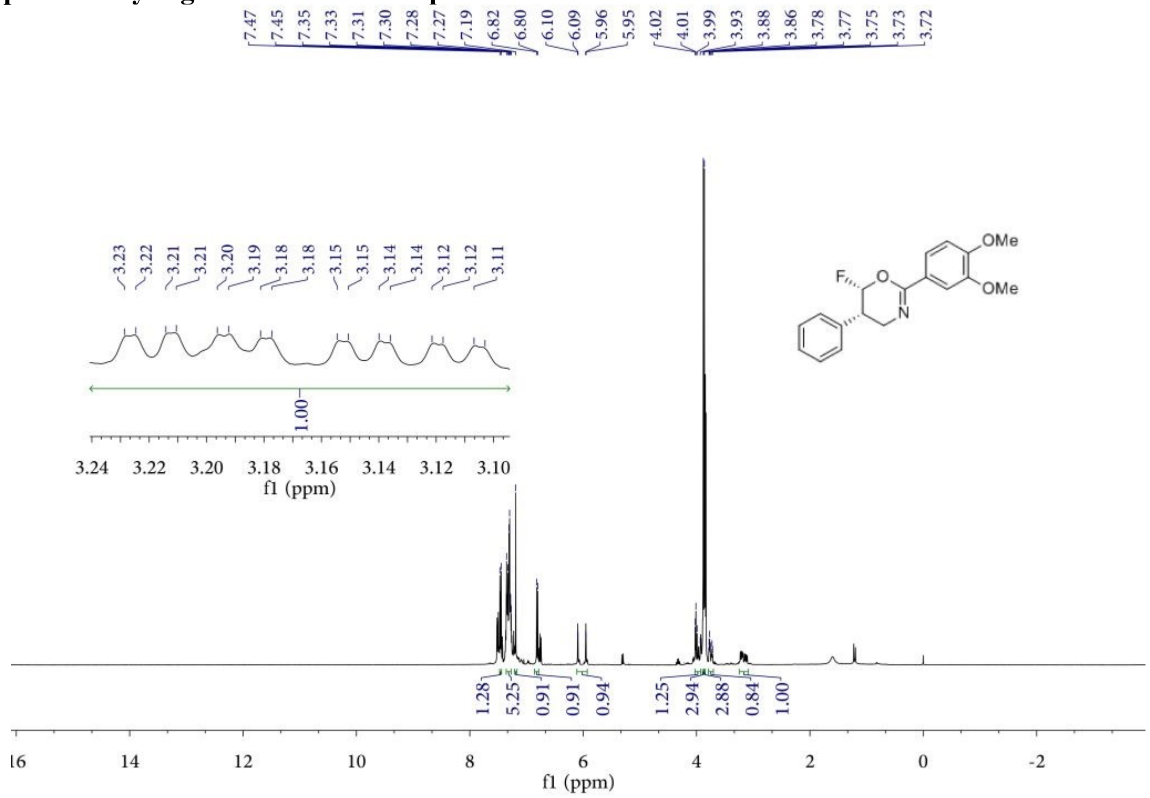

Supplementary Figure 118. ^{13}C NMR spectrum of 22b

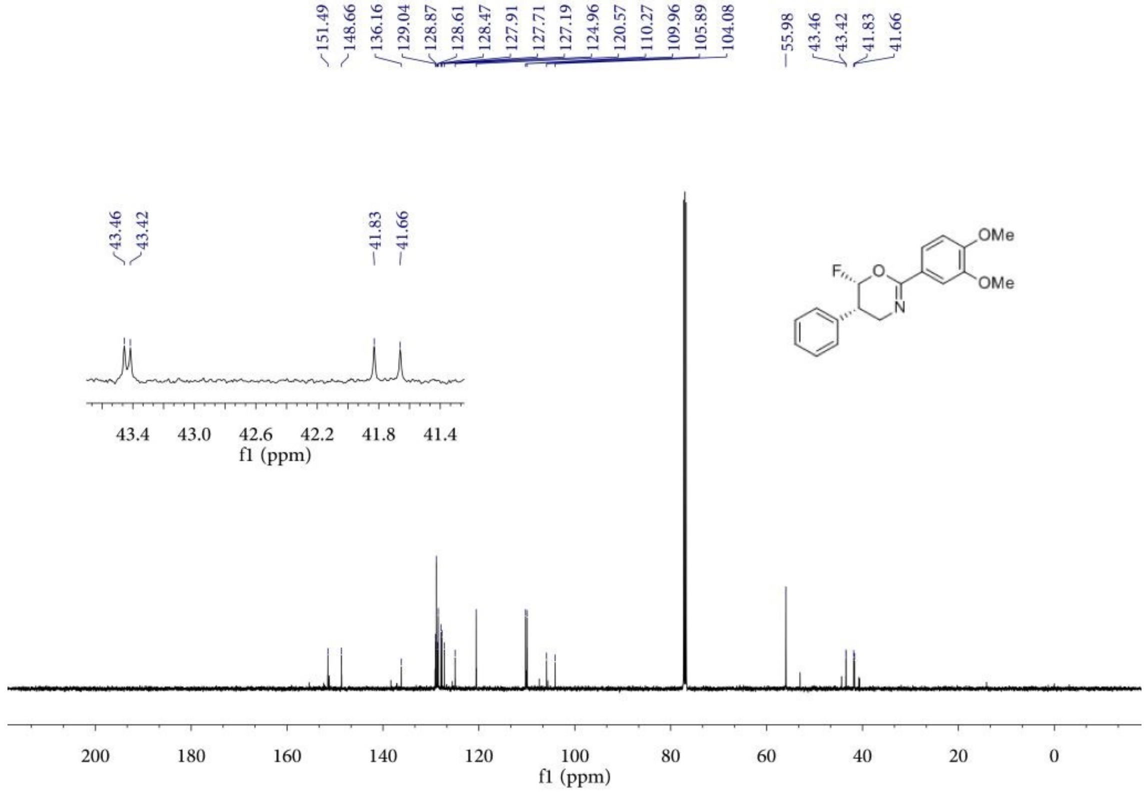

Supplementary Figure 119. ^{19}F NMR spectrum of 22b

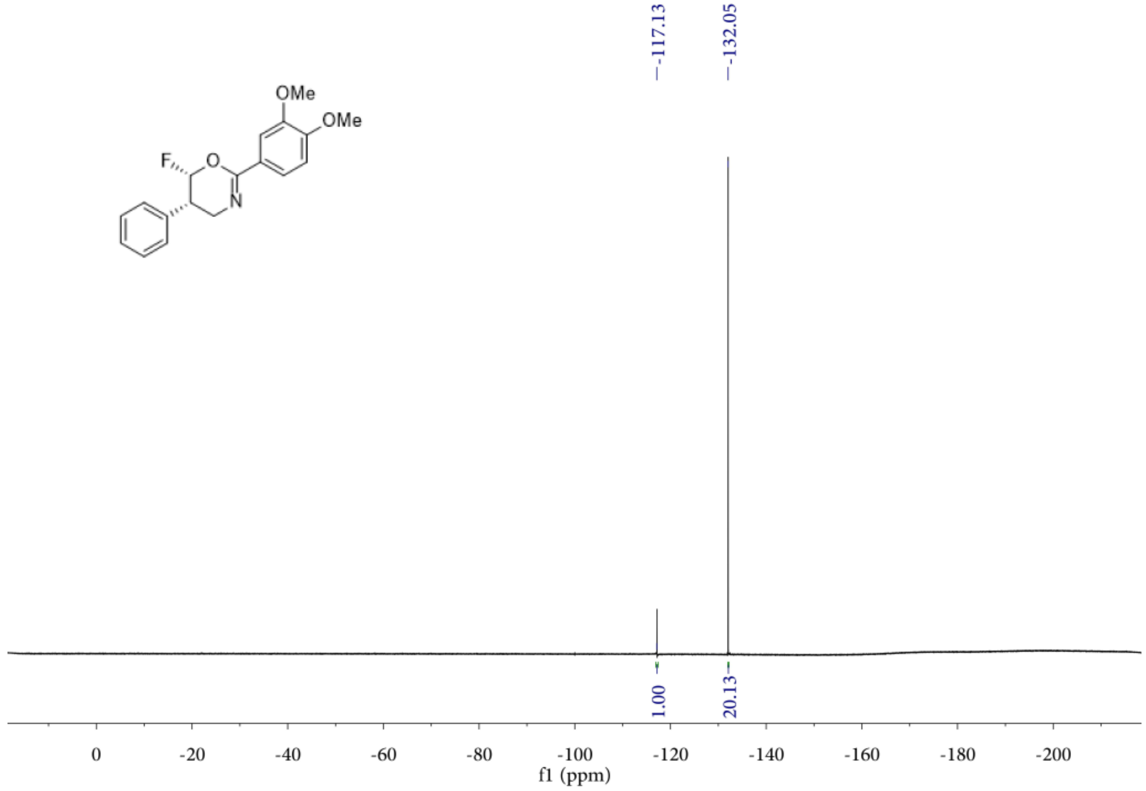

Supplementary Figure 120. ^{1}H NMR spectrum of 23b

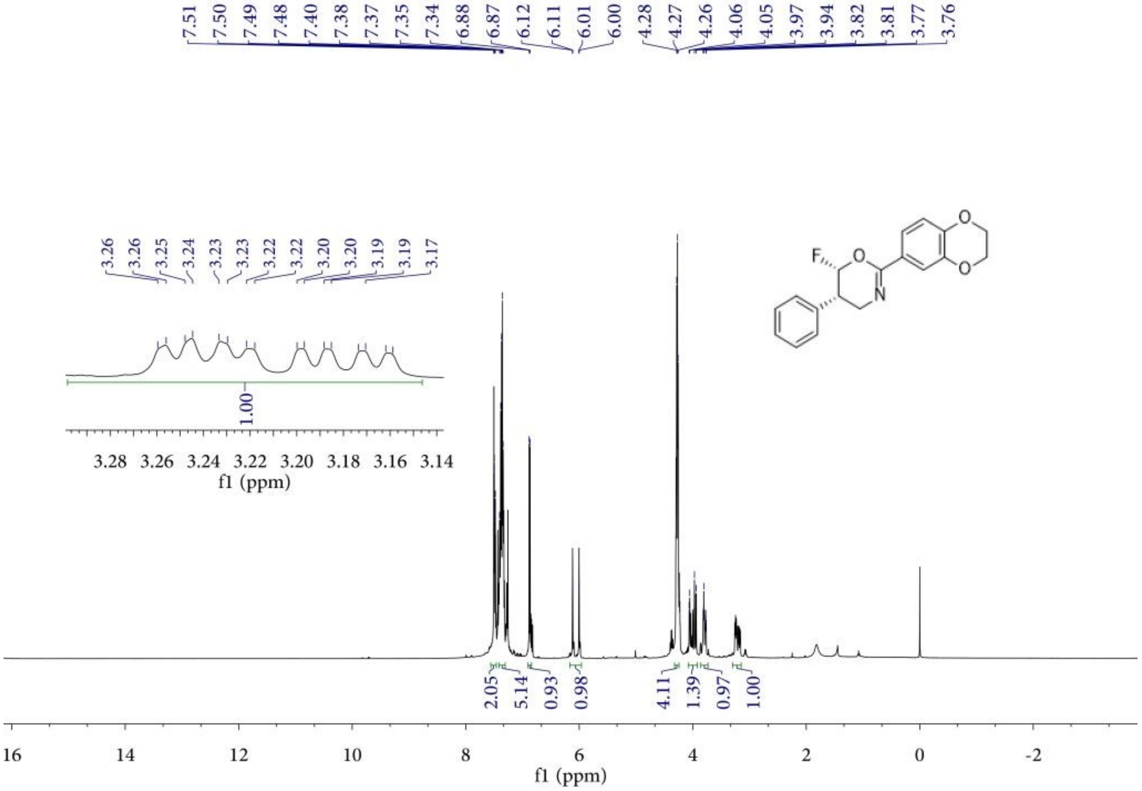

Supplementary Figure 121. ^{13}C NMR spectrum of 23b

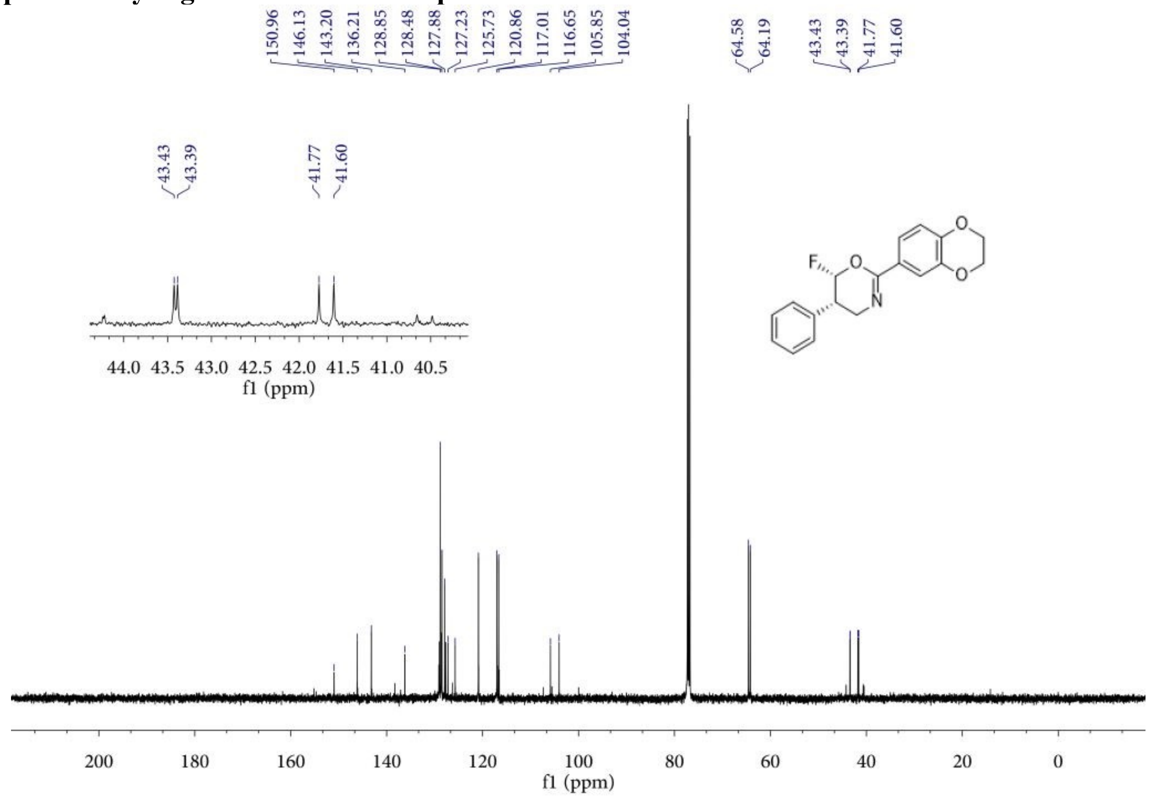

Supplementary Figure 122. ^{19}F NMR spectrum of 23b

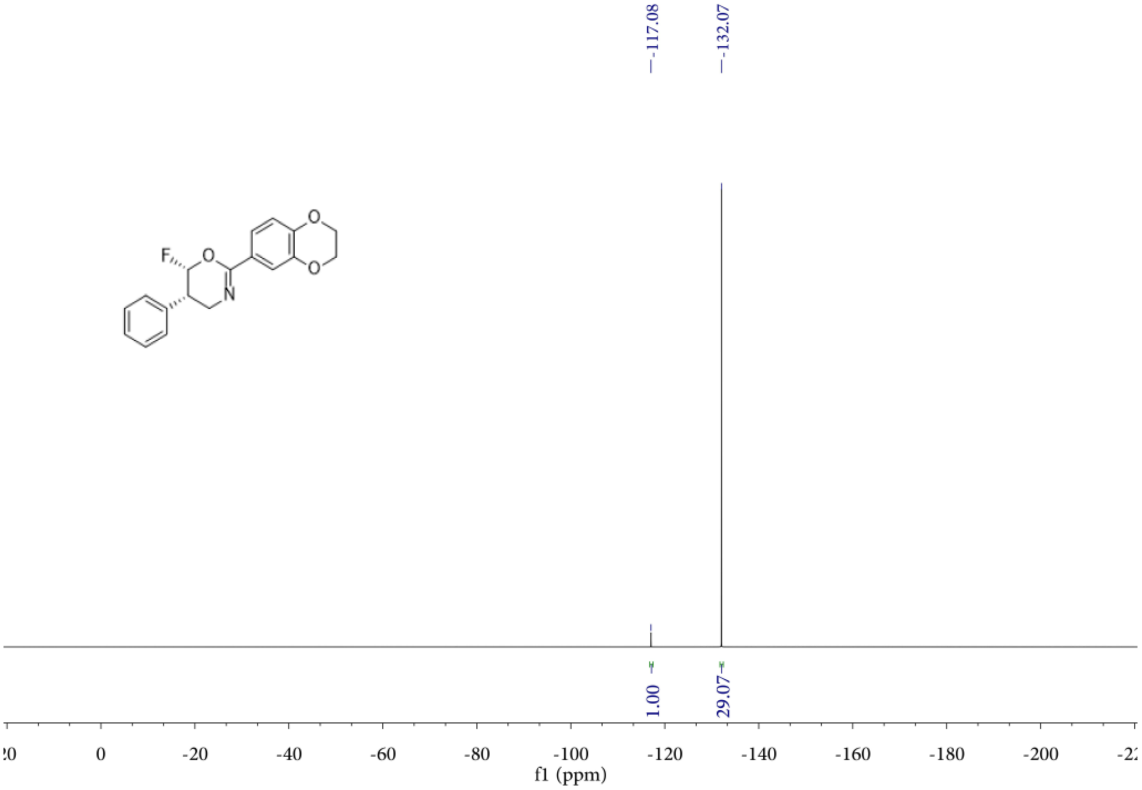

Supplementary Figure 123. ^{1}H NMR spectrum of 24b

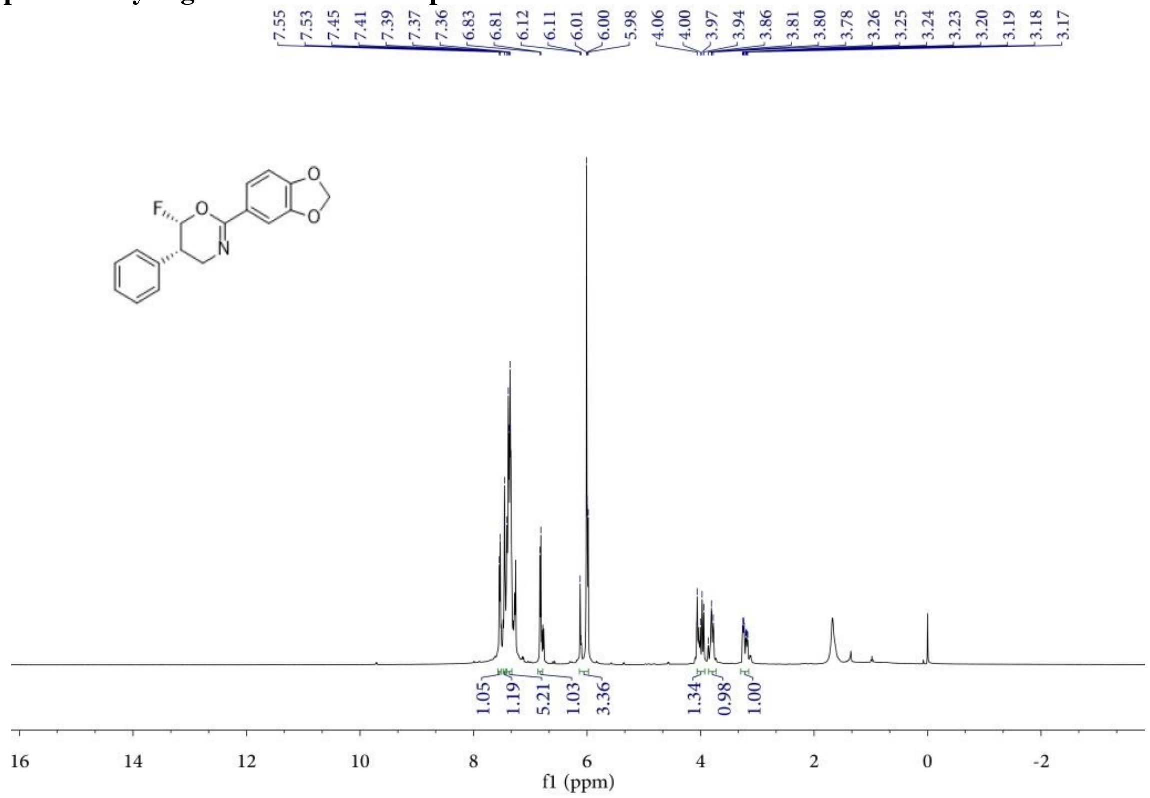

Supplementary Figure 124. ^{13}C NMR spectrum of 24b

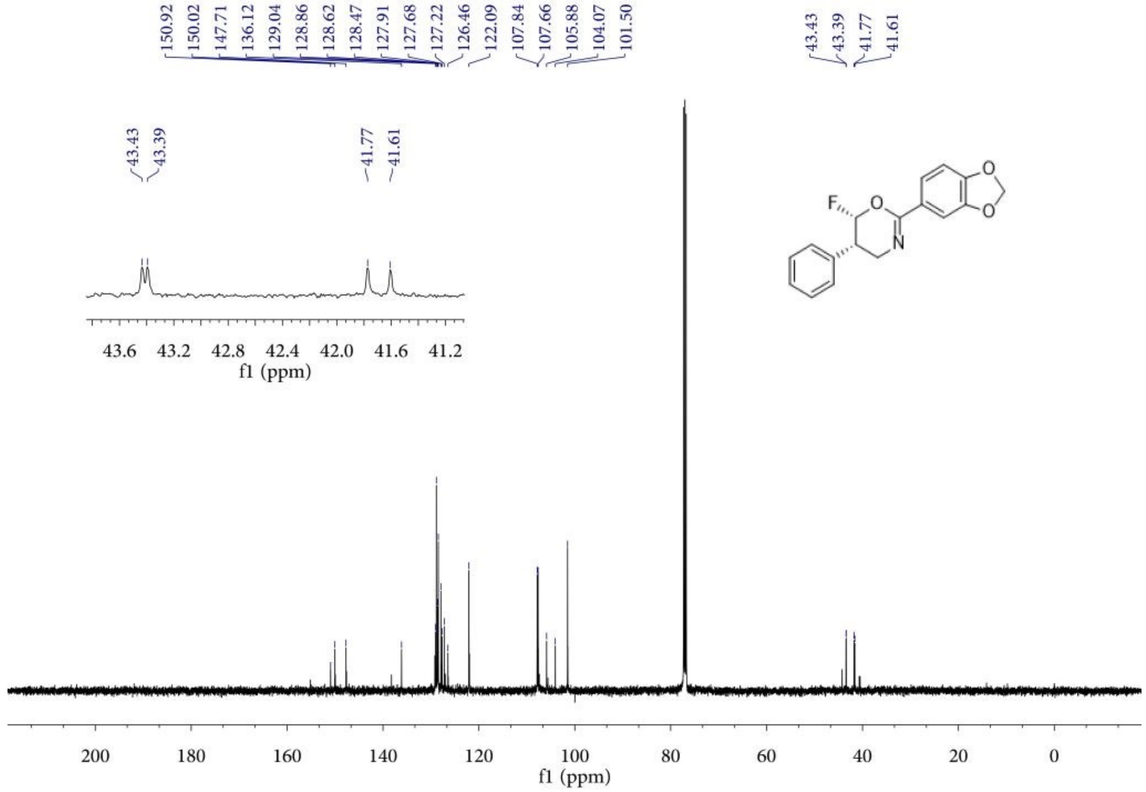

Supplementary Figure 125. ^{19}F NMR spectrum of 24b

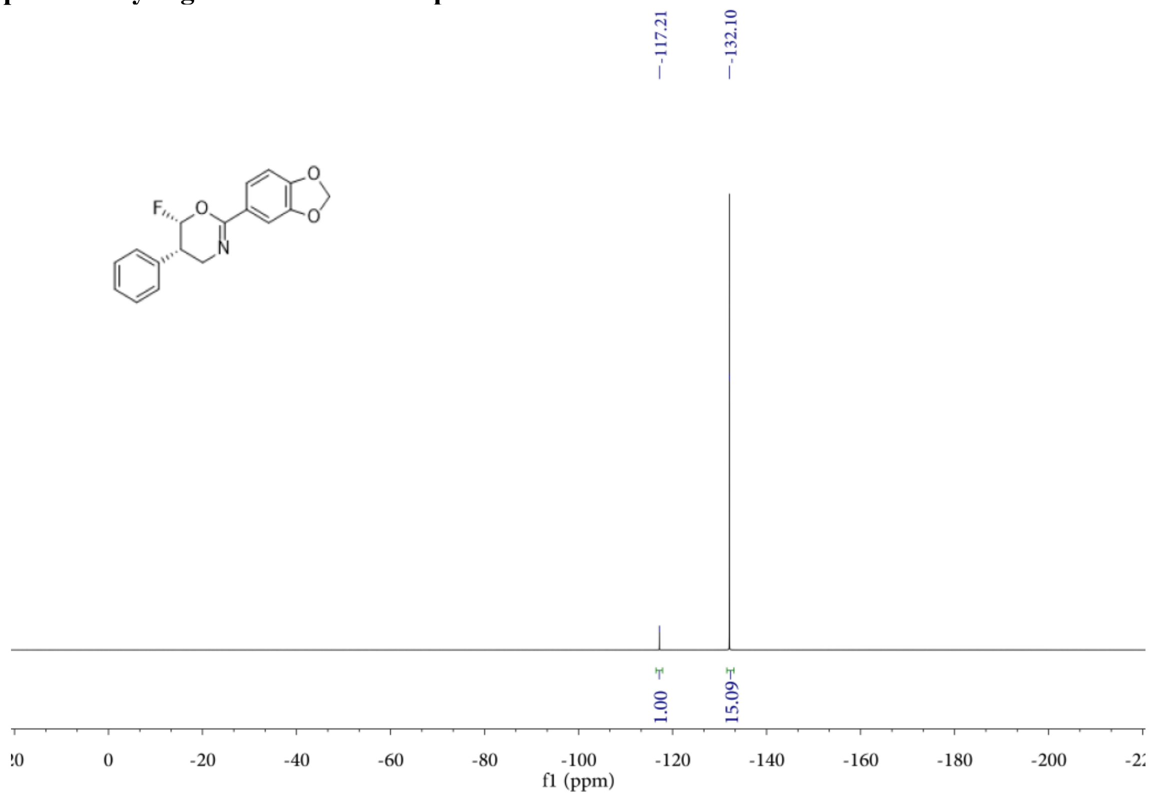

Supplementary Figure 126. ^{1}H NMR spectrum of 25b

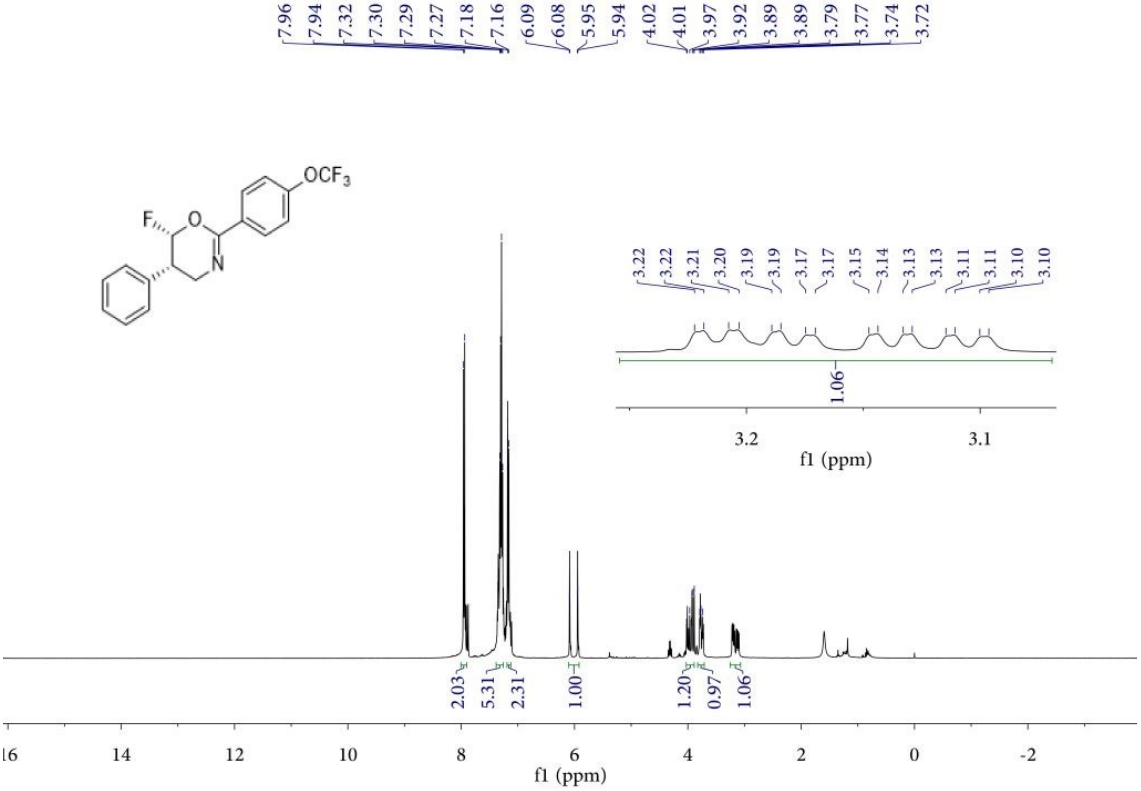

Supplementary Figure 127. ^{13}C NMR spectrum of 25b

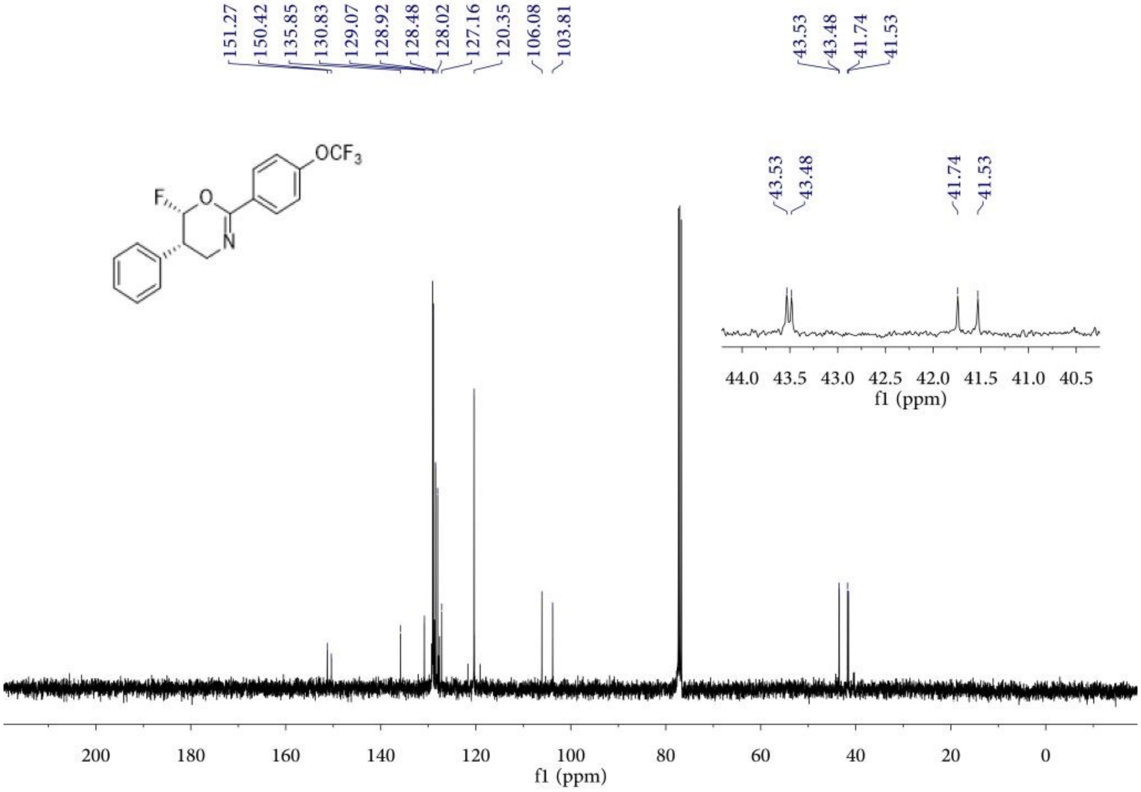

Supplementary Figure 128. ^{19}F NMR spectrum of 25b

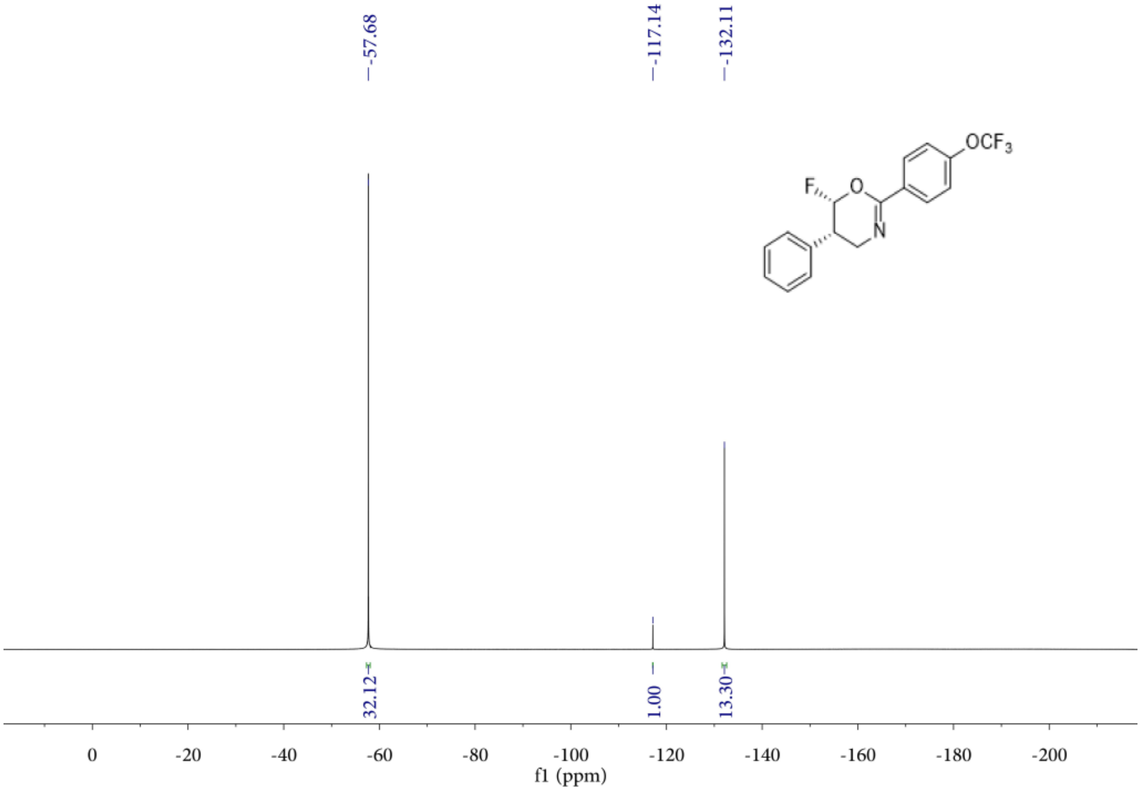

Supplementary Figure 129. ^{1}H NMR spectrum of 26b

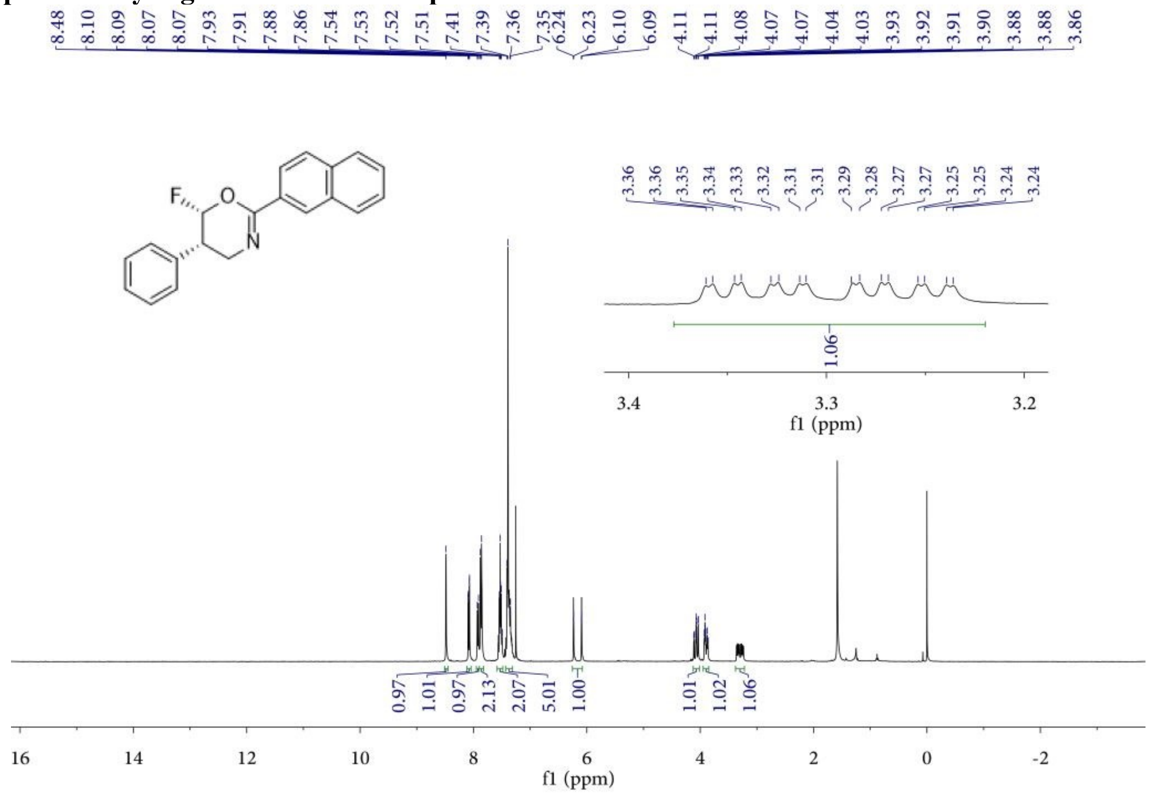

Supplementary Figure 130. ^{13}C NMR spectrum of 26b

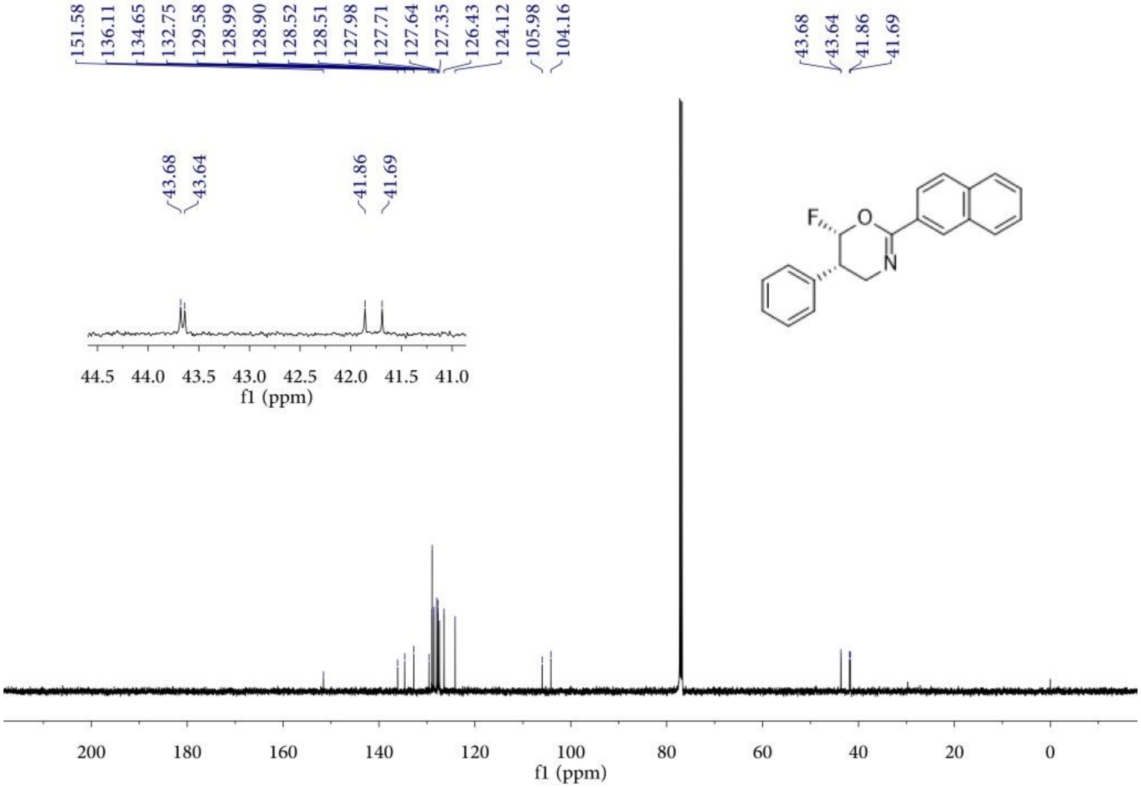

Supplementary Figure 131. ^{19}F NMR spectrum of 26b

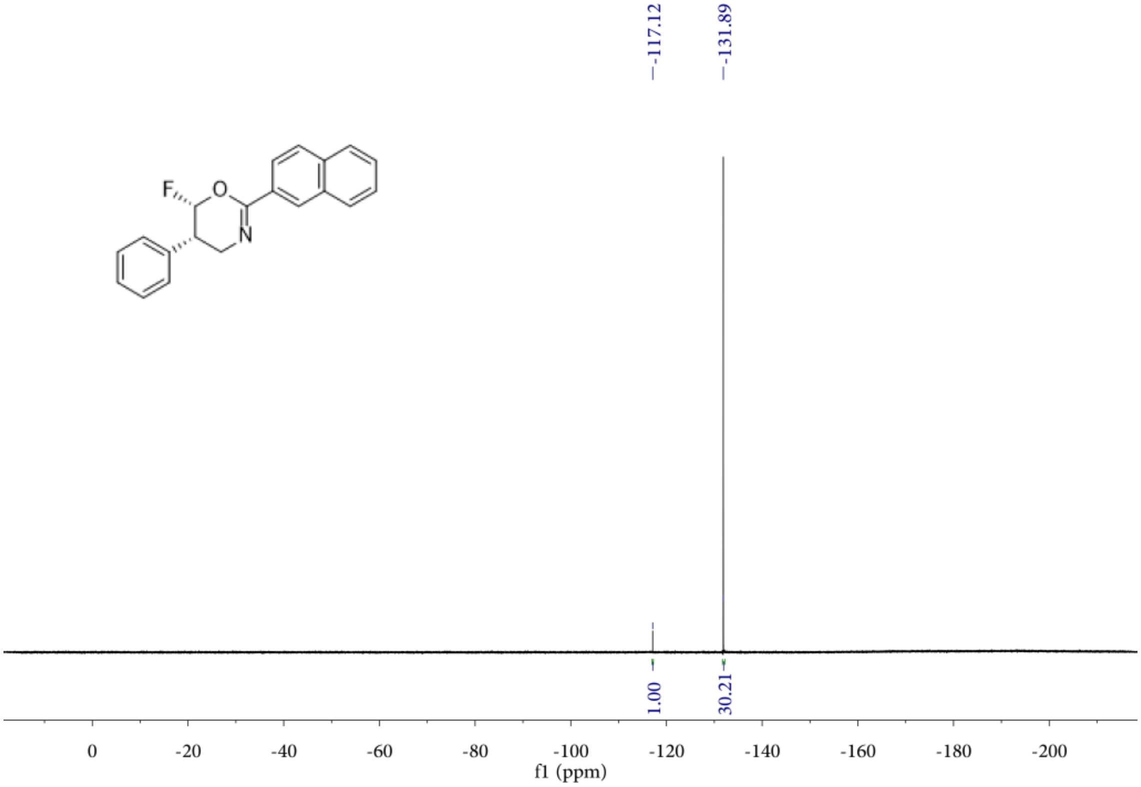

Supplementary Figure 132. ^{1}H NMR spectrum of 27b

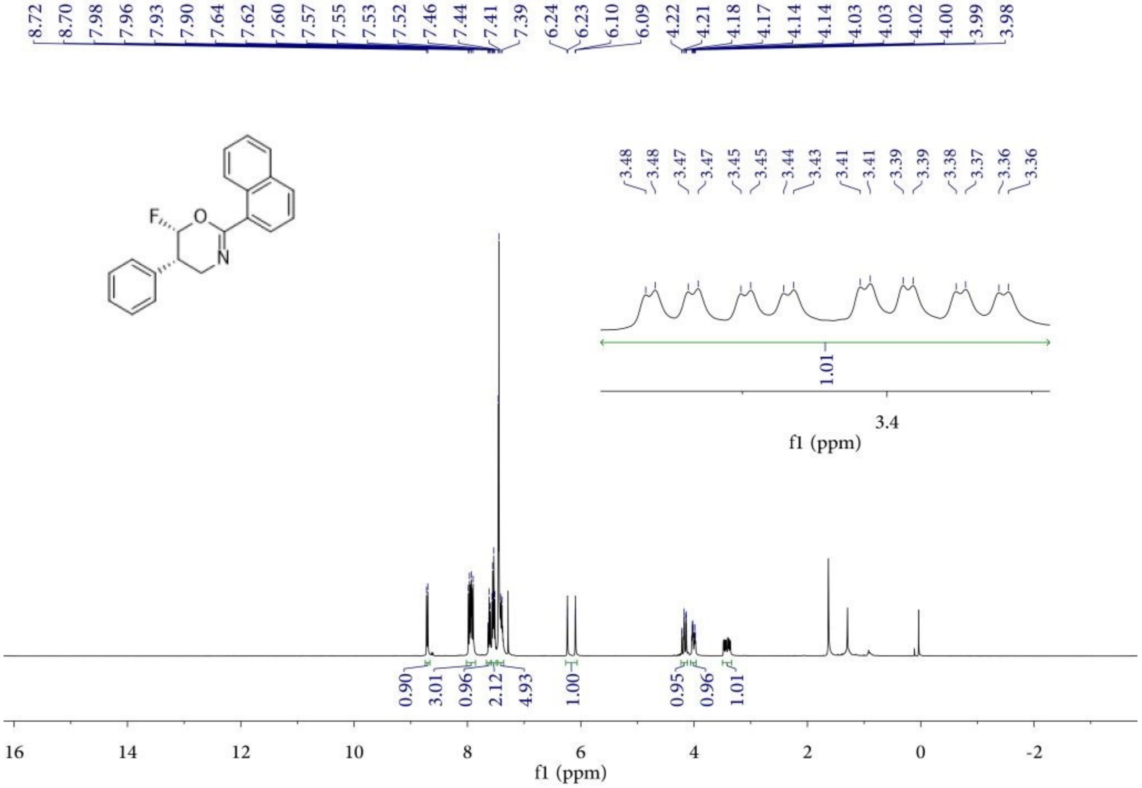

Supplementary Figure 133. ^{13}C NMR spectrum of 27b

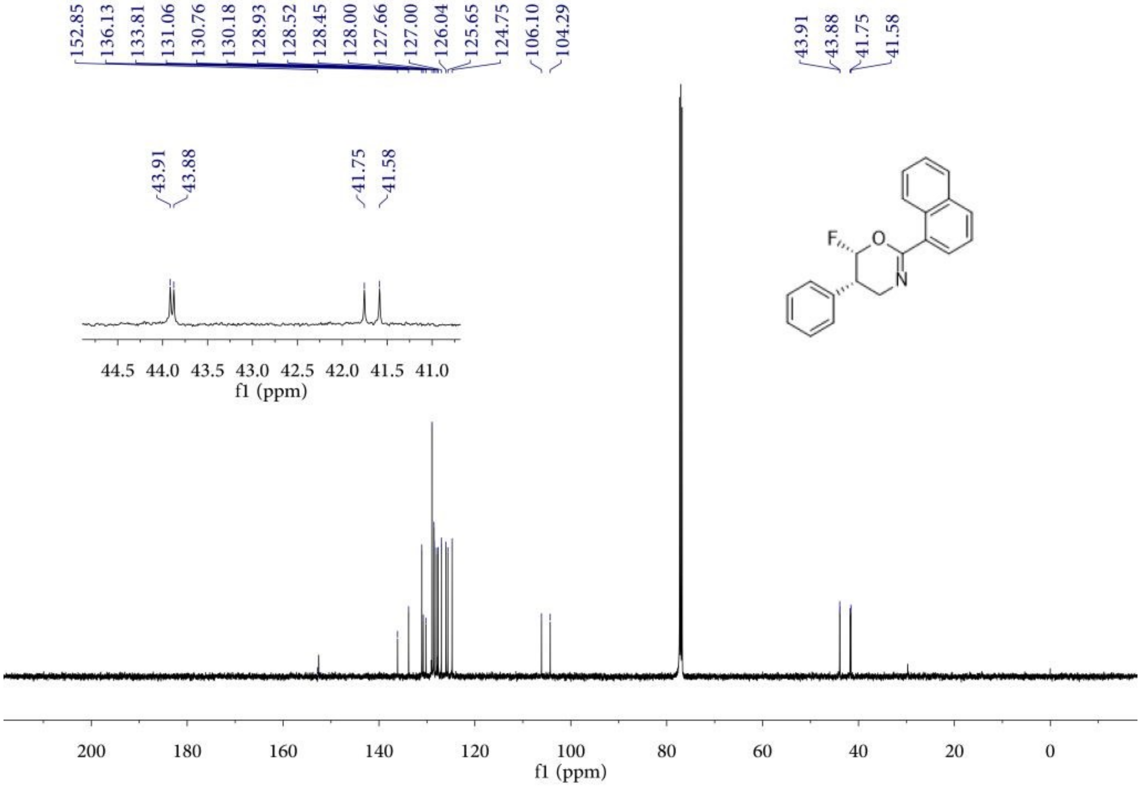

Supplementary Figure 134. ^{19}F NMR spectrum of 27b

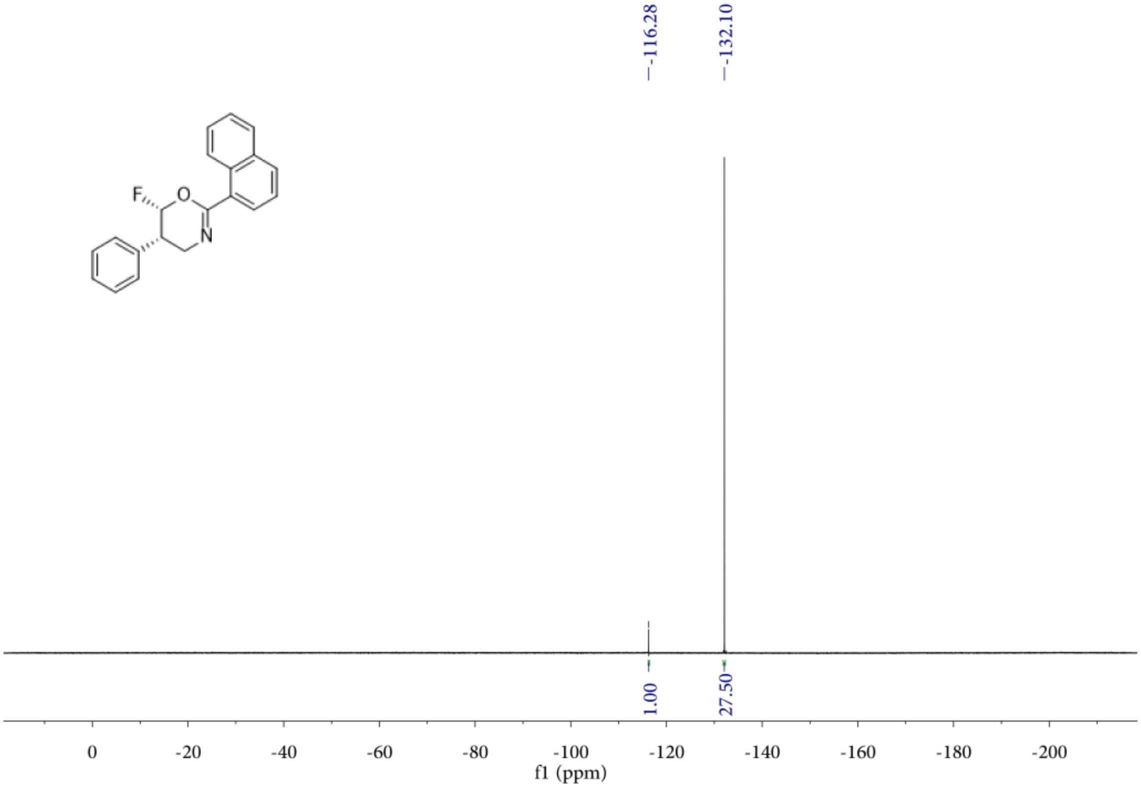

Supplementary Figure 135. ^{1}H NMR spectrum of 28b

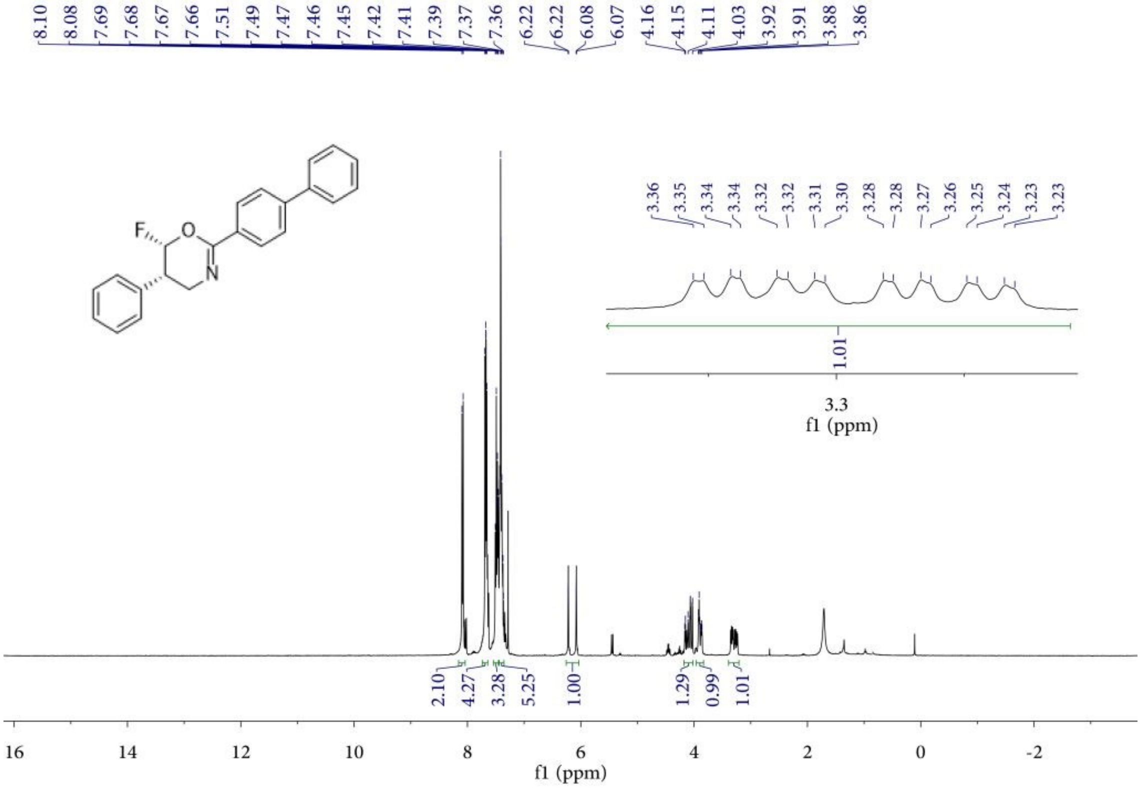

Supplementary Figure 136. ^{13}C NMR spectrum of 28b

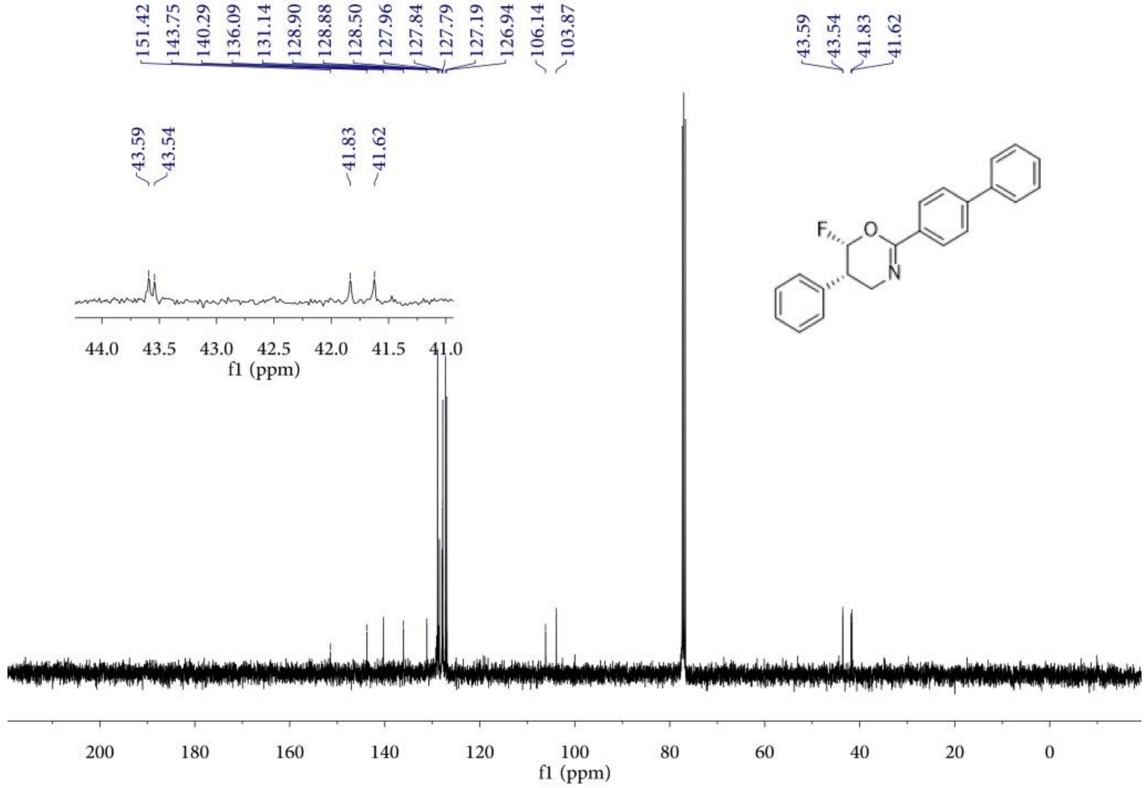

Supplementary Figure 137. ^{19}F NMR spectrum of 28b

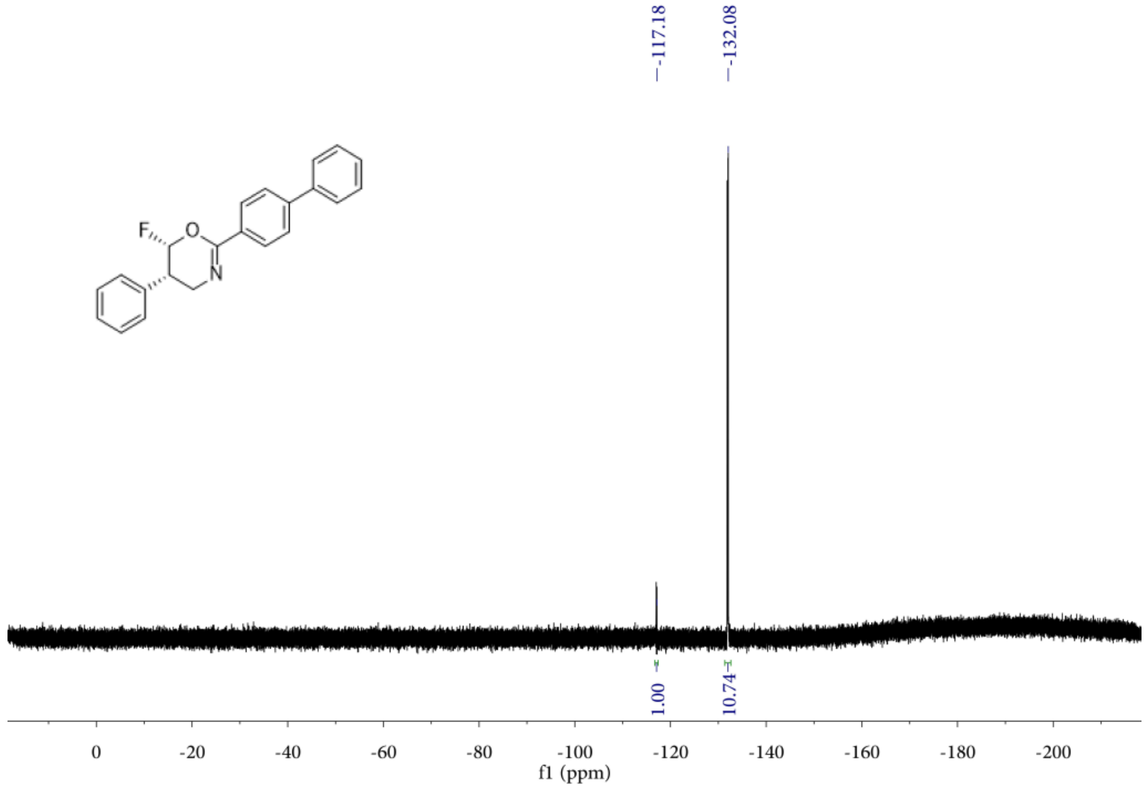

Supplementary Figure 138. ^{1}H NMR spectrum of 29b

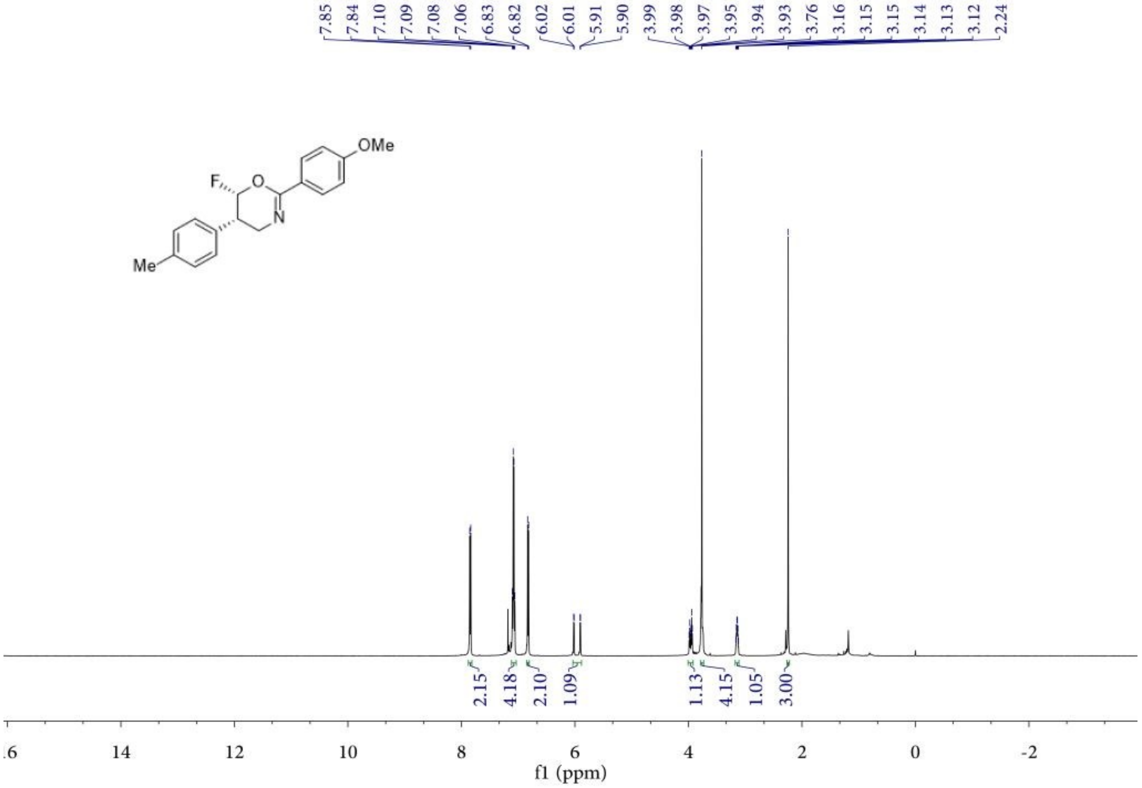

Supplementary Figure 139. ^{13}C NMR spectrum of 29b

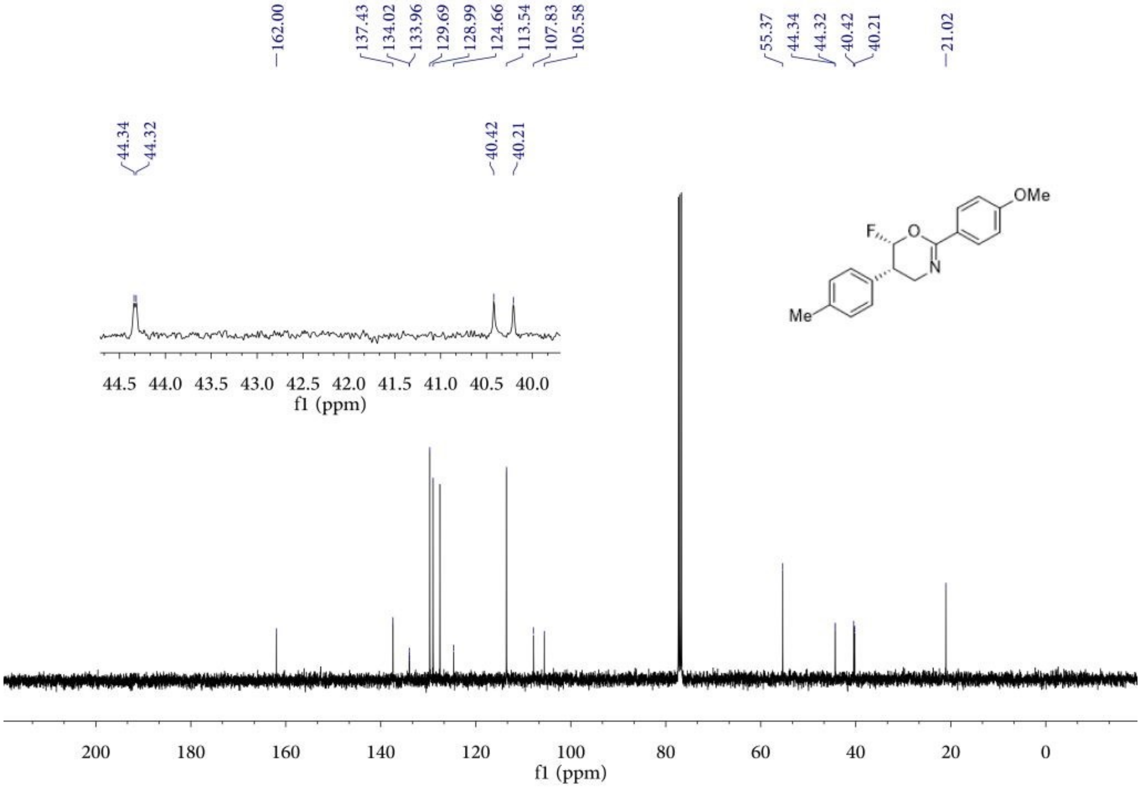

Supplementary Figure 140. ^{19}F NMR spectrum of 29b

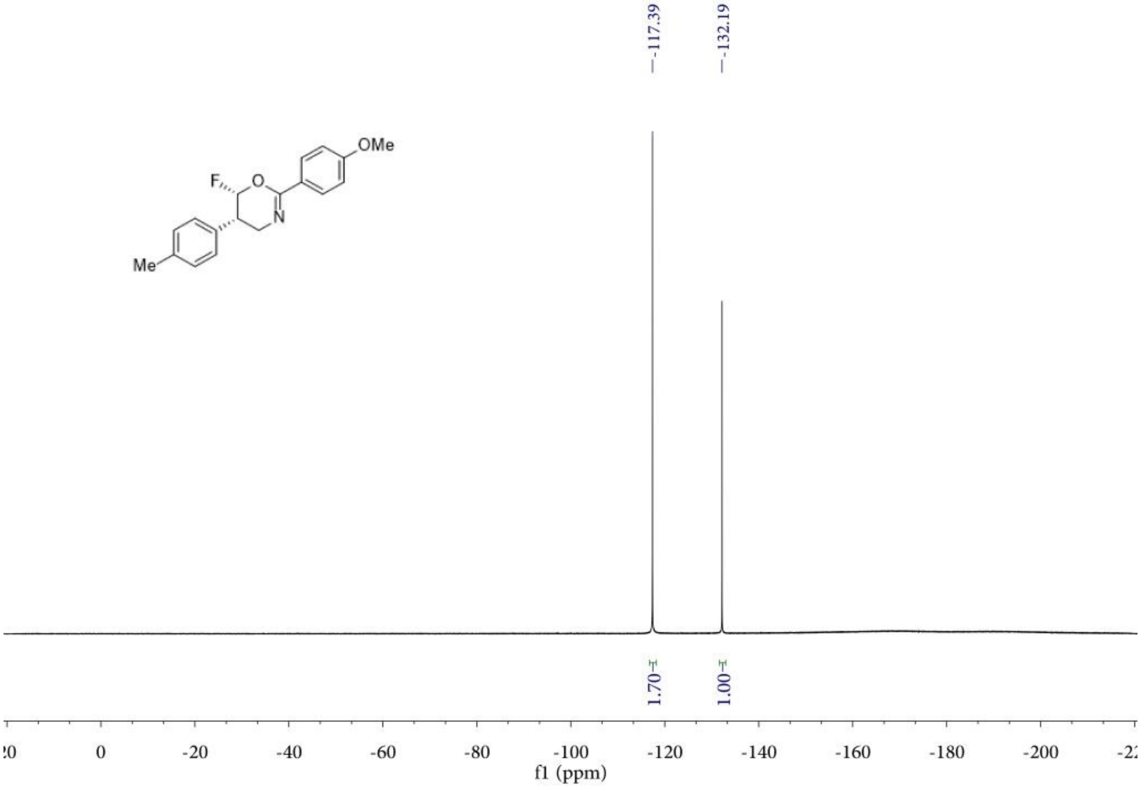

Supplementary Figure 141. ^{1}H NMR spectrum of 30b

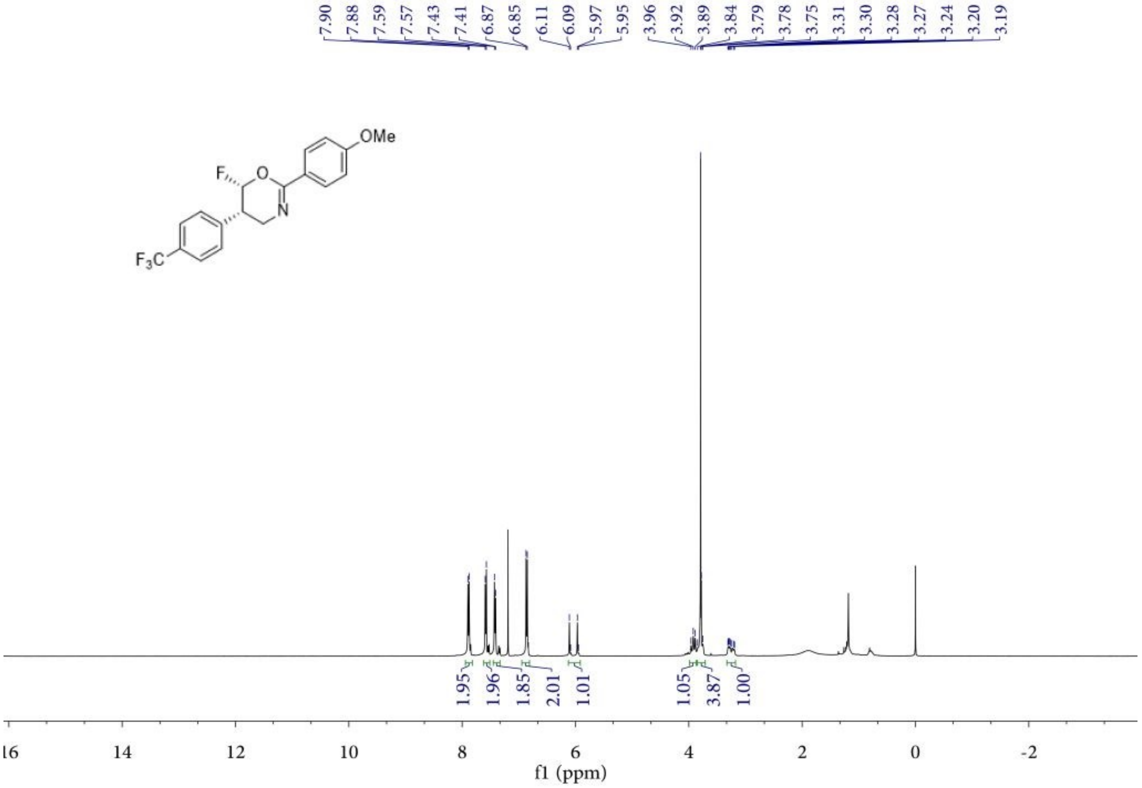

Supplementary Figure 142. ^{13}C NMR spectrum of 30b

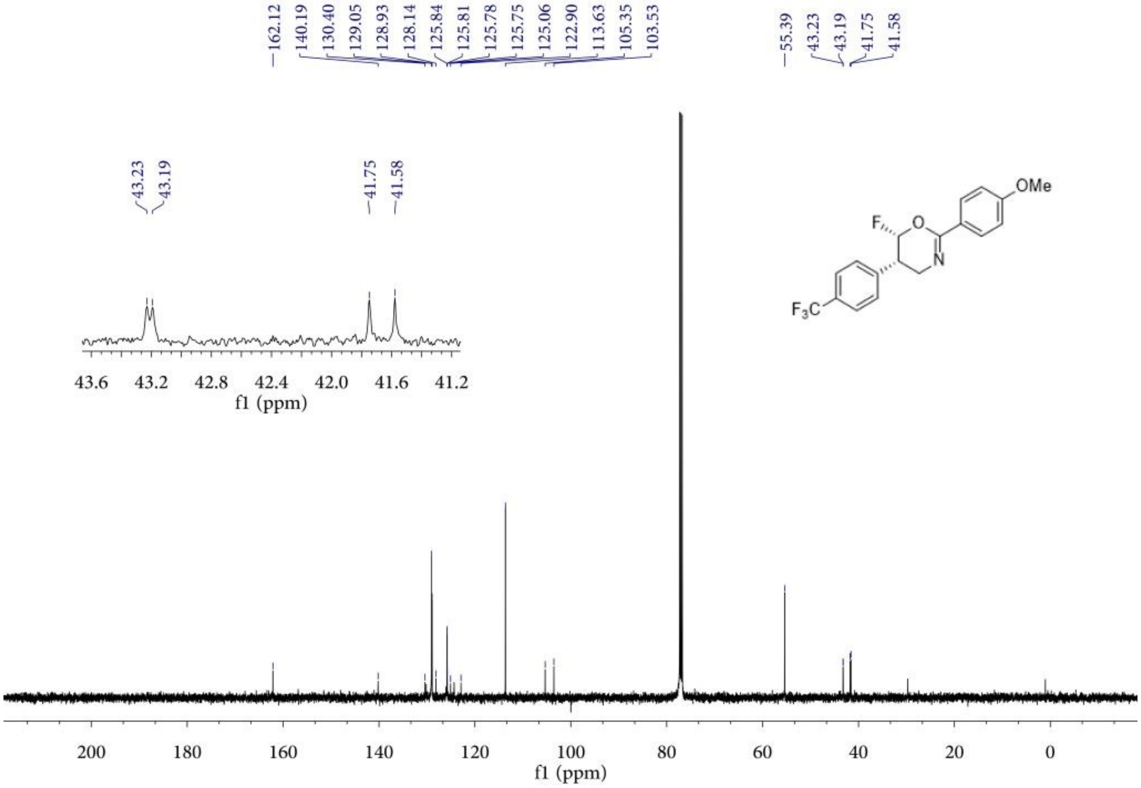

Supplementary Figure 143. ^{19}F NMR spectrum of 30b

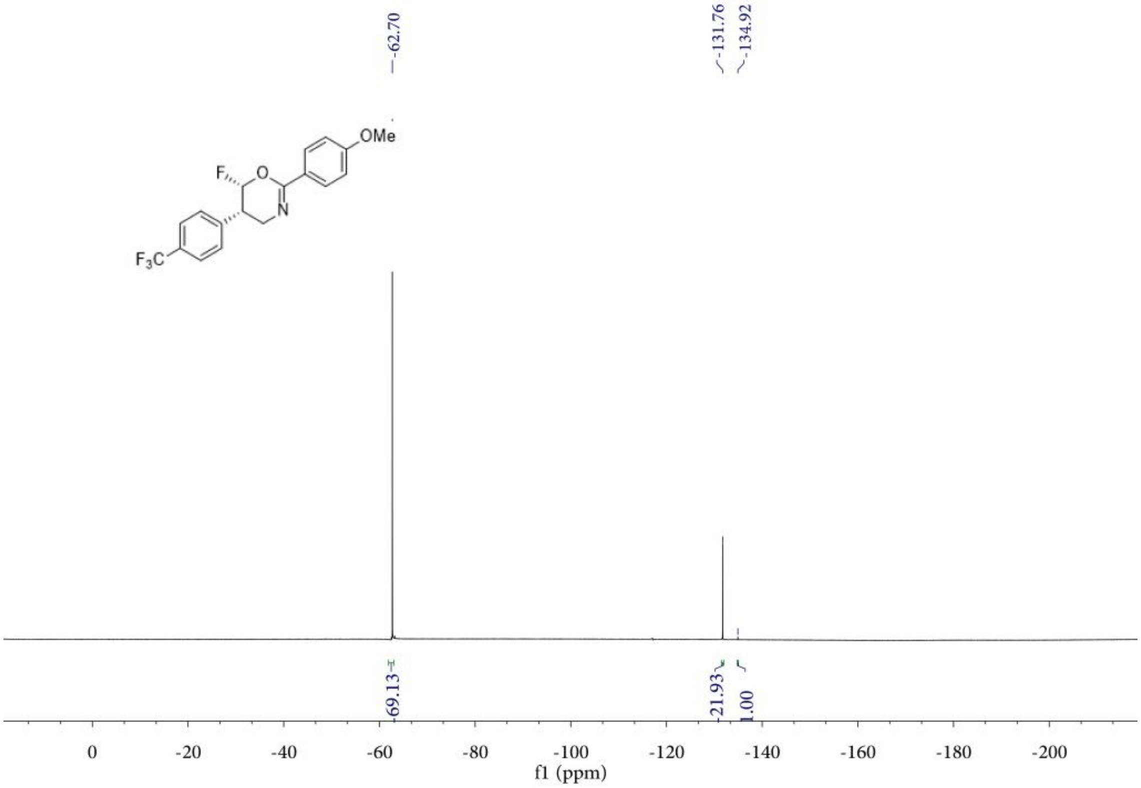

Supplementary Figure 144. ^{1}H NMR spectrum of 31b

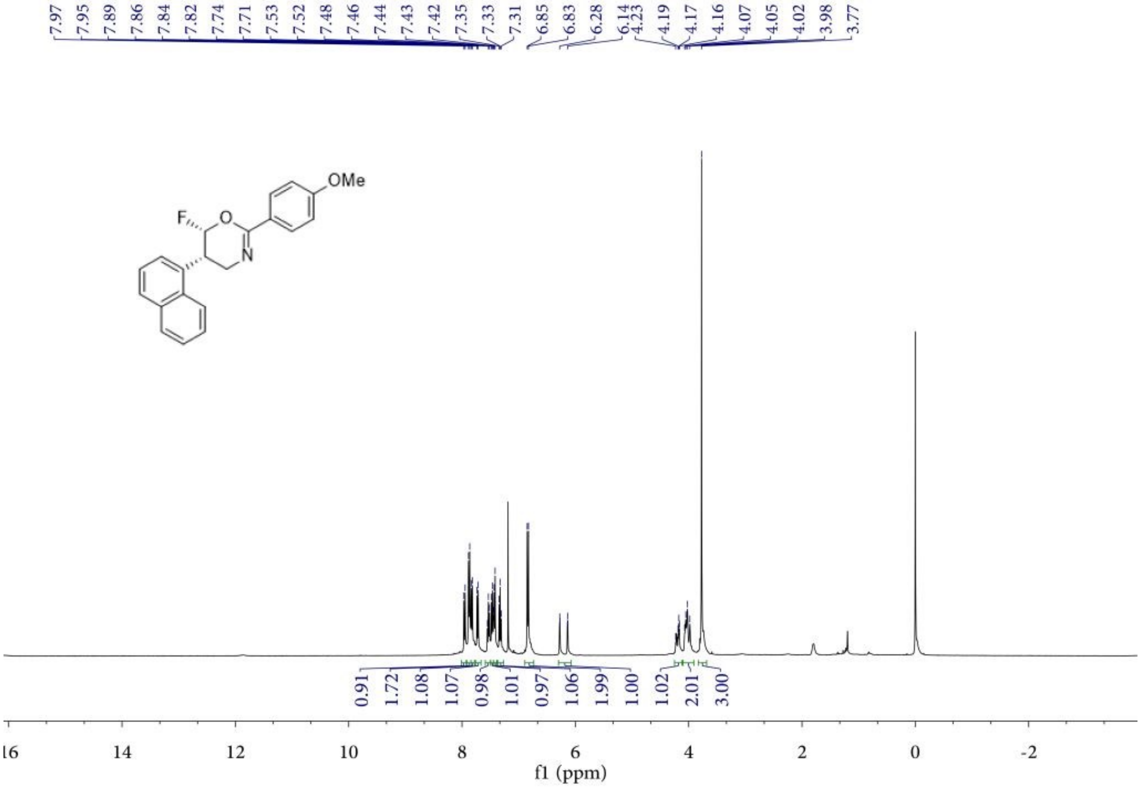

Supplementary Figure 145. ^{13}C NMR spectrum of 31b

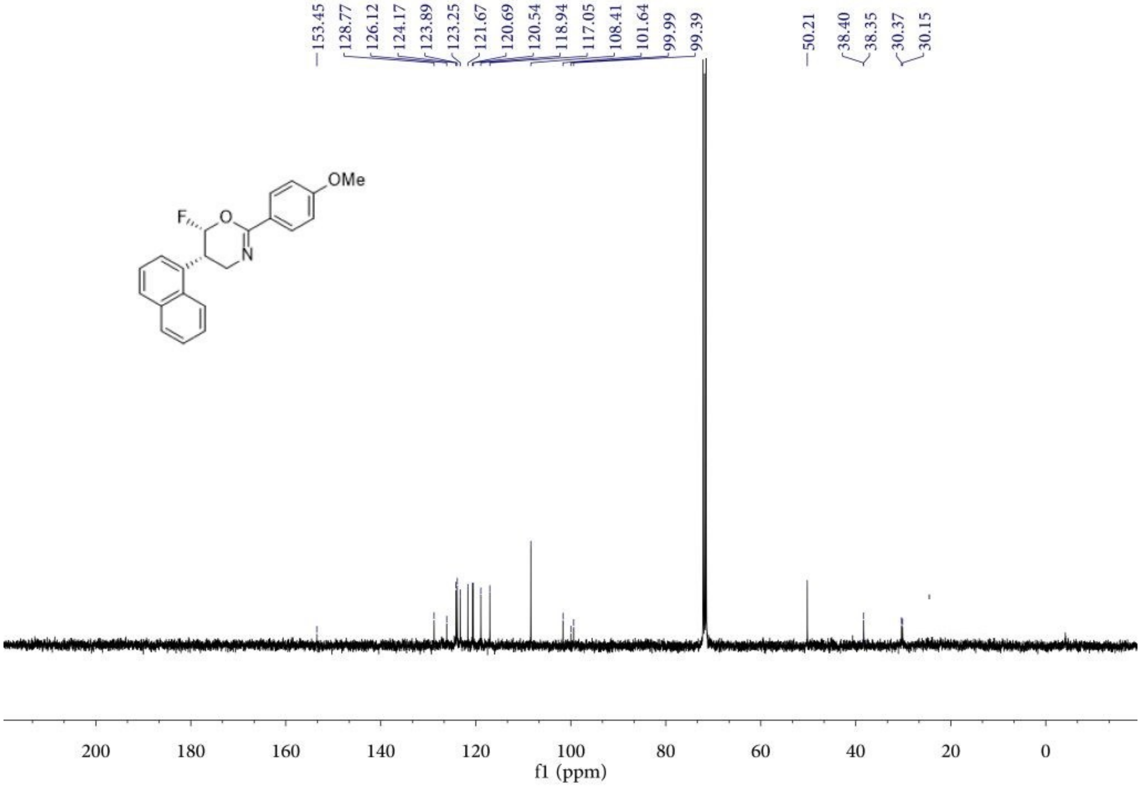

Supplementary Figure 146. ^{19}F NMR spectrum of 32b

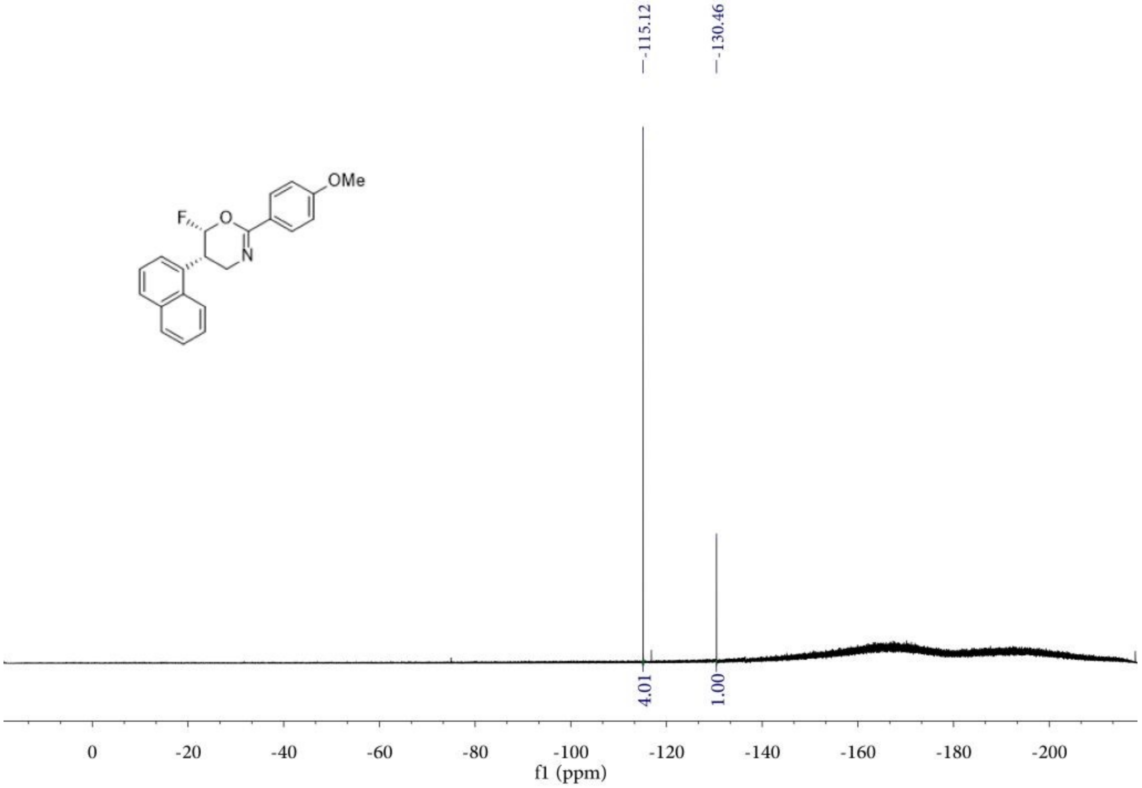

Supplementary Figure 147. ^{1}H NMR spectrum of 32b

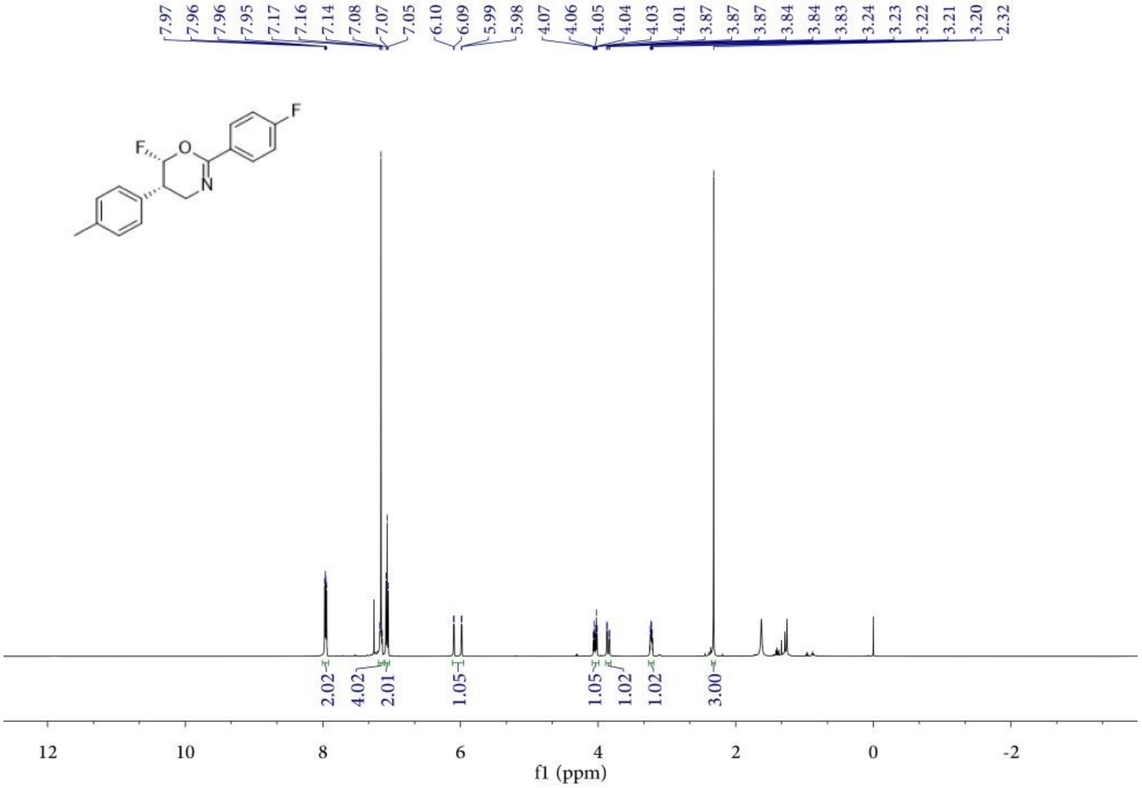

Supplementary Figure 148. ^{13}C NMR spectrum of 32b

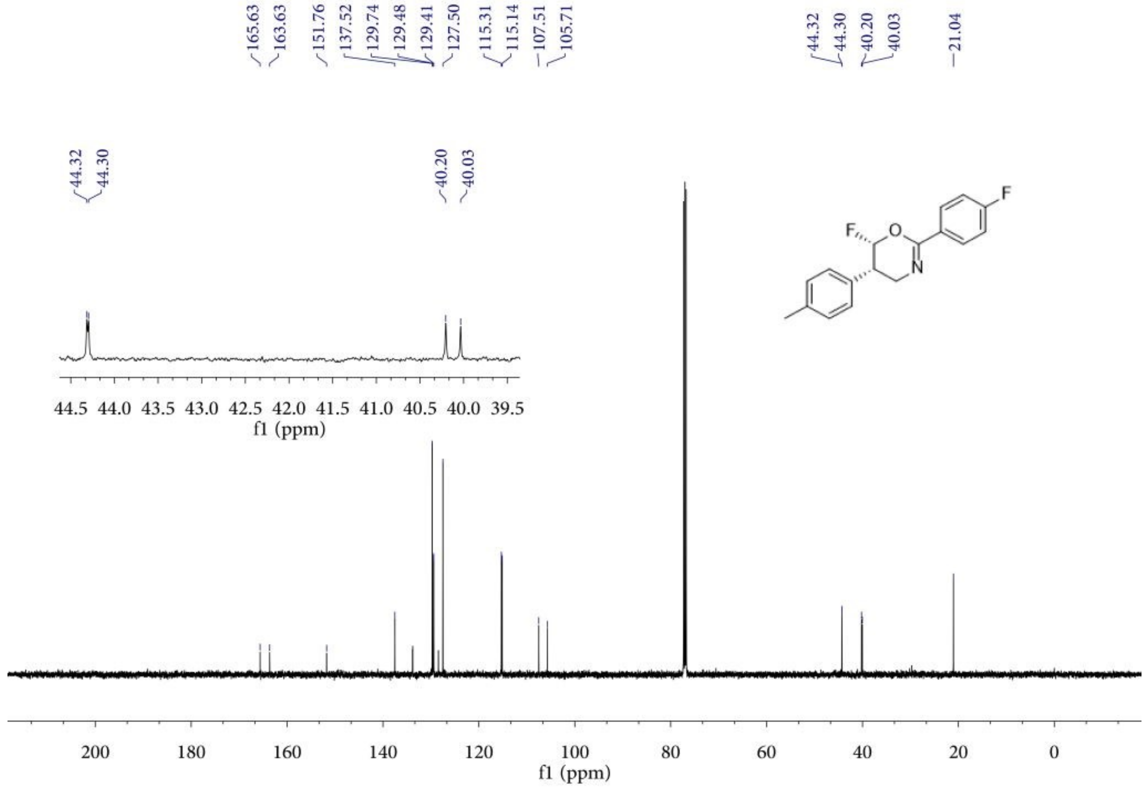

Supplementary Figure 149. ^{19}F NMR spectrum of 32b

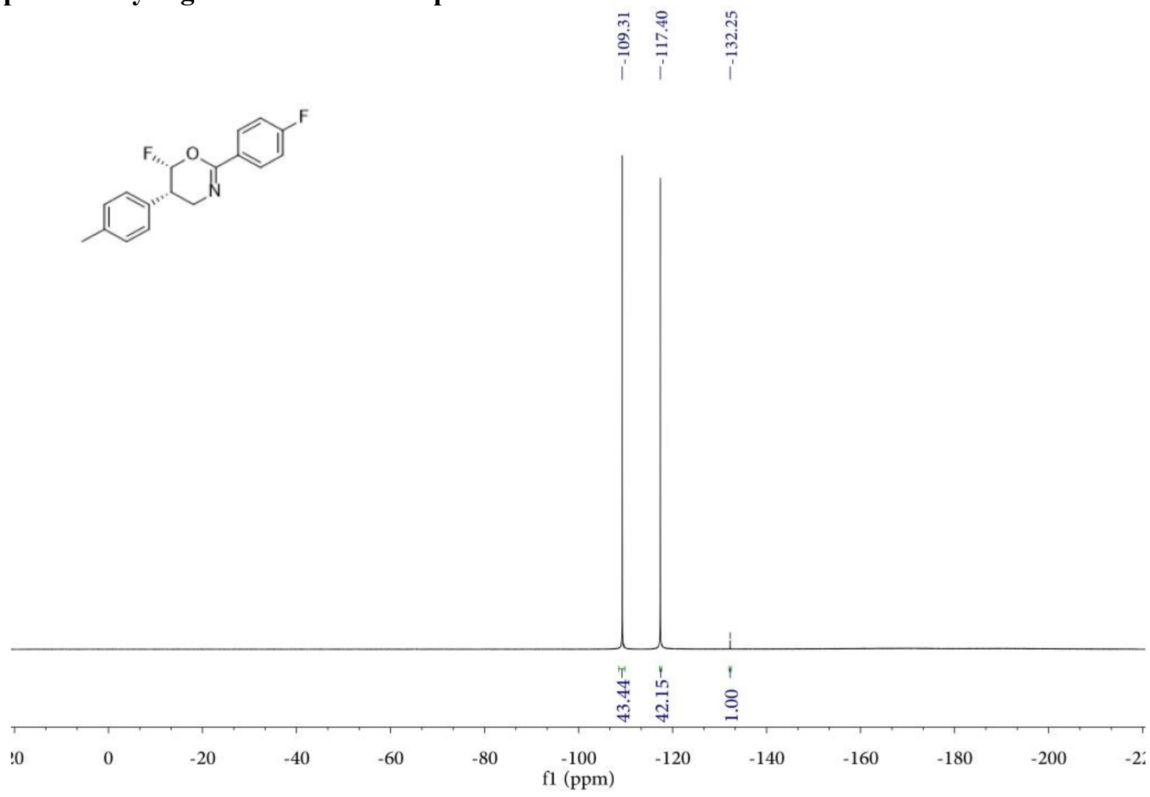

Supplementary Figure 150. ^{1}**H NMR spectrum of 33b**

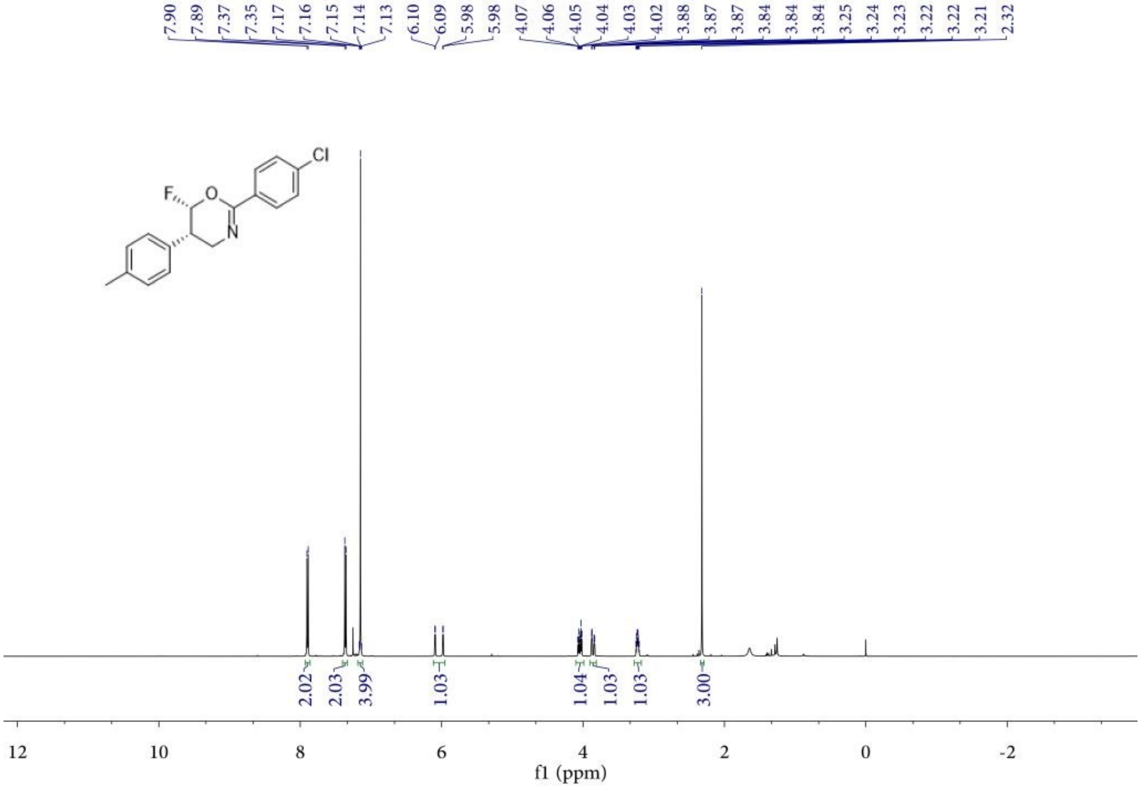

Supplementary Figure 151. ^{13}**C NMR spectrum of 33b**

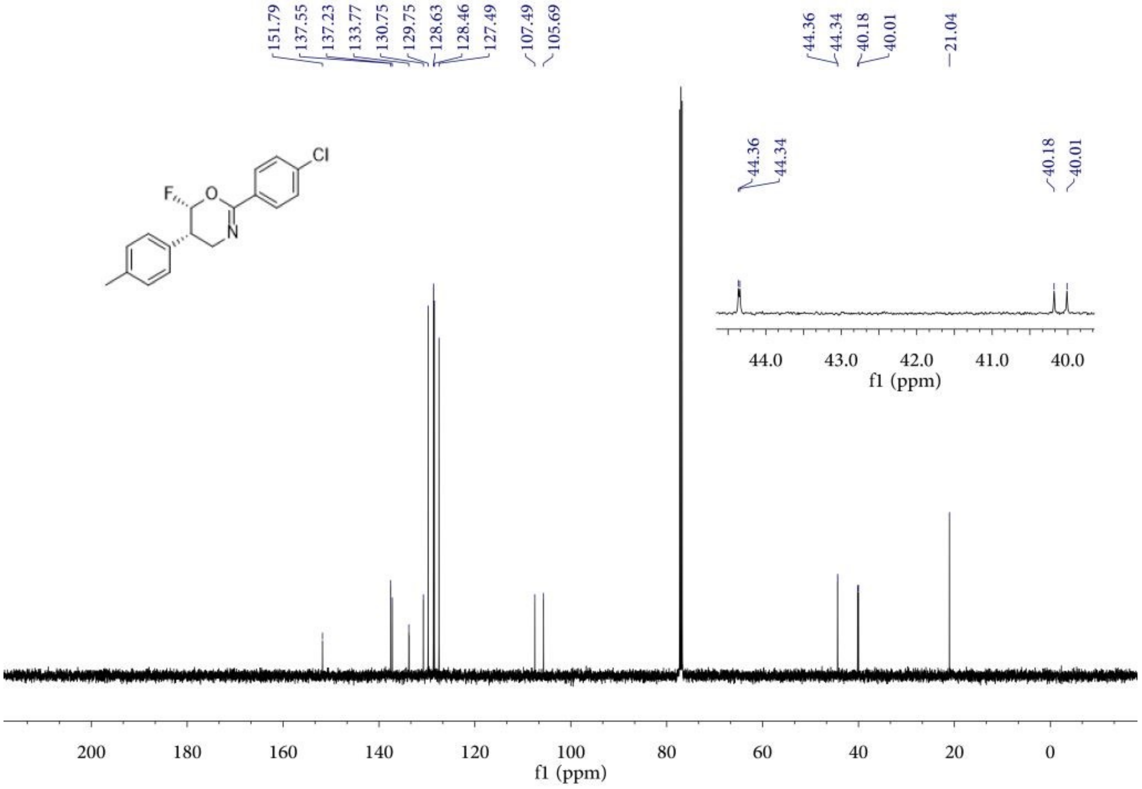

Supplementary Figure 152. ^{19}F NMR spectrum of 33b

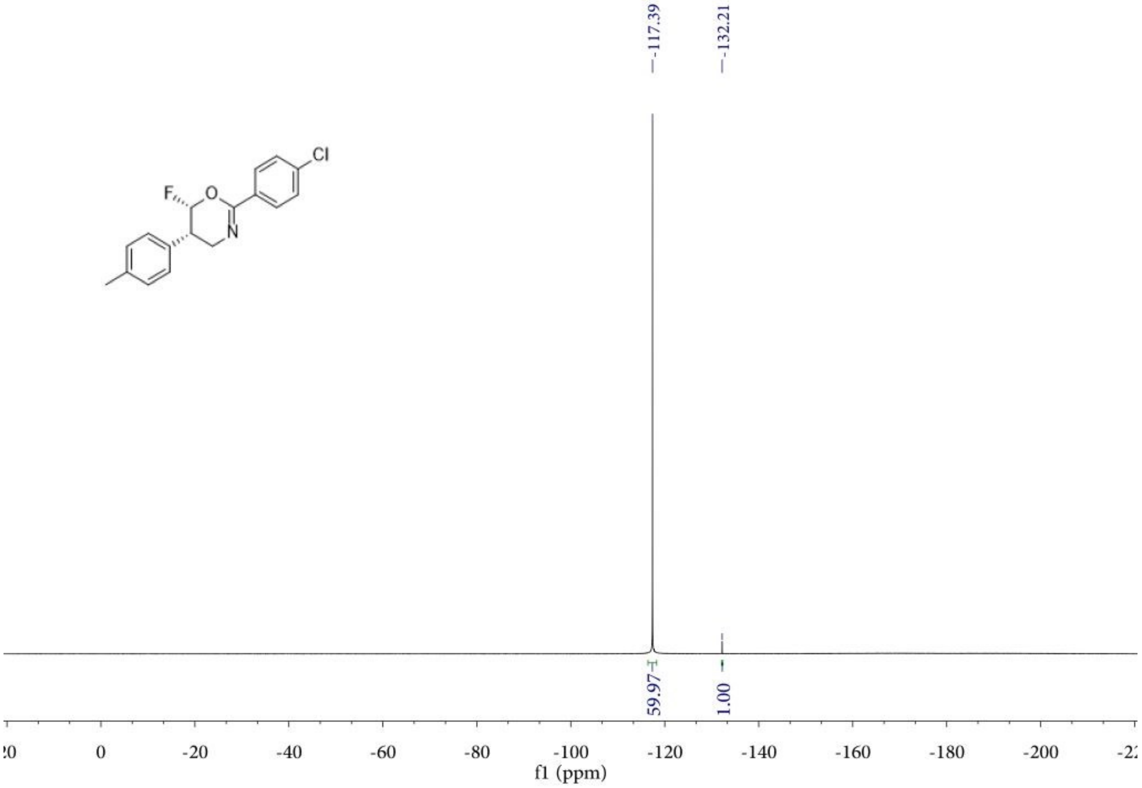

Supplementary Figure 153. ^{1}H NMR spectrum of 34b

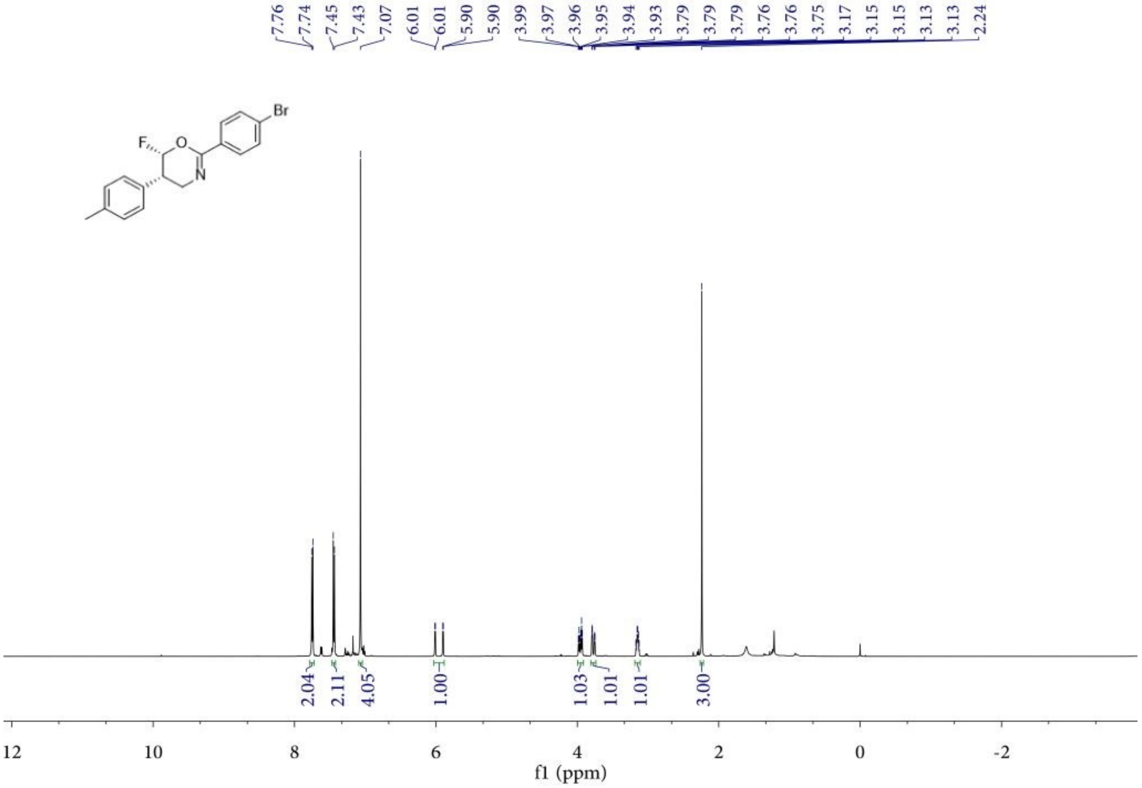

Supplementary Figure 154. ^{13}C NMR spectrum of 34b

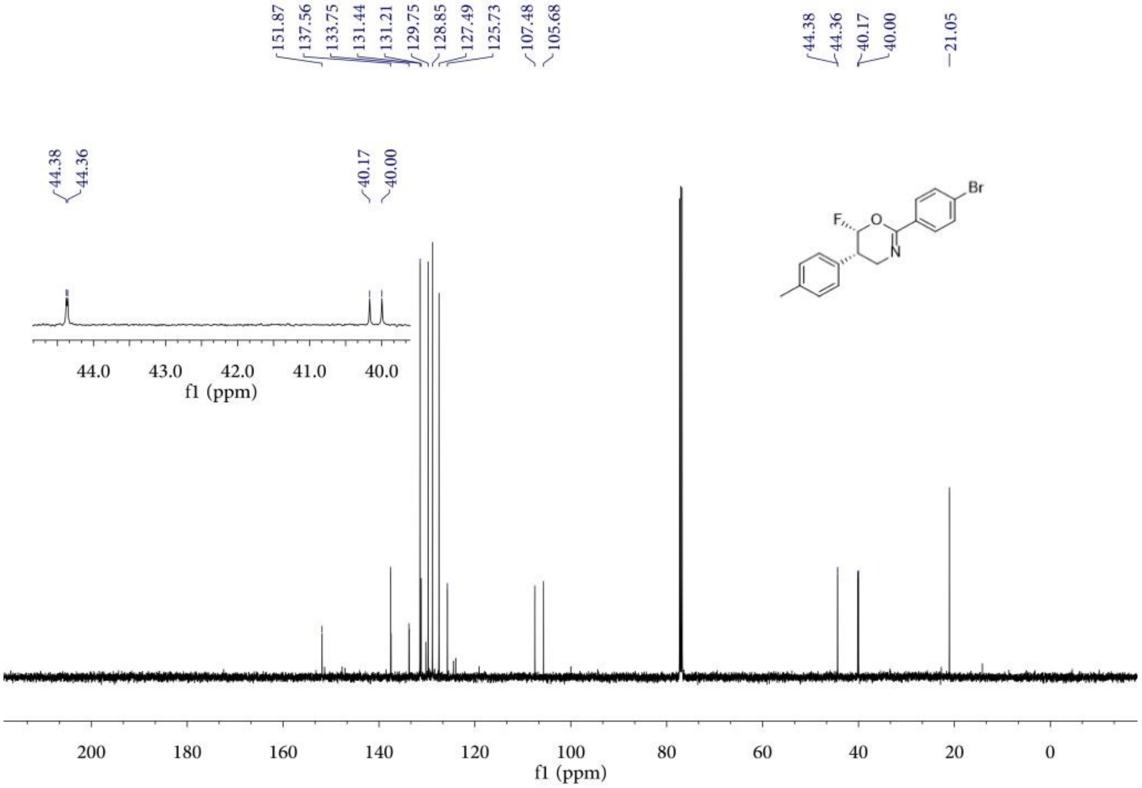

Supplementary Figure 155. ^{19}F NMR spectrum of 34b

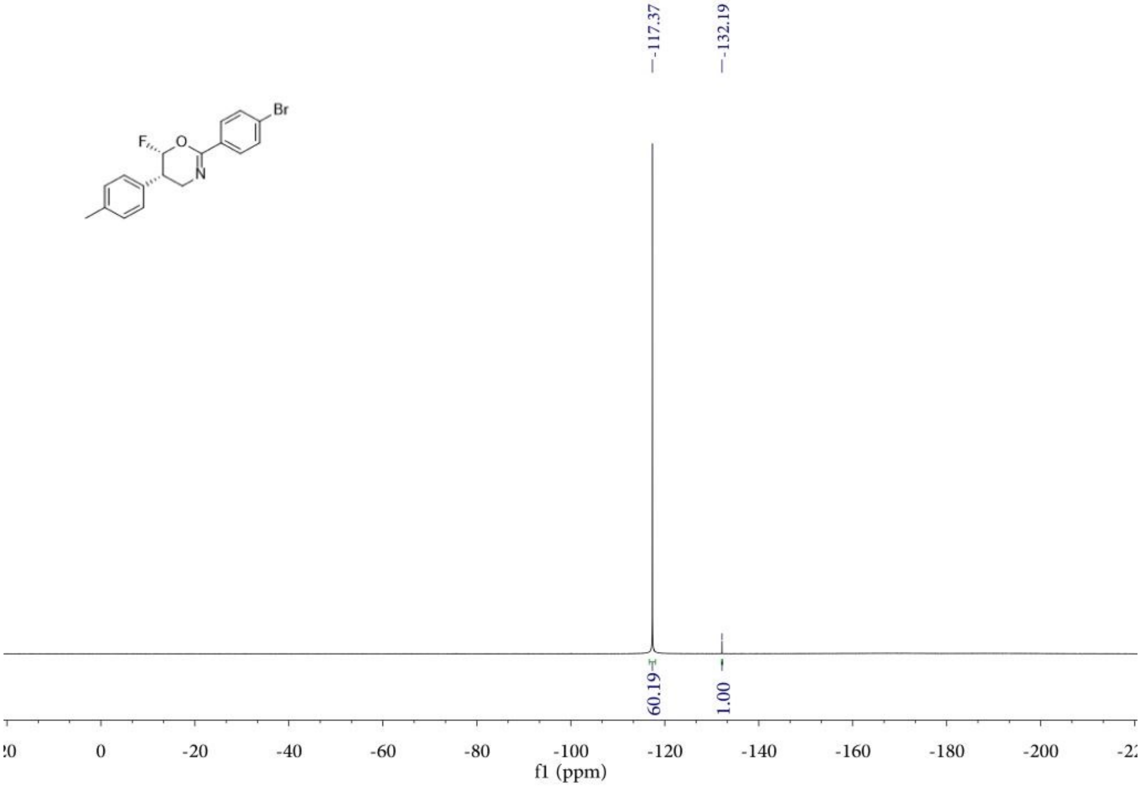

Supplementary Figure 156. ^{1}H NMR spectrum of 35b

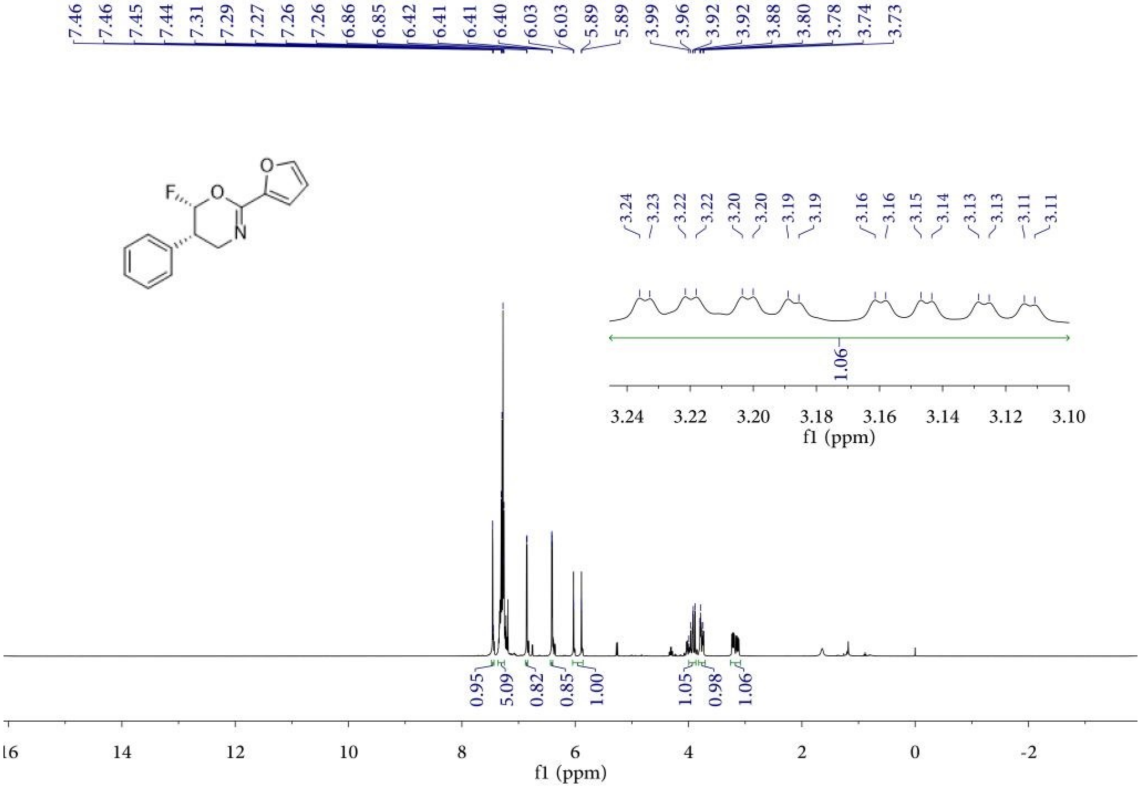

Supplementary Figure 157. ^{13}C NMR spectrum of 35b

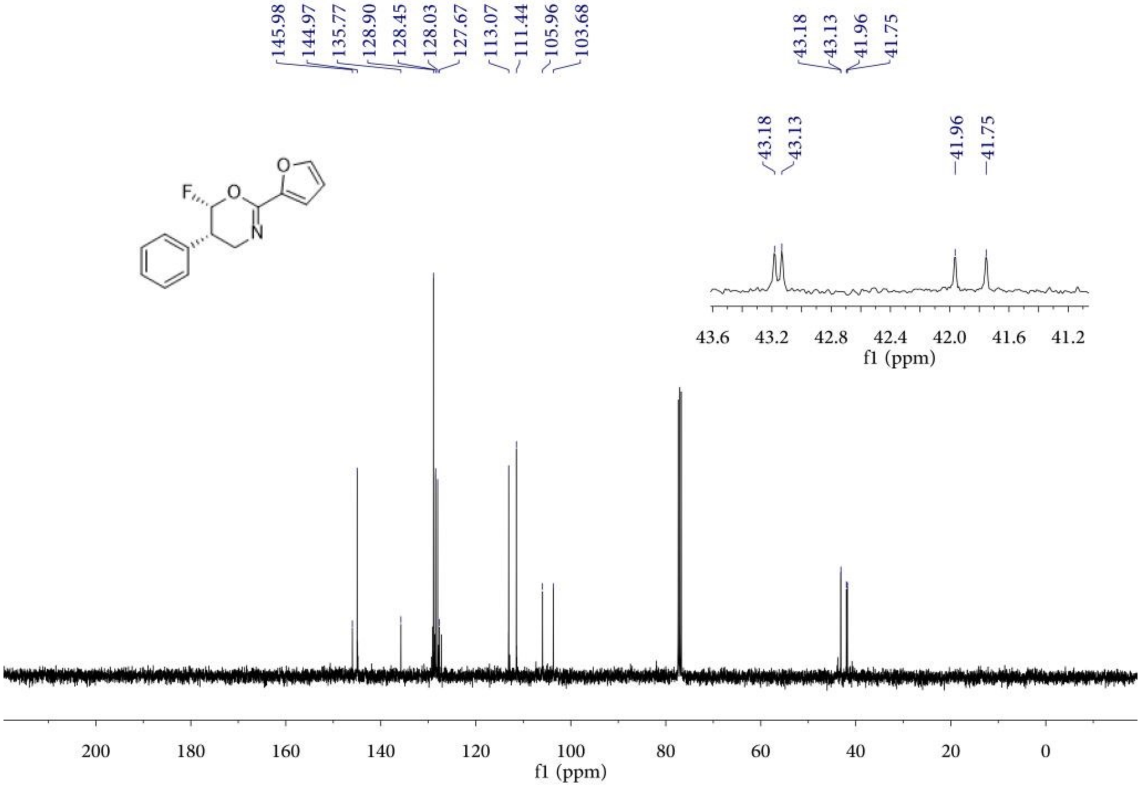

Supplementary Figure 158. ^{19}F **NMR spectrum of 35b**

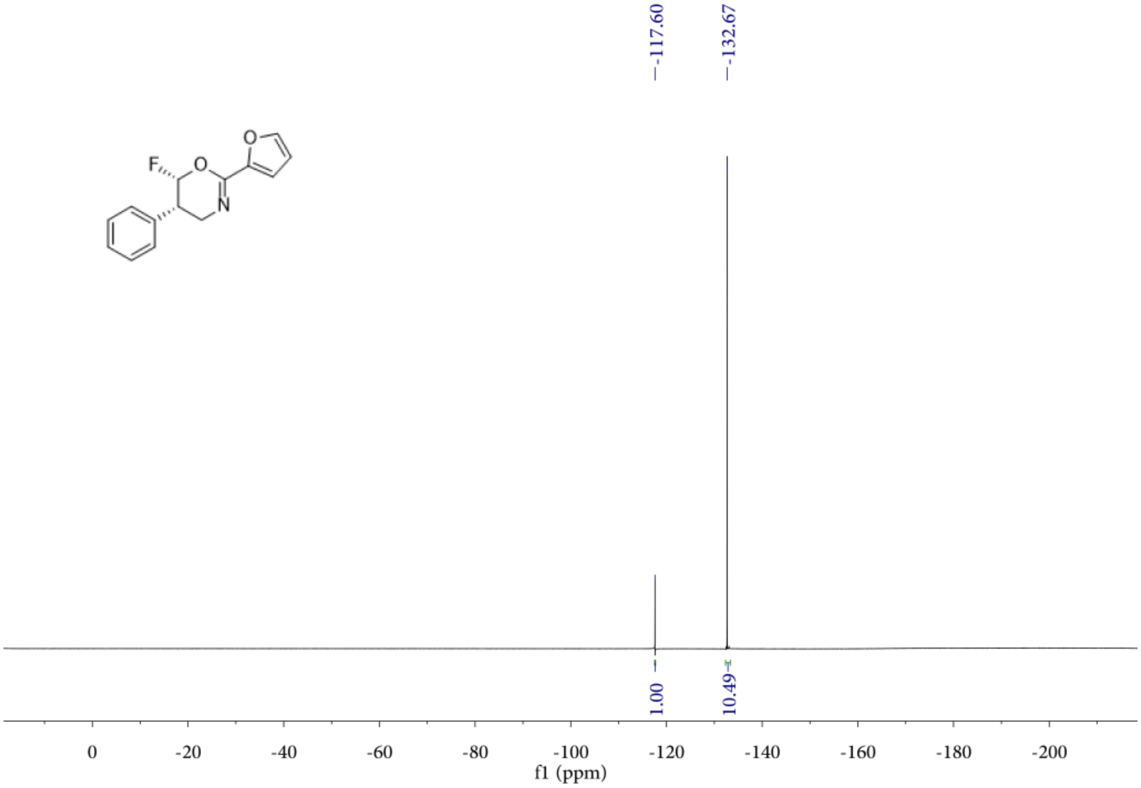

Supplementary Figure 159. ^{1}H **NMR spectrum of 36b**

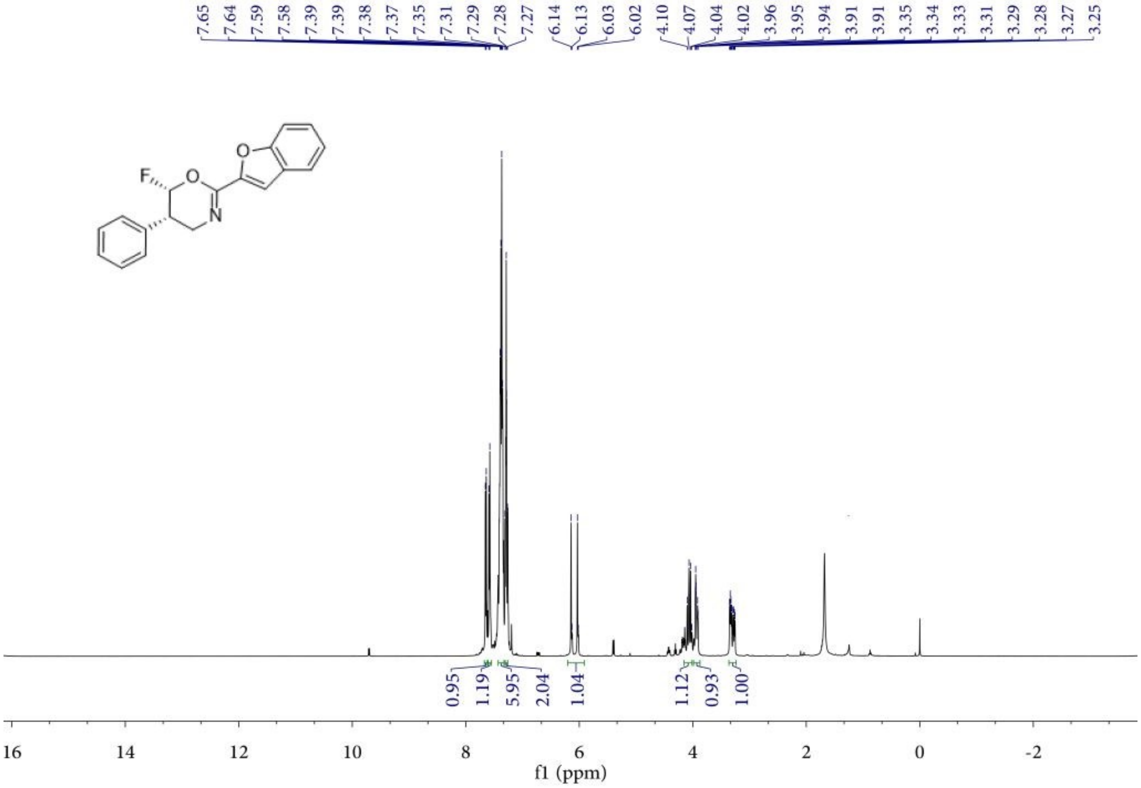

Supplementary Figure 160. ^{13}C NMR spectrum of 36b

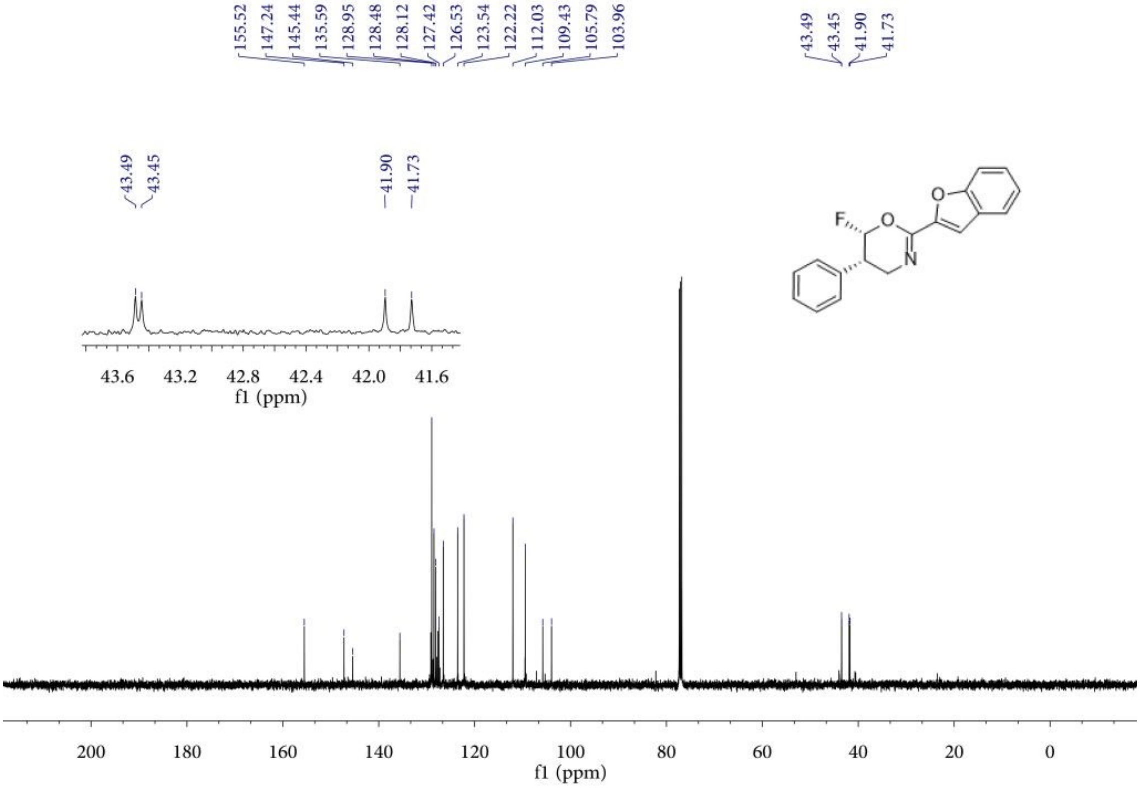

Supplementary Figure 161. ^{19}F NMR spectrum of 36b

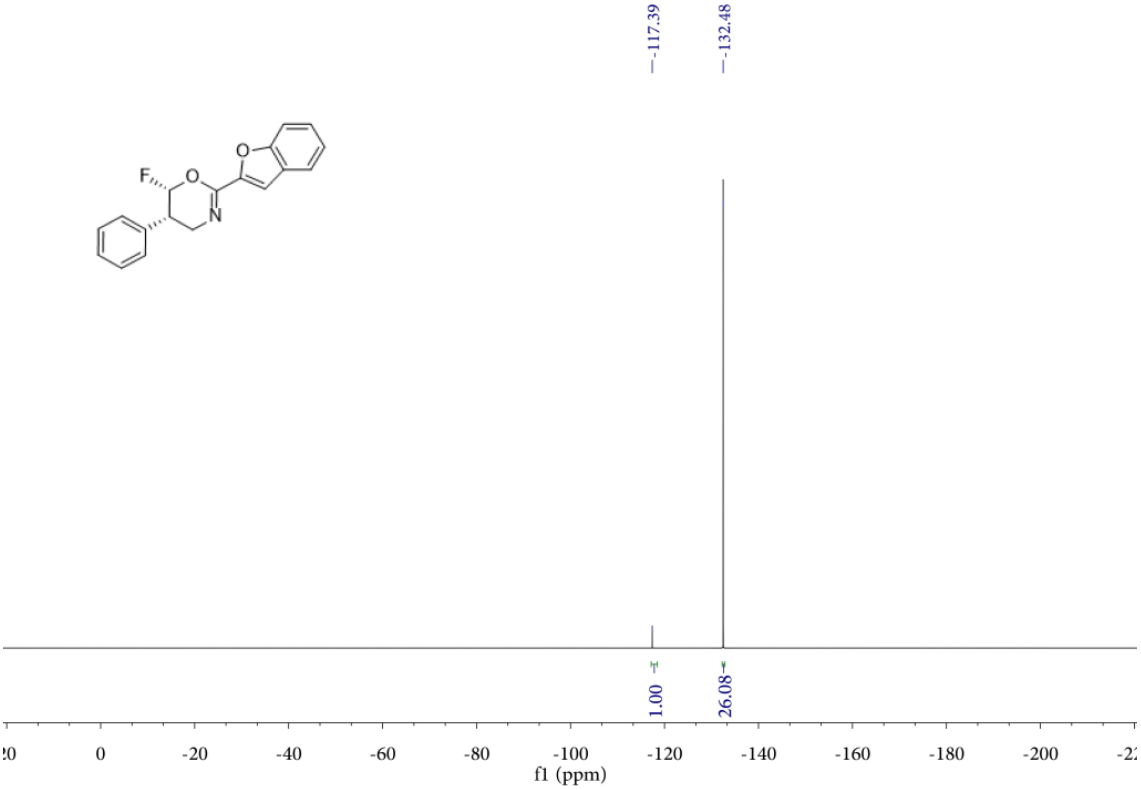

Supplementary Figure 162. ^{1}**H NMR spectrum of 37b**

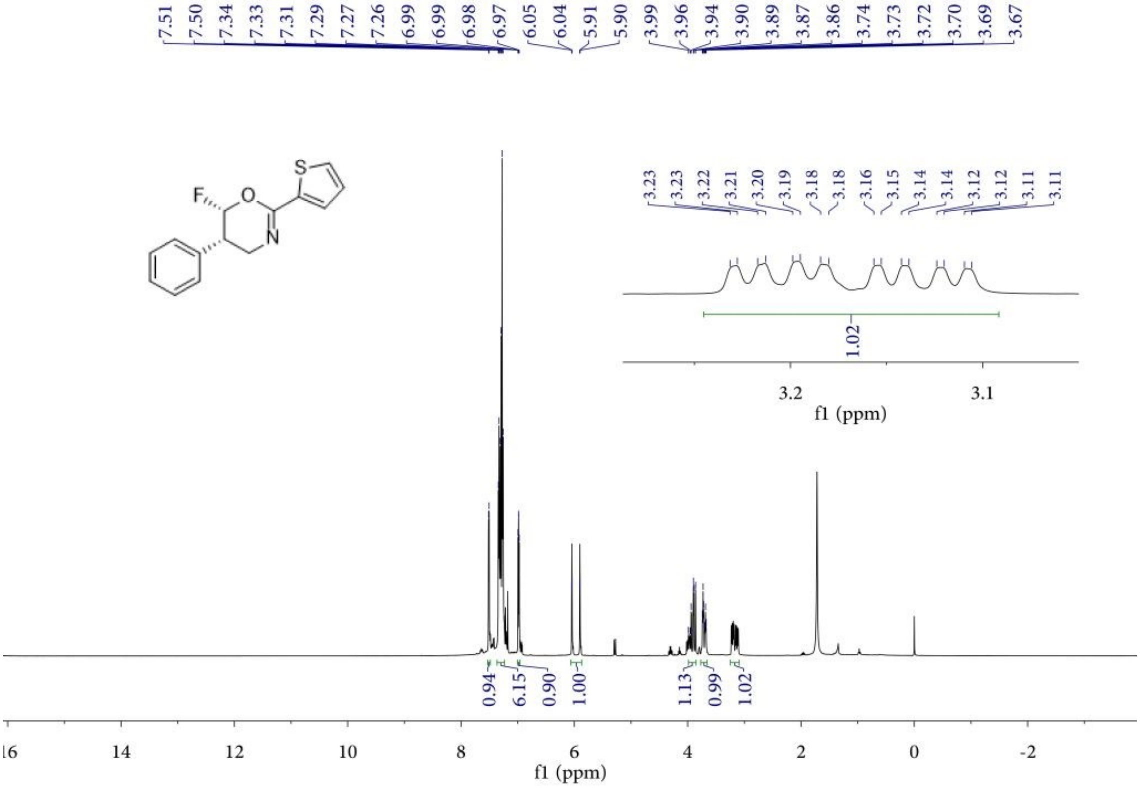

Supplementary Figure 163. ^{13}**C NMR spectrum of 37b**

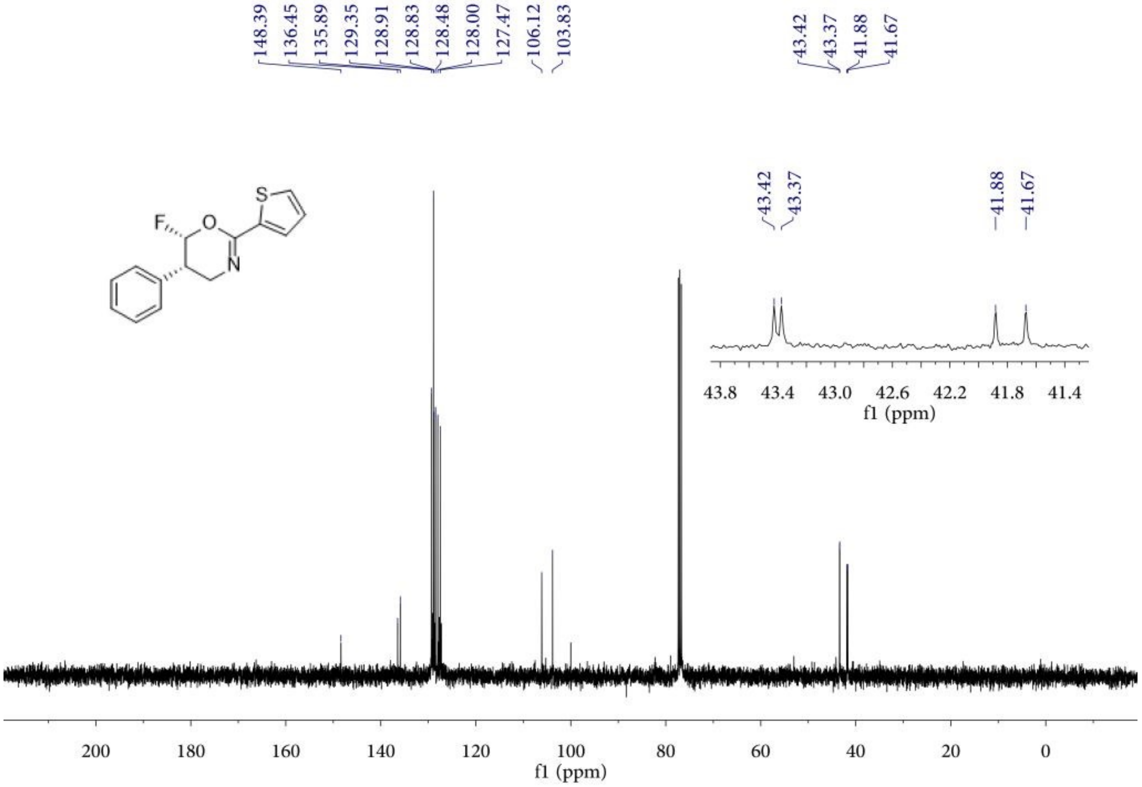

Supplementary Figure 164. ^{19}F NMR spectrum of 37b

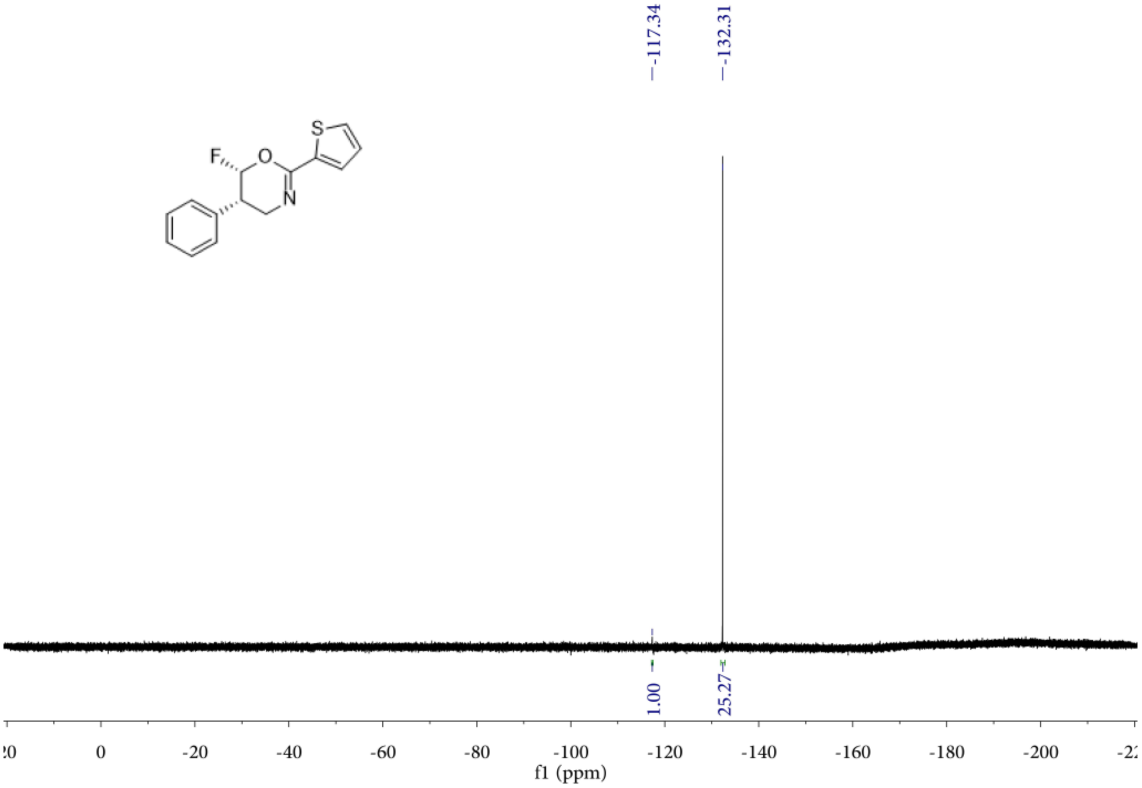

Supplementary Figure 165. ^{1}H NMR spectrum of 38b

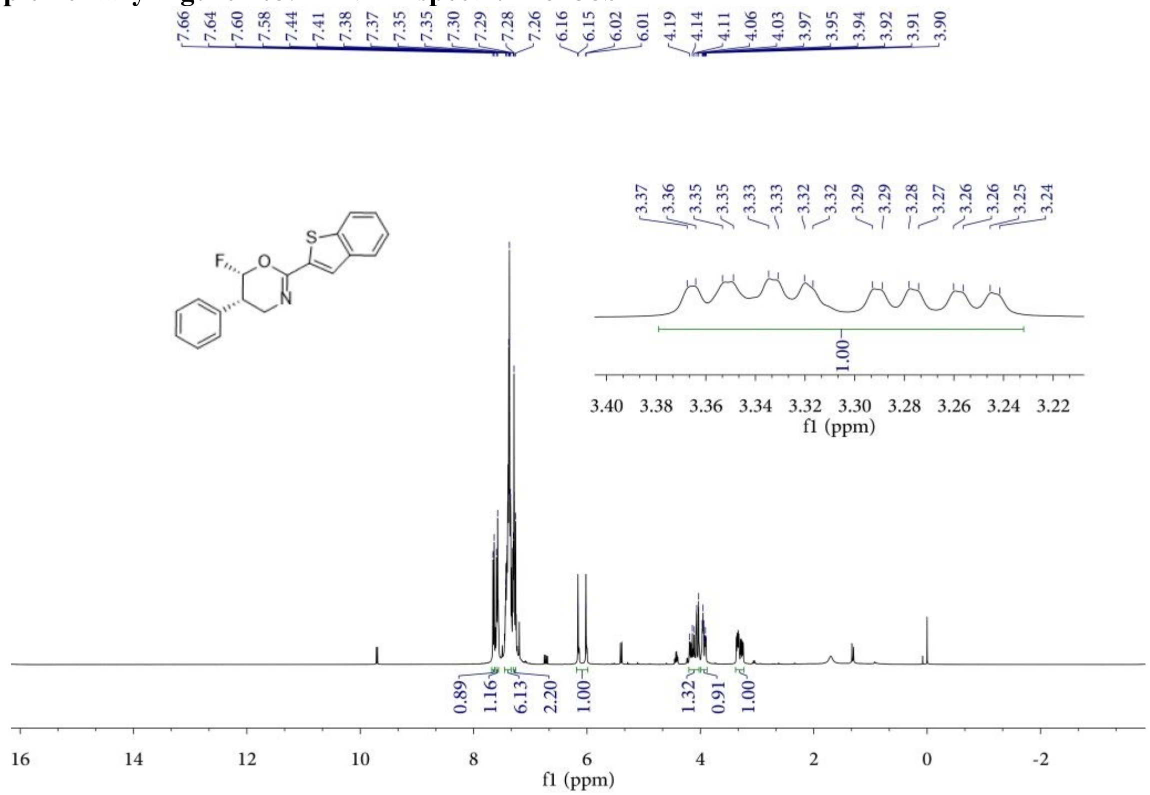

Supplementary Figure 166. ^{13}C NMR spectrum of 38b

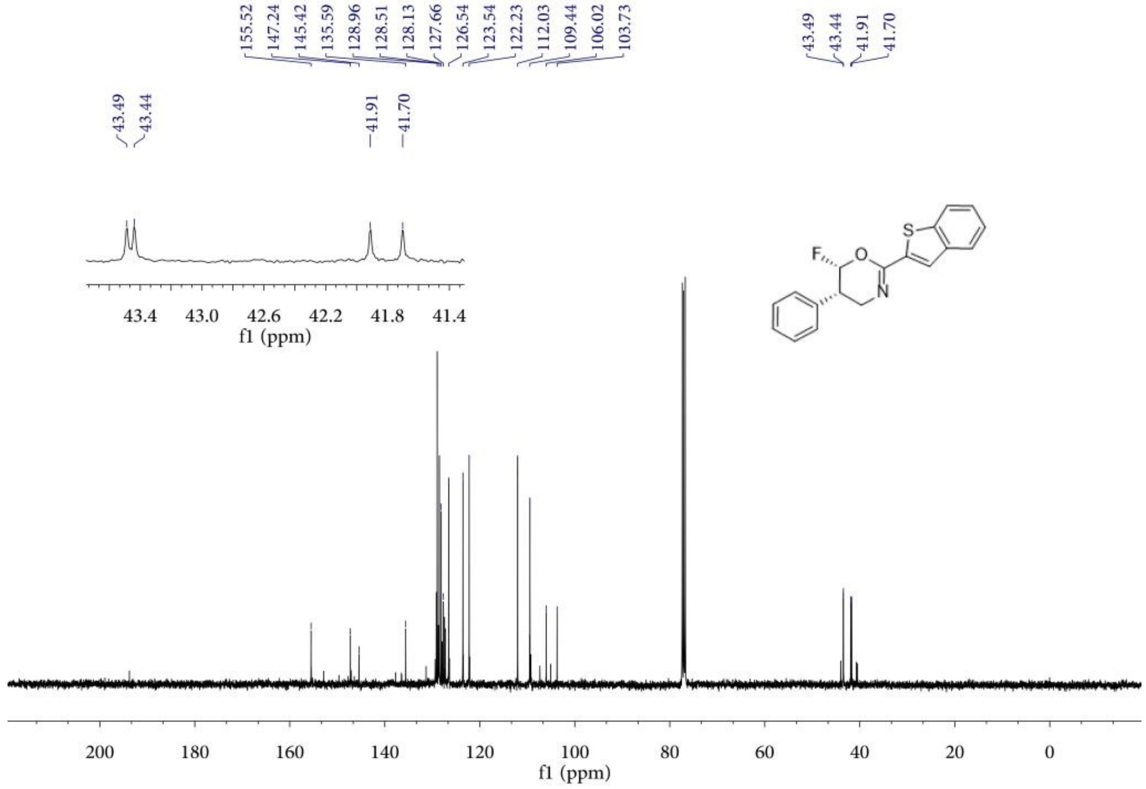

Supplementary Figure 167. ^{19}F NMR spectrum of 38b

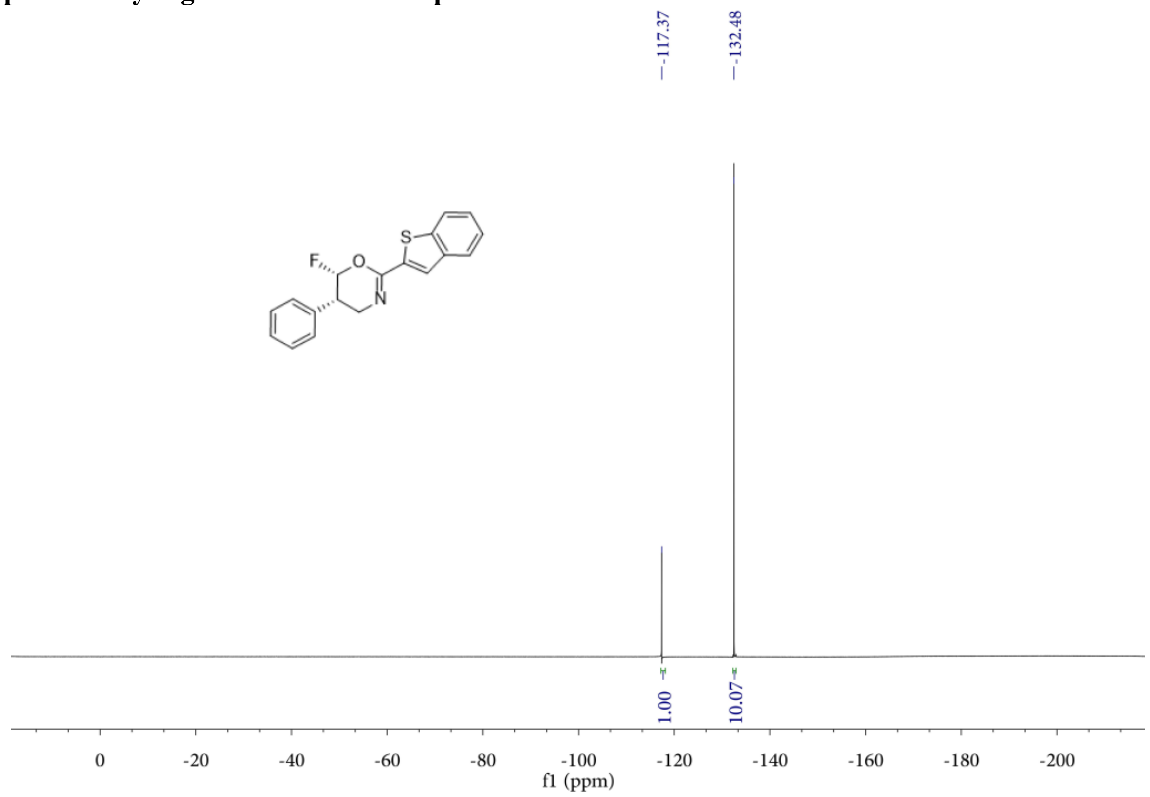

Supplementary Figure 168. ^{1}H NMR spectrum of 39b

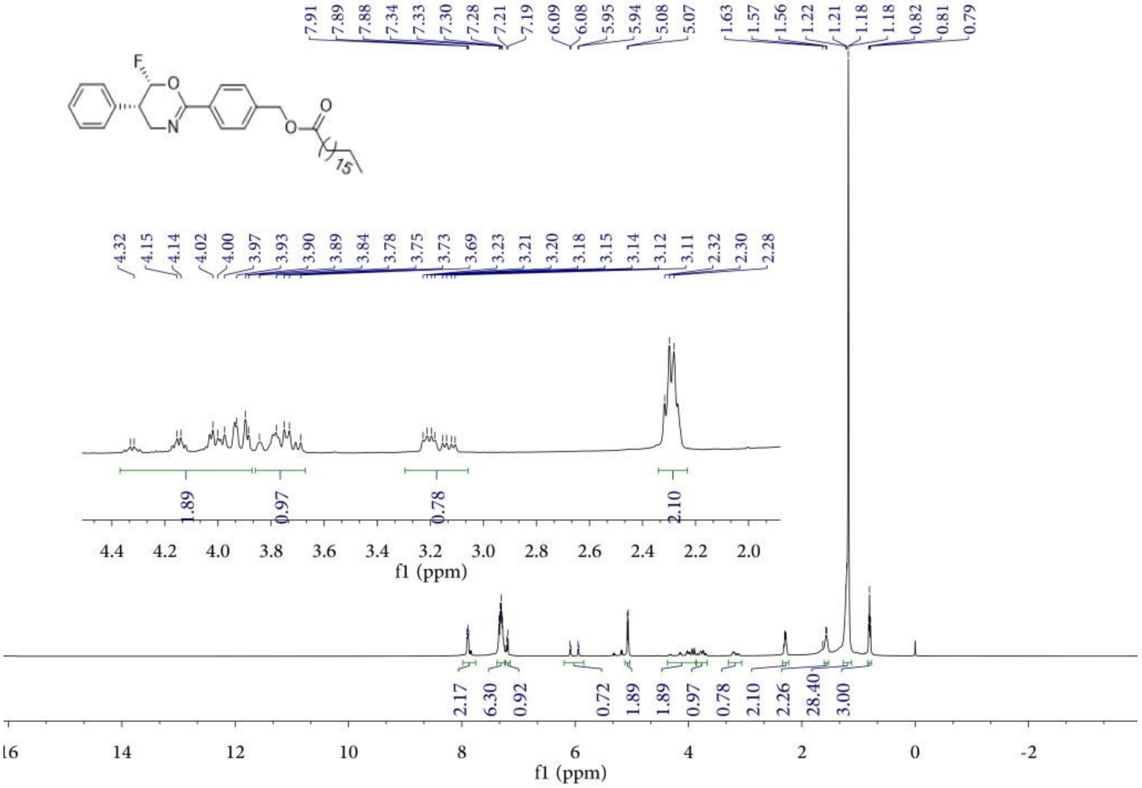

Supplementary Figure 169. ^{13}C NMR spectrum of 39b

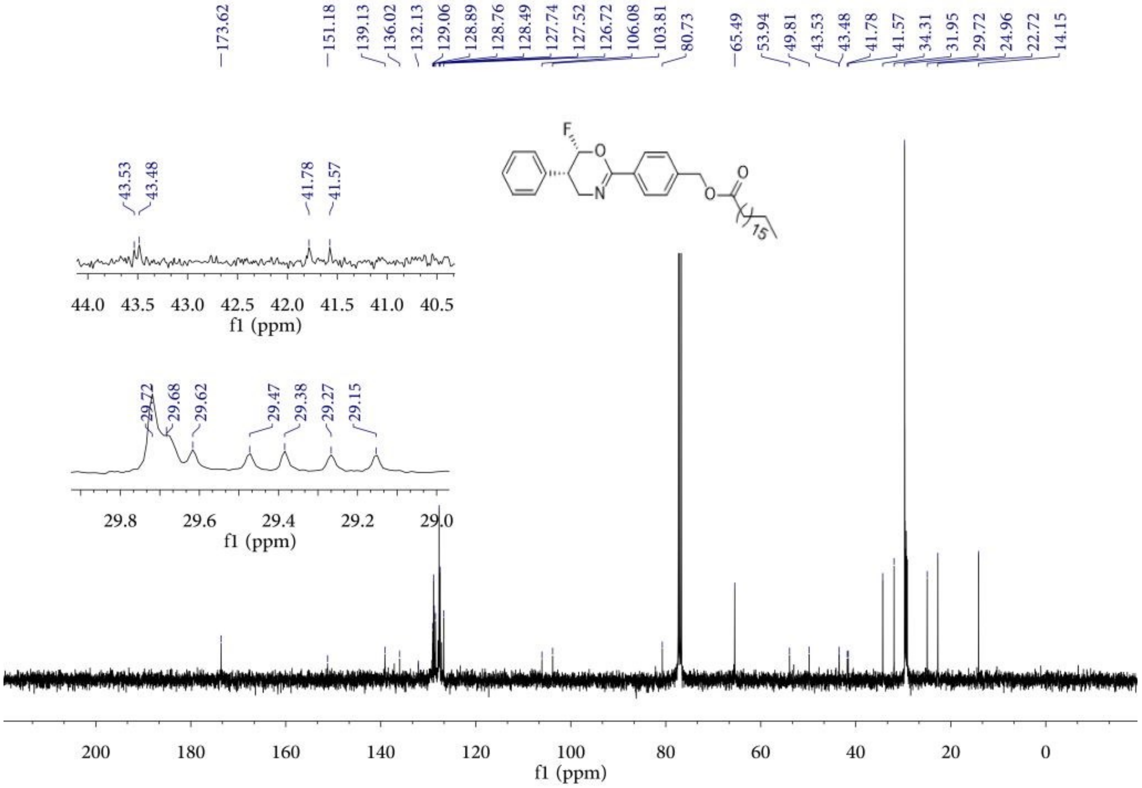

Supplementary Figure 170. ^{19}F NMR spectrum of 39b

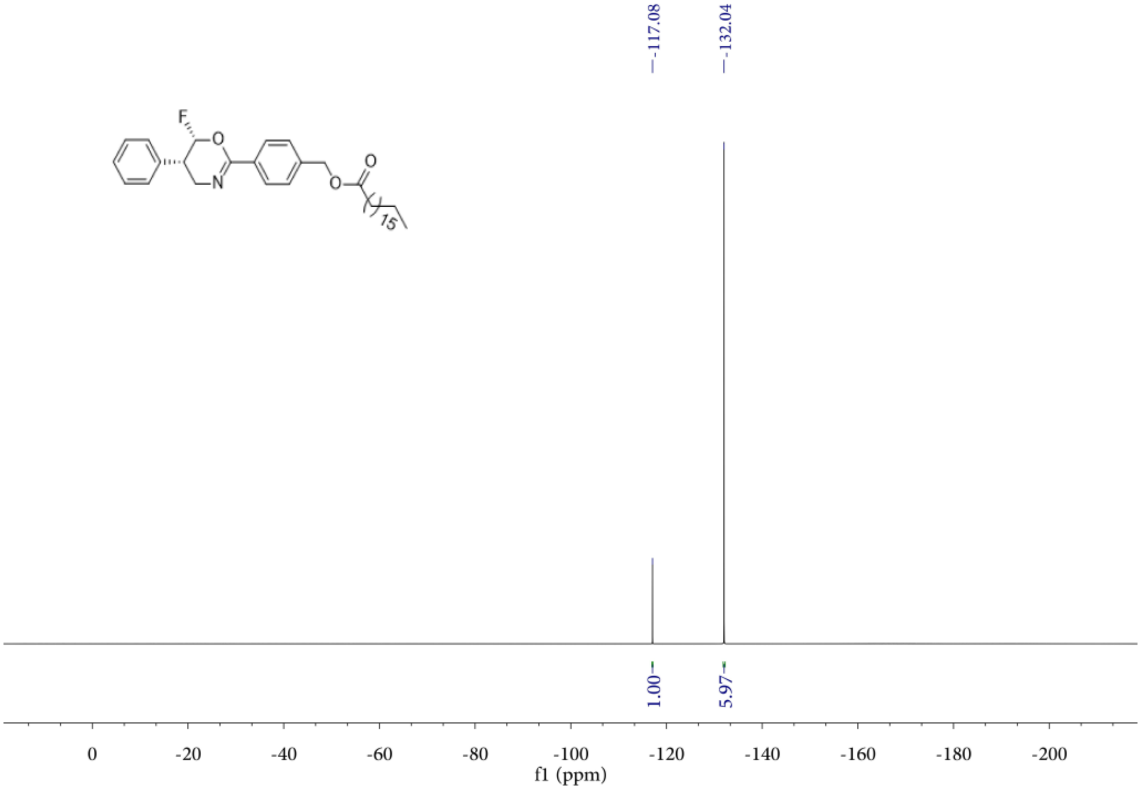

Supplementary Figure 171. ^{1}H NMR spectrum of 40b

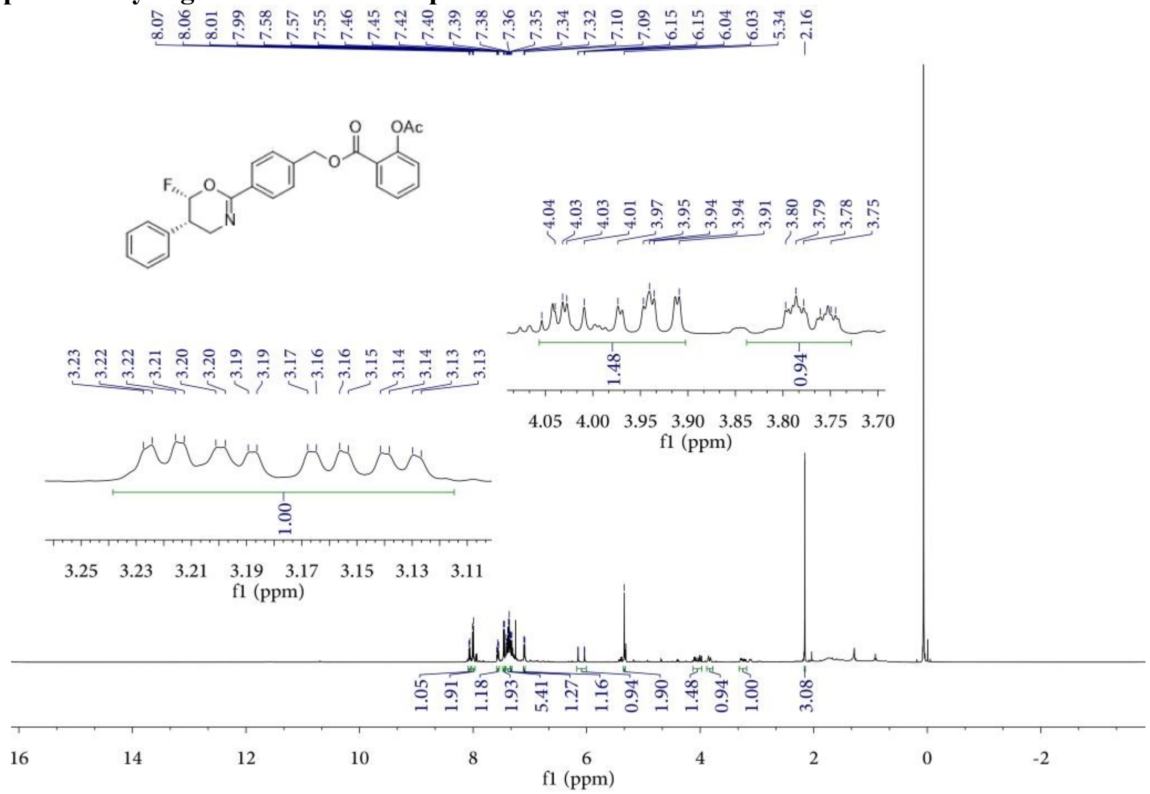

Supplementary Figure 172. ^{13}C NMR spectrum of 40b

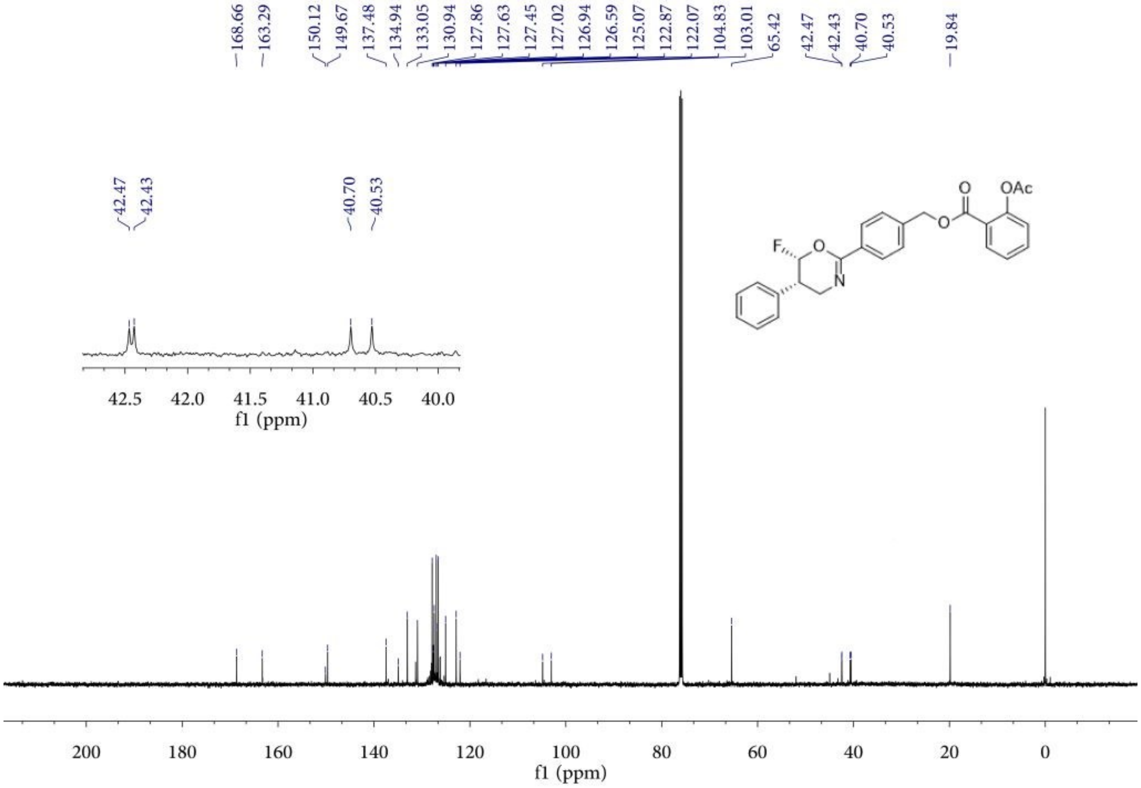

Supplementary Figure 173. ^{19}F NMR spectrum of 40b

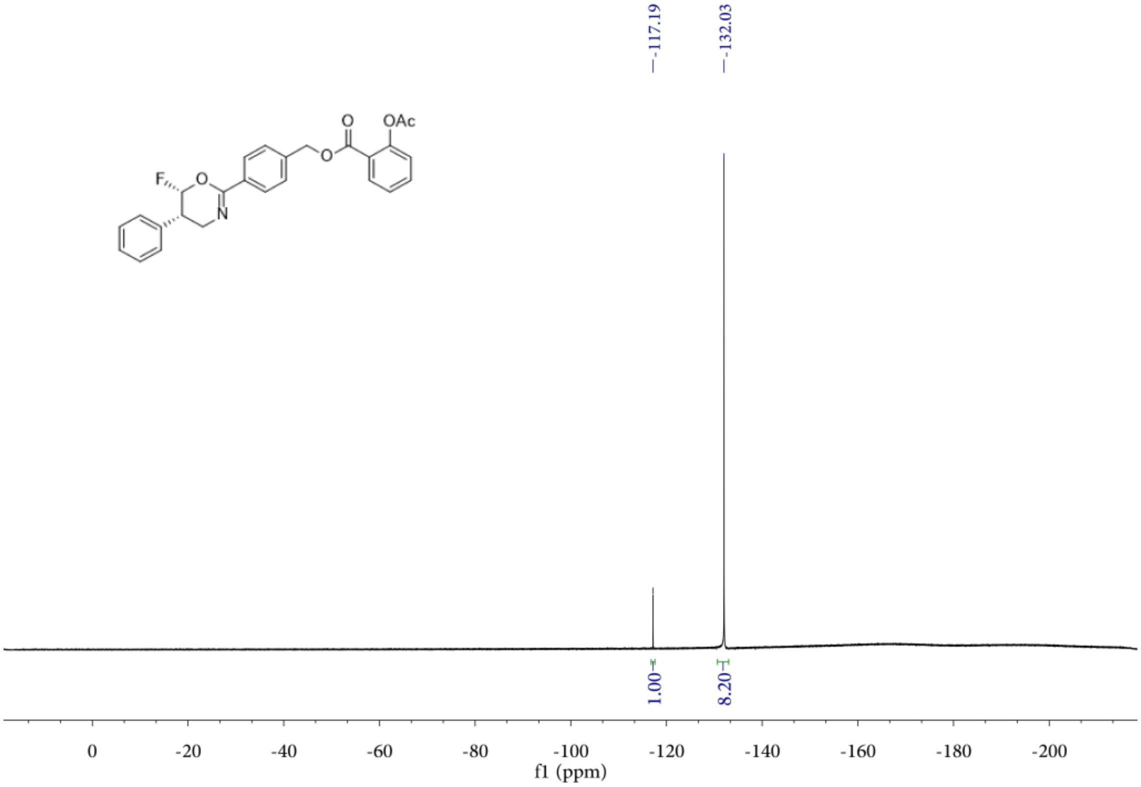

Supplementary Figure 174. ^{1}H NMR spectrum of 41b

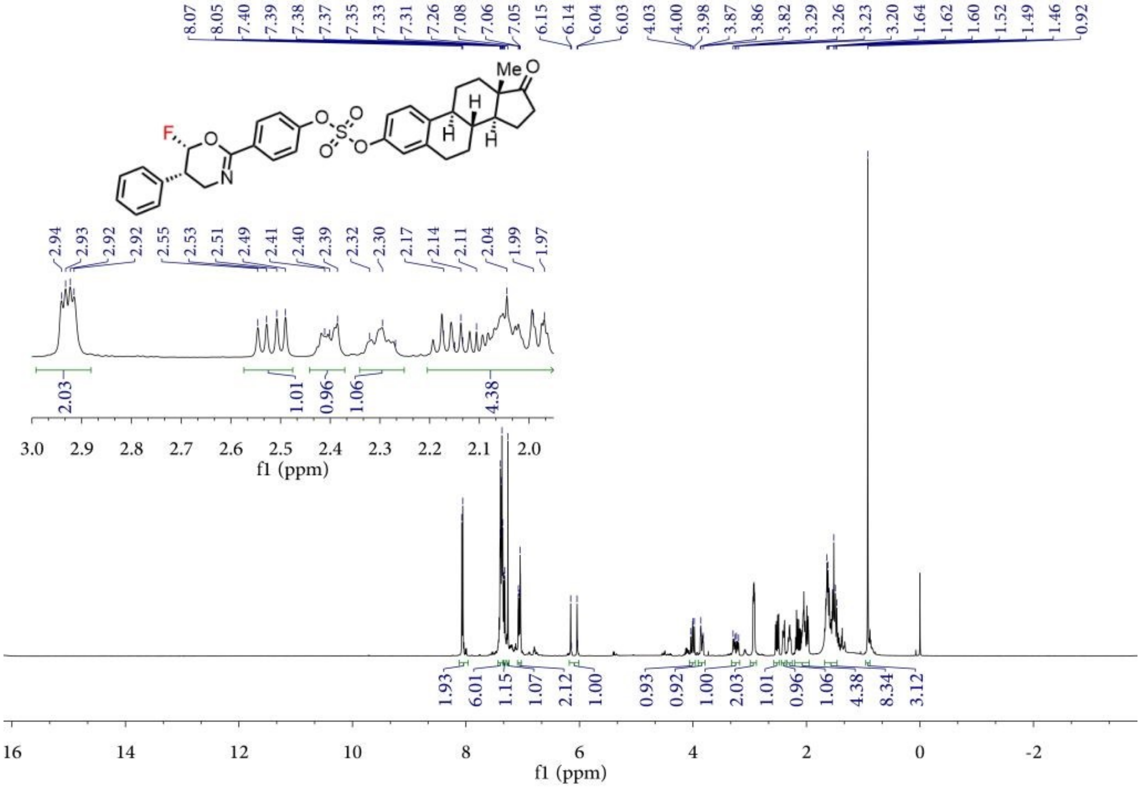

Supplementary Figure 175. ^{13}C NMR spectrum of 41b

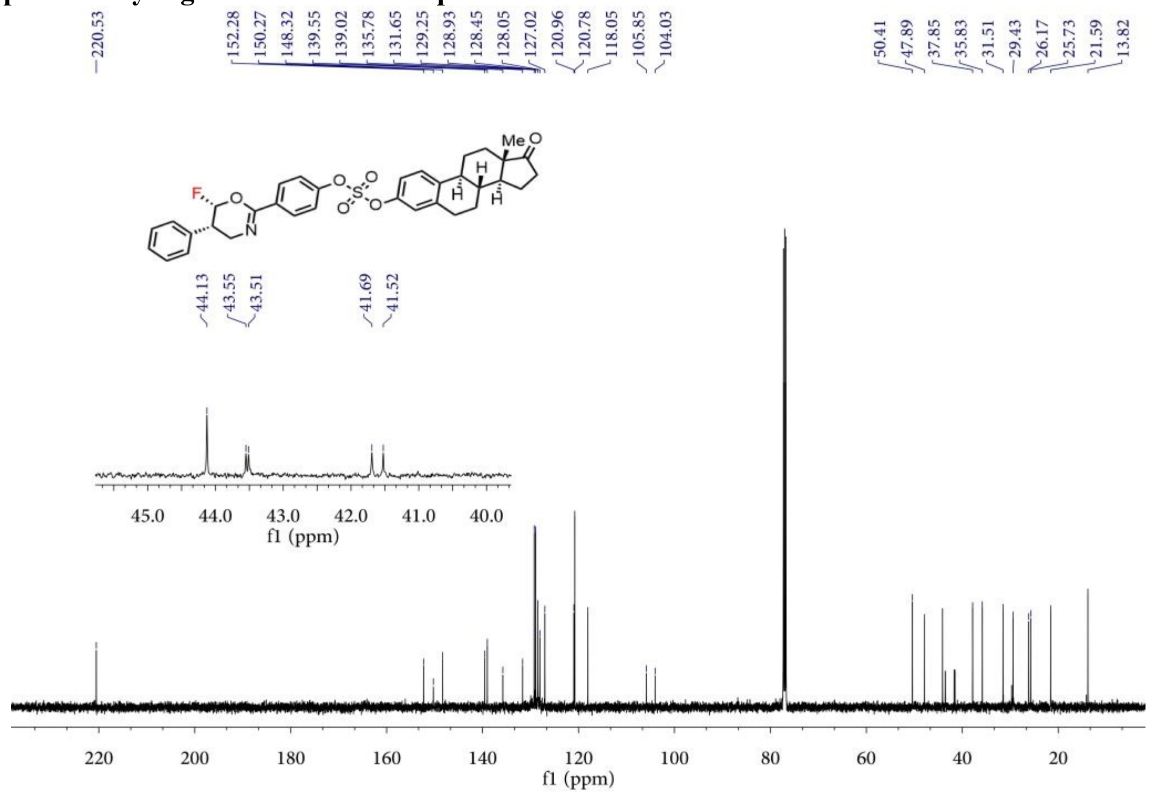

Supplementary Figure 176. ^{19}F NMR spectrum of 41b

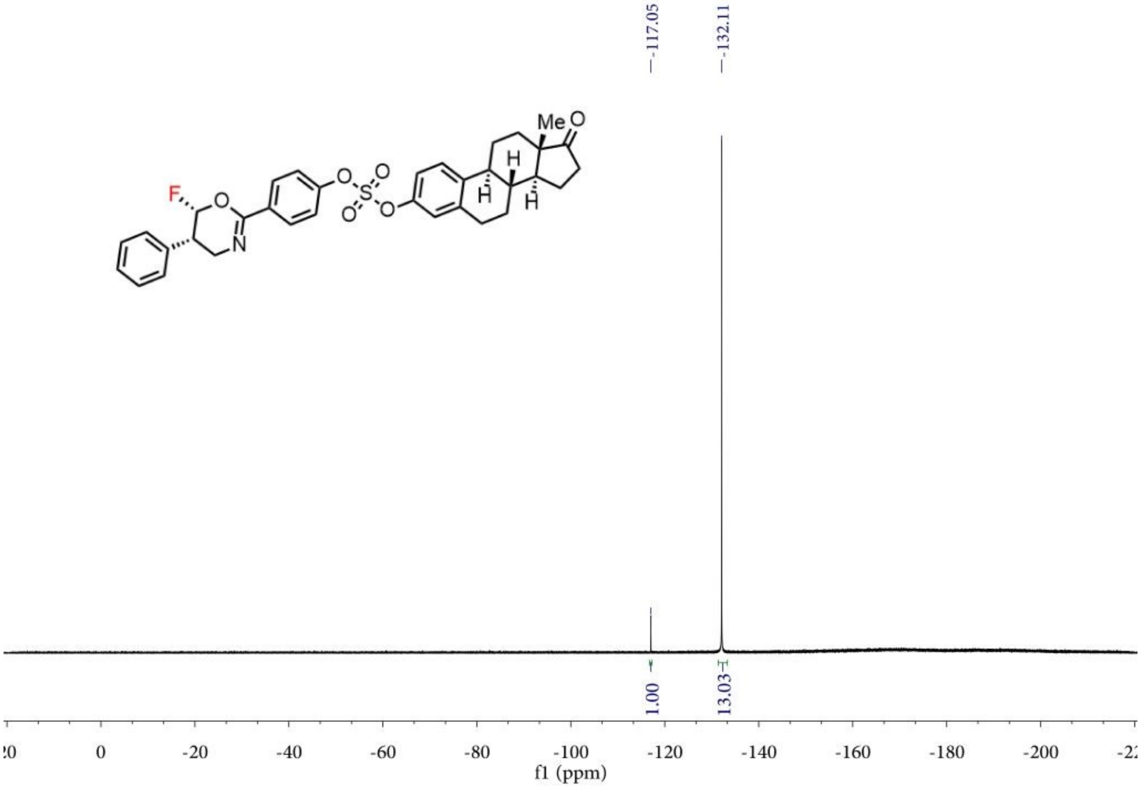

Supplementary Figure 177. ^{1}H NMR spectrum of 42b

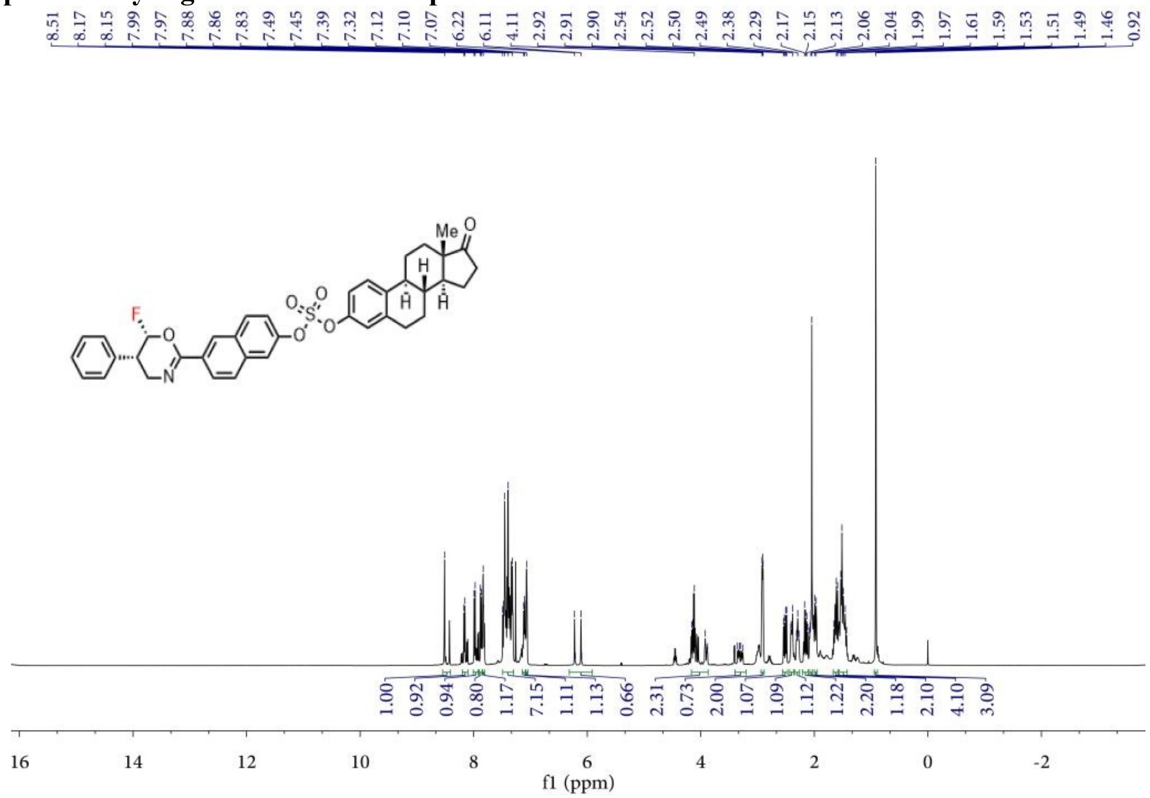

Supplementary Figure 178. ^{13}C NMR spectrum of 42b

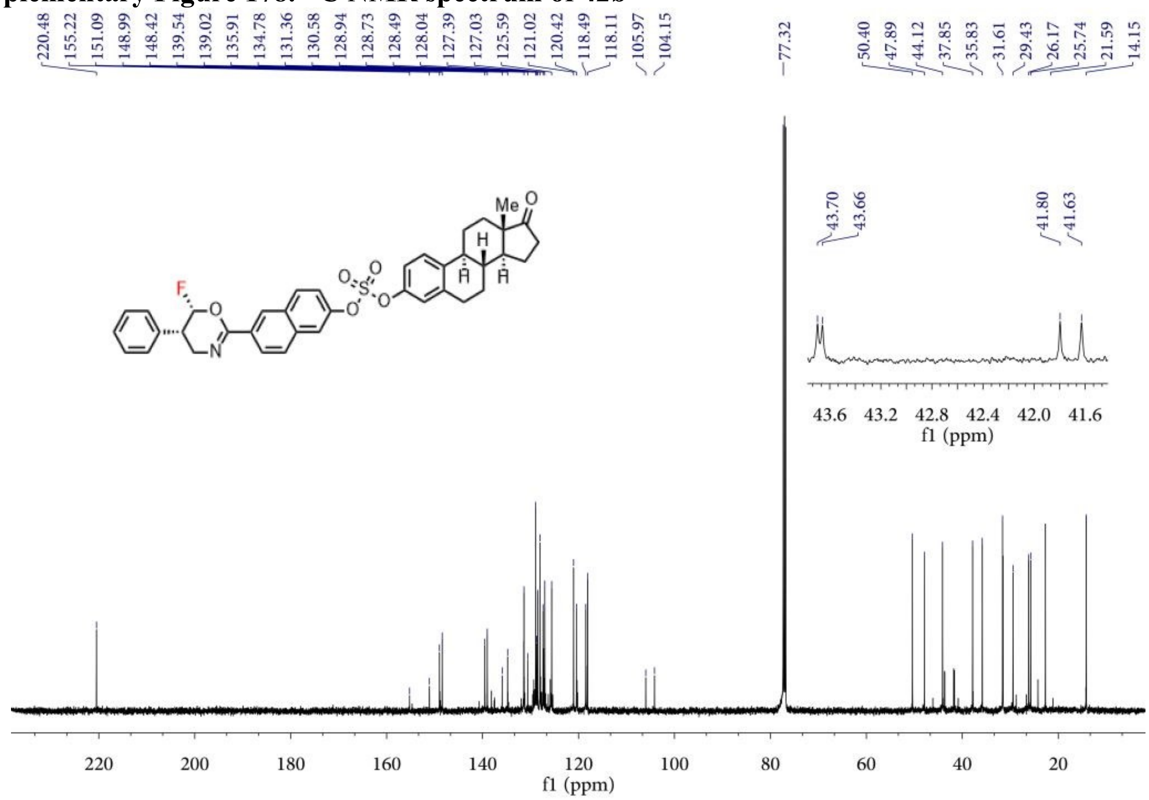

Supplementary Figure 179. ^{19}F NMR spectrum of 42b

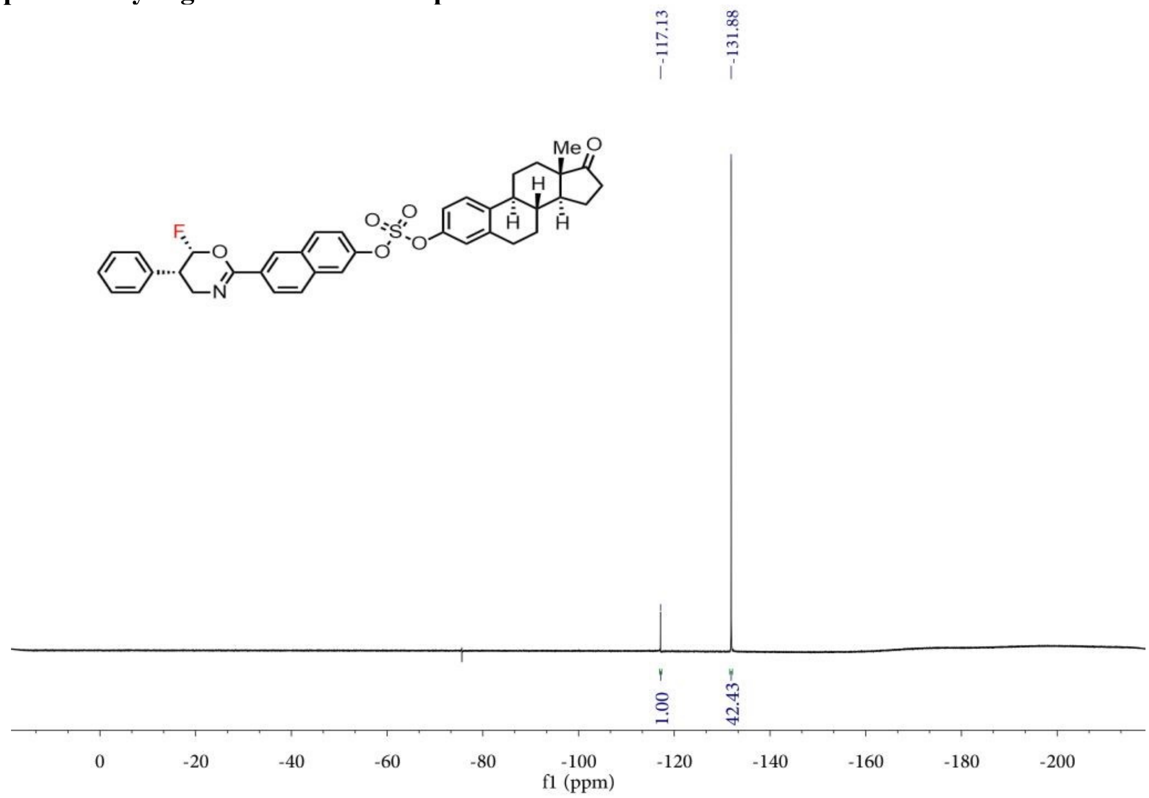

Supplementary Figure 180. ^{1}H NMR spectrum of 43b

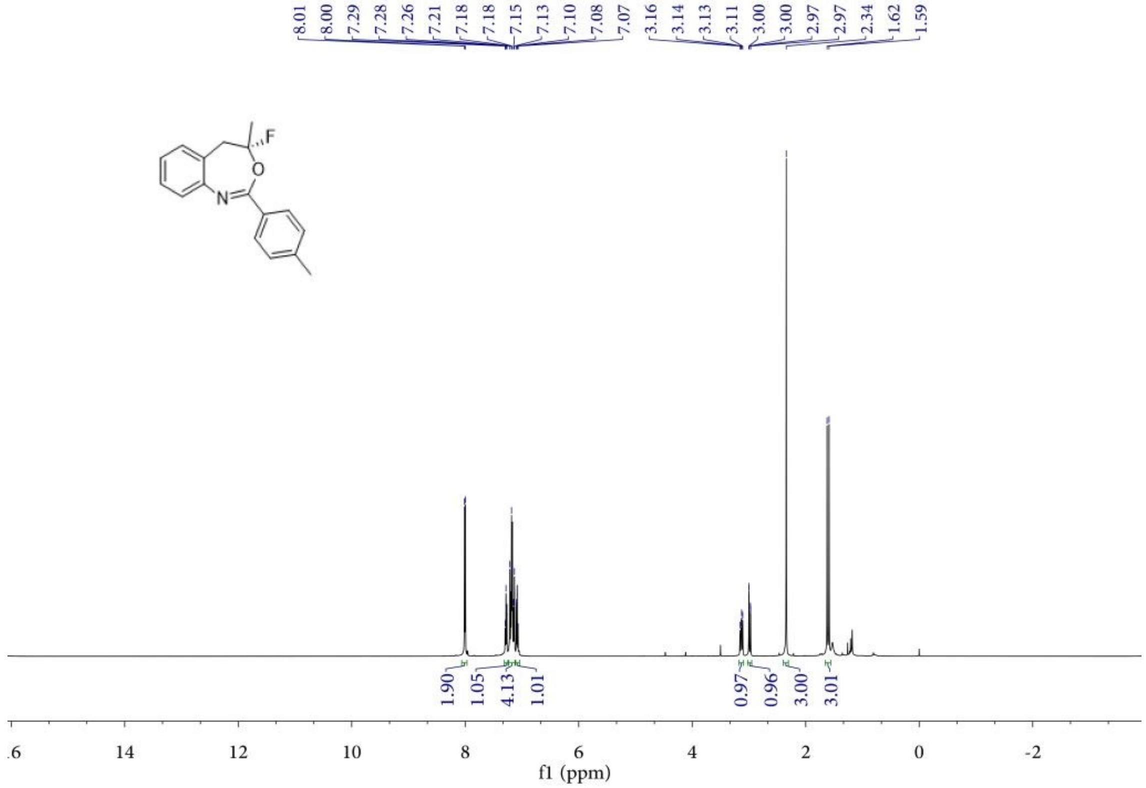

Supplementary Figure 181. ^{13}C NMR spectrum of 43b

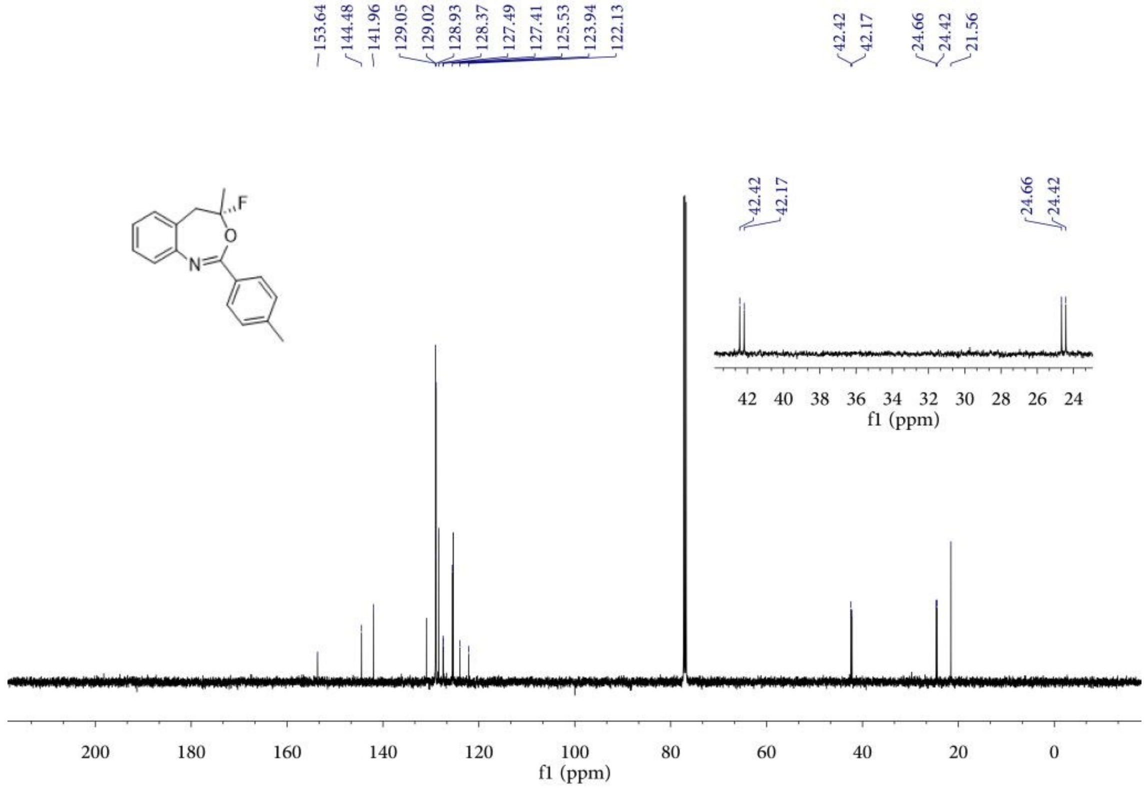

Supplementary Figure 182. ^{19}F NMR spectrum of 43b

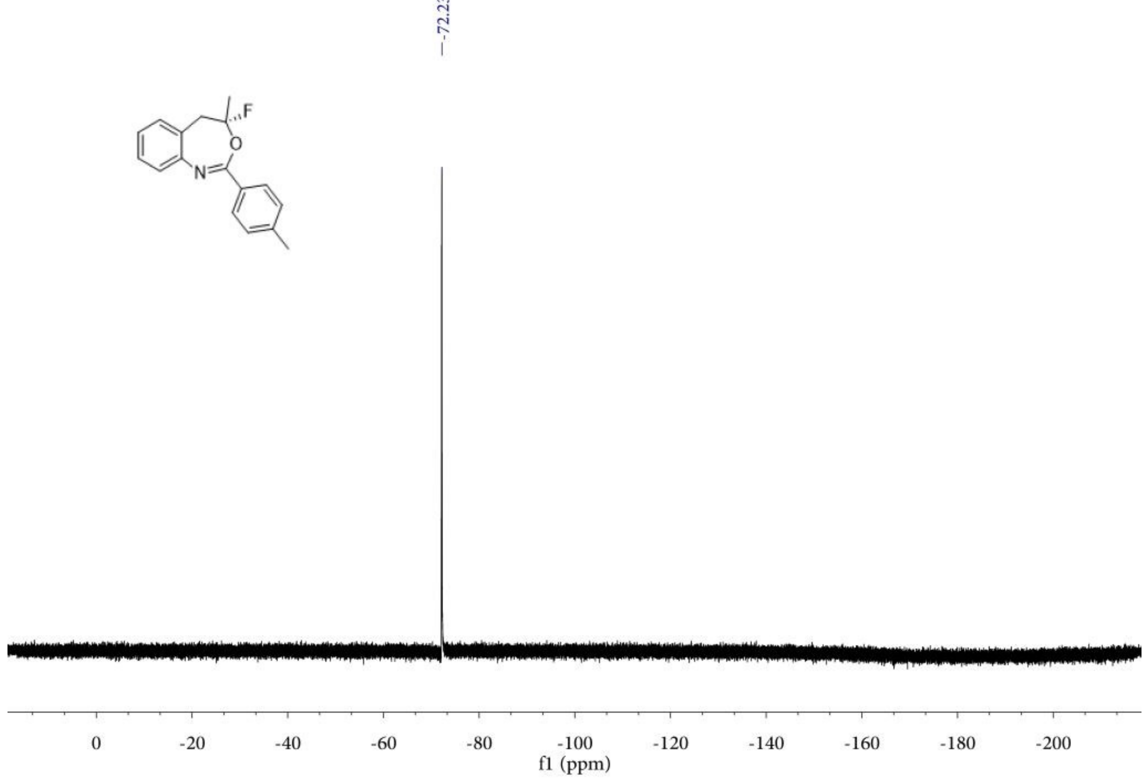

Supplementary Figure 183. ^{1}H NMR spectrum of 44b

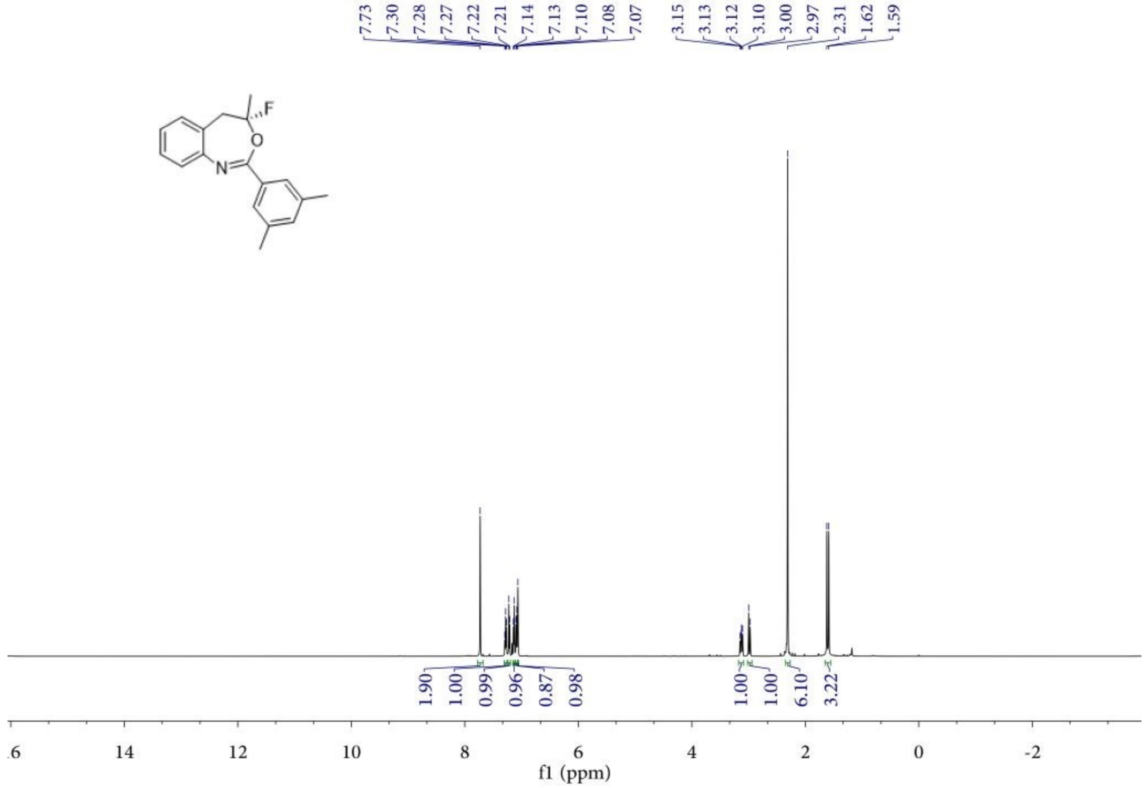

Supplementary Figure 184. ^{13}C NMR spectrum of 44b

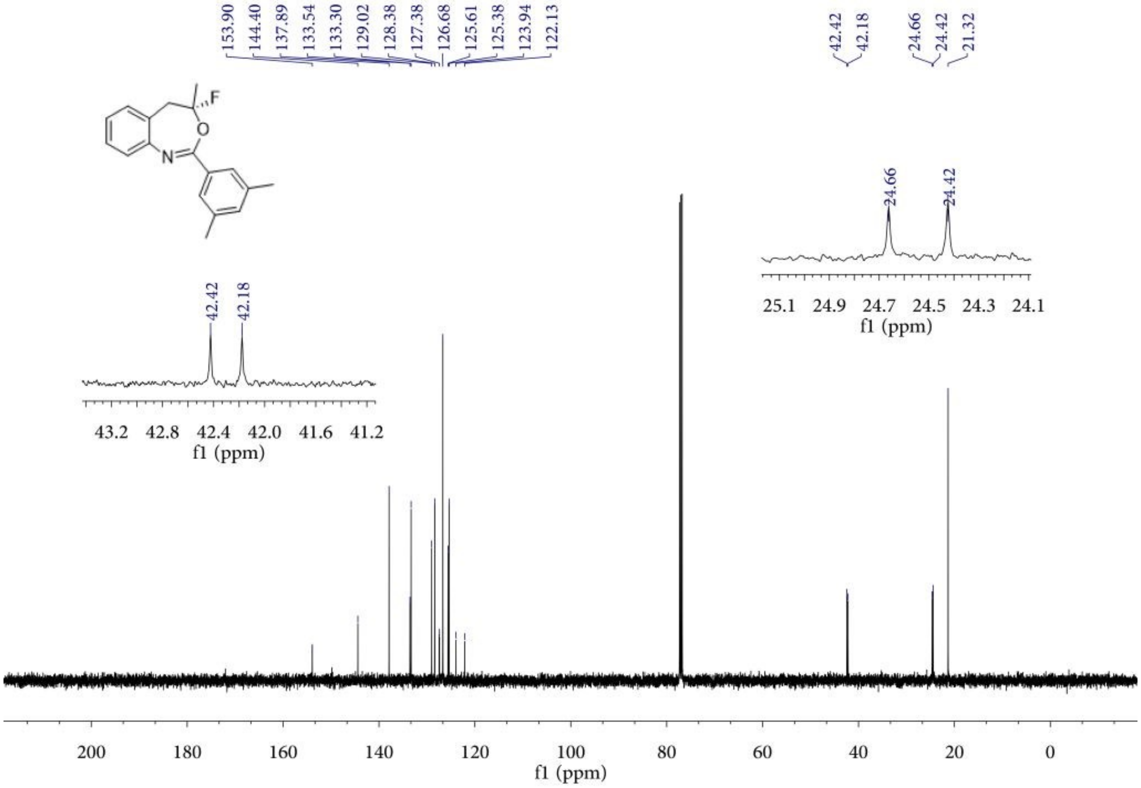

Supplementary Figure 185. ^{19}F NMR spectrum of 44b

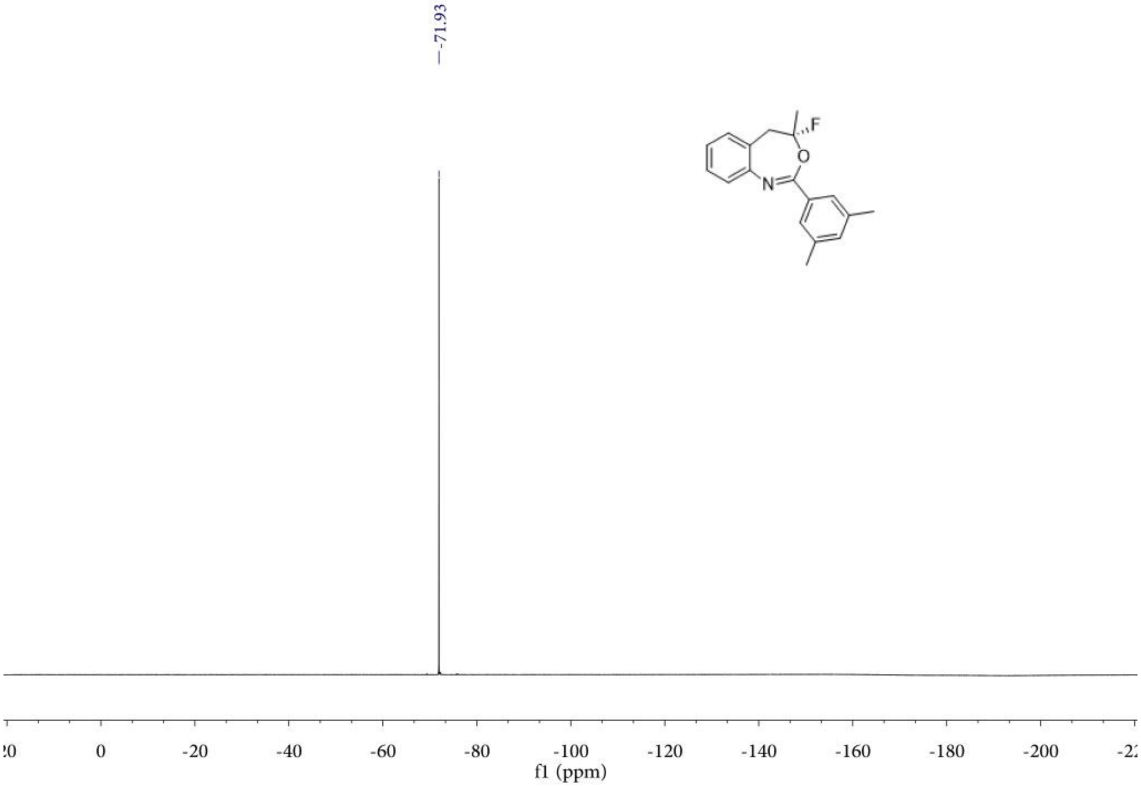

Supplementary Figure 186. ^{1}H NMR spectrum of 45b

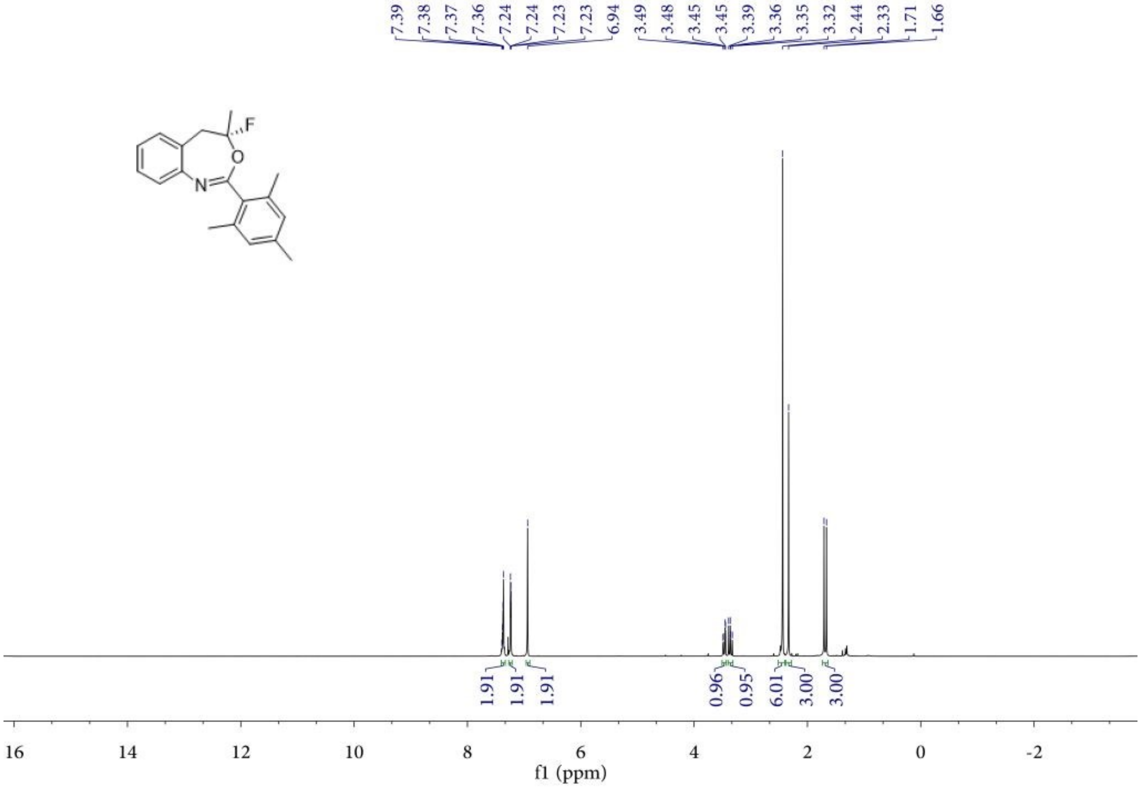

Supplementary Figure 187. ^{13}C NMR spectrum of 45b

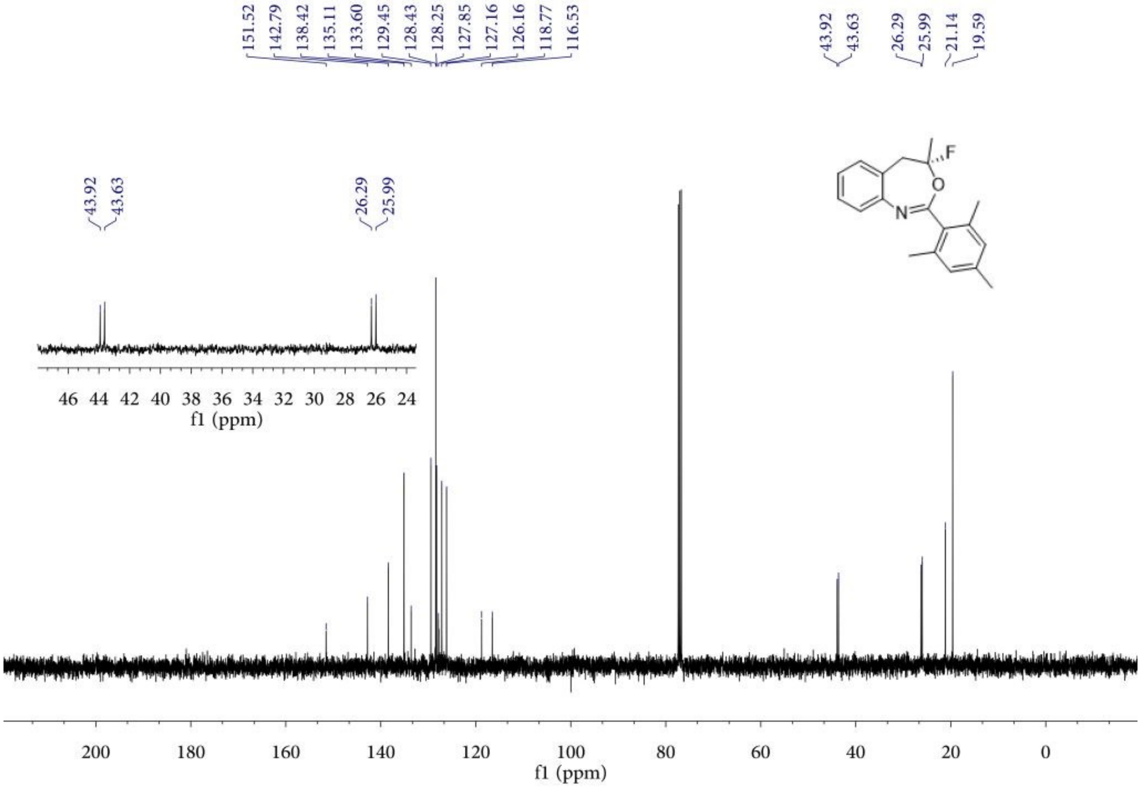

Supplementary Figure 188. ^{19}F NMR spectrum of 45b

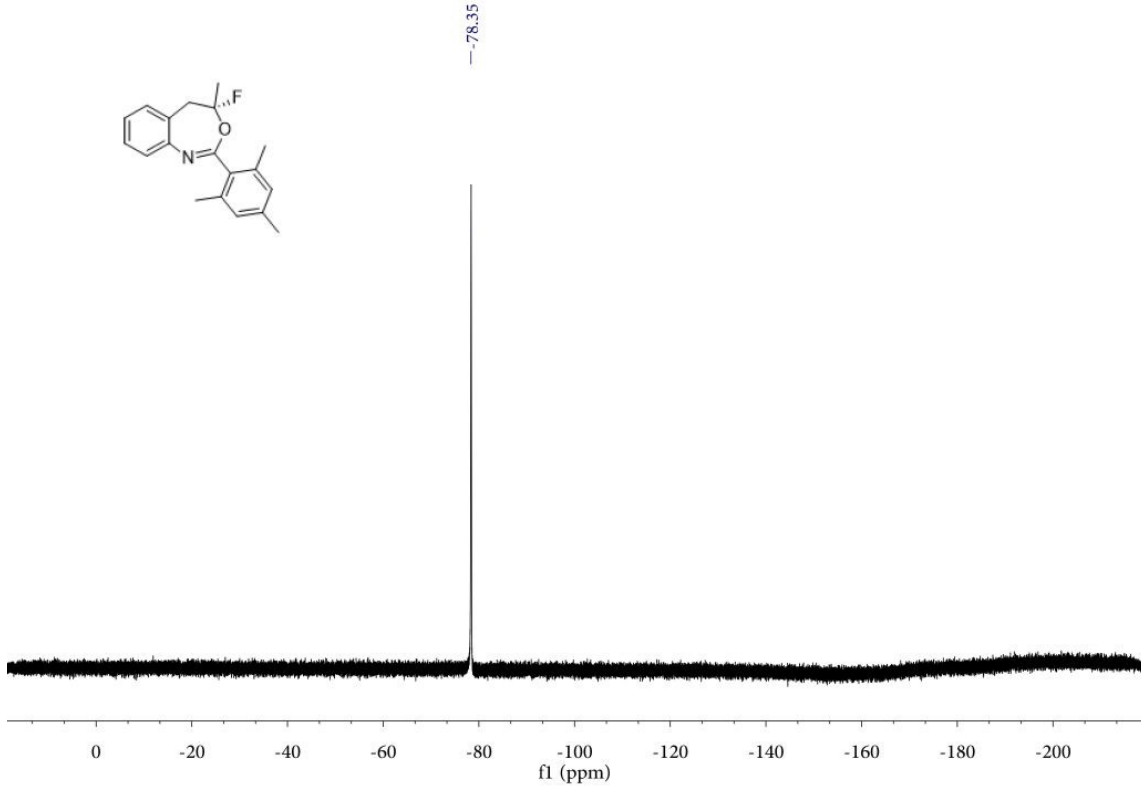

Supplementary Figure 189. ^{1}H NMR spectrum of 46b

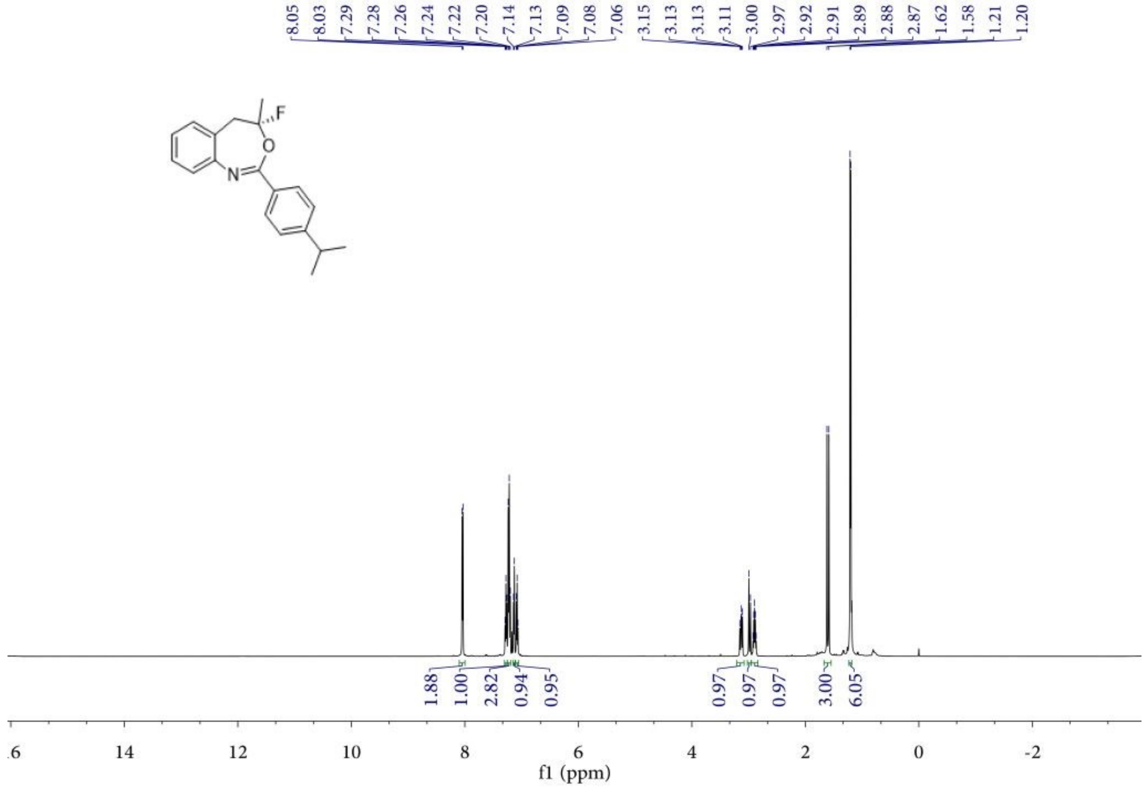

Supplementary Figure 190. ^{13}C NMR spectrum of 46b

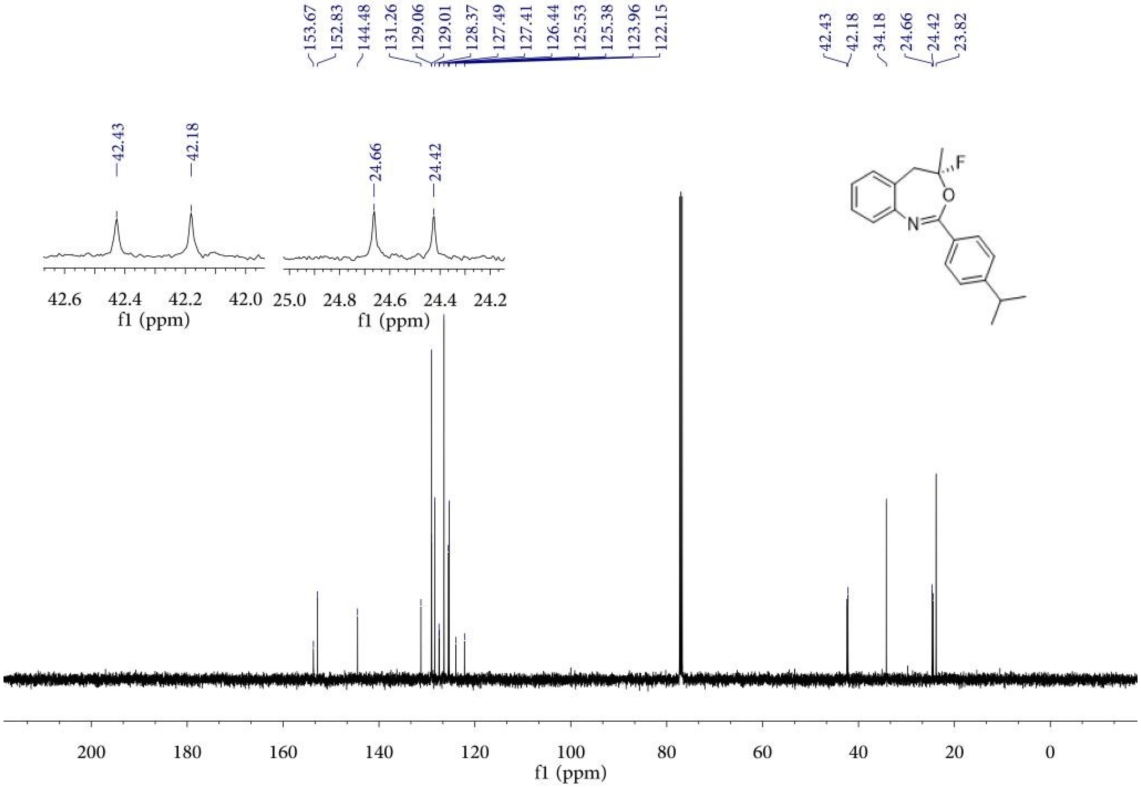

Supplementary Figure 191. ^{19}F NMR spectrum of 46b

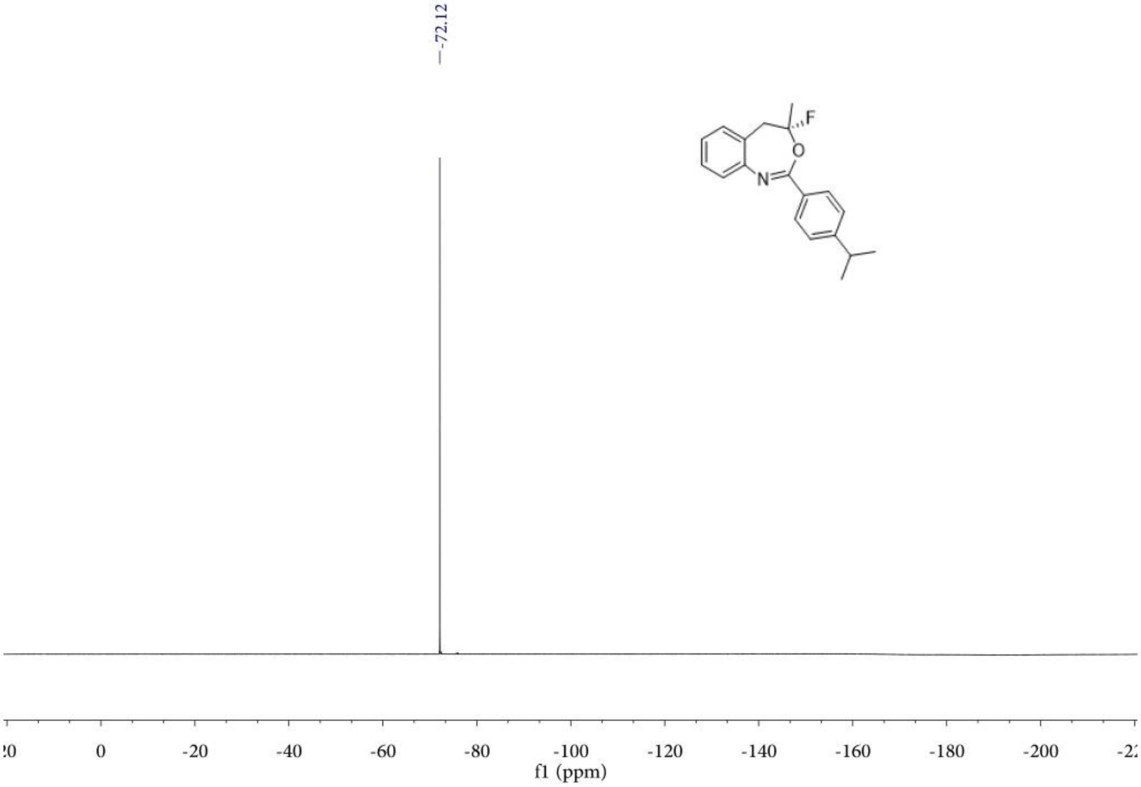

Supplementary Figure 192. ^{1}H NMR spectrum of 47b

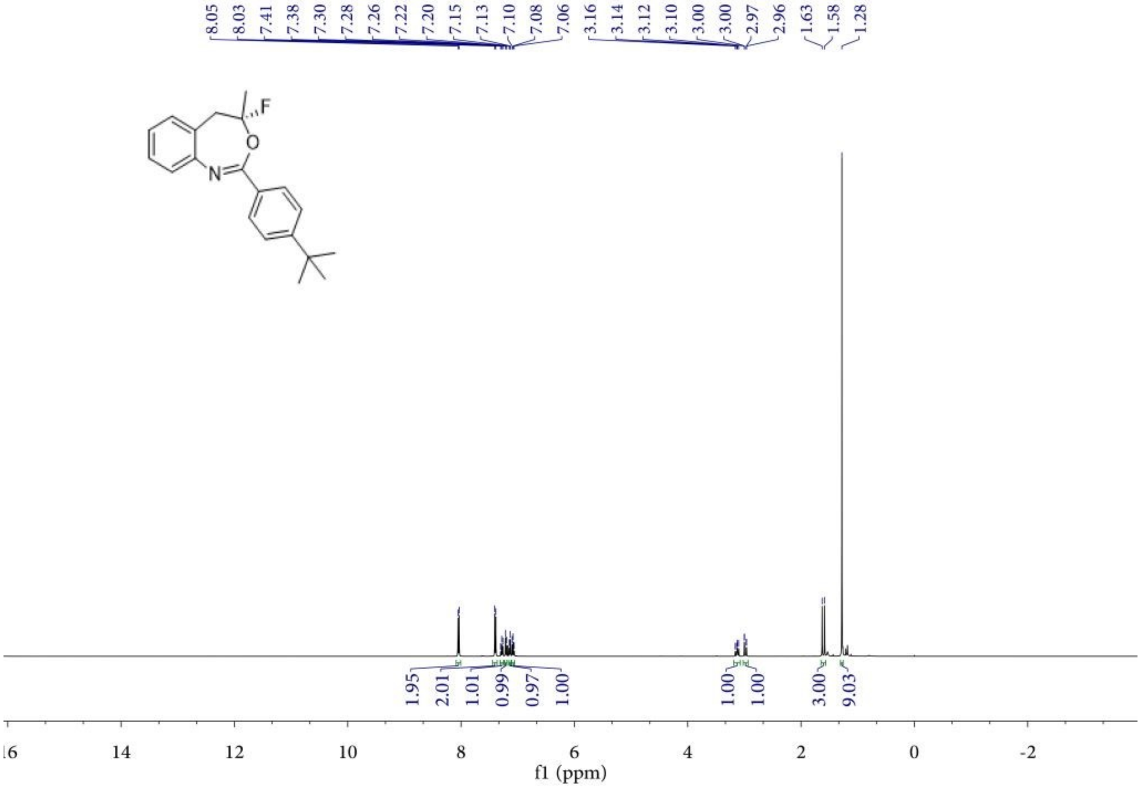

Supplementary Figure 193. ^{13}C NMR spectrum of 47b

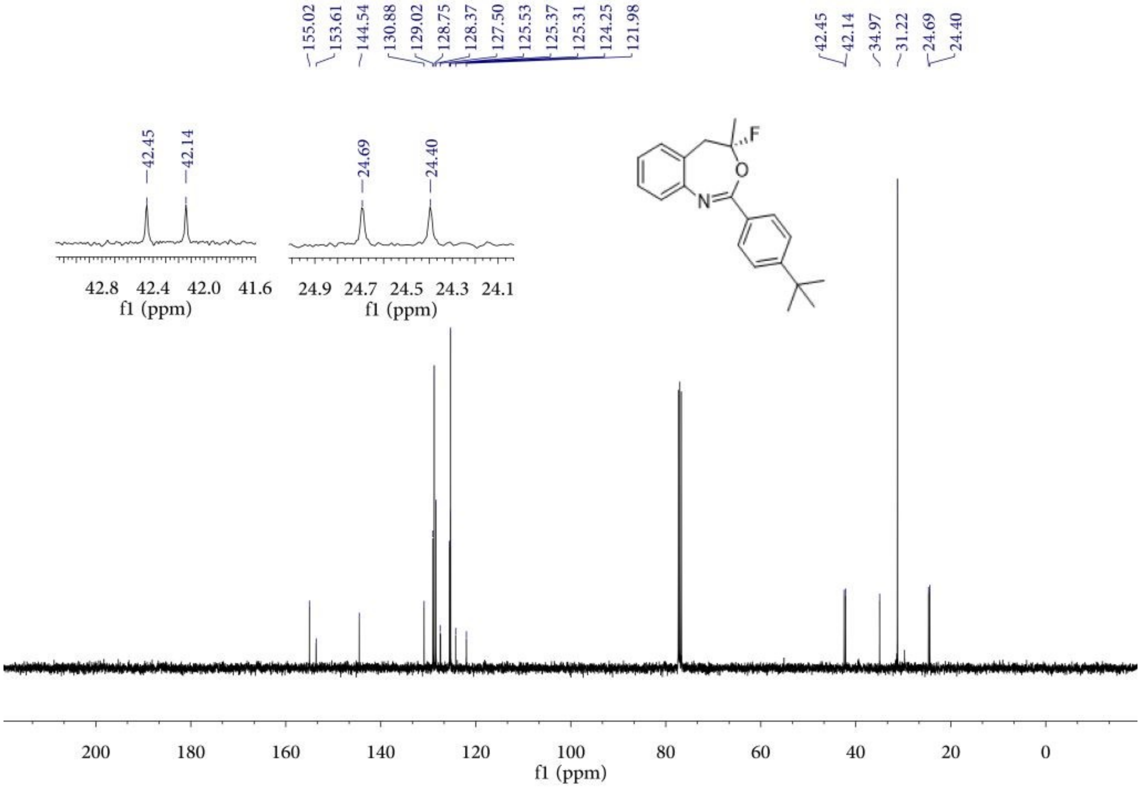

Supplementary Figure 194. ^{19}F NMR spectrum of 47b

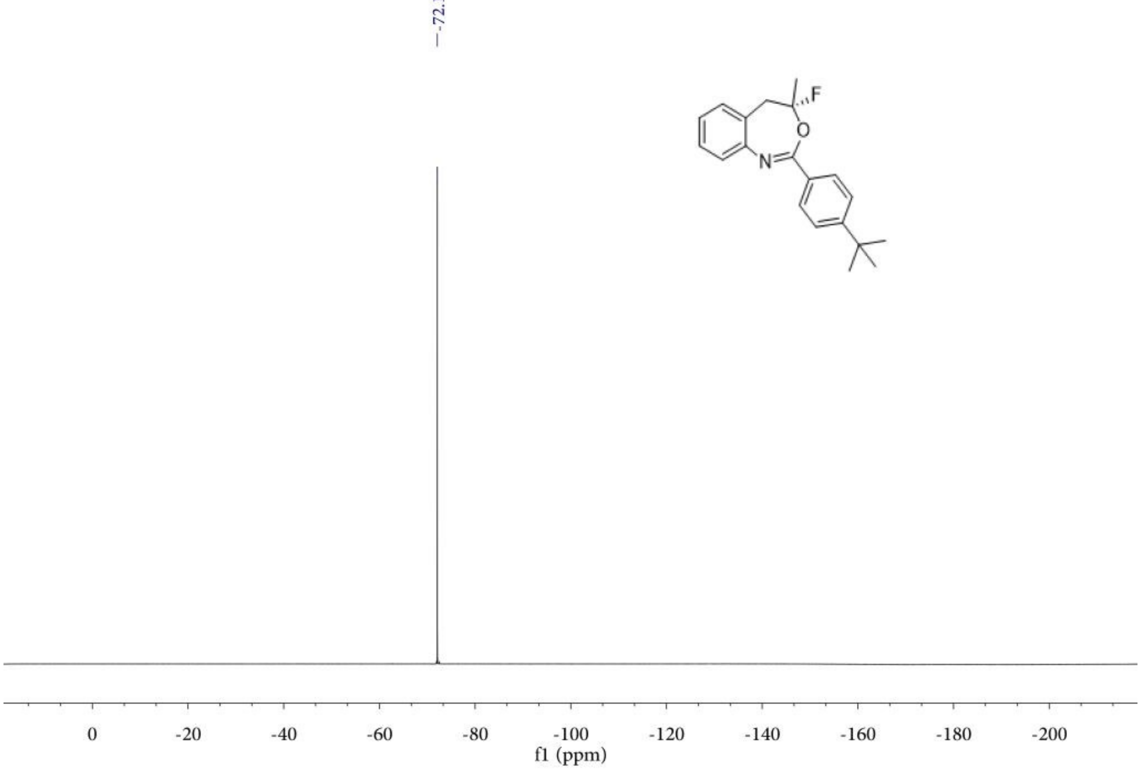

Supplementary Figure 195. ^{1}H NMR spectrum of 48b

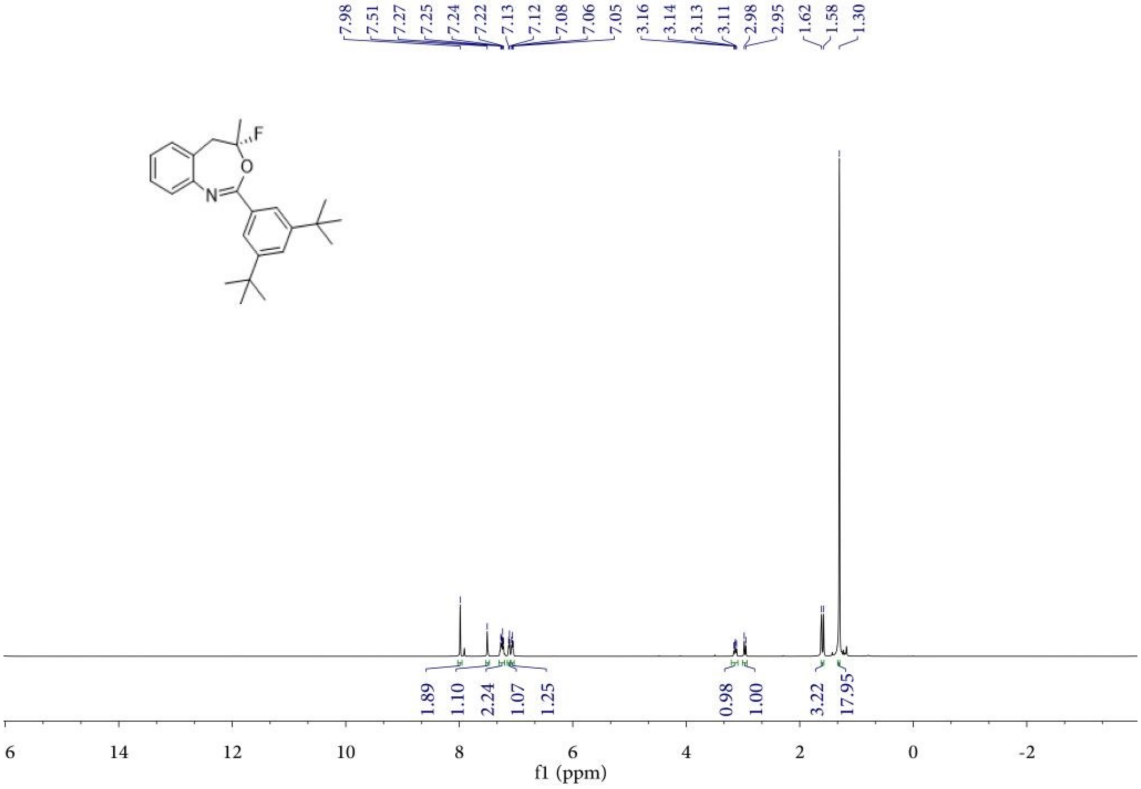

Supplementary Figure 196. ^{13}C NMR spectrum of 48b

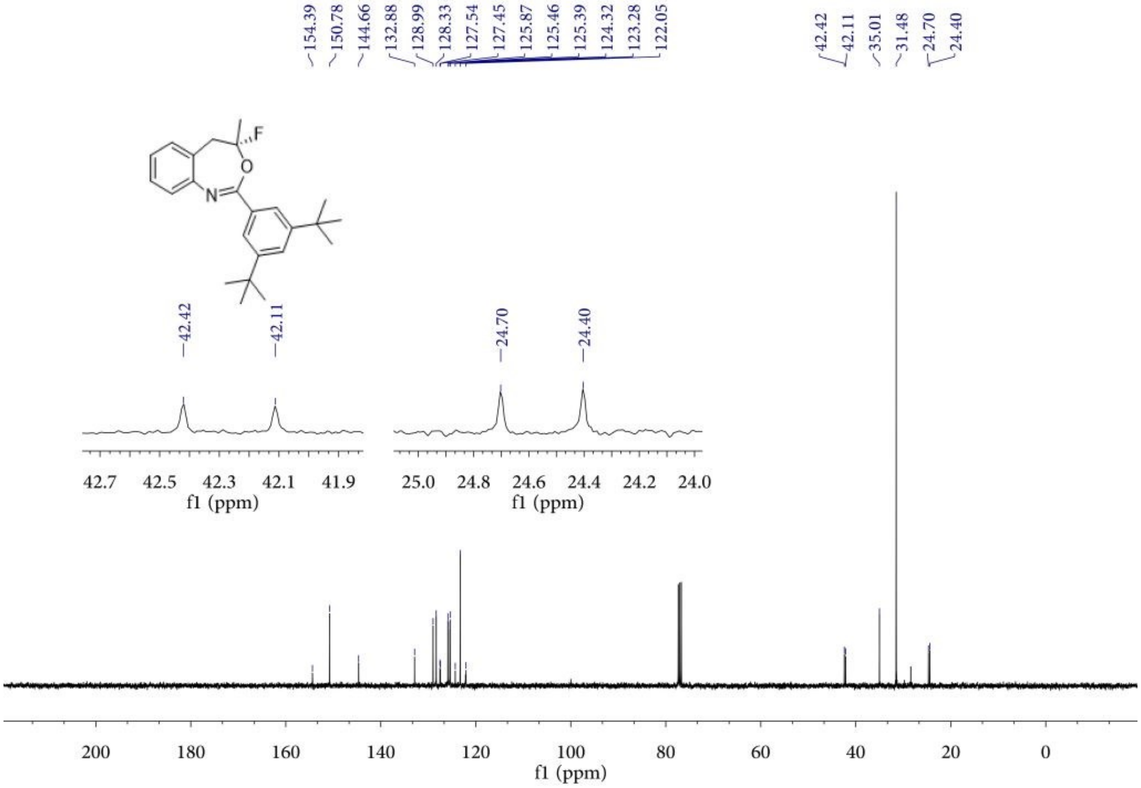

Supplementary Figure 197. ^{19}F NMR spectrum of 48b

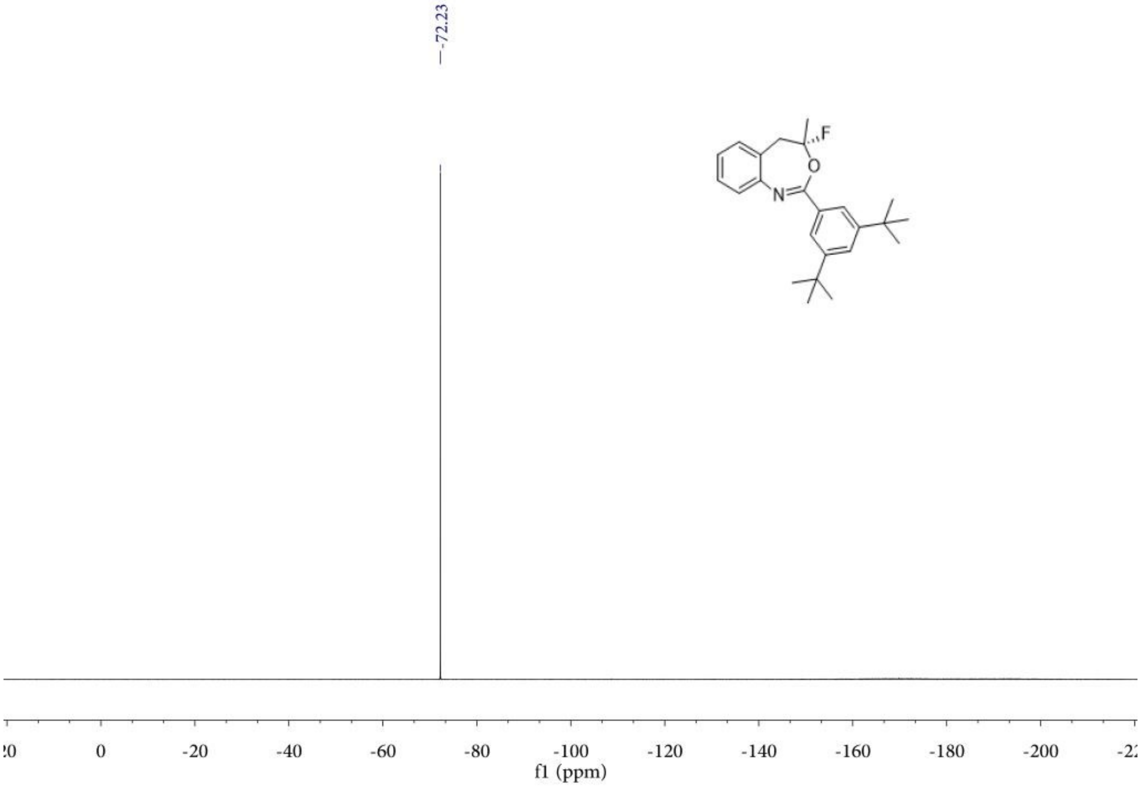

Supplementary Figure 198. ^{1}H NMR spectrum of 49b

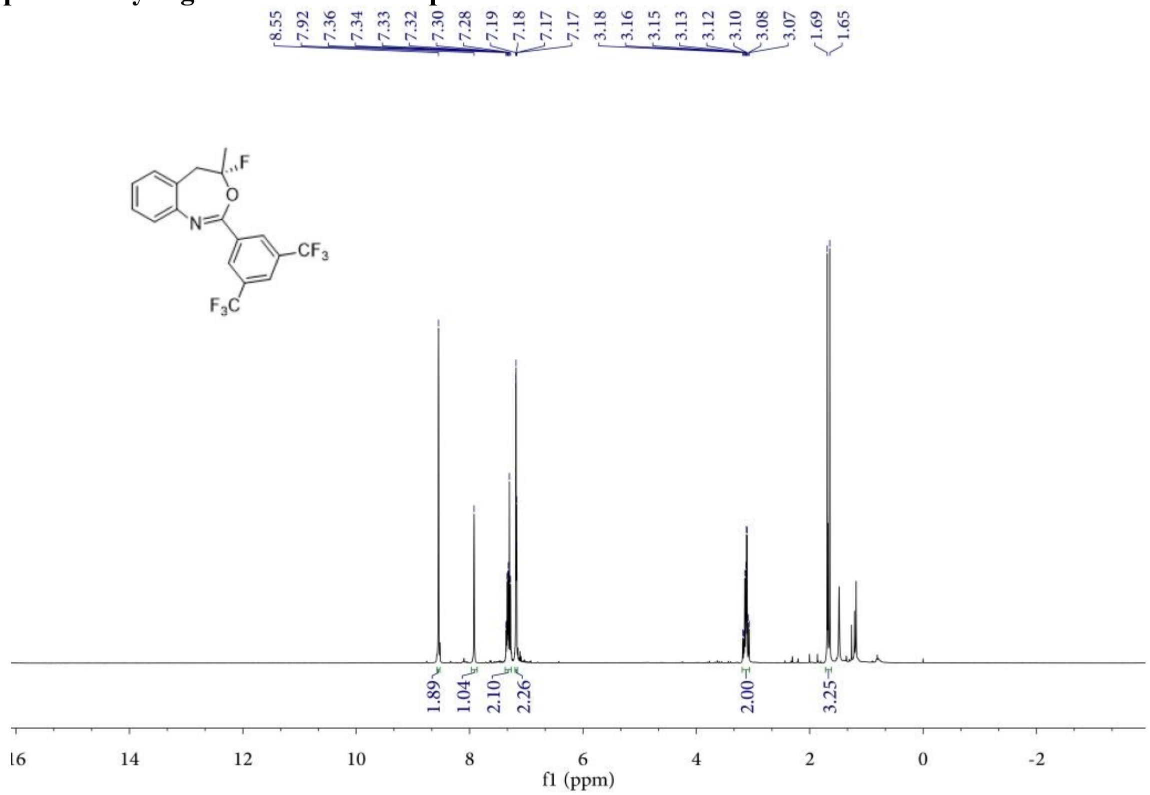

Supplementary Figure 199. ^{13}C NMR spectrum of 49b

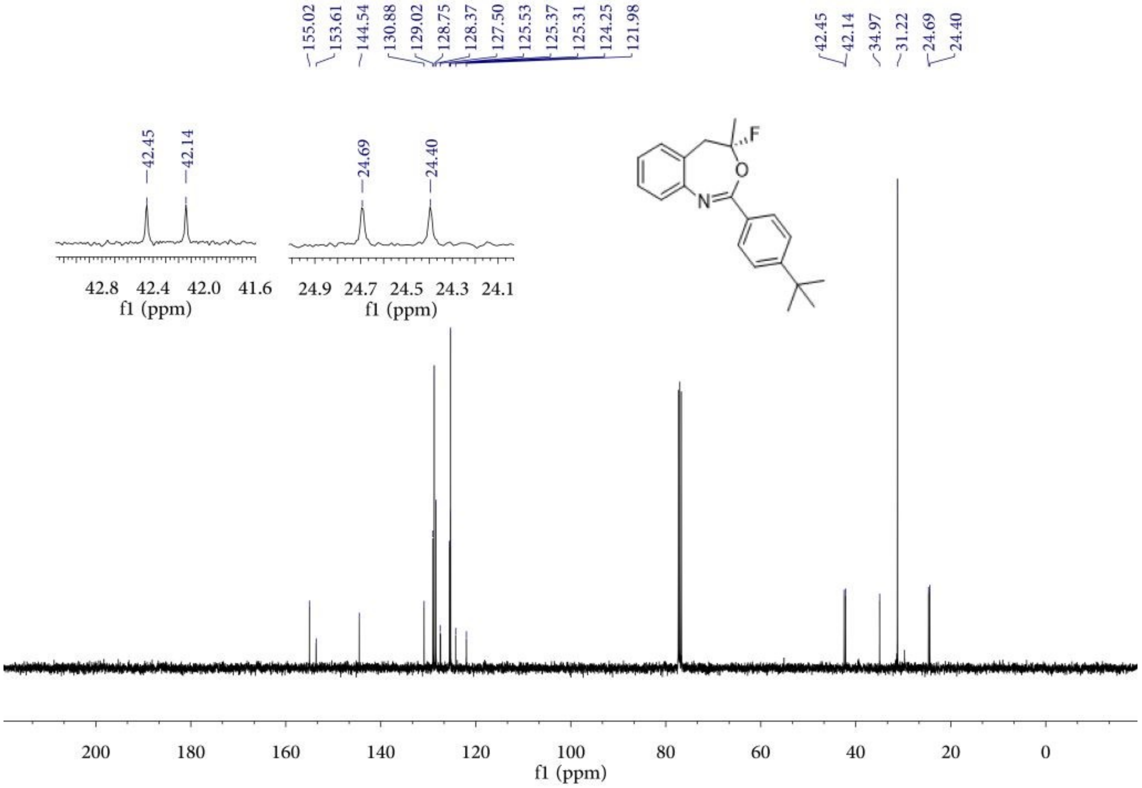

Supplementary Figure 200. ^{19}F NMR spectrum of 49b

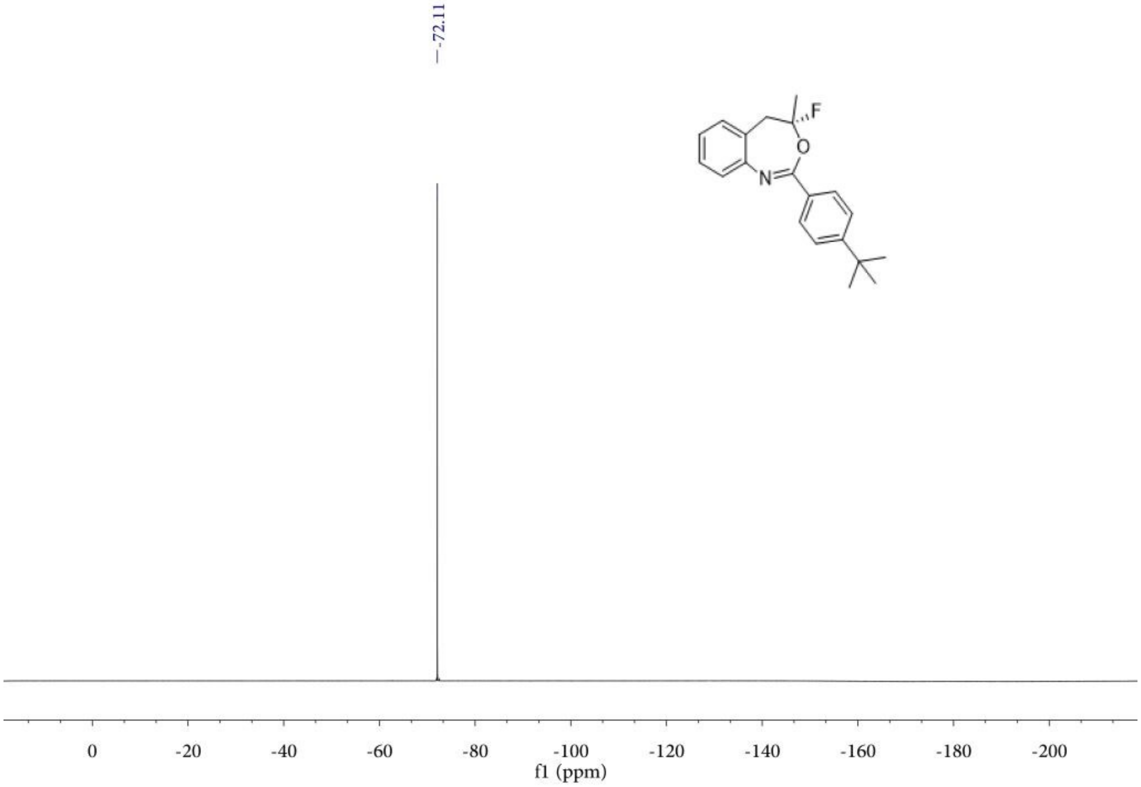

Supplementary Figure 201. ^{1}H NMR spectrum of 50b

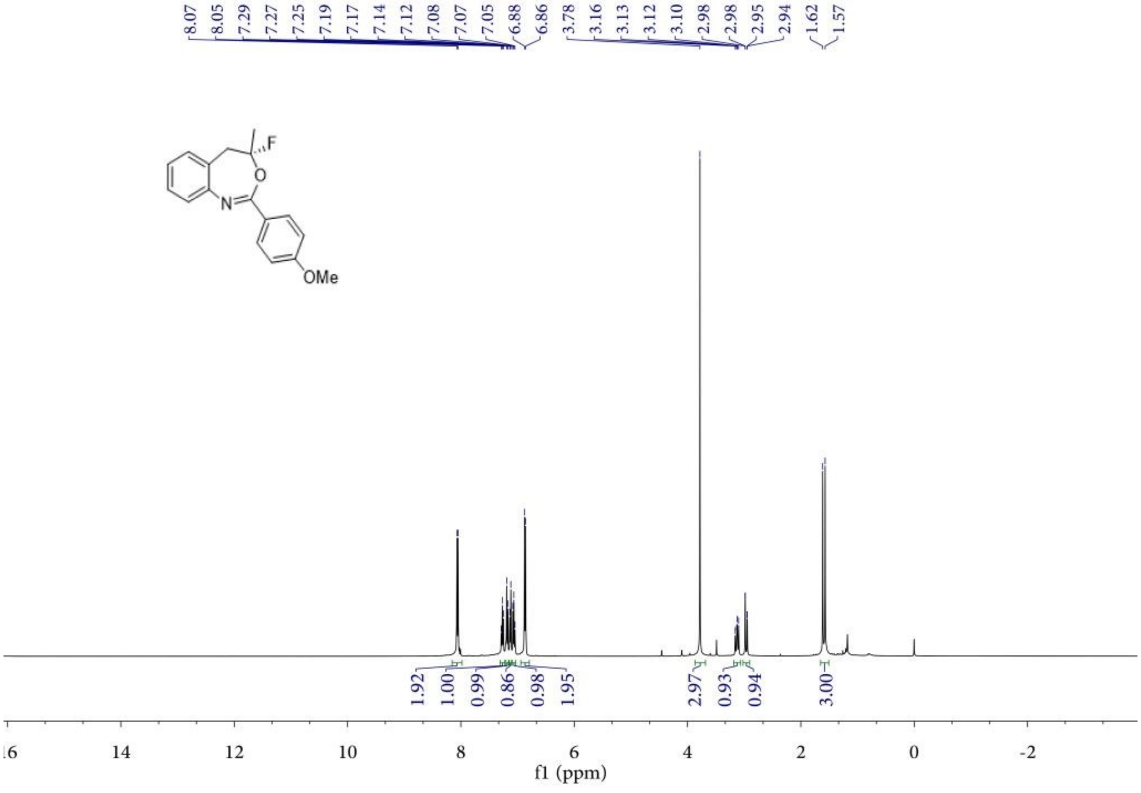

Supplementary Figure 202. ^{13}C NMR spectrum of 50b

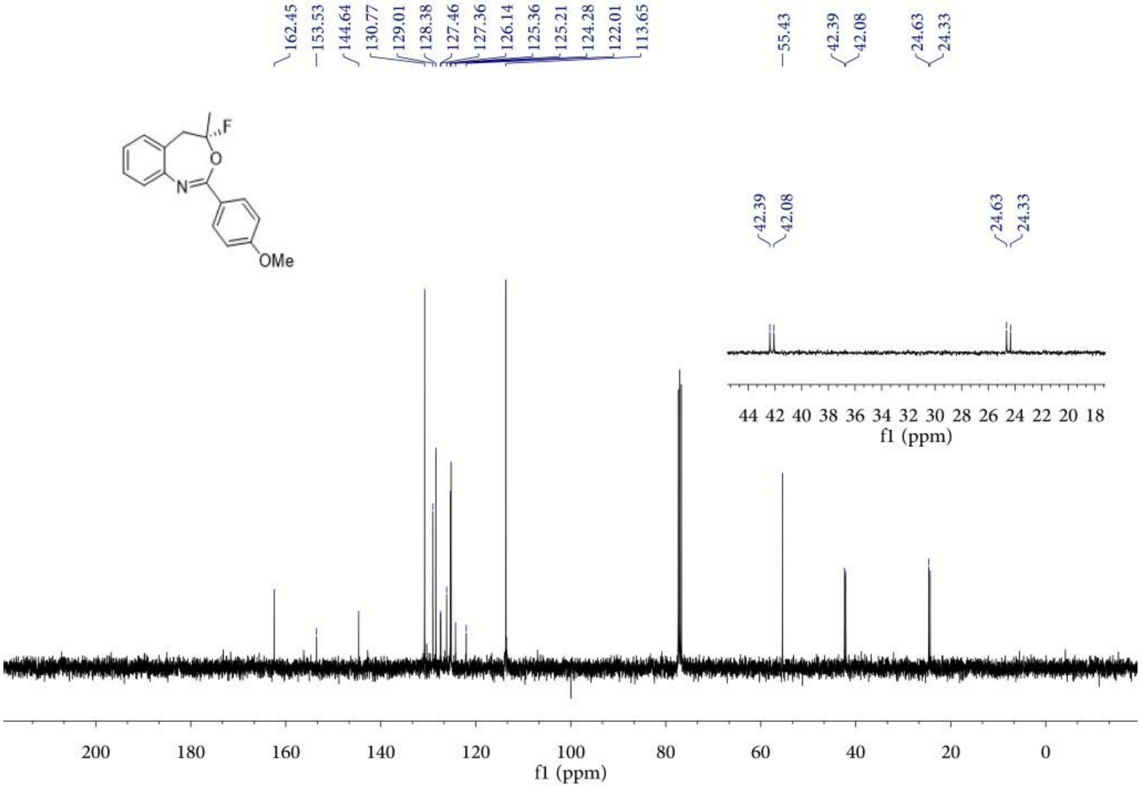

Supplementary Figure 203. ^{19}F NMR spectrum of 50b

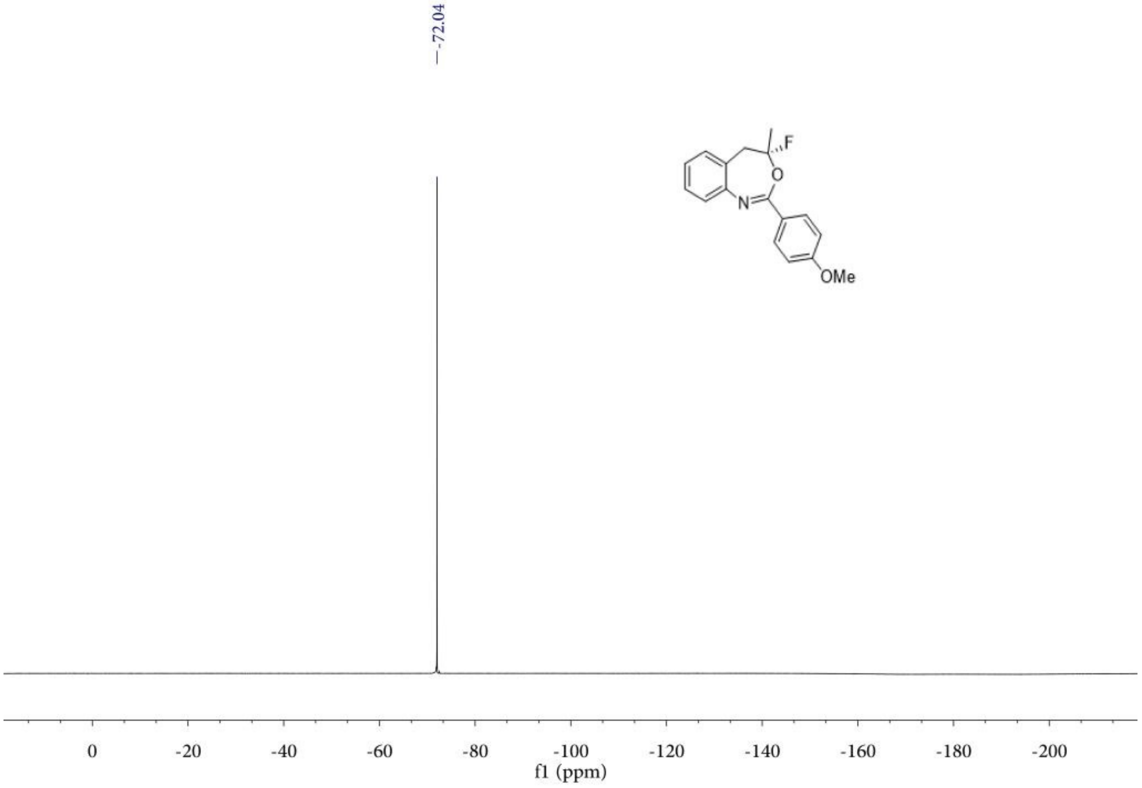

Supplementary Figure 204. ^{1}H NMR spectrum of 51b

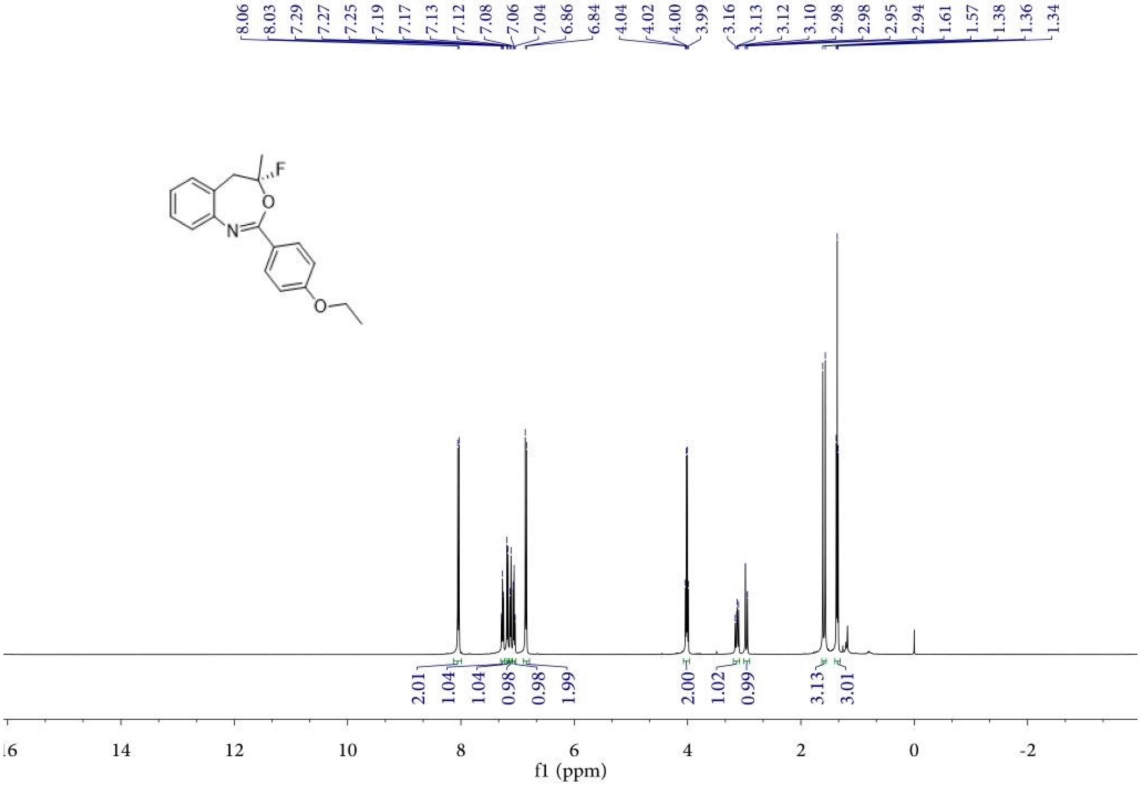

Supplementary Figure 205. ^{13}C NMR spectrum of 51b

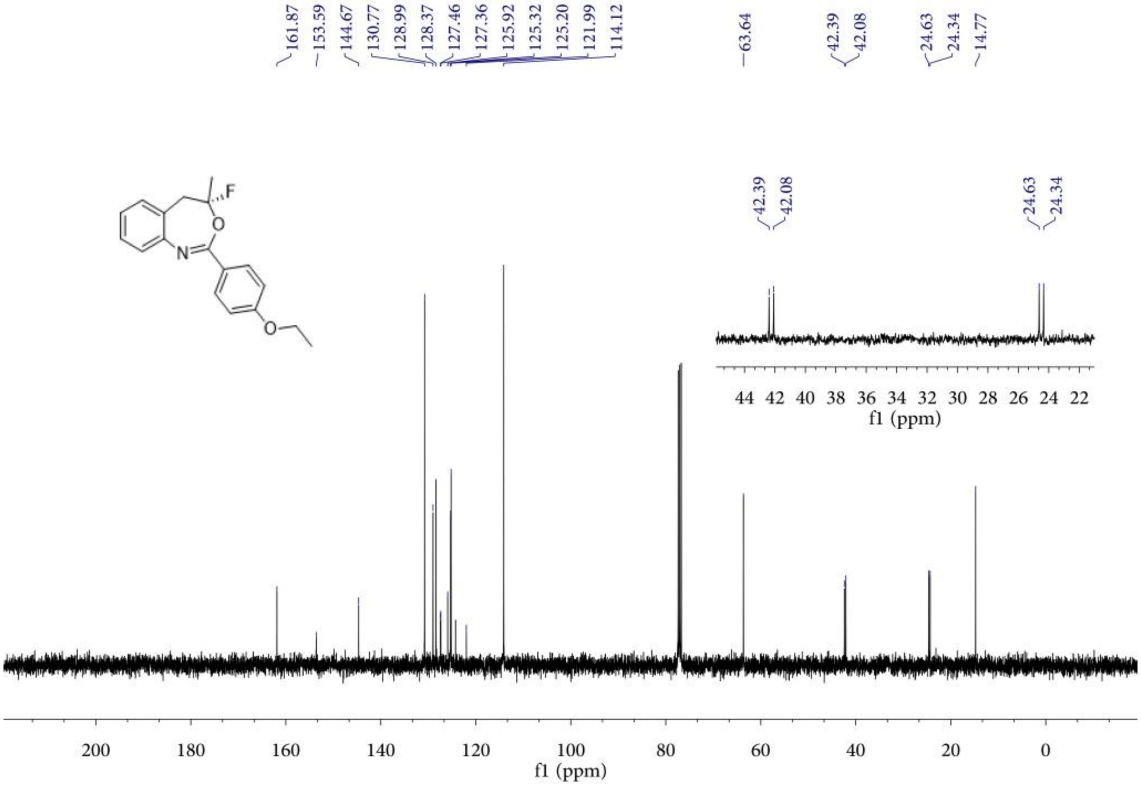

Supplementary Figure 206. ^{19}F NMR spectrum of 51b

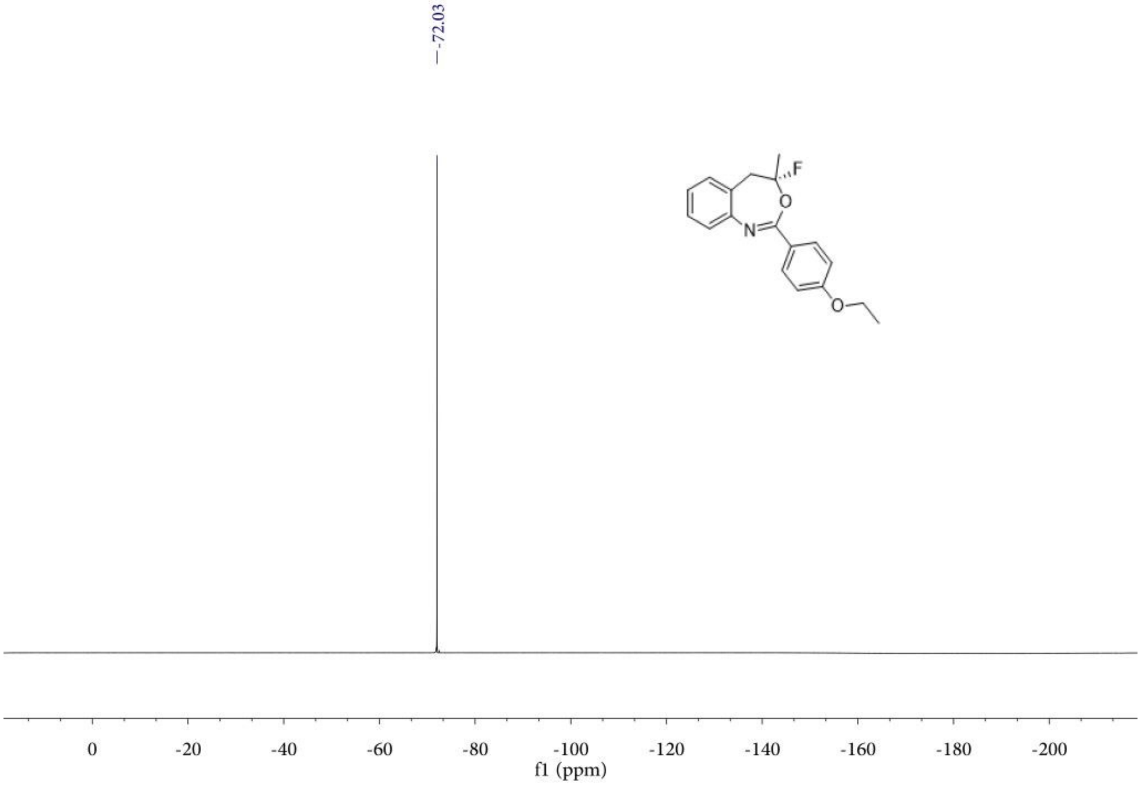

Supplementary Figure 207. ^{1}H NMR spectrum of 52b

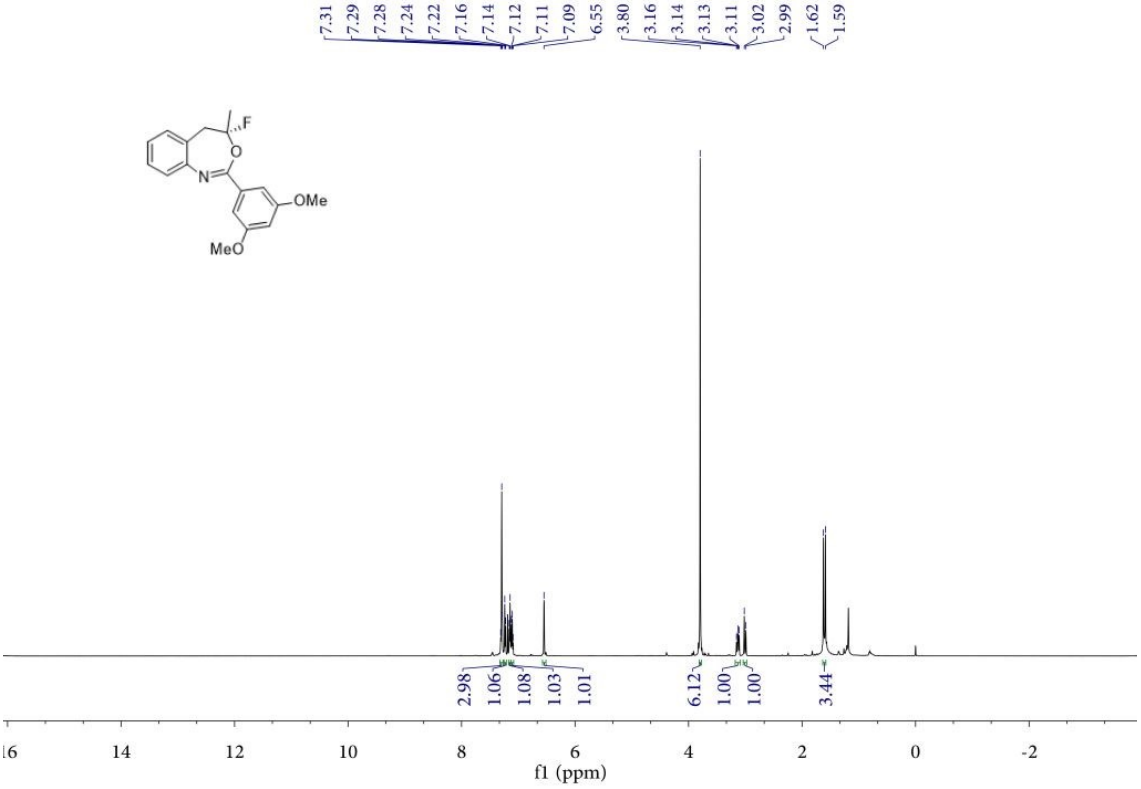

Supplementary Figure 208. ^{13}C NMR spectrum of 52b

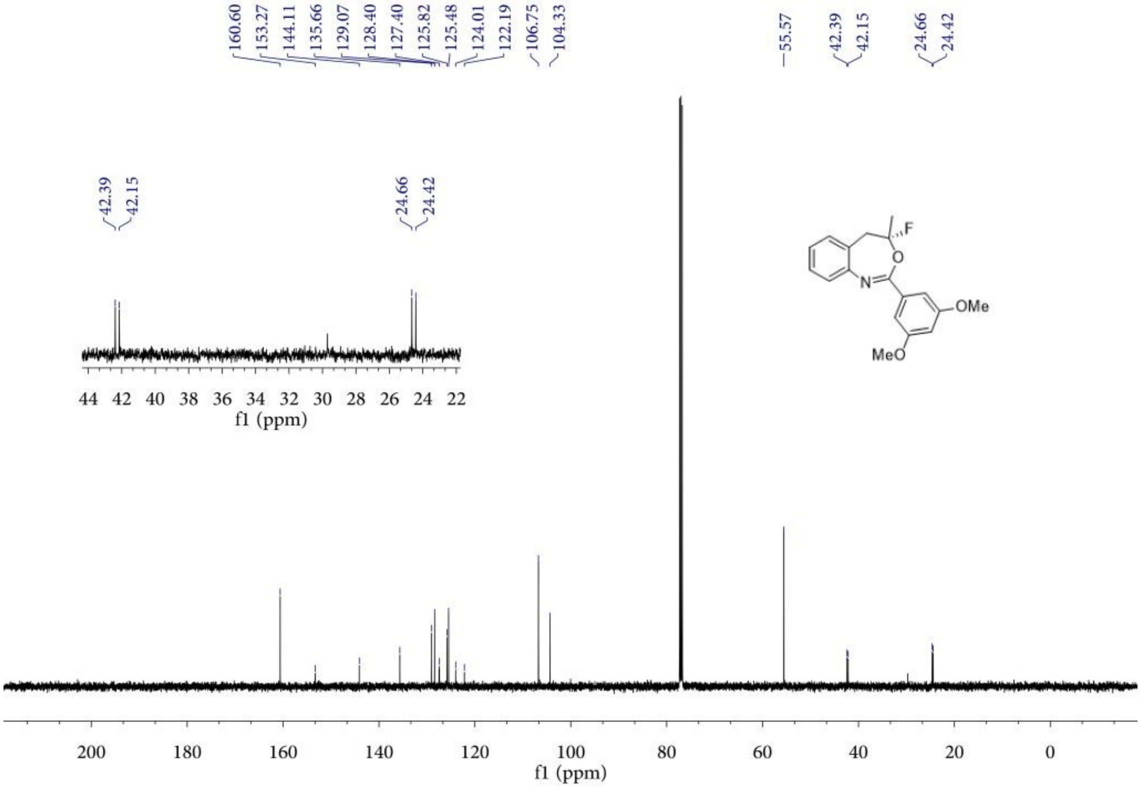

Supplementary Figure 209. ^{19}F NMR spectrum of 52b

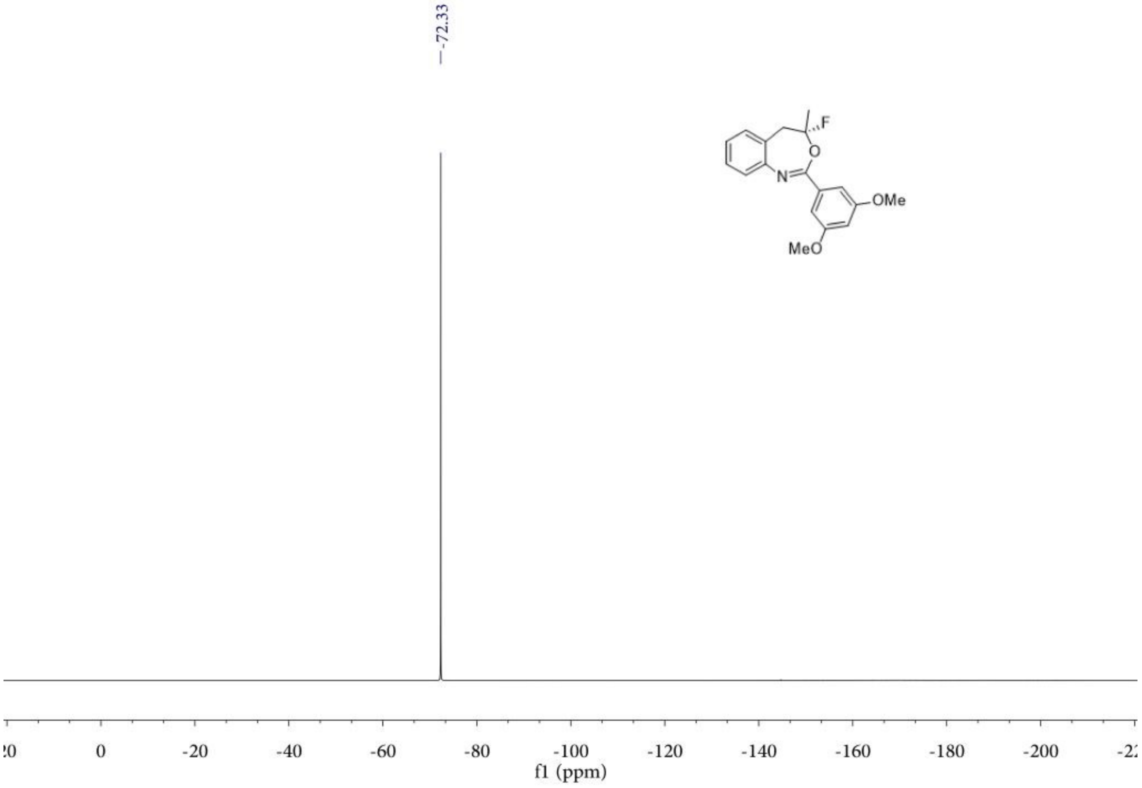

Supplementary Figure 210. ^{1}H NMR spectrum of 53b

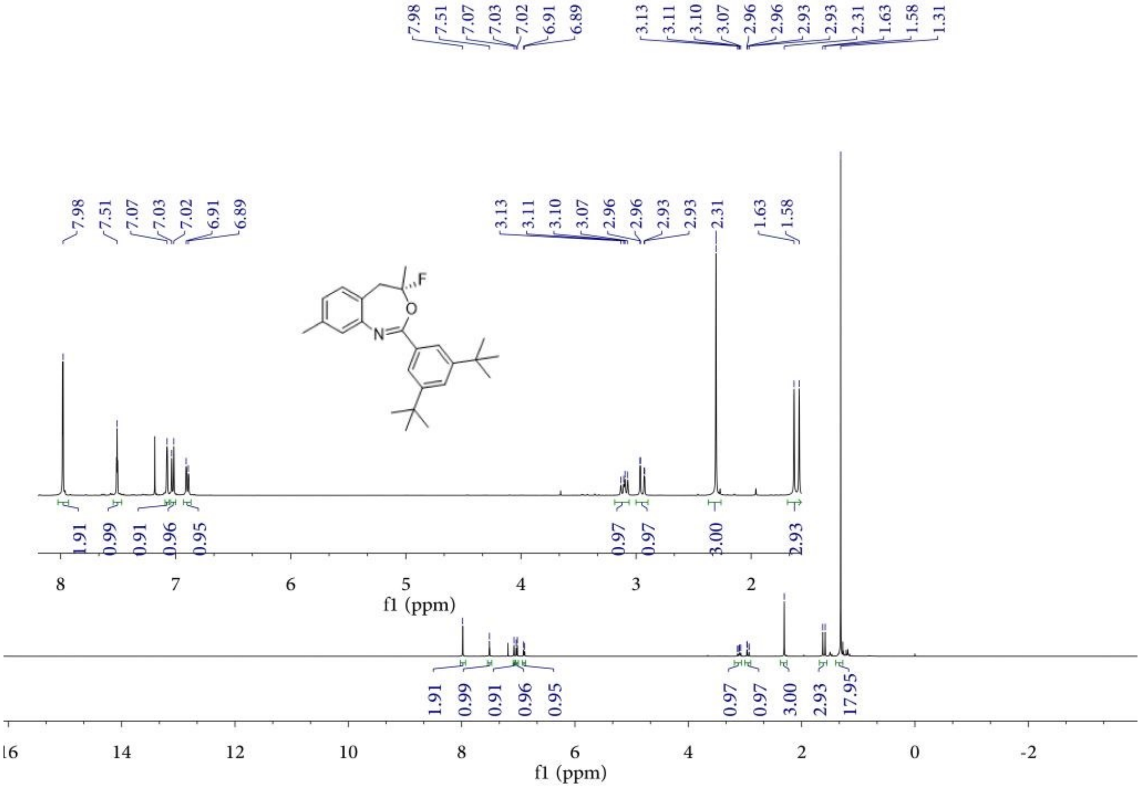

Supplementary Figure 211. ^{13}C NMR spectrum of 53b

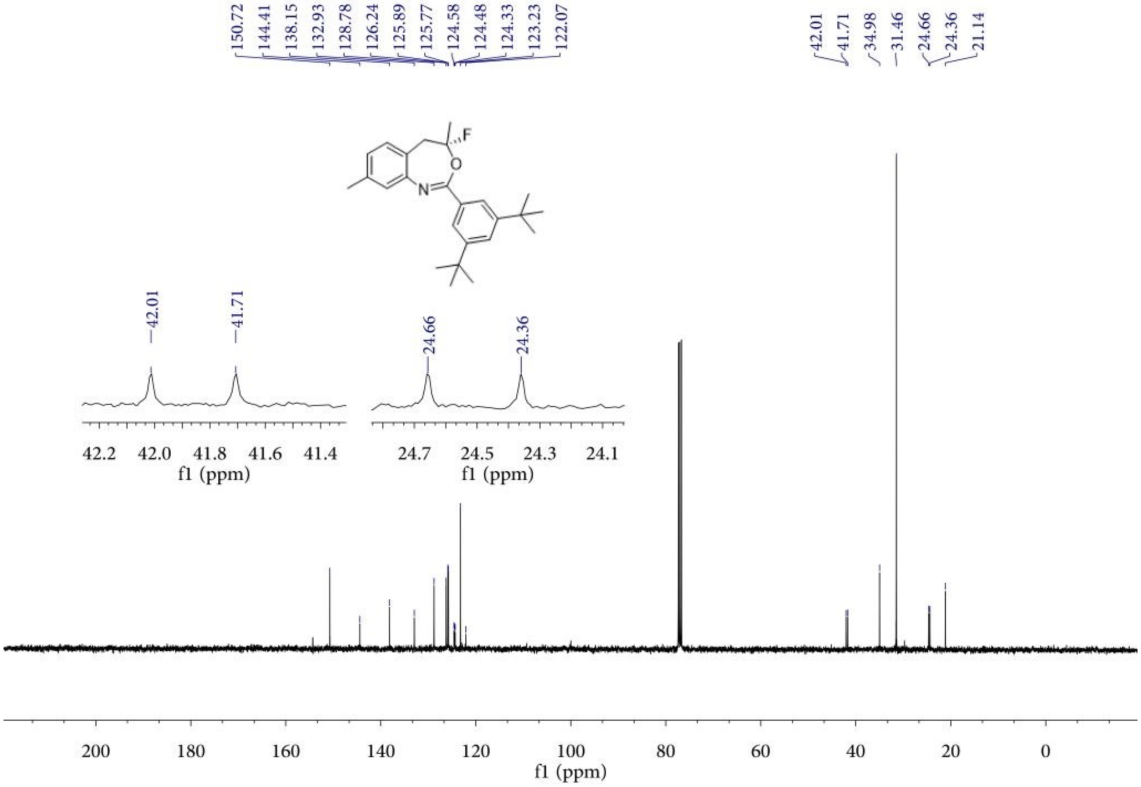

Supplementary Figure 212. ^{19}F NMR spectrum of 53b

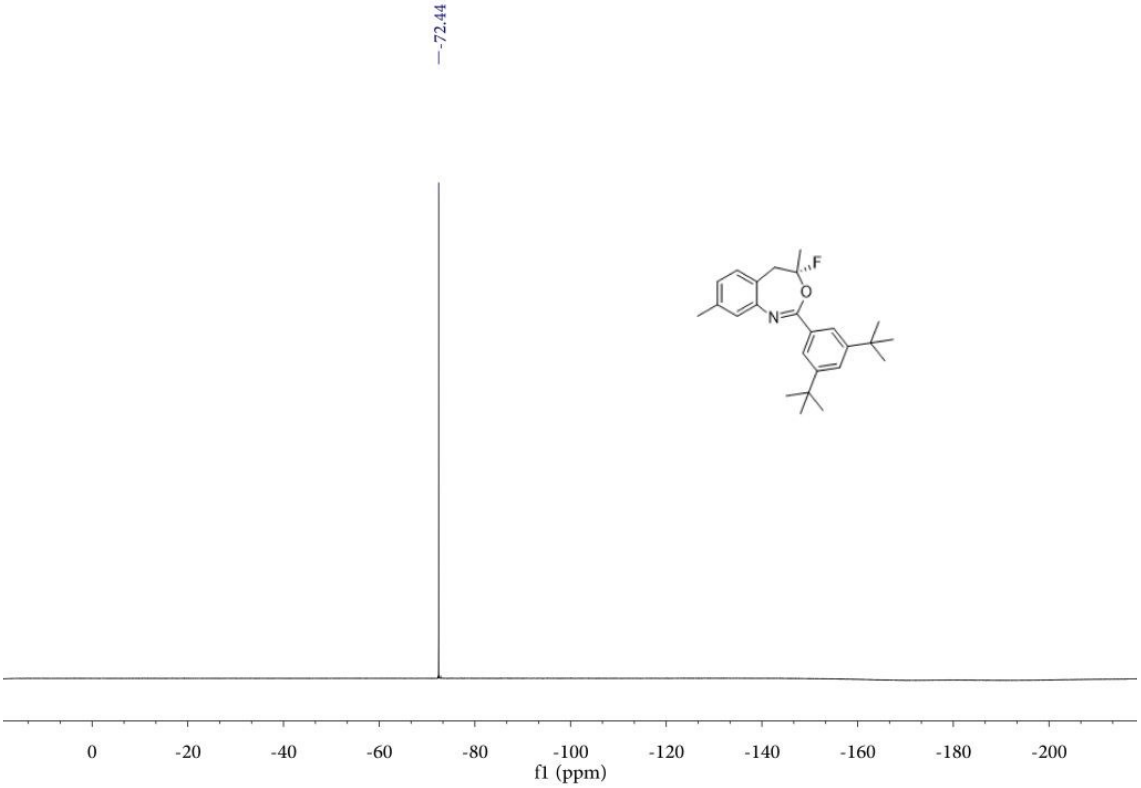

Supplementary Figure 213. ^{1}H NMR spectrum of 54b

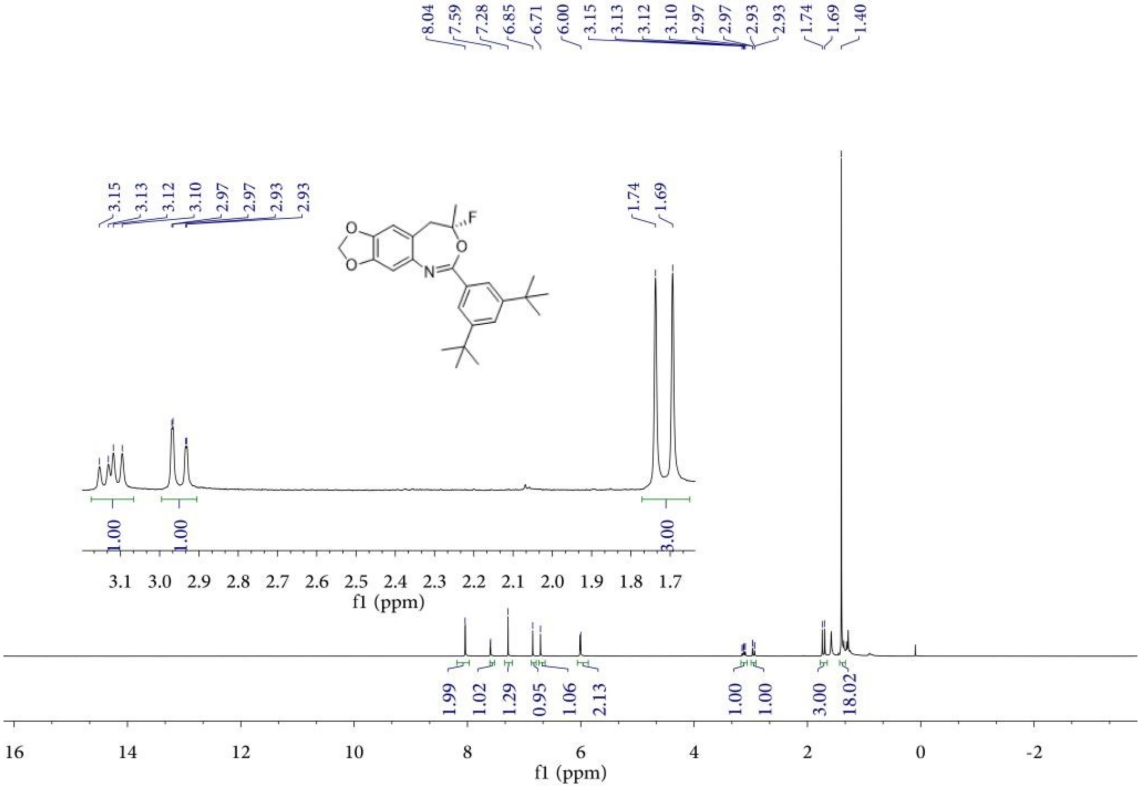

Supplementary Figure 214. ^{13}C NMR spectrum of 54b

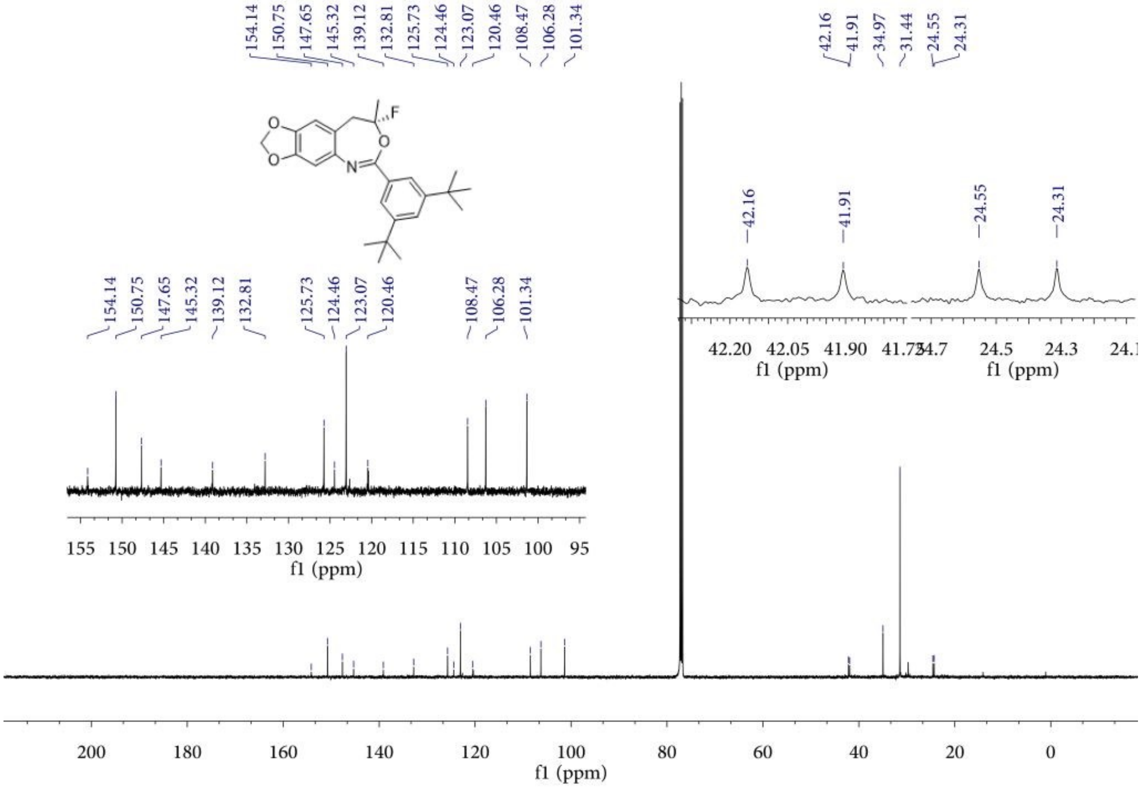

Supplementary Figure 215. ^{19}F NMR spectrum of 54b

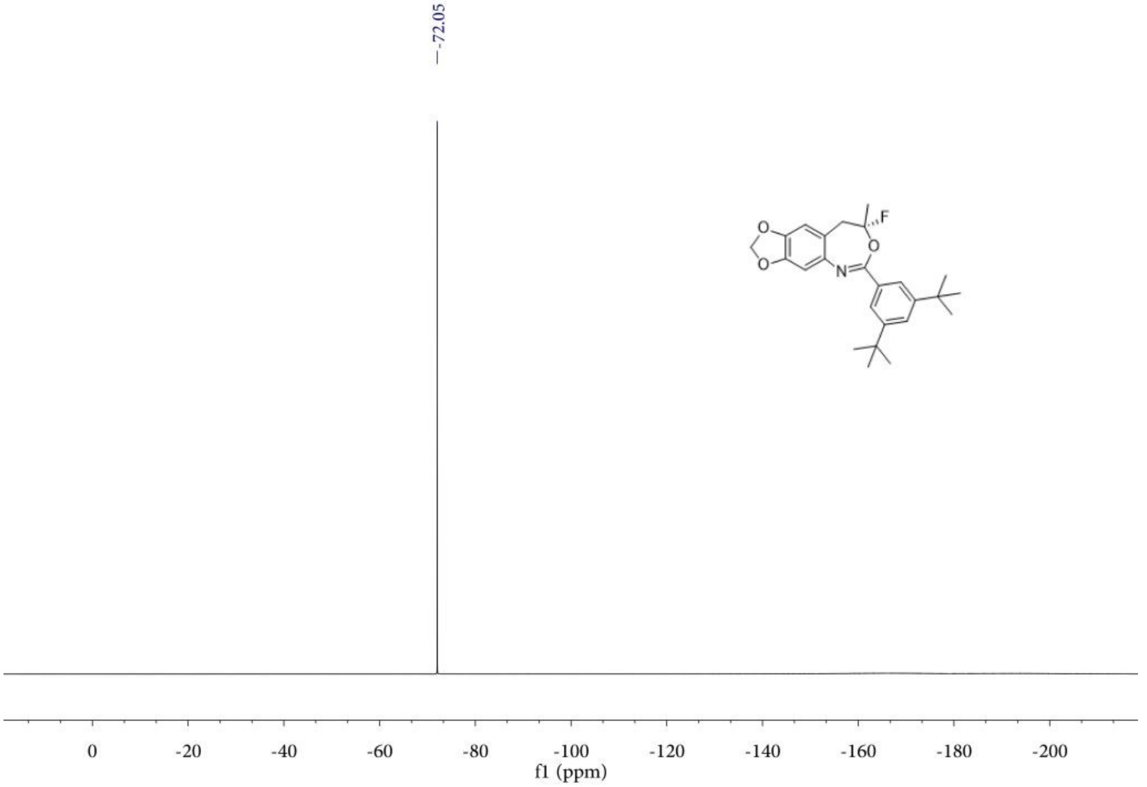

10. Supplementary References

1. Nakajima, M., et al. Structure–activity relationships of substituted oxyoxalamides as inhibitors of the human soluble epoxide hydrolase. J. *Bioorg. Med. Chem*. **22**, 1176-1194 (2014).
2. Banik, S. M., Medley, J. W. & Jacobsen, E. N. Catalytic, asymmetric difluorination of alkenes to generate difluoromethylated stereocenters. Science 353, 51-54 (2016).
3. Uyanik, M.; Yasui, T. & Ishihara, K. Chiral hypervalent iodine-catalyzed enantioselective oxidative Kita spirolactonization of 1-naphthol derivatives and one-pot diastereo-selective oxidation to epoxyspirolactones. *Tetrahedron* **66**, 5841-5851 (2010).
4. Patel, P., et al. Two potent OXE-R antagonists: assignment of stereochemistry. *ACS Med. Chem. Lett.* **5**, 815-819 (2014).
5. Tsujiyama, S. I. & Suzuki K. Preparation of benzocyclobutenone derivatives based on an efficient generation of benzynes. *Org. Synth.* **84**, 272-284 (2007).
6. Haubenreisser, S., et al. Structurally defined molecular hypervalent iodine catalysts for intermolecular enantioselective reactions. *Angew. Chem. Int. Edit.* **55**, 413-417 (2016).
7. Fujita, M., et al. Enantiodifferentiating *endo*-selective oxylactonization of ortho-alk-1-enylbenzoate with a Lactate-derived Aryl-λ^3-iodane. *Angew. Chem. Int. Ed.* **49**, 7068-7071 (2010).
8. Dohi, T., et al. Asymmetric dearomatizing spirolactonization of naphthols catalyzed by spirobiindane-based chiral hypervalent iodine species. *J. Am. Chem. Soc.* **135**, 4558-4566 (2013).
9. Dohi, T., et al. A chiral hypervalent iodine(III) reagent for enantioselective dearomatization of phenols. *Angew. Chem. Int. Ed.* 2008, **47**, 3787-3790 (2008).
10. Soltanzadeh, B., et al. Highly stereoselective intermolecular haloetherification and haloesterification of allyl amides. *Angew. Chem. Int. Ed.* **54**, 9517-9522 (2015).
11. Einaru, S., et al., *trans*-Cyclooctenes as halolactonization catalysts. *Angew. Chem. Int. Ed.* **57**, 13863-13867 (2018).
12. Zhang, D.G., et al. Discovery of novel 5-methyl-1*H*-pyrazole derivatives as potential antiprostate cancer agents: Design, synthesis, molecular modeling, and biological evaluation. *Chem. Biol. Drug. Des.* **91**, 1113–1124 (2018).
13. Mao, Y. J., et al. Pd-catalyzed debenzylation and deallylation of ethers and esters with sodium hydride. *ACS Catal.* **8**, 3016-3020 (2018).
14. Giancotti, G., et al. Rational modifications on a benzylidene-acrylohydrazide antiviral scaffold, synthesis and evaluation of bioactivity against Chikungunya virus. *Eur. J. Med. Chem.* **149**, 56-68 (2018).
15. Dong J.J., et al. SuFEx-based synthesis of polysulfates. Angew. Chem. Int. Edit. **53**, 9466-9470 (2014).
16. Zhu, W.W., et al. Nucleophilic construction of sulfate bonds: simplified access to polysulfates and polysulfonates. *React. Chem. Eng.* **4**, 2074-2080 (2019).
17. Wang, Y.M., et al. Enantioselective halocyclization using reagents tailored for chiral anion phase-transfer catalysis. *J. Am. Chem. Soc.* **134**, 12928-12931 (2012).
