# Supplementary material for: Catalytic asymmetric nucleophilic fluorination using BF3·Et2O as fluorine source and activating reagent
Source: Nat Commun. 2021 Jun 25;12:3957. doi: 10.1038/s41467-021-24278-3 (PMC8233348; doi:10.1038/s41467-021-24278-3)
Supplement: Supplementary file 3 — Reporting Summary [file 41467_2021_24278_MOESM3_ESM.pdf]

nature research

Corresponding author(s): Xianxing Jiang

Last updated by author(s): May 31, 2021

Reporting Summary

Nature Research wishes to improve the reproducibility of the work that we publish. This form provides structure for consistency and transparency in reporting. For further information on Nature Research policies, see our Editorial Policies and the Editorial Policy Checklist.

Statistics

For all statistical analyses, confirm that the following items are present in the figure legend, table legend, main text, or Methods section.

n/a	Confirmed	
☐	☒	The exact sample size (*n*) for each experimental group/condition, given as a discrete number and unit of measurement
☐	☒	A statement on whether measurements were taken from distinct samples or whether the same sample was measured repeatedly
☒	☐	The statistical test(s) used AND whether they are one- or two-sided<br>*Only common tests should be described solely by name; describe more complex techniques in the Methods section.*
☐	☒	A description of all covariates tested
☐	☒	A description of any assumptions or corrections, such as tests of normality and adjustment for multiple comparisons
☒	☐	A full description of the statistical parameters including central tendency (e.g. means) or other basic estimates (e.g. regression coefficient) AND variation (e.g. standard deviation) or associated estimates of uncertainty (e.g. confidence intervals)
☒	☐	For null hypothesis testing, the test statistic (e.g. *F*, *t*, *r*) with confidence intervals, effect sizes, degrees of freedom and *P* value noted<br>*Give P values as exact values whenever suitable.*
☒	☐	For Bayesian analysis, information on the choice of priors and Markov chain Monte Carlo settings
☐	☒	For hierarchical and complex designs, identification of the appropriate level for tests and full reporting of outcomes
☐	☒	Estimates of effect sizes (e.g. Cohen's *d*, Pearson's *r*), indicating how they were calculated

Our web collection on statistics for biologists contains articles on many of the points above.

Software and code

Policy information about availability of computer code

Data collection	All calculations were carried out with the Gaussian 09 software. The B3LYP functional was adopted for all calculations in combination with the D3BJ dispersion correction. For geometry optimization and frequency calculations, the SDD ECP and basis set was used for I and 6-31G(d) for others. The singlet point energy calculations were performed with a larger basis set combination, in which the def2-TZVP basis set was used for I, and 6-311+G(d,p) for others. The SMD implicit solvation model was used to account for the solvation effect of DCE when performing single point energy calculations.
Data analysis	Gibbs free energy profiles (in kcal/mol) for the reaction at B3LYP-D3BJ/def2-TZVP/6-311+G(d,p)/SMD(DCE)//B3LYP-D3BJ/SDD/6-31G(d) leve.

For manuscripts utilizing custom algorithms or software that are central to the research but not yet described in published literature, software must be made available to editors and reviewers. We strongly encourage code deposition in a community repository (e.g. GitHub). See the Nature Research guidelines for submitting code & software for further information.

Data

Policy information about availability of data

All manuscripts must include a data availability statement. This statement should provide the following information, where applicable:

- Accession codes, unique identifiers, or web links for publicly available datasets
- A list of figures that have associated raw data
- A description of any restrictions on data availability

All relevant data are available from the corresponding author upon reasonable request. All the data supporting the findings of this study are available within this article, and supplementary information files. The authors declare that all data generated or analyzed during this study are included in this Article (and its Supplementary Information). The X-ray crystallographic coordinates for the structure of 4b are available free of charge from the Cambridge Crystallographic Data Centre under deposition number CCDC 1960281.

Field-specific reporting

Please select the one below that is the best fit for your research. If you are not sure, read the appropriate sections before making your selection.

☒ Life sciences ☐ Behavioural & social sciences ☐ Ecological, evolutionary & environmental sciences

For a reference copy of the document with all sections, see nature.com/documents/nr-reporting-summary-flat.pdf

Life sciences study design

All studies must disclose on these points even when the disclosure is negative.

Sample size	The absolute configuration of 4b was assigned by X-ray crystallography, and the configuration of all other products was assigned by analogy. The NMR spectra of all the compounds were recorded with BRUKER Ascend 400M & BRUKER Ascend 500M at 25 degree Celsius using Deuterated chloroform as solvent. The ee values were determined by HPLC analysis.
Data exclusions	No data were excluded from the analyses.
Replication	We confirm that all attempts at replication were successful.
Randomization	Author contributions: X.X.J. and W.W.Z. conceived of and directed the project; W.W.Z. developed the catalytic asymmetric fluorinations; W.W.Z., X.Z. and J.K.A. conducted most of the experiments; Y.P.C., J.Y.W. and W.W.Z. conducted the DFT calculations and provided mechanism analysis; X.Y.M., J.K.L, Y.J.Q, H.Z. and J.J.X. helped the conduction of the project; and X.X.J. and W.W.Z. co-wrote the manuscript. We also thank Dr. Chaoxian Yan for his help in DFT calculations.
Blinding	The investigators were not blinded to group allocation during data collection of analysis.

Reporting for specific materials, systems and methods

We require information from authors about some types of materials, experimental systems and methods used in many studies. Here, indicate whether each material, system or method listed is relevant to your study. If you are not sure if a list item applies to your research, read the appropriate section before selecting a response.

Materials & experimental systems

n/a	Involved in the study	
☒	☐	Antibodies
☒	☐	Eukaryotic cell lines
☒	☐	Palaeontology and archaeology
☒	☐	Animals and other organisms
☒	☐	Human research participants
☒	☐	Clinical data
☒	☐	Dual use research of concern

Methods

n/a	Involved in the study	
☒	☐	ChIP-seq
☒	☐	Flow cytometry
☒	☐	MRI-based neuroimaging
